# An Alternative Strategy for Trypanosome Survival in the Mammalian Bloodstream Revealed through Genome and Transcriptome Analysis of the Ubiquitous Bovine Parasite *Trypanosoma (Megatrypanum) theileri*

**DOI:** 10.1093/gbe/evx152

**Published:** 2017-08-14

**Authors:** Steven Kelly, Alasdair Ivens, G. Adam Mott, Ellis O’Neill, David Emms, Olivia Macleod, Paul Voorheis, Kevin Tyler, Matthew Clark, Jacqueline Matthews, Keith Matthews, Mark Carrington

**Affiliations:** 1 Department of Plant Sciences, University of Oxford, United Kingdom; 2 Centre for Immunity, Infection and Evolution and Institute for Immunology and Infection Research, School of Biological Sciences, University of Edinburgh, United Kingdom; 3 Department of Biochemistry, University of Cambridge, United Kingdom; 4 School of Biochemistry and Immunology, Trinity College, Dublin, Ireland; 5 Norwich Medical School, University of East Anglia, Norwich Research Park, Norwich, Norfolk, United Kingdom; 6 Earlham Institute, Norwich Research Park, Norwich, Norfolk, United Kingdom; 7 Moredun Research Institute, Pentlands Science Park, Bush Loan, Penicuik, Midlothian, United Kingdom

**Keywords:** *Trypanosoma theileri*, genome, transcriptome, cell surface components

## Abstract

There are hundreds of *Trypanosoma* species that live in the blood and tissue spaces of their vertebrate hosts. The vast majority of these do not have the ornate system of antigenic variation that has evolved in the small number of African trypanosome species, but can still maintain long-term infections in the face of the vertebrate adaptive immune system. *Trypanosoma theileri* is a typical example, has a restricted host range of cattle and other Bovinae, and is only occasionally reported to cause patent disease although no systematic survey of the effect of infection on agricultural productivity has been performed. Here, a detailed genome sequence and a transcriptome analysis of gene expression in bloodstream form *T. theileri* have been performed. Analysis of the genome sequence and expression showed that *T. theileri* has a typical kinetoplastid genome structure and allowed a prediction that it is capable of meiotic exchange, gene silencing via RNA interference and, potentially, density-dependent growth control. In particular, the transcriptome analysis has allowed a comparison of two distinct trypanosome cell surfaces, *T. brucei* and *T. theileri*, that have each evolved to enable the maintenance of a long-term extracellular infection in cattle. The *T. theileri* cell surface can be modeled to contain a mixture of proteins encoded by four novel large and divergent gene families and by members of a major surface protease gene family. This surface composition is distinct from the uniform variant surface glycoprotein coat on African trypanosomes providing an insight into a second mechanism used by trypanosome species that proliferate in an extracellular milieu in vertebrate hosts to avoid the adaptive immune response.

## Introduction

Trypanosomatid parasites have been extensively studied over the last 100 years since their discovery as the agents of a number of important diseases of the tropics (Steverding 2008, 2014; Azizi etal. 2016; Cox 2016). Major pathogens in this group include African trypanosomes, such as *Trypanosoma brucei*, *T. congolense*, and *T. vivax*, the causal agents of Human and Animal African Trypanosomiasis; *T. cruzi* causing Human American Trypanosomasis or Chagas’ disease, and *Leishmania* species responsible for cutaneous and visceral Leishmaniasis. The importance of these arthropod borne protozoal infections has driven detailed molecular analyses of their immune evasion mechanisms, gene function, biochemistry, and genome structure to the extent that they now represent the best characterized eukaryotic microbes beyond the established unicellular genetic models such as yeasts and *Dictyostelium*. In addition, the distant evolutionary divergence of this group from other eukaryotes is evidenced by the array of molecular and biochemical peculiarities discovered in these organisms that reflect their >1 billion years of separate evolution since the last common ancestor (Cavalier-Smith 2010; Burki 2014). Examples of their molecular novelty include a genome organized to facilitate polycistronic transcription of protein coding genes (Martínez-Calvillo etal. 2003, 2004; Berriman etal. 2005; El-Sayed 2005b; Ivens etal. 2005), ubiquitous *trans*-splicing of a common short exon to the 5′ end of all mRNAs (Sutton and Boothroyd 1986), transcription of some protein coding genes by RNA polymerase I in African trypanosomes (Gunzl etal. 2003), RNA editing of mitochondrial transcripts (Aphasizhev R and Aphasizheva I 2014; Aphasizheva I and Aphasizhev R 2016), and possession of highly divergent chromosomal biology (Ersfeld and Gull 1997; Akiyoshi and Gull 2013), including unique epigenetic modifications (Siegel etal. 2009) and kinetochore proteins (Akiyoshi and Gull 2014). All of these facets have combined to make trypanosomatid organisms of particular interest both in terms of both eukaryotic cell evolution and the host–parasite interactions.

Pathogenic kinetoplastid organisms are intensively studied to understand their biology and disease etiology, but also for their immune evasion mechanisms that enable persistent infections. Molecular interactions involved in evasion of host defenses occur at the kinetoplastid cell surface and as a consequence the composition is specialized for each niche in the host. Thus, African trypanosomes are extracellular and proliferate in blood and tissues fluids, and the cell surface is dominated by a single variant surface glycoprotein (VSG) packed to close to maximum possible density (Schwede etal. 2015). The long-term survival of an infection is dependent on population level antigenic variation of the VSG, underpinned by a genomic repertoire of >1,000 VSG coding sequences, comprising ∼10% of all genes in the nuclear genome (Schwede and Carrington 2010; Horn 2014).

In contrast, both *T. cruzi* and *Leishmania species* proliferate inside host cells, a range of cell types for *T. cruzi*, and macrophages in the case of *Leishmania*. In *T. cruzi*, 6% of nuclear-encoded genes comprise diverse families of mucins (MUC I, MUC II, and SMUG) that encode O-glycosylated proteins expressed in various combinations in the different developmental forms (Buscaglia etal. 2006; Urban etal. 2011). The initial O-glycosylation added during export to the cell surface is further modified at the cell surfaces by trans-sialidases that transfer sialic acid from host proteins to mucin acceptors, the sialylated oligosaccharides are then capped by a terminal α1, 3-galactose. The trans-sialidase gene family has expanded and diverged to a range of functions, many forms having lost catalytic activity (Nardy etal. 2016). There are two other large gene families encoding cell surface proteins, amastins, and MASPs, that are less well-characterized (Jackson 2010; De Pablos and Osuna 2012; dos Santos etal. 2012). In *Leishmania* sp., the cell surface is dominated by “major surface protease” (MSP, also known as GP63 and leishmanolysin) surrounded by a sea of lipophosphoglycan and glycosylinositol phospholipids (Yao etal. 2003; Franco etal. 2012). MSP is a zinc metalloprotease encoded by a gene family that again has diverged to include both catalytically active and inactive members. As illustrated by the separate evolution of intracellular proliferation in *T. cruzi* and *Leishmania*, it is likely that the ability to proliferate intracellularly has evolved many times in different kinetoplastids and thus also likely involved separate evolution of immune evasion strategies.

In addition to the well-characterized kinetoplastids described earlier, there is a large group of mammalian infective trypanosomatid species that are extracellular during proliferation in the mammalian host. In the vast majority of cases, these species do not cause overt disease unless host immunity is compromised. In contrast to African trypanosomes which can infect most species of mammal, these species have a relatively narrow host range. One example is *Trypanosoma (Megatrypanum) theileri* which infects Bovinae (cattle, buffalo, yaks, and some antelopes) and is prevalent in cattle throughout the world (Niak 1978; Matthews etal. 1979; Farrar and Klei 1990; Greco etal. 2000; Rodrigues etal. 2003; Villa etal. 2008; Lee etal. 2010; Garcia etal. 2011). Infection with *T. theileri* normally results in a low parasitaemia that has been shown experimentally to be sustained for at least 12 weeks (Mott etal. 2011) and is almost certainly lifelong. This indicates that the parasite can persist well beyond the impact of a primary immune response, demonstrating that effective immune evasion must be in operation, although the mechanisms that have evolved for evasion are unknown. Since parasite numbers in infected livestock can rapidly increase in immunocompromised, ill, or stressed animals, the parasitaemia in healthy animals is probably limited by the host immune system (Townsend and Duffus 1985) and symptoms of disease are infrequent (Doherty etal. 1993; Seifi 1995). This matches observations for African trypanosomes, such as *T. brucei*, that produce a higher parasitaemia but one that is also limited by both self-imposed quorum sensing and by the host, probably by innate and adaptive immune factors. Surveys in United States and Europe, and most recently United Kingdom, indicate that *T. theileri* is present in >80% cattle but at very low parasitaemias within the blood and tissues of infected animals (Matthews etal. 1979; Schlafer 1979; Farrar and Klei 1990; Mott etal. 2011). *Trypanosoma theileri* is transmitted by tabanid flies, where it undergoes a developmental cycle (Bose and Heister 1993). Infection of cattle is most likely mediated by ingestion of infected flies and also through vertical transmission. Related trypanosomes infect a range of mammals: *T. melophagium* in sheep, spread by keds (Gibson etal. 2010); *T. pestanai* in badgers (Peirce and Neal 1974) spread by fleas (Lizundia etal. 2011); *T. nabiasi* in rabbits (Grewal 1957), and *T. cervi* in deer (Matthews etal. 1977) spread by keds (Bose and Petersen 1991).

Recent work has developed *T. theileri* as a potential vaccine delivery vehicle able to express antigens in sustained infections in recipient cattle and effective immune responses have been successfully generated to an encoded antigen (Mott etal. 2011). These studies demonstrated that gene expression in *T. theileri* is similar to other trypanosomatids with polycistronic transcription of protein coding genes followed by *trans*-splicing, cleavage, and polyadenylation acting to resolve monocistronic mRNAs. Moreover, protein trafficking mechanisms were cross-functional with N-terminal signal sequences and GPI-addition sequences from *T. brucei* correctly targeting proteins to the exterior surface of the cell in *T. theileri*. In contrast, several expression regulatory elements that occur within the intergenic regions of polycistronic transcription in *Trypanosoma brucei* did not enable effective gene expression in *T. theileri*, suggesting that regulatory elements may not be conserved.

Here, we have derived a detailed picture of the genome and transcriptome of *T. theileri* in its mammalian infectious stage and present an analysis of its gene expression profile and a map of its RNA processing sites. These data allow us to predict that *T. theileri* is capable of meiotic exchange, gene silencing via RNA interference, and that it contains conserved machinery for density-dependent growth control or cellular quiescence. In particular, the transcriptome has allowed a comparison of two distinct trypanosome cell surfaces, *T. brucei* and *T. theileri*, that have each evolved to enable the maintenance of a long-term extracellular infection in cattle. Notably, this provides evidence that *T. theileri* exploits a novel immune evasion mechanism distinct from that of the well-characterized African trypanosome paradigm.

## Materials and Methods

### Genome Sequencing and Assembly

*Trypanosoma theileri* was isolated from a primary cell culture derived from a cow from the north west of England. *Trypanosoma theileri* was cultured invitro as in (Mott etal. 2011) and genomic DNA was extracted using Qiagen DNeasy Blood and Tissue kit. Isolated DNA was sequenced using a five library Illumina approach at the Beijing Genomics institute (www.genomics.cn/en/index; last accessed August 23, 2017). The number of reads, read length, and insert size of each library are shown in supplementary table S1, Supplementary Material online. Prior to assembly, reads were subject to quality filtering using trimmomatic (Bolger etal. 2014) to remove low quality bases and read-pairs as well as contaminating adaptor sequences. Sequences were searched for all common Illumina adaptors (the default option) and the settings used for read processing by trimmomatic were “LEADING:10 TRAILING:10 SLIDINGWINDOW:5:15 MINLEN:50.” The quality filtered paired-end reads were then subject to assembly using ALLPATHS-LG (Maccallum etal. 2009) using the default program settings. The resulting assembly was subject to 32 rounds of assembly error correction and gap filling using Pilon (Walker etal. 2014) using the “–fix all” option and setting the expected ploidy to diploid. All filtered 91-bp paired-end reads were mapped to this assembly set using BWA-MEM (Li and Durbin 2010), and read-pairs that did not map to the assembly were isolated and assembled separately using SGA (Simpson and Durbin 2012) using the default parameters. Contigs produced using SGA whose length was >1,000 bp were added into the original assembly and subject to iterative scaffolding using SSPACE (Boetzer etal. 2011). This process of identifying unmapped reads, assembly of unmapped reads, and scaffolding was repeated until no further contigs >1,000 bp were produced. The final draft assembly contained 319 sequences with an N50 515 kb and a total assembly length of 29.8 Mb and an average coverage per assembled contig of ∼105×. This Whole Genome Shotgun project has been deposited at DDBJ/ENA/GenBank under the accession NBCO00000000. The version described in this paper is version NBCO01000000.

### Prediction of Gene Models

The assembled genome of *T. theileri* was subject to gene model prediction using Augustus (Stanke etal. 2006). In brief, an initial set of gene models was predicted using gene prediction parameters inferred by a training gene model parameter using the set of genes currently annotated in the *T. cruzi* genome. These gene model parameters were used to predict a training set of genes in the draft assembly of *T. theileri*. The training set of genes were then used for multiple iterations of prediction and training until prediction converged on a final set of gene models and no further genes could be detected using Augustus. Gene models were also predicted using GeneMarkES (Besemer and Borodovsky 2005). GeneMarkES gene models were only kept if they did not overlap with an existing Augustus derived gene model and were consistent the gene orientation of their direct neighbour genes. The final set of predicted coding sequences comprised 11,312 open reading frames.

### Identification of Polyadenylation and Spliced-Leader Acceptor Sites

*Trypanosoma theileri* was grown as above and total RNA was extracted using the Qiagen RNeasy kit. Isolated RNA was sequenced at the Beijing Genomics institute (www.genomics.cn/en/index; last accessed August 23, 2017) using two different approaches, a conventional TrueSeq protocol and a protocol designed to enrich for 3′ ends of mRNA messages (Fiebig etal. 2015). The number of reads, read length, and insert size of each library are shown in supplementary table S1, Supplementary Material online. The raw reads were used to identify and quantify the polyadenylation and spliced-leader acceptor sites in the genome of *T. theileri* using the SLaP mapper (Fiebig etal. 2014) using the default settings. Raw RNASeq reads are available from EBI ArrayExpress under the accession number E-MTAB-5327.

### Quantification of mRNA Abundance

The conventional TruSeq reads above were also used to quantify the mRNA abundance for each predicted gene in the *T. theileri* genome. Prior to quantification, reads were subject to quality filtering using trimmomatic as described earlier. The quality filtered paired-end reads were then used to quantify the abundance of the predicted gene models using RSEM (Li and Dewey 2011) utilizing the default program parameters. mRNA abundances in *T. brucei* were derived from EBI ArrayExpress E-MTAB-3335 and E-MTAB-5460.

### Inference of Orthologous Gene Groups

The annotated proteins for *Trypanosoma brucei* TREU927, *T. congolense* IL3000, *T. cruzi* CL Brenner Esmereldo-like, *T. rangeli* SC58, *T. grayi* ANR4, *T. vivax* Y486, *Leishmania tarentolae* ParrotTarII, *L. major* Friedlin, *L. infantum* JPCM5, *L. mexicana* MHOMGT2001U1103, *L. donovani* BPK282A1, *L. braziliensis* MHOMBR71973M2269, and *Crithidia fasiculata* CfCl were downloaded from TriTrypDb V8.0 (Aslett etal. 2010). The predicted proteins from *Phytomonas EM1* and *Phytomonas Hart1* were obtained from (Porcel etal. 2014). The predicted proteins from *Phytomonas serpens* were obtained from (Koreny etal. 2012) and for *Trypanoplasma borrelli* from (Carrington et al. 2017). Orthologous gene groups were inferred using OrthoFinder (Emms and Kelly 2015) using default parameters.

### Phylogenetic Inference

Orthologous gene groups containing only single copy genes in all species were selected for inclusion in the phylogenetic analysis. The individual orthologous groups were aligned using MergeAlign (Collingridge and Kelly 2012) and edited to remove all gap-containing columns and columns containing fewer than two character states. MergeAlign was selected as it has previously been shown to increase topological congruity between individual gene trees in multi-gene phylogenomic analyses (Collingridge and Kelly 2012). This resulted in a set of 375 alignments containing ≥ 127 phylogenetically informative ungapped aligned positions in all 19 species. 100 bootstrapped concatenated multi-gene multiple sequence alignments were then constructed from this subset using an equi-sampling strategy. Each bootstrap replicate sampled, at random with replacement, 127 ungapped phylogenetically informative columns from each multiple sequence alignment. This equi-sampling strategy was performed so that each gene contributed equally to the final phylogenetic tree thus preventing longer genes from biasing the result. Thus, each bootstrap replicate contained 47,625 phylogenetically informative, ungapped aligned positions spanning all species. Trees were inferred from each resampled multi-gene alignment using RAxML (Stamatakis 2006) utilizing the PROTGAMMAAUTO model of sequence evolution and using minimum evolution principle (with log corrected scores) implemented in FastME (Desper and Gascuel 2002). The complete data set including all alignments and phylogenetic trees is available from the Zenodo research data repository at https://doi.org/10.5281/zenodo.193020; last accessed August 23, 2017.

## Results

### Genome Sequence, Structure, and Cultured Bloodstream Form Transcriptome

The *Trypanosoma theileri* genome was assembled using end reads from five paired end or jumping DNA libraries with insert sizes ranging from 170 to 5,000 bp (supplementary table S1, Supplementary Material online). The genome contained 319 contigs and scaffolds with an N50 length of 515 kb, a total length of 29.8 Mb, and an average coverage depth of 105-fold (supplementary. fig. S1, Supplementary Material online). Subsequent analysis of the genome revealed that tandem repeats of near-identical genes were often absent from the genome sequence and exhaustive attempts to develop in silico methods to assemble these were not successful. These tandemly repeated gene families remain a problem for a complete analysis of any Euglenozoan genome as many tandem repeats are compressed or absent from sequences assembled by large scale shotgun approaches using short reads, an example being the compression of the tubulin gene loci in the *T. brucei* reference genome (compare Ersfeld etal. 1998 and Hall etal. 2003). After assembly of the *T. theileri* genome, 91.4% of the raw genome reads that had passed quality control mapped back to the assembly indicating that the assembly was relatively complete with respect to the input data. Several assembled scaffolds were equivalent in length to chromosomes present in other kinetoplastids (supplementary fig. S1, Supplementary Material online), this highlighting the presence of clusters of directionally orientated genes indicative of polycistronic transcription units.

The total number of predicted protein coding genes in the genome assembly was 11,312. This complement of protein coding genes was compared with other kinetoplastids by inferring orthologous sequence groups (orthogroups) (Emms and Kelly 2015) (supplementary table S2, Supplementary Material online). The result was a list of orthogroups each containing more than one gene from one or more species. The *T. theileri* proteins fell into 8,398 orthogroups and 9 of these were unique to *T. theileri*, including 5 orthogroups with more than ten members. Transcriptome data derived from bloodstream forms of *T. theileri* growing in axenic culture (supplementary table S1, Supplementary Material online) were used to validate gene models, and transcripts originating unambiguously from 11,033 of the predicted genes was confidently detected. These transcriptome data were also used to map 5′ *trans*-splice sites that act as recipients for the *T. theileri* spliced leader RNA (Rodrigues etal. 2010), the short exon *trans*-spliced onto each mRNA molecule in kinetoplastid organisms. In addition, a dedicated transcriptome library was used to predict 3′ polyadenylation sites (Fiebig etal. 2014, 2015) (supplementary table S1, Supplementary Material online). Together, these data allowed mapping of 5′ *trans*-splice sites to 8,585 genes and polyadenlyation sites to 9,160 genes and defined the processing sites and the untranslated regions (UTRs), which were then further analyzed (supplementary fig. S2, Supplementary Material online). For *trans*-splicing, the sequences 15-bp upstream and 10-bp downstream of the AG dinucleotide defining the splice leader addition site were analyzed and showed little conservation (supplementary fig. S2, Supplementary Material online). The majority of genes (∼9,000) contained one or two splice acceptor sites with the remaining ∼2,000 genes containing between three and nine splice acceptor sites; the length of the 5′ UTR was <200 nucleotides for the vast majority of mRNAs (supplementary fig. S2, Supplementary Material online). As with other kinetoplastids, the number of polyadenylation sites for each predicted gene was generally larger, multiple sites being the norm and the length of the 3′ UTR also showed a greater range. The length distribution of 5′ UTR (0–500 nt) and 3′ UTRs (50–1,500 nt) (supplementary fig. S2, Supplementary Material online) was similar to that measured in *T. brucei* (Kolev etal. 2010).

**Fig. 1.**
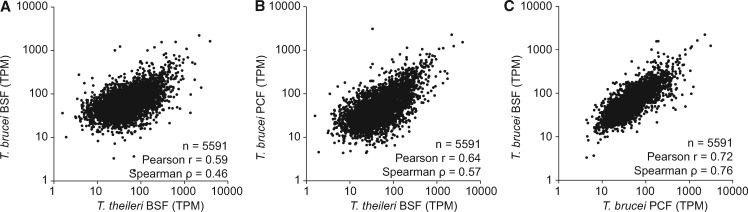
—Comparison of mRNA levels, for 5,591 common single copy genes, between: *Trypanosoma theileri* cultured bloodstream forms (BSF) and (*a*) *T. brucei* cultured BSFs and (*b*) *T. brucei* cultured procyclic (insect) forms (PCF). (*c*) A comparison between the two *T. brucei* life cycle stages. mRNA abundance is shown as Transcripts per Million Transcripts (TPM).

The transcriptome data was used to quantify mRNA levels as transcripts per million transcripts (TPM, the number of transcripts for a given gene per million mRNA transcripts) and this allowed a comparison of expression levels of genes/orthogroups between *T. theileri* and *T. brucei*. This revealed a good correlation between individual mRNA abundances in bloodstream forms of *T. theileri* and *T. brucei* when 5,591 single copy orthologous genes were compared (fig. 1 and supplementary table S2, Supplementary Material online). One unexpected finding was a better correlation between *T. theileri* mammalian bloodstream form (BSF) and *T. brucei* insect procyclic form (PCF) transcripts than found between *T. theileri* BSF and *T. brucei* BSF transcripts (Spearman’s coefficient ρ = 0.46 and ρ = 0.57, respectively, Monte Carlo resampling probability that the difference is chance *P* < 0.001). There are many possible explanations for the similarity, one is cell volume, with both *T. theileri* BSF and *T. brucei* PCF being significantly larger than *T. brucei* BSF. Presumably, cell volume to surface area ratio has an effect on the relative abundance of many hundreds or thousands of cytoplasmic and cytoskeletal mRNAs. Another is that *T. brucei* may exhibit a particularly extreme developmental adaptation as a bloodstream form, exaggerating differences from its insect-dwelling forms, a feature not present in *T. theileri*, or possibly even other African trypanosome species.

**Fig. 2.**
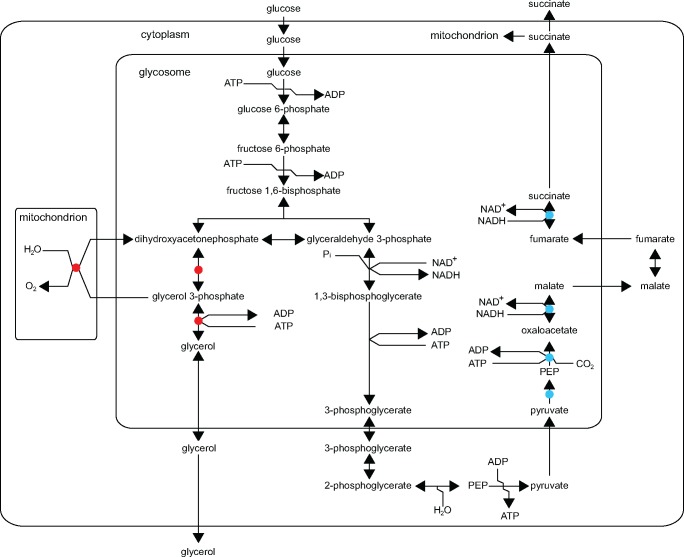
—The measurements from table 1 shown on the glycolytic pathway in trypanosomes. The blue spots indicate enzymes encoded by mRNAs that have >10-fold higher expression (measured as TPM) in *Trypanosoma theileri* BSFs than in *T. brucei* BSFs and the red spots < 10-fold lower expression in *T. theileri*.

Transcripts with the most different abundances in *T. theileri* and *T. brucei* BSFs were identified by selecting genes and orthogroups with more than an 8-fold difference in TPMs between the two species. This revealed that several glycosomal enzymes involved in the fate of the end products of glycolysis were differentially abundant in *T. brucei* when compared with *T. theileri* (table 1). In *T. theileri*, mRNAs for putative glycosomal isoforms of pyruvate phosphate dikinase, phosphoenolpyruvate carboxykinase, and malate dehydrogenase were both 10-fold more abundant than in *T. brucei* BSF while mRNAs encoding glycerol-3-phosphate dehydrogenase, glycerol kinase, and mitochondrial alternative oxidase were at least 10-fold less abundant than in *T. brucei* BSFs. In the proliferating bloodstream form of *T. brucei*, ATP is generated by glycolysis and NAD^+^ is regenerated through a cycle that uses the reduction of dihydroxyacetonephosphate (DHAP) to glycerol-3-phosphate to remove NADH and the alternative oxidase to convert glycerol-3-phosphate back to DHAP independently of NAD(H). Three carbon sugars enter this cycle as DHAP from glycolysis and leave on the conversion of glycerol-3-phosphate to glycerol generating ATP, and with glycerol being secreted. In contrast, in *T. theileri* mRNA expression levels are consistent with the conversion of pyruvate to PEP to oxaloacetate that re-enters the glycosome where it is converted to succinate allowing recovery of NAD^+^, the majority of succinate could then be secreted (fig. 2). This model deduced from differential mRNA levels is consistent with the measured conversion of glucose to succinate in bloodstream form *T. theileri* (van Hellemond etal. 2007). Other than this metabolic distinction, there were no obvious further gross differences when sets of mRNAs corresponding to individual GO terms were compared.

**Table 1:** Comparison of mRNA Expression Levels, Expressed as Transcripts Per Million Transcripts (TPM), for Enzymes Involved in Glycolysis and The Resolution of Products of Glycolysis in *Trypanosoma theileri* Bloodstream Forms (Tt BSF) and *T. brucei* Bloodstream Forms (Tb BSF)

Enzyme	Tb Gene		Tt Gene	Tb BSF	Tt BSF	Tb BSF/Tt BSF	Tt BSF/Tb BSF
Glycerol-3-phosphate dehydrogenase	Tb927.1.1130	Cytosolic	Tth.14.2510	29	17	1.71	0.59
	Tb927.8.3530	Glycosome	Tth.25.1530	1,022	33	30.97	0.03
	Tb927.11.7380	Mitochondrion	Tth.16.2900	100	31	3.23	0.31
Glycerol kinase	Tb927.9.12550	Glycosome	Tth.4.3700	872	48	18.17	0.06
	Tb927.9.12570						
	Tb927.9.12590						
	Tb927.9.12610						
	Tb927.9.12630						
Alternative Oxidase	Tb927.10.7090	Mitochondrion	Tth.8.1700	244	21	11.62	0.09
	Tb927.10.9760						
Pyruvate phosphate dikinase	Tb927.11.6280	Glycosome	Tth.16.1420	41	680	0.06	16.59
Phosphoenolpyruvate carboxykinase	Tb927.2.4210	Glycosome	Tth.23.1750	116	2,421	0.05	20.87
Malate dehydrogenase	Tb927.10.2560	Mitochondrion	Tth.1.1170	72	434	0.17	6.03
	Tb927.10.15410	Glycosome	Tth.3.2160	81	1,394	0.06	17.21
	Tb927.11.11250	Cytosolic	Tth.24.1250	176	826	0.21	4.69
Fumarate hydratase	Tb927.3.4500	Glycosome	Tth.9.3160	28	164	0.17	5.86
	Tb927.11.5050	Mitochondrion	Tth.43.1640	70	43	1.63	0.61
Fumarate reductase	Tb927.5.940		Tth.75.1060	17	251	0.07	14.76
			Tth.88.1120				
	Tb927.5.930	Glycosome	Tth.75.1070	28	220	0.13	7.86
			Tth.75.1080				
	Tb927.10.3650	Mitochondrion	Tth.42.1190	43	56	0.77	1.30
Succinate dehydrogenase	Tb927.8.6580	Mitochondrion	Tth.15.2280	57	190	0.30	3.33
LHR1 haem uptake protein	Tb927.8.6010			47	532	0.09	11.35

Note.—In addition, a comparison of the haem transporter LHR1 is shown at the bottom of the table. Increased abundance in *T. theileri* relative to *T. brucei* is shown in blue and decreased in red.

### The Phylogenetic Position of *T. theileri*

**Fig. 3.**
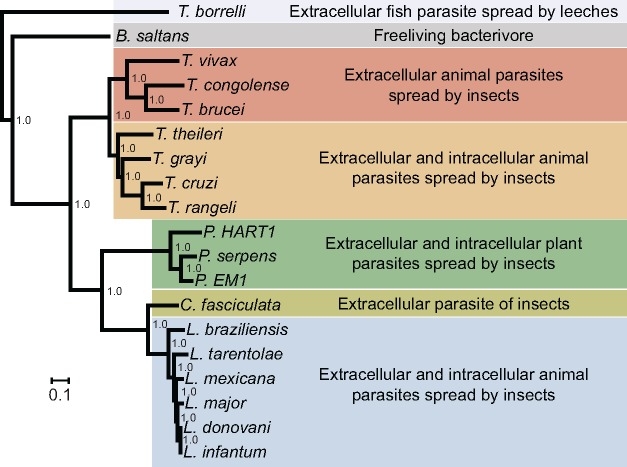
—Phylogenetic relationship between Kinetoplastida with substantial genome/transcriptome sequence availability. The relationship is based on an equal-sampled concatenated alignment of 375 ubiquitously conserved single copy genes (see Materials and Methods). Bootstrap replicate support values (percentages) are shown at internal nodes. Scale bar indicates the number of substitutions per site.

The predicted protein coding genes from *T. theileri* and 18 other kinetoplastids were used to infer orthogroups. Lists of genes in each orthogroup for each species are provided in supplementary table S2, Supplementary Material online. A phylogenomic analysis of 375 ubiquitously conserved single copy genes was conducted to investigate the relationship of *T. theileri* to other kinetoplastids with available genome sequences (fig. 3). This showed that *T. theileri* is positioned at the base of a monophyletic group containing the African crocodilian trypanosome *T. grayi* (Kelly etal. 2014) as well as *T. cruzi* (El-Sayed 2005b) and *T. rangeli* (Stoco etal. 2014). Thus, *T. theileri* is more closely related to these species than it is to African trypanosomes. This multi-gene view of the phylogenetic relationship between trypanosome species is compatible with previous studies that used fewer gene sequences for phylogenetic inference (Hamilton etal. 2004, 2008).

### Interaction with the Mammalian Host and Predicted Cell Surface

**Fig. 4.**
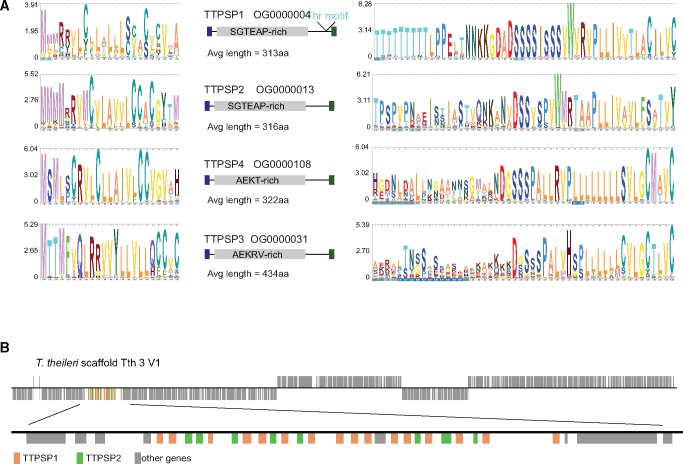
—(*a*) Four novel gene families in *Trypanosoma theileri* encode polypeptides with conserved signal sequences and C-termini. The comparisons were made using Logos (Crooks etal. 2004) after alignment of either the N- or C-termini. (*b*) Genomic structure of a tandem array of interspersed genes encoding members of OG000004 and OG000013, two orthogroups encoding abundant putative cell surface proteins.

Three features characterize the abundant cell surface proteins on kinetoplastids: 1) a significant part of the genome is dedicated to the encoding genes, 2) the proteins and mRNAs are very abundant, for example VSG is >10% of total cell protein, and 3) the majority are attached to the plasma membrane via a GPI-anchor (the major exception being amastins in Leishmania). These three criteria were applied to the *T. theileri* genome and transcriptome data sets to identify genes encoding abundant cell surface proteins (table 2). Homologs of genes present on the surface of other non-African trypanosomes and *Leishmania* sp. were present in *T. theileri*, including a large gene family encoding 216 distinct MSP-related proteins in orthogroup 2 (supplementary table S2, Supplementary Material online, OG0000002) and 36 genes encoding proteins with similarity to the trans-sialidase family (supplementary table S2, Supplementary Material online, OG0000005). In contrast to *T. cruzi*, there were relatively small numbers of amastin (supplementary table S2, Supplementary Material online, OG0000019) and MASP (supplementary table S2, Supplementary Material online, OG0007431) genes, and *T. brucei* VSG-like genes (supplementary table S2, Supplementary Material online, OG0000000) were not found. However, there were four large orthogroups, provisionally named *T. theileri* putative surface protein (TTPSP) 1–4, encoding proteins not present, or not readily detected, in other trypanosomatid species: 556 genes in OG0000004 encoding the TTPSP1 family, 30 in OG0000013 encoding TTPSP2, 145 in OG0000031 encoding TTPSP3, and 61 in OG0000108 encoding TTPSP4. Together, the MSP and four novel orthogroups contained 1,008 genes, representing ∼9% of the genes predicted to be present in the genome. All four novel orthogroups encoded polypeptides with conserved putative N-terminal signal and C-terminal GPI-addition sequences, represented by the logos in figure 4*a*. All four also contained highly conserved residues close to the mature C-terminus that tended to be rich in serine residues but otherwise the mature polypeptides were divergent. The N-terminal signal and C-terminal GPI-anchor addition sequences from the four orthogroups all contained two or more cysteine residues and had some sequence similarity with each other. The TTPSP1 proteins were characterized by a conserved motif, including a run of threonine residues, close to the putative C-terminal GPI-anchor addition site which may be the substrate for O-glycosylation to produce mucin-like proteins, but otherwise had little conservation (fig. 4*a* and supplementary fig. S3*a*, Supplementary Material online). The TTPSP2 proteins were also more conserved toward the C-terminus but without any obvious features (fig. 4*a* and supplementary fig. S3*b*, Supplementary Material online). The TTPSP3 and 4 proteins had some regions with sequence conservation (fig. 4*a* and supplementary fig. S3*c* and *d*, Supplementary Material online) that may reflect conserved secondary structure features. The four TTPSP families are novel and it remains to be determined whether they are simply structural or have additional activities. For example, although the TTPSP gene families had no overt similarity to VSGs, when the predicted structure of some members of TTPSP3 were analyzed, for example Tth.6.1050, they yielded a high confidence (>95%) structural prediction with similarity to the *T. congolense* haptoglobin–haemoglobin receptor (HpHbR) (PDB 4E40) (Higgins etal. 2013) (supplementary fig. S4, Supplementary Material online). The HpHbR is an elongated three helical bundle and it has been proposed that a protein with this structure was the common ancestor of many African trypanosome cell surface proteins including HpHbR and GARP, an abundant protein in the surface coat of the tsetse forms of *T. congolense* (Higgins etal. 2013). Transcription of the vast majority of the genes encoding MSP and the four TTPSP gene families was detected in RNAseq data. However, this RNAseq data was obtained from a population of cells in culture and thus it remains unknown whether all genes are expressed in all cells or whether individual cells express distinct cohorts of these genes.

**Table 2:** *Trypanosoma theileri*: Putative Cell Surface Proteins and Modifying Enzymes

Orthogroup	Gene Copy Number in Orthogroup	Orthogroup mRNA Level (Sum TPM)	mRNA Abundance Relative to Alpha Tubulin	mRNA Rank	*T. theileri*	*T. grayi*	*T. cruzi*	*T. rangeli*	*T. brucei*	*Leishmania*
*T. theileri*										
OG0000130										
Alpha tubulin	?	18,047	1.00	13	✓	✓	✓	✓	✓	✓
Conserved cell surface proteins										
OG0000002										
MSP	216	95,986	5.32	1	✓	✓	✓	✓	✓	✓
OG0000019										
Amastin	2	498	0.03	244	✓	✓	✓	✓	✓	✓
OG0000908										
PSSA-2	5	200	0.01	664	✓	✓	✓	✓	✓	O
OG0007431										
MASP	1	491	0.03	252	✓	✓	✓	✓	O	O
Unique cell surface proteins										
OG0000004										
TTPSP1	556	21,178	1.17	6	✓	O	O	O	O	O
OG0000013										
TTPSP2	304	26,071	1.44	5	✓	O	O	O	O	O
OG0000031										
TTPSP3	145	30,737	1.70	4	✓	O	O	O	O	O
OG0000108										
TTPSP4	61	31,188	1.73	3	✓	O	O	O	O	O
Oligosaccharide modifying enzymes										
OG0000015										
UDP-Gal or UDP-GlcNAc- dependent glycosyltransferase	60	2,631	0.15	39	✓	✓	✓	✓	✓	?
OG0000061										
Galactokinase	2	1,789	0.10	71	✓	✓	✓	✓	✓	✓
OG0000080										
Glycosyl hydrolase/beta fructofuranosidase sucrose	20	512	0.03	239	✓	✓	✓	✓	✓	✓
OG0000005										
Trans-sialidase	36	2,227	0.12	54	✓	✓	✓	✓	✓	O
*T. brucei* for comparison										
OG0000130										
Alpha tubulin	∼20		1	5	✓	✓	✓	✓	✓	✓
OG0000000										
VSG	1		9.93	1	O	O	O	O	✓	O

Note.—Expression levels, in TPM, relative to alpha tubulin mRNA, and in rank of abundance are shown, as is the presence of orthologs in other species.

The composition of the predicted cell surface (of the population) was estimated using transcript abundance as a proxy for protein abundance. To allow an approximate comparison between species, mRNA TPM values were normalized against alpha tubulin mRNA. For example, in bloodstream form *T. brucei* expressing VSG121, the VSG mRNA has a relative abundance to α-tubulin (Relative Abundance to α-Tubulin; RAT) of 9.9 as the TPM value for VSG mRNA is 9.9 times greater than α-tubulin mRNA. In *T. theileri*, five gene families encoding proteins with predicted GPI-anchors were expressed with transcript abundances close to or greater than α-tubulin (table 2). The most abundant was MSP, RAT 5.32, followed by the four novel gene families: TTPSP4, RAT 1.73; TTPSP3, RAT 1.70; TTPSP2, RAT 1.44; and TTPSP1, RAT 1.17. In combination, these transcripts are 11.4 times more abundant than the α-tubulin mRNA, similar to the abundance of VSG mRNA in *T. brucei*. With the caveat that mRNA rather than protein has been measured, it can be predicted that the cell surface of *T. theileri*, irrespective of the precise cohort of genes that are expressed, is a densely packed coat of GPI-anchored proteins dominated by members of these five families.

The potential to modify cell surface molecules with carbohydrate was represented by the abundance of mRNAs for the addition of galactose with 60 genes encoding putative UDP-galactose/UDP-N-acetylglucosamine transferases (OG000015, RAT 0.15) and 2 genes encoding galactokinases (OG000061, 2 genes, RAT 0.10) (table 2). Both are predicted to be involved in mucin biosynthesis but also possibly in the modification of other surface glycans. A family of 36 genes encoding trans-sialidase homologs (OG0000005, RAT 0.12) contained potentially active members and some that are probably cell surface localized as they contain potential GPI-anchor addition sequences. The active site of trans-sialidases is characterized by a GRW motif and, of the 36 sequences, 30 spanned this motif and 9 of this 30 contained the motif G(R/K)W, whereas the majority of the others have a precise deletion of these three residues indicating that they are no longer enzymatically active (supplementary fig. S5, Supplementary Material online).

In MSP, the presence of a conserved HExxHxxGF motif that binds the Zn^++^ ion in the active site was used as a measure of probable proteolytic activity. Of the 216 MSP sequences derived from the *T. theileri* genome, 158 spanned this motif, and of these 126 contained an intact motif (supplementary fig. S6, Supplementary Material online). This observation suggests that the majority of MSP genes encode a potentially active protease, and thus proteolysis may have a role in immune evasion in *T. theileri*.

The vast majority of genes encoding putative cell surface proteins occurred in tandem arrays, some >100 kb, that were distributed throughout the genome assembly (supplementary fig. S1, Supplementary Material online). MSP, TTPSP3, and TTPSP4 genes were present in arrays containing just members of the same orthogroup. In contrast, TTPSP1 and TTPSP2 genes were interspersed in the same arrays (fig. 4*b*). Though it is clear that they represent a significant proportion of the genome, the full extent of the tandem arrays cannot be determined because of difficulties in assembling contigs containing large numbers of closely related genes using short reads.

### *Trypanosoma theileri* Encodes Genes for Utilization of Exogenous Sucrose

A further gene family encoding invertase (glycosyl hydrolase/beta fructofuranosidase; OG0000080, 20 genes) (table 2) was identified that is presumably is involved in acquiring hexose from sucrose in the gut of the insect vector. Although after mating female Tabanids become haematophagous they are still commonly found on flowers and feed on nectar (Kniepert 1980). Although these invertases are related to the soluble extracellular sucrases of *Leishmania* (Gontijo etal. 1996) (supplementary fig. S7, Supplementary Material online), all the copies in the *T. theileri* genome are predicted to be anchored to the cell surface by a GPI-anchor. In *Leishmania*, sucrases are expressed during the promastigote stage (Lyda etal. 2015) and are thought to be important for proliferation of the parasite in the gut of their host insects (Blum and Opperdoes 1994), which also feed on plant juices. The predicted tethering of these sucrase enzymes to the *T. theileri* cell surface, via GPI-anchors, may enable the development of a locally higher glucose concentration at the cell surface where it can be actively transported into the cell. The presence of these sucrase genes in *T. theileri*, and in *Leishmania* sp. (fig. 3) indicates that the invertase gene family must have been lost from other lineages during the diversification of trypanosomes, possibly subsequent to adapting to a vector that does not feed on plant sugars.

### Conserved Features in Trypanosomes

Analysis of the *T. theileri* genome highlights a number of predicted surface proteins conserved among all *Trypanosoma* species. This conserved cell surface group includes: 46 genes encoding adenylyl cyclase homologous to *T. brucei* ESAG4 (OG0000008) (Alexandre etal. 1996) and 17 genes encoding ESAG3 homologs (OG0000025) (Pays etal. 1989), both gene families contain many fragmentary copies as in *T. brucei*. Among other molecules universally conserved, *T. theileri* contains a highly conserved homolog of PSSA-2 (OG0000908) (Jackson etal. 1993), as well as the aforementioned MSP, transialidase, and the amastin families. No apparent orthologs of the *T. brucei* transferrin receptor or the haptoglobin–haemoglobin receptor were detected in the *T. theileri* genome. However, it was found that mRNA encoding a putative haem transporter, LHR1 (OG0006128), was >10-fold higher in *T. theileri* than in *T. brucei* BSFs (table 2) and this may provide haem and iron.

### Host Cell Invasion in *T. cruzi*

A characteristic distinction between different kinetoplastid parasites is the capacity of some species to proliferate as an intracellular form in a variety of mammalian cell types. Although African trypanosomes are exclusively extracellular in all stages of their life cycle, and *T. theileri* and *T. grayi* have only been detected in the extracellular milieu, *T. cruzi* has evolved the ability to invade mammalian cells replicating as intracellular amastigote forms. The molecules that have been described as linked to intracellular invasion by *T. cruzi* include trans-sialidase family members, Trypomastigote small surface antigen (TSSA), DGF, TcSMP, and members of the SAP protein family (Epting etal. 2010; Maeda etal. 2012; Martins etal. 2015). A comparison of genomes of *T. theileri, T. grayi, T. rangeli*, and *T. cruzi* was carried out to explore the conservation of cell surface protein families and, by identifying those unique to *T. cruzi*, potentially necessary for host cell invasion (supplementary table S3, Supplementary Material online). Orthogroups containing *T. cruzi* mucins, SAP, and TSSA are restricted to *T. cruzi*, whereas homologs of the other molecules were present in *T. theileri.* One possible interpretation is that SAP and TSSA evolved to facilitate a unique step cell invasion but there is evidence that many other proteins are involved, including those conserved in noninvading species. Finally, it is possible that one or more of the TTPSP gene families shares a common origin with *T. cruzi* mucins but that they were not placed in the same orthogroup.

### Life Cycle

A recent genome wide survey of genes linked to density-dependent developmental events in the *T. brucei* life cycle identified a cohort of genes involved in quorum sensing and/or quiescence (Mony etal. 2014). It is not known whether *T. theileri* has a mechanism to restrict uncontrolled proliferation, although cell cycle arrest in preparation for developmental progression is a common feature of kinetoplastid life cycles. Analysis of the *T. theileri* genome identified many of the signaling components identified in *T. brucei* that are involved in developmental arrest (Mony and Matthews 2015) including genes including AMPK (Saldivia etal. 2016) and YAK protein kinases (OG0005762 and OG0001009, respectively), protein phosphatase 1 (OG0000045) and an ortholog of the TbRBP7 predicted RNA binding protein (OG0000232), overexpression of which drives premature differentiation to stumpy forms in the mammalian bloodstream. The presence of these genes could indicate a form a density-dependent growth restraint in *T. theileri* operates, or the capacity for cellular quiescence, which is a ubiquitous but diverse feature of eukaryotic life (O’Farrell 2011). Whether this is linked to developmental progression however is unknown. Nonetheless, molecules implicated in other differentiation events in the trypanosome life cycle were detected in the *T. theileri* genome including the tyrosine phosphatase TbPTP1 (OG0000462) (Szoor etal. 2006), the glycosomal serine threonine phosphatase TbPIP39 (OG0000450) (Szoor etal. 2010), and the RNA regulators of development, TbZFP1, 2, and 3 (Walrad etal. 2009). Other signaling molecules linked to developmental events included NrkA/B (Gale and Parsons 1993; Gale etal. 1994; Domingo-Sananes etal. 2015) (OG0000380), which promotes development upon entry into the tsetse fly in *T. brucei*, and RDK1 and RDK2 (Jones etal. 2014) kinases (OG0000116 and OG0000231, respectively) which inhibit differentiation to procyclic forms; MAPK2, associated with proliferation of differentiated procyclic forms in *T. brucei* was also detected (OG0005019) (Muller etal. 2002). Further evidence for a developmental cycle within the arthropod vector is the presence of genes uniquely expressed during, and necessary for, meiosis. Thus, all four of the genes, SPO11 (OG0003147), MND1 (OG0003699), HOP1 (OG0002840), and DMC1 (OG004601) (Peacock etal. 2011) are present in the *T. theileri* genome. With respect to the morphological transformations that accompany the developmental cycle, the trypanosome flagellum attachment zone associated cytoskeletal protein GM6 (OG0001193) (Hayes etal. 2014) was present in *T. theileri*. Each of these analyses provides evidence for a functional developmental cycle in *T. theileri*, distinct from simple mechanical transmission. The predicted underlying molecular controls are also similar to those identified in *T. brucei*.

### Signaling and the Cell Cycle

To explore conservation of the cell cycle machinery, we first analyzed the presence of molecules required for normal cell growth or viability previously characterized in *T. brucei*. Orthologs of the 43 protein kinases that have been found by RNAi analysis in *T. brucei* to be required for normal growth or viability are all present in the *T. theileri* genome (Alsford etal. 2011; Jones etal. 2014). Of genes with a predicted protein kinase function in *T. theileri*, four have no ortholog in *T. brucei*, with Tth.16.1150 (OG0008376) and Tth.10.1250 (OG0002653) having an ortholog in *T. cruzi* but not Leishmania and Phytomonas. Tth.23.1270 (OG0005077) is a further protein kinase absent in the African trypanosomes but present in *T. grayi* as well as *T. theileri*, whereas Tth.37.2060 (OG0002304) has a small open reading frame with other related and longer members of the encoded protein family detected in the genomes of *T. grayi*, *T. congolense*, and *T. vivax*. Progression through the cell cycle is regulated by cyclin-dependent kinases, and all the cyclin and kinase components identified in *T. brucei* were present in the *T. theileri* genome.

### RNA Interference

The RNAi machinery shows variable presence among different kinetoplastid species. To be operational a core set of five proteins are required for RNAi, these being AGO1 (OG0005445), DCL1 (OG0006584), DCL2 (OG0007047), RIF4 (OG0007336), and RIF5 (OG0006482). All of these genes were detected in the *T. theileri* genome, indicating an intact RNAi based gene silencing machinery.

## Discussion

Nearly every vertebrate that has been specifically investigated has been found to harbour one or more trypanosomatid species and most proliferate in the blood and sometimes tissue spaces of their hosts. The best-characterized are *T. brucei* and *T. cruzi* as both can cause human disease. However, these are not typical of the genus as the majority of species have evolved neither VSG-based antigenic variation, as found in African trypanosomes such as *T. brucei*, nor the ability to proliferate inside a host cell like *T. cruzi*. Most are also not overtly pathogenic.

*Trypanosoma theileri* represents a more typical trypanosome where overt disease symptoms are an unusual sequelae of natural infection. It has a narrow host range, infecting cattle, and other Bovinae, and can maintain a low level infection for months and probably for the lifetime of the host. *Trypanosoma theileri* provides an informative contrast to *T. brucei*: although both have evolved the ability to infect cattle in parallel, *T. theileri* is a Stercorarian trypanosome, whereas *T. brucei* is a Salivarian, these clades having separated before the emergence of Bovinae from other mammals (Stevens and Gibson 1999). Unlike *T. theileri*, *T. brucei* is characterized by a large host range including cattle, and it is possible that cattle have only been available as a host since the introduction of domesticated cattle to sub-Saharan Africa in the last 10,000 years, although there are several other Bovinae species among the indigenous fauna. Nonetheless, both trypanosome species can maintain persistent extracellular infections in cattle and, at least in an African context, would often coexist simultaneously in coinfections. Here, a genome and transcriptome of *T. theileri* has been determined to provide an insight into the norm with respect to the trypanosomatid lifestyle and as an informative comparator with more pathogenic and better characterized trypanosomes. The main findings are:

- The structure of the genome and the features of the transcriptome is typical for a kinetoplastid.
- A phylogenetic analysis using sequences of 375 polypeptides universally conserved in trypanosomes confirms earlier work placing *T. theileri* with the Stercorarian trypanosomes, closer to *T. cruzi*, *T. rangeli*, and *T. grayi*, than to Salivarian trypanosomes such as *T. brucei*.
- The core cellular machinery is conserved and comparison of relative transcript abundance with *T. brucei* identified few major differences, the main exception being in the fate of glycolytic end products with possible secretion of succinate in *T. theileri* compared with secretion of glycerol in *T. brucei*, consistent with previous measurements (van Hellemond etal. 2007).
- An analysis of transcript abundance allowed a prediction that the *T. theileri* cell surface is dominated by five polypeptide families, MSP and four novel genes families, TTPSP1 to TTPSP4. Transcripts encoding GPI-anchored trans-sialidases and galactose transferases able to modify N- and O-linked oligosaccharides were abundant.
- A complete life cycle was indicated by the presence of a full complement of genes involved in cell cycle arrest, developmental transitions and meiosis. It can be predicted that *T. theileri* is competent for RNAi.
- A comparison of the genomes of Stercorarian trypanosomes to find putative orthologs of genes implicated in cell invasion in *T. cruzi* identified TSSA and SAP genes being unique to *T. cruzi*. These two gene families may have evolved specifically for host cell invasion.

The structure of the *T. theileri* genome was typical for a kinetoplastid (El-Sayed 2005a). Genes are arranged in closely spaced tandem arrays consistent with polycistronic transcription from occasional start sites and linked processing to monocistronic mRNAs through *trans*-splicing to add a short capped exon at the 5′ end (the spliced leader) and linked cleavage and polyadenylation. This model was supported by the identification of RNA processing sites for spliced leader addition and polyadenylation with the resolution of the transcriptome enabling detection of intergenic sequences as well as mature mRNAs. Further experimental support comes from the efficacy of transgenic dicistronic transcription units in *T. theileri* (Mott etal. 2011).

Analysis of the cellular core machinery encoded in the genome revealed the expected conservation of fundamental cellular processes. The function of the identified genes was analyzed by assigning the encoded polypeptides to orthogroups (Emms and Kelly 2015). An orthogroup is the set of genes descended from a single copy gene in the last common ancestor of the species being analyzed, and thus orthogroups, like orthologs, are a natural unit for comparison between species. To add depth to this analysis, we compared the expression levels of mRNAs from both single copy orthologous genes and shared orthogroups in *T. brucei* and *T. theileri*. This approach compared two species that proliferate at a similar rate in culture and have independently evolved to grow in the same host. With the proviso that mRNA levels are only a proxy for the relative importance of pathways or pathway components, and translational control will certainly be important, the analysis suggested general similarity. However, the main identifiable difference was that the end products of glycolysis differ between the two species with production and possible secretion of succinate in *T. theileri* as opposed to the secretion of glycerol in *T. brucei* bloodstream forms (van Hellemond etal. 2007).

Although the core cellular functions or adaptations to different environments are expected to be similar between the trypanosome species, the most significant differences will lie in their interactions with their hosts including regulation of parasitaemia and immune evasion. These interactions are mediated by components present on trypanosome cell surfaces that are usually characterized by densely packed coats of GPI-anchored proteins and oligo- and/or polysaccharides in some species. In the case of African trypanosomes, the coat is dominated by a single VSG packed to close to maximum possible physical density (Manna etal. 2014; Schwede etal. 2015). Outside African trypanosomes, the best characterized kinetoplast cell surfaces are from *T. cruzi*, the causal agent of human American trypanosomiasis, which proliferates inside a range of host cells, and various *Leishmania* species, the causal agents of a range of diseases and characterized by an intracellular proliferative cycle in macrophages. In *T. cruzi*, 6% of genes encode diverse families of mucins (MUC I, MUC II, and SMUG) that encode O-glycosylated proteins expressed in various developmental forms (Buscaglia etal. 2006). The initial O-glycosylation added during biosynthesis is further modified at the cell surfaces by trans-sialidases that transfer sialic acid from host proteins to mucin acceptors. The modified oligosaccharides are capped by a terminal α1,3-galactose. In addition, there are two other large gene families encoding cell surface proteins, amastins, and MASPs, that are less well-characterized. In *Leishmania*, the cell surface is dominated by MSP (Yao 2010) surrounded by a sea of lipophosphoglycan and glycosylinositol phospholipids (Medina-Acosta etal. 1993; Ilg 2000). To gain insight into the *T. theileri* cell surface, we analyzed the abundant cell surface proteins predicted by mRNA levels. This revealed the presence of a large family of MSP proteins and four other novel families, TTPSP1 to TTPSP4, that are very diverse in sequence except at their N- and C-termini. The MSP family contained potentially proteolytically active members as well as inactive members. Hence, the cell surface composition of MSP and the four novel protein families represents a distinct architecture to the monotonous simplicity of the VSG in *T. brucei*. Although MSP is well-characterized, structural and functional characteristics of the proteins encoded by the other four families were not readily apparent. However, TTPSP1, the most numerous gene family, encoded proteins with runs of approximately ten threonine residues in a location proximal to the putative GPI-anchor and thus plasma membrane. This suggests an extracellular domain that is likely to be O-glycosylated and subsequently modified by cell surface trans-sialidases. The combination of a densely packed coat containing sialic acid modified mucins and proteolytically active MSPs is a potent cocktail and could well enable the parasite to escape immune recognition. Potential MSP substrates include immunoglobulins and complement components attached to the trypanosome surface. The importance of these surface families is reflected in their overall representation in the genome, where the number of genes encoding predicted major surface proteins totals >1,000. However, this represents a minimum estimate since difficulties in the assembly of the genome sequence for tandemly arrayed gene families means that the full extent of these tandem gene arrays is unclear. Nonetheless, it is interesting that the number of genes encoding abundant putative cell surface proteins represent ∼10% of the coding capacity of the genome, similar to the overall contribution of VSG genes to the genome of *T. brucei*. The combined expression level of the mRNAs of the surface proteins predicted in *T. theileri* also approximates to the contribution of the VSG mRNA to the overall transcript abundance in a *T. brucei* cell. However, it is important to note that it is not clear whether the diverse gene families are uniformly expressed in individual cells or different members of the population express distinct representatives of the families. Nonetheless, the genomic organization of these gene families coupled with the detection of expression of the majority of members indicates that most can be expressed and that the cell surface is complex.

Although the vast majority are detected as expressed, the genomic arrangement of TTPSP genes into tandem arrays is reminiscent of the arrays of silent VSG gene arrays in *T. brucei*. The TTPSP and VSG gene families also share other similar features. For example, both the *T. theileri* gene families and the *T. brucei* VSGs have moderately well-conserved N-terminal signal and C-terminal GPI-anchor addition sequences but are highly divergent for nearly all of the mature polypeptide sequence. Moreover, some members of TTPSP2 have confident predictions for a three helical bundle structure present in several African trypanosome cell surface proteins and proposed to be the origin of the VSG N-terminal domain fold (Higgins etal. 2013). The presence of proteins with this fold in both African trypanosomes and *T. theileri* indicates that three helical bundle cell surface proteins were present in the common ancestor of *T. theileri* and *T. brucei*. With these observations in mind, it is possible to speculate that the key step in the evolution of VSG-based immune evasion that evolved in African trypanosomes was the evolution of RNA polymerase I transcription of a proto-VSG from a pre-existing diverse gene family. Such events have been suggested before (Jackson and Barry 2012; Manna etal. 2014) but this the first evidence that three helical bundle proteins predated VSGs.

VSG expression in one of multiple telomeric sites (Horn 2014) and the coevolution of diverse expression associated gene families might have enabled the large host range exhibited by *T. brucei.* In contrast, the more restricted host range of *T. theileri* probably results from one or more essential molecular interactions with host macromolecules that are conserved in Bovinae but have diverged in other mammal families. With the current state of knowledge, it is not possible to predict what these interactions are from genome and transcriptome sequence data alone.

Interrogation of the *T. theileri* genome identified genes encoding orthologs of several proteins with experimentally determined functions in the developmental cycle of *T. brucei*. Molecules linked to quorum sensing and cellular quiescence were identified, as were molecules associated with life in the arthropod vector, including those associated with sexual exchange. The implication is that *T theileri* is normally competent for a full developmental cycle in an arthropod vector, most frequently tabanid flies although ticks have also been proposed to be competent for transmission (Morzaria etal. 1986). Moreover, the conservation of molecules linked to the morphological events and developmentally regulated alteration in cell surface proteins was evidenced by the presence of the GM6 and PSSA2, both of which are present throughout the genus. Interestingly, although the genome and transcriptome of the *T. theileri* is distinct from *T. brucei*, a regulator of stumpy form development in *T. brucei* in the bloodstream, RBP7, is also present and syntenic in *T. theileri*, with two closely related isoforms present in tandem, as in *T. brucei*. The master regulator of development through the tsetse fly, RBP6, is also present and syntenic in *T. theileri*. Taken together, this suggests that conserved regulatory mechanisms underly the developmental cycle of trypanosome parasites, which nonetheless differ in their developmental morphotypes.

Finally, one feature of kinetoplastid parasites that has been either retained or lost in the different representatives is the presence of a functional RNAi machinery. This is defined by the presence of five core proteins, AGO, DCL1, DCL2, RIF 4, and RIF5. All of these components are detectable within the *T. theileri* genome, as in *T. grayi*, but unlike *T. cruzi* and *T. rangeli* where it is absent. The presence of the RNAi machinery, the genome sequence, and the amenability to culture means that many of the predictions and hypotheses above will be testable in the future by laboratory manipulation.

In conclusion, this analysis has provided evidence for an alternative means to evade the mammalian immune systems as a replicative extracellular trypanosome distinct from the VSG-based antigenic variation characterized in African trypanosomes. Particularly, these successful parasites occupy the same host, the same niche, and commonly at the same time as the pathogenic African trypanosomes. The respective evolutionary trade-offs between immune evasion mechanisms, level of parasitaemia in the host, and transmission efficiency using distinct arthropod vectors in each of these trypanosome species represents an interesting area of comparative further study.

## Supplementary Material

Supplementary data are available at *Genome Biology and Evolution* online.

## Supplementary Material

Supplementary figures_1-4

Supplementary figure_5

Supplementary figure_6

Supplementary figure_7

Supplementary materials

Supplementary table_1

Supplementary table_2

Supplementary table_3
