# Supplementary material for: An Alternative Strategy for Trypanosome Survival in the Mammalian Bloodstream Revealed through Genome and Transcriptome Analysis of the Ubiquitous Bovine Parasite Trypanosoma (Megatrypanum) theileri
Source: Genome Biol Evol. 2017 Aug 14;9(8):2093–109. doi: 10.1093/gbe/evx152 (PMC5737535; doi:10.1093/gbe/evx152)
Supplement: Supplementary figures_1-4 [file evx152_suppfigs_1-4.pdf]

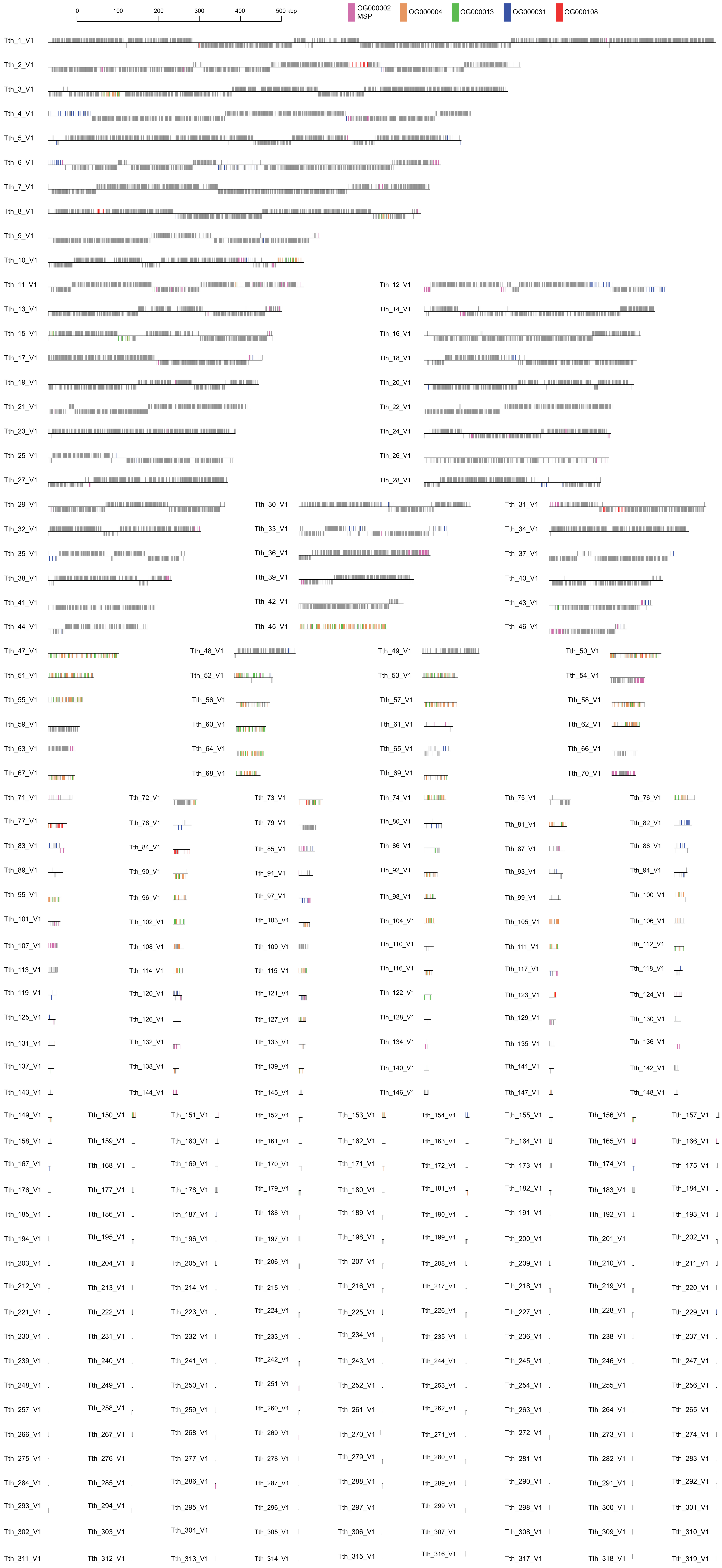

0
100
200
300
400
500 kbp
OG000002 MSP
OG000004
OG000013
OG000031
OG000108
Tth_1_V1
Tth_2_V1
Tth_3_V1
Tth_4_V1
Tth_5_V1
Tth_6_V1
Tth_7_V1
Tth_8_V1
Tth_9_V1
Tth_10_V1
Tth_11_V1
Tth_12_V1
Tth_13_V1
Tth_14_V1
Tth_15_V1
Tth_16_V1
Tth_17_V1
Tth_18_V1
Tth_19_V1
Tth_20_V1
Tth_21_V1
Tth_22_V1
Tth_23_V1
Tth_24_V1
Tth_25_V1
Tth_26_V1
Tth_27_V1
Tth_28_V1
Tth_29_V1
Tth_30_V1
Tth_31_V1
Tth_32_V1
Tth_33_V1
Tth_34_V1
Tth_35_V1
Tth_36_V1
Tth_37_V1
Tth_38_V1
Tth_39_V1
Tth_40_V1
Tth_41_V1
Tth_42_V1
Tth_43_V1
Tth_44_V1
Tth_45_V1
Tth_46_V1
Tth_47_V1
Tth_48_V1
Tth_49_V1
Tth_50_V1
Tth_51_V1
Tth_52_V1
Tth_53_V1
Tth_54_V1
Tth_55_V1
Tth_56_V1
Tth_57_V1
Tth_58_V1
Tth_59_V1
Tth_60_V1
Tth_61_V1
Tth_62_V1
Tth_63_V1
Tth_64_V1
Tth_65_V1
Tth_66_V1
Tth_67_V1
Tth_68_V1
Tth_69_V1
Tth_70_V1
Tth_71_V1
Tth_72_V1
Tth_73_V1
Tth_74_V1
Tth_75_V1
Tth_76_V1
Tth_77_V1
Tth_78_V1
Tth_79_V1
Tth_80_V1
Tth_81_V1
Tth_82_V1
Tth_83_V1
Tth_84_V1
Tth_85_V1
Tth_86_V1
Tth_87_V1
Tth_88_V1
Tth_89_V1
Tth_90_V1
Tth_91_V1
Tth_92_V1
Tth_93_V1
Tth_94_V1
Tth_95_V1
Tth_96_V1
Tth_97_V1
Tth_98_V1
Tth_99_V1
Tth_100_V1
Tth_101_V1
Tth_102_V1
Tth_103_V1
Tth_104_V1
Tth_105_V1
Tth_106_V1
Tth_107_V1
Tth_108_V1
Tth_109_V1
Tth_110_V1
Tth_111_V1
Tth_112_V1
Tth_113_V1
Tth_114_V1
Tth_115_V1
Tth_116_V1
Tth_117_V1
Tth_118_V1
Tth_119_V1
Tth_120_V1
Tth_121_V1
Tth_122_V1
Tth_123_V1
Tth_124_V1
Tth_125_V1
Tth_126_V1
Tth_127_V1
Tth_128_V1
Tth_129_V1
Tth_130_V1
Tth_131_V1
Tth_132_V1
Tth_133_V1
Tth_134_V1
Tth_135_V1
Tth_136_V1
Tth_137_V1
Tth_138_V1
Tth_139_V1
Tth_140_V1
Tth_141_V1
Tth_142_V1
Tth_143_V1
Tth_144_V1
Tth_145_V1
Tth_146_V1
Tth_147_V1
Tth_148_V1
Tth_149_V1
Tth_150_V1
Tth_151_V1
Tth_152_V1
Tth_153_V1
Tth_154_V1
Tth_155_V1
Tth_156_V1
Tth_157_V1
Tth_158_V1
Tth_159_V1
Tth_160_V1
Tth_161_V1
Tth_162_V1
Tth_163_V1
Tth_164_V1
Tth_165_V1
Tth_166_V1
Tth_167_V1
Tth_168_V1
Tth_169_V1
Tth_170_V1
Tth_171_V1
Tth_172_V1
Tth_173_V1
Tth_174_V1
Tth_175_V1
Tth_176_V1
Tth_177_V1
Tth_178_V1
Tth_179_V1
Tth_180_V1
Tth_181_V1
Tth_182_V1
Tth_183_V1
Tth_184_V1
Tth_185_V1
Tth_186_V1
Tth_187_V1
Tth_188_V1
Tth_189_V1
Tth_190_V1
Tth_191_V1
Tth_192_V1
Tth_193_V1
Tth_194_V1
Tth_195_V1
Tth_196_V1
Tth_197_V1
Tth_198_V1
Tth_199_V1
Tth_200_V1
Tth_201_V1
Tth_202_V1
Tth_203_V1
Tth_204_V1
Tth_205_V1
Tth_206_V1
Tth_207_V1
Tth_208_V1
Tth_209_V1
Tth_210_V1
Tth_211_V1
Tth_212_V1
Tth_213_V1
Tth_214_V1
Tth_215_V1
Tth_216_V1
Tth_217_V1
Tth_218_V1
Tth_219_V1
Tth_220_V1
Tth_221_V1
Tth_222_V1
Tth_223_V1
Tth_224_V1
Tth_225_V1
Tth_226_V1
Tth_227_V1
Tth_228_V1
Tth_229_V1
Tth_230_V1
Tth_231_V1
Tth_232_V1
Tth_233_V1
Tth_234_V1
Tth_235_V1
Tth_236_V1
Tth_238_V1
Tth_237_V1
Tth_239_V1
Tth_240_V1
Tth_241_V1
Tth_242_V1
Tth_243_V1
Tth_244_V1
Tth_245_V1
Tth_246_V1
Tth_247_V1
Tth_248_V1
Tth_249_V1
Tth_250_V1
Tth_251_V1
Tth_252_V1
Tth_253_V1
Tth_254_V1
Tth_255_V1
Tth_256_V1
Tth_257_V1
Tth_258_V1
Tth_259_V1
Tth_260_V1
Tth_261_V1
Tth_262_V1
Tth_263_V1
Tth_264_V1
Tth_265_V1
Tth_266_V1
Tth_267_V1
Tth_268_V1
Tth_269_V1
Tth_270_V1
Tth_271_V1
Tth_272_V1
Tth_273_V1
Tth_274_V1
Tth_275_V1
Tth_276_V1
Tth_277_V1
Tth_278_V1
Tth_279_V1
Tth_280_V1
Tth_281_V1
Tth_282_V1
Tth_283_V1
Tth_284_V1
Tth_285_V1
Tth_286_V1
Tth_287_V1
Tth_288_V1
Tth_289_V1
Tth_290_V1
Tth_291_V1
Tth_292_V1
Tth_293_V1
Tth_294_V1
Tth_295_V1
Tth_296_V1
Tth_297_V1
Tth_299_V1
Tth_298_V1
Tth_300_V1
Tth_301_V1
Tth_302_V1
Tth_303_V1
Tth_304_V1
Tth_305_V1
Tth_306_V1
Tth_307_V1
Tth_308_V1
Tth_309_V1
Tth_310_V1
Tth_311_V1
Tth_312_V1
Tth_313_V1
Tth_314_V1
Tth_315_V1
Tth_316_V1
Tth_317_V1
Tth_318_V1
Tth_319_V1

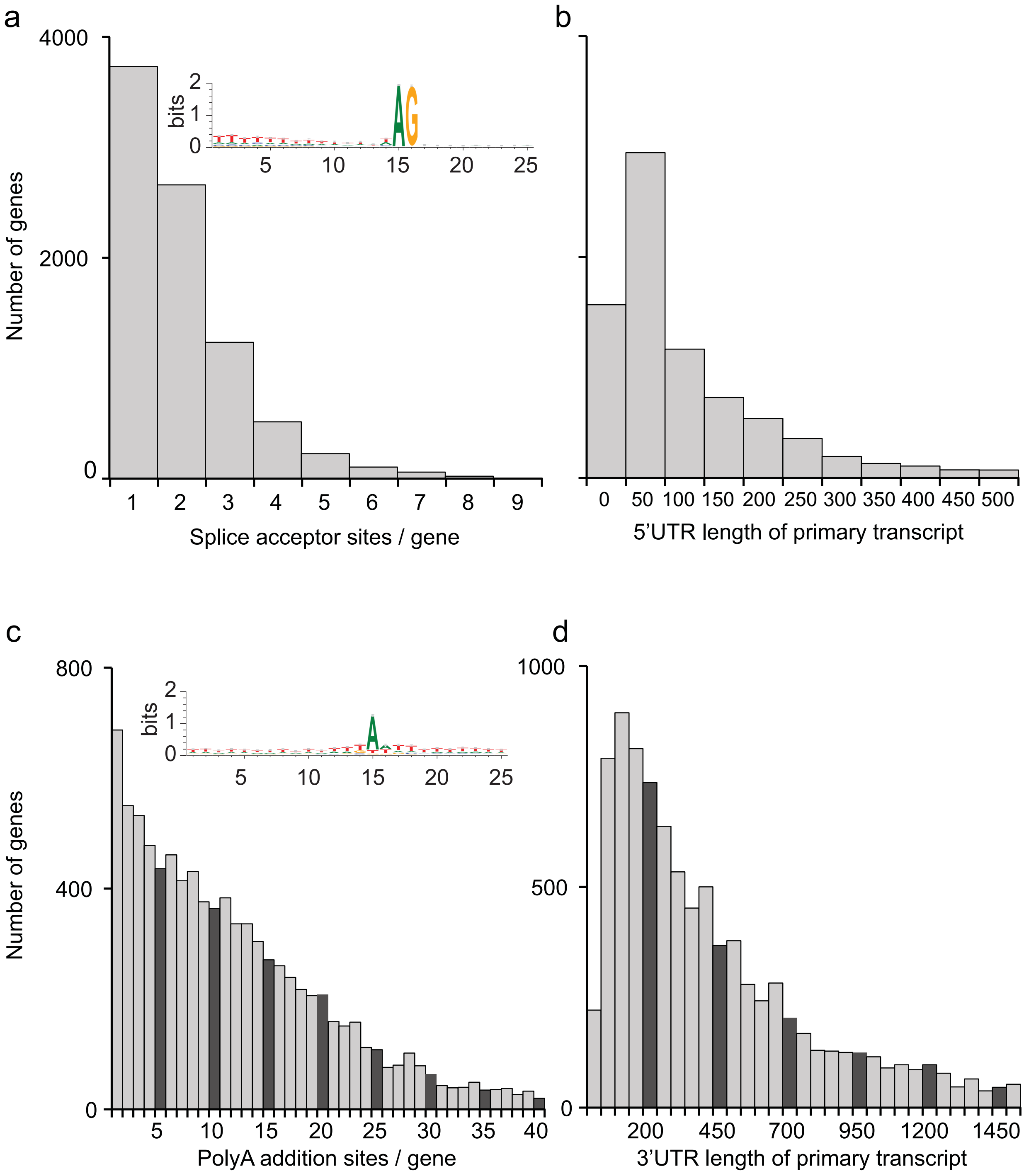

Supp Figure 2

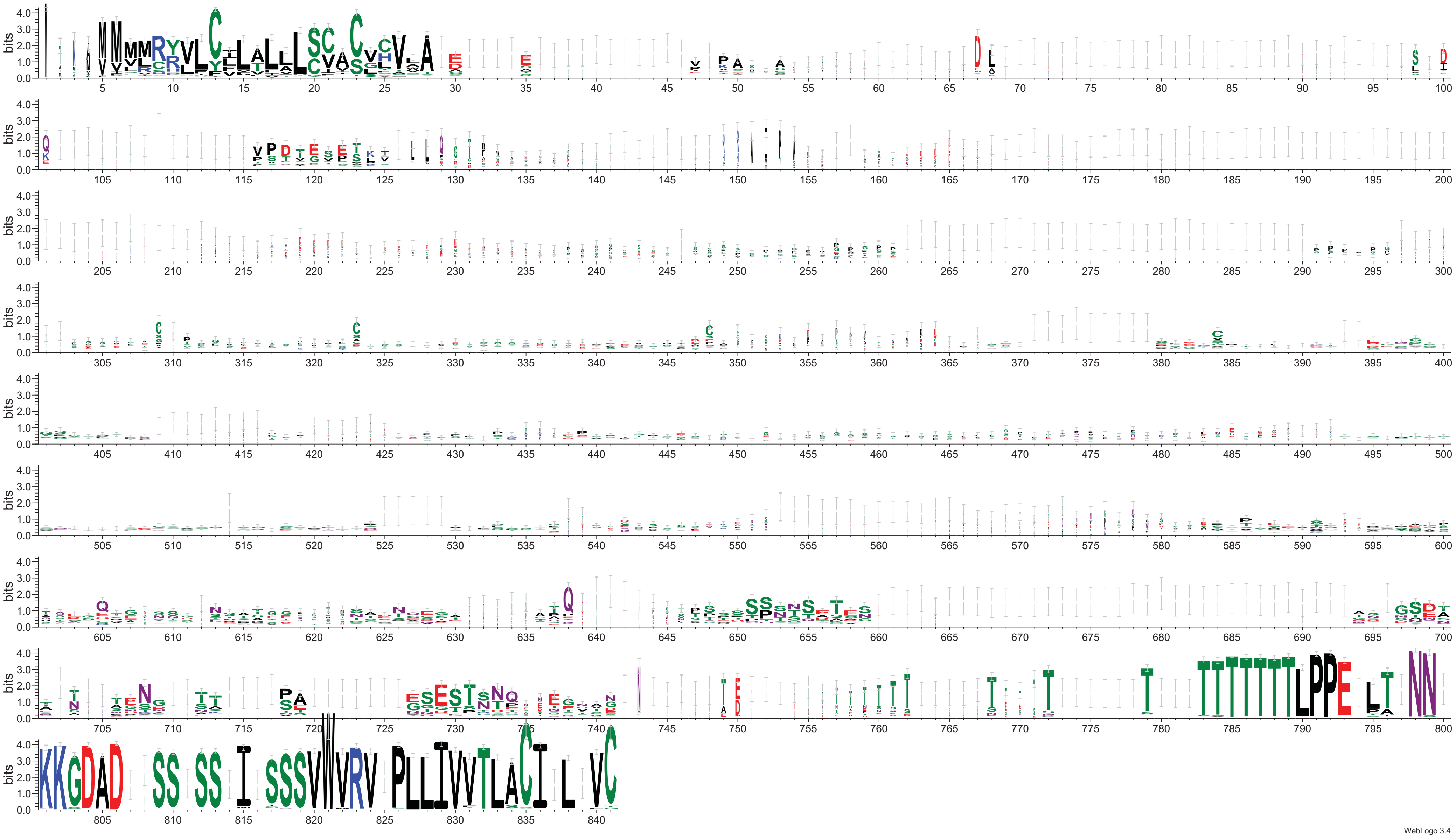

Supplementary Figure 3a. LOGO of multiple sequence alignment of full length representatives from TTPSP1 (OG0000004)

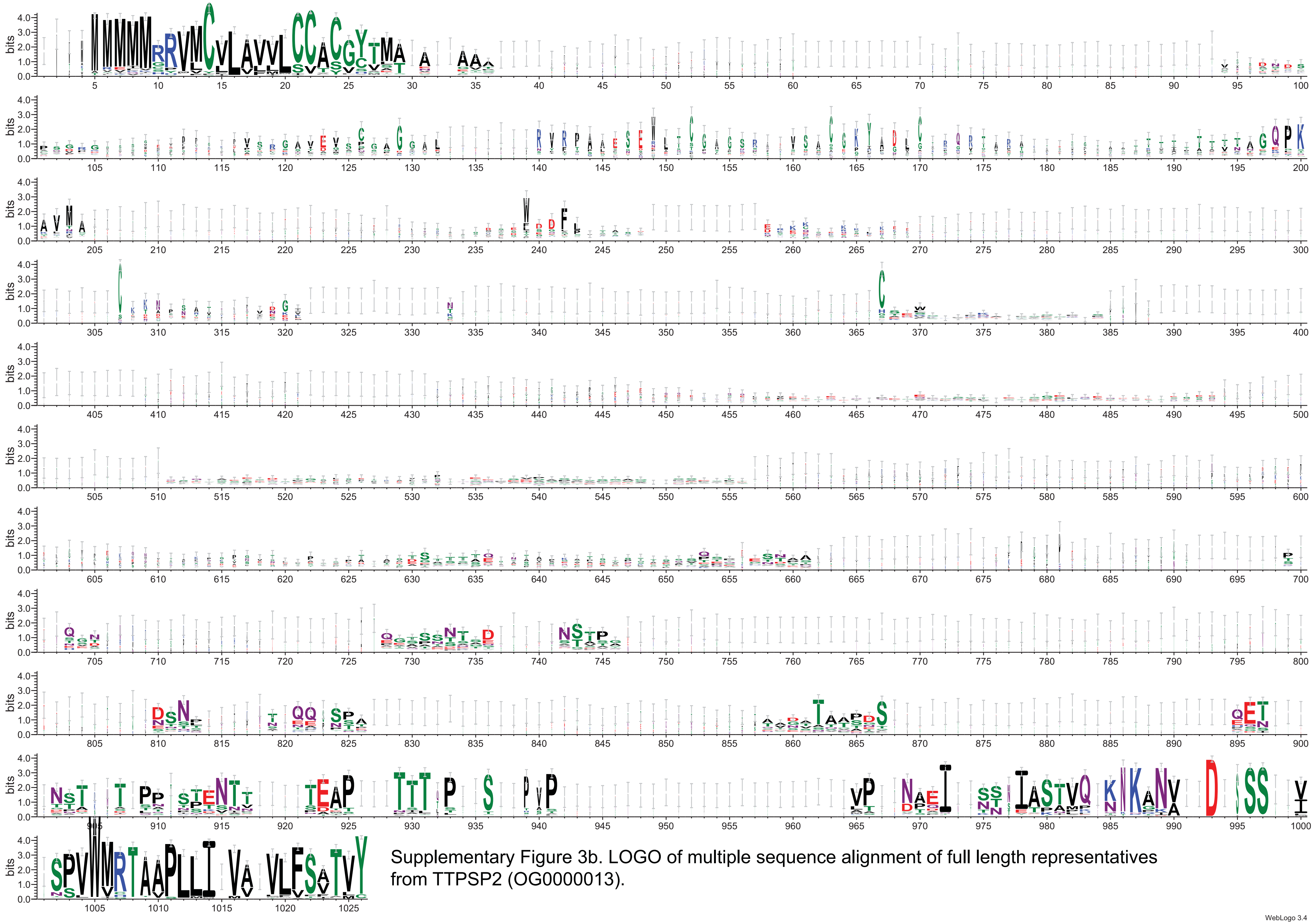

Supplementary Figure 3b. LOGO of multiple sequence alignment of full length representatives from TTPSP2 (OG0000013).

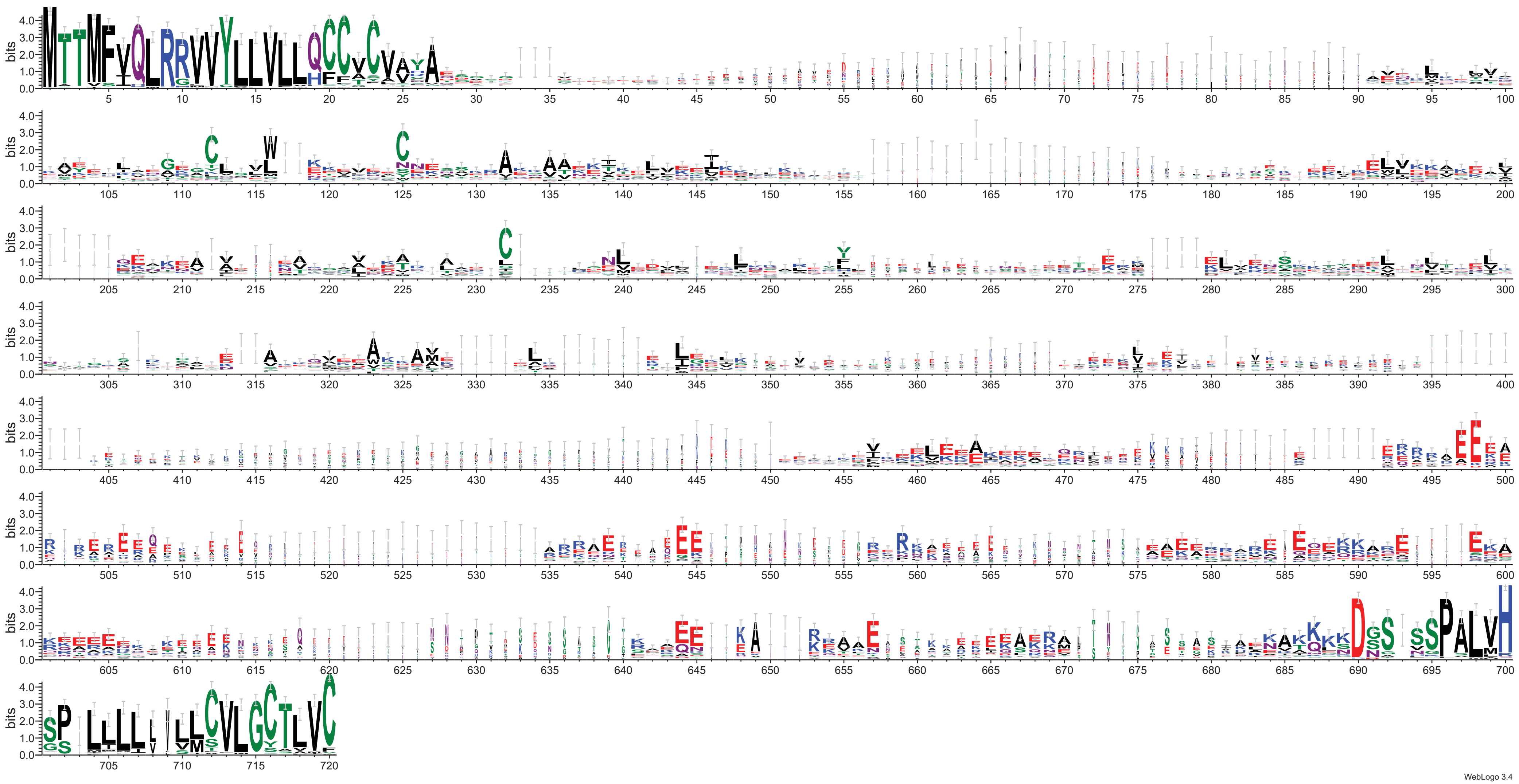

Supplementary Figure 3c. LOGO of multiple sequence alignment of full length representatives from TTPSP3 (OG0000031).

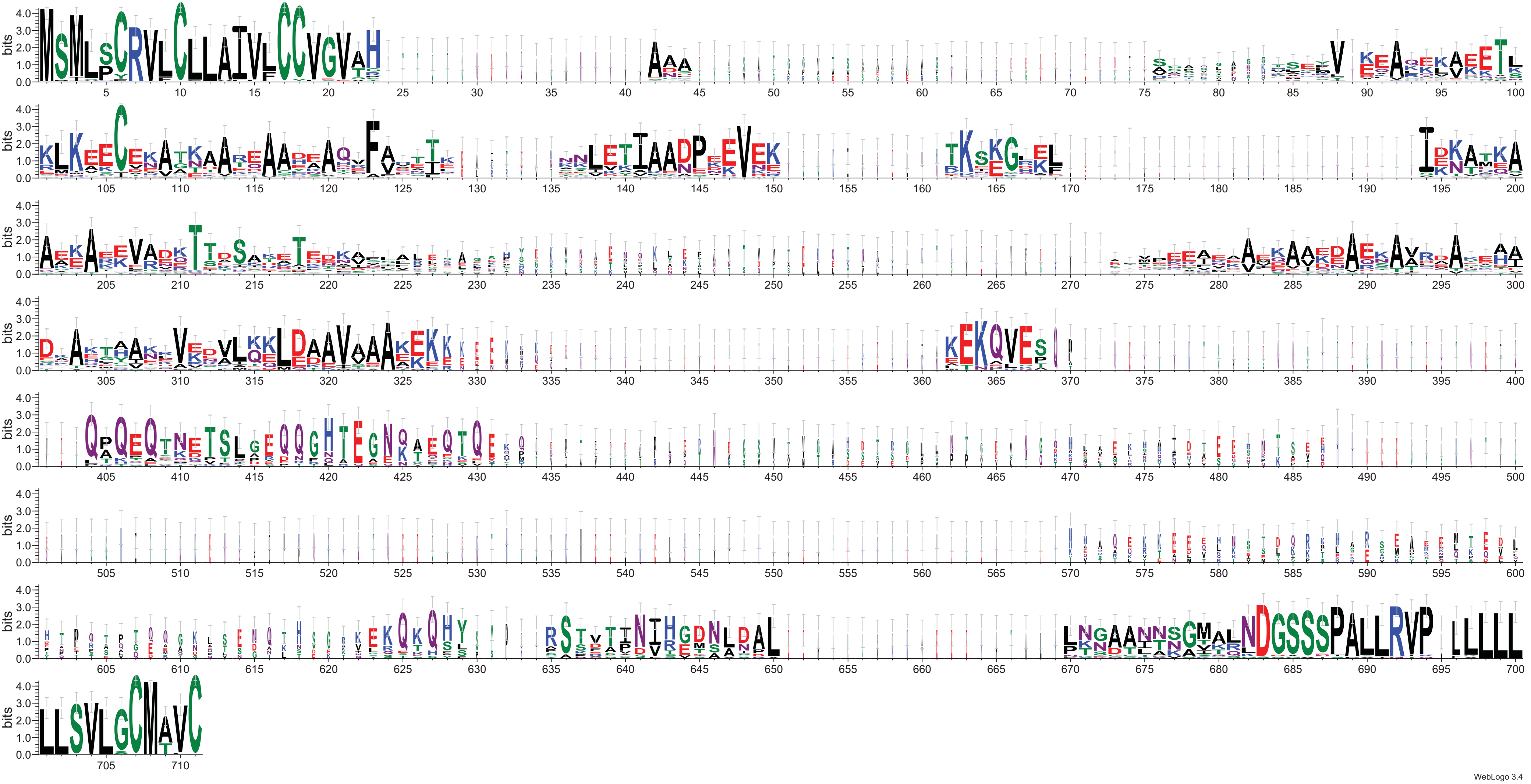

Supplementary Figure 3d. LOGO of multiple sequence alignment of full length representatives from TTPSP4 (OG000108).

a

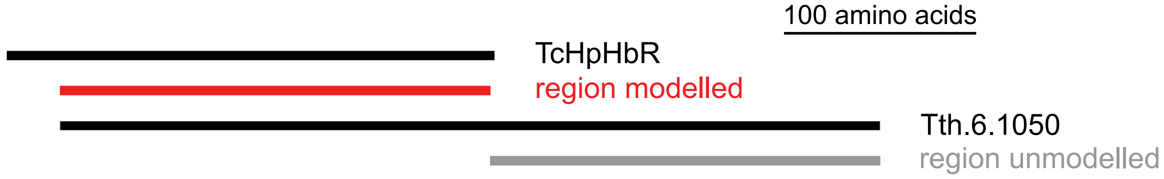

b

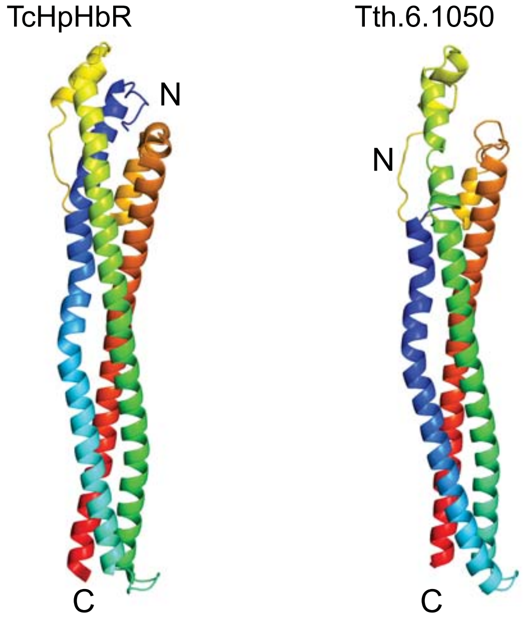

c

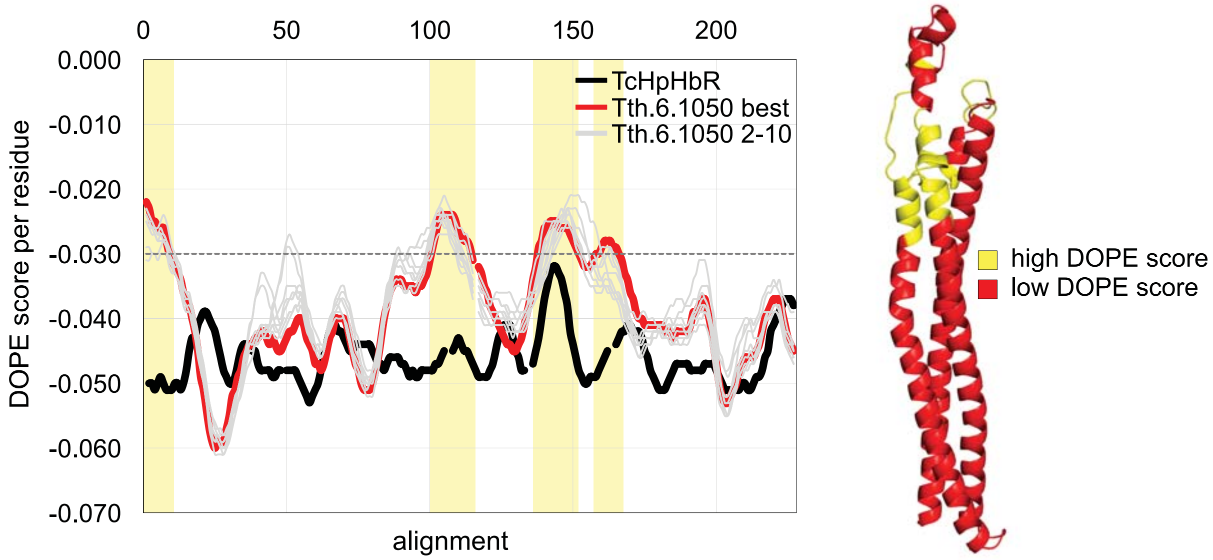

Supp Figure 4
