# Supplementary material for: An Alternative Strategy for Trypanosome Survival in the Mammalian Bloodstream Revealed through Genome and Transcriptome Analysis of the Ubiquitous Bovine Parasite Trypanosoma (Megatrypanum) theileri
Source: Genome Biol Evol. 2017 Aug 14;9(8):2093–109. doi: 10.1093/gbe/evx152 (PMC5737535; doi:10.1093/gbe/evx152)

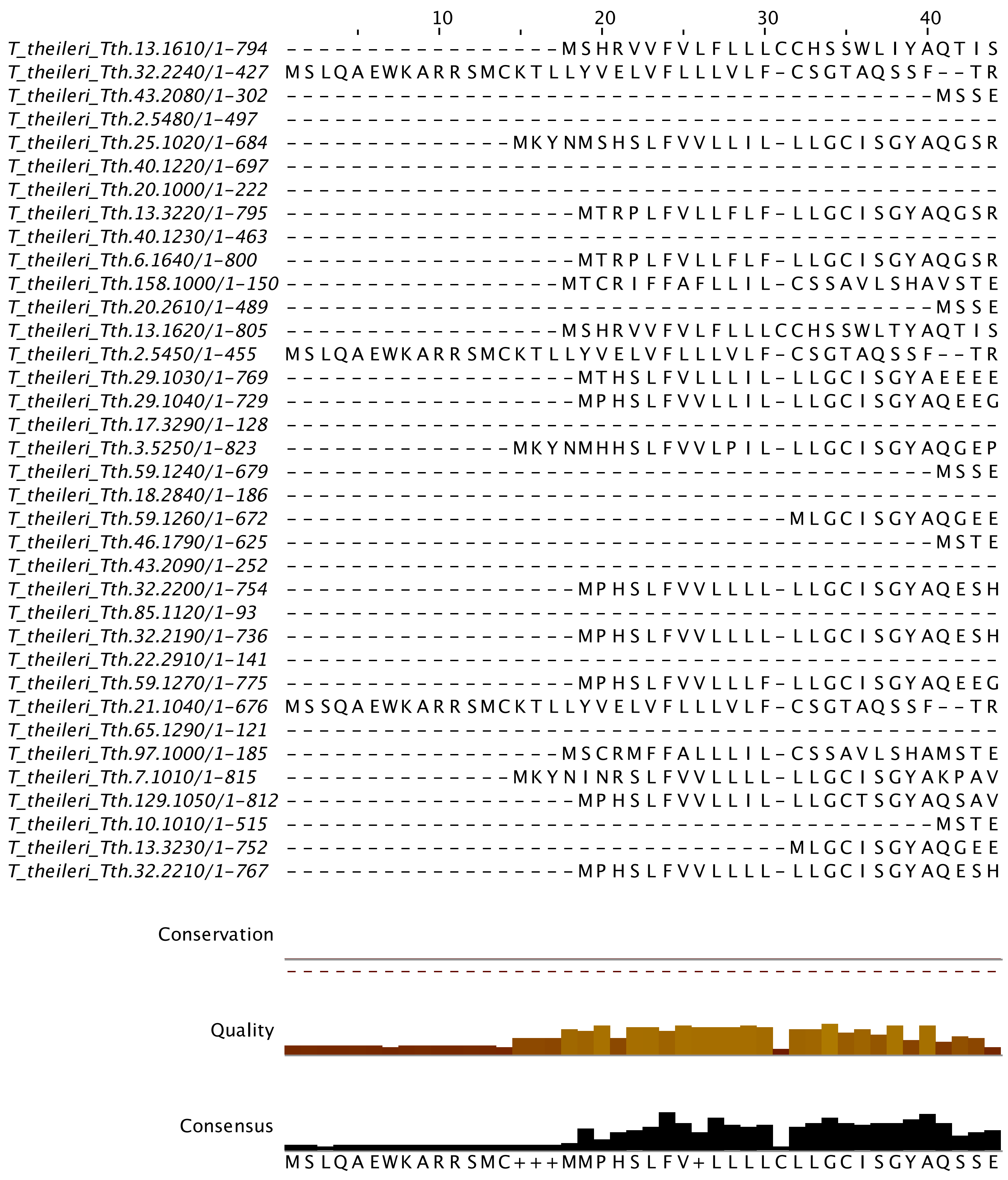
10 20 30 40
T_theileri_Tth.13.1610/1-794 -----------------MSHRVVFVLFLLLCCHSSWLIYAQTIS
T_theileri_Tth.32.2240/1-427 MSLQAEWKARRSMCKTLLYVELVFLLLVLF-CSGTAQSSF--TR
T_theileri_Tth.43.2080/1-302 --MSSE
T_theileri_Tth.2.5480/1-497 --
T_theileri_Tth.25.1020/1-684 --------------MKYNMSHSLFVVLLIL-LLGCISGYAQGSR
T_theileri_Tth.40.1220/1-697 --
T_theileri_Tth.20.1000/1-222 --
T_theileri_Tth.13.3220/1-795 ------------------MTRPLFVLLFLF-LLGCISGYAQGSR
T_theileri_Tth.40.1230/1-463 --
T_theileri_Tth.6.1640/1-800 ------------------MTRPLFVLLFLF-LLGCISGYAQGSR
T_theileri_Tth.158.1000/1-150 -----------------MTCRIFFAFLLLIL-CSSAVLSHAVSTE
T_theileri_Tth.20.2610/1-489 --MSSE
T_theileri_Tth.13.1620/1-805 -----------------MSHRVVFVLFLLLCCHSSWLTYAQTIS
T_theileri_Tth.2.5450/1-455 MSLQAEWKARRSMCKTLLYVELVFLLLVLF-CSGTAQSSF--TR
T_theileri_Tth.29.1030/1-769 ------------------MTHSLFVLLLIL-LLGCISGYAEEEE
T_theileri_Tth.29.1040/1-729 ------------------MPHSLFVVLLIL-LLGCISGYAQEEG
T_theileri_Tth.17.3290/1-128 --
T_theileri_Tth.3.5250/1-823 --------------MKYNMHHSLFVVLPIL-LLGCISGYAQGEP
T_theileri_Tth.59.1240/1-679 --MSSE
T_theileri_Tth.18.2840/1-186 --
T_theileri_Tth.59.1260/1-672 -------------------------------MLGCISGYAQGEE
T_theileri_Tth.46.1790/1-625 --MSTE
T_theileri_Tth.43.2090/1-252 --
T_theileri_Tth.32.2200/1-754 ------------------MPHSLFVVLLLL-LLGCISGYAQESH
T_theileri_Tth.85.1120/1-93 --
T_theileri_Tth.32.2190/1-736 ------------------MPHSLFVVLLLL-LLGCISGYAQESH
T_theileri_Tth.22.2910/1-141 --
T_theileri_Tth.59.1270/1-775 ------------------MPHSLFVVLLLF-LLGCISGYAQEEG
T_theileri_Tth.21.1040/1-676 MSSQAEWKARRSMCKTLLYVELVFLLLVLF-CSGTAQSSF--TR
T_theileri_Tth.65.1290/1-121 --
T_theileri_Tth.97.1000/1-185 -----------------MSCRMFFALLLLIL-CSSAVLSHAMSTE
T_theileri_Tth.7.1010/1-815 --------------MKYNINRSLFVVLLLL-LLGCISGYAKPAV
T_theileri_Tth.129.1050/1-812 ------------------MPHSLFVVLLIL-LLGCTSGYAQSAV
T_theileri_Tth.10.1010/1-515 --MSTE
T_theileri_Tth.13.3230/1-752 -------------------------------MLGCISGYAQGEE
T_theileri_Tth.32.2210/1-767 ------------------MPHSLFVVLLLL-LLGCISGYAQESH
Conservation

Quality
Consensus
MSLQAEWKARRSMC+++MMPHSLFV+LLLLCLLGCISGYAQSSE

```
                               50        60        70        80
T_theileri_Tth.13.1610/1-794   GREESR---------------------TMLFKQGESKVPF----
T_theileri_Tth.32.2240/1-427   RGRPKR---------------------TFLFKQGVTSVPF----
T_theileri_Tth.43.2080/1-302   ERQRSR---------------------VILFERGVTTVPF----
T_theileri_Tth.2.5480/1-497    --------------------------------------------
T_theileri_Tth.25.1020/1-684   SEESII---------------------RTIGGDSKEVYQTKEST
T_theileri_Tth.40.1220/1-697   ------------------------------FERMKSTVPF----
T_theileri_Tth.20.1000/1-222   --------------------------------------------
T_theileri_Tth.13.3220/1-795   FKERTT---------------------RTIGND-KELFQVKNSA
T_theileri_Tth.40.1230/1-463   --------------------------------------------
T_theileri_Tth.6.1640/1-800    FKESTT---------------------RTIGND-KELFQAKNSA
T_theileri_Tth.158.1000/1-150  ERERSR---------------------VILFERGVTTVPF----
T_theileri_Tth.20.2610/1-489   ERERSR---------------------VTLFKEGVTAVPF----
T_theileri_Tth.13.1620/1-805   GREKSR---------------------ITLFERNVSTVPF----
T_theileri_Tth.2.5450/1-455    RGGPKR---------------------KFLFRQGKTSVPF----
T_theileri_Tth.29.1030/1-769   PIR-----------------------------------------
T_theileri_Tth.29.1040/1-729   PQEETV---------------------PIVDSIPSELFKSINMS
T_theileri_Tth.17.3290/1-128   --------------------------------------------
T_theileri_Tth.3.5250/1-823    EQLRAT---------------------ILFAEKQVTSAPSTRNR
T_theileri_Tth.59.1240/1-679   ERERSR---------------------VILFKEGVTTVPF----
T_theileri_Tth.18.2840/1-186   --------------------------------------------
T_theileri_Tth.59.1260/1-672   PQEKTT---------------------LL---------------
T_theileri_Tth.46.1790/1-625   ERERSR---------------------VILFERGVTTVPF----
T_theileri_Tth.43.2090/1-252   --------------------------------------------
T_theileri_Tth.32.2200/1-754   SQGSPV-DDGVPNDEPTEKMMPGHEELQQLHERLKELR------
T_theileri_Tth.85.1120/1-93    --------------------------------------------
T_theileri_Tth.32.2190/1-736   SQGSPVDDDGVHQRELKEKMMPGHEELQTLQKKVK---------
T_theileri_Tth.22.2910/1-141   --------------------------------------------
T_theileri_Tth.59.1270/1-775   PQEETV---------------------AIVDSTRSELCKPMT--
T_theileri_Tth.21.1040/1-676   RGRPKR---------------------TFLFKQGVTSVPF----
T_theileri_Tth.65.1290/1-121   --------------------------------------------
T_theileri_Tth.97.1000/1-185   ERERSR---------------------VILFERGVTAVPF----
T_theileri_Tth.7.1010/1-815    PQTKSA---------------------SVFTITQKTTLPTARYQ
T_theileri_Tth.129.1050/1-812  QQEEIT---------------------LL---------------
T_theileri_Tth.10.1010/1-515   ERERSR---------------------VILFERGVTTVPF----
T_theileri_Tth.13.3230/1-752   PQEKTT---------------------LL---------------
T_theileri_Tth.32.2210/1-767   SQRSAD-DDGVPSDEPTEKMMPGHDELQQLHERLKELRKKEEEE
```

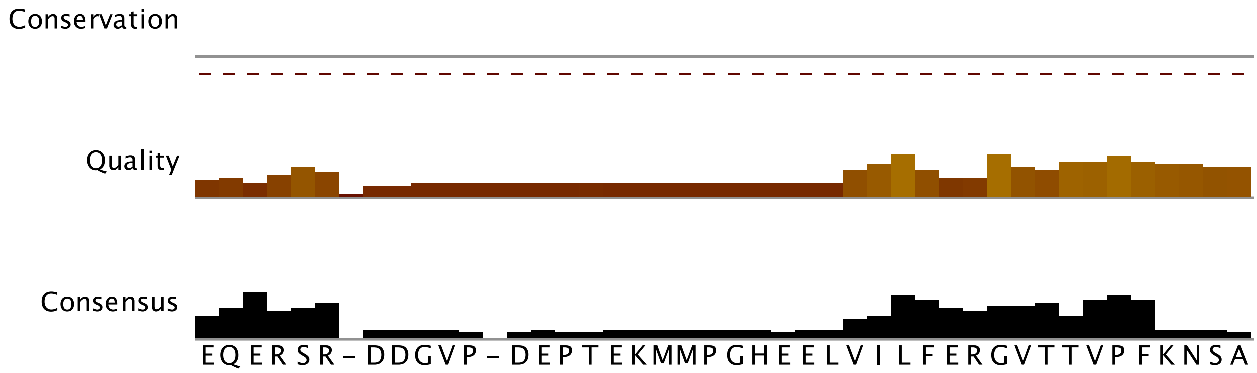

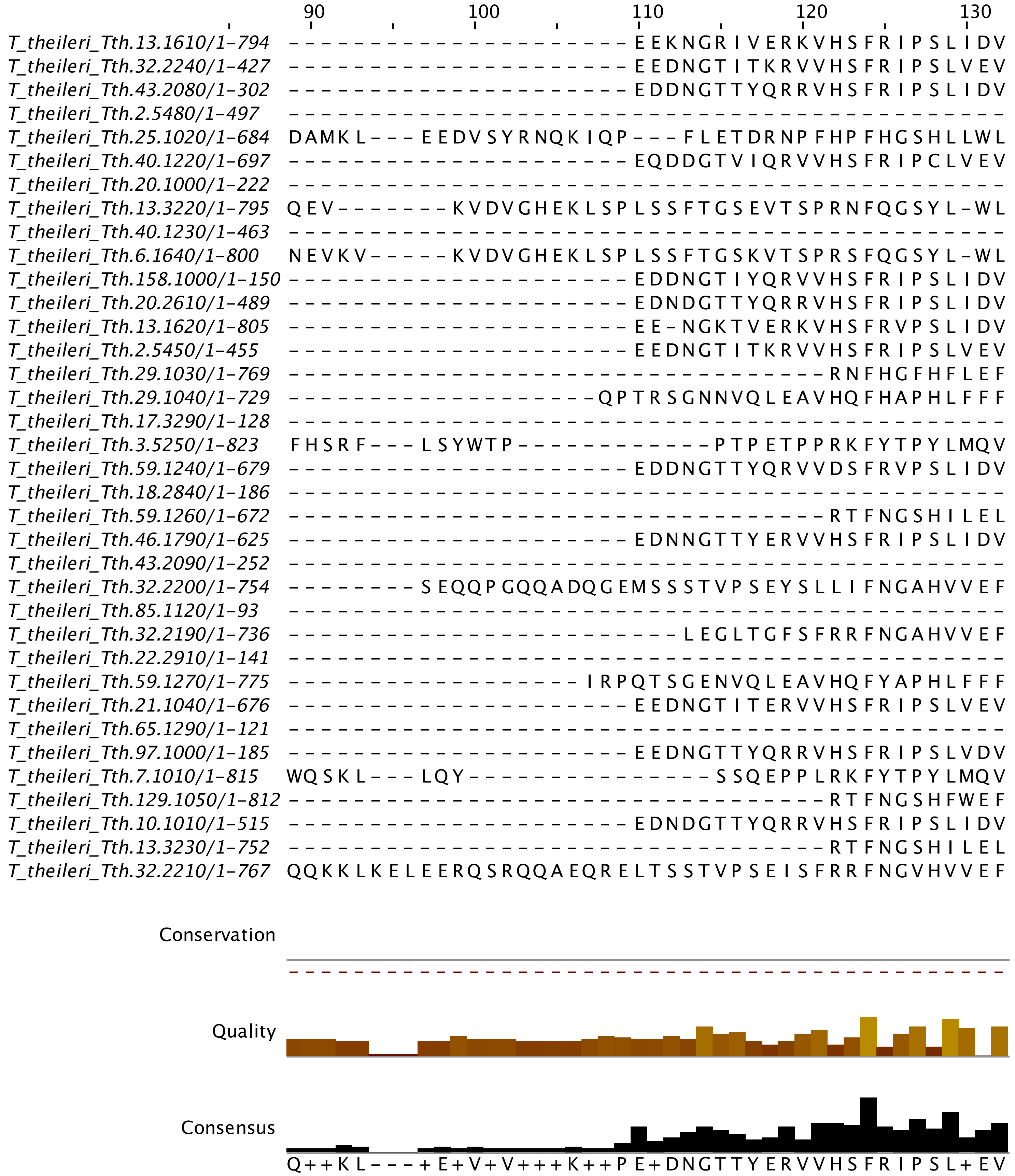
90 100 110 120 130
T_theileri_Tth.13.1610/1-794 ---------------------EEKNGRIVERKVHSFRIPSLIDV
T_theileri_Tth.32.2240/1-427 ---------------------EEDNGTITKRVVHSFRIPSLVEV
T_theileri_Tth.43.2080/1-302 ---------------------EDDNGTTYQRRVHSFRIPSLIDV
T_theileri_Tth.2.5480/1-497 --
T_theileri_Tth.25.1020/1-684 DAMKL---EEDVSYRNQKIQP---FLETDRNPFHPFHGSHLLWL
T_theileri_Tth.40.1220/1-697 ---------------------EQDDGTVIQRVVHSFRIPCLVEV
T_theileri_Tth.20.1000/1-222 --
T_theileri_Tth.13.3220/1-795 QEV-------KVDVGHEKLSPLSSFTGSEVTSPRNFQGSYL-WL
T_theileri_Tth.40.1230/1-463 --
T_theileri_Tth.6.1640/1-800 NEVKV-----KVDVGHEKLSPLSSFTGSKVTSPRSFQGSYL-WL
T_theileri_Tth.158.1000/1-150 ---------------------EDDNGTIYQRVVHSFRIPSLIDV
T_theileri_Tth.20.2610/1-489 ---------------------EDNDGTTYQRRVHSFRIPSLIDV
T_theileri_Tth.13.1620/1-805 ---------------------EE-NGKTVERKVHSFRVPSLIDV
T_theileri_Tth.2.5450/1-455 ---------------------EEDNGTITKRVVHSFRIPSLVEV
T_theileri_Tth.29.1030/1-769 ---------------------------------RNFHGFHFLEF
T_theileri_Tth.29.1040/1-729 -------------------QPTRSGNNVQLEAVHQFHAPHLFFF
T_theileri_Tth.17.3290/1-128 --
T_theileri_Tth.3.5250/1-823 FHSRF---LSYWTP------------PTPETPPRKFYTPYLMQV
T_theileri_Tth.59.1240/1-679 ---------------------EDDNGTTYQRVVDSFRVPSLIDV
T_theileri_Tth.18.2840/1-186 --
T_theileri_Tth.59.1260/1-672 ---------------------------------RTFNGSHILEL
T_theileri_Tth.46.1790/1-625 ---------------------EDNNGTTYERVVHSFRIPSLIDV
T_theileri_Tth.43.2090/1-252 --
T_theileri_Tth.32.2200/1-754 --------SEQQPGQQADQGEMSSSTVPSEYSLLIFNGAHVVEF
T_theileri_Tth.85.1120/1-93 --
T_theileri_Tth.32.2190/1-736 ------------------------LEGLTGFSFRRFNGAHVVEF
T_theileri_Tth.22.2910/1-141 --
T_theileri_Tth.59.1270/1-775 ------------------IRPQTSGENVQLEAVHQFYAPHLFFF
T_theileri_Tth.21.1040/1-676 ---------------------EEDNGTITERVVHSFRIPSLVEV
T_theileri_Tth.65.1290/1-121 --
T_theileri_Tth.97.1000/1-185 ---------------------EEDNGTTYQRRVHSFRIPSLVDV
T_theileri_Tth.7.1010/1-815 WQSKL---LQY---------------SSQEPPLRKFYTPYLMQV
T_theileri_Tth.129.1050/1-812 ---------------------------------RTFNGSHFWEF
T_theileri_Tth.10.1010/1-515 ---------------------EDNDGTTYQRRVHSFRIPSLIDV
T_theileri_Tth.13.3230/1-752 ---------------------------------RTFNGSHILEL
T_theileri_Tth.32.2210/1-767 QQKKLKELEERQSRQQAEQRELTSSTVPSEISFRRFNGVHVVEF
Conservation

Quality
Consensus
Q++KL---+E+V+V+++K++P E+DNGTTYERVVHSFRIPSL+EV

```
                                140       150       160       170
T_theileri_Tth.13.1610/1-794    -DGVMVAIADA--RY---DDSNDNSFIETLSAHS---VDDGKTW
T_theileri_Tth.32.2240/1-427    -NGVLVAIADA--RY---ISGDDQSFIETVVKYS---VDGGKTW
T_theileri_Tth.43.2080/1-302    -DGVMVAIGDA--RY---NTSNDNSFIETVVKFS---ADNGKTW
T_theileri_Tth.2.5480/1-497     --------------------------------------------
T_theileri_Tth.25.1020/1-684    GNKKLVTVATS--RLLQ-LFADKTEFIDFFVGES---SNNGEKW
T_theileri_Tth.40.1220/1-697    -EGVMVAIADA--RY---TTSNDNSFIETVAKFS---VDNGETW
T_theileri_Tth.20.1000/1-222    --------------------------------------------
T_theileri_Tth.13.3220/1-795    GNGKLVAIDTAATAMYQ-KSTNKSEFIDFLTQES---SDNGKNW
T_theileri_Tth.40.1230/1-463    --------------------------------------------
T_theileri_Tth.6.1640/1-800     GNGKLVAIDTAATAMYQ-KSANKSEFIDFLTQES---SDNGKKW
T_theileri_Tth.158.1000/1-150   -DGVMVAIGDA--RY---NTSSDNSFIETVVKFS---VDNGKTW
T_theileri_Tth.20.2610/1-489    -DGVMIAIGDA--RY---NTSNDNSFIETVVKFS---VDNGKTW
T_theileri_Tth.13.1620/1-805    -DGVMVVIADA--RY---VDSNDNSFIETLSAHS---VDDLKTW
T_theileri_Tth.2.5450/1-455     -NGVLVAIADA--RY---ISGDDQSFIETVVKYS---VDGGSTW
T_theileri_Tth.29.1030/1-769    GNGDLVAIDGA--NLYR-WNKHKVEYMELLTPAS---GEGQGAW
T_theileri_Tth.29.1040/1-729    -NGYLVSITAA--AVYQDRDTKQNEFIKLLKKNKTVESETVLKW
T_theileri_Tth.17.3290/1-128    --------------------------------------------
T_theileri_Tth.3.5250/1-823     -NGELVAMASA--TVQPGLSNNQYEIVDFQRRKS---SDNGTKW
T_theileri_Tth.59.1240/1-679    -DGVMVAIADA--RY---NSDNDNSFIETVVKYS---VDDGKTW
T_theileri_Tth.18.2840/1-186    --------------------------------------------
T_theileri_Tth.59.1260/1-672    ENGQLVAINGA--NLYR-WNGSQREYMELLTPAS---SEGENAL
T_theileri_Tth.46.1790/1-625    -DGVMVAIGDA--RY---NTSSDNSFIETVVKFS---VDDGKTW
T_theileri_Tth.43.2090/1-252    --------------------------------------------
T_theileri_Tth.32.2200/1-754    VSGELVAIDAA--LVEQ-SLKDKNELVTFVSQDM---NNNG-EW
T_theileri_Tth.85.1120/1-93     --------------------------------------------
T_theileri_Tth.32.2190/1-736    VNGELVAIDAA--AVEQ-SRKDKNELVKFLSQDM---NNNG-EW
T_theileri_Tth.22.2910/1-141    --------------------------------------------
T_theileri_Tth.59.1270/1-775    -NGSLVSITAA--AVCQEQGSNQNEFIKILKNKN---EEGKKQW
T_theileri_Tth.21.1040/1-676    -NGVLVALADA--RY---ISGDDQSFIETVVKYS---VDGGKTW
T_theileri_Tth.65.1290/1-121    --------------------------------------------
T_theileri_Tth.97.1000/1-185    -DGVMVAIGDA--RY---NTSNDNSFIETVVKYS---VDDGKTW
T_theileri_Tth.7.1010/1-815     -NGELVVMASA--TIHP-ASNNQYETINFQRRKS---SDNGTKW
T_theileri_Tth.129.1050/1-812   GNGELVAIDAA--VWFP-PNATQHEYIELLTPAS---SEGENAW
T_theileri_Tth.10.1010/1-515    -DGVMVAIGDA--RY---NASNDNSFIETVVKYS---VDDGKTW
T_theileri_Tth.13.3230/1-752    ENGQLVAINGA--NLYP-RNGSQPDSIELLTPAN---GVGENAW
T_theileri_Tth.32.2210/1-767    VNGELVAIDAA--GVEQ-SLKDNSEFVEFLSQDM---NNNG-EW
```

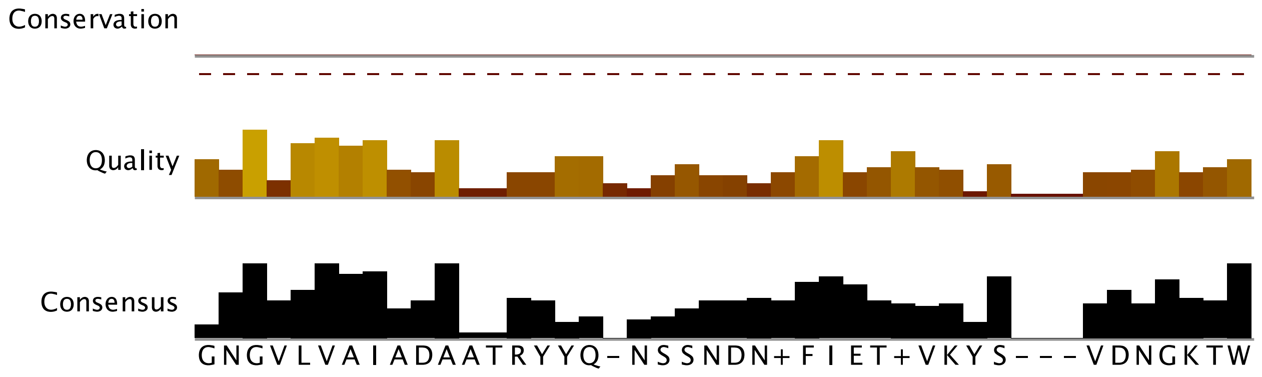

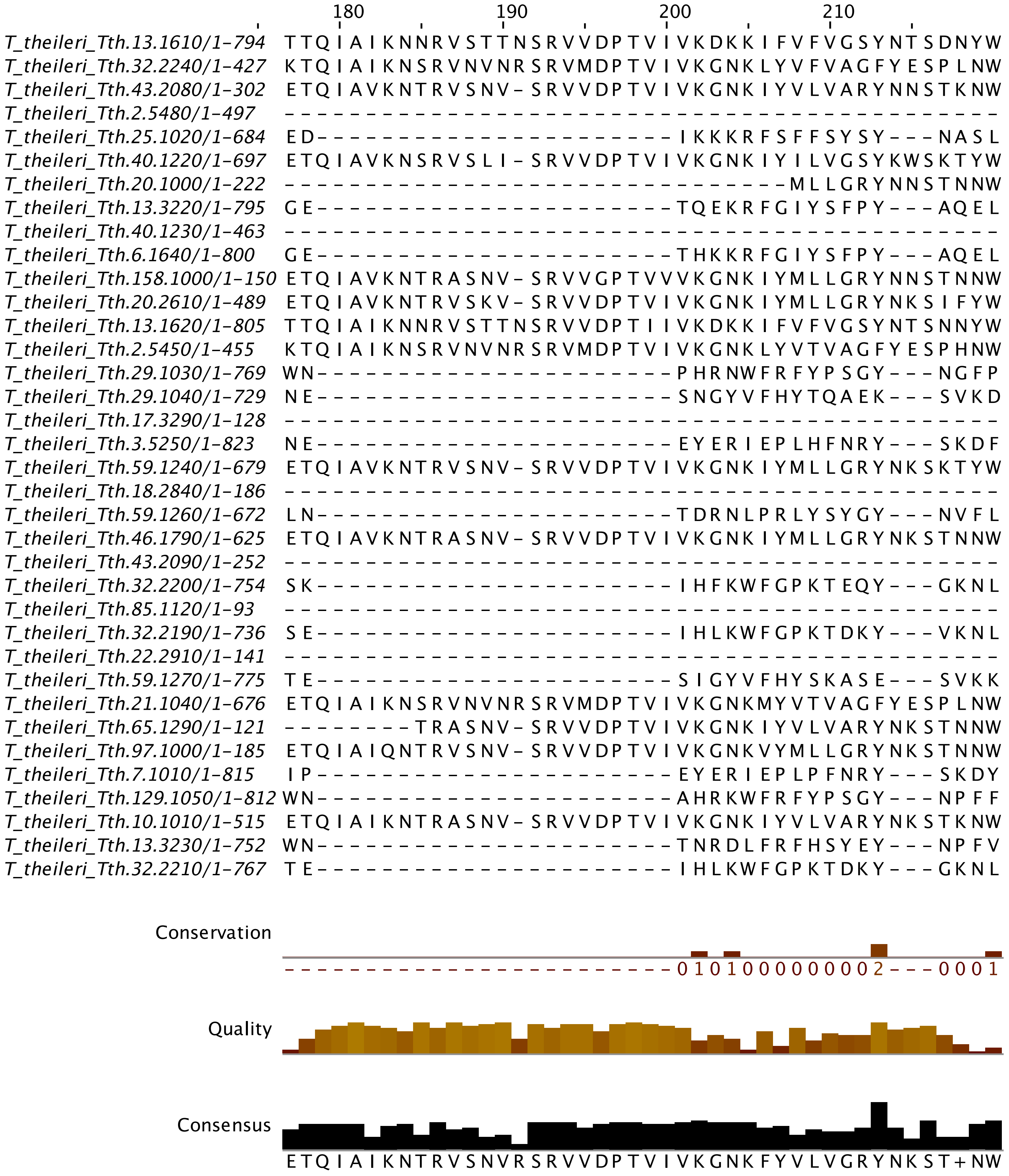
180 190 200 210
T_theileri_Tth.13.1610/1-794 TTQIAIKNNRVSTTNSRVVDPTVIVKDKKIFVFVGSYNTSDNYW
T_theileri_Tth.32.2240/1-427 KTQIAIKNSRVNVNRSRVMDPTVIVKGNKLYVFVAGFYESPLNW
T_theileri_Tth.43.2080/1-302 ETQIAVKNTRVSNV-SRVVDPTVIVKGNKIYVLVARYNNSTKNW
T_theileri_Tth.2.5480/1-497 --
T_theileri_Tth.25.1020/1-684 ED----------------------IKKKRFSFFSYSY---NASL
T_theileri_Tth.40.1220/1-697 ETQIAVKNSRVSLI-SRVVDPTVIVKGNKIYILVGSYKWSKTYW
T_theileri_Tth.20.1000/1-222 -------------------------------MLLGRYNNSTNNW
T_theileri_Tth.13.3220/1-795 GE----------------------TQEKRFGIYSFPY---AQEL
T_theileri_Tth.40.1230/1-463 --
T_theileri_Tth.6.1640/1-800 GE----------------------THKKRFGIYSFPY---AQEL
T_theileri_Tth.158.1000/1-150 ETQIAVKNTRASNV-SRVVGPTVVVKGNKIYMLLGRYNNSTNNW
T_theileri_Tth.20.2610/1-489 ETQIAVKNTRVSKV-SRVVDPTVIVKGNKIYMLLGRYNKSIFYW
T_theileri_Tth.13.1620/1-805 TTQIAIKNNRVSTTNSRVVDPTIIVKDKKIFVFVGSYNTSNNYW
T_theileri_Tth.2.5450/1-455 KTQIAIKNSRVNVNRSRVMDPTVIVKGNKLYVTVAGFYESPHNW
T_theileri_Tth.29.1030/1-769 WN----------------------PHRNWFRFYPSGY---NGFP
T_theileri_Tth.29.1040/1-729 NE----------------------SNGYVFHYTQAEK---SVKD
T_theileri_Tth.17.3290/1-128 --
T_theileri_Tth.3.5250/1-823 NE----------------------EYERIEPLHFNRY---SKDF
T_theileri_Tth.59.1240/1-679 ETQIAVKNTRVSNV-SRVVDPTVIVKGNKIYMLLGRYNKSKTYW
T_theileri_Tth.18.2840/1-186 --
T_theileri_Tth.59.1260/1-672 LN----------------------TDRNLPRLYSYGY---NVFL
T_theileri_Tth.46.1790/1-625 ETQIAVKNTRASNV-SRVVDPTVIVKGNKIYMLLGRYNKSTNNW
T_theileri_Tth.43.2090/1-252 --
T_theileri_Tth.32.2200/1-754 SK----------------------IHFKWFGPKTEQY---GKNL
T_theileri_Tth.85.1120/1-93 --
T_theileri_Tth.32.2190/1-736 SE----------------------IHLKWFGPKTDKY---VKNL
T_theileri_Tth.22.2910/1-141 --
T_theileri_Tth.59.1270/1-775 TE----------------------SIGYVFHYSKASE---SVKK
T_theileri_Tth.21.1040/1-676 ETQIAIKNSRVNVNRSRVMDPTVIVKGNKMYVTVAGFYESPLNW
T_theileri_Tth.65.1290/1-121 --------TRASNV-SRVVDPTVIVKGNKIYVLVARYNKSTNNW
T_theileri_Tth.97.1000/1-185 ETQIAIQNTRVSNV-SRVVDPTVIVKGNKVYMLLGRYNKSTNNW
T_theileri_Tth.7.1010/1-815 IP----------------------EYERIEPLPFNRY---SKDY
T_theileri_Tth.129.1050/1-812 WN----------------------AHRKWFRFYPSGY---NPFF
T_theileri_Tth.10.1010/1-515 ETQIAIKNTRASNV-SRVVDPTVIVKGNKIYVLVARYNKSTKNW
T_theileri_Tth.13.3230/1-752 WN----------------------TNRDLFRFHSYEY---NPFV
T_theileri_Tth.32.2210/1-767 TE----------------------IHLKWFGPKTDKY---GKNL
Conservation
------------------------0101000000002---0001
Quality
Consensus
ETQIAIKNTRVSNVRSRVVDPTVIVKGNKFYVLVGRYNKST+NW

```
                                                230       240       250       260
T_theileri_Tth.13.1610/1-794    QWH-AGKDWDPLLVVGEVSKTNENGK--------------VKAT
T_theileri_Tth.32.2240/1-427    VQQPDGSDWYTPLAIGEVNKWTVNGK--------------PIAS
T_theileri_Tth.43.2080/1-302    GQHYDGKDWEPVFSVGEVKKTAINGK--------------VNAT
T_theileri_Tth.2.5480/1-497     --------------------------------------------
T_theileri_Tth.25.1020/1-684    WNPKDYGTSHDTFVLVESKALPSKYDSKSTQTK----IQNSTLR
T_theileri_Tth.40.1220/1-697    TQH-KGKDWEPLLAIGEVKKTNISGN--------------VTAT
T_theileri_Tth.20.1000/1-222    NQHTTGNDWEPVLSVGEVKKKTINGK--------------VNAT
T_theileri_Tth.13.3220/1-795    EKHKMYDNHRSVFVLVESNNLQSQYRSQSQGTE--INTKNSTLR
T_theileri_Tth.40.1230/1-463    --------------------------------------------
T_theileri_Tth.6.1640/1-800     EKHKMYDNHRSVFVLVESNNLQSQYRSQSQGTE--INTKNSTLR
T_theileri_Tth.158.1000/1-150   NQHT----------------------------------------
T_theileri_Tth.20.2610/1-489    TQHTTGNDWEPVLSVGEVKKTTIDGK--------------VNAT
T_theileri_Tth.13.1620/1-805    QWH-PGTDWDPLLVVGEVSKTNENGE--------------VKAT
T_theileri_Tth.2.5450/1-455     VQQPDGSDWYTLLAIGEVNKWTVNGK--------------PIAS
T_theileri_Tth.29.1030/1-769    INYGSGTPFRAVFALVENNNPPSKEGAHPVRSKNPPTPEYSFIH
T_theileri_Tth.29.1040/1-729    VFDNDKKDYQAVLALVESSNPPSQEG--------------SVLR
T_theileri_Tth.17.3290/1-128    --------------------------------------------
T_theileri_Tth.3.5250/1-823     VSSPDMKDYYPAFVLVESNNTPTVKDSPYILSTTSEKKD-SILR
T_theileri_Tth.59.1240/1-679    NQQYDGKDWEPVFSVGEVKKTTINGK--------------VNAT
T_theileri_Tth.18.2840/1-186    --------------------------------------------
T_theileri_Tth.59.1260/1-672    MRYGSGTSFRAVFALVESNDPPSEKY--------------SVLR
T_theileri_Tth.46.1790/1-625    NQHTTGNDWEPVLSVGEVKKTTIDGK--------------VNAT
T_theileri_Tth.43.2090/1-252    --------------------------------------------
T_theileri_Tth.32.2200/1-754    EKYKPHDVPRAVVALVESDNPPIEAG--------------SVLR
T_theileri_Tth.85.1120/1-93     --------------------------------------------
T_theileri_Tth.32.2190/1-736    EKYKPHGDPRAVVALVESDNPQSQAD--------------SVVR
T_theileri_Tth.22.2910/1-141    --------------------------------------------
T_theileri_Tth.59.1270/1-775    VFDNDKNDYQAALALVESKEDSSGTA---------DGPQNFTIR
T_theileri_Tth.21.1040/1-676    VQQPDGSDWYTLLAIGEVKKRTVNGK--------------LSAS
T_theileri_Tth.65.1290/1-121    NQHPDGKDWEPVLSVGEVKKTNINGK--------------VNAT
T_theileri_Tth.97.1000/1-185    NQHPDGKDWEPVLSVGEVKKTTINGK--------------VNAT
T_theileri_Tth.7.1010/1-815     IPSPNEKDYYPAFVLVESNNTPTVDGTPYYFSQNAEKKY-STLR
T_theileri_Tth.129.1050/1-812   MGYGSGKPFHAVFALVESNNPPRRED--------------STLR
T_theileri_Tth.10.1010/1-515    GQHPDGKDWEPVFSVGEVKKTAINGK--------------VNAT
T_theileri_Tth.13.3230/1-752    RHYGFGRWFRAMYALVESNNPPSQEN--------------SVLH
T_theileri_Tth.32.2210/1-767    EKYKPHGDPRAVVALVERNIPPIEAG--------------SVLR
```

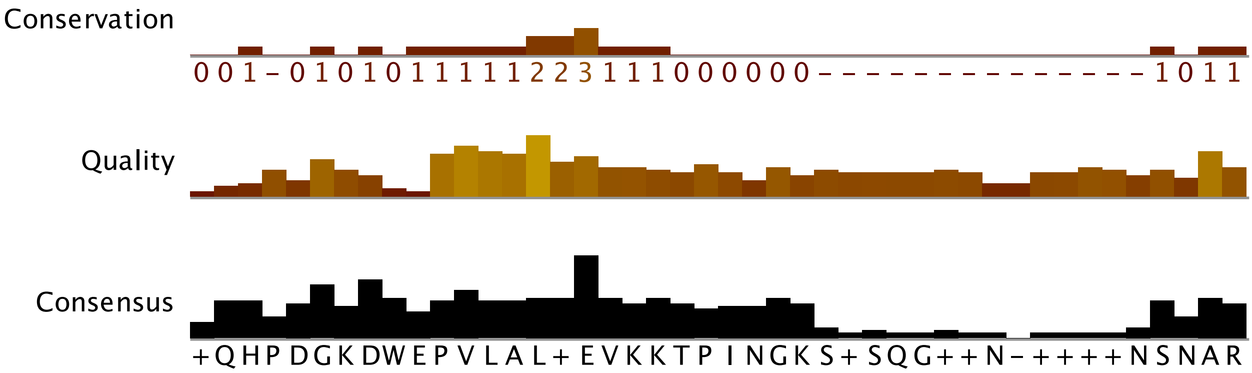

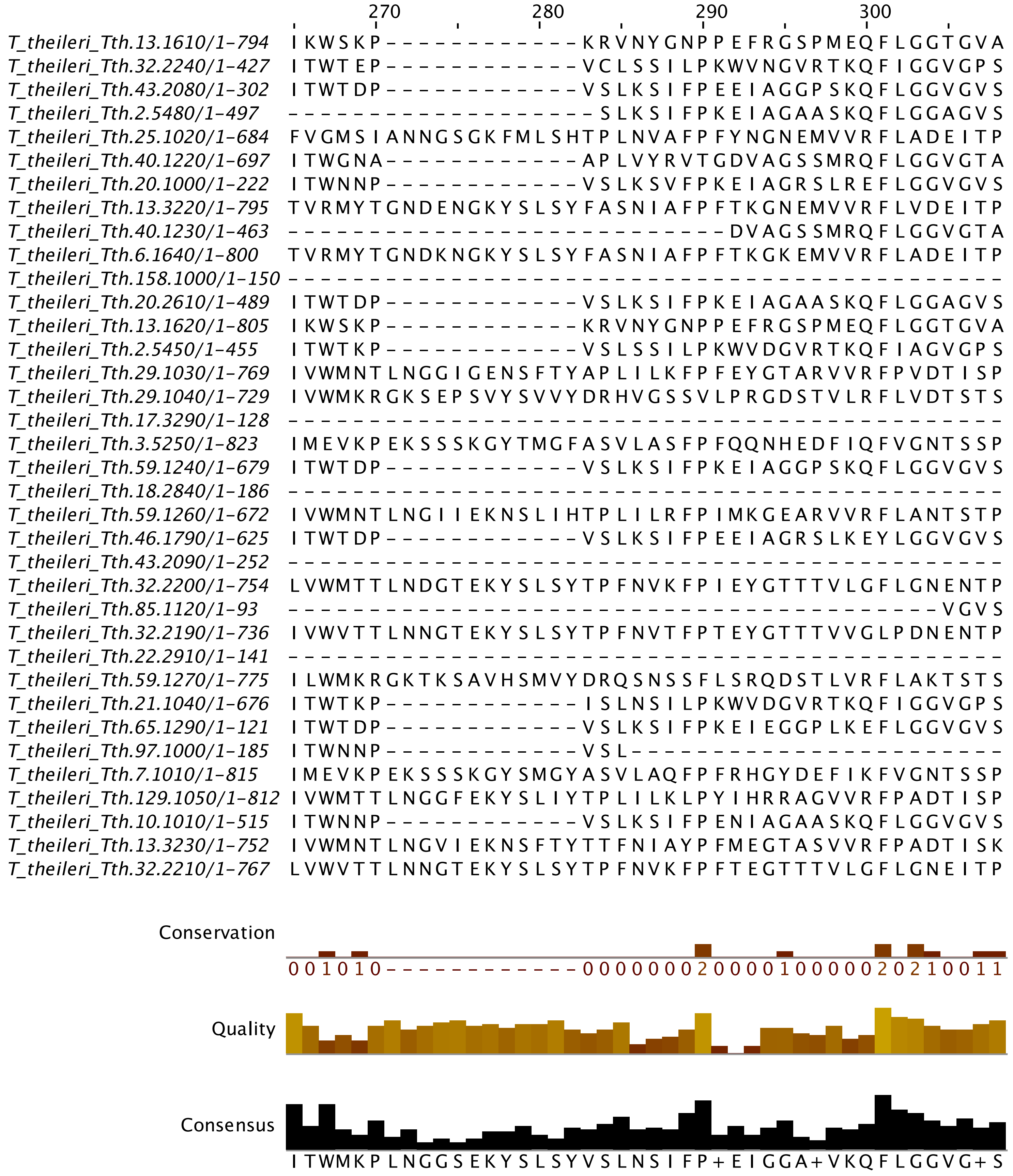
270 280 290 300
T_theileri_Tth.13.1610/1-794 IKWSKP------------KRVNYGNPPEFRGSPMEQFLGGTGVA
T_theileri_Tth.32.2240/1-427 ITWTEP------------VCLSSILPKWVNGVRTKQFIGGVGPS
T_theileri_Tth.43.2080/1-302 ITWTDP------------VSLKSIFPEEIAGGPSKQFLGGVGVS
T_theileri_Tth.2.5480/1-497 -------------------SLKSIFPKEIAGAASKQFLGGAGVS
T_theileri_Tth.25.1020/1-684 FVGMSIANNGSGKFMLSHTPLNVAFPFYNGNEMVVRFLADEITP
T_theileri_Tth.40.1220/1-697 ITWGNA------------APLVYRVTGDVAGSSMRQFLGGVGTA
T_theileri_Tth.20.1000/1-222 ITWNNP------------VSLKSVFPKEIAGRSLREFLGGVGVS
T_theileri_Tth.13.3220/1-795 TVRMYTGNDENGKYSLSYFASNIAFPFTKGNEMVVRFLVDEITP
T_theileri_Tth.40.1230/1-463 ---------------------------DVAGSSMRQFLGGVGTA
T_theileri_Tth.6.1640/1-800 TVRMYTGNDKNGKYSLSYFASNIAFPFTKGKEMVVRFLADEITP
T_theileri_Tth.158.1000/1-150 --
T_theileri_Tth.20.2610/1-489 ITWTDP------------VSLKSIFPKEIAGAASKQFLGGAGVS
T_theileri_Tth.13.1620/1-805 IKWSKP------------KRVNYGNPPEFRGSPMEQFLGGTGVA
T_theileri_Tth.2.5450/1-455 ITWTKP------------VSLSSILPKWVDGVRTKQFIAGVGPS
T_theileri_Tth.29.1030/1-769 IVWMNTLNGGIGENSFTYAPLILKFPFEYGTARVVRFPVDTISP
T_theileri_Tth.29.1040/1-729 IVWMKRGKSEPSVYSVVYDRHVGSSVLPRGDSTVLRFLVDTSTS
T_theileri_Tth.17.3290/1-128 --
T_theileri_Tth.3.5250/1-823 IMEVKPEKSSSKGYTMGFASVLASFPFQQNHEDFIQFVGNTSSP
T_theileri_Tth.59.1240/1-679 ITWTDP------------VSLKSIFPKEIAGGPSKQFLGGVGVS
T_theileri_Tth.18.2840/1-186 --
T_theileri_Tth.59.1260/1-672 IVWMNTLNGIIEKNSLIHTPLILRFPIMKGEARVVRFLANTSTP
T_theileri_Tth.46.1790/1-625 ITWTDP------------VSLKSIFPEEIAGRSLKEYLGGVGVS
T_theileri_Tth.43.2090/1-252 --
T_theileri_Tth.32.2200/1-754 LVWMTTLNDGTEKYSLSYTPFNVKFPIEYGTTTVLGFLGNENTP
T_theileri_Tth.85.1120/1-93 --VGVS
T_theileri_Tth.32.2190/1-736 IVWVTTLNNGTEKYSLSYTPFNVTFPTEYGTTTVVGLPDNENTP
T_theileri_Tth.22.2910/1-141 --
T_theileri_Tth.59.1270/1-775 ILWMKRGKTKSAVHSMVYDRQSNSSFLSRQDSTLVRFLAKTSTS
T_theileri_Tth.21.1040/1-676 ITWTKP------------ISLNSILPKWVDGVRTKQFIGGVGPS
T_theileri_Tth.65.1290/1-121 ITWTDP------------VSLKSIFPKEIEGGPLKEFLGGVGVS
T_theileri_Tth.97.1000/1-185 ITWNNP------------VSL-----------------------
T_theileri_Tth.7.1010/1-815 IMEVKPEKSSSKGYSMGYASVLAQFPFRHGYDEFIKFVGNTSSP
T_theileri_Tth.129.1050/1-812 IVWMTTLNGGFEKYSLIYTPLILKLPYIHRRAGVVRFPADTISP
T_theileri_Tth.10.1010/1-515 ITWNNP------------VSLKSIFPENIAGAASKQFLGGVGVS
T_theileri_Tth.13.3230/1-752 IVWMNTLNGVIEKNSFTYTTFNIAYPFMEGTASVVRFPADTISK
T_theileri_Tth.32.2210/1-767 LVWVTTLNNGTEKYSLSYTPFNVKFPFTEGTTTVLGFLGNEITP
Conservation
001010------------00000002000010000020210011
Quality
Consensus
ITWMKPLNGGSEKYSLSYVSLNSIFP+EIGGA+VKQFLGGVG+S

```
                                   310       320       330       340       350
T_theileri_Tth.13.1610/1-794     IV-IQNGTLVYPIQVKNKRGQFFSTIMYSKDDGETWKIASGLTQ
T_theileri_Tth.32.2240/1-427     IV-ASNGTLLFPVQVSNMDKRVSVSMIYSVDDGATWAFSKGFTP
T_theileri_Tth.43.2080/1-302     IV-TTNGALVFPVQAMSSIRRTTAMIMYSKDDGETWKFANGITA
T_theileri_Tth.2.5480/1-497      IV-TTDGTLVFPVQATNKNKEITAMLMYSKDDGETWKFANGFTE
T_theileri_Tth.25.1020/1-684     ILRMTNYGFVFPVQFLTADDKFRSSVMVDNFEHGDWNVGG-LTD
T_theileri_Tth.40.1220/1-697     IT-TTEGILVYPIQVMNMRNDVVSLVITSADDGKTWRFANGMTD
T_theileri_Tth.20.1000/1-222     IV-TTNGTLVFPVQAMSSIRRTTAMIMYSQDDGETWKFANGITA
T_theileri_Tth.13.3220/1-795     ILRMTNYGFVFPVQFVTTDKKIISTVMVDKFEDDNWNVSS-LTE
T_theileri_Tth.40.1230/1-463     IT-TTEGILVYPIQVMNKKRKVVSLIMYSADDGKTWRFANGMTD
T_theileri_Tth.6.1640/1-800      ILRMTNYGFVFPVQFITAKDKIISTVMVDNFEDDNWNVGS-LTE
T_theileri_Tth.158.1000/1-150    --------------------------------------------
T_theileri_Tth.20.2610/1-489     IV-TTDGTLVFPVQATNKNKEITAMLMYSKDGGETWKFANGITA
T_theileri_Tth.13.1620/1-805     IV-IQNGTLVYPIQVKNKRGQFFSTIMYSKDDGETWKIGSGVEA
T_theileri_Tth.2.5450/1-455      IV-TSNGTLLFPVQVSNFDKRVSVSMIYSVDDGATWAFSKGFTP
T_theileri_Tth.29.1030/1-769     ILTTKNNTLVFPVQFVTEDNKTISSVMYYRPHDTKWKIGGALPD
T_theileri_Tth.29.1040/1-729     ILTMKNNASVFPVQFVTNDNKTISTVMYRRPDEQQWKIGGALPH
T_theileri_Tth.17.3290/1-128     --------------------------------------------
T_theileri_Tth.3.5250/1-823      ILKMNSTTLVFPVQLLSRYNRTAAGVMYFKPDERVWKLDGVVSE
T_theileri_Tth.59.1240/1-679     IV-TTNGTLVFPVQAMNTNKEITAMIMYSKDGGDTWKFANGITA
T_theileri_Tth.18.2840/1-186     ----------------NFDKRVSVSMIYSVDDGATWAFSKGFTP
T_theileri_Tth.59.1260/1-672     ILTTKNNTLLFPVQFVTEDNKVFSTVMYYWPHDTKWNIGGALPD
T_theileri_Tth.46.1790/1-625     IV-TTNGTLVFPVQAMSSIGRTTAMIMYSQDDGETWKFANGITA
T_theileri_Tth.43.2090/1-252     --------------------------------------------
T_theileri_Tth.32.2200/1-754     ILTMKNGTFVFPVQFRTTDEKHVSTVMYYTSHDKQWNIAG-LAD
T_theileri_Tth.85.1120/1-93      IV-TTNGTLVFPVQAMSSIRRTTAMLMYSKDDGETWKFANGITA
T_theileri_Tth.32.2190/1-736     ILTMKNGTFVFPVQLVTTENRFISTVMYYTSHDKQWNIAG-LAD
T_theileri_Tth.22.2910/1-141     --------------------------------------------
T_theileri_Tth.59.1270/1-775     ILTTENNVSVFPVQFVTKNNKIRSTVMYYRPYDTKWNVGG-LTD
T_theileri_Tth.21.1040/1-676     IV-ASNGTLLFPVQVSNFDKRVSVSMIYSVDDGATWAFSKGFTP
T_theileri_Tth.65.1290/1-121     IV-TTNGTLVFPVQAMSSIRRTTAM--------------------
T_theileri_Tth.97.1000/1-185     --------------------------------------------
T_theileri_Tth.7.1010/1-815      ILTMKSNTLVFPVQLLSRYNRSAAGVMYFKPDEKVWKLGGVVSD
T_theileri_Tth.129.1050/1-812    ILTTKNNILLFPVQFVATENKVFSTVMYYTPHDKQWNFGG-LTD
T_theileri_Tth.10.1010/1-515     IV-TTNGTLVFPVQAMNKINGVASMIMYSQDDGNTWKFANGITA
T_theileri_Tth.13.3230/1-752     ILTTKNNTLLFPVQFVTEDNKVFSTVLYYRPHDKQWNVGG-LTD
T_theileri_Tth.32.2210/1-767     ILTMKNGTFVFPVQLVTTENRFISTVMYYTSHDKQWNIAG-LAD

Conservation                     20-0110002222110200010010111000000030120-110
Quality
Consensus                        I+TTTNGTLVFPVQF+NKDNRVTSTVMYSKDDG+TWKFAGGLTD
```

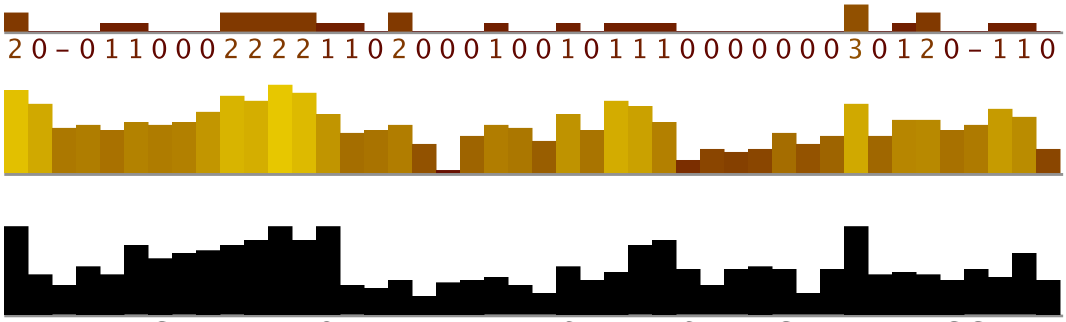

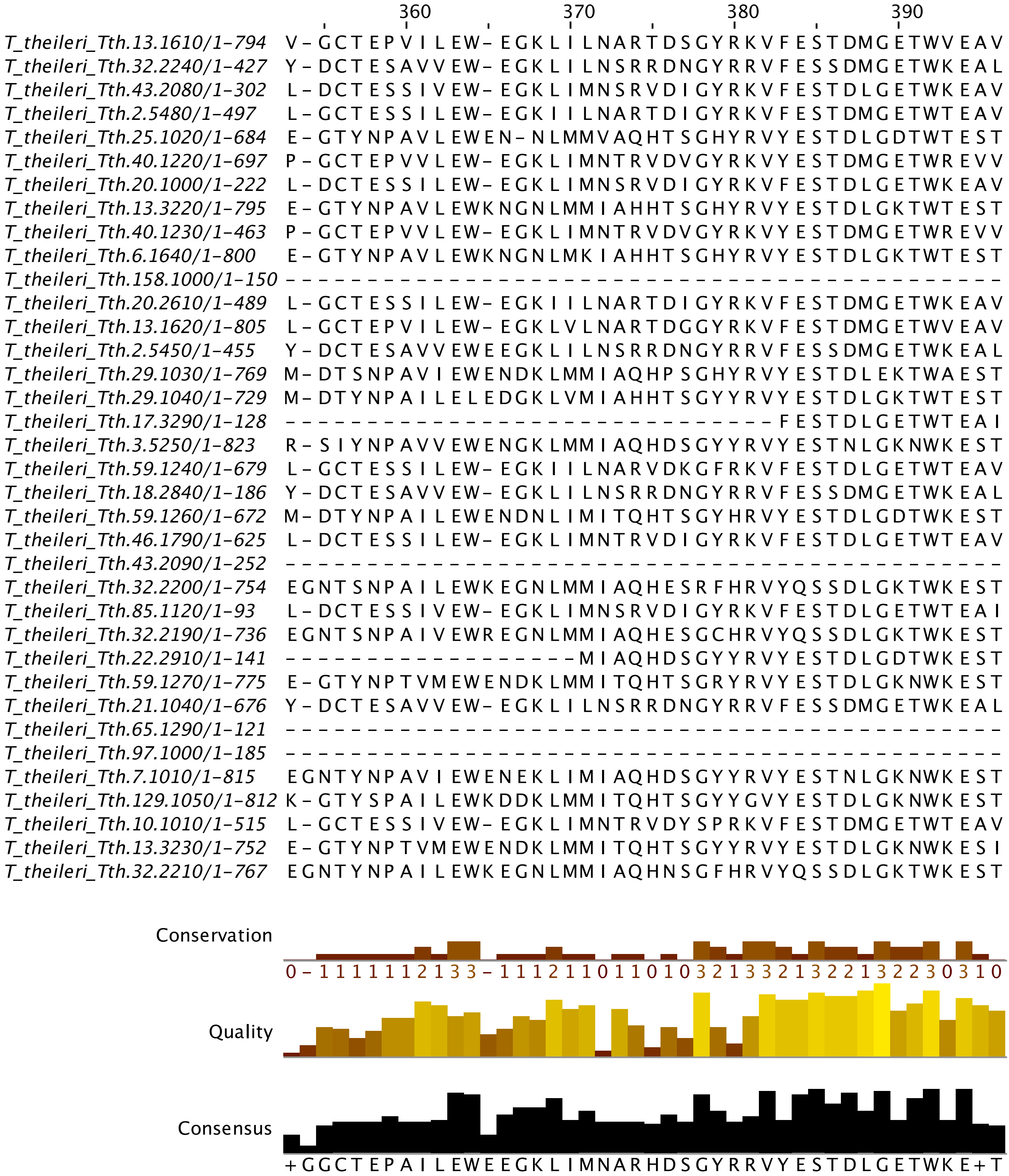

360 370 380 390
T_theileri_Tth.13.1610/1-794 V-GCTEPVILEW-EGKLILNARTDSGYRKVFESTDMGETWVEAV
T_theileri_Tth.32.2240/1-427 Y-DCTESAVVEW-EGKLILNSRRDNGYRRVFESSDMGETWKEAL
T_theileri_Tth.43.2080/1-302 L-DCTESSIVEW-EGKLIMNSRVDIGYRKVFESTDLGETWKEAV
T_theileri_Tth.2.5480/1-497 L-GCTESSILEW-EGKIILNARTDIGYRKVFESTDMGETWTEAV
T_theileri_Tth.25.1020/1-684 E-GTYNPAVLEWEN-NLMMVAQHTSGHYRVYESTDLGDTWTEST
T_theileri_Tth.40.1220/1-697 P-GCTEPVVLEW-EGKLIMNTRVDVGYRKVYESTDMGETWREVV
T_theileri_Tth.20.1000/1-222 L-DCTESSILEW-EGKLIMNSRVDIGYRKVFESTDLGETWKEAV
T_theileri_Tth.13.3220/1-795 E-GTYNPAVLEWKNGNLMMIAHHTSGHYRVYESTDLGKTWTEST
T_theileri_Tth.40.1230/1-463 P-GCTEPVVLEW-EGKLIMNTRVDVGYRKVYESTDMGETWREVV
T_theileri_Tth.6.1640/1-800 E-GTYNPAVLEWKNGNLMKIAHHTSGHYRVYESTDLGKTWTEST
T_theileri_Tth.158.1000/1-150 --
T_theileri_Tth.20.2610/1-489 L-GCTESSILEW-EGKIILNARTDIGYRKVFESTDMGETWKEAV
T_theileri_Tth.13.1620/1-805 L-GCTEPVILEW-EGKLVLNARTDGGYRKVFESTDMGETWVEAV
T_theileri_Tth.2.5450/1-455 Y-DCTESAVVEWEEGKLILNSRRDNGYRRVFESSDMGETWKEAL
T_theileri_Tth.29.1030/1-769 M-DTSNPAVIEWENDKLMMIAQHPSGHYRVYESTDLEKTWAEST
T_theileri_Tth.29.1040/1-729 M-DTYNPAILELEDGKLVMIAHHTSGYYRVYESTDLGKTWTEST
T_theileri_Tth.17.3290/1-128 ------------------------------FESTDLGETWTEAI
T_theileri_Tth.3.5250/1-823 R-SIYNPAVVEWENGKLMMIAQHDSGYYRVYESTNLGKNWKEST
T_theileri_Tth.59.1240/1-679 L-GCTESSILEW-EGKIILNARVDKGFRKVFESTDLGETWTEAV
T_theileri_Tth.18.2840/1-186 Y-DCTESAVVEW-EGKLILNSRRDNGYRRVFESSDMGETWKEAL
T_theileri_Tth.59.1260/1-672 M-DTYNPAILEWENDNLIMITQHTSGYHRVYESTDLGDTWKEST
T_theileri_Tth.46.1790/1-625 L-DCTESSILEW-EGKLIMNTRVDIGYRKVFESTDLGETWTEAV
T_theileri_Tth.43.2090/1-252 --
T_theileri_Tth.32.2200/1-754 EGNTSNPAILEWKEGNLMMIAQHESRFHRVYQSSDLGKTWKEST
T_theileri_Tth.85.1120/1-93 L-DCTESSIVEW-EGKLIMNSRVDIGYRKVFESTDLGETWTEAI
T_theileri_Tth.32.2190/1-736 EGNTSNPAIVEWREGNLMMIAQHESGCHRVYQSSDLGKTWKEST
T_theileri_Tth.22.2910/1-141 -------------------MIAQHDSGYYRVYESTDLGDTWKEST
T_theileri_Tth.59.1270/1-775 E-GTYNPTVMEWENDKLMMITQHTSGRYRVYESTDLGKNWKEST
T_theileri_Tth.21.1040/1-676 Y-DCTESAVVEW-EGKLILNSRRDNGYRRVFESSDMGETWKEAL
T_theileri_Tth.65.1290/1-121 --
T_theileri_Tth.97.1000/1-185 --
T_theileri_Tth.7.1010/1-815 EGNTYNPAVIEWENEKLIMIAQHDSGYYRVYESTNLGKNWKEST
T_theileri_Tth.129.1050/1-812 K-GTYSPAILEWKDDKLMMITQHTSGYYGVYESTDLGKNWKEST
T_theileri_Tth.10.1010/1-515 L-GCTESSIVEW-EGKLIMNTRVDYSPRKVFESTDMGETWTEAV
T_theileri_Tth.13.3230/1-752 E-GTYNPTVMEWENDKLMMITQHTSGYYRVYESTDLGKNWKESI
T_theileri_Tth.32.2210/1-767 EGNTYNPAILEWKEGNLMMIAQHNSGFHRVYQSSDLGKTWKEST
Conservation
0-11111121 33-111211011010321332132213223 0310
Quality
Consensus
+GGCTEPAILEWEEGKLIMNARHDSGYRRVYESTDLGETWKE+T

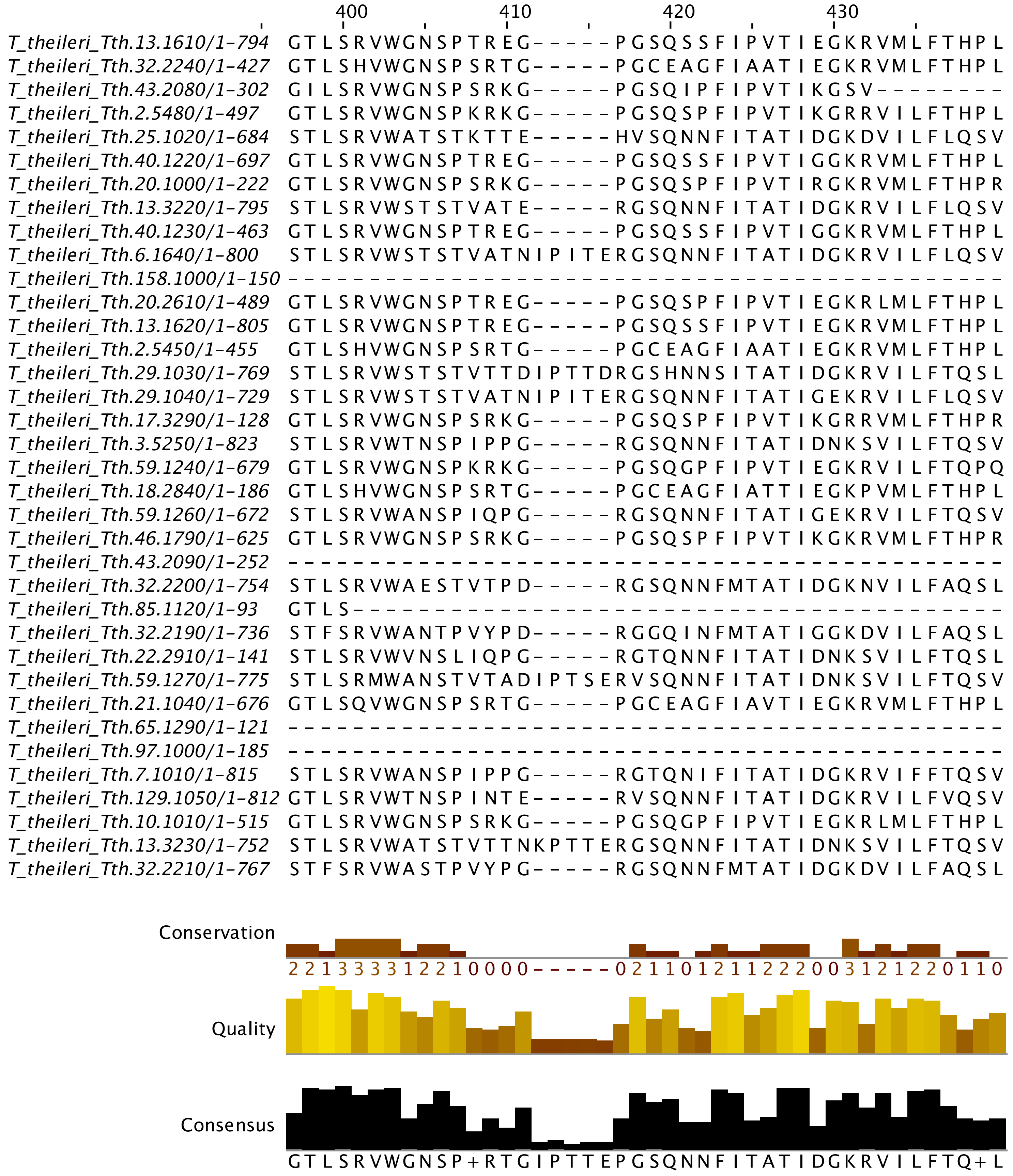
400 410 420 430
T_theileri_Tth.13.1610/1-794 GTLSRVWGNSPTREG-----PGSQSSFIPVTIEGKRVMLFTHPL
T_theileri_Tth.32.2240/1-427 GTLSHVWGNSPSRTG-----PGCEAGFIAATIEGKRVMLFTHPL
T_theileri_Tth.43.2080/1-302 GILSRVWGNSPSRKG-----PGSQIPFIPVTIKGSV--------
T_theileri_Tth.2.5480/1-497 GTLSRVWGNSPKRKG-----PGSQSPFIPVTIKGRRVILFTHPL
T_theileri_Tth.25.1020/1-684 STLSRVWATSTKTTE-----HVSQNNFITATIDGKDVILFLQSV
T_theileri_Tth.40.1220/1-697 GTLSRVWGNSPTREG-----PGSQSSFIPVTIGGKRVMLFTHPL
T_theileri_Tth.20.1000/1-222 GTLSRVWGNSPSRKG-----PGSQSPFIPVTIRGKRVMLFTHPR
T_theileri_Tth.13.3220/1-795 STLSRVWSTSTVATE-----RGSQNNFITATIDGKRVILFLQSV
T_theileri_Tth.40.1230/1-463 GTLSRVWGNSPTREG-----PGSQSSFIPVTIGGKRVMLFTHPL
T_theileri_Tth.6.1640/1-800 STLSRVWSTSTVATNIPITERGSQNNFITATIDGKRVILFLQSV
T_theileri_Tth.158.1000/1-150 --
T_theileri_Tth.20.2610/1-489 GTLSRVWGNSPTREG-----PGSQSPFIPVTIEGKRLMLFTHPL
T_theileri_Tth.13.1620/1-805 GTLSRVWGNSPTREG-----PGSQSSFIPVTIEGKRVMLFTHPL
T_theileri_Tth.2.5450/1-455 GTLSHVWGNSPSRTG-----PGCEAGFIAATIEGKRVMLFTHPL
T_theileri_Tth.29.1030/1-769 STLSRVWSTSTVTTDIPTTDRGSHNNSITATIDGKRVILFTQSL
T_theileri_Tth.29.1040/1-729 STLSRVWSTSTVATNIPITERGSQNNFITATIGEKRVILFLQSV
T_theileri_Tth.17.3290/1-128 GTLSRVWGNSPSRKG-----PGSQSPFIPVTIKGRRVMLFTHPR
T_theileri_Tth.3.5250/1-823 STLSRVWTNSPIPPG-----RGSQNNFITATIDNKSVILFTQSV
T_theileri_Tth.59.1240/1-679 GTLSRVWGNSPKRKG-----PGSQGPFIPVTIEGKRVILFTQPQ
T_theileri_Tth.18.2840/1-186 GTLSHVWGNSPSRTG-----PGCEAGFIATTIEGKPVMLFTHPL
T_theileri_Tth.59.1260/1-672 STLSRVWANSPIQPG-----RGSQNNFITATIGEKRVILFTQSV
T_theileri_Tth.46.1790/1-625 GTLSRVWGNSPSRKG-----PGSQSPFIPVTIKGKRVMLFTHPR
T_theileri_Tth.43.2090/1-252 --
T_theileri_Tth.32.2200/1-754 STLSRVWAESTVTPD-----RGSQNNFMTATIDGKNVILFAQSL
T_theileri_Tth.85.1120/1-93 GTLS--
T_theileri_Tth.32.2190/1-736 STFSRVWANTPVYPD-----RGGQINFMTATIGGKDVILFAQSL
T_theileri_Tth.22.2910/1-141 STLSRVWVNSLIQPG-----RGTQNNFITATIDNKSVILFTQSL
T_theileri_Tth.59.1270/1-775 STLSRMWANSTVTADIPTSERVSQNNFITATIDNKSVILFTQSV
T_theileri_Tth.21.1040/1-676 GTLSQVWGNSPSRTG-----PGCEAGFIAVTIEGKRVMLFTHPL
T_theileri_Tth.65.1290/1-121 --
T_theileri_Tth.97.1000/1-185 --
T_theileri_Tth.7.1010/1-815 STLSRVWANSPIPPG-----RGTQNIFITATIDGKRVIFFTQSV
T_theileri_Tth.129.1050/1-812 GTLSRVWTNSPINTE-----RVSQNNFITATIDGKRVILFVQSV
T_theileri_Tth.10.1010/1-515 GTLSRVWGNSPSRKG-----PGSQGPFIPVTIEGKRLMLFTHPL
T_theileri_Tth.13.3230/1-752 STLSRVWATSTVTTNKPTTERGSQNNFITATIDNKSVILFTQSV
T_theileri_Tth.32.2210/1-767 STFSRVWASTPVYPG-----RGSQNNFMTATIDGKDVILFAQSL
Conservation
221333312210000-----021101211222003121220110
Quality
Consensus
GTLSRVWGNSP+RTGIPTTEPGSQNNFITATIDGKRVILFTQ+L

```
                                                  450       460       470       480
T_theileri_Tth.13.1610/1-794   NFKGKWERDRLHLWLTDNNRIFDVGQISVGDENAAYSSLLYKND
T_theileri_Tth.32.2240/1-427   NFQGAYNRDRLHLWMTDNQRIFDVGQISHGVEKTPYSSLLYTDD
T_theileri_Tth.43.2080/1-302   --------------------------------------------
T_theileri_Tth.2.5480/1-497    NFKGRWNRDRLHLWVTDNNRIFDVGQISIRDENAAYSSLLYKDD
T_theileri_Tth.25.1020/1-684   GSG---EENKLHLWVTDNTRIYHVGLIV-EKKKVKRSTLLFKGD
T_theileri_Tth.40.1220/1-697   NFKGRWERDRLHLWLTDNNRIFDVGQISVGSENAAYSSLLYKDD
T_theileri_Tth.20.1000/1-222   NFKGRWNRDRLHLWVTDNNRIFD---------------------
T_theileri_Tth.13.3220/1-795   GSE---YRKKLYLWVTDNTRIYHVGMIV-ENKKVKRSTLLFKDN
T_theileri_Tth.40.1230/1-463   NFKGRWERDRLHLWLTDNNRIFDVGQISVGSENAAYSSLLYKDD
T_theileri_Tth.6.1640/1-800    GSE---HRKKLYLWVTDNTRIYHVGMIV-ENKKVKRSTLLFKDN
T_theileri_Tth.158.1000/1-150  --------------------------------------------
T_theileri_Tth.20.2610/1-489   NYKGKWERDRLHLWVTDNNRIFDVGQISVGDENAAYSSVLYKDD
T_theileri_Tth.13.1620/1-805   NFKGKWERDRLHLWLTDNNRIFDVGQISVGDENAAYSSLLYKND
T_theileri_Tth.2.5450/1-455    NFQGAYNRDRLHLWMTDNQRIFDVGQISHGVEKTPYSSLLYTDD
T_theileri_Tth.29.1030/1-769   VSG---PQNVFYLWLTDNTHIYYVDFIA-TRPRAATSSLLFKNG
T_theileri_Tth.29.1040/1-729   ETE---EENELHLWVTDKTHINRVGMIV-KDKKVKSSTLLFKDD
T_theileri_Tth.17.3290/1-128   NFKGRWNRDRLHLWLTDNNRIFDIGQISIRDENAAYSSLLYKDG
T_theileri_Tth.3.5250/1-823    ETE---AGNELHLWLTDNTHIYHVGLIS-KAQKETTSTLLFKDD
T_theileri_Tth.59.1240/1-679   NYKESFKRDRLHLWVTDNNRIFNVGQISVDDENAPYSSVLYRDD
T_theileri_Tth.18.2840/1-186   NFQGAYNRDRLHLWMTDNQRIFDVGQISHGVEKTPYSSLLYTDD
T_theileri_Tth.59.1260/1-672   ETE---EENELHLWVTDKTHIYHAGLIV-KDKKVKSSTLLFKDD
T_theileri_Tth.46.1790/1-625   NFKGRWNRDRLHLWVTDNNRIFDIGQISIRDENAAYSSLLYKDD
T_theileri_Tth.43.2090/1-252   --------------------------------------------
T_theileri_Tth.32.2200/1-754   DF----DGIQRHLWLTDDTRIYHVGVIN-KDNKRMFCTLLFKDH
T_theileri_Tth.85.1120/1-93    --------------------------------------------
T_theileri_Tth.32.2190/1-736   DF----DGIQRHLWLTDNTRIYHVGVIN-KDKERMSCTLLFKDH
T_theileri_Tth.22.2910/1-141   VYG---AQNVFYLWLTDNTHIYYVGFIA-TDYNATTSTLLLKDG
T_theileri_Tth.59.1270/1-775   VSG---EEEQLYLWVTDNTRIYYVGLIV-KDKKVKSSTLLFRDN
T_theileri_Tth.21.1040/1-676   NFQGDYNRDRLHLWMTDNQRIFDVGQISYGIEKTPYSSLLYTND
T_theileri_Tth.65.1290/1-121   --------------------------------------------
T_theileri_Tth.97.1000/1-185   --------------------------------------------
T_theileri_Tth.7.1010/1-815    ETE---AGNELHLWLTDNTHIYHVGLIS-KGQKVTTSTLLFKDD
T_theileri_Tth.129.1050/1-812  VSG---EDKELHLWVTDNTRIHHVGLIV-KDKKVKSSTLLFKDD
T_theileri_Tth.10.1010/1-515   NFKGMWNRDRLHLWVTDNNRIFDVGQISVDDENAAYSSLLYRGD
T_theileri_Tth.13.3230/1-752   VSG---EKKQLHLWLTDNTHIYYVGFIA-TDPNAATSTLLFKNG
T_theileri_Tth.32.2210/1-767   DS----EGIERHLWLTDNTRIYHVGVIN-KDKEGIPCTLLFKDH
```

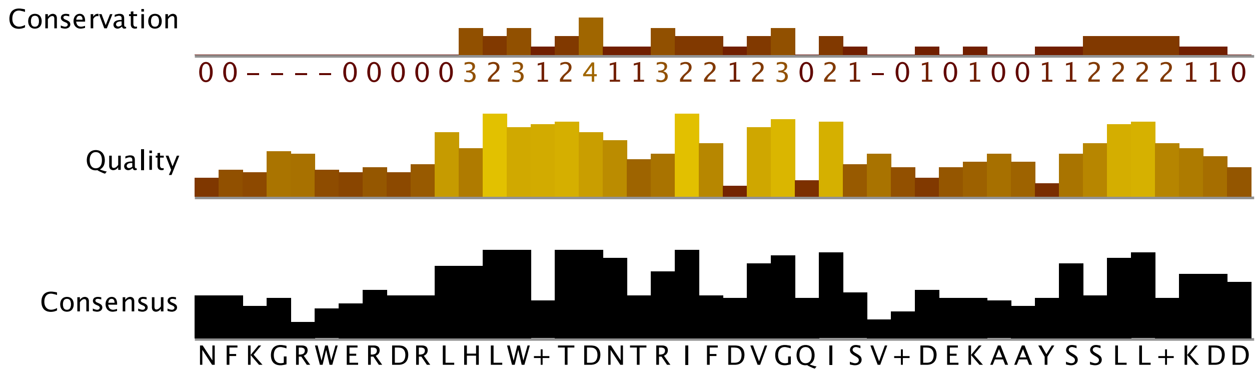

```
                                  490       500       510       520
T_theileri_Tth.13.1610/1-794    KLYCLHEVNHN-ERYSLAFLELKEELNLIKSVVKTWIDQDNNFS
T_theileri_Tth.32.2240/1-427    KLFCLHEIKTEDEIYSIVLSYLENELQLMKSVLNS---------
T_theileri_Tth.43.2080/1-302    --------------------------------------------
T_theileri_Tth.2.5480/1-497     KLYCLHETNLQ-ENYSLVFLELKEELNLIKSVVKTWIKQDKNFS
T_theileri_Tth.25.1020/1-684    KLYCLYETVEGAGKYKVFFVDLTTSMEEIKRVLNKWSKQDALLS
T_theileri_Tth.40.1220/1-697    KLYCLHETNLQ-EIYSIVFVRLIDELNLIKSVVASWVAQDNYFS
T_theileri_Tth.20.1000/1-222    --------------------------------------------
T_theileri_Tth.13.3220/1-795    KLYSLYETVEGGDQHKVIFVDLTTSMEEIKRVLNKWTELDQTLS
T_theileri_Tth.40.1230/1-463    KLYCLHETNLQ-EKYSLVFVRLIDELNLIKSVVASWVAQDNYFS
T_theileri_Tth.6.1640/1-800     KLYCLYETVEGTGKHKVFFLDLTTSMEEIKRVLNKWTELDQTLS
T_theileri_Tth.158.1000/1-150   --------------------------------------------
T_theileri_Tth.20.2610/1-489    KLYCLHEVNIQ-ERYSLIFVELKEELNLIKSVVKTWIEQDKKFS
T_theileri_Tth.13.1620/1-805    KLYCLHEVNHN-ENYSIVFLELKEELNLIKSVVKTWIDQDNNFS
T_theileri_Tth.2.5450/1-455     KLFCLHEIKTEDEIYSIVLSYLENELQLMKSVLTSWKDQDEYLS
T_theileri_Tth.29.1030/1-769    ELYFRYETVEESGKHKVFFVDLTTAMESIKSVVKKWTEQDQILT
T_theileri_Tth.29.1040/1-729    KLYCLYESSKEEGKYTVLFVDLTNKVNKIKEVLNTW---DTIAS
T_theileri_Tth.17.3290/1-128    KLYCLHETNLQ-ENYSLVFLELKEELNLIKSV------------
T_theileri_Tth.3.5250/1-823     KLYCLYESHKEEDNHKVLFVNLTVAMKKIKIVLSTWTNQDTLVS
T_theileri_Tth.59.1240/1-679    KLYILHEKNIR-A-YSLIFLELKEELNLIKSVVKTWIEQDKNFS
T_theileri_Tth.18.2840/1-186    KLFCLHEIKTEDEIYSIVLSYLENELQLMKSVL-----------
T_theileri_Tth.59.1260/1-672    KLYCLYEASKEEGKYEVLFVDLTNKVKEIKEVLNTW---DTITS
T_theileri_Tth.46.1790/1-625    KLYCLHETNLQ-ENYSLVFLELKEELNLIKSVVKTWIKQDKNFS
T_theileri_Tth.43.2090/1-252    --------------------------------------------
T_theileri_Tth.32.2200/1-754    KLYCLYDPRNMEDEHEDFFVELTTAMEDIKRVLTTWTEQDKTLS
T_theileri_Tth.85.1120/1-93     --------------------------------------------
T_theileri_Tth.32.2190/1-736    KLYCLYHPDSMGNEHKEFFVELTTAMEDIKRVLTTWNEQDKTLS
T_theileri_Tth.22.2910/1-141    KLSFRYETGQEEG-YGVLFVDLTTAMESIKGAVKKM---D----
T_theileri_Tth.59.1270/1-775    KLYCLYDTVEGTDKYKVFFVDLTNKVKKIKEVLNKW---DTIAS
T_theileri_Tth.21.1040/1-676    KLFCLHEIKTEDEIYSIVLSHLENELQLMKSVLKSWKSQDEYLS
T_theileri_Tth.65.1290/1-121    --------------------------------------------
T_theileri_Tth.97.1000/1-185    --------------------------------------------
T_theileri_Tth.7.1010/1-815     KLYCLYEAGTPEE-YKVLFVDLTAVMGKIKIVLSTWTNQDTLVS
T_theileri_Tth.129.1050/1-812   KLYFRYETGERKG-YGFLYMDTTTMLEKIKSALTAWTKQDTLVS
T_theileri_Tth.10.1010/1-515    KLYCLHETNLQ-ENYSLVFLEPKEELNLIKSVVKTWIEQDKNFS
T_theileri_Tth.13.3230/1-752    ELYFRYLILEREGNPQVLFVDLSTAMERIKSVVKKWTEQDQTLT
T_theileri_Tth.32.2210/1-767    KLYCLYEAGIENDENRDFSVELTTAMEDIKRVLTTWTEQDKTLS
```

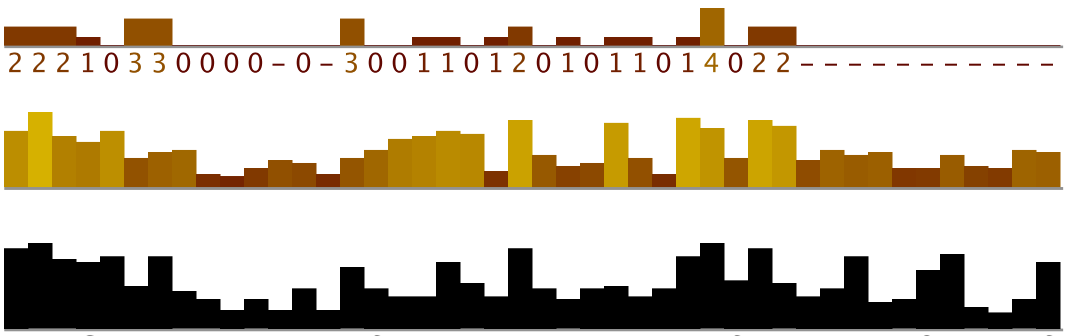

```
Conservation                    2221033000 0-0-300110 1201011014 022-----------
Quality
Consensus                       KLYCLYETNEEEE+YSVVFV+LTTELNLIKSVLKTWTEQDK+LS
```

```
                               530       540       550       560       570
T_theileri_Tth.13.1610/1-794   SICSSTEPKLVAPCEKRCDASFP-TAGLVGFLSDKVNNN-HWRD
T_theileri_Tth.32.2240/1-427   --------------------------------------------
T_theileri_Tth.43.2080/1-302   --------------------------------------------
T_theileri_Tth.2.5480/1-497    DICTPVNPKT-QAGASDCDLPFP-TAGLVGFLSDGGDGK-QWKD
T_theileri_Tth.25.1020/1-684   GKVCP--------SSRQLKNPVP-TVGLVGYLSENRTES-KWND
T_theileri_Tth.40.1220/1-697   HICTPTDPQS-PPRENGCGAPFP-TAGLVGFLSESVEEG-VWHD
T_theileri_Tth.20.1000/1-222   --------------------------------------------
T_theileri_Tth.13.3220/1-795   ESRCCADNSGI--SSRQCKYPVP-TIGLVGYLSGNIDEG-KWND
T_theileri_Tth.40.1230/1-463   HICTPTDPQS-PPRENGCGAPFP-TAGLVGFLSESVEEG-VWHD
T_theileri_Tth.6.1640/1-800    ESRCCADNSGI--SSRQCKYPVP-TIGLVGYLSGNIDEG-KWND
T_theileri_Tth.158.1000/1-150  --------------------------------------------
T_theileri_Tth.20.2610/1-489   DICTSVDPKT-QAGASDCGLPFP-TAGLVGFLSDGGDGK-QWKD
T_theileri_Tth.13.1620/1-805   SICSSTEPKLVAPREKRCDASFP-TAGLVGFLSDKVNNN-HWRD
T_theileri_Tth.2.5450/1-455    GICPSAASVV-PSQKGECN-------------------------
T_theileri_Tth.29.1030/1-769   ESRCCTDE-SC--ASV-CR--IP-TIGLVGYLSGNIDGN-KWKD
T_theileri_Tth.29.1040/1-729   KGICCTDE-AC--TSFDCR--IS-TTKLVGYLSGTIDEESNWKD
T_theileri_Tth.17.3290/1-128   --------------------------------------------
T_theileri_Tth.3.5250/1-823    KCHCHKDEKTC--ATCDSR--IP-TTGLVGYLSGNIDGD-KWKD
T_theileri_Tth.59.1240/1-679   DICTPVDPKKLSVVASDCGLPFP-TAGLVGFLSDGGDSE-QWKD
T_theileri_Tth.18.2840/1-186   --------------------------------------------
T_theileri_Tth.59.1260/1-672   NGICCTDE-AC--TSFDCR--IS-PTKLVGYLSETIDEESNWKD
T_theileri_Tth.46.1790/1-625   DICTPVNPKT-QAGASDCDLPFP-TAGLVGFLSDGGDGK-QWKD
T_theileri_Tth.43.2090/1-252   --------------------------------------------
T_theileri_Tth.32.2200/1-754   GKAC---------TAGQCKYPVS-TIGLVGHLSGKSDGG-KWND
T_theileri_Tth.85.1120/1-93    --------------------------------------------
T_theileri_Tth.32.2190/1-736   GKAC---------TSGQCKYPVS-TTGLVGYLSGKSDGG-EWND
T_theileri_Tth.22.2910/1-141   --------------------------------------------
T_theileri_Tth.59.1270/1-775   KGICCTDK-TC--ASSDCR--IP-TTGLVGYLSGTIDEERIWKD
T_theileri_Tth.21.1040/1-676   GICPSAASVV-PSQKGECNATFP-TRGLVGFLSGEYNKP-YWRD
T_theileri_Tth.65.1290/1-121   --------------------------------------------
T_theileri_Tth.97.1000/1-185   --------------------------------------------
T_theileri_Tth.7.1010/1-815    KCHCYNE--TC--ASSECR--IP-TTGLVGYLSGNIDGD-KWND
T_theileri_Tth.129.1050/1-812  KATNQTLV-SCYLSYLKCKEPIPITTGLVGYLSGNIDGD-KWKD
T_theileri_Tth.10.1010/1-515   DICTPVDPKE-LSGASDCDLPFP-TAGLVGFLSDGGDGK-QWKD
T_theileri_Tth.13.3230/1-752   ESRCCTDE-NC--ASSECR--IP-TTGLVGYLSGNIDGD-KWRD
T_theileri_Tth.32.2210/1-767   GKAC---------TSGQCKYPVS-TIGLVGYLSGKSDGG-EWND
```

Conservation

Quality

Consensus

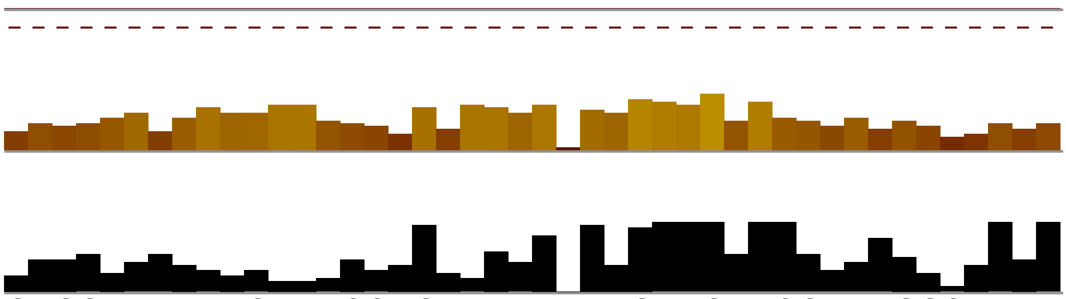

```
GICC+TDPKTCPP+SSDC++PFP-TAGLVGYLSGNIDGGSKWKD
```

```
                                               580       590       600       610
T_theileri_Tth.13.1610/1-794   AYRCVDAEIA-HAEKVP-NGLRFKGGYGGGARWPVSKQGQNQRY
T_theileri_Tth.32.2240/1-427   --------------------------------------------
T_theileri_Tth.43.2080/1-302   --------------------------------------------
T_theileri_Tth.2.5480/1-497    AYRCVDARVK-NAEKVL-NGMKFK-EVGAGAIWPVGKQGQNQRY
T_theileri_Tth.25.1020/1-684   EYLGVNAVVSGTTEKVS-NGLKFN-GVDAGARWPVSINELAKQY
T_theileri_Tth.40.1220/1-697   VYHCMGANVI-NAEKVP-GGFKFK-GGGGGAQWPVGRQGQNQLY
T_theileri_Tth.20.1000/1-222   --------------------------------------------
T_theileri_Tth.13.3220/1-795   EYLGVSASVSGVPEKNP-NGLTLK-GIGAGAKWPVNTDGLAKQY
T_theileri_Tth.40.1230/1-463   VYHCMGANVI-NAEKVP-GGFKFK-GGGGGAQWPVGRQGQNQLY
T_theileri_Tth.6.1640/1-800    EYLGVSASVSGTPEKNP-NGLTLK-GIGAGAKWPVNTDGLAKQY
T_theileri_Tth.158.1000/1-150  --------------------------------------------
T_theileri_Tth.20.2610/1-489   AYRCVDASVK-NAEKVL-NGMKFK-GVGAGAIWPVGKQGQNQRY
T_theileri_Tth.13.1620/1-805   AYRCVDAEIA-HAEKVP-NGLRFKGGYGGGARWPVSKQGQNQRY
T_theileri_Tth.2.5450/1-455    --------------------------------------------
T_theileri_Tth.29.1030/1-769   EYLGVDASVSGITEKVT-NGLKFM-GPGSGAIWPVSTNVLARQY
T_theileri_Tth.29.1040/1-729   EYLGVDASVSGTVKKNP-NGFTFM-GIGAGAMWPVSINEMARQY
T_theileri_Tth.17.3290/1-128   --------------------------------------------
T_theileri_Tth.3.5250/1-823    EYLCVDATVKGAIPRNGFKGLIFK-GADAGAIWPVSTNRAARQY
T_theileri_Tth.59.1240/1-679   AYRCVDASVK-NGEKVL-NGMRFK-GVGAGALWPVGKQGQNQRY
T_theileri_Tth.18.2840/1-186   --------------------------------------------
T_theileri_Tth.59.1260/1-672   EYLGVDASVSGTVKKNP-NGFTFM-GIGAGAMWPVSTDELARQY
T_theileri_Tth.46.1790/1-625   AYRCVDARVK-NAKKVP-NGMKFK-GVGAGALWPVGKQGQNQRY
T_theileri_Tth.43.2090/1-252   --------------------MKFK-GVGAGAIWPVGEQGQNQRY
T_theileri_Tth.32.2200/1-754   EYLCVNAVVSGTTENVS-NGLKFN-GAGAGAVWPVSNGRLAWQY
T_theileri_Tth.85.1120/1-93    --------------------------------------------
T_theileri_Tth.32.2190/1-736   EYLCVNAVVSGTTENVS-NGLKFN-GADAGAMWPVSNGRLARQY
T_theileri_Tth.22.2910/1-141   --------------------------------------------
T_theileri_Tth.59.1270/1-775   EYLCVDASVSGTARKNP-NGFTFM-GIGAGAMWPVSTDGLPRQY
T_theileri_Tth.21.1040/1-676   VYSCVDAFVV-NGARVH-NGLRFA-GIGGGARWPVGKQGQNQRY
T_theileri_Tth.65.1290/1-121   --------------------------------------------
T_theileri_Tth.97.1000/1-185   --------------------------------------------
T_theileri_Tth.7.1010/1-815    EYLCVDATVKGAKSNYEFNGLIFK-GADAGAIWPVSANRLAKQY
T_theileri_Tth.129.1050/1-812  EYLGVNAFVSGITERVT-DGLKFM-GVGAGAMWPVSINGPARQY
T_theileri_Tth.10.1010/1-515   AYRCVDASVK-NAKKVP-NGMRFK-GVGAGALWPVGKQGQNQRY
T_theileri_Tth.13.3230/1-752   EYLGVNAVLIGTVMKVT-NGLKFM-GPGSGAIWPVSINVLARQY
T_theileri_Tth.32.2210/1-767   EYLCVNAVVSGTTEKVS-NGLNFN-GAGAGAVWPLSDGRLARQY
```

Conservation

Quality

Consensus

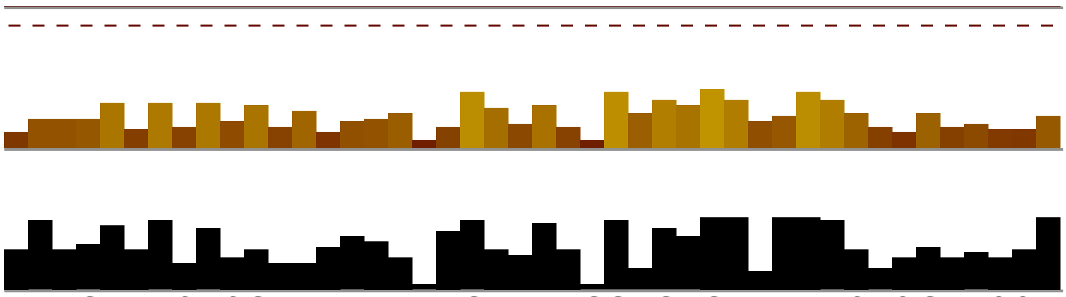

```
                               EYLCVDASVSGTAEKVPFNGLKFKGGVGAGAIWPVSKQG+AQQY
```

```
                              620       630       640       650
T_theileri_Tth.13.1610/1-794  HFVNHVFTLVATVKIDEQPP------------------------
T_theileri_Tth.32.2240/1-427  --------------------------------------------
T_theileri_Tth.43.2080/1-302  --------------------------------------------
T_theileri_Tth.2.5480/1-497   HFANYMFTLVATVXVDGEPLACSGEKLQYDTDSPVVSHFYIGGY
T_theileri_Tth.25.1020/1-684  HFTNYAFTLVATVTIHEVP-------------------------
T_theileri_Tth.40.1220/1-697  HFANYMFTLVATVSVNELP-------------------------
T_theileri_Tth.20.1000/1-222  --------------------------------------------
T_theileri_Tth.13.3220/1-795  YFTNYAFTLAATVTIHEVP-------------------------
T_theileri_Tth.40.1230/1-463  HFANYMFTLVATVSVNELP-------------------------
T_theileri_Tth.6.1640/1-800   YFTNYAFTLAATVTIHEVP-------------------------
T_theileri_Tth.158.1000/1-150 --------------------------------------------
T_theileri_Tth.20.2610/1-489  HFANYMFTLV----------------------------------
T_theileri_Tth.13.1620/1-805  HFVNHVFTLVATVKIDEQPP------------------------
T_theileri_Tth.2.5450/1-455   --------------------------------------------
T_theileri_Tth.29.1030/1-769  HFTNYAFTLVATVTIHKVP-------------------------
T_theileri_Tth.29.1040/1-729  HFTNYAFTLVATVAIHAAP-------------------------
T_theileri_Tth.17.3290/1-128  --------------------------------------------
T_theileri_Tth.3.5250/1-823   HFTNYAFTLVATVTIHEFP-------------------------
T_theileri_Tth.59.1240/1-679  HFANYMFTLVATVQIIDLP-------------------------
T_theileri_Tth.18.2840/1-186  --------------------------------------------
T_theileri_Tth.59.1260/1-672  HFTNYAFTLVATVAIHAAP-------------------------
T_theileri_Tth.46.1790/1-625  HFANYMFTLVATVQIVELP-------------------------
T_theileri_Tth.43.2090/1-252  HFANYMFTLVATVQIIELP-------------------------
T_theileri_Tth.32.2200/1-754  HFANYAFTLAATVTIHAVP-------------------------
T_theileri_Tth.85.1120/1-93   --------------------------------------------
T_theileri_Tth.32.2190/1-736  HFANYAFTLAATVTIHAVP-------------------------
T_theileri_Tth.22.2910/1-141  --------------------------------------------
T_theileri_Tth.59.1270/1-775  HFTNYAFTLVATVAIHAAP-------------------------
T_theileri_Tth.21.1040/1-676  HFANYGFTLAATVSIDEVP-------------------------
T_theileri_Tth.65.1290/1-121  --------------------------------------------
T_theileri_Tth.97.1000/1-185  --------------------------------------------
T_theileri_Tth.7.1010/1-815   HFTNYAFTLAATVTIHEVP-------------------------
T_theileri_Tth.129.1050/1-812 HFTNYAFTLVATVAIHEVP-------------------------
T_theileri_Tth.10.1010/1-515  HFSNYMFTLVATVQIVELP-------------------------
T_theileri_Tth.13.3230/1-752  HFTNYAFTLVATVTIHKVP-------------------------
T_theileri_Tth.32.2210/1-767  HFANYAFTLAATVTIHAVP-------------------------

Conservation                  --------------------------------------------
Quality
Consensus                     HF+NYAFTLVATVTIHEVPP------------------------
```

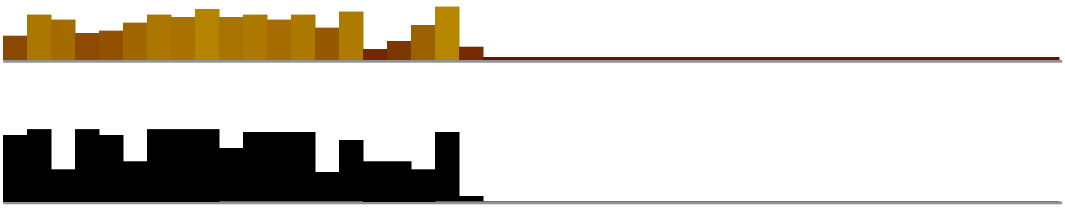

```
                                    670       680       690       700
T_theileri_Tth.13.1610/1-794     -------------------------------------------E
T_theileri_Tth.32.2240/1-427     --------------------------------------------
T_theileri_Tth.43.2080/1-302     --------------------------------------------
T_theileri_Tth.2.5480/1-497      GTSELETDGHVTVTNVLLYNRQLNQSELKTLFLARDQIADNTWX
T_theileri_Tth.25.1020/1-684     --------------------------------------------
T_theileri_Tth.40.1220/1-697     -------------------------------------------A
T_theileri_Tth.20.1000/1-222     --------------------------------------------
T_theileri_Tth.13.3220/1-795     --------------------------------------------
T_theileri_Tth.40.1230/1-463     -------------------------------------------A
T_theileri_Tth.6.1640/1-800      --------------------------------------------
T_theileri_Tth.158.1000/1-150    --------------------------------------------
T_theileri_Tth.20.2610/1-489     --------------------------------------------
T_theileri_Tth.13.1620/1-805     -------------------------------------------E
T_theileri_Tth.2.5450/1-455      --------------------------------------------
T_theileri_Tth.29.1030/1-769     --------------------------------------------
T_theileri_Tth.29.1040/1-729     --------------------------------------------
T_theileri_Tth.17.3290/1-128     --------------------------------------------
T_theileri_Tth.3.5250/1-823      --------------------------------------------
T_theileri_Tth.59.1240/1-679     -------------------------------------------K
T_theileri_Tth.18.2840/1-186     --------------------------------------------
T_theileri_Tth.59.1260/1-672     --------------------------------------------
T_theileri_Tth.46.1790/1-625     -------------------------------------------E
T_theileri_Tth.43.2090/1-252     -------------------------------------------E
T_theileri_Tth.32.2200/1-754     --------------------------------------------
T_theileri_Tth.85.1120/1-93      --------------------------------------------
T_theileri_Tth.32.2190/1-736     --------------------------------------------
T_theileri_Tth.22.2910/1-141     --------------------------------------------
T_theileri_Tth.59.1270/1-775     --------------------------------------------
T_theileri_Tth.21.1040/1-676     -------------------------------------------K
T_theileri_Tth.65.1290/1-121     --------------------------------------------
T_theileri_Tth.97.1000/1-185     --------------------------------------------
T_theileri_Tth.7.1010/1-815      --------------------------------------------
T_theileri_Tth.129.1050/1-812    --------------------------------------------
T_theileri_Tth.10.1010/1-515     -------------------------------------------K
T_theileri_Tth.13.3230/1-752     --------------------------------------------
T_theileri_Tth.32.2210/1-767     --------------------------------------------

Conservation
                                 --------------------------------------------

Quality

Consensus
                                 -------------------------------------------E
```

```
                                  710       720       730       740
T_theileri_Tth.13.1610/1-794   TRVPVPLLGVSM-DESDYYKVLGLSYTHDMKWNPIYGKLYCLS-
T_theileri_Tth.32.2240/1-427   --------------------------------------------
T_theileri_Tth.43.2080/1-302   --------------------------------------------
T_theileri_Tth.2.5480/1-497    VGNPVPLLGVSL-DEAGGKKVLGLSFNSDQKWNPICGKIKAAP-
T_theileri_Tth.25.1020/1-684   KKESSPLLGVRL-DNNEKGLLFGVSYNKDQTWSTIRKDEAEKS-
T_theileri_Tth.40.1220/1-697   DDSATPLLGVSL-DDSGEHKLLGLSYGSNKRWIPIYGKRHVSS-
T_theileri_Tth.20.1000/1-222   --------------------------------------------
T_theileri_Tth.13.3220/1-795   KKENSPLLGVKM-NKSKKRILFGLSFNKDNTWSTINRDESSGP-
T_theileri_Tth.40.1230/1-463   DDSATPLLGVSL-DDSGEHKLLGLSYGSNKRWIPIYGKRHVSS-
T_theileri_Tth.6.1640/1-800    KKESSPFLGVRL-NKSKKRILFGLSFNKDNTWSTINRDEASGP-
T_theileri_Tth.158.1000/1-150  --------------------------------------------
T_theileri_Tth.20.2610/1-489   --------------------------------------------
T_theileri_Tth.13.1620/1-805   TRVPVPLLGVSL-VHHDYYKVLGLSYTHDMEWNPIYGTDSYMR-
T_theileri_Tth.2.5450/1-455    --------------------------------------------
T_theileri_Tth.29.1030/1-769   KNDSSPLLGVRMWDKNKHSILLGVSYNNDKTWSAINRGTKSKS-
T_theileri_Tth.29.1040/1-729   KKGSSPLLGVSL-LGAGHSILLGLSYNKNKTWNTIHRNVVQAS-
T_theileri_Tth.17.3290/1-128   --------------------------------------------
T_theileri_Tth.3.5250/1-823    -TESRPLLGVRL-NKSKKEILLGVSYNKNKTWSTISKSFVKAL-
T_theileri_Tth.59.1240/1-679   AGNPVPLLGVSL-DEAGGKKVLGLSFNSDQQWNPIYGKFEAAP-
T_theileri_Tth.18.2840/1-186   --------------------------------------------
T_theileri_Tth.59.1260/1-672   KKGSSPLLGVSL-KNTRGEILLGLSYNKNKTWSTIHRNVVQAS-
T_theileri_Tth.46.1790/1-625   VGNPVPLLGVSL-DEAGHKKLLGLSFNSDQQWNPICGKIKAAP-
T_theileri_Tth.43.2090/1-252   VGNPVPLLGVSL-DEAGGKKLLGLSFNSDQQWNPICGKIETAP-
T_theileri_Tth.32.2200/1-754   EEDSRPLLGARLWDHTKHIIFLGLSYNKDKTWSTVKNGNEGKAE
T_theileri_Tth.85.1120/1-93    --------------------------------------------
T_theileri_Tth.32.2190/1-736   EEDSRPLLGARLWDHTKHIIFLGLSYNKDKTWSTINRGEGRKVE
T_theileri_Tth.22.2910/1-141   --------------------------------------------
T_theileri_Tth.59.1270/1-775   KKGSSPVLGVRLMDKNGQELPFGLLYNNDKTWSTINRGTKSKS-
T_theileri_Tth.21.1040/1-676   EEGSIPLMGVSV-DESGSKKLLGLSYNFKQQWNPIYGASEIS--
T_theileri_Tth.65.1290/1-121   --------------------------------------------
T_theileri_Tth.97.1000/1-185   --------------------------------------------
T_theileri_Tth.7.1010/1-815    KKDSSPLLGVKLKDRNGQEILLGVSYTNESKWSTVKNGNEGKAE
T_theileri_Tth.129.1050/1-812  KKGSSPLLGVKLKDRNGQEILLGVSYTNESKWSTINRGTTSKS-
T_theileri_Tth.10.1010/1-515   VGNPVPLLGVSL-DEAG---------------------------
T_theileri_Tth.13.3230/1-752   KNDSSPLLGVRMWDNNKHSILLGVSYNNDKTWSATNRGTKSKS-
T_theileri_Tth.32.2210/1-767   EEDSRPLLGARLWG-AERMIFLGVSYNKDKTWSTVKNDNEGKAE
```

Conservation

Quality

Consensus

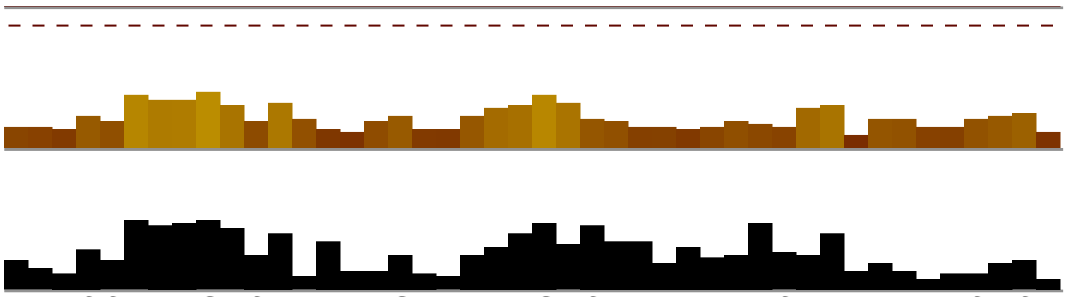

```
KKDSSPLLGVSLWDE+GH+ILLGLSYNKDKTWSTIN+++ESKSE
```

```
                                 750       760       770       780       790
T_theileri_Tth.13.1610/1-794   TGSWELNKTYQVVLSVKENVGSIYIDGRLLGGSRRELK-HRTLT
T_theileri_Tth.32.2240/1-427   --------------------------------------------
T_theileri_Tth.43.2080/1-302   --------------------------------------------
T_theileri_Tth.2.5480/1-497    TGSWELNKAYQVALTFEHGVGSIYVDGEPLACSGEKLQ-YDTDS
T_theileri_Tth.25.1020/1-684   TIKFELQKAYSVILTFKDGKGIVYINGKRI-NKLYYAE-I-QNS
T_theileri_Tth.40.1220/1-697   TQLLVPNKTYQVVLTYQDGVGSVYVDGEPLEESGKKLPGISKEG
T_theileri_Tth.20.1000/1-222   --------------------------------------------
T_theileri_Tth.13.3220/1-795   TGKWEVNKAYSVILTLKDGKAIVYINGIRL-SAPYYAE-M-EGE
T_theileri_Tth.40.1230/1-463   TQLLVPNKTYQVVLTYQDGVGSVYVDGEPLEESGKKLPGISKEG
T_theileri_Tth.6.1640/1-800    TGKWEVNKAYSVILTLKDGKAIVYINGIRL-SAPYYAE-M-EGE
T_theileri_Tth.158.1000/1-150  --------------------------------------------
T_theileri_Tth.20.2610/1-489   --------------------------------------------
T_theileri_Tth.13.1620/1-805   TGSWEVNKTYQVVLTVNKRVGSIYIDGKLLYGSPTSLK-YRVWR
T_theileri_Tth.2.5450/1-455    --------------------------------------------
T_theileri_Tth.29.1030/1-769   TGTWEVDNESSLVLTFKNGTGSVYINSTRL-HVGTNLN-I-EEV
T_theileri_Tth.29.1040/1-729   DIRWEVDRTYTVVLTFANSDCSVQIDGDPM-GFVEHN--FNRET
T_theileri_Tth.17.3290/1-128   --------------------------------------------
T_theileri_Tth.3.5250/1-823    TSKWKENETYSVVLTFDGGKGSVYIDGILI-DERADLK-I-AEK
T_theileri_Tth.59.1240/1-679   TGSWERKKTYQVALTFEHGVGSIYVDGEPLAGSGERLQ-YDTDS
T_theileri_Tth.18.2840/1-186   --------------------------------------------
T_theileri_Tth.59.1260/1-672   DNRWEVDRTYTVVLTFANSDCSVQIDGDPM-GFVKHN--FNRES
T_theileri_Tth.46.1790/1-625   NGSWELNKAYQVALTFEHGVGSIYVDGEPLACSGEKLQ-YDTRS
T_theileri_Tth.43.2090/1-252   NGSWELNKAYQVALTFQDGVGSIYVDGEPLAGSGEKLQ-YDTRS
T_theileri_Tth.32.2200/1-754   IPKWEVDNEYSVILTFENGKGSVYINGTRL-DVGTDLR-IAEEG
T_theileri_Tth.85.1120/1-93    --------------------------------------------
T_theileri_Tth.32.2190/1-736   ILQWEVDNEYSVILTFENGKGSVYINGTRL-DVGTDLR-IAEEE
T_theileri_Tth.22.2910/1-141   --------------------------------------------
T_theileri_Tth.59.1270/1-775   TRTWELNNKYSVILTFENGESSLYINGNRL-DVGTDLR-I-EAN
T_theileri_Tth.21.1040/1-676   TGFWSAKKTYHVVLSMQNDVGSVYVDGEPLPGSGELLM-YNSHS
T_theileri_Tth.65.1290/1-121   --------------------------------------------
T_theileri_Tth.97.1000/1-185   --------------------------------------------
T_theileri_Tth.7.1010/1-815    ILKWEVEKNYSVILTFENGKGSVYINGTRL-DVGTDLK-I-TEK
T_theileri_Tth.129.1050/1-812  TGTWELNNGYSVILTFENRESSVYINGTRL-GVGADLG-IAEEG
T_theileri_Tth.10.1010/1-515   --------------------------------------------
T_theileri_Tth.13.3230/1-752   TGTWEVDNESSLVLTFKNGTGSVYINSTRL-HVGTNLN-I-EEV
T_theileri_Tth.32.2210/1-767   IPKWEVDNEYSVILTFENGKGSVYINGTRL-DVGTDLR-IAQEG
```

Conservation

Quality

Consensus

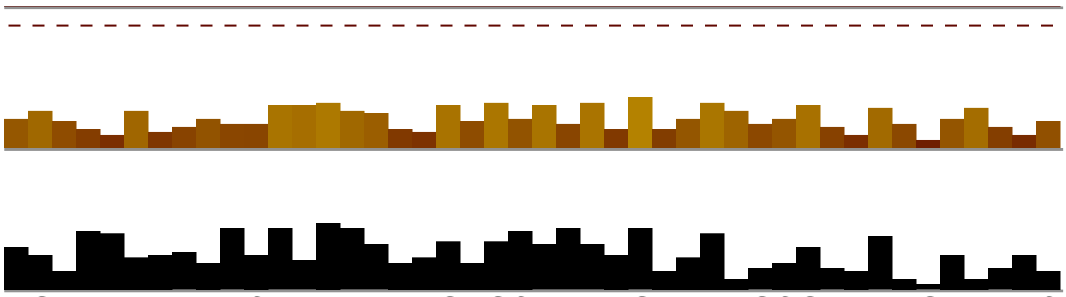

```
                               TGKWEVNKTYSVVLTFENGVGSVYIDG+RLAGSGTDL+GI+EES
```

```
                                800       810       820       830
T_theileri_Tth.13.1610/1-794    TDVSHFYIGGYKNRH--VK----------SD---SRVTVTNVFL
T_theileri_Tth.32.2240/1-427    --------------------------------------------
T_theileri_Tth.43.2080/1-302    --------------------------------------------
T_theileri_Tth.2.5480/1-497     PVVSHF--------------------------------------
T_theileri_Tth.25.1020/1-684    KEVSHFYFGWDGKNNDERSRTIPKPKTSIAGEEIVHITVKDVLL
T_theileri_Tth.40.1220/1-697    IKVSHFYIGGYGSRR--MR----------SD---SHVTVTNVLL
T_theileri_Tth.20.1000/1-222    --------------------------------------------
T_theileri_Tth.13.3220/1-795    EEVSHFYFGSENWGG--------------DN---IHVTIKNVML
T_theileri_Tth.40.1230/1-463    IKVSHFYIGGYGSRR--MR----------SD---SHVTVTNVLL
T_theileri_Tth.6.1640/1-800     EEVSHFYFGSENWGG--------------DN---IHVTIKNVML
T_theileri_Tth.158.1000/1-150   --------------------------------------------
T_theileri_Tth.20.2610/1-489    --------------------------------------------
T_theileri_Tth.13.1620/1-805    PDVSHFYIGGYKRRN--VY----------SD---SRVTVTNVFL
T_theileri_Tth.2.5450/1-455     --------------------------------------------
T_theileri_Tth.29.1030/1-769    VKISHFYFGWDGESV--------------EG---SHISVKDVML
T_theileri_Tth.29.1040/1-729    EEISHFYFGSDGEAG--------------DS---TFATVTNIML
T_theileri_Tth.17.3290/1-128    --------------------------------------------
T_theileri_Tth.3.5250/1-823     EEISHFYFGEYRETV--------------DG---THMTVTDVML
T_theileri_Tth.59.1240/1-679    PVVSHFYIGGYGNRK--LK----------TD---GHVTVTNVLL
T_theileri_Tth.18.2840/1-186    --------------------------------------------
T_theileri_Tth.59.1260/1-672    EEISHFYFGSDGEAG--------------DS---TFATVTNIML
T_theileri_Tth.46.1790/1-625    LAVSHFYIGGYGTSE--LE----------TD---GHVTVTNVLL
T_theileri_Tth.43.2090/1-252    LVVSHFYIGGYGTSE--LE----------TD---GHVTVTNVLL
T_theileri_Tth.32.2200/1-754    EVISDFYFGAYSREDDEVK----------AN---VHITVKDVML
T_theileri_Tth.85.1120/1-93     --------------------------------------------
T_theileri_Tth.32.2190/1-736    KEISHFYFGAYSREDEEVK----------AN---VHITMKDVML
T_theileri_Tth.22.2910/1-141    --------------------------------------------
T_theileri_Tth.59.1270/1-775    EEISHFYFGGYSRENEEVK----------VN---VHISVTNVML
T_theileri_Tth.21.1040/1-676    PGISHFYIGGYGNSS--MK----------TD---SRVTVTNVLL
T_theileri_Tth.65.1290/1-121    --------------------------------------------
T_theileri_Tth.97.1000/1-185    --------------------------------------------
T_theileri_Tth.7.1010/1-815     KEITHFYFGEYSEGG--------------KGNVIVNMSVKDVML
T_theileri_Tth.129.1050/1-812   EEISHFYFGAYSDKVPRTIHILQAKPE--ET---VHISVKDVML
T_theileri_Tth.10.1010/1-515    --------------------------------------------
T_theileri_Tth.13.3230/1-752    VKISHFYFGWDGESV--------------EG---SHISVKDVML
T_theileri_Tth.32.2210/1-767    EVISHFYFGAYSREDEEVK----------AN---VHITVKDVML
```

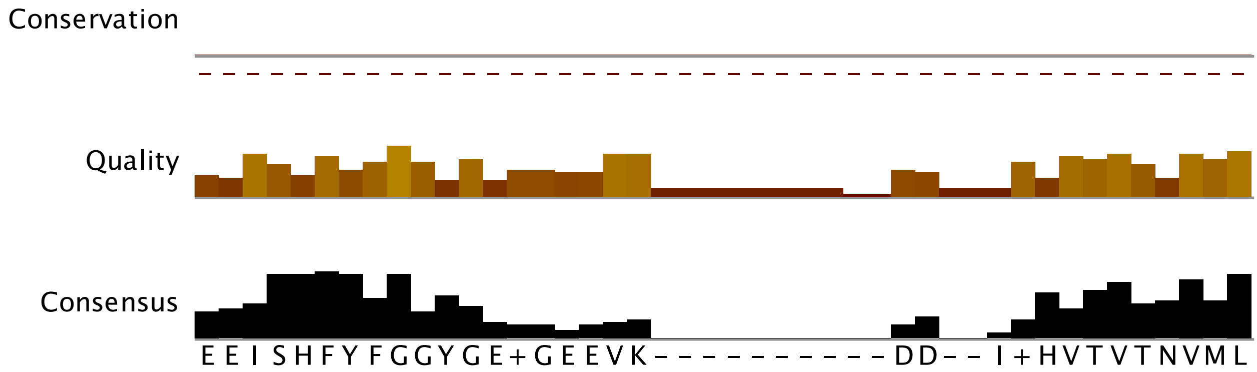

```
                                 840       850       860       870
T_theileri_Tth.13.1610/1-794     YNRELSQGEINTLLLNKDTDVAA---------------------
T_theileri_Tth.32.2240/1-427     --------------------------------------------
T_theileri_Tth.43.2080/1-302     --------------------------------------------
T_theileri_Tth.2.5480/1-497      --------------------------------------------
T_theileri_Tth.25.1020/1-684     YNRALDTEEIDVLVKNKENGNAL---------------------
T_theileri_Tth.40.1220/1-697     YNRQLNQNEVKTVFLNRNNIAVATEVVPYLQRGSIIPILSV---
T_theileri_Tth.20.1000/1-222     --------------------------------------------
T_theileri_Tth.13.3220/1-795     YNRVLLRREINALVENNMSIVVADAKKE-ISEQ-----------
T_theileri_Tth.40.1230/1-463     YNRQLNQNEVKTVFLNRN--------------------------
T_theileri_Tth.6.1640/1-800      YNRVLLRREINALVKNNMSIVVADGEKE-ISEQ-----------
T_theileri_Tth.158.1000/1-150    --------------------------------------------
T_theileri_Tth.20.2610/1-489     --------------------------------------------
T_theileri_Tth.13.1620/1-805     YNRALSQDEINTLLLNKGSDVEA---------------------
T_theileri_Tth.2.5450/1-455      --------------------------------------------
T_theileri_Tth.29.1030/1-769     YNRVLSNEEIDVLLRSRMNISFSPSDAEQIQEE---TNDKSS--
T_theileri_Tth.29.1040/1-729     YNSVLTQTEIDALIKKKLVISLSESVKQMLSEE-----------
T_theileri_Tth.17.3290/1-128     --------------------------------------------
T_theileri_Tth.3.5250/1-823      YNRVLKNIEIDALFGNKVNTSLPQSVTKTMAEE---LFKQSNND
T_theileri_Tth.59.1240/1-679     YNRQLNQSELKTLFLARDFISSN---------------------
T_theileri_Tth.18.2840/1-186     --------------------------------------------
T_theileri_Tth.59.1260/1-672     YNGVLTPTEIDALIKKKLVISLSESVKQMLTEE-----------
T_theileri_Tth.46.1790/1-625     YNRQLNQSELKT--------------------------------
T_theileri_Tth.43.2090/1-252     YNRQLNQSELKTLFLARDQIADNTWG------------------
T_theileri_Tth.32.2200/1-754     YNRGLLPAEIDALIKNKANVLLSESEIKRISGQ-----------
T_theileri_Tth.85.1120/1-93      --------------------------------------------
T_theileri_Tth.32.2190/1-736     YNRGLLPTEIDALVKNKANVLLSESEIKRISGQ-----------
T_theileri_Tth.22.2910/1-141     --------------------------------------------
T_theileri_Tth.59.1270/1-775     YNRVLLSAEIDELVKNKANVLLSESEIKGNSEQGTATTADTGNK
T_theileri_Tth.21.1040/1-676     YNRELSHDEIRTLFLGSEQIKAVTL-------------------
T_theileri_Tth.65.1290/1-121     --------------------------------------------
T_theileri_Tth.97.1000/1-185     --------------------------------------------
T_theileri_Tth.7.1010/1-815      YNRVLSNEEIDTLLRSRMNISFEDRDGEKILQD---ILNRPGVV
T_theileri_Tth.129.1050/1-812    YNRVLSREEIDVLHKSKMSTLYGARDAEEILKK---IMSRPNVV
T_theileri_Tth.10.1010/1-515     --------------------------------------------
T_theileri_Tth.13.3230/1-752     YNRVLSKEEIAALLRSRMNISFSPSDAEEIQEG---TNDKSS--
T_theileri_Tth.32.2210/1-767     YNRGLLPTEIDALIKNKANVLLSESEIKRISEQ-----------

Conservation                     --------------------------------------------

Quality

Consensus
                                 YNRVLSQEEID+LFKNKMNISLSESE+ERISEQ---TND+S++V
```

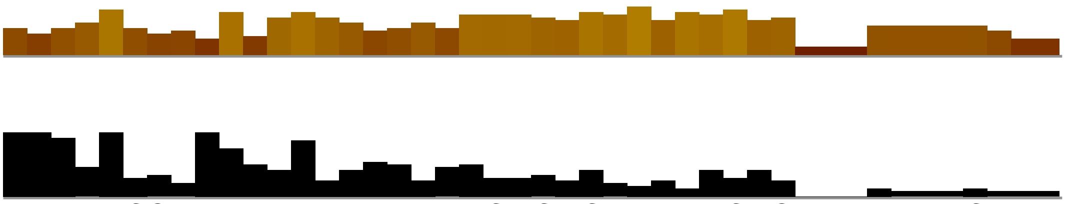

```
                                            890       900       910       920
T_theileri_Tth.13.1610/1-794    ----------------------IKKKPTE----GNVSPPQREED
T_theileri_Tth.32.2240/1-427    --------------------------------------------
T_theileri_Tth.43.2080/1-302    --------------------------------------------
T_theileri_Tth.2.5480/1-497     --------------------------------------------
T_theileri_Tth.25.1020/1-684    --------------------------------------------
T_theileri_Tth.40.1220/1-697    ----------------------APTTPAPMTDNTSTHLHGENLP
T_theileri_Tth.20.1000/1-222    --------------------------------------------
T_theileri_Tth.13.3220/1-795    --------------------ASMTDSSQSPVIHSPSRPQSTSSR
T_theileri_Tth.40.1230/1-463    --------------------------------------------
T_theileri_Tth.6.1640/1-800     --------------------ASMTDSSQSPVIHSPSRPQSTSSR
T_theileri_Tth.158.1000/1-150   --------------------------------------------
T_theileri_Tth.20.2610/1-489    --------------------------------------------
T_theileri_Tth.13.1620/1-805    ----------------------IKKKPTEVVPRDSVRPPKRKEN
T_theileri_Tth.2.5450/1-455     --------------------------------------------
T_theileri_Tth.29.1030/1-769    ----------QEQNTPQSSTTSTPAK----TPVATVQPESQPES
T_theileri_Tth.29.1040/1-729    --------------------IKMQSTLPTPATSSTAGPHSMKKP
T_theileri_Tth.17.3290/1-128    --------------------------------------------
T_theileri_Tth.3.5250/1-823     THTMVREVKIQHQNT-TSQNNDTSIRTEATVDLPAALSESAAES
T_theileri_Tth.59.1240/1-679    ----------------------KPEGSGS---------------
T_theileri_Tth.18.2840/1-186    --------------------------------------------
T_theileri_Tth.59.1260/1-672    --------------------IKMQSTLPTPATSSTAGPQSIKKP
T_theileri_Tth.46.1790/1-625    --------------------------------------------
T_theileri_Tth.43.2090/1-252    ----------------------VDDDSSTSGSSSESSAPEEASS
T_theileri_Tth.32.2200/1-754    ----------------------ATASHQNSATSSIAGAEPTKDT
T_theileri_Tth.85.1120/1-93     --------------------------------------------
T_theileri_Tth.32.2190/1-736    ----------------------ATASHQNPATSTTAGADPTKDT
T_theileri_Tth.22.2910/1-141    --------------------------------------------
T_theileri_Tth.59.1270/1-775    NSGEEDNTKNTEAAV--SQDNATSTQ----AATTAVRPETGTET
T_theileri_Tth.21.1040/1-676    --------------------------------------------
T_theileri_Tth.65.1290/1-121    --------------------------------------------
T_theileri_Tth.97.1000/1-185    --------------------------------------------
T_theileri_Tth.7.1010/1-815     KSSPLE-------ATETPQNSTTSTPAKTTNASAPVIPESQPSP
T_theileri_Tth.129.1050/1-812   NSPPLEATKIQQNAT--SQDNATPTQTETP--TATLQPEPSTEV
T_theileri_Tth.10.1010/1-515    --------------------------------------------
T_theileri_Tth.13.3230/1-752    ----------QVQNTETPQDIDTPTQMETTNASATVQPESQPES
T_theileri_Tth.32.2210/1-767    ----------------------VTASHQNSATSTTAGAEPTKDT
```

Conservation

```
--------------------------------------------
```

Quality

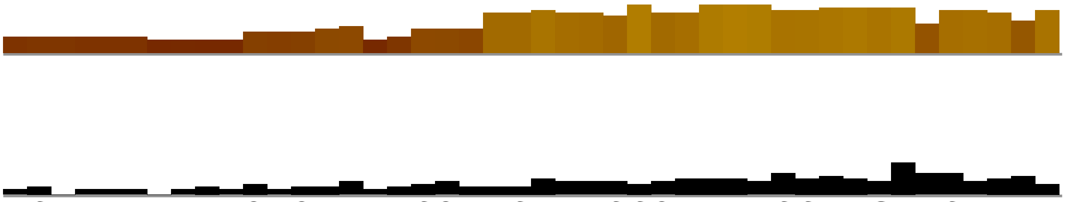

Consensus

```
NS-PLE-TKIQEQ+TETSQD+ST+TSSQTPATSST+GPESTKE+
```

```
                                     930       940       950       960
T_theileri_Tth.13.1610/1-794   KPGAETRQKRSTDGTSSSNNIGTAVDAARDGDDMRNDVRSETPE
T_theileri_Tth.32.2240/1-427   --------------------------------------------
T_theileri_Tth.43.2080/1-302   --------------------------------------------
T_theileri_Tth.2.5480/1-497    --------------------------------------------
T_theileri_Tth.25.1020/1-684   --------------------------------------------
T_theileri_Tth.40.1220/1-697   SPGALPVQGSTSITTSNGGKVVAGLNTSEIPDPE----------
T_theileri_Tth.20.1000/1-222   --------------------------------------------
T_theileri_Tth.13.3220/1-795   EYRFNERQANSQTADPLRRQGGRRRRSAET--------------
T_theileri_Tth.40.1230/1-463   --------------------------------------------
T_theileri_Tth.6.1640/1-800    EYRFNERQGNSQTADSLRRQGG-RRRSPET--------------
T_theileri_Tth.158.1000/1-150  --------------------------------------------
T_theileri_Tth.20.2610/1-489   --------------------------------------------
T_theileri_Tth.13.1620/1-805   KPRAETRQKRSTDGTSSSNNIGTAVDAARGGNDMRNDVWTETPE
T_theileri_Tth.2.5450/1-455    --------------------------------------------
T_theileri_Tth.29.1030/1-769   ---QENQQPAPVVDIPPEKTQQEVNTTENK--------------
T_theileri_Tth.29.1040/1-729   QNTNSGNQGNA---------------------------------
T_theileri_Tth.17.3290/1-128   --------------------------------------------
T_theileri_Tth.3.5250/1-823    APQQESQSPVTETYETQENQQHEQNATEKR--------------
T_theileri_Tth.59.1240/1-679   --------------------------------------------
T_theileri_Tth.18.2840/1-186   --------------------------------------------
T_theileri_Tth.59.1260/1-672   QNTNSG--------------------------------------
T_theileri_Tth.46.1790/1-625   --------------------------------------------
T_theileri_Tth.43.2090/1-252   SESHNTEEWDS---------------------------------
T_theileri_Tth.32.2200/1-754   GNTISGGQANSQITVEL---------------------------
T_theileri_Tth.85.1120/1-93    --------------------------------------------
T_theileri_Tth.32.2190/1-736   VNTISGGQANSQISVEL---------------------------
T_theileri_Tth.22.2910/1-141   --------------------------------------------
T_theileri_Tth.59.1270/1-775   APQRENATSVNNTHQTQ---------------------------
T_theileri_Tth.21.1040/1-676   --------------------------------------------
T_theileri_Tth.65.1290/1-121   --------------------------------------------
T_theileri_Tth.97.1000/1-185   --------------------------------------------
T_theileri_Tth.7.1010/1-815    ---EPASRIVPEVKESPEKPQQKEKIPENK--------------
T_theileri_Tth.129.1050/1-812  ATQSENKTAVTATAQTQEN-QHSTQSTEKQEQNKAPEALNESTH
T_theileri_Tth.10.1010/1-515   --------------------------------------------
T_theileri_Tth.13.3230/1-752   ---QENQQPAPVVEIPPEKTQQEVNTTENK--------------
T_theileri_Tth.32.2210/1-767   GNTNSGGQANSQITVEL---------------------------
```

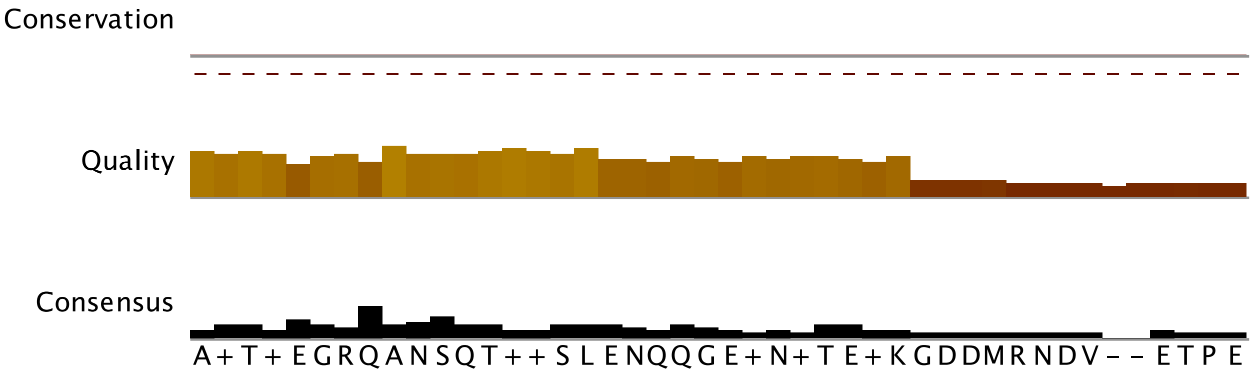

```
                              970       980       990       1000      1010
T_theileri_Tth.13.1610/1-794  SKNEEPPKN--------TIGGAHESTATPTETTDQMECMFSSYL
T_theileri_Tth.32.2240/1-427  --------------------------------------------
T_theileri_Tth.43.2080/1-302  --------------------------------------------
T_theileri_Tth.2.5480/1-497   --------------------------------------------
T_theileri_Tth.25.1020/1-684  --------------------------------------------
T_theileri_Tth.40.1220/1-697  --------------------------------------------
T_theileri_Tth.20.1000/1-222  --------------------------------------------
T_theileri_Tth.13.3220/1-795  ----------------PKTEDASENNMQKHSSSKTTLPNIHDN-
T_theileri_Tth.40.1230/1-463  --------------------------------------------
T_theileri_Tth.6.1640/1-800   ----------------LETEDASENNMQE-DPSKTILPNIHDN-
T_theileri_Tth.158.1000/1-150 --------------------------------------------
T_theileri_Tth.20.2610/1-489  --------------------------------------------
T_theileri_Tth.13.1620/1-805  SKNEEPPKNTIDDTIDDTIDGAHESTETPTETTDQIAGALNAYL
T_theileri_Tth.2.5450/1-455   --------------------------------------------
T_theileri_Tth.29.1030/1-769  ------------TSTETTVPVSSTNTKQQDLLQNTTQSMINNNV
T_theileri_Tth.29.1040/1-729  --------------------------------------------
T_theileri_Tth.17.3290/1-128  --------------------------------------------
T_theileri_Tth.3.5250/1-823   ------------TFSSTPVPVSSTNT-TQDLLQKTNHLMINNTV
T_theileri_Tth.59.1240/1-679  -------------------------------------------L
T_theileri_Tth.18.2840/1-186  --------------------------------------------
T_theileri_Tth.59.1260/1-672  --------------------------------------------
T_theileri_Tth.46.1790/1-625  --------------------------------------------
T_theileri_Tth.43.2090/1-252  -------------------------------------------L
T_theileri_Tth.32.2200/1-754  -------------------------------------------A
T_theileri_Tth.85.1120/1-93   --------------------------------------------
T_theileri_Tth.32.2190/1-736  -------------------------------------------A
T_theileri_Tth.22.2910/1-141  --------------------------------------------
T_theileri_Tth.59.1270/1-775  -----------------------------------GEALINKNV
T_theileri_Tth.21.1040/1-676  --------------------------------------------
T_theileri_Tth.65.1290/1-121  --------------------------------------------
T_theileri_Tth.97.1000/1-185  --------------------------------------------
T_theileri_Tth.7.1010/1-815   -----------ATSTKTTAPAHISNRIQEH-LQNTTQSNTIRDV
T_theileri_Tth.129.1050/1-812 AEPPEKDETTEATTTGTTVPVSSTNTMQQDLLRNTTQSMINNNV
T_theileri_Tth.10.1010/1-515  --------------------------------------------
T_theileri_Tth.13.3230/1-752  ------------TSTETTVPDSSTNTMQQDLLQNTTQSMINNNV
T_theileri_Tth.32.2210/1-767  -------------------------------------------A
```

Conservation

Quality

Consensus

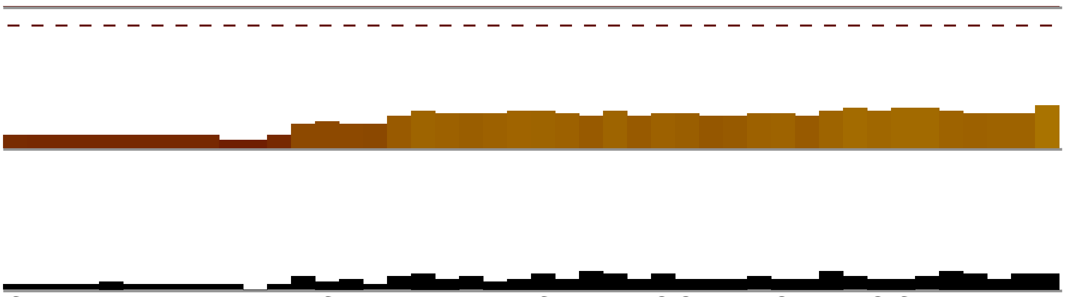

```
SKNEEPPKNT-ATSTETTVP++S+NTMQQDLLQNTTQSMINNNV
```

```
                                  1020      1030      1040
T_theileri_Tth.13.1610/1-794   NGELDSLICDGSFYFVPRLPLLALLGSTLIVALF-
T_theileri_Tth.32.2240/1-427   -----------------------------------
T_theileri_Tth.43.2080/1-302   -----------------------------------
T_theileri_Tth.2.5480/1-497    -----------------------------------
T_theileri_Tth.25.1020/1-684   -----------------------------------
T_theileri_Tth.40.1220/1-697   -----------------------------------
T_theileri_Tth.20.1000/1-222   -----------------------------------
T_theileri_Tth.13.3220/1-795   NTHPQNTIDGTARVYGYGLPMLVVGLWALATILL-
T_theileri_Tth.40.1230/1-463   -----------------------------------
T_theileri_Tth.6.1640/1-800    NTHPQNTIDGTVRVYGYGLPILLLGLWALATSLL-
T_theileri_Tth.158.1000/1-150  -----------------------------------
T_theileri_Tth.20.2610/1-489   -----------------------------------
T_theileri_Tth.13.1620/1-805   NGKSELPTGDGSFYFVPRLPLLALLGSTFIVALF-
T_theileri_Tth.2.5450/1-455    -----------------------------------
T_theileri_Tth.29.1030/1-769   NAHKNKSIDGTVRIYESVLPMLVLGLWALATVFP-
T_theileri_Tth.29.1040/1-729   -TNRKQGSDGTVSIYASVLPMLVLGLWALATVFP-
T_theileri_Tth.17.3290/1-128   -----------------------------------
T_theileri_Tth.3.5250/1-823    NAHKNKTIDGTVRIYESVLPMLVLGLWALATVFP-
T_theileri_Tth.59.1240/1-679   ETDGSTLCFGVNCFSMMLLPLLTLALVLFDHGIFI
T_theileri_Tth.18.2840/1-186   -----------------------------------
T_theileri_Tth.59.1260/1-672   ----NQTIDGTVRIYESVLSMLLLGLWALATVLP-
T_theileri_Tth.46.1790/1-625   -----------------------------------
T_theileri_Tth.43.2090/1-252   ETDGSTLCFGVNYFSMMLLPLLTLALVLFDHGIFI
T_theileri_Tth.32.2200/1-754   KAHQN-TIDGTVRVYGHGLPILLLGLWALATVLL-
T_theileri_Tth.85.1120/1-93    -----------------------------------
T_theileri_Tth.32.2190/1-736   KAHQN-TIDGTVRVYGHGLPILLLGLWALATSLL-
T_theileri_Tth.22.2910/1-141   -----------------------------------
T_theileri_Tth.59.1270/1-775   KARQNKTVDGTVRIYESVLPMLLLGLWALATVLP-
T_theileri_Tth.21.1040/1-676   -----------------------------------
T_theileri_Tth.65.1290/1-121   -----------------------------------
T_theileri_Tth.97.1000/1-185   -----------------------------------
T_theileri_Tth.7.1010/1-815    KARQNKTIDGTVRIYESVLPILLLGLWALATSLL-
T_theileri_Tth.129.1050/1-812  NAHKNKSIDGTVRIYESVLPMLLLGLWALVTVFP-
T_theileri_Tth.10.1010/1-515   -----------------------------------
T_theileri_Tth.13.3230/1-752   NAHKNKSIDGTVRIYESVLPMLLLVLWALATVFP-
T_theileri_Tth.32.2210/1-767   KAHQN-TIDGTVRVYGYGLPILLLGLWALATILL-
```

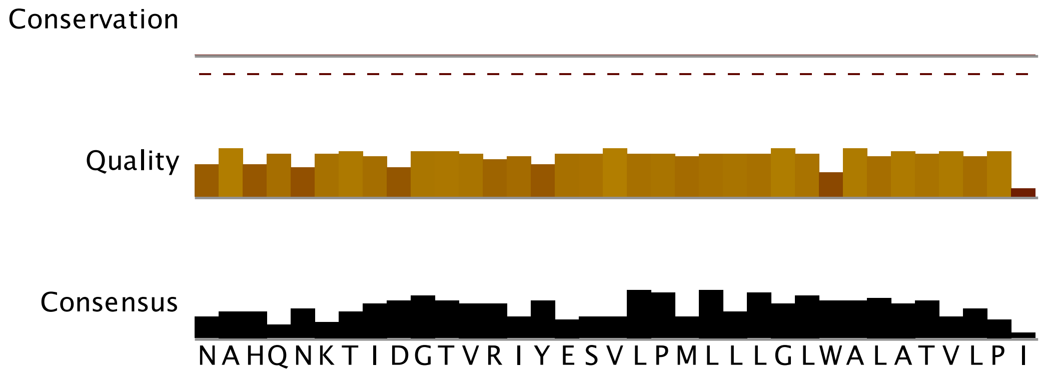

Supplement: Supplementary figure_5 [file evx152_suppfig_5.pdf]
