# Supplementary material for: An Alternative Strategy for Trypanosome Survival in the Mammalian Bloodstream Revealed through Genome and Transcriptome Analysis of the Ubiquitous Bovine Parasite Trypanosoma (Megatrypanum) theileri
Source: Genome Biol Evol. 2017 Aug 14;9(8):2093–109. doi: 10.1093/gbe/evx152 (PMC5737535; doi:10.1093/gbe/evx152)
Supplement: Supplementary figure_6 [file evx152_suppfig_6.pdf]

```
                                       10        20        30        40
T_theileri_Tth.54.1380/1-425   --------------------------------------------
T_theileri_Tth.107.1000/1-244  --------------------------------------------
T_theileri_Tth.54.1340/1-680   --------------------------------------------
T_theileri_Tth.117.1040/1-195  --------------------------------------------
T_theileri_Tth.6.5060/1-554    --------------------------------------------
T_theileri_Tth.1.6360/1-511    --------------------------------------------
T_theileri_Tth.46.1110/1-516   --------------------------------------------
T_theileri_Tth.33.1800/1-520   --------------------------------------------
T_theileri_Tth.46.1020/1-842   --------------------------------------------
T_theileri_Tth.70.1140/1-882   --------------------------------------------
T_theileri_Tth.87.1050/1-130   --------------------------------------------
T_theileri_Tth.144.1010/1-374  --------------------------------------------
T_theileri_Tth.39.1000/1-654   --------------------------------------------
T_theileri_Tth.12.1010/1-802   --------------------------------------------
T_theileri_Tth.11.3500/1-528   --------------------------------------------
T_theileri_Tth.124.1020/1-215  --------------------------------------------
T_theileri_Tth.54.1390/1-443   --------------------------------------------
T_theileri_Tth.26.2480/1-314   --------------------------------------------
T_theileri_Tth.43.1970/1-318   --------------------------------------------
T_theileri_Tth.37.1300/1-102   --------------------------------------------
T_theileri_Tth.11.2410/1-509   --------------------------------------------
T_theileri_Tth.70.1150/1-914   --------------------------------------------
T_theileri_Tth.31.1050/1-313   --------------------------------------------
T_theileri_Tth.70.1010/1-670   --------------------------------------------
T_theileri_Tth.36.2090/1-806   --------------------------------------------
T_theileri_Tth.117.1050/1-116  --------------------------------------------
T_theileri_Tth.11.3480/1-173   --------------------------------------------
T_theileri_Tth.31.1070/1-736   --------------------------------------------
T_theileri_Tth.19.2140/1-590   --------------------------------------------
T_theileri_Tth.10.2870/1-312   --------------------------------------------
T_theileri_Tth.46.1010/1-442   --------------------------------------------
T_theileri_Tth.12.1820/1-197   --------------------------------------------
T_theileri_Tth.2.2110/1-566    --------------------------------------------
T_theileri_Tth.70.1050/1-869   --------------------------------------------
T_theileri_Tth.71.1110/1-97    --------------------------------------------
T_theileri_Tth.4.4270/1-365    --------------------------------------------
T_theileri_Tth.70.1120/1-459   --------------------------------------------
T_theileri_Tth.36.1940/1-208   --------------------------------------------
T_theileri_Tth.14.1390/1-407   --------------------------------------------
T_theileri_Tth.24.2340/1-572   --------------------------------------------
T_theileri_Tth.101.1060/1-233  --------------------------------------------
T_theileri_Tth.9.3510/1-513    --------------------------------------------
T_theileri_Tth.33.1860/1-659   --------------------------------------------
T_theileri_Tth.134.1010/1-723  --------------------------------------------
T_theileri_Tth.11.3620/1-114   --------------------------------------------
T_theileri_Tth.144.1000/1-229  --------------------------------------------
T_theileri_Tth.24.1780/1-518   --------------------------------------------
T_theileri_Tth.12.1030/1-678   --------------------------------------------
T_theileri_Tth.129.1010/1-165  --------------------------------------------
T_theileri_Tth.2.1510/1-887    SHWERRDAKDELMSVFIGDVSGMFYTTLTLSIFEDMKFYKANLS
T_theileri_Tth.31.1030/1-517   --------------------------------------------
T_theileri_Tth.36.2110/1-825   --------------------------------------------
T_theileri_Tth.151.1000/1-123  --------------------------------------------
T_theileri_Tth.136.1000/1-654  --------------------------------------------
T_theileri_Tth.14.1410/1-752   --------------------------------------------
T_theileri_Tth.46.1740/1-120   --------------------------------------------
T_theileri_Tth.31.1020/1-118   --------------------------------------------
T_theileri_Tth.124.1030/1-577  --------------------------------------------
```

```
                                         50        60        70        80
T_theileri_Tth.54.1380/1-425    --------------------------------------------
T_theileri_Tth.107.1000/1-244   --------------------------------------------
T_theileri_Tth.54.1340/1-680    --------------------------------------------
T_theileri_Tth.117.1040/1-195   --------------------------------------------
T_theileri_Tth.6.5060/1-554     --------------------------------------------
T_theileri_Tth.1.6360/1-511     --------------------------------------------
T_theileri_Tth.46.1110/1-516    --------------------------------------------
T_theileri_Tth.33.1800/1-520    --------------------------------------------
T_theileri_Tth.46.1020/1-842    --------------------------------------------
T_theileri_Tth.70.1140/1-882    --------------------------------------------
T_theileri_Tth.87.1050/1-130    --------------------------------------------
T_theileri_Tth.144.1010/1-374   --------------------------------------------
T_theileri_Tth.39.1000/1-654    --------------------------------------------
T_theileri_Tth.12.1010/1-802    --------------------------------------------
T_theileri_Tth.11.3500/1-528    --------------------------------------------
T_theileri_Tth.124.1020/1-215   --------------------------------------------
T_theileri_Tth.54.1390/1-443    --------------------------------------------
T_theileri_Tth.26.2480/1-314    --------------------------------------------
T_theileri_Tth.43.1970/1-318    --------------------------------------------
T_theileri_Tth.37.1300/1-102    --------------------------------------------
T_theileri_Tth.11.2410/1-509    --------------------------------------------
T_theileri_Tth.70.1150/1-914    --------------------------------------------
T_theileri_Tth.31.1050/1-313    --------------------------------------------
T_theileri_Tth.70.1010/1-670    --------------------------------------------
T_theileri_Tth.36.2090/1-806    --------------------------------------------
T_theileri_Tth.117.1050/1-116   --------------------------------------------
T_theileri_Tth.11.3480/1-173    --------------------------------------------
T_theileri_Tth.31.1070/1-736    --------------------------------------------
T_theileri_Tth.19.2140/1-590    --------------------------------------------
T_theileri_Tth.10.2870/1-312    --------------------------------------------
T_theileri_Tth.46.1010/1-442    --------------------------------------------
T_theileri_Tth.12.1820/1-197    --------------------------------------------
T_theileri_Tth.2.2110/1-566     --------------------------------------------
T_theileri_Tth.70.1050/1-869    --------------------------------------------
T_theileri_Tth.71.1110/1-97     --------------------------------------------
T_theileri_Tth.4.4270/1-365     --------------------------------------------
T_theileri_Tth.70.1120/1-459    --------------------------------------------
T_theileri_Tth.36.1940/1-208    --------------------------------------------
T_theileri_Tth.14.1390/1-407    --------------------------------------------
T_theileri_Tth.24.2340/1-572    --------------------------------------------
T_theileri_Tth.101.1060/1-233   --------------------------------------------
T_theileri_Tth.9.3510/1-513     --------------------------------------------
T_theileri_Tth.33.1860/1-659    --------------------------------------------
T_theileri_Tth.134.1010/1-723   --------------------------------------------
T_theileri_Tth.11.3620/1-114    -----------------------------MNILPLHNGLCFFAT
T_theileri_Tth.144.1000/1-229   --------------------------------------------
T_theileri_Tth.24.1780/1-518    --------------------------------------------
T_theileri_Tth.12.1030/1-678    --------------------------------------------
T_theileri_Tth.129.1010/1-165   --------------------------------------------
T_theileri_Tth.2.1510/1-887     MAEPMSWGKNAGCSFLTDKCVKKGVTEYPNMFCTESKTALQCTS
T_theileri_Tth.31.1030/1-517    --------------------------------------------
T_theileri_Tth.36.2110/1-825    --------------------------------------------
T_theileri_Tth.151.1000/1-123   --------------------------------------------
T_theileri_Tth.136.1000/1-654   --------------------------------------------
T_theileri_Tth.14.1410/1-752    --------------------------------------------
T_theileri_Tth.46.1740/1-120    --------------------------------------------
T_theileri_Tth.31.1020/1-118    --------------------------------------------
T_theileri_Tth.124.1030/1-577   --------------------------------------------
```

```
                                        90        100       110       120       130
T_theileri_Tth.54.1380/1-425    --------------------------------------------
T_theileri_Tth.107.1000/1-244   --------------------------------------------
T_theileri_Tth.54.1340/1-680    --------------------------------------------
T_theileri_Tth.117.1040/1-195   --------------------------------------------
T_theileri_Tth.6.5060/1-554     --------------------------------------------
T_theileri_Tth.1.6360/1-511     --------------------------------------------
T_theileri_Tth.46.1110/1-516    --------------------------------------------
T_theileri_Tth.33.1800/1-520    --------------------------------------------
T_theileri_Tth.46.1020/1-842    --------------------------------------------
T_theileri_Tth.70.1140/1-882    --------------------------------------------
T_theileri_Tth.87.1050/1-130    --------------------------------------------
T_theileri_Tth.144.1010/1-374   --------------------------------------------
T_theileri_Tth.39.1000/1-654    --------------------------------------------
T_theileri_Tth.12.1010/1-802    --------------------------------------------
T_theileri_Tth.11.3500/1-528    --------------------------------------------
T_theileri_Tth.124.1020/1-215   --------------------------------------------
T_theileri_Tth.54.1390/1-443    --------------------------------------------
T_theileri_Tth.26.2480/1-314    --------------------------------------------
T_theileri_Tth.43.1970/1-318    --------------------------------------------
T_theileri_Tth.37.1300/1-102    --------------------------------------------
T_theileri_Tth.11.2410/1-509    --------------------------------------------
T_theileri_Tth.70.1150/1-914    --------------------------------------------
T_theileri_Tth.31.1050/1-313    --------------------------------------------
T_theileri_Tth.70.1010/1-670    --------------------------------------------
T_theileri_Tth.36.2090/1-806    --------------------------------------------
T_theileri_Tth.117.1050/1-116   --------------------------------------------
T_theileri_Tth.11.3480/1-173    --------------------------------------------
T_theileri_Tth.31.1070/1-736    --------------------------------------------
T_theileri_Tth.19.2140/1-590    --------------------------------------------
T_theileri_Tth.10.2870/1-312    --------------------------------------------
T_theileri_Tth.46.1010/1-442    --------------------------------------------
T_theileri_Tth.12.1820/1-197    --------------------------------------------
T_theileri_Tth.2.2110/1-566     --------------------------------------------
T_theileri_Tth.70.1050/1-869    --------------------------------------------
T_theileri_Tth.71.1110/1-97     --------------------------------------------
T_theileri_Tth.4.4270/1-365     --------------------------------------------
T_theileri_Tth.70.1120/1-459    --------------------------------------------
T_theileri_Tth.36.1940/1-208    --------------------------------------------
T_theileri_Tth.14.1390/1-407    --------------------------------------------
T_theileri_Tth.24.2340/1-572    --------------------------------------------
T_theileri_Tth.101.1060/1-233   --------------------------------------------
T_theileri_Tth.9.3510/1-513     --------------------------------------------
T_theileri_Tth.33.1860/1-659    --------------------------------------------
T_theileri_Tth.134.1010/1-723   --------------------------------------------
T_theileri_Tth.11.3620/1-114    TVVPFDLDWHVRDCHCPALYCAPHSFFSLPHNPCLPLVSFSTLD
T_theileri_Tth.144.1000/1-229   --------------------------------------------
T_theileri_Tth.24.1780/1-518    --------------------------------------------
T_theileri_Tth.12.1030/1-678    --------------------------------------------
T_theileri_Tth.129.1010/1-165   --------------------------------------------
T_theileri_Tth.2.1510/1-887     DRFALGKCTVRTFDQRLPERYRFFEKPKVGAPSSELMNHCPAIK
T_theileri_Tth.31.1030/1-517    --------------------------------------------
T_theileri_Tth.36.2110/1-825    --------------------------------------------
T_theileri_Tth.151.1000/1-123   --------------------------------------------
T_theileri_Tth.136.1000/1-654   --------------------------------------------
T_theileri_Tth.14.1410/1-752    --------------------------------------------
T_theileri_Tth.46.1740/1-120    --------------------------------------------
T_theileri_Tth.31.1020/1-118    --------------------------------------------
T_theileri_Tth.124.1030/1-577   --------------------------------------------
```

```
                                       140       150       160       170
T_theileri_Tth.54.1380/1-425   --------------------------------------------
T_theileri_Tth.107.1000/1-244  ----------------EGMRRLFL----FAPL--LLLYCTLVT-
T_theileri_Tth.54.1340/1-680   --------------------------------------------
T_theileri_Tth.117.1040/1-195  --------------------------------------------
T_theileri_Tth.6.5060/1-554    --------------------------------------------
T_theileri_Tth.1.6360/1-511    ----------------MTRQMHVSLFLLLLLCLFVQLCSTSGIL
T_theileri_Tth.46.1110/1-516   -----------------------MRRLLCTAL--LLLCCAYGCI
T_theileri_Tth.33.1800/1-520   -----------------------MIRLLFHAVVLLFLFCTFTCL
T_theileri_Tth.46.1020/1-842   -----------------------MRRLLCTAL--LLLCCAYGC-
T_theileri_Tth.70.1140/1-882   -----------------------MRRLLCTAL--FLLCCAYGC-
T_theileri_Tth.87.1050/1-130   --------------------------------------------
T_theileri_Tth.144.1010/1-374  ------------------MEKHSMRHLLWAAL--FLLYCSCGC-
T_theileri_Tth.39.1000/1-654   ----------------------MMRHSLLHVVLLLFICGTVARP
T_theileri_Tth.12.1010/1-802   ------------------MEKHSMRHLLWTAL--FLLYCSCGC-
T_theileri_Tth.11.3500/1-528   --------------------------------------------
T_theileri_Tth.124.1020/1-215  --------------------------------------------
T_theileri_Tth.54.1390/1-443   --------------------------------------------
T_theileri_Tth.26.2480/1-314   --------------------------------------------
T_theileri_Tth.43.1970/1-318   ------------------MEKHSMRHLLWAAL--FLLYCSCGC-
T_theileri_Tth.37.1300/1-102   --------------------------------------------
T_theileri_Tth.11.2410/1-509   --------------------------------------------
T_theileri_Tth.70.1150/1-914   -----------------------MRRLLCTAL--LLLCCAYGC-
T_theileri_Tth.31.1050/1-313   --------------------------------------------
T_theileri_Tth.70.1010/1-670   -----------------------MRRLLCTAL--LLLCCAYGC-
T_theileri_Tth.36.2090/1-806   --------------------------------------------
T_theileri_Tth.117.1050/1-116  ---------MHNSYTRAVVGRYSPHPRVLTLLLLVLLCCTAGCL
T_theileri_Tth.11.3480/1-173   -----MRKIRNSYETTHSQIGKPLYITPLLLLLLFLCCASVCV
T_theileri_Tth.31.1070/1-736   --------------------------------------------
T_theileri_Tth.19.2140/1-590   --------------------MRHLLYVTVLLLVLLPLYCTNGLA
T_theileri_Tth.10.2870/1-312   --------------------------------------------
T_theileri_Tth.46.1010/1-442   --------------------------------------------
T_theileri_Tth.12.1820/1-197   --------------------------------------------
T_theileri_Tth.2.2110/1-566    -------------------------------MLFESVCVSFGFV
T_theileri_Tth.70.1050/1-869   -----------------VFKKIHMRCLLCTAL--LLLCCAYGC-
T_theileri_Tth.71.1110/1-97    --------------------------------------------
T_theileri_Tth.4.4270/1-365    ---------MHTPCARAVVGRYSSHPPVLTLLLLVLLCCTAGCL
T_theileri_Tth.70.1120/1-459   -----------------------MRRLLCTAL--FLLCCAYGCI
T_theileri_Tth.36.1940/1-208   --------------------------------------------
T_theileri_Tth.14.1390/1-407   --------------------------------------------
T_theileri_Tth.24.2340/1-572   ----MTKQANKLSARTPATVRQSLYVMP-LLLLLLFLCCACVCV
T_theileri_Tth.101.1060/1-233  ----MPQQVNKLSARTPATVCQSLYVMPLLLLLLLFLCCASVCV
T_theileri_Tth.9.3510/1-513    -----------------------MRE------------------
T_theileri_Tth.33.1860/1-659   ----------------MTRQMHVSLFLLLLLFLFVQLCSTSGIL
T_theileri_Tth.134.1010/1-723  ------------------MEKHSMRHLLWAAL--FLLYCSCGC-
T_theileri_Tth.11.3620/1-114   SLHYMPHQANKLSVRTPATVCQSLYVMP-LLLLLLFLCCASVCV
T_theileri_Tth.144.1000/1-229  --------------------------------------------
T_theileri_Tth.24.1780/1-518   -----MPQQANKLSARTPATVRQSLYVMPLLLLLLFLCCASVCV
T_theileri_Tth.12.1030/1-678   ------------------MEKHSMRHLLWTAL--FLLYCSFGC-
T_theileri_Tth.129.1010/1-165  --------------------------------------------
T_theileri_Tth.2.1510/1-887    PLASTSCEDGDSTQMPGSLLGPRSRCLKGESLELKEPSAENKPM
T_theileri_Tth.31.1030/1-517   -----------------------MRCLLCTAL--LLLCCAYGC-
T_theileri_Tth.36.2110/1-825   ------------------MRRLLL----FAPL--LLLYCTLVT-
T_theileri_Tth.151.1000/1-123  ------------------------------------------CV
T_theileri_Tth.136.1000/1-654  ----------------------MMRHSLLQVVLLLFICGAMAGP
T_theileri_Tth.14.1410/1-752   ---------MHTPCARAVVCRYSPHPRVLTLLLLLLLLCCTAGCL
T_theileri_Tth.46.1740/1-120   --------------------------------------------
T_theileri_Tth.31.1020/1-118   --------------------------------------------
T_theileri_Tth.124.1030/1-577  --------------------------------------------
```

```
                                        180       190       200       210
T_theileri_Tth.54.1380/1-425   --------------------------------------------
T_theileri_Tth.107.1000/1-244  L-------------------------------------------
T_theileri_Tth.54.1340/1-680   --------------------------------------------
T_theileri_Tth.117.1040/1-195  --------------------------------------------
T_theileri_Tth.6.5060/1-554    --------------------------------------------
T_theileri_Tth.1.6360/1-511    A-------------------------------------------
T_theileri_Tth.46.1110/1-516   --------------------------------------------
T_theileri_Tth.33.1800/1-520   A-------------------------------------------
T_theileri_Tth.46.1020/1-842   I-------------------------------------------
T_theileri_Tth.70.1140/1-882   I-------------------------------------------
T_theileri_Tth.87.1050/1-130   --------------------------------------------
T_theileri_Tth.144.1010/1-374  L-------------------------------------------
T_theileri_Tth.39.1000/1-654   A-------------------------------------------
T_theileri_Tth.12.1010/1-802   L-------------------------------------------
T_theileri_Tth.11.3500/1-528   --------------------------------------------
T_theileri_Tth.124.1020/1-215  --------------------------------------------
T_theileri_Tth.54.1390/1-443   --------------------------------------------
T_theileri_Tth.26.2480/1-314   --------------------------------------------
T_theileri_Tth.43.1970/1-318   L-------------------------------------------
T_theileri_Tth.37.1300/1-102   --------------------------------------------
T_theileri_Tth.11.2410/1-509   --------------------------------------------
T_theileri_Tth.70.1150/1-914   I-------------------------------------------
T_theileri_Tth.31.1050/1-313   --------------------------------------------
T_theileri_Tth.70.1010/1-670   I-------------------------------------------
T_theileri_Tth.36.2090/1-806   --------------------------------------------
T_theileri_Tth.117.1050/1-116  A-------------------------------------------
T_theileri_Tth.11.3480/1-173   A-------------------------------------------
T_theileri_Tth.31.1070/1-736   --------------------------------------------
T_theileri_Tth.19.2140/1-590   A-------------------------------------------
T_theileri_Tth.10.2870/1-312   --------------------------------------------
T_theileri_Tth.46.1010/1-442   --------------------------------------------
T_theileri_Tth.12.1820/1-197   --------------------------------------------
T_theileri_Tth.2.2110/1-566    E-------------------------------------------
T_theileri_Tth.70.1050/1-869   I-------------------------------------------
T_theileri_Tth.71.1110/1-97    --------------------------------------------
T_theileri_Tth.4.4270/1-365    A-------------------------------------------
T_theileri_Tth.70.1120/1-459   --------------------------------------------
T_theileri_Tth.36.1940/1-208   --------------------------------------------
T_theileri_Tth.14.1390/1-407   --------------------------------------------
T_theileri_Tth.24.2340/1-572   A-------------------------------------------
T_theileri_Tth.101.1060/1-233  A-------------------------------------------
T_theileri_Tth.9.3510/1-513    --------------------------------------------
T_theileri_Tth.33.1860/1-659   A-------------------------------------------
T_theileri_Tth.134.1010/1-723  L-------------------------------------------
T_theileri_Tth.11.3620/1-114   A-------------------------------------------
T_theileri_Tth.144.1000/1-229  --------------------------------------------
T_theileri_Tth.24.1780/1-518   A-------------------------------------------
T_theileri_Tth.12.1030/1-678   L-------------------------------------------
T_theileri_Tth.129.1010/1-165  --------------------------------------------
T_theileri_Tth.2.1510/1-887    GDICANVRCGENNTVMVQYKGDDNWYECPDGENIKPSEKSVFSK
T_theileri_Tth.31.1030/1-517   I-------------------------------------------
T_theileri_Tth.36.2110/1-825   L-------------------------------------------
T_theileri_Tth.151.1000/1-123  A-------------------------------------------
T_theileri_Tth.136.1000/1-654  A-------------------------------------------
T_theileri_Tth.14.1410/1-752   A-------------------------------------------
T_theileri_Tth.46.1740/1-120   --------------------------------------------
T_theileri_Tth.31.1020/1-118   --------------------------------------------
T_theileri_Tth.124.1030/1-577  --------------------------------------------
```

```
                                          230       240       250       260
T_theileri_Tth.54.1380/1-425    --------------------------------------------
T_theileri_Tth.107.1000/1-244   --------------------------------------------
T_theileri_Tth.54.1340/1-680    --------------------------------------------
T_theileri_Tth.117.1040/1-195   --------------------------------------------
T_theileri_Tth.6.5060/1-554     --------------------------------------------
T_theileri_Tth.1.6360/1-511     --------------------------------------------
T_theileri_Tth.46.1110/1-516    --------------------------------------------
T_theileri_Tth.33.1800/1-520    --------------------------------------------
T_theileri_Tth.46.1020/1-842    --------------------------------------------
T_theileri_Tth.70.1140/1-882    --------------------------------------------
T_theileri_Tth.87.1050/1-130    --------------------------------------------
T_theileri_Tth.144.1010/1-374   --------------------------------------------
T_theileri_Tth.39.1000/1-654    --------------------------------------------
T_theileri_Tth.12.1010/1-802    --------------------------------------------
T_theileri_Tth.11.3500/1-528    --------------------------------------------
T_theileri_Tth.124.1020/1-215   --------------------------------------------
T_theileri_Tth.54.1390/1-443    --------------------------------------------
T_theileri_Tth.26.2480/1-314    --------------------------------------------
T_theileri_Tth.43.1970/1-318    --------------------------------------------
T_theileri_Tth.37.1300/1-102    --------------------------------------------
T_theileri_Tth.11.2410/1-509    --------------------------------------------
T_theileri_Tth.70.1150/1-914    --------------------------------------------
T_theileri_Tth.31.1050/1-313    --------------------------------------------
T_theileri_Tth.70.1010/1-670    --------------------------------------------
T_theileri_Tth.36.2090/1-806    --------------------------------------------
T_theileri_Tth.117.1050/1-116   --------------------------------------------
T_theileri_Tth.11.3480/1-173    --------------------------------------------
T_theileri_Tth.31.1070/1-736    --------------------------------------------
T_theileri_Tth.19.2140/1-590    --------------------------------------------
T_theileri_Tth.10.2870/1-312    --------------------------------------------
T_theileri_Tth.46.1010/1-442    --------------------------------------------
T_theileri_Tth.12.1820/1-197    --------------------------------------------
T_theileri_Tth.2.2110/1-566     --------------------------------------------
T_theileri_Tth.70.1050/1-869    --------------------------------------------
T_theileri_Tth.71.1110/1-97     --------------------------------------------
T_theileri_Tth.4.4270/1-365     --------------------------------------------
T_theileri_Tth.70.1120/1-459    --------------------------------------------
T_theileri_Tth.36.1940/1-208    --------------------------------------------
T_theileri_Tth.14.1390/1-407    --------------------------------------------
T_theileri_Tth.24.2340/1-572    --------------------------------------------
T_theileri_Tth.101.1060/1-233   --------------------------------------------
T_theileri_Tth.9.3510/1-513     --------------------------------------------
T_theileri_Tth.33.1860/1-659    --------------------------------------------
T_theileri_Tth.134.1010/1-723   --------------------------------------------
T_theileri_Tth.11.3620/1-114    --------------------------------------------
T_theileri_Tth.144.1000/1-229   --------------------------------------------
T_theileri_Tth.24.1780/1-518    --------------------------------------------
T_theileri_Tth.12.1030/1-678    --------------------------------------------
T_theileri_Tth.129.1010/1-165   --------------------------------------------
T_theileri_Tth.2.1510/1-887     GMIVCPKYHEVCTGLPEITPDDAVENKETEKSEDGHESVGSAEQ
T_theileri_Tth.31.1030/1-517    --------------------------------------------
T_theileri_Tth.36.2110/1-825    --------------------------------------------
T_theileri_Tth.151.1000/1-123   --------------------------------------------
T_theileri_Tth.136.1000/1-654   --------------------------------------------
T_theileri_Tth.14.1410/1-752    --------------------------------------------
T_theileri_Tth.46.1740/1-120    --------------------------------------------
T_theileri_Tth.31.1020/1-118    --------------------------------------------
T_theileri_Tth.124.1030/1-577   --------------------------------------------
```

```
                                      270       280       290       300
T_theileri_Tth.54.1380/1-425   ----------------------------------MHDTVAMRVG
T_theileri_Tth.107.1000/1-244  ----------------------AVTEH----HRCISDTVALRAG
T_theileri_Tth.54.1340/1-680   --------------------------------------------
T_theileri_Tth.117.1040/1-195  --------------------------------------------
T_theileri_Tth.6.5060/1-554    --------------------------------------------
T_theileri_Tth.1.6360/1-511    -------------------APSAGVVQ----EVPLK--------
T_theileri_Tth.46.1110/1-516   ----------------------AAVVQ----PLPKR--------
T_theileri_Tth.33.1800/1-520   ----AIHSRCSFDDIMRTGGNPVSVVR----ELPSK--------
T_theileri_Tth.46.1020/1-842   ----------------------AAVVQ----PLPQK--------
T_theileri_Tth.70.1140/1-882   ----------------------AAVVQ----PLPQR--------
T_theileri_Tth.87.1050/1-130   --------------------------------------------
T_theileri_Tth.144.1010/1-374  ----------------------AAVVQ----QLPQK--------
T_theileri_Tth.39.1000/1-654   -------YACNYDEIKEKSGPPVVVVR----ELPKK--------
T_theileri_Tth.12.1010/1-802   ----------------------ATVVQ----QLPQK--------
T_theileri_Tth.11.3500/1-528   --------------------------------------------
T_theileri_Tth.124.1020/1-215  --------------------------------------------
T_theileri_Tth.54.1390/1-443   --------------------------------------------
T_theileri_Tth.26.2480/1-314   --------------------------------------------
T_theileri_Tth.43.1970/1-318   ----------------------AAVVQ----QLPQK--------
T_theileri_Tth.37.1300/1-102   --------------------------------------------
T_theileri_Tth.11.2410/1-509   --------------------------------------------
T_theileri_Tth.70.1150/1-914   ----------------------AAVVQ----PLPKR--------
T_theileri_Tth.31.1050/1-313   --------------------------------------------
T_theileri_Tth.70.1010/1-670   ----------------------AAVVQ----PLPQK--------
T_theileri_Tth.36.2090/1-806   ----------------------------------MSDFVALRVG
T_theileri_Tth.117.1050/1-116  -----ASHHRCMHDQMAQGSEQMAVVR----EIPRK--------
T_theileri_Tth.11.3480/1-173   QKYG--------------GVQSTGVVR----ELPRK--------
T_theileri_Tth.31.1070/1-736   --------------------------------------------
T_theileri_Tth.19.2140/1-590   ---TGGRSCGFDAVRRRRGNTPVAVVR----ELPVK--------
T_theileri_Tth.10.2870/1-312   --------------------------------------------
T_theileri_Tth.46.1010/1-442   ---------------------------------PQK--------
T_theileri_Tth.12.1820/1-197   --------------------------------------------
T_theileri_Tth.2.2110/1-566    --------HRCTFDQRVKHGEPLPVVR----ELPRK--------
T_theileri_Tth.70.1050/1-869   ----------------------AAVVQ----PLPQR--------
T_theileri_Tth.71.1110/1-97    --------------------------------------------
T_theileri_Tth.4.4270/1-365    -----TIPHHSMYGETSQKNRPTGVVR----EIPRR--------
T_theileri_Tth.70.1120/1-459   ----------------------AAVVQ----PLPQT--------
T_theileri_Tth.36.1940/1-208   --------------------------------------------
T_theileri_Tth.14.1390/1-407   --------------------------------------------
T_theileri_Tth.24.2340/1-572   QKDG--------------GVQSTGVVR----ELPRK--------
T_theileri_Tth.101.1060/1-233  -WDDGMQRCMFEEMINRSGMFSTGVVR----ELPRK--------
T_theileri_Tth.9.3510/1-513    --------------------------------------------
T_theileri_Tth.33.1860/1-659   -------------------ESLTGVVL----QVPRN--------
T_theileri_Tth.134.1010/1-723  ----------------------AAVVQ----QLPQK--------
T_theileri_Tth.11.3620/1-114   QKDG--------------GVQSTGV-------------------
T_theileri_Tth.144.1000/1-229  -----------MMPCVRAVPPAKVGVR----ELLSA--------
T_theileri_Tth.24.1780/1-518   --------------QKDGGVQSTGVVR----ELPRK--------
T_theileri_Tth.12.1030/1-678   ----------------------AAVVQ----QLPQK--------
T_theileri_Tth.129.1010/1-165  --------------------------------------------
T_theileri_Tth.2.1510/1-887    GEDSNDAGIFENSGDDEEEATSSSVVR----ELPTK--------
T_theileri_Tth.31.1030/1-517   ----------------------AAVVQ----PLPQK--------
T_theileri_Tth.36.2110/1-825   ----------------------AVTE-----HRCIHDRVMMHVG
T_theileri_Tth.151.1000/1-123  QKDG--------------GVQSTGVVR----ELPRK--------
T_theileri_Tth.136.1000/1-654  -------YACNYDEIKEKSGPPVVVVR----ELPKK--------
T_theileri_Tth.14.1410/1-752   -----ASHHRCMHDKISELNKPVTVVR----EIPRK--------
T_theileri_Tth.46.1740/1-120   --------------------------------------------
T_theileri_Tth.31.1020/1-118   --------------------------------------------
T_theileri_Tth.124.1030/1-577  --------------------------------------------
```

```
                                   310       320       330       340       350
T_theileri_Tth.54.1380/1-425   VGSTVESDEEITKK---REAQQ-TLFSS---------NNQPTPA
T_theileri_Tth.107.1000/1-244  PTMSMDIFMKITEK---RQAQI-TRF-A---------QDQPLPA
T_theileri_Tth.54.1340/1-680   --------------------------------------------
T_theileri_Tth.117.1040/1-195  --------------------------------------------
T_theileri_Tth.6.5060/1-554    --------------------------------------------
T_theileri_Tth.1.6360/1-511    -----------------GQSGV-QAYTVST--------------
T_theileri_Tth.46.1110/1-516   -----------------GQGPW-DTYTLSV-------PGV--DT
T_theileri_Tth.33.1800/1-520   -----------------GQGAW-EVYTAAT--------------
T_theileri_Tth.46.1020/1-842   -----------------GQGPW-DTYIVSA------------PK
T_theileri_Tth.70.1140/1-882   -----------------GQGPW-DTYTLSV--------------
T_theileri_Tth.87.1050/1-130   --------------------------------------------
T_theileri_Tth.144.1010/1-374  -----------------GQSAL-QAYTVSS-----------DTA
T_theileri_Tth.39.1000/1-654   -----------------GEGTW-QAYTVST--------------
T_theileri_Tth.12.1010/1-802   -----------------GQSGL-QAYTVST------------AT
T_theileri_Tth.11.3500/1-528   --------------------------------------------
T_theileri_Tth.124.1020/1-215  --------------------------------------------
T_theileri_Tth.54.1390/1-443   --------------------------------------------
T_theileri_Tth.26.2480/1-314   --------------------------------------------
T_theileri_Tth.43.1970/1-318   -----------------GESAL-QAYTVSV-------------P
T_theileri_Tth.37.1300/1-102   --------------------------------------------
T_theileri_Tth.11.2410/1-509   -----------------------QAYTVAT--------------
T_theileri_Tth.70.1150/1-914   -----------------GQGPW-DTYTLSV-----------PGG
T_theileri_Tth.31.1050/1-313   --------------------------------------------
T_theileri_Tth.70.1010/1-670   -----------------GQGPW-DTYTVAE----------VGSV
T_theileri_Tth.36.2090/1-806   SPTTPQFYEEIMTK---REAQK-KRFSS---------RDRPPSA
T_theileri_Tth.117.1050/1-116  -----------------GQGAV-QAYAASA--------------
T_theileri_Tth.11.3480/1-173   -----------------GQSGV-QAYTVAT--------------
T_theileri_Tth.31.1070/1-736   --------------------------------------------
T_theileri_Tth.19.2140/1-590   -----------------GQGPW-QAYTAAT--------------
T_theileri_Tth.10.2870/1-312   --------------------------------------------
T_theileri_Tth.46.1010/1-442   -----------------GQGPW-DTYTLSV--------------
T_theileri_Tth.12.1820/1-197   --------------------------------------------
T_theileri_Tth.2.2110/1-566    -----------------GRNGL-QAYIAAN--------------
T_theileri_Tth.70.1050/1-869   -----------------GQGPW-DTYTVAE------------VG
T_theileri_Tth.71.1110/1-97    --------------------------------------------
T_theileri_Tth.4.4270/1-365    -----------------GQGAV-QAYTTSA------------AI
T_theileri_Tth.70.1120/1-459   -----------------GQGPW-DTYTVAV-----------TDD
T_theileri_Tth.36.1940/1-208   --------------------------------------------
T_theileri_Tth.14.1390/1-407   --------------------------------------------
T_theileri_Tth.24.2340/1-572   -----------------GQSGV-QAYTVSA--------------
T_theileri_Tth.101.1060/1-233  -----------------GQSGV-QAYTVAT--------------
T_theileri_Tth.9.3510/1-513    --------------------------------------------
T_theileri_Tth.33.1860/1-659   -----------------GDDAS-QAYTVAT--------------
T_theileri_Tth.134.1010/1-723  -----------------GQSAL-QAYTVS---------A-G---
T_theileri_Tth.11.3620/1-114   --------------------------------------------
T_theileri_Tth.144.1000/1-229  -----------------GDRASHQINIVSS--------------
T_theileri_Tth.24.1780/1-518   -----------------GQSGV-QAYTVAT--------------
T_theileri_Tth.12.1030/1-678   -----------------GESAL-QAYTVSD--------------
T_theileri_Tth.129.1010/1-165  --------------------------------------------
T_theileri_Tth.2.1510/1-887    -----------------GDELA-EKYAIVT--------------
T_theileri_Tth.31.1030/1-517   -----------------GQGPW-DAYVVSA------------PK
T_theileri_Tth.36.2110/1-825   ADFNGDDRLLGMTTVSRHRD---SNANS---------RSSPTLK
T_theileri_Tth.151.1000/1-123  -----------------GQSGV-QAYTVAT--------------
T_theileri_Tth.136.1000/1-654  -----------------GEGAW-QAYTVST--------------
T_theileri_Tth.14.1410/1-752   -----------------GQGAV-QAYTANA------------AE
T_theileri_Tth.46.1740/1-120   --------------------------------------------
T_theileri_Tth.31.1020/1-118   --------------------------------------------
T_theileri_Tth.124.1030/1-577  --------------------------------------------
```

```
                                              360       370       380       390
T_theileri_Tth.54.1380/1-425    GR-EEPVGE----GWGPIRIKLVTDNLEK---------------
T_theileri_Tth.107.1000/1-244   VR-EEQVGE----GWKPIRIKLVTGTLKR---------------
T_theileri_Tth.54.1340/1-680    --------------------------------------------
T_theileri_Tth.117.1040/1-195   --------------------------------------------
T_theileri_Tth.6.5060/1-554     --------------------------------------------
T_theileri_Tth.1.6360/1-511     ------GYR------HLIRIVTSTKDLEN---------------
T_theileri_Tth.46.1110/1-516    DV-PEKVEE----NWQPIRFAVSSHGVAW---------------
T_theileri_Tth.33.1800/1-520    -----------SESWKPIRIIASREDLED---------------
T_theileri_Tth.46.1020/1-842    GS-IDKSGK----NWQPIRFAVSAKEIDR---------------
T_theileri_Tth.70.1140/1-882    -T-DSEKKK----DFKPIRFAVSAKEIDR---------------
T_theileri_Tth.87.1050/1-130    --------------------------------------------
T_theileri_Tth.144.1010/1-374   AD-DTKIGS----DWKAIRIGVYTKLVED---------------
T_theileri_Tth.39.1000/1-654    SE-DSNGNK----GWEALRIRVSIEDLKK---------------
T_theileri_Tth.12.1010/1-802    ---DGKEG-----DWQPIRIAVYTKRVED---------------
T_theileri_Tth.11.3500/1-528    --------KNENERWELIRIEAFTKDLEN---------------
T_theileri_Tth.124.1020/1-215   --------------------------------------------
T_theileri_Tth.54.1390/1-443    --------------------------------------------
T_theileri_Tth.26.2480/1-314    --------------------------------------------
T_theileri_Tth.43.1970/1-318    AS-DDKNGE----DWKPIRIGVYTRLVEE---------------
T_theileri_Tth.37.1300/1-102    --------------------------------------------
T_theileri_Tth.11.2410/1-509    ------SKR------QLIRIKTSMEDLKD---------------
T_theileri_Tth.70.1150/1-914    VD-LERKKE----GFQPIRFAVSAKEIDR---------------
T_theileri_Tth.31.1050/1-313    --------------------------------------------
T_theileri_Tth.70.1010/1-670    PD-HDSGKN----NFEPIRFAVSAKEIDR---------------
T_theileri_Tth.36.2090/1-806    VR-EEP-QE----EWKAIRIKLVSDHLKK---------------
T_theileri_Tth.117.1050/1-116   --------------------------------------------
T_theileri_Tth.11.3480/1-173    ---------QENGGWELIRIEAFTRDLED---------------
T_theileri_Tth.31.1070/1-736    --------------------------------------------
T_theileri_Tth.19.2140/1-590    -----------YSPWAPIRILVSTIDLED---------------
T_theileri_Tth.10.2870/1-312    --------------------------------------------
T_theileri_Tth.46.1010/1-442    PD-HDSGKD----NFQPIRFAVSAKEIDR---------------
T_theileri_Tth.12.1820/1-197    --------------------------------------------
T_theileri_Tth.2.2110/1-566     --------Q----GWERMRFGVSIEDLKD---------------
T_theileri_Tth.70.1050/1-869    SD-HVSGKD----NFQPIRFAVSAKEFDR---------------
T_theileri_Tth.71.1110/1-97     --------------------------------------------
T_theileri_Tth.4.4270/1-365     DSSDSKDNK----NFEPIRIKAYTRDLND---------------
T_theileri_Tth.70.1120/1-459    GL-PKKGQE----NWQPIRIAVSSHGVAW---------------
T_theileri_Tth.36.1940/1-208    --------------------------------------------
T_theileri_Tth.14.1390/1-407    --------------------------------------------
T_theileri_Tth.24.2340/1-572    -------QEKDDKEWKLIRIKASTKDLED---------------
T_theileri_Tth.101.1060/1-233   -------QKNENERWELIRIEAFTKDLED---------------
T_theileri_Tth.9.3510/1-513     --------------------------------------------
T_theileri_Tth.33.1860/1-659    ------ENR------KFIRIHASYEDLTK---------------
T_theileri_Tth.134.1010/1-723   -----DNNE----KWEPIRFAVYTKYVED---------------
T_theileri_Tth.11.3620/1-114    --------------------------------------------
T_theileri_Tth.144.1000/1-229   -------SD----KWEPLRINVSSLDLDD---------------
T_theileri_Tth.24.1780/1-518    ------EK---NEGWELIRIEAFTKDLEN---------------
T_theileri_Tth.12.1030/1-678    -----TPTE----KWEPIRIAVYTKYVED---------------
T_theileri_Tth.129.1010/1-165   --------------------------------------------
T_theileri_Tth.2.1510/1-887     -----REGQ----DWQPIRIHVSAEAVDA---------------
T_theileri_Tth.31.1030/1-517    TVDDVPEKK----DFQPIRFAVSAKEVDR---------------
T_theileri_Tth.36.2110/1-825    IR-EDP-SE----GWKPIRIKLVTDDLKK---------------
T_theileri_Tth.151.1000/1-123   ------QEKNENGGWKLIRIEAFTRDLED---------------
T_theileri_Tth.136.1000/1-654   SN-KENSNE----GWEPLRIKVSYEDLKS---------------
T_theileri_Tth.14.1410/1-752    NSNIFGDNK----NWVPIRIKAFTRDLND---------------
T_theileri_Tth.46.1740/1-120    --------------------------------------------
T_theileri_Tth.31.1020/1-118    --------------------------------------------
T_theileri_Tth.124.1030/1-577   --------------------------------------------
```

```
                                         400       410       420       430
T_theileri_Tth.54.1380/1-425   -----AGHDCTGK-ETNRITTLL---------------------
T_theileri_Tth.107.1000/1-244  -----DGHYCGEKGNKNPIITLL---------------------
T_theileri_Tth.54.1340/1-680   --------------------------------------------
T_theileri_Tth.117.1040/1-195  --------------------------------------------
T_theileri_Tth.6.5060/1-554    --------------------------------------------
T_theileri_Tth.1.6360/1-511    -----KRKYCSAEGMPIWDFDG----------------------
T_theileri_Tth.46.1110/1-516   -----TVNHCLYRKVGARWSYGYNNPH-----------------
T_theileri_Tth.33.1800/1-520   -----STKYCTADGEERVDYSS----------------------
T_theileri_Tth.46.1020/1-842   -----VLKYCEQWKKDPWAK--------DIYDEDYGFILEVAK-
T_theileri_Tth.70.1140/1-882   -----VLKYCKQSDVDLDEDQEEEYDAEYDDTKEYGFPLDPEE-
T_theileri_Tth.87.1050/1-130   --------------------------------------------
T_theileri_Tth.144.1010/1-374  -----MVEFCTTKKKEMPREYEELARKYEEEEEDADEEHEEPAQ
T_theileri_Tth.39.1000/1-654   ------ENYCASTGETRKDFLD----------------------
T_theileri_Tth.12.1010/1-802   -----VLNFCGKNK--LPPEYYEEFLDPQELEE-----------
T_theileri_Tth.11.3500/1-528   -----ESRYCTKVDDEVMDFKG----------------------
T_theileri_Tth.124.1020/1-215  --------------------------------------------
T_theileri_Tth.54.1390/1-443   --------------------------------------------
T_theileri_Tth.26.2480/1-314   --------------------------------------------
T_theileri_Tth.43.1970/1-318   -----IISYCERTKHLDDEDEEEEFG------------------
T_theileri_Tth.37.1300/1-102   --------------------------------------------
T_theileri_Tth.11.2410/1-509   -----EKKYCTTENAKLRTSDG----------------------
T_theileri_Tth.70.1150/1-914   -----IVKNCEHNGGEWDVE--------YDDTKDYGFKLNIKE-
T_theileri_Tth.31.1050/1-313   --------------------------------------------
T_theileri_Tth.70.1010/1-670   -----VLAYCKDFKGVLDEDQEEDKYEPKKDEEEYGFPLDPEE-
T_theileri_Tth.36.2090/1-806   ------GQYCSGGVTQ--INTTL---------------------
T_theileri_Tth.117.1050/1-116  --------------------------------------------
T_theileri_Tth.11.3480/1-173   -----TSKYCTKAGQEVIDFNG----------------------
T_theileri_Tth.31.1070/1-736   --------------------------------------------
T_theileri_Tth.19.2140/1-590   -----DSRHCTAFGQLRPDFEF----------------------
T_theileri_Tth.10.2870/1-312   --------------------------------------------
T_theileri_Tth.46.1010/1-442   -----VLKYCDEYDYKEAAE--------KEFDEEYGFTLSIRE-
T_theileri_Tth.12.1820/1-197   --------------------------------------------
T_theileri_Tth.2.2110/1-566    -----DFRYCTRAGQRRP-DFE----------------------
T_theileri_Tth.70.1050/1-869   -----VMKYCKHLNGDLDEDQEGDKYDPKKDKEEYGFEIGVKE-
T_theileri_Tth.71.1110/1-97    --------------------------------------------
T_theileri_Tth.4.4270/1-365    -----PSKYCTRAGDYCDTPSY----------------------
T_theileri_Tth.70.1120/1-459   -----TVNHCLYKKVGARWSYGYNNPH-----------------
T_theileri_Tth.36.1940/1-208   --------------------------------------------
T_theileri_Tth.14.1390/1-407   --------------------------------------------
T_theileri_Tth.24.2340/1-572   -----ASRYCTKIGEQIIDFEG----------------------
T_theileri_Tth.101.1060/1-233  -----KSRYCTTSGVQVMNFKG----------------------
T_theileri_Tth.9.3510/1-513    --------------------------------------------
T_theileri_Tth.33.1860/1-659   ------GKYCSKVGEMIQNFQG----------------------
T_theileri_Tth.134.1010/1-723  -----VMNYCIAEKKVMPSEYNVLPGVRDDLEG-----------
T_theileri_Tth.11.3620/1-114   --------------------------------------------
T_theileri_Tth.144.1000/1-229  -----EKKYCRGHLDEHKSYFK----------------------
T_theileri_Tth.24.1780/1-518   -----PSEYCRKVKENNVNG------------------------
T_theileri_Tth.12.1030/1-678   -----VVKECIKQRSEGDSNTTV---------------------
T_theileri_Tth.129.1010/1-165  --------------------------------------------
T_theileri_Tth.2.1510/1-887    -----AVEKCKDWAAKATEKNIRGDTFDPNYVELVYPE------
T_theileri_Tth.31.1030/1-517   -----VLKYCKQWKKDPSAK--------DVYDNEYGFTLEVAN-
T_theileri_Tth.36.2110/1-825   -----GEHYCLDDAKK--SEILL---------------------
T_theileri_Tth.151.1000/1-123  -----ESKYCTKAGQEVIDFDG----------------------
T_theileri_Tth.136.1000/1-654  ------DKYCVKEKEKRMDFLN----------------------
T_theileri_Tth.14.1410/1-752   -----PSKYCTVAGEERYDQTY----------------------
T_theileri_Tth.46.1740/1-120   --------------------------------------------
T_theileri_Tth.31.1020/1-118   --------------------------------------------
T_theileri_Tth.124.1030/1-577  -----HSLLCSKVNETKH-EEKVKRENRFGEDDGRAEHTKVTVE
```

```
                                           450       460       470       480
T_theileri_Tth.54.1380/1-425   -----------K---------EYFTCT---ENDVLTEEKKKILV
T_theileri_Tth.107.1000/1-244  -----------N---------KEYTCT---DDDVLTPEKEHILV
T_theileri_Tth.54.1340/1-680   --------------------------------------KKKILV
T_theileri_Tth.117.1040/1-195  --------------------------------------------
T_theileri_Tth.6.5060/1-554    ----------------------------------MTTKRMHTLF
T_theileri_Tth.1.6360/1-511    ---------------------NVIKC---HNYHVLKTEDVQYLK
T_theileri_Tth.46.1110/1-516   -----------F---------DKVVCY---RRDGITVEKANLFL
T_theileri_Tth.33.1800/1-520   -----------G---------LALQC---ASFDILTTAKKNLII
T_theileri_Tth.46.1020/1-842   -----------H---------KDEFCY-EEEKTKMTDVKKSTLL
T_theileri_Tth.70.1140/1-882   ---------------------INEFCE---GQNEVKLYVRKTLL
T_theileri_Tth.87.1050/1-130   --------------------------------------------
T_theileri_Tth.144.1010/1-374  ELY--------D---------LKDLCN----ADKITEDKKKVLF
T_theileri_Tth.39.1000/1-654   -----------G---------QNAPC---TPNDFMMPGKEKAIK
T_theileri_Tth.12.1010/1-802   -----------E---------FEELCGDDGRKKMMIKEKKDILL
T_theileri_Tth.11.3500/1-528   ---------------------GTSKC---YETEILNDKKKETLI
T_theileri_Tth.124.1020/1-215  --------------------------------------------
T_theileri_Tth.54.1390/1-443   --------------------------------------------
T_theileri_Tth.26.2480/1-314   --------------------------------------------
T_theileri_Tth.43.1970/1-318   -----------V---------FNDFCE-RDDHKKMTTQIKDTLF
T_theileri_Tth.37.1300/1-102   --------------------------------------------
T_theileri_Tth.11.2410/1-509   ---------------------RELSC---DKDYILTEEQKTNLM
T_theileri_Tth.70.1150/1-914   -----------I---------NTNFCD---GPNEVTAYRRKTLL
T_theileri_Tth.31.1050/1-313   --------------------------------------------
T_theileri_Tth.70.1010/1-670   ---------------------ISEFCE---GQNEVKQYMRKTLL
T_theileri_Tth.36.2090/1-806   -----------K---------NNITCT---DHDVLTPEKEEILV
T_theileri_Tth.117.1050/1-116  --------------------------------------------
T_theileri_Tth.11.3480/1-173   ---------------------NKAKC---KEAEVLNDERHREYI
T_theileri_Tth.31.1070/1-736   ----------------------------------MTDVKKKTLL
T_theileri_Tth.19.2140/1-590   -----------G---------QKV-C---LADHVLTNEKRNALI
T_theileri_Tth.10.2870/1-312   --------------------------------------------
T_theileri_Tth.46.1010/1-442   -----------H---------REDFCE--EPKTKMTDVKKKTLL
T_theileri_Tth.12.1820/1-197   --------------------------------------------
T_theileri_Tth.2.2110/1-566    -----------G---------GTLVC---TEADILTTEKKILLV
T_theileri_Tth.70.1050/1-869   -----------I---------NDDFCD-EEGHNTVTPGKKRTLL
T_theileri_Tth.71.1110/1-97    --------------------------------------------
T_theileri_Tth.4.4270/1-365    ---------------------NRQYC---HHSSVLTVRKRKILV
T_theileri_Tth.70.1120/1-459   -----------F---------DKVVCY---RRDGITVEKANLFL
T_theileri_Tth.36.1940/1-208   --------------------------------------------
T_theileri_Tth.14.1390/1-407   --------------------------------------------
T_theileri_Tth.24.2340/1-572   ---------------------DEVVC---DEEDILTDEKKKTII
T_theileri_Tth.101.1060/1-233  ---------------------GTAIC---NDDNVLTDEKKETLI
T_theileri_Tth.9.3510/1-513    --------------------------------------------
T_theileri_Tth.33.1860/1-659   ---------------------EDVKC---EDGNTFTDEMISKYI
T_theileri_Tth.134.1010/1-723  -----------Q---------FKQLCD-GEN--KMTTKRMHILF
T_theileri_Tth.11.3620/1-114   --------------------------------------------
T_theileri_Tth.144.1000/1-229  -----------G---------SKLSCR---------EDKKNTLL
T_theileri_Tth.24.1780/1-518   ---------------------GRMEC----STSFITAEQKKDIE
T_theileri_Tth.12.1030/1-678   -----------------------EFCS-GDN--QMTAAKMNTLF
T_theileri_Tth.129.1010/1-165  --------------------------------------------
T_theileri_Tth.2.1510/1-887    ---------------------ATEHCS---EETLITADKKKTLV
T_theileri_Tth.31.1030/1-517   -----------S---------RFHFCK---EESKMTAAKKRILL
T_theileri_Tth.36.2110/1-825   -----------H---------HTYNCS---KDDELTPEKKKILV
T_theileri_Tth.151.1000/1-123  ---------------------KKAKC---EEAEVLNDERHREYI
T_theileri_Tth.136.1000/1-654  -----------G---------ESADC---HAHDIMLRGKEEAIK
T_theileri_Tth.14.1410/1-752   ---------------------TKLYC---RANGVLTVRKKRIIE
T_theileri_Tth.46.1740/1-120   ------------------------------------------LL
T_theileri_Tth.31.1020/1-118   --------------------------------------------
T_theileri_Tth.124.1030/1-577  ---------------------DDKKC----GYERLPLEERNKLM
```

```
                                        490       500       510       520
T_theileri_Tth.54.1380/1-425   DEILPEAIKLHEQRLFVQPLKGPIVVPN---------FSDEN-L
T_theileri_Tth.107.1000/1-244  KEILPEAIKLHEERLFVEPLKGPIVVPQ---------FSKNDGL
T_theileri_Tth.54.1340/1-680   EEILPEAVKLHEERLFVERLKGPIVLPT---------FTNKDGL
T_theileri_Tth.117.1040/1-195  --------------------------------------------
T_theileri_Tth.6.5060/1-554    KEVLPEAIKLHRDRLNVKRVENNLILWKENYN-DTT-ERFVKY-
T_theileri_Tth.1.6360/1-511    NEILPAAVKLHADRLRVDPVKGSLVVPE---------FEEEST-
T_theileri_Tth.46.1110/1-516   NKILPAAISLHTERLKVKRVSGNLMISRSTG------VFLGKM-
T_theileri_Tth.33.1800/1-520   QQLLPVAIQLHAVRLLIHRETNSLVVPA----------FTSPI-
T_theileri_Tth.46.1020/1-842   RKVLPAALKLHSERLSVDRVE-KLELPFNKTEKE---YPVLPL-
T_theileri_Tth.70.1140/1-882   RDVLPAALKLHSERLRVEQVHGKLILPFVREKE----YPYLSE-
T_theileri_Tth.87.1050/1-130   --------------------------------------------
T_theileri_Tth.144.1010/1-374  ERVLPKAIKLHADRLKVKQVKGTLMIPRKEE------QKIRNNI
T_theileri_Tth.39.1000/1-654   DDILPAAIKLHTDRLQVQRMKTPLQVPK---------FNELSV-
T_theileri_Tth.12.1010/1-802   DKVLPKAVKLHADRLKVKQVKESLKIESDTV------TELPEE-
T_theileri_Tth.11.3500/1-528   NEVIPAAIKLHRDRLLVQPQEGKLTVPE---------FQDESY-
T_theileri_Tth.124.1020/1-215  --------------------------------------------
T_theileri_Tth.54.1390/1-443   --------------------------------------------
T_theileri_Tth.26.2480/1-314   --------------------------------------------
T_theileri_Tth.43.1970/1-318   KDILPKAIKLHTDRLKVKREEKSLNPRIAGL------DSLPEK-
T_theileri_Tth.37.1300/1-102   --------------------------------------------
T_theileri_Tth.11.2410/1-509   EKIIPAAVKLHAERLLVDPESTPLIIPQ---------FNQSTD-
T_theileri_Tth.70.1150/1-914   REVLPAALKLHSERLSVERVEDMLKLPFNETHP----YPVLPN-
T_theileri_Tth.31.1050/1-313   --------------------------------------------
T_theileri_Tth.70.1010/1-670   RKVLPAALKLHSERLSVEREKENLMVRKDV-------DKFSSK-
T_theileri_Tth.36.2090/1-806   KKILPEAIKLHEERLFVKRLNGPIVVPK---------FSDE-NL
T_theileri_Tth.117.1050/1-116  --------------------------------------------
T_theileri_Tth.11.3480/1-173   KNIIPAAIKLHRDRLRVQPHKGKIIVPK---------FEDEVF-
T_theileri_Tth.31.1070/1-736   RDVLPAALKLHSERLNVDPVKDMLKLPFKEE------YPYLPN-
T_theileri_Tth.19.2140/1-590   NHIIPGAVKLHQDRLLVKPLKHAFQVPN---------FSLGHP-
T_theileri_Tth.10.2870/1-312   --------------------------------------------
T_theileri_Tth.46.1010/1-442   RDVLPAALKLHSERLNVDPVKDMLKLPFKEE------YPYLPN-
T_theileri_Tth.12.1820/1-197   --------------------------------------------
T_theileri_Tth.2.2110/1-566    NHLLPESIKLHTDRLLVERMDGIVVD-----------WFVTPL-
T_theileri_Tth.70.1050/1-869   REVLPAALKLHSERLSVKREEENLIVRKDK-------HEFSSK-
T_theileri_Tth.71.1110/1-97    --------------------------------------------
T_theileri_Tth.4.4270/1-365    EWSIPAAIKLHTDRLLVHRAEGIVKLRR---------EDVRGQ-
T_theileri_Tth.70.1120/1-459   NKILPAAISLHTERLKVKRVSGNLRISGSTD------VFLGTF-
T_theileri_Tth.36.1940/1-208   --------------------------------------------
T_theileri_Tth.14.1390/1-407   --------------------------------------------
T_theileri_Tth.24.2340/1-572   NTILPAAIKLHKDRLRVQPHEGKLKVPN---------FEDGSD-
T_theileri_Tth.101.1060/1-233  NEVIPAAIKLHRDRLLVKPQEGKLKVTN---------FENGNP-
T_theileri_Tth.9.3510/1-513    --------------------------------------------
T_theileri_Tth.33.1860/1-659   NTIIPAAIKLHADRLLVDPVSSPLVVPA---------FKEDSV-
T_theileri_Tth.134.1010/1-723  KDLLPQAIKLHRDRLKVKPVQGNLSLWKENYE-DDKLDPMTRN-
T_theileri_Tth.11.3620/1-114   --------------------------------------------
T_theileri_Tth.144.1000/1-229  NEILPAAINLHSDLLLVKQLETPFKVPDFGK-----------TL
T_theileri_Tth.24.1780/1-518   EKIIPAAKKLHEDRLFVRRGSGKLKVPL---------FEVNND-
T_theileri_Tth.12.1030/1-678   KDLLPEAIKLHRDRLNVKPLKSNLKIQEEEV------RWFTSN-
T_theileri_Tth.129.1010/1-165  --------------------------------------------
T_theileri_Tth.2.1510/1-887    EKLLPAAIKLHSERLSVHPVQGNLVLHKTL-------FEGDAP-
T_theileri_Tth.31.1030/1-517   MDVLPAALKLHSERLSIDPEKEKLKLPFKEENQK---YPVLPN-
T_theileri_Tth.36.2110/1-825   DEILPEAIKLHEERLFVERLKGPIVVPK---------FSDK-SL
T_theileri_Tth.151.1000/1-123  KNIIPAAIKLHEERLRVQPHKGKIIVXI----------------
T_theileri_Tth.136.1000/1-654  NEILPKAIKLHTDRLLVQRMKTPLQVPK---------FSKPSV-
T_theileri_Tth.14.1410/1-752   EQLIPEAIKMHTERIFVVRETGIVKVPS----------MEHTY-
T_theileri_Tth.46.1740/1-120   NKILPAAINLHSDILLVKQLETPFKVPDFGK-----------TL
T_theileri_Tth.31.1020/1-118   --------------------------------------------
T_theileri_Tth.124.1030/1-577  NEVIPAAIKLHRDRLLVRPVSGKLKVPE---------FKDEA--
```

```
                                   530       540       550       560       570
T_theileri_Tth.54.1380/1-425   CSQFI--PTTAES-------TPISDYDMVLYVAATPTP-E----
T_theileri_Tth.107.1000/1-244  CYKFI--SQDKER-------EKSFAADMVLFVAAEPTP-E----
T_theileri_Tth.54.1340/1-680   CSKFTTPTAADKS-------TEISDADMVLYAAAVPTP-E----
T_theileri_Tth.117.1040/1-195  --------------------------------------------
T_theileri_Tth.6.5060/1-554    CQQFKM-PENHFK-------DGIPNADFMLYVDLH---QK----
T_theileri_Tth.1.6360/1-511    CSYFTVP-VSHHC-------KGVNNSDMVLYVAAKP--------
T_theileri_Tth.46.1110/1-516   CKGVGI-PAAHLE-------EGIPDADFVLYVGIAIYKPA----
T_theileri_Tth.33.1800/1-520   CLRFTVP-ASHHT-------TGVANADTVIYVASTPTSA-----
T_theileri_Tth.46.1020/1-842   CTNVSI-PKEHQQ--------GISNADFMLYVGVTTSADS----
T_theileri_Tth.70.1140/1-882   CPNVSI-PKEHRD--------GIPDADFMLYVGLIPSADS----
T_theileri_Tth.87.1050/1-130   --------------------------------------------
T_theileri_Tth.144.1010/1-374  CQFFKD-PLTKNV-------TDGPVVDFMIFASLS---KE----
T_theileri_Tth.39.1000/1-654   CSHFTIPSGHYAP-------NGVENADFVLYVAAGPSKNT----
T_theileri_Tth.12.1010/1-802   CEAFGI-PQRHKE-------KGIPDADFVIYALLT---SS----
T_theileri_Tth.11.3500/1-528   CKHFTVP-SDHHD-------KGVENADFVLYVAAGT--------
T_theileri_Tth.124.1020/1-215  --------------------------------------------
T_theileri_Tth.54.1390/1-443   --------------------------------------------
T_theileri_Tth.26.2480/1-314   --------------------------------------------
T_theileri_Tth.43.1970/1-318   CRLIKD-PLGQQM-------QYSVDVDFMIYVALS---AK----
T_theileri_Tth.37.1300/1-102   ---------------------------MVLYVAAGPPNG-----
T_theileri_Tth.11.2410/1-509   CRFFTVP-QDHHN-------PGVENADFVLYVAAKP--------
T_theileri_Tth.70.1150/1-914   CTDVSI-PKDHIK--------GIPNADFMLYVGLI---DE----
T_theileri_Tth.31.1050/1-313   --------------------------------------------
T_theileri_Tth.70.1010/1-670   CSNVSV-PKKHQE-------QGIPNVDFVLYVGVK---SS----
T_theileri_Tth.36.2090/1-806   CSQFTTPTSADKS-------TEISDADMVLYVAAEPTP-E----
T_theileri_Tth.117.1050/1-116  --------------------------------------------
T_theileri_Tth.11.3480/1-173   CGKFTVP-KEHRT-------------------------------
T_theileri_Tth.31.1070/1-736   CTNASI-PKDHRK-------KGISNADFMLYVGLT---EK----
T_theileri_Tth.19.2140/1-590   CSAFSIP-KDHYN-------PGISGYDTVMYAAAGPSHLD----
T_theileri_Tth.10.2870/1-312   --------------------------------------------
T_theileri_Tth.46.1010/1-442   CTNASI-PKDHRK-------KGISNADFMLYVGLT---EK----
T_theileri_Tth.12.1820/1-197   ---MVPVFDGDQG-------MGIPNADFVIYLGLSTKKP-----
T_theileri_Tth.2.2110/1-566    CSPFKIP-VNHHY-------PGVTGVDILLYVAAGPTPE-----
T_theileri_Tth.70.1050/1-869   CAKVSI-PKQHQE-------HGVPNVDFVLYVGVT---DT----
T_theileri_Tth.71.1110/1-97    --------------------------------------------
T_theileri_Tth.4.4270/1-365    CGWYNIP-EEHYT-------EGLGDADLHVYVSAAAS-------
T_theileri_Tth.70.1120/1-459   CKGAGI-PAAHLD-------QGIPDADFVLYVGLAIYKPA----
T_theileri_Tth.36.1940/1-208   --------------------------------------------
T_theileri_Tth.14.1390/1-407   --------------------------------------------
T_theileri_Tth.24.2340/1-572   CEYFTVP-DEHHT-------EGVENADFVFYVGSGP--------
T_theileri_Tth.101.1060/1-233  CTHFTVP-DEHRS-------EGVENADFVLYVAAGT--------
T_theileri_Tth.9.3510/1-513    CGVKTP--KNHK--------KNFPNADFVLFVCLDASKTS----
T_theileri_Tth.33.1860/1-659   CKNFTVP-ESHHK-------EGVRDADMVLYVAARN--------
T_theileri_Tth.134.1010/1-723  CKQFLV-PEEHLK-------HGIPDADFLLYVRLS---PT----
T_theileri_Tth.11.3620/1-114   --------------------------------------------
T_theileri_Tth.144.1000/1-229  CSHFTVP-TTHIS-------EGVKDAYMVLYVAAGPSNTP----
T_theileri_Tth.24.1780/1-518   CKHFTVP-EEHHS-------DGV-DADFVLYVAAVQ--------
T_theileri_Tth.12.1030/1-678   CWGFII-PSEHST-------EGIPDADFMLYVNLG---TE----
T_theileri_Tth.129.1010/1-165  --------------------------------------------
T_theileri_Tth.2.1510/1-887    CSFFKP-PEGHHS-------TGVPGADFVLYVTTNKKSNE----
T_theileri_Tth.31.1030/1-517   CTDVSI-PKEHRD-------EGISNADFMLYVGLT---EE----
T_theileri_Tth.36.2110/1-825   CSKFTTPTSADKS-------TEISDYDMVLFAAAVPTP-K----
T_theileri_Tth.151.1000/1-123  -----------RS-------------------------------
T_theileri_Tth.136.1000/1-654  CSHFTIP-GDRAP-------YGVENADFLLYVAAGPSKKL----
T_theileri_Tth.14.1410/1-752   CSHYTIP-EEHHT-------IGLQDAELYFYVSALQTD------
T_theileri_Tth.46.1740/1-120   CSHFTVP-TTHIS-------EGVEDAYMVLYVAVGPSKG-----
T_theileri_Tth.31.1020/1-118   --------------------------------------------
T_theileri_Tth.124.1030/1-577  CDFFTVP-KEHRD-------VGV-DADFVLYVIAT---------
```

580 590 600 610

```
T_theileri_Tth.54.1380/1-425   --GTFAWAATCAILS-SNG------RPVIGIINYGPRYIV----
T_theileri_Tth.107.1000/1-244  --ETFAWAATCATLG-PDG------RPVVGIINYGPRYIV----
T_theileri_Tth.54.1340/1-680   --GTFAWAATCATLG-RNG------RPVIGIINYGPRYIV----
T_theileri_Tth.117.1040/1-195  --------------------------------------------
T_theileri_Tth.6.5060/1-554    --PTKI---DECTKE-QNYG----GRPTSALITFLPKEIA----
T_theileri_Tth.1.6360/1-511    ---VNPFATICANA--SSG------RPIAAAMNFRVYRKY----
T_theileri_Tth.46.1110/1-516   --------TTVCSYN-AEE------RPTSASIKFRPRDIV----
T_theileri_Tth.33.1800/1-520   --FEPLWAIPCAQ---VGN------RFVAGVMNIGPLSID----
T_theileri_Tth.46.1020/1-842   --TNSV---KICSKN-TEG------RPTSALIKFIPIEID----
T_theileri_Tth.70.1140/1-882   --TNSV---KICSYN-DQK------RPTSALIKFVPKEIV----
T_theileri_Tth.87.1050/1-130   --------------------------------------------
T_theileri_Tth.144.1010/1-374  --PQKV---VICSQD-NEN------RPTSAVIKFIPKEIE----
T_theileri_Tth.39.1000/1-654   --NHSSWAVTCAIDD-QSK------RPIVGAMNIHPMHAD----
T_theileri_Tth.12.1010/1-802   --TRDV---KICSKD-GEG------RPTSAVIKFILQDIE----
T_theileri_Tth.11.3500/1-528   ---GEPFGVTCAPAE-ASS------RPIAGAINISPYTLV----
T_theileri_Tth.124.1020/1-215  --------------------------------------------
T_theileri_Tth.54.1390/1-443   --------------------------------------------
T_theileri_Tth.26.2480/1-314   --------------------------------------------
T_theileri_Tth.43.1970/1-318   --PENV---EICTRD-EEN------RPTSAVISFIPDEIK----
T_theileri_Tth.37.1300/1-102   --LDDIWAVPCAVLE-QDG------RPVAAGINFVVRNIV----
T_theileri_Tth.11.2410/1-509   ---GDAFGVTCANKE-PSG------RPIAGGLNFIPYQTV----
T_theileri_Tth.70.1150/1-914   --HQPP---KICSRN-TKG------RPTSALIKFVPEEID----
T_theileri_Tth.31.1050/1-313   --------------------------------------------
T_theileri_Tth.70.1010/1-670   --PDPVNDAKICSKN-ENG------RPTSALIKFIPKEIA----
T_theileri_Tth.36.2090/1-806   --GTFAWAATCATLS-SNG------RPVIGIINYGPRYIV----
T_theileri_Tth.117.1050/1-116  --------------------------------------------
T_theileri_Tth.11.3480/1-173   --------------------------------------------
T_theileri_Tth.31.1070/1-736   --YVPV---QICSKN-EKD------RPTSALIKFVPEEID----
T_theileri_Tth.19.2140/1-590   --GNVAWAILCATL--ANS------RPVTGVMHLAPESIT----
T_theileri_Tth.10.2870/1-312   --------------------------------------------
T_theileri_Tth.46.1010/1-442   --YVPV---QICSKN-EKD------RPTSALIKFVPEEID----
T_theileri_Tth.12.1820/1-197   -------GTKICTYG-DDG------RPTSAMIKLNPFEIK----
T_theileri_Tth.2.2110/1-566    --GVIAWGVPCYTL--DNG------RPVVGAMNFGPRYIT----
T_theileri_Tth.70.1050/1-869   --SVPV---QICSKN-EKG------RPTSALIKFVPEEID----
T_theileri_Tth.71.1110/1-97    --------------------------------------------
T_theileri_Tth.4.4270/1-365    --DSYSREFTCQRL--KNH------RPIAVAINLEPTSVL----
T_theileri_Tth.70.1120/1-459   --------TTVCSYN-AEK------RPTSASIKFRPRDIA----
T_theileri_Tth.36.1940/1-208   --------------------------------------------
T_theileri_Tth.14.1390/1-407   --------------------------------------------
T_theileri_Tth.24.2340/1-572   ---GTSFGVTCAPED-LSS------RPIAGAMNIAVFKYA----
T_theileri_Tth.101.1060/1-233  ---GEPFGVTCAPAE-ASS------RPIAGAINISPYT------
T_theileri_Tth.9.3510/1-513    --------TRVYTEE--DN------RPTSALIKFVPKDIV----
T_theileri_Tth.33.1860/1-659   ---LKEFGVICAKN--DDG------RPIAGAINFCLYTRR----
T_theileri_Tth.134.1010/1-723  --EQDY---HICTKD-RGNQ----IRPTSARISFVPKEIN----
T_theileri_Tth.11.3620/1-114   --------------------------------------------
T_theileri_Tth.144.1000/1-229  --FIDNYNTS--------GN----ECLSCGERKNIPTERE----
T_theileri_Tth.24.1780/1-518   --WHWGSGVTCAVEN-STG------RPIVGAMNYVPQLQD----
T_theileri_Tth.12.1030/1-678   --RKSS---AVCSID-ENK------RPTSARISFVPKEIV----
T_theileri_Tth.129.1010/1-165  --------------------------------------------
T_theileri_Tth.2.1510/1-887    -------SVKICAYG-YGH------RPIAAVKNFLPSEIG----
T_theileri_Tth.31.1030/1-517   --YKPV---KICSRN-EKNH-----RPTSAFIKFIPREIA----
T_theileri_Tth.36.2110/1-825   --GTFAWAATCATLG-PNG------RPVVGIINYGPRYIV----
T_theileri_Tth.151.1000/1-123  --------------------------------------------
T_theileri_Tth.136.1000/1-654  --TSFTWAVTCAIDD-QSK------RPIVGAMNIHPMHAD----
T_theileri_Tth.14.1410/1-752   --RAMAWGAYCACT--DDG------RPIVGSLNLNPTYVE----
T_theileri_Tth.46.1740/1-120   --LDDNYNTS--------GN----EFLCCGERKNIPTERV----
T_theileri_Tth.31.1020/1-118   --------------------------------------------
T_theileri_Tth.124.1030/1-577  ---KTTFGYTCAWD--STG------RPIVGAVSYASESHV----
```

```
                                      620       630       640       650
T_theileri_Tth.54.1380/1-425    --ATPQRVRVAAHEIAHALGFSFSEMERK-NM-----LPIWN--
T_theileri_Tth.107.1000/1-244   --ATPQRVRVAAHEIAH---------------------------
T_theileri_Tth.54.1340/1-680    --ATPQRVRVAAHEIAHALGFNFELMERK-EL-----GGKAD--
T_theileri_Tth.117.1040/1-195   --------------------------------------------
T_theileri_Tth.6.5060/1-554     --ATRQYIRLAARDIAIGLGFEILY--IG-GQ-----NMPYP--
T_theileri_Tth.1.6360/1-511     --GLRYNVRLATHEIAHALGFDYQNFVDN-KM-----VSTLE--
T_theileri_Tth.46.1110/1-516    --DTRHFTRIVAHEIGHGLGFNNRLMNSK-EM-----LTLVT--
T_theileri_Tth.33.1800/1-520    --GTRISSRHVAHEIAHILGFEYTMMHSL-GM-----TTNVS--
T_theileri_Tth.46.1020/1-842    --ATRYFIRFTAHEVAHALGFEIEMMKKY-ME-----IKEEK--
T_theileri_Tth.70.1140/1-882    --DTRHFIRLTAHEVAHALGFDIETMKEH-KV-----IVEEN--
T_theileri_Tth.87.1050/1-130    --------------------------------------------
T_theileri_Tth.144.1010/1-374   --ATRQYIRLTAHEIAHGLGFQHELMKEL-NM-----IKERS--
T_theileri_Tth.39.1000/1-654    --FTRVNVRLAAHELAHALGFDYERMKER-GM-----TRYSS--
T_theileri_Tth.12.1010/1-802    --ATRKYIRFTTHEIAHGLGFQHEFMDNL-TM-----IEPSN--
T_theileri_Tth.11.3500/1-528    --HPRWGVRAVAHEMAHALGFDCKRMEQL-KM-----VTKIN--
T_theileri_Tth.124.1020/1-215   --------------------------------------------
T_theileri_Tth.54.1390/1-443    -------------------------------M-----VRQGI--
T_theileri_Tth.26.2480/1-314    ----------------------------M-GMIARSGITTTT--
T_theileri_Tth.43.1970/1-318    --ATRQYIRLTAHEIAHGLGFDYEVMETQ-GM-----IEPRK--
T_theileri_Tth.37.1300/1-102    --FERSVARVTAHVIAHALGFSYGRMKAL-NM-----ISNVT--
T_theileri_Tth.11.2410/1-509    --STRPNVRQAAHHIAHALGFDYERMKSL-GM-----ISVIT--
T_theileri_Tth.70.1150/1-914    --ATRHYIRFTAHEVAHALGFEIEMMKEH-VK-----EGKET--
T_theileri_Tth.31.1050/1-313    --------------------------------------------
T_theileri_Tth.70.1010/1-670    --DTRHFIRFAAHEVAHALGFETEMMNKYFDV-----VEEEN--
T_theileri_Tth.36.2090/1-806    --ATPQRVRVAAHEIAHALGFNVPEMERM-QL-----VGRVD--
T_theileri_Tth.117.1050/1-116   --AT----------------------------------------
T_theileri_Tth.11.3480/1-173    --------------------------------------------
T_theileri_Tth.31.1070/1-736    --DTRYFIRFAAHEVAHALGFDIEIMKEH-KV-----IVKEN--
T_theileri_Tth.19.2140/1-590    --NTSQMVRVVAHEMAHILGFDREVFSAN-KM-----ITLVH--
T_theileri_Tth.10.2870/1-312    --------------------------------------------
T_theileri_Tth.46.1010/1-442    --DTRHFIRFAAHEVAHALGFDIETIKNYINV----TVEEKE--
T_theileri_Tth.12.1820/1-197    --YTQQYVRLVAHEIAHGLGFSMDLDKFR-QM-----VLNGE--
T_theileri_Tth.2.2110/1-566     --ATRLVSRAAAHEIAHVLGFSSFLFAKS-GM-----LSIVP--
T_theileri_Tth.70.1050/1-869    --ATRHFIRFAAHEVAHALGFEIETMKEY-VK-----EGNEM--
T_theileri_Tth.71.1110/1-97     --------------------------------------------
T_theileri_Tth.4.4270/1-365     --ATEHHIRTVAHEIAHGLGFDGTTFAHL-KM-----ISAVE--
T_theileri_Tth.70.1120/1-459    --DTRHFTRIVAHEIGHGLGFNSRVIGSR-EM-----LTIVT--
T_theileri_Tth.36.1940/1-208    --------------------------------------------
T_theileri_Tth.14.1390/1-407    -----------------ALGFDSAMFPRL-GM-----MGTFF--
T_theileri_Tth.24.2340/1-572    --GLRLSVRSIAHEMAHALGFDYERMKEL-NM-----VTTVT--
T_theileri_Tth.101.1060/1-233   --------------------------------------------
T_theileri_Tth.9.3510/1-513     --DTRHFVRTAAHEIAHGLGFDVGRMRKL-KK-----IKSGT--
T_theileri_Tth.33.1860/1-659    --SQQFFVRSAAHEIAHALGFDYDVFVNN-NM-----VAESA--
T_theileri_Tth.134.1010/1-723   --ATRHFIRMAARNIAFGLGFKEFVFGTS-SS-----ISGRV--
T_theileri_Tth.11.3620/1-114    --------------------------------------------
T_theileri_Tth.144.1000/1-229   --LIGGVHKSHVHRIIAILLLTWMSKRKKQII------------
T_theileri_Tth.24.1780/1-518    --GIRFNVRRIAREIAHALGFNFVEMEKK-GI-----VTEVE--
T_theileri_Tth.12.1030/1-678    --ATRHYIRLAAHDIAVGLGFTTEY-MSK-YL-----TKESA--
T_theileri_Tth.129.1010/1-165   --------------------------------------------
T_theileri_Tth.2.1510/1-887     --ETRRLVRMAAHDIAHALGFDTRRMERI-GM-----IYRPKRS
T_theileri_Tth.31.1030/1-517    --ATRHYIRFAAHEVAHALGFDIEIMKEN-NV-----IEEKQ--
T_theileri_Tth.36.2110/1-825    --ATPQRVRVAAHEIAHALGFNFEAMKRK-GM-----VGEVN--
T_theileri_Tth.151.1000/1-123   --------------------------------------------
T_theileri_Tth.136.1000/1-654   --FTRVNVRLAAHELAHALGFDYERMKER-GM-----TRYSS--
T_theileri_Tth.14.1410/1-752    --ATDAAVRTVAHEIAHLLGFDYYMFLKL-GI-----VDTGV--
T_theileri_Tth.46.1740/1-120    --LIGGVHKA----MCMGLLLFYRGEKQLQEV------------
T_theileri_Tth.31.1020/1-118    --------------------------------------------
T_theileri_Tth.124.1030/1-577   --GLRLRVRRVAHEIAHALGFSFREMGKK-GI-----MFEEE--
```

	670 680 690 700
T_theileri_Tth.54.1380/1-425	-NSYN-------------RF---DVPLVNSSNVLRE-TKNHYNC
T_theileri_Tth.107.1000/1-244	--
T_theileri_Tth.54.1340/1-680	-SLRD-------------TA---DVYVVSSNNTRRE-AMKHYNC
T_theileri_Tth.117.1040/1-195	--
T_theileri_Tth.6.5060/1-554	-LLDV------NG-V---KK---YVRVLTGKDLKEK-MNEHYNC
T_theileri_Tth.1.6360/1-511	-NLRGK-----------------SRVVVVSPRTLEE-AKKHYGC
T_theileri_Tth.46.1110/1-516	------------------GGSYGVQYEFRSTLMEKM-VRDHYGC
T_theileri_Tth.33.1800/1-520	-GIRG-------------KTN--DVVVVSSAKTKAI-TQTYYEC
T_theileri_Tth.46.1020/1-842	-TG---------------KK---EVQLVASNALIDK-MKEQYKC
T_theileri_Tth.70.1140/1-882	-TG----------------K---KVLLVESNDVIEK-MKAQYAC
T_theileri_Tth.87.1050/1-130	--
T_theileri_Tth.144.1010/1-374	-IDLP------RELVPYGRN---EFYMVNSTKIVEV-LKSHYRC
T_theileri_Tth.39.1000/1-654	-SYHD------------------NRAVIISAKVLEK-ARDHYSC
T_theileri_Tth.12.1010/1-802	-SYLE------SKGVSVRVN---TPRILRFKNSVDM-LKNHYGC
T_theileri_Tth.11.3500/1-528	--VRDK-----------------DRKVVSSKLTKEK-AQAHYNC
T_theileri_Tth.124.1020/1-215	---------------------------IASQVNR----------
T_theileri_Tth.54.1390/1-443	-SVRG-------------KS---NISFVVSQNTRRE-SIGHYNC
T_theileri_Tth.26.2480/1-314	------------------TAEKRVMFTVNSHSTVEA-AKWHYGC
T_theileri_Tth.43.1970/1-318	-NGLS------STGGKHGGE---EFYM-NSSE-----TXLHCSC
T_theileri_Tth.37.1300/1-102	-NKRK-------------DN---KAVVVKSEKKNEG--------
T_theileri_Tth.11.2410/1-509	--VRGV-----------------KRVVVSSDMTKKM-AQEHYGC
T_theileri_Tth.70.1150/1-914	-TG---------------KK---KVRLVTYPTVIEK-MKDQYKC
T_theileri_Tth.31.1050/1-313	--
T_theileri_Tth.70.1010/1-670	-AG---------------NK---LVYLVKSNNLVDKMMKAQYDC
T_theileri_Tth.36.2090/1-806	-SLRG-------------KT---NVALVASDNTRRE-AMKHYNC
T_theileri_Tth.117.1050/1-116	-------------------------------------TEEPWQC
T_theileri_Tth.11.3480/1-173	--
T_theileri_Tth.31.1070/1-736	-TG----------------K---TFSLVTSNTVLDEKMKEQYDC
T_theileri_Tth.19.2140/1-590	-DVRG-------------KS---NVHMLTSEKVMEK-AREHYNC
T_theileri_Tth.10.2870/1-312	--
T_theileri_Tth.46.1010/1-442	-TG---------------KK---IVRLVKAPIVLDK-MKGHYGC
T_theileri_Tth.12.1820/1-197	------------------NSSYPGYKELSSPEVKNA-VQDHYIC
T_theileri_Tth.2.2110/1-566	-ILRG-------------KR---DVLVVSSKKTLEV-MRKHFNC
T_theileri_Tth.70.1050/1-869	-AG---------------KK---KVSWVTYPTVIKE-VNKHYGC
T_theileri_Tth.71.1110/1-97	--
T_theileri_Tth.4.4270/1-365	NDVRG-------------KP---HVFLVVSPKAKEV-AQKYYNC
T_theileri_Tth.70.1120/1-459	------------------DGNYGVQYEVRRTLMVEK-VREHYDC
T_theileri_Tth.36.1940/1-208	--
T_theileri_Tth.14.1390/1-407	-GVRG-------------RE---FVWMINVSKAKEV-ARKYFNC
T_theileri_Tth.24.2340/1-572	-NVRDK-----------------DRVLVNTAVTKEK-AQEHYNC
T_theileri_Tth.101.1060/1-233	--
T_theileri_Tth.9.3510/1-513	------------------VGAIGKVTAVDSEIMQEM-MRKHYGC
T_theileri_Tth.33.1860/1-659	-FADGKK----------------KRMLVVSKQTKEK-VQKHYGC
T_theileri_Tth.134.1010/1-723	-GVFL------NG-G---KK---DVTIVTGGNMKEK-IVEQFNC
T_theileri_Tth.11.3620/1-114	--
T_theileri_Tth.144.1000/1-229	--
T_theileri_Tth.24.1780/1-518	--LRGA-----------------KRKVVNSSKTVEK-AQKHYNC
T_theileri_Tth.12.1030/1-678	-PIFM------NG-V---PN---YLMMVQSNTVKDK-AKKHYNC
T_theileri_Tth.129.1010/1-165	--
T_theileri_Tth.2.1510/1-887	SNSTMSSGG--------------LIYFVRSPNTKEV-VRKHYAC
T_theileri_Tth.31.1030/1-517	----------------EGNK---KIRLVESKAVLDK-MKEHYGC
T_theileri_Tth.36.2110/1-825	-SLRG-------------KS---DVARVTSDNTRRE-AIDHYKC
T_theileri_Tth.151.1000/1-123	--
T_theileri_Tth.136.1000/1-654	-SYHD------------------NRAVIISAKVLEK-ARDHYSC
T_theileri_Tth.14.1410/1-752	-TLRQR-----------------VVAVINTTQSKKV-ASEHYGC
T_theileri_Tth.46.1740/1-120	--
T_theileri_Tth.31.1020/1-118	--
T_theileri_Tth.124.1030/1-577	------------------LEGVRMRRFVNSTVTVKK-AQKHYNC

```
                                   710       720       730       740
T_theileri_Tth.54.1380/1-425  NS--------AEGMKIQNE-------------------------
T_theileri_Tth.107.1000/1-244 --------------------------------------------
T_theileri_Tth.54.1340/1-680  DS--------AEGMELQGV-------------------------
T_theileri_Tth.117.1040/1-195 --------------------------------------------
T_theileri_Tth.6.5060/1-554   -----KD---YGGMIVHYN-------------------------
T_theileri_Tth.1.6360/1-511   GN--------LKGVHL----------------------------
T_theileri_Tth.46.1110/1-516  HN--------ATGIRLEND-------------------------
T_theileri_Tth.33.1800/1-520  SS--------LVGMELEDE-------------------------
T_theileri_Tth.46.1020/1-842  PSDEGNA--EIKGMPLQSK-------------------------
T_theileri_Tth.70.1140/1-882  ANDN-----EIKSLVFEND-------------------------
T_theileri_Tth.87.1050/1-130  --------------------------------------------
T_theileri_Tth.144.1010/1-374 KDGE------IEGLYLEDE-------------------------
T_theileri_Tth.39.1000/1-654  LS--------IEEVELEHT-------------------------
T_theileri_Tth.12.1010/1-802  EN---GE---IKGLYLENY-------------------------
T_theileri_Tth.11.3500/1-528  ST--------LEGMELQLG-------------------------
T_theileri_Tth.124.1020/1-215 --------------------------------------------
T_theileri_Tth.54.1390/1-443  EN--------VQGMELEDE-------------------------
T_theileri_Tth.26.2480/1-314  SS--------LREMRLDHN-------------------------
T_theileri_Tth.43.1970/1-318  CTA-------AAGVWLL---------------------------
T_theileri_Tth.37.1300/1-102  --------------------------------------------
T_theileri_Tth.11.2410/1-509  SN--------LEGMELDYR-------------------------
T_theileri_Tth.70.1150/1-914  TNDEGDN--EITGMALENE-------------------------
T_theileri_Tth.31.1050/1-313  --------------------------------------------
T_theileri_Tth.70.1010/1-670  SNGN-----EIKGMILQNK-------------------------
T_theileri_Tth.36.2090/1-806  DS--------AKGMELQNTPNPQKTNGRGGSGGERKVQQHTAPA
T_theileri_Tth.117.1050/1-116 RP------------------------------------------
T_theileri_Tth.11.3480/1-173  --------------------------------------------
T_theileri_Tth.31.1070/1-736  SNDG-----EIKGMPLQSE-------------------------
T_theileri_Tth.19.2140/1-590  SS--------LDGIEMEK--------------------------
T_theileri_Tth.10.2870/1-312  --------------------------------------------
T_theileri_Tth.46.1010/1-442  SDDDKCDE-VIKKVAFENE-------------------------
T_theileri_Tth.12.1820/1-197  DD--------YIGMKLDNS-------------------------
T_theileri_Tth.2.2110/1-566   DD--------VVGMELEDE-------------------------
T_theileri_Tth.70.1050/1-869  PIVEGDS--EIKGMALEND-------------------------
T_theileri_Tth.71.1110/1-97   --------------------------------------------
T_theileri_Tth.4.4270/1-365   SK--------APGLELEDQ-------------------------
T_theileri_Tth.70.1120/1-459  PT--------ATGMKLEKD-------------------------
T_theileri_Tth.36.1940/1-208  --------------------------------------------
T_theileri_Tth.14.1390/1-407  PT--------AEGLEMEST-------------------------
T_theileri_Tth.24.2340/1-572  PE--------LQGMELNNE-------------------------
T_theileri_Tth.101.1060/1-233 --------------------------------------------
T_theileri_Tth.9.3510/1-513   TLQH------ITGMYMEDE-------------------------
T_theileri_Tth.33.1860/1-659  DT--------AQGMELEYE-------------------------
T_theileri_Tth.134.1010/1-723 -----SS---YGGIPLDFK-------------------------
T_theileri_Tth.11.3620/1-114  --------------------------------------------
T_theileri_Tth.144.1000/1-229 --------------------------------------------
T_theileri_Tth.24.1780/1-518  PN--------LKGMELEPH-------------------------
T_theileri_Tth.12.1030/1-678  -----QN---IKGMILDYK-------------------------
T_theileri_Tth.129.1010/1-165 --------------------------------------------
T_theileri_Tth.2.1510/1-887   ENS-------TNGMRLDME-------------------------
T_theileri_Tth.31.1030/1-517  SDGKCDE--VIKKVAFENE-------------------------
T_theileri_Tth.36.2110/1-825  DS--------AKGMELSVIRRPLFAARGDSILKGRQMQS-----
T_theileri_Tth.151.1000/1-123 --------------------------------------------
T_theileri_Tth.136.1000/1-654 LS--------IEEVELEHT-------------------------
T_theileri_Tth.14.1410/1-752  PN--------ATGVELEST-------------------------
T_theileri_Tth.46.1740/1-120  --------------------------------------------
T_theileri_Tth.31.1020/1-118  --------------------------------------------
T_theileri_Tth.124.1030/1-577 PT--------LKEMNLVYN-------------------------
```

```
                              750       760       770       780       790
T_theileri_Tth.54.1380/1-425  ----------------------------LFFMQLK---------
T_theileri_Tth.107.1000/1-244 --------------------------------------------
T_theileri_Tth.54.1340/1-680  ----------------------------AFVQI-----------
T_theileri_Tth.117.1040/1-195 --------------------------------------------
T_theileri_Tth.6.5060/1-554   --------------------------------------------
T_theileri_Tth.1.6360/1-511   --------------------------------------------
T_theileri_Tth.46.1110/1-516  --------------------------------------------
T_theileri_Tth.33.1800/1-520  --------------------------------------------
T_theileri_Tth.46.1020/1-842  --------------------------------------------
T_theileri_Tth.70.1140/1-882  --------------------------------------------
T_theileri_Tth.87.1050/1-130  --------------------------------------------
T_theileri_Tth.144.1010/1-374 --------------------------------------------
T_theileri_Tth.39.1000/1-654  --------------------------------------------
T_theileri_Tth.12.1010/1-802  --------------------------------------------
T_theileri_Tth.11.3500/1-528  --------------------------------------------
T_theileri_Tth.124.1020/1-215 --------------------------------------------
T_theileri_Tth.54.1390/1-443  --------------------------------------------
T_theileri_Tth.26.2480/1-314  --------------------------------------------
T_theileri_Tth.43.1970/1-318  --------------------------------------------
T_theileri_Tth.37.1300/1-102  --------------------------------------------
T_theileri_Tth.11.2410/1-509  --------------------------------------------
T_theileri_Tth.70.1150/1-914  --------------------------------------------
T_theileri_Tth.31.1050/1-313  --------------------------------------------
T_theileri_Tth.70.1010/1-670  --------------------------------------------
T_theileri_Tth.36.2090/1-806  GITPSREAGSLKHSLRLEQDARGVLSRPPLPRQ-----------
T_theileri_Tth.117.1050/1-116 --------------------------------------------
T_theileri_Tth.11.3480/1-173  --------------------------------------------
T_theileri_Tth.31.1070/1-736  --------------------------------------------
T_theileri_Tth.19.2140/1-590  --------------------------------------------
T_theileri_Tth.10.2870/1-312  --------------------------------------------
T_theileri_Tth.46.1010/1-442  --------------------------------------------
T_theileri_Tth.12.1820/1-197  --------------------------------------------
T_theileri_Tth.2.2110/1-566   --------------------------------------------
T_theileri_Tth.70.1050/1-869  --------------------------------------------
T_theileri_Tth.71.1110/1-97   --------------------------------------------
T_theileri_Tth.4.4270/1-365   --------------------------------------------
T_theileri_Tth.70.1120/1-459  --------------------------------------------
T_theileri_Tth.36.1940/1-208  --------------------------------------------
T_theileri_Tth.14.1390/1-407  --------------------------------------------
T_theileri_Tth.24.2340/1-572  --------------------------------------------
T_theileri_Tth.101.1060/1-233 --------------------------------------------
T_theileri_Tth.9.3510/1-513   --------------------------------------------
T_theileri_Tth.33.1860/1-659  --------------------------------------------
T_theileri_Tth.134.1010/1-723 --------------------------------------------
T_theileri_Tth.11.3620/1-114  --------------------------------------------
T_theileri_Tth.144.1000/1-229 --------------------------------------------
T_theileri_Tth.24.1780/1-518  --------------------------------------------
T_theileri_Tth.12.1030/1-678  --------------------------------------------
T_theileri_Tth.129.1010/1-165 --------------------------------------------
T_theileri_Tth.2.1510/1-887   --------------------------------------------
T_theileri_Tth.31.1030/1-517  --------------------------------------------
T_theileri_Tth.36.2110/1-825  --------------------------------------------
T_theileri_Tth.151.1000/1-123 --------------------------------------------
T_theileri_Tth.136.1000/1-654 --------------------------------------------
T_theileri_Tth.14.1410/1-752  --------------------------------------------
T_theileri_Tth.46.1740/1-120  --------------------------------------------
T_theileri_Tth.31.1020/1-118  --------------------------------------------
T_theileri_Tth.124.1030/1-577 --------------------------------------------
```

```
                                800       810       820       830
T_theileri_Tth.54.1380/1-425    -------------------------------------------S
T_theileri_Tth.107.1000/1-244   --------------------------------------------
T_theileri_Tth.54.1340/1-680    -----RKPSETEVAQRSGAQ------------PSTLTPK-GSTI
T_theileri_Tth.117.1040/1-195   --------------------------------------------
T_theileri_Tth.6.5060/1-554     --------------------------------------------
T_theileri_Tth.1.6360/1-511     --------------------------------------------
T_theileri_Tth.46.1110/1-516    --------------------------------------------
T_theileri_Tth.33.1800/1-520    --------------------------------------------
T_theileri_Tth.46.1020/1-842    --------------------------------------------
T_theileri_Tth.70.1140/1-882    --------------------------------------------
T_theileri_Tth.87.1050/1-130    --------------------------------------------
T_theileri_Tth.144.1010/1-374   --------------------------------------------
T_theileri_Tth.39.1000/1-654    --------------------------------------------
T_theileri_Tth.12.1010/1-802    --------------------------------------------
T_theileri_Tth.11.3500/1-528    --------------------------------------------
T_theileri_Tth.124.1020/1-215   --------------------------------------------
T_theileri_Tth.54.1390/1-443    --NPYAMIRPGSQASERSRRPRGHQTVLGGPRQGRRNRQILPSS
T_theileri_Tth.26.2480/1-314    --------------------------------------------
T_theileri_Tth.43.1970/1-318    --------------------------------------------
T_theileri_Tth.37.1300/1-102    --------------------------------------------
T_theileri_Tth.11.2410/1-509    --------------------------------------------
T_theileri_Tth.70.1150/1-914    --------------------------------------------
T_theileri_Tth.31.1050/1-313    --------------------------------------------
T_theileri_Tth.70.1010/1-670    --------------------------------------------
T_theileri_Tth.36.2090/1-806    -----QLLPRRERLQQRQGH--------------------LQAP
T_theileri_Tth.117.1050/1-116   --------------------------------------------
T_theileri_Tth.11.3480/1-173    --------------------------------------------
T_theileri_Tth.31.1070/1-736    --------------------------------------------
T_theileri_Tth.19.2140/1-590    --------------------------------------------
T_theileri_Tth.10.2870/1-312    --------------------------------------------
T_theileri_Tth.46.1010/1-442    --------------------------------------------
T_theileri_Tth.12.1820/1-197    --------------------------------------------
T_theileri_Tth.2.2110/1-566     --------------------------------------------
T_theileri_Tth.70.1050/1-869    --------------------------------------------
T_theileri_Tth.71.1110/1-97     --------------------------------------------
T_theileri_Tth.4.4270/1-365     --------------------------------------------
T_theileri_Tth.70.1120/1-459    --------------------------------------------
T_theileri_Tth.36.1940/1-208    --------------------------------------------
T_theileri_Tth.14.1390/1-407    --------------------------------------------
T_theileri_Tth.24.2340/1-572    --------------------------------------------
T_theileri_Tth.101.1060/1-233   --------------------------------------------
T_theileri_Tth.9.3510/1-513     --------------------------------------------
T_theileri_Tth.33.1860/1-659    --------------------------------------------
T_theileri_Tth.134.1010/1-723   --------------------------------------------
T_theileri_Tth.11.3620/1-114    --------------------------------------------
T_theileri_Tth.144.1000/1-229   --------------------------------------------
T_theileri_Tth.24.1780/1-518    --------------------------------------------
T_theileri_Tth.12.1030/1-678    --------------------------------------------
T_theileri_Tth.129.1010/1-165   --------------------------------------------
T_theileri_Tth.2.1510/1-887     --------------------------------------------
T_theileri_Tth.31.1030/1-517    --------------------------------------------
T_theileri_Tth.36.2110/1-825    -----RLGTQTAHAGNRRDRSAQLQGPQVRITRPSRPRPQPSTV
T_theileri_Tth.151.1000/1-123   --------------------------------------------
T_theileri_Tth.136.1000/1-654   --------------------------------------------
T_theileri_Tth.14.1410/1-752    --------------------------------------------
T_theileri_Tth.46.1740/1-120    --------------------------------------------
T_theileri_Tth.31.1020/1-118    --------------------------------------------
T_theileri_Tth.124.1030/1-577   --------------------------------------------
```

```
                                     840       850       860       870
T_theileri_Tth.54.1380/1-425    NMAAQTVSLESPLRSSL----------------LSAVQAEFTEEE
T_theileri_Tth.107.1000/1-244   --------------------------------------------
T_theileri_Tth.54.1340/1-680    LAQVASVREEYELGESH----------------SNVVDGRLTGKV
T_theileri_Tth.117.1040/1-195   --------------------------------------------
T_theileri_Tth.6.5060/1-554     --------------------------------------------
T_theileri_Tth.1.6360/1-511     --------------------------------------------
T_theileri_Tth.46.1110/1-516    --------------------------------------------
T_theileri_Tth.33.1800/1-520    --------------------------------------------
T_theileri_Tth.46.1020/1-842    --------------------------------------------
T_theileri_Tth.70.1140/1-882    --------------------------------------------
T_theileri_Tth.87.1050/1-130    --------------------------------------------
T_theileri_Tth.144.1010/1-374   --------------------------------------------
T_theileri_Tth.39.1000/1-654    --------------------------------------------
T_theileri_Tth.12.1010/1-802    --------------------------------------------
T_theileri_Tth.11.3500/1-528    --------------------------------------------
T_theileri_Tth.124.1020/1-215   --------------------------------------------
T_theileri_Tth.54.1390/1-443    AAQEASVREEYDQPEVQ----------------GNIPSADFDHHM
T_theileri_Tth.26.2480/1-314    --------------------------------------------
T_theileri_Tth.43.1970/1-318    --------------------------------------------
T_theileri_Tth.37.1300/1-102    --------------------------------------------
T_theileri_Tth.11.2410/1-509    --------------------------------------------
T_theileri_Tth.70.1150/1-914    --------------------------------------------
T_theileri_Tth.31.1050/1-313    --------------------------------------------
T_theileri_Tth.70.1010/1-670    --------------------------------------------
T_theileri_Tth.36.2090/1-806    RVLTASVQEEYQLGVSH----------------SNVMDGRLTGKE
T_theileri_Tth.117.1050/1-116   --------------------------------------------
T_theileri_Tth.11.3480/1-173    --------------------------------------------
T_theileri_Tth.31.1070/1-736    --------------------------------------------
T_theileri_Tth.19.2140/1-590    --------------------------------------------
T_theileri_Tth.10.2870/1-312    --------------------------------------------
T_theileri_Tth.46.1010/1-442    --------------------------------------------
T_theileri_Tth.12.1820/1-197    --------------------------------------------
T_theileri_Tth.2.2110/1-566     --------------------------------------------
T_theileri_Tth.70.1050/1-869    --------------------------------------------
T_theileri_Tth.71.1110/1-97     --------------------------------------------
T_theileri_Tth.4.4270/1-365     --------------------------------------------
T_theileri_Tth.70.1120/1-459    --------------------------------------------
T_theileri_Tth.36.1940/1-208    --------------------------------------------
T_theileri_Tth.14.1390/1-407    --------------------------------------------
T_theileri_Tth.24.2340/1-572    --------------------------------------------
T_theileri_Tth.101.1060/1-233   --------------------------------------------
T_theileri_Tth.9.3510/1-513     --------------------------------------------
T_theileri_Tth.33.1860/1-659    --------------------------------------------
T_theileri_Tth.134.1010/1-723   --------------------------------------------
T_theileri_Tth.11.3620/1-114    --------------------------------------------
T_theileri_Tth.144.1000/1-229   --------------------------------------------
T_theileri_Tth.24.1780/1-518    --------------------------------------------
T_theileri_Tth.12.1030/1-678    --------------------------------------------
T_theileri_Tth.129.1010/1-165   --------------------------------------------
T_theileri_Tth.2.1510/1-887     --------------------------------------------
T_theileri_Tth.31.1030/1-517    --------------------------------------------
T_theileri_Tth.36.2110/1-825    HAQVASVQYMYDLGETH----------------SNAMDDRLTAME
T_theileri_Tth.151.1000/1-123   --------------------------------------------
T_theileri_Tth.136.1000/1-654   --------------------------------------------
T_theileri_Tth.14.1410/1-752    --------------------------------------------
T_theileri_Tth.46.1740/1-120    --------------------------------------------
T_theileri_Tth.31.1020/1-118    --------------------------------------------
T_theileri_Tth.124.1030/1-577   --------------------------------------------
```

```
                                            890       900       910       920
T_theileri_Tth.54.1380/1-425   RSMEGAEPHMGENHLRNADTHSTSEITSGSSNL--------RGN
T_theileri_Tth.107.1000/1-244  --------------------------------------------
T_theileri_Tth.54.1340/1-680   SVGNVVQERRGEE---GSSAAKFTQNVHHSSNV--------LSK
T_theileri_Tth.117.1040/1-195  --------------------------------------------
T_theileri_Tth.6.5060/1-554    --------------------------------------------
T_theileri_Tth.1.6360/1-511    --------------------------------------------
T_theileri_Tth.46.1110/1-516   --------------------------------------------
T_theileri_Tth.33.1800/1-520   --------------------------------------------
T_theileri_Tth.46.1020/1-842   --------------------------------------------
T_theileri_Tth.70.1140/1-882   --------------------------------------------
T_theileri_Tth.87.1050/1-130   --------------------------------------------
T_theileri_Tth.144.1010/1-374  --------------------------------------------
T_theileri_Tth.39.1000/1-654   --------------------------------------------
T_theileri_Tth.12.1010/1-802   --------------------------------------------
T_theileri_Tth.11.3500/1-528   --------------------------------------------
T_theileri_Tth.124.1020/1-215  --------------------------------------------
T_theileri_Tth.54.1390/1-443   NLTESFNYNRPLP-------------------------------
T_theileri_Tth.26.2480/1-314   --------------------------------------------
T_theileri_Tth.43.1970/1-318   --------------------------------------------
T_theileri_Tth.37.1300/1-102   --------------------------------------------
T_theileri_Tth.11.2410/1-509   --------------------------------------------
T_theileri_Tth.70.1150/1-914   --------------------------------------------
T_theileri_Tth.31.1050/1-313   --------------------------------------------
T_theileri_Tth.70.1010/1-670   --------------------------------------------
T_theileri_Tth.36.2090/1-806   SVGSDVQERRGEE---GSSAAKYTENVHHSSNV--------LSK
T_theileri_Tth.117.1050/1-116  --------------------------------------------
T_theileri_Tth.11.3480/1-173   --------------------------------------------
T_theileri_Tth.31.1070/1-736   --------------------------------------------
T_theileri_Tth.19.2140/1-590   --------------------------------------------
T_theileri_Tth.10.2870/1-312   --------------------------------------------
T_theileri_Tth.46.1010/1-442   --------------------------------------------
T_theileri_Tth.12.1820/1-197   --------------------------------------------
T_theileri_Tth.2.2110/1-566    --------------------------------------------
T_theileri_Tth.70.1050/1-869   --------------------------------------------
T_theileri_Tth.71.1110/1-97    --------------------------------------------
T_theileri_Tth.4.4270/1-365    --------------------------------------------
T_theileri_Tth.70.1120/1-459   --------------------------------------------
T_theileri_Tth.36.1940/1-208   --------------------------------------------
T_theileri_Tth.14.1390/1-407   --------------------------------------------
T_theileri_Tth.24.2340/1-572   --------------------------------------------
T_theileri_Tth.101.1060/1-233  --------------------------------------------
T_theileri_Tth.9.3510/1-513    --------------------------------------------
T_theileri_Tth.33.1860/1-659   --------------------------------------------
T_theileri_Tth.134.1010/1-723  --------------------------------------------
T_theileri_Tth.11.3620/1-114   --------------------------------------------
T_theileri_Tth.144.1000/1-229  --------------------------------------------
T_theileri_Tth.24.1780/1-518   --------------------------------------------
T_theileri_Tth.12.1030/1-678   --------------------------------------------
T_theileri_Tth.129.1010/1-165  --------------------------------------------
T_theileri_Tth.2.1510/1-887    --------------------------------------------
T_theileri_Tth.31.1030/1-517   --------------------------------------------
T_theileri_Tth.36.2110/1-825   SVGSVVQERRGEE---GFSSAKFTQNVHHSSNV--------LSK
T_theileri_Tth.151.1000/1-123  --------------------------------------------
T_theileri_Tth.136.1000/1-654  --------------------------------------------
T_theileri_Tth.14.1410/1-752   --------------------------------------------
T_theileri_Tth.46.1740/1-120   --------------------------------------------
T_theileri_Tth.31.1020/1-118   --------------------------------------------
T_theileri_Tth.124.1030/1-577  --------------------------------------------
```

```
                                   930       940       950       960
T_theileri_Tth.54.1380/1-425   RNSYLLKSATKASNIAGLTTSFPQ---KMNERIEEVQK---RRV
T_theileri_Tth.107.1000/1-244  --------------------------------------------
T_theileri_Tth.54.1340/1-680   RRSLHVQAAVAASD-SSECTDKKQVETATGSIMCIVQA---PMF
T_theileri_Tth.117.1040/1-195  --------------------------------------------
T_theileri_Tth.6.5060/1-554    -------------------------------------------A
T_theileri_Tth.1.6360/1-511    --------------------------------------------
T_theileri_Tth.46.1110/1-516   --------------------------------------------
T_theileri_Tth.33.1800/1-520   --------------------------------------------
T_theileri_Tth.46.1020/1-842   ------------------------------------------AS
T_theileri_Tth.70.1140/1-882   ------------------------------------------RG
T_theileri_Tth.87.1050/1-130   --------------------------------------------
T_theileri_Tth.144.1010/1-374  ------------------------------------------ED
T_theileri_Tth.39.1000/1-654   --------------------------------------------
T_theileri_Tth.12.1010/1-802   -------------------------------------------N
T_theileri_Tth.11.3500/1-528   --------------------------------------------
T_theileri_Tth.124.1020/1-215  --------------------------------------------
T_theileri_Tth.54.1390/1-443   -RSPYRLGAHTANA-----------DGGSSRS-TFVEK-----L
T_theileri_Tth.26.2480/1-314   --------------------------------------------
T_theileri_Tth.43.1970/1-318   --------------------------------------------
T_theileri_Tth.37.1300/1-102   --------------------------------------------
T_theileri_Tth.11.2410/1-509   --------------------------------------------
T_theileri_Tth.70.1150/1-914   ------------------------------------------FT
T_theileri_Tth.31.1050/1-313   --------------------------------------------
T_theileri_Tth.70.1010/1-670   ------------------------------------------PT
T_theileri_Tth.36.2090/1-806   RRSLYLQATVAEST--NTCDKGKPVETATGK-QCIVET---MQV
T_theileri_Tth.117.1050/1-116  --------------------------------------------
T_theileri_Tth.11.3480/1-173   --------------------------------------------
T_theileri_Tth.31.1070/1-736   ------------------------------------------PT
T_theileri_Tth.19.2140/1-590   --------------------------------------------
T_theileri_Tth.10.2870/1-312   --------------------------------------------
T_theileri_Tth.46.1010/1-442   ------------------------------------------PG
T_theileri_Tth.12.1820/1-197   --------------------------------------------
T_theileri_Tth.2.2110/1-566    --------------------------------------------
T_theileri_Tth.70.1050/1-869   ------------------------------------------AT
T_theileri_Tth.71.1110/1-97    --------------------------------------------
T_theileri_Tth.4.4270/1-365    --------------------------------------------
T_theileri_Tth.70.1120/1-459   --------------------------------------------
T_theileri_Tth.36.1940/1-208   --------------------------------------------
T_theileri_Tth.14.1390/1-407   --------------------------------------------
T_theileri_Tth.24.2340/1-572   ----------------------------------------EQEE
T_theileri_Tth.101.1060/1-233  --------------------------------------------
T_theileri_Tth.9.3510/1-513    --------------------------------------------
T_theileri_Tth.33.1860/1-659   --------------------------------------------
T_theileri_Tth.134.1010/1-723  -------------------------------------------G
T_theileri_Tth.11.3620/1-114   --------------------------------------------
T_theileri_Tth.144.1000/1-229  --------------------------------------------
T_theileri_Tth.24.1780/1-518   --------------------------------------------
T_theileri_Tth.12.1030/1-678   -------------------------------------------G
T_theileri_Tth.129.1010/1-165  --------------------------------------------
T_theileri_Tth.2.1510/1-887    --------------------------------------------
T_theileri_Tth.31.1030/1-517   ------------------------------------------LS
T_theileri_Tth.36.2110/1-825   RRLLYPQTAMMASEPSIECATENQVKTAKGTTECIVETIQVP--
T_theileri_Tth.151.1000/1-123  --------------------------------------------
T_theileri_Tth.136.1000/1-654  --------------------------------------------
T_theileri_Tth.14.1410/1-752   --------------------------------------------
T_theileri_Tth.46.1740/1-120   --------------------------------------------
T_theileri_Tth.31.1020/1-118   --------------------------------------------
T_theileri_Tth.124.1030/1-577  --------------------------------------------
```

```
                                      970       980       990       1000      1010
T_theileri_Tth.54.1380/1-425    PPRYIITLPSSHWSRRIAKDE--LMVGLVGA--------GYYTA
T_theileri_Tth.107.1000/1-244   --------------------------------------------
T_theileri_Tth.54.1340/1-680    IPKHKGKMTRSHWSRRIAKDE--LMADLVGA--------GYYTA
T_theileri_Tth.117.1040/1-195   --------------------------------------------
T_theileri_Tth.6.5060/1-554     TSF------SSLWERRIAKDE--LMSHYTGE-----PTGMFYTN
T_theileri_Tth.1.6360/1-511     --------VSSHWDHRIAKDE--LMSSYTVF------GAGYYTA
T_theileri_Tth.46.1110/1-516    ----AQHYYYPHWDRRIAKDE--LMSPYSGS-----SNGMFYTA
T_theileri_Tth.33.1800/1-520    ----VVGGVESHWKRRNAKDE--LMTAPFSD------GAMLYTA
T_theileri_Tth.46.1020/1-842    QTN------PPHWKGLIAKDE--LMSPYTSEPGVKDVNGAYYTS
T_theileri_Tth.70.1140/1-882    EAD------PLHWKRLIAKDE--LMSTYSSS-----SNGMFYTA
T_theileri_Tth.87.1050/1-130    --------------------------------------------
T_theileri_Tth.144.1010/1-374   SDL------PSHWERLIAKDE--LMSTYFGE-----PSGMYYSA
T_theileri_Tth.39.1000/1-654    ----AEGNITSHWNRSNMKDE--LMSMVSKV--GHIEGIGYYTA
T_theileri_Tth.12.1010/1-802    AWP-------SHWGSRIAKDE--LMSTYIGE-----PTGMYYTA
T_theileri_Tth.11.3500/1-528    ----NGDEVQSHWTQRNAKDE--LMAPTDAF------GAGYYTA
T_theileri_Tth.124.1020/1-215   --------------------------------------------
T_theileri_Tth.54.1390/1-443    KSSYSNIPSFSHWKRRNAKDE--LMVGFVGA--------GYYTA
T_theileri_Tth.26.2480/1-314    ----MAVGVSSHWDGYIAKDE--LMSPYTTG----EPSGMFYTI
T_theileri_Tth.43.1970/1-318    -----------------------LYSSYHKK---EKVLYRHTPS
T_theileri_Tth.37.1300/1-102    --------------------------------------------
T_theileri_Tth.11.2410/1-509    ERKVFRMDVSSTWSSRNAKDE--LMSSLHAF------GGAYYTV
T_theileri_Tth.70.1150/1-914    PGA------PLHWERRIAKDE--LMSTYSGS-----SNGMFYTA
T_theileri_Tth.31.1050/1-313    ------------------------MSTYSEE-----PSGMYYTS
T_theileri_Tth.70.1010/1-670    EEK------PPHWKRRIAKDE--LMSPYTSDLDAKDVTGAYYTA
T_theileri_Tth.36.2090/1-806    PPNGDKTITRSHWSRRIAKDE--LMVGLVGA--------GYYTA
T_theileri_Tth.117.1050/1-116   -------------TQRGAKVQYFVLSEEAKS-------------
T_theileri_Tth.11.3480/1-173    --------------------------------------------
T_theileri_Tth.31.1070/1-736    KDA------PPHWKRLIAKDE--LMSPYTPD--DKYVTGAYYTA
T_theileri_Tth.19.2140/1-590    ---GEWQTSLSHWERRSAKDE--LMTGITGI--------GYYTA
T_theileri_Tth.10.2870/1-312    --------------------------------------------
T_theileri_Tth.46.1010/1-442    KDA------PLHWERRIAKDE--LMSTYSPD---LDVTGAYYTA
T_theileri_Tth.12.1820/1-197    -PPETGRNDDPHFDGRVARDE--LMAPLHGV----TGDKRSYSS
T_theileri_Tth.2.2110/1-566     ---GSDGTAHSHWERRNVKDE--LMAGLSGV--------SYYTA
T_theileri_Tth.70.1050/1-869    DGA------PLHWERRIAKDE--LMSTYSSS-----SNGMFYTA
T_theileri_Tth.71.1110/1-97     --------------------------------------------
T_theileri_Tth.4.4270/1-365     -----GNSALSHFEMRNVRGE--LMSPKAPG-------GGSYSA
T_theileri_Tth.70.1120/1-459    ----AQHYYYPHFDRRIAKDE--LMSPYSGS-----SNGMFYTA
T_theileri_Tth.36.1940/1-208    --------------------------------------------
T_theileri_Tth.14.1390/1-407    -----GWGSASHFSIRVAKGD--MMGPGYSP--------LVYSE
T_theileri_Tth.24.2340/1-572    GEDQEEDEVSSHWSVRLARDE--LMAPPTGL------TAGYYTA
T_theileri_Tth.101.1060/1-233   --------------------------------------------
T_theileri_Tth.9.3510/1-513     ----NNGEYKLHWERRIAKDE--LMSPYTGE-----PSGMFYTN
T_theileri_Tth.33.1860/1-659    --KWEKGKVSSHWNYRVAKDD--LMSSSIAE------SAMYYSA
T_theileri_Tth.134.1010/1-723   ESL------ELEWERRIARDE--LMGAYTGD-----PTGMFYTN
T_theileri_Tth.11.3620/1-114    --------------------------------------------
T_theileri_Tth.144.1000/1-229   ----------IKWKPWKSQML--IQATTSLMYLELQ--------
T_theileri_Tth.24.1780/1-518    -----RGHTQYHWAKRIAKDE--LMALSYFD------NGGYYTA
T_theileri_Tth.12.1030/1-678    NTY------SGYWERRIARDE--LMSPYTGE-----PTGMYYTN
T_theileri_Tth.129.1010/1-165   --------------------------------------------
T_theileri_Tth.2.1510/1-887     ----SNTVPSSHWNRRIAKDE--LMSTYGDE-----SSGMFYTA
T_theileri_Tth.31.1030/1-517    EGA------PLHWERRIAKEE--LMSTYTLD---FDVTGAYYTA
T_theileri_Tth.36.2110/1-825    ---RDNMGYSSHWSRRIAKDE--LMVGLVGA--------GYYTA
T_theileri_Tth.151.1000/1-123   --------------------------------------------
T_theileri_Tth.136.1000/1-654   ----AEGNITSHWNRSNMKDE--LMSMVSKV--GHIEGIGYYTA
T_theileri_Tth.14.1410/1-752    ---SSCGTARSHLEFRNAKDE--MMAPSSKA--------SYYTK
T_theileri_Tth.46.1740/1-120    ----------NH--------------------------------
T_theileri_Tth.31.1020/1-118    --------------------------------------------
T_theileri_Tth.124.1030/1-577   ----EEGGVQPIWLRRIAKDE--LMALPTDE-----HSAGYYTA
```

```
                                    1020      1030      1040      1050
T_theileri_Tth.54.1380/1-425   ITMGAFADLGY---------------------------------
T_theileri_Tth.107.1000/1-244  --------------------------------------------
T_theileri_Tth.54.1340/1-680   LTLGAFADLGYYKVNWTMEEQMSWGNNSKCEFLNNKCVNSGE--
T_theileri_Tth.117.1040/1-195  --------------------------------------------
T_theileri_Tth.6.5060/1-554    LTLAVFHSMTFYRANFSMAEPMSWGKQSECGLFKS-TCGDVE-K
T_theileri_Tth.1.6360/1-511    LTMSVFDDLPYYSANWGMEEPMAWGNNSGCEFINGACTVNTENT
T_theileri_Tth.46.1110/1-516   LTLAVFESTGHYKANFSRAENMSWGRNAGCDFLDKKCRDDNMS-
T_theileri_Tth.33.1800/1-520   ITMSTFEDMGVYKANWGMEESMFWGNKSGCDLLNNKCM--DNNI
T_theileri_Tth.46.1020/1-842   LTLAVFDSMPFYKADFNMAESMSWGKNVGCDFLKGEEQMKSGII
T_theileri_Tth.70.1140/1-882   LTLSVFDSMPFYKANFKMAENMSWGKDAGCDFLKGGNKEEYKQT
T_theileri_Tth.87.1050/1-130   --------------------------------------------
T_theileri_Tth.144.1010/1-374  LTLAAFEGMKFYQAV---------SLVRMCYLIV----------
T_theileri_Tth.39.1000/1-654   LTIAAFHDMKFYRGNFNMSEKMSWGYLGGCNFGSLTPEGTKTV-
T_theileri_Tth.12.1010/1-802   LTLATFHEMPFYSANFSMAEPMSWGNESLCEFLKGKKKEEML--
T_theileri_Tth.11.3500/1-528   LTMSTFEDMGYYRANWGMEEPMSWGNHSGCDFFEDLCI--VNNA
T_theileri_Tth.124.1020/1-215  --------------------------------------------
T_theileri_Tth.54.1390/1-443   LTLGAFADLGYYRVNWAMAEQMSWGNKAGCEFLNDKCVKEGS--
T_theileri_Tth.26.2480/1-314   LTLSLFEDMKFYRANYSMAETMRWGNHSGCGFLISDCVEKGEG-
T_theileri_Tth.43.1970/1-318   LFLLVMIRMARIGSPFAFL-------------------------
T_theileri_Tth.37.1300/1-102   --------------------------------------------
T_theileri_Tth.11.2410/1-509   LTMAAFHDMKYYSANWGMEEPMSWGNKSGCDFIRKGCL-TENGV
T_theileri_Tth.70.1150/1-914   LTLAVFDSMPFYKAEFKMAENMSWGKDAGCDFLKGNDKEDYKQT
T_theileri_Tth.31.1050/1-313   LTLAIFNDMKFYKANFSMAETMSWGSNAGCEFLTGKCIQENII-
T_theileri_Tth.70.1010/1-670   LTLAVFDSMPFYKANFDMAESMSWGKDAGCDFLKGGKEKKDEII
T_theileri_Tth.36.2090/1-806   ITMGAFADLGYYKVNWTMAEQMSWGNNSKCEFLNDTCMKNGE--
T_theileri_Tth.117.1050/1-116  --------------------------------------------
T_theileri_Tth.11.3480/1-173   --------------------------------------------
T_theileri_Tth.31.1070/1-736   LTLAVFHSMPFYKADFKMAESMSWGKNAGCDFLKGKGKEEYKGT
T_theileri_Tth.19.2140/1-590   LTMAAFEGTNHYKANWGMEETMSWGKNAGCSFHTEKCIERGTT-
T_theileri_Tth.10.2870/1-312   ---------------------MSWGNQSICDLLEV-ESGQKLI-
T_theileri_Tth.46.1010/1-442   LTLAVFHSMPFYRANFEMAENMSWGKVAGCDFLEGKDKDIIITE
T_theileri_Tth.12.1820/1-197   LTLAAFESTGYYKADYNKAEKF-WGSKMGCKFLKKP--------
T_theileri_Tth.2.2110/1-566    LTMAAFEDLGYYRANWGMEEQMSWGKDAGCEFLYEKCV--KNNN
T_theileri_Tth.70.1050/1-869   LTLAVFDSMPFYKAEFKMAESMSWGKDAGCDFLKDGKEKKDEII
T_theileri_Tth.71.1110/1-97    --------------------------------------------
T_theileri_Tth.4.4270/1-365    LTLAVFDDMPFYKAN-----------------------------
T_theileri_Tth.70.1120/1-459   LTLAAFESTGHYKAVFSKAENMSWGRNAGCDFLKKKCRDDELS-
T_theileri_Tth.36.1940/1-208   --------------------------------------------
T_theileri_Tth.14.1390/1-407   LSLAVFDSMPFYKANFDKAEPLVWGKNAGCDFLEKKCL--ENGV
T_theileri_Tth.24.2340/1-572   LTMSTFEGLGYYKANWGMEEPMSWGHKGGCDFINGSCM--TNGV
T_theileri_Tth.101.1060/1-233  --------------------------------------------
T_theileri_Tth.9.3510/1-513    LTLAAFHSMPFYSANFNMAEPMSWGKQTGCDLLHNTCTGSREEL
T_theileri_Tth.33.1860/1-659   LTMAVFDDLPYYSANWGMEESMIWGNNSGCDLLENNFN--DTFV
T_theileri_Tth.134.1010/1-723  LTLTLFNALGFYRAEFSMAEPMSWGKDFGCELFES-TCQEIIEK
T_theileri_Tth.11.3620/1-114   --------------------------------------------
T_theileri_Tth.144.1000/1-229  --------------------------------------------
T_theileri_Tth.24.1780/1-518   LTMALFEDLQYYKANWGMEEQMSWGNQSGCDFLEKKCM--ENNI
T_theileri_Tth.12.1030/1-678   LTLAAFHAMPYYQANFSMAEPMSWGNQSTCDFITK-KCTEII-Q
T_theileri_Tth.129.1010/1-165  -----------------MAEPMSWGKQTGCGLLHNTCTGGNDEL
T_theileri_Tth.2.1510/1-887    LTLATFHDMKFYQANFSMAESMSWGKGGKCLVFRKYEEEKLKK-
T_theileri_Tth.31.1030/1-517   LTLAVFDSMPFYSANFTMAESMNWGKNAGCDFLEGKEKDEIIKK
T_theileri_Tth.36.2110/1-825   ITMGAFADLGHYKVNWKMAEQMSWGNNSGCEFLNNKCVNSGE--
T_theileri_Tth.151.1000/1-123  --------------------------------------------
T_theileri_Tth.136.1000/1-654  LTIAAFEDLEFYRGVFEKAENMSWGYHANCTFLAATCKEKDKQ-
T_theileri_Tth.14.1410/1-752   LTLGIFESMPFYKVNYSMAEPMKWGNNSGCGFLEKKCLTGGET-
T_theileri_Tth.46.1740/1-120   --------------------------------------------
T_theileri_Tth.31.1020/1-118   --------------------------------------------
T_theileri_Tth.124.1030/1-577  LTMALFEDLGYYRANWGMEEQMSWGNQSGCAFLEEDCG--KKHN
```

	1060 1070 1080 1090
T_theileri_Tth.54.1380/1-425	--
T_theileri_Tth.107.1000/1-244	--
T_theileri_Tth.54.1340/1-680	----T------NFPNMFCTTKSGDIPDTPAGLQCTSDRQSLGRC
T_theileri_Tth.117.1040/1-195	--
T_theileri_Tth.6.5060/1-554	H--LG------KFPNTLCKESE-----GESTLQCTSDRFGLGVC
T_theileri_Tth.1.6360/1-511	K----------TTTKMFCS--------RGVYSQCTSDRFAVGSC
T_theileri_Tth.46.1110/1-516	-----------KYSGMFCNLKS------TEELQCTSDRFALGKC
T_theileri_Tth.33.1800/1-520	T----------NYPDMFCN--------TTSSTGCISGRNGVGAC
T_theileri_Tth.46.1020/1-842	D----------NNTEMFCKEVK-------SSLQCTSDRFSLGMC
T_theileri_Tth.70.1140/1-882	K--SS------KYSEMFCDEVN-------PALQCTSDRFSLGMC
T_theileri_Tth.87.1050/1-130	--
T_theileri_Tth.144.1010/1-374	--
T_theileri_Tth.39.1000/1-654	-----------LAPDGYCI--------EAREKSCSSDRLGVINS
T_theileri_Tth.12.1010/1-802	----N------SHPNMFCKKEE-----KKEILQCTSDRFALGMC
T_theileri_Tth.11.3500/1-528	A----------KYPEMFCG---------KSVSRCTTGRSSPGYC
T_theileri_Tth.124.1020/1-215	--
T_theileri_Tth.54.1390/1-443	----T------KFPDMFCTTESTD---GTDSLQCTSDRQSMGRC
T_theileri_Tth.26.2480/1-314	-----------KHSDVFCDSSG---ATRTTTLQCTSDRFALGHC
T_theileri_Tth.43.1970/1-318	--
T_theileri_Tth.37.1300/1-102	--
T_theileri_Tth.11.2410/1-509	S----------NYPNAFCT---------DERLRCSSDRFGLAKC
T_theileri_Tth.70.1150/1-914	K--PN------KYSEMFCDEVN-------PALQCTSDRFSLGMC
T_theileri_Tth.31.1050/1-313	-----------KYPNTFCT-------ESRASLQCTSDRLSLGRC
T_theileri_Tth.70.1010/1-670	Q----------NQPKMFCNEVK-------PTLQCTSDRFALGMC
T_theileri_Tth.36.2090/1-806	----T------DFPNMFCTAKSGD---IPESLQCTSDRQSLGRC
T_theileri_Tth.117.1050/1-116	--
T_theileri_Tth.11.3480/1-173	--
T_theileri_Tth.31.1070/1-736	K--PT------KYAEMFCDEVN-------PALQCTSDRFALGMC
T_theileri_Tth.19.2140/1-590	-----------KFPDFFCT-------APSKTPVCTSDRRALGEC
T_theileri_Tth.10.2870/1-312	-----------DYFALFWKGND------KVILQCTSDRFALGIR
T_theileri_Tth.46.1010/1-442	RPIPE------KYSEMFCNEME-------AVLECTSDRFALGRC
T_theileri_Tth.12.1820/1-197	--
T_theileri_Tth.2.2110/1-566	T----------RYPDMFCT-------KKFKGPRCTSDRLALGNC
T_theileri_Tth.70.1050/1-869	Q----------NKPKMFCDEVK-------PTLQCSSDRLSLGMC
T_theileri_Tth.71.1110/1-97	--
T_theileri_Tth.4.4270/1-365	--
T_theileri_Tth.70.1120/1-459	-----------KYKDMFCDIKS------TEPLQCTSDRFALGKC
T_theileri_Tth.36.1940/1-208	---------------MFCTTESTD---GTDSLQCTSDRQSMGRC
T_theileri_Tth.14.1390/1-407	S----------PNPEIFCN----RSRSSDMYFCTPDRMGLGYC
T_theileri_Tth.24.2340/1-572	T----------AFPKTFCN--------DTKFRCTSDRHAIGRC
T_theileri_Tth.101.1060/1-233	--
T_theileri_Tth.9.3510/1-513	M----------KQTSMFCDKNG-------PVLQCTSDRFALGMC
T_theileri_Tth.33.1860/1-659	D----------EYPNMFCK------LLGGPYFCTNNQLGFGNC
T_theileri_Tth.134.1010/1-723	H--SG------KFPKTFCKES------DKPVLQCTSDRFGLGVC
T_theileri_Tth.11.3620/1-114	--
T_theileri_Tth.144.1000/1-229	--
T_theileri_Tth.24.1780/1-518	T----------RYPQYFCN------------------------
T_theileri_Tth.12.1030/1-678	D---S------KSSNVFCEES------DKPVLQCTSDRFGLGVC
T_theileri_Tth.129.1010/1-165	M----------NHTSMFCKENE-------PVLQCTSDRFALGMC
T_theileri_Tth.2.1510/1-887	-----------SHSKILCKEEP-------NMLQCTSDRFGLGIC
T_theileri_Tth.31.1030/1-517	KIIPE------KYREMFCNKIK-------WGLECTSDRFALGRC
T_theileri_Tth.36.2110/1-825	----T------DFPNMFCTAKSGD---IPESLQCTSDRQSMGSC
T_theileri_Tth.151.1000/1-123	--
T_theileri_Tth.136.1000/1-654	-----------IEAGDSCS--------VYRAGSCSSDRLGLTKC
T_theileri_Tth.14.1410/1-752	-----------DYPDMFCN----QHRKQVPFLCTHDFLALGYC
T_theileri_Tth.46.1740/1-120	--
T_theileri_Tth.31.1020/1-118	--
T_theileri_Tth.124.1030/1-577	D----------FINDVFDG---------KTVFRCTSDRTAYGTA

```
                                                            1110      1120      1130      1140
T_theileri_Tth.54.1380/1-425   --------------------------------------------
T_theileri_Tth.107.1000/1-244  --------------------------------------------
T_theileri_Tth.54.1340/1-680   SSIGAKTV-E--DLPKQF-QYFSEDTKRVGSDSS-EHMDYCPFI
T_theileri_Tth.117.1040/1-195  --------------------------------------------
T_theileri_Tth.6.5060/1-554    SKDN-N---TR-GMPAEY-KFFET--V--G-EQS-EMIKECPII
T_theileri_Tth.1.6360/1-511    TSVMGYRVY--------------------------TEPDECPFA
T_theileri_Tth.46.1110/1-516   SKTWKSD-----ALKWQHCLF-----NERDGPKMAELDDECPII
T_theileri_Tth.33.1800/1-520   AIDSEYDE----ALPVQY-QYFTS--DRTGGPSY-LKMDYCPVM
T_theileri_Tth.46.1020/1-842   SKDPGKGV----QIPDEYFSLFTP--ILKDTDD--DLMDGYPII
T_theileri_Tth.70.1140/1-882   SMKPSGV-----EIPDDYAMYFGT--VLYNSDN--DLMDGYHII
T_theileri_Tth.87.1050/1-130   --------------------------------------------
T_theileri_Tth.144.1010/1-374  --------------------------------------------
T_theileri_Tth.39.1000/1-654   VASK-------------------------------AGESICSIL
T_theileri_Tth.12.1010/1-802   LTKKSS---LD-VPPAGY-QYFS----DISVEWDGDLMDGYPII
T_theileri_Tth.11.3500/1-528   SVPASYHWE--------------------------SEKERCPYT
T_theileri_Tth.124.1020/1-215  --------------------------------------------
T_theileri_Tth.54.1390/1-443   G---IQTY-ER-DLEGQF-QYFSD--AMTGGERA-SQMDYCPFI
T_theileri_Tth.26.2480/1-314   TRSLSSQP------------------ISVFSADHREEKRECAVV
T_theileri_Tth.43.1970/1-318   --------------------------------------------
T_theileri_Tth.37.1300/1-102   --------------------------------------------
T_theileri_Tth.11.2410/1-509   VDVDEGTVNA-------------------------DKTGSCPVL
T_theileri_Tth.70.1150/1-914   SMKPYGV-----KIPLDYEMYFPP--VLNDPDD--DLMDGYTII
T_theileri_Tth.31.1050/1-313   TSSNFPQ-----DYPEDY-RNFRN--NTFTYLYG-ELTDGCPII
T_theileri_Tth.70.1010/1-670   SAKEQPTI----HIPYDYLEQYYL--IANNPDN--DLMDGYSII
T_theileri_Tth.36.2090/1-806   S---IQPY-ER-NIPQQF-QYFSEE-KKVGSAPS-EQMDYCPFI
T_theileri_Tth.117.1050/1-116  --------------------------------------------
T_theileri_Tth.11.3480/1-173   --------------------------------------------
T_theileri_Tth.31.1070/1-736   SKKPSGV-----NIPLDYVEHFNT--VLKDTDD--DLMDGYSII
T_theileri_Tth.19.2140/1-590   VLFKYNF-----HL--RY-QYFED--KRVGGRLG-DMTDLCPII
T_theileri_Tth.10.2870/1-312   SKTDLN------DSPEGY-EYVQN----GTKEAN-DLMDNVSFI
T_theileri_Tth.46.1010/1-442   TKLNGIEAAK--KYSPVY-LQFSPETDTTDSKN--DLTDGYPII
T_theileri_Tth.12.1820/1-197   --------------------------------------------
T_theileri_Tth.2.2110/1-566    DLESFGR-----TLPLQY-QYFTE--TSIAGGGR-QLTDHCPVI
T_theileri_Tth.70.1050/1-869   SMGTSDVV----EIPFEYQKYFGT--VLYNSDN--DLMDGYPII
T_theileri_Tth.71.1110/1-97    --------------------------------------------
T_theileri_Tth.4.4270/1-365    -----------------------------------SLVRMCYLI
T_theileri_Tth.70.1120/1-459   SKTWKSD-----ALKWQHCLF-----NQRDGPKMAELDDECPII
T_theileri_Tth.36.1940/1-208   G---IQTY-ER-DLEGQF-QYFSD--AMTGGERA-SQMDYCPFI
T_theileri_Tth.14.1390/1-407   TLKTYYR-----ALPEPF-QYFKD--KRMGGDL--RALDYCPYV
T_theileri_Tth.24.2340/1-572   ELSKDVESK--------------------------VFLDQCPTM
T_theileri_Tth.101.1060/1-233  --------------------------------------------
T_theileri_Tth.9.3510/1-513    SSTPVPD-----TLSTVY-HYFDK---IASAGKD-EMTNGCPII
T_theileri_Tth.33.1860/1-659   DYQFHYYDSD-------------------------GEVNFAPIL
T_theileri_Tth.134.1010/1-723  SKDNTN---KH-GIPAEY-HYFQK--N--NLRNS-EMMEGCPII
T_theileri_Tth.11.3620/1-114   --------------------------------------------
T_theileri_Tth.144.1000/1-229  --------------------------------------------
T_theileri_Tth.24.1780/1-518   --------------------------------------------
T_theileri_Tth.12.1030/1-678   SKVDTK---KD-GIPTEY-KFFEI--V--G-EQS-DMMKGCPII
T_theileri_Tth.129.1010/1-165  SSKSLPG-----TLPEVY-SYFTK-----TAGQN-EMTNGCPII
T_theileri_Tth.2.1510/1-887    ARTNFSHAVHGDRISTP---------DDYPKETK-WYKPYWPVV
T_theileri_Tth.31.1030/1-517   TKEMDLTDRQ--FISSEY-AFFND--LTSSASGMYDLTDGYPII
T_theileri_Tth.36.2110/1-825   SSIGANPV-KN-DLPVGF-RYFSEK-KKVGSLPL-EQMDYCPFI
T_theileri_Tth.151.1000/1-123  --------------------------------------------
T_theileri_Tth.136.1000/1-654  IPPK-------------------------------EGESICPIF
T_theileri_Tth.14.1410/1-752   TMTWYST-----PLPDEY-QYFLG--KHFGGTI--REMDYCPFV
T_theileri_Tth.46.1740/1-120   --------------------------------------------
T_theileri_Tth.31.1020/1-118   ----------------------------RDGPKMAELDDVCPII
T_theileri_Tth.124.1030/1-577  ALVDKGFQA--------------------------EYQRICKVN
```

```
                                  1150      1160      1170      1180
T_theileri_Tth.54.1380/1-425    --------------------------------------------
T_theileri_Tth.107.1000/1-244   --------------------------------------------
T_theileri_Tth.54.1340/1-680    KAL-----P---------QKSC----INGEQ-SQ--MPESVIA-
T_theileri_Tth.117.1040/1-195   ---------------------C----TDGSR-WT--IIGSFVG-
T_theileri_Tth.6.5060/1-554     KPF-----Q---------QTMC----EKGNE-TL--MPGSIVD-
T_theileri_Tth.1.6360/1-511     KPE-----HIYTPHGML-SALC----TLTVN-NT--IPGSVTG-
T_theileri_Tth.46.1110/1-516    KPL-----V---------RTSC----ETGNA-DI--MPGSIVS-
T_theileri_Tth.33.1800/1-520    TLAT--------------GILC----VNEEE-NE--DGGFLFG-
T_theileri_Tth.46.1020/1-842    KPF-----I---------TTSC----EEGTV-EY--MPGSIVS-
T_theileri_Tth.70.1140/1-882    KPI-----P---------KTNC----EEDEF-KL--IPGSILS-
T_theileri_Tth.87.1050/1-130    KATA--------------ATVC----ENGSE-AH--MPGSLLG-
T_theileri_Tth.144.1010/1-374   --------------------------------------------
T_theileri_Tth.39.1000/1-654    VPD-----E---------RFFC----TSEG--DIPGRDMGLFR-
T_theileri_Tth.12.1010/1-802    KPL-----I---------GTAC----EDGNE-TL--LSGSLFG-
T_theileri_Tth.11.3500/1-528    KPY------FGNFEEKYRYAFC----FSDDV-PA--LDGSLMG-
T_theileri_Tth.124.1020/1-215   ------------------IAFC----FSEGI-PA--LGGSLIG-
T_theileri_Tth.54.1390/1-443    TAE-----S---------GFSC----INGEQSDN--MPGSLIA-
T_theileri_Tth.26.2480/1-314    KATA--------------ATVC----ENGSE-AH--MPGSRIG-
T_theileri_Tth.43.1970/1-318    --------------------------------------------
T_theileri_Tth.37.1300/1-102    --------------------------------------------
T_theileri_Tth.11.2410/1-509    VSPL----SVREDASLF-SFAC----TELGD-AD--FPGFLTG-
T_theileri_Tth.70.1150/1-914    KPI-----P---------KTNC----EEDKF-NL--IPGSILS-
T_theileri_Tth.31.1050/1-313    RQL-----E---------ETTC----QSGDL-EL--MPGSILG-
T_theileri_Tth.70.1010/1-670    KPI-----I---------MTSC----EEGKV-EF--MPGSIVS-
T_theileri_Tth.36.2090/1-806    KAL-----P---------QKSC----ISGEQ-NQ--MPGSKIG-
T_theileri_Tth.117.1050/1-116   --------------------------------QRRFSSGRTVG-
T_theileri_Tth.11.3480/1-173    --------------------------------------------
T_theileri_Tth.31.1070/1-736    KPT-----L---------QTSC----EGHQF-NL--MPGSVLG-
T_theileri_Tth.19.2140/1-590    SPI-----V---------DGDC----NIDIA-NA--FPGSRTG-
T_theileri_Tth.10.2870/1-312    RPL-----K---------GTAC----EDGNE-SL--MPGSLFG-
T_theileri_Tth.46.1010/1-442    KPI-----H---------MTSC----ENGNV-EY--MPGSVVG-
T_theileri_Tth.12.1820/1-197    --------------------------------------------
T_theileri_Tth.2.2110/1-566     KPA-----V---------RGVC----TDVE--NY--WPGSRVG-
T_theileri_Tth.70.1050/1-869    KAI-----P---------QTSC----ERDQL-DL--MPGSDLG-
T_theileri_Tth.71.1110/1-97     --------------------------------------------
T_theileri_Tth.4.4270/1-365     V-------------------------------------------
T_theileri_Tth.70.1120/1-459    KPL-----V---------RTSC----ESGDV-TV--MPGSIVS-
T_theileri_Tth.36.1940/1-208    TAE-----S---------GFSC----TDGDQ-SQ--MPGSLIA-
T_theileri_Tth.14.1390/1-407    EAYS--------------DTTC----TSGFH-LQ--SRGSFIG-
T_theileri_Tth.24.2340/1-572    EPIWKLEGEEEEEEESSPTSLC----TDGEE-GL--FPGSLTG-
T_theileri_Tth.101.1060/1-233   --------------------------------------------
T_theileri_Tth.9.3510/1-513     KPL-----K---------ETTC----ESGNV-EL--MPGSIVS-
T_theileri_Tth.33.1860/1-659    SI------HMYTLHNFRISTMC----NDQPR-VK--VTPSIFA-
T_theileri_Tth.134.1010/1-723   KPF-----Q---------QTMC----ETGDE-TL--MPGSIVS-
T_theileri_Tth.11.3620/1-114    --------------------------------------------
T_theileri_Tth.144.1000/1-229   --------------------------------------------
T_theileri_Tth.24.1780/1-518    --------------------------------------------
T_theileri_Tth.12.1030/1-678    KPF-----Q---------ETMC----ENGNE-TL--MPGSIVD-
T_theileri_Tth.129.1010/1-165   KPL-----K---------ETTC----ENGDV-AL--MPGSIVS-
T_theileri_Tth.2.1510/1-887     QTS-----P---------TTAC----EGGNE-EL--MPGSRLG-
T_theileri_Tth.31.1030/1-517    KPI-----Y---------TTSC----ESGNL-SL--MPGSIVG-
T_theileri_Tth.36.2110/1-825    MAK-----P---------GYSC----ISGEESNN--VPGSKIA-
T_theileri_Tth.151.1000/1-123   --------------------------------------------
T_theileri_Tth.136.1000/1-654   ESD-----Q---------SFFC----STEE--DVPDRGLGLVG-
T_theileri_Tth.14.1410/1-752    QELK--------------DSSC----TTSTT-IQ--RRGSFVG-
T_theileri_Tth.46.1740/1-120    --------------------------------------------
T_theileri_Tth.31.1020/1-118    KPL-----V---------HTSC----ETGNA-KD--MPGSILS-
T_theileri_Tth.124.1030/1-577   EN------YITHEIAQN-PSYC----TDESDTST--ATGSLKG-
```

```
                                    1190      1200      1210      1220      1230
T_theileri_Tth.54.1380/1-425   --------------------------------------------
T_theileri_Tth.107.1000/1-244  --------------------------------------------
T_theileri_Tth.54.1340/1-680   NNSRCVEGGDL-KTIK---------DEAA----VGAVCVEVSCK
T_theileri_Tth.117.1040/1-195  PNARCVKGNEL-KYRTRS---------------IGDVCVNTKCE
T_theileri_Tth.6.5060/1-554    KMSRCLNVEEL-QFND---GNN--VTGLK----VQGICAKVKCE
T_theileri_Tth.1.6360/1-511    NDSWCLDGDSL-KVKVG-------NANKV----VGGVCVRVSCD
T_theileri_Tth.46.1110/1-516   NTSRCLSRVEF-VSDSEVRS-------------IGDICAQVKCD
T_theileri_Tth.33.1800/1-520   PDSWCLEGSSL-ILEYG-------GSNAL----VGGVCVAVKCH
T_theileri_Tth.46.1020/1-842   NISRCLKGVGL-QLRE---QNVKKKHLL----VGDICADVKCD
T_theileri_Tth.70.1140/1-882   NISRCLKGENL-KLKEP-------DGKPP----VGDICAKVKCE
T_theileri_Tth.87.1050/1-130   NTSRCLKGDAL-RLRD--------VATIDIHSIIGDICANVKCE
T_theileri_Tth.144.1010/1-374  --------------------------------------------
T_theileri_Tth.39.1000/1-654   NDSWCLDVDYF-NLTIEEEKRM-----------KHGMCAAVECT
T_theileri_Tth.12.1010/1-802   RDSRCLKADGL-KLKD----SN--GTPLN----IAGVCAKVKCD
T_theileri_Tth.11.3500/1-528   NDSWCLDGESL-HVNFD---------DGSVKDDVAGVCALVSCN
T_theileri_Tth.124.1020/1-215  KDSWCLDGESL-HVNLD-------DVNVR--YNVAAVCALVSCD
T_theileri_Tth.54.1390/1-443   ANSRCVQGENL-IAD-----------DTA----VGAVCVEVSCK
T_theileri_Tth.26.2480/1-314   NTSRCLKGDAL-RLRD--------VSTIDIHSIIGDICANVKCE
T_theileri_Tth.43.1970/1-318   --------------------------------------------
T_theileri_Tth.37.1300/1-102   --------------------------------------------
T_theileri_Tth.11.2410/1-509   ENSMCLTTNEY-DVEKY-------GNTTK----LSGVCAQVLCD
T_theileri_Tth.70.1150/1-914   NISRCLKGDKL-KLKE----PN--ENNLT----VGDICADVKCE
T_theileri_Tth.31.1050/1-313   KESRCLQGDEL-IQKESA------QTFLP----VGDICANVKCE
T_theileri_Tth.70.1010/1-670   NISRCLNGDSL-TLKT----PN--ENDLT----VGDICAEVRCD
T_theileri_Tth.36.2090/1-806   INSRCVEGNEL-RADN----------NAA----VGAVCVEVSCK
T_theileri_Tth.117.1050/1-116  --------------------------------------------
T_theileri_Tth.11.3480/1-173   --------------------------------------------
T_theileri_Tth.31.1070/1-736   KESRCLKGKEL-KLKT----PN--EDNLT----VGDICAKVKCK
T_theileri_Tth.19.2140/1-590   PSSWCLKGESL-VLNGKM---------------VGDVCAEVQCD
T_theileri_Tth.10.2870/1-312   RDSRCLNVKEP-AEFKENKGDT--GSGVK----VYGICAKVKCE
T_theileri_Tth.46.1010/1-442   NMSRCLKG------------------------------------
T_theileri_Tth.12.1820/1-197   --------------------------------------------
T_theileri_Tth.2.2110/1-566    PESRCLHGDSL-HIGNRF---------------VGDVCVEVSCN
T_theileri_Tth.70.1050/1-869   KESRCLKGENL-TLKE----QN--KNNLT----VGDICAKVKCE
T_theileri_Tth.71.1110/1-97    ---------------D--------VSTIDIHSIIGDICANVKCE
T_theileri_Tth.4.4270/1-365    --------------------------------------------
T_theileri_Tth.70.1120/1-459   NISRCLSRVKL-FASNKVRP-------------IGGICA-----
T_theileri_Tth.36.1940/1-208   ANSRCVQGENL-IAD-----------DTA----VGAVCVEVSCK
T_theileri_Tth.14.1390/1-407   ENARCVKGVEL-EISGKS---------------VGDVCVNTKCD
T_theileri_Tth.24.2340/1-572   EDSWCLDAESL-TVVTN---------KRSMRNAIRGVCARVKCS
T_theileri_Tth.101.1060/1-233  --------------------------------------------
T_theileri_Tth.9.3510/1-513    NMSRCLNVEGT-KLTYGT------KNEVT----VKGICAKVKCE
T_theileri_Tth.33.1860/1-659   NDSVCLNTQHY-KGTIESNLGN-----------MYGICARVSCD
T_theileri_Tth.134.1010/1-723  NISRCLDVTEP-VGID----EN--GKKVK----VQGICAKVKCE
T_theileri_Tth.11.3620/1-114   --------------------------------------------
T_theileri_Tth.144.1000/1-229  --------------------------------------------
T_theileri_Tth.24.1780/1-518   ----------------------------ETIXNQNGVCARVLCD
T_theileri_Tth.12.1030/1-678   KMSRCLSVKDP-QIKD----VN--GNDVT----VQGICAKVKCE
T_theileri_Tth.129.1010/1-165  NMSRCLNVNKPLEFSEGVQ-----KNGVT----VKGICAKVKCE
T_theileri_Tth.2.1510/1-887    PQSRCLTTESL-KVKNGT------LSYKS----VQGICANVKCD
T_theileri_Tth.31.1030/1-517   NTSRCLKGDKL-KLNK----GN--KFALS----VGDICADVKCD
T_theileri_Tth.36.2110/1-825   NNSRCVEGNGL-TAN-----------SAA----VGAVCVEVSCK
T_theileri_Tth.151.1000/1-123  --------------------------------------------
T_theileri_Tth.136.1000/1-654  KDSWCFDVDYL-NLKIEENNM------------KHGVCAAVNCS
T_theileri_Tth.14.1410/1-752   PKSRCVKGENL-KFTTQP---------------IGDVCVNTKCE
T_theileri_Tth.46.1740/1-120   --------------------------------------------
T_theileri_Tth.31.1020/1-118   NTSRCLSRVEF-VSDSEVRS-------------IGDICAQVKCD
T_theileri_Tth.124.1030/1-577  PNSWCLDGEEL-EVKD--------TESTF--SNVNGVCALVSCD
```

```
                                   1240      1250      1260      1270
T_theileri_Tth.54.1380/1-425   --------------------------------------------
T_theileri_Tth.107.1000/1-244  --------------------------------------------
T_theileri_Tth.54.1340/1-680   -FKK--VSLRYSG-----NDK--------WYSC-HEGENLT---
T_theileri_Tth.117.1040/1-195  --EEGKLSVQFLG-----DNETT------WYECE-ENKKI----
T_theileri_Tth.6.5060/1-554    -DDKQTVSVQLKG-----QESK----ET-WHVC-ENDGT---T-
T_theileri_Tth.1.6360/1-511    ---EGKVKVMYAG-----NDK--------WHDCP-EGGS-----
T_theileri_Tth.46.1110/1-516   ---GGKVHIRYKG-----NNS--------WHVCDNETENDVISP
T_theileri_Tth.33.1800/1-520   ---DGHVSVRYRG-----STT--------WIDCP-SGETIT---
T_theileri_Tth.46.1020/1-842   ---DNKVHIRYKG-----NDS--------WHVC-NEENSVITPA
T_theileri_Tth.70.1140/1-882   ---NNKVSVQYSG-----SDK--------WHVC-EDGK-----I
T_theileri_Tth.87.1050/1-130   ---GGKVSVQYKG-----DDH--------WYEC-KEGSSISPS-
T_theileri_Tth.144.1010/1-374  --------------------------------------------
T_theileri_Tth.39.1000/1-654   --DNRVVNIKLTV-----DGA--------WHRCREDGF------
T_theileri_Tth.12.1010/1-802   -NEKKKVSVQLKG-----YEEN---KEE-WHDCENDDKA---TV
T_theileri_Tth.11.3500/1-528   -ESSRTVMVKYNG-----SDE--------WHDCP-EGKSIE---
T_theileri_Tth.124.1020/1-215  -ESSRTVKVQYNG-----SDE--------WHNCP-EGES-----
T_theileri_Tth.54.1390/1-443   -FKV--VSVRYSG-----NIE--------WHSC-YEGETLT---
T_theileri_Tth.26.2480/1-314   ---GGKVSVQYKG-----DDH--------WYEC-KEGSSILPS-
T_theileri_Tth.43.1970/1-318   --------------------------------------------
T_theileri_Tth.37.1300/1-102   --------------------------------------------
T_theileri_Tth.11.2410/1-509   -KSNRKVKVKYSG-----ILE--------FQECP-SGSE-----
T_theileri_Tth.70.1150/1-914   -KDTKKILVQYSG-----SEK--------WRVC-EDGKK----I
T_theileri_Tth.31.1050/1-313   ---DGKVKVQYKG-----SDK--------WHEC-PDGGKIESL-
T_theileri_Tth.70.1010/1-670   ---GGKVHIRYEG-----NDN--------WHEC-NDEKS---VI
T_theileri_Tth.36.2090/1-806   -FNK--VIVRYSG-----DEE--------WHSC-HEGENLT---
T_theileri_Tth.117.1050/1-116  --------------------------------------------
T_theileri_Tth.11.3480/1-173   --------------------------------------------
T_theileri_Tth.31.1070/1-736   -KDTNKVLVKYSD-----TTD--------WQEC-EDGGK----I
T_theileri_Tth.19.2140/1-590   ---GKSVQVRYYG-----DDK--------WYPCP-EGKVL----
T_theileri_Tth.10.2870/1-312   --SGNKVSVLLKG-----ENE----KES-WHDC-SDGKK---TF
T_theileri_Tth.46.1010/1-442   --------------------------------------------
T_theileri_Tth.12.1820/1-197   --------------------------------------------
T_theileri_Tth.2.2110/1-566    --VSGILYLRYLG-----EDN--------WYPCP-EGGG-----
T_theileri_Tth.70.1050/1-869   -KDNNTFKVQYKG-----STN--------WHEC-KNGK-----I
T_theileri_Tth.71.1110/1-97    ---GGKVSVQYKG-----DDD--------WYEC-KEGSSISPS-
T_theileri_Tth.4.4270/1-365    --------------------------------------------
T_theileri_Tth.70.1120/1-459   --------------------------------------------
T_theileri_Tth.36.1940/1-208   -FKV--VSVRYSG-----NIE--------WHSC-YEGETLT---
T_theileri_Tth.14.1390/1-407   ---NGILSVQFLGDD---DDE--------WHVCE-EKKEI----
T_theileri_Tth.24.2340/1-572   --SGGGVKVQYKG-----NDK--------WHDCP-EGKSID---
T_theileri_Tth.101.1060/1-233  --------------------------------------------
T_theileri_Tth.9.3510/1-513    ---NDKVSVQYKG-----KEGPND-----WIECEKDGDKITL--
T_theileri_Tth.33.1860/1-659   ---EGKVRVIYDE-----SED--------WQECP-ENTSMD---
T_theileri_Tth.134.1010/1-723  -DGK--VKVHYKG-----YNEE-NGKEK-WEEC-SDEKS---TI
T_theileri_Tth.11.3620/1-114   --------------------------------------------
T_theileri_Tth.144.1000/1-229  --------------------------------------------
T_theileri_Tth.24.1780/1-518   -YEKRTVKVQYKGKED--END--------WYECP-ENGT-----
T_theileri_Tth.12.1030/1-678   -DGK--VHVQYKG-----QKSK----ET-WHVC-ENDGD---TI
T_theileri_Tth.129.1010/1-165  ---NGKVSVQY-------KEDTTE-----WIECSGEN-------
T_theileri_Tth.2.1510/1-887    ---NDTVRVQYKD-----DET--------WHLC-PQGQTIDV--
T_theileri_Tth.31.1030/1-517   -KDNKKVMVRYSG-----SEI--------WHNC-SD--------
T_theileri_Tth.36.2110/1-825   -FKK--VIVRYSG-----NNE--------WHSC-HEGENLT---
T_theileri_Tth.151.1000/1-123  --------------------------------------------
T_theileri_Tth.136.1000/1-654  --DNDVFKVQLKK-----NGE--------WYNCPKEGDEF----
T_theileri_Tth.14.1410/1-752   ---NGKLSVQFRL-----DEE--------WYECE-EKKKV----
T_theileri_Tth.46.1740/1-120   --------------------------------------------
T_theileri_Tth.31.1020/1-118   ---SGKVHIRYKG-----NNS--------WHVCDNETENDVILP
T_theileri_Tth.124.1030/1-577  -AKSKKVSVKYKGN----QDK--------WHDCP-EGES-----
```

```
                                   1280      1290      1300      1310
T_theileri_Tth.54.1380/1-425   --------------------------------------------
T_theileri_Tth.107.1000/1-244  --------------------------------------------
T_theileri_Tth.54.1340/1-680   ----V-----KGDVL--K-GKIV-CPKYADVCNTINKTLDESQG
T_theileri_Tth.117.1040/1-195  ---------TLNNKN--WTGTIT-CPKYADVCTRYPNITNKEPL
T_theileri_Tth.6.5060/1-554    -N--L-----DGSVF--KKGTIIRCPKYEEVCTGLPETEPF-NI
T_theileri_Tth.1.6360/1-511    ----ITPLASTSTSF--VSGKIK-CPKYREVCTMTPDGGSRLIP
T_theileri_Tth.46.1110/1-516   QS--------NDSAL--SSGVIL-CPKYSEVCM-----------
T_theileri_Tth.33.1800/1-520   ---------PSGTGF--VSGSIV-CPNYNEVCVIS---------
T_theileri_Tth.46.1020/1-842   ----------KDSAF--SSGTIQ-CPKYSEVCNEKKKS--TEFT
T_theileri_Tth.70.1140/1-882   EV--T-----DGQEF--QSGSIL-CPNYTEVCNDFSEVKDINFT
T_theileri_Tth.87.1050/1-130   ----------VTSAF--SSGRIV-CSPYSEVCMDLPIEPPPTEA
T_theileri_Tth.144.1010/1-374  ------------------------VPRLST--------------
T_theileri_Tth.39.1000/1-654   ----VTLNKTDFPMY--VNGSIR-CPRYDEVCTMRSDGSSRLTS
T_theileri_Tth.12.1010/1-802   K---L-----DGSVF--SGGTIE-CPKYEEVCTGMPKTNS-LKI
T_theileri_Tth.11.3500/1-528   ---------AKPPTF--KSGHIK-CPKYTEVCTIAPNGSSLLPL
T_theileri_Tth.124.1020/1-215  ----IE---AKPPTF--KSGHIK-CPNYTEVCTIASNGSSLITV
T_theileri_Tth.54.1390/1-443   ----V-----NSGAL--Q-GKIV-CPKYADVCNTINRKVDESQG
T_theileri_Tth.26.2480/1-314   ----------VTSAF--SSGRIV-CPPYSEVCMDLPIEPPPTEA
T_theileri_Tth.43.1970/1-318   --------------------------------------------
T_theileri_Tth.37.1300/1-102   -----------------DGKGVL---------------------
T_theileri_Tth.11.2410/1-509   ----IA---VGSSSASLKFKRIH-CPMYTEVCTVASDGSSLHPL
T_theileri_Tth.70.1150/1-914   EV--T-----GSTEF--ASGSIL-CPNYTEVCNDFPEVKDINFT
T_theileri_Tth.31.1050/1-313   ----------EGTEF--QSGSIL-CPKYAEVCTDLPSISERRIE
T_theileri_Tth.70.1010/1-670   RP--T-----EDSVF--SSGTIK-CPKYSEVCNDFSEVKDINFT
T_theileri_Tth.36.2090/1-806   ----V-----KGDVL--K-GEIV-CPKYADVCNTINKTLDESQG
T_theileri_Tth.117.1050/1-116  -------------------------P------------------
T_theileri_Tth.11.3480/1-173   --------------------------------------------
T_theileri_Tth.31.1070/1-736   DV--K-----DSKEF--ASGSIL-CPNYTEVCNDFPVVKDINFT
T_theileri_Tth.19.2140/1-590   ---------KPNNTF--TSGSIV-CPKYREVCTIAADGSSRIVK
T_theileri_Tth.10.2870/1-312   DV--V-----AGSVF--IGGKIE-CPKYEEVCIGLLEDEPPTDI
T_theileri_Tth.46.1010/1-442   --------------------------------------------
T_theileri_Tth.12.1820/1-197   --------------------------------------------
T_theileri_Tth.2.2110/1-566    ----I----IPKKPF--TKGRIL-CPKYMDVCVTLALNITNKSY
T_theileri_Tth.70.1050/1-869   EV--T-----EGTEF--TGGSIV-CPTYAEVCTELSST--IDFN
T_theileri_Tth.71.1110/1-97    ----------VTSAF--SSGRIV-CPPYSEVCMDLPIEPPTVEA
T_theileri_Tth.4.4270/1-365    ------------------------VPRLST--------------
T_theileri_Tth.70.1120/1-459   --------------------------------------------
T_theileri_Tth.36.1940/1-208   ----V-----NGGAL--Q-GKIV-CPKYADVCNTLNRKVDESQG
T_theileri_Tth.14.1390/1-407   ---------IPNKEN--WSGKII-CPRYDAVCTKLPDITIPLKE
T_theileri_Tth.24.2340/1-572   ---------VRSSAF--TNGKIK-CPKYDEVCTITPDGRSRLSM
T_theileri_Tth.101.1060/1-233  --------------------------------------------
T_theileri_Tth.9.3510/1-513    ----------EGSAF--NGGSIV-CPKYSEVCTGLNETD-----
T_theileri_Tth.33.1860/1-659   ---------VQPSGFS-TTISVQ-CPKYSGVCIIAPNGSSRRPM
T_theileri_Tth.134.1010/1-723  -T--L-----QGSEF--KSGTTIRCPKYEEVCTKETNTDLLPEI
T_theileri_Tth.11.3620/1-114   --------------------------------------------
T_theileri_Tth.144.1000/1-229  --------------------------------------------
T_theileri_Tth.24.1780/1-518   ----IV---VESPAFE-SGGKIK-CPKYDEVCTIAFNGSSHVPF
T_theileri_Tth.12.1030/1-678   -D--L-----QGSVF--SGGTIV-CPKYAEVCTGLPETEPF-NI
T_theileri_Tth.129.1010/1-165  --------------------------------------------
T_theileri_Tth.2.1510/1-887    ----------TGSAF--SGGKIQ-CPKYSEVCSREPSSAGVTVF
T_theileri_Tth.31.1030/1-517   --------------------------------------------
T_theileri_Tth.36.2110/1-825   ----V-----NGSVL--Q-GKIV-CPKYADVCNTINKTLDESQG
T_theileri_Tth.151.1000/1-123  --------------------------------------------
T_theileri_Tth.136.1000/1-654  ----IIPNKTDNLTH--VKGWIR-CPKYYEVCTMKSDGSSRLTS
T_theileri_Tth.14.1410/1-752   ---------SSKNGN--WSGSII-CPAYEEVCTEHRNVTIVLEE
T_theileri_Tth.46.1740/1-120   --------------------------------------------
T_theileri_Tth.31.1020/1-118   QS--------NDSAL--SSGVIL-CPKYSEVCM-----------
T_theileri_Tth.124.1030/1-577  ----IQ---ANSPTFK-SGGKIK-CPKYDEVCTVASNGSSLVPF
```

```
                                     1330      1340      1350      1360
T_theileri_Tth.54.1380/1-425     --------------------------------------------
T_theileri_Tth.107.1000/1-244    --------------------------------------------
T_theileri_Tth.54.1340/1-680     PQKDPDVVEEVQTRQPEVETTPT---------------------
T_theileri_Tth.117.1040/1-195    PEVTDKDNEPAPANITWPIKPKD---------------------
T_theileri_Tth.6.5060/1-554      SYTEVNVSEDVVE-E---PKQEE----------KEK---E----
T_theileri_Tth.1.6360/1-511      KTSEQKQ-------------------------------------
T_theileri_Tth.46.1110/1-516     --------------------------------------------
T_theileri_Tth.33.1800/1-520     --------------------------------------------
T_theileri_Tth.46.1020/1-842     IVYDEGEEEKLKREEEQEKDEMEVEAQEAETDQKKKEQE-----
T_theileri_Tth.70.1140/1-882     IEYDEDEKERIKKEKEQEKNEMEEEEKQRSEAEAEADEQ-----
T_theileri_Tth.87.1050/1-130     VV-MEEEVYNVTASINE---------------------------
T_theileri_Tth.144.1010/1-374    --------------------------------------------
T_theileri_Tth.39.1000/1-654     QVQENTQLIIEPKWVSSTPADPN---------------------
T_theileri_Tth.12.1010/1-802     KYYSGTQDLEAREVVVNDDDEKKEEVVKDPNHNENGETNEEEPP
T_theileri_Tth.11.3500/1-528     VKPPDPTPPGGDNNGG----------------------------
T_theileri_Tth.124.1020/1-215    VDPPTDPTPPPDGDGGGGGDNDG---------------------
T_theileri_Tth.54.1390/1-443     PRTRAAIMGV----------------------------------
T_theileri_Tth.26.2480/1-314     VV--VEEVYNVTASINLINS------------------------
T_theileri_Tth.43.1970/1-318     --------------------------------------------
T_theileri_Tth.37.1300/1-102     --------------------------------------------
T_theileri_Tth.11.2410/1-509     ANAP----------------------------------------
T_theileri_Tth.70.1150/1-914     IEYDEDEKERIKKEEEEKDEMDVETQEAEEETLEEERER-----
T_theileri_Tth.31.1050/1-313     SNEAADTEILQDYVIGRDLHDIS---------------------
T_theileri_Tth.70.1010/1-670     IEYDEDVKLEIKKEK-EAEDKAQEKKQREEEDRKKNEQE-----
T_theileri_Tth.36.2090/1-806     PQKDPDIVEAIQTTLPEGETTSA---------------------
T_theileri_Tth.117.1050/1-116    --------------------------------------------
T_theileri_Tth.11.3480/1-173     --------------------------------------------
T_theileri_Tth.31.1070/1-736     IEYDEDEKERIKKEEKEDEDEMEKEEKQHEEEEENEKTL-----
T_theileri_Tth.19.2140/1-590     KIIPEARHSSDTESSMGPTANLVSGNRGNEGNMTDKKKQGGFIE
T_theileri_Tth.10.2870/1-312     KFYDGNKVTNGYEGDVIIVKEEK---------------------
T_theileri_Tth.46.1010/1-442     --------------------------------------------
T_theileri_Tth.12.1820/1-197     --------------------------------------------
T_theileri_Tth.2.2110/1-566      IFGKPSTQYILSGAEDWHNYING---------------------
T_theileri_Tth.70.1050/1-869     ITYDEGEKRLMDEEDRREAEEEEEIQRIKVEAEAERNQK-----
T_theileri_Tth.71.1110/1-97      VV-VEEEVYNVTASINLINS------------------------
T_theileri_Tth.4.4270/1-365      --------------------------------------------
T_theileri_Tth.70.1120/1-459     --------------------------------------------
T_theileri_Tth.36.1940/1-208     PRTRAAIMGV----------------------------------
T_theileri_Tth.14.1390/1-407     LPYQDGDYKPAPGNITWNKTETP---------------------
T_theileri_Tth.24.2340/1-572     NVELDSTRRAAA--------------------------------
T_theileri_Tth.101.1060/1-233    --------------------------------------------
T_theileri_Tth.9.3510/1-513      ---------LPTIEYDEKNNDNS---------------------
T_theileri_Tth.33.1860/1-659     VYPPGYEETTTAPNTGEEKF------------------------
T_theileri_Tth.134.1010/1-723    KYNVVNVSEDKVE-E---HKQDE----------EEE---E----
T_theileri_Tth.11.3620/1-114     --------------------------------------------
T_theileri_Tth.144.1000/1-229    --------------------------------------------
T_theileri_Tth.24.1780/1-518     VVNRPPAATAEESASSVD--------------------------
T_theileri_Tth.12.1030/1-678     SYTEVVNPKE---------REED----------SKE---E----
T_theileri_Tth.129.1010/1-165    --------------------------------------------
T_theileri_Tth.2.1510/1-887      TPVDIKESTGTHGSSSAASTRSD---------------------
T_theileri_Tth.31.1030/1-517     --------------------------------------------
T_theileri_Tth.36.2110/1-825     PSKDPDIVEEVQTSLPDGETTTA---------------------
T_theileri_Tth.151.1000/1-123    --------------------------------------------
T_theileri_Tth.136.1000/1-654    HVQENTPLEIKAKWVSSTPTNSN---------------------
T_theileri_Tth.14.1410/1-752     LPHQDDDDQPAPKNITWPIKPKD---------------------
T_theileri_Tth.46.1740/1-120     --------------------------------------------
T_theileri_Tth.31.1020/1-118     --------------------------------------------
T_theileri_Tth.124.1030/1-577    VNPHQPSAASAGP-------------------------------
```

```
                                       1370      1380      1390      1400
T_theileri_Tth.54.1380/1-425   --------------------------------------------
T_theileri_Tth.107.1000/1-244  --------------------------------------------
T_theileri_Tth.54.1340/1-680   --------------------------------------------
T_theileri_Tth.117.1040/1-195  --------------------------------------------
T_theileri_Tth.6.5060/1-554    --------------------------------------------
T_theileri_Tth.1.6360/1-511    --------------------------------------------
T_theileri_Tth.46.1110/1-516   --------------------------------------------
T_theileri_Tth.33.1800/1-520   --------------------------------------------
T_theileri_Tth.46.1020/1-842   -----------------------------------AKNKMEEDN
T_theileri_Tth.70.1140/1-882   -------------------------------------TRRESQQ
T_theileri_Tth.87.1050/1-130   --------------------------------------------
T_theileri_Tth.144.1010/1-374  --------------------------------------------
T_theileri_Tth.39.1000/1-654   --------------------------------------------
T_theileri_Tth.12.1010/1-802   AS---PPTVPGDV-------------VEENEEV-----PPENDN
T_theileri_Tth.11.3500/1-528   --------------------------------------------
T_theileri_Tth.124.1020/1-215  --------------------------------------------
T_theileri_Tth.54.1390/1-443   --------------------------------------------
T_theileri_Tth.26.2480/1-314   --------------------------------------------
T_theileri_Tth.43.1970/1-318   --------------------------------------------
T_theileri_Tth.37.1300/1-102   --------------------------------------------
T_theileri_Tth.11.2410/1-509   --------------------------------------------
T_theileri_Tth.70.1150/1-914   --------------------------------------------
T_theileri_Tth.31.1050/1-313   --------------------------------------------
T_theileri_Tth.70.1010/1-670   ----------------------------------------ANGK
T_theileri_Tth.36.2090/1-806   --------------------------------------------
T_theileri_Tth.117.1050/1-116  --------------------------------------------
T_theileri_Tth.11.3480/1-173   --------------------------------------------
T_theileri_Tth.31.1070/1-736   --------------------------------------------
T_theileri_Tth.19.2140/1-590   KLLAALSTPFLFLLALFSNIS-----------------------
T_theileri_Tth.10.2870/1-312   --------------------------------------------
T_theileri_Tth.46.1010/1-442   --------------------------------------------
T_theileri_Tth.12.1820/1-197   --------------------------------------------
T_theileri_Tth.2.2110/1-566    --------------------------------------------
T_theileri_Tth.70.1050/1-869   --------------------------------------EEEHQK
T_theileri_Tth.71.1110/1-97    --------------------------------------------
T_theileri_Tth.4.4270/1-365    --------------------------------------------
T_theileri_Tth.70.1120/1-459   --------------------------------------------
T_theileri_Tth.36.1940/1-208   --------------------------------------------
T_theileri_Tth.14.1390/1-407   --------------------------------------------
T_theileri_Tth.24.2340/1-572   --------------------------------------------
T_theileri_Tth.101.1060/1-233  --------------------------------------------
T_theileri_Tth.9.3510/1-513    --------------------------------------------
T_theileri_Tth.33.1860/1-659   --------------------------------------------
T_theileri_Tth.134.1010/1-723  --------------------------------------------
T_theileri_Tth.11.3620/1-114   --------------------------------------------
T_theileri_Tth.144.1000/1-229  --------------------------------------------
T_theileri_Tth.24.1780/1-518   --------------------------------------------
T_theileri_Tth.12.1030/1-678   --------------------------------------------
T_theileri_Tth.129.1010/1-165  --------------------------------------------
T_theileri_Tth.2.1510/1-887    --------------------------------------------
T_theileri_Tth.31.1030/1-517   --------------------------------------------
T_theileri_Tth.36.2110/1-825   --------------------------------------------
T_theileri_Tth.151.1000/1-123  --------------------------------------------
T_theileri_Tth.136.1000/1-654  --------------------------------------------
T_theileri_Tth.14.1410/1-752   --------------------------------------------
T_theileri_Tth.46.1740/1-120   --------------------------------------------
T_theileri_Tth.31.1020/1-118   --------------------------------------------
T_theileri_Tth.124.1030/1-577  --------------------------------------------
```

```
                              1410      1420      1430      1440      1450
T_theileri_Tth.54.1380/1-425  --------------------------------------------
T_theileri_Tth.107.1000/1-244 --------------------------------------------
T_theileri_Tth.54.1340/1-680  --------------------------------------------
T_theileri_Tth.117.1040/1-195 --------------------------------------------
T_theileri_Tth.6.5060/1-554   --------------------------------------------
T_theileri_Tth.1.6360/1-511   --------------------------------------------
T_theileri_Tth.46.1110/1-516  --------------------------------------------
T_theileri_Tth.33.1800/1-520  --------------------------------------------
T_theileri_Tth.46.1020/1-842  HKMEEKEQLVEERSQVNTNSQQVPPLPADGKMK-----------
T_theileri_Tth.70.1140/1-882  REKEREKEKGKETAKQPEPSPPTDLQQVQQSHTQPNPRENPTSD
T_theileri_Tth.87.1050/1-130  --------------------------------------------
T_theileri_Tth.144.1010/1-374 --------------------------------------------
T_theileri_Tth.39.1000/1-654  --------------------------------------------
T_theileri_Tth.12.1010/1-802  --------------------------------------------
T_theileri_Tth.11.3500/1-528  --------------------------------------------
T_theileri_Tth.124.1020/1-215 --------------------------------------------
T_theileri_Tth.54.1390/1-443  --------------------------------------------
T_theileri_Tth.26.2480/1-314  --------------------------------------------
T_theileri_Tth.43.1970/1-318  --------------------------------------------
T_theileri_Tth.37.1300/1-102  --------------------------------------------
T_theileri_Tth.11.2410/1-509  --------------------------------------------
T_theileri_Tth.70.1150/1-914  -EKERERDEDKENAKHPEPSPPTRSQRAPQTNPQPNPRENPTSE
T_theileri_Tth.31.1050/1-313  --------------------------------------------
T_theileri_Tth.70.1010/1-670  AEEEEKSNTQSGLASQTQPQHHVPALA-----------------
T_theileri_Tth.36.2090/1-806  --------------------------------------------
T_theileri_Tth.117.1050/1-116 --------------------------------------------
T_theileri_Tth.11.3480/1-173  --------------------------------------------
T_theileri_Tth.31.1070/1-736  ----KDAQPEQESPVHPDPKLNTSSKP-----------------
T_theileri_Tth.19.2140/1-590  --------------------------------------------
T_theileri_Tth.10.2870/1-312  --------------------------------------------
T_theileri_Tth.46.1010/1-442  --------------------------------------------
T_theileri_Tth.12.1820/1-197  --------------------------------------------
T_theileri_Tth.2.2110/1-566   --------------------------------------------
T_theileri_Tth.70.1050/1-869  VEKEKEVISIQGPSLPPDSEMK----------------------
T_theileri_Tth.71.1110/1-97   --------------------------------------------
T_theileri_Tth.4.4270/1-365   --------------------------------------------
T_theileri_Tth.70.1120/1-459  --------------------------------------------
T_theileri_Tth.36.1940/1-208  --------------------------------------------
T_theileri_Tth.14.1390/1-407  --------------------------------------------
T_theileri_Tth.24.2340/1-572  --------------------------------------------
T_theileri_Tth.101.1060/1-233 --------------------------------------------
T_theileri_Tth.9.3510/1-513   --------------------------------------------
T_theileri_Tth.33.1860/1-659  --------------------------------------------
T_theileri_Tth.134.1010/1-723 --------------------------------------------
T_theileri_Tth.11.3620/1-114  --------------------------------------------
T_theileri_Tth.144.1000/1-229 --------------------------------------------
T_theileri_Tth.24.1780/1-518  --------------------------------------------
T_theileri_Tth.12.1030/1-678  --------------------------------------------
T_theileri_Tth.129.1010/1-165 --------------------------------------------
T_theileri_Tth.2.1510/1-887   --------------------------------------------
T_theileri_Tth.31.1030/1-517  --------------------------------------------
T_theileri_Tth.36.2110/1-825  --------------------------------------------
T_theileri_Tth.151.1000/1-123 --------------------------------------------
T_theileri_Tth.136.1000/1-654 --------------------------------------------
T_theileri_Tth.14.1410/1-752  --------------------------------------------
T_theileri_Tth.46.1740/1-120  --------------------------------------------
T_theileri_Tth.31.1020/1-118  --------------------------------------------
T_theileri_Tth.124.1030/1-577 --------------------------------------------
```

```
                                        1460      1470      1480      1490
T_theileri_Tth.54.1380/1-425   --------------------------------------------
T_theileri_Tth.107.1000/1-244  --------------------------------------------
T_theileri_Tth.54.1340/1-680   --------------------------------------------
T_theileri_Tth.117.1040/1-195  --------------------------------------------
T_theileri_Tth.6.5060/1-554    ------------------------------------TKDQNNSS
T_theileri_Tth.1.6360/1-511    --------------------------------------------
T_theileri_Tth.46.1110/1-516   --------------------------------------------
T_theileri_Tth.33.1800/1-520   --------------------------------------------
T_theileri_Tth.46.1020/1-842   -------------------------------------ENQSKPA
T_theileri_Tth.70.1140/1-882   VT-----------------------------------VEHGGSA
T_theileri_Tth.87.1050/1-130   --------------------------------------------
T_theileri_Tth.144.1010/1-374  --------------------------------------------
T_theileri_Tth.39.1000/1-654   --------------------------------------------
T_theileri_Tth.12.1010/1-802   ------------------------------------NGTETGKE
T_theileri_Tth.11.3500/1-528   --------------------------------------------
T_theileri_Tth.124.1020/1-215  --------------------------------------------
T_theileri_Tth.54.1390/1-443   --------------------------------------------
T_theileri_Tth.26.2480/1-314   --------------------------------------------
T_theileri_Tth.43.1970/1-318   --------------------------------------------
T_theileri_Tth.37.1300/1-102   --------------------------------------------
T_theileri_Tth.11.2410/1-509   --------------------------------------------
T_theileri_Tth.70.1150/1-914   VT-----------------------------------VEHGGSD
T_theileri_Tth.31.1050/1-313   --------------------------------------------
T_theileri_Tth.70.1010/1-670   -------------------------------------VDQRGSA
T_theileri_Tth.36.2090/1-806   --------------------------------------------
T_theileri_Tth.117.1050/1-116  --------------------------------------------
T_theileri_Tth.11.3480/1-173   --------------------------------------------
T_theileri_Tth.31.1070/1-736   -------------------------------------------D
T_theileri_Tth.19.2140/1-590   --------------------------------------------
T_theileri_Tth.10.2870/1-312   --------------------------------------------
T_theileri_Tth.46.1010/1-442   --------------------------------------------
T_theileri_Tth.12.1820/1-197   --------------------------------------------
T_theileri_Tth.2.2110/1-566    --------------------------------------------
T_theileri_Tth.70.1050/1-869   -------------------------------------ENHSKPA
T_theileri_Tth.71.1110/1-97    --------------------------------------------
T_theileri_Tth.4.4270/1-365    --------------------------------------------
T_theileri_Tth.70.1120/1-459   --------------------------------------------
T_theileri_Tth.36.1940/1-208   --------------------------------------------
T_theileri_Tth.14.1390/1-407   --------------------------------------------
T_theileri_Tth.24.2340/1-572   --------------------------------------------
T_theileri_Tth.101.1060/1-233  --------------------------------------------
T_theileri_Tth.9.3510/1-513    --------------------------------------------
T_theileri_Tth.33.1860/1-659   --------------------------------------------
T_theileri_Tth.134.1010/1-723  ------------------------------------TKAQNSSS
T_theileri_Tth.11.3620/1-114   --------------------------------------------
T_theileri_Tth.144.1000/1-229  --------------------------------------------
T_theileri_Tth.24.1780/1-518   --------------------------------------------
T_theileri_Tth.12.1030/1-678   ------------------------------------TKEESNSS
T_theileri_Tth.129.1010/1-165  --------------------------------------------
T_theileri_Tth.2.1510/1-887    --------------------------------------------
T_theileri_Tth.31.1030/1-517   --------------------------------------------
T_theileri_Tth.36.2110/1-825   --------------------------------------------
T_theileri_Tth.151.1000/1-123  --------------------------------------------
T_theileri_Tth.136.1000/1-654  --------------------------------------------
T_theileri_Tth.14.1410/1-752   --------------------------------------------
T_theileri_Tth.46.1740/1-120   --------------------------------------------
T_theileri_Tth.31.1020/1-118   --------------------------------------------
T_theileri_Tth.124.1030/1-577  --------------------------------------------
```

```
                                  1500      1510      1520      1530
T_theileri_Tth.54.1380/1-425    --------------------------------------------
T_theileri_Tth.107.1000/1-244   --------------------------------------------
T_theileri_Tth.54.1340/1-680    --------------------------------------------
T_theileri_Tth.117.1040/1-195   --------------------------------------------
T_theileri_Tth.6.5060/1-554     TNSEVSGDV-ANTESGTS-----------------------TN-
T_theileri_Tth.1.6360/1-511     --------------------------------------------
T_theileri_Tth.46.1110/1-516    --------------------------------------------
T_theileri_Tth.33.1800/1-520    --------------------------------------------
T_theileri_Tth.46.1020/1-842    EQSALDTSGKQSPAPSNAGNQGDSA-GVGQEDVSL--PASPEPR
T_theileri_Tth.70.1140/1-882    VAGNANGTSHVPTAPSQAEDQRDSA-GGGKEEVALPHPAVPEPQ
T_theileri_Tth.87.1050/1-130    --------------------------------------------
T_theileri_Tth.144.1010/1-374   --------------------------------------------
T_theileri_Tth.39.1000/1-654    --------------------------------------------
T_theileri_Tth.12.1010/1-802    --------------------------------PGTP-EVPPT--
T_theileri_Tth.11.3500/1-528    --------------------------------------------
T_theileri_Tth.124.1020/1-215   --------------------------------------------
T_theileri_Tth.54.1390/1-443    --------------------------------------------
T_theileri_Tth.26.2480/1-314    --------------------------------------------
T_theileri_Tth.43.1970/1-318    --------------------------------------------
T_theileri_Tth.37.1300/1-102    --------------------------------------------
T_theileri_Tth.11.2410/1-509    --------------------------------------------
T_theileri_Tth.70.1150/1-914    VANKGNGTSHVPTAPSQAEDQRDSA-GGGKEEVALPHPAVPEPQ
T_theileri_Tth.31.1050/1-313    --------------------------------------------
T_theileri_Tth.70.1010/1-670    VADKENVESHVSPASSQARDQRGSS-AEGEKEVFISSSCT----
T_theileri_Tth.36.2090/1-806    --------------------------------------------
T_theileri_Tth.117.1050/1-116   --------------------------------------------
T_theileri_Tth.11.3480/1-173    --------------------------------------------
T_theileri_Tth.31.1070/1-736    EAPKDREDSQELPAAEVTENQRDSP-REGVKEVLSP-LAVPEPQ
T_theileri_Tth.19.2140/1-590    --------------------------------------------
T_theileri_Tth.10.2870/1-312    --------------------------------------------
T_theileri_Tth.46.1010/1-442    --------------------------------------------
T_theileri_Tth.12.1820/1-197    --------------------------------------------
T_theileri_Tth.2.2110/1-566     --------------------------------------------
T_theileri_Tth.70.1050/1-869    EHSAGEISGKQPVASPQAGNQGGAP-AEGESEVLSP-PASTAPQ
T_theileri_Tth.71.1110/1-97     --------------------------------------------
T_theileri_Tth.4.4270/1-365     --------------------------------------------
T_theileri_Tth.70.1120/1-459    --------------------------------------------
T_theileri_Tth.36.1940/1-208    --------------------------------------------
T_theileri_Tth.14.1390/1-407    --------------------------------------------
T_theileri_Tth.24.2340/1-572    --------------------------------------------
T_theileri_Tth.101.1060/1-233   --------------------------------------------
T_theileri_Tth.9.3510/1-513     --------------------------------------------
T_theileri_Tth.33.1860/1-659    --------------------------------------------
T_theileri_Tth.134.1010/1-723   TNSEASRGV-VNGGSETS-----------------------AT-
T_theileri_Tth.11.3620/1-114    --------------------------------------------
T_theileri_Tth.144.1000/1-229   --------------------------------------------
T_theileri_Tth.24.1780/1-518    --------------------------------------------
T_theileri_Tth.12.1030/1-678    SNSEASGVS-VNAESGTS-----------------------TN-
T_theileri_Tth.129.1010/1-165   --------------------------------------------
T_theileri_Tth.2.1510/1-887     --------------------------------------------
T_theileri_Tth.31.1030/1-517    --------------------------------------------
T_theileri_Tth.36.2110/1-825    --------------------------------------------
T_theileri_Tth.151.1000/1-123   --------------------------------------------
T_theileri_Tth.136.1000/1-654   --------------------------------------------
T_theileri_Tth.14.1410/1-752    --------------------------------------------
T_theileri_Tth.46.1740/1-120    --------------------------------------------
T_theileri_Tth.31.1020/1-118    --------------------------------------------
T_theileri_Tth.124.1030/1-577   --------------------------------------------
```

```
                                     1550      1560      1570      1580
T_theileri_Tth.54.1380/1-425   --------------------------------------------
T_theileri_Tth.107.1000/1-244  --------------------------------------------
T_theileri_Tth.54.1340/1-680   --------------------------------------------
T_theileri_Tth.117.1040/1-195  --------------------------------------------
T_theileri_Tth.6.5060/1-554    -----------------------------TQGESAPAAP-----
T_theileri_Tth.1.6360/1-511    --------------------------------------------
T_theileri_Tth.46.1110/1-516   --------------------------------------------
T_theileri_Tth.33.1800/1-520   --------------------------------------------
T_theileri_Tth.46.1020/1-842   AANEENRAQ-DSNINNSSGALPQEPSIPAPKGPNSEASKEVSS-
T_theileri_Tth.70.1140/1-882   LEREEKQAHENSNNNNTGGLQPQQPTLSAQENSSEPVK------
T_theileri_Tth.87.1050/1-130   --------------------------------------------
T_theileri_Tth.144.1010/1-374  --------------------------------------------
T_theileri_Tth.39.1000/1-654   --------------------------------------------
T_theileri_Tth.12.1010/1-802   ----TNGETMEE------EEVKEEDEEKENEKNETDVVPSGNGT
T_theileri_Tth.11.3500/1-528   --------------------------------------------
T_theileri_Tth.124.1020/1-215  --------------------------------------------
T_theileri_Tth.54.1390/1-443   --------------------------------------------
T_theileri_Tth.26.2480/1-314   --------------------------------------------
T_theileri_Tth.43.1970/1-318   --------------------------------------------
T_theileri_Tth.37.1300/1-102   --------------------------------------------
T_theileri_Tth.11.2410/1-509   --------------------------------------------
T_theileri_Tth.70.1150/1-914   LEREEKQAHENSNNNNTGGLQPQQPTLSAQENSSEPVK------
T_theileri_Tth.31.1050/1-313   --------------------------------------------
T_theileri_Tth.70.1010/1-670   --------------------------------------------
T_theileri_Tth.36.2090/1-806   --------------------------------------------
T_theileri_Tth.117.1050/1-116  --------------------------------------------
T_theileri_Tth.11.3480/1-173   --------------------------------------------
T_theileri_Tth.31.1070/1-736   PAGEENQTQ-NTNNNNRGALPQEPSSISAPKVPSKPVK------
T_theileri_Tth.19.2140/1-590   --------------------------------------------
T_theileri_Tth.10.2870/1-312   --------------------------------------------
T_theileri_Tth.46.1010/1-442   --------------------------------------------
T_theileri_Tth.12.1820/1-197   --------------------------------------------
T_theileri_Tth.2.2110/1-566    --------------------------------------------
T_theileri_Tth.70.1050/1-869   PAGEENKAP-NGNNNNSTALPQEPSSIPPEKNSSEPAK------
T_theileri_Tth.71.1110/1-97    --------------------------------------------
T_theileri_Tth.4.4270/1-365    --------------------------------------------
T_theileri_Tth.70.1120/1-459   --------------------------------------------
T_theileri_Tth.36.1940/1-208   --------------------------------------------
T_theileri_Tth.14.1390/1-407   --------------------------------------------
T_theileri_Tth.24.2340/1-572   --------------------------------------------
T_theileri_Tth.101.1060/1-233  --------------------------------------------
T_theileri_Tth.9.3510/1-513    --------------------------------------------
T_theileri_Tth.33.1860/1-659   --------------------------------------------
T_theileri_Tth.134.1010/1-723  -----------------------------EGEKNASAAPDANGS
T_theileri_Tth.11.3620/1-114   --------------------------------------------
T_theileri_Tth.144.1000/1-229  --------------------------------------------
T_theileri_Tth.24.1780/1-518   --------------------------------------------
T_theileri_Tth.12.1030/1-678   -----------------------------TQGESVPAAPGVNGS
T_theileri_Tth.129.1010/1-165  --------------------------------------------
T_theileri_Tth.2.1510/1-887    --------------------------------------------
T_theileri_Tth.31.1030/1-517   --------------------------------------------
T_theileri_Tth.36.2110/1-825   --------------------------------------------
T_theileri_Tth.151.1000/1-123  --------------------------------------------
T_theileri_Tth.136.1000/1-654  --------------------------------------------
T_theileri_Tth.14.1410/1-752   --------------------------------------------
T_theileri_Tth.46.1740/1-120   --------------------------------------------
T_theileri_Tth.31.1020/1-118   --------------------------------------------
T_theileri_Tth.124.1030/1-577  --------------------------------------------
```

```
                                   1590      1600      1610      1620
T_theileri_Tth.54.1380/1-425   --------------------------------------------
T_theileri_Tth.107.1000/1-244  --------------------------------------------
T_theileri_Tth.54.1340/1-680   --------------------------------------------
T_theileri_Tth.117.1040/1-195  --------------------------------------------
T_theileri_Tth.6.5060/1-554    --------------------------------------------
T_theileri_Tth.1.6360/1-511    --------------------------------------------
T_theileri_Tth.46.1110/1-516   --------------------------------------------
T_theileri_Tth.33.1800/1-520   --------------------------------------------
T_theileri_Tth.46.1020/1-842   --------------------------------------ETTETP
T_theileri_Tth.70.1140/1-882   -------------------------------KPEVEKKPTSEIS
T_theileri_Tth.87.1050/1-130   --------------------------------------------
T_theileri_Tth.144.1010/1-374  --------------------------------------------
T_theileri_Tth.39.1000/1-654   --------------------------------------------
T_theileri_Tth.12.1010/1-802   TTEEGS--------------GKQPTDTTT---GPSD--ESHEEV
T_theileri_Tth.11.3500/1-528   --------------------------------------------
T_theileri_Tth.124.1020/1-215  --------------------------------------------
T_theileri_Tth.54.1390/1-443   --------------------------------------------
T_theileri_Tth.26.2480/1-314   --------------------------------------------
T_theileri_Tth.43.1970/1-318   --------------------------------------------
T_theileri_Tth.37.1300/1-102   --------------------------------------------
T_theileri_Tth.11.2410/1-509   --------------------------------------------
T_theileri_Tth.70.1150/1-914   -------------------------------KPEVENMPTTETP
T_theileri_Tth.31.1050/1-313   --------------------------------------------
T_theileri_Tth.70.1010/1-670   --------------------------------------------
T_theileri_Tth.36.2090/1-806   --------------------------------------------
T_theileri_Tth.117.1050/1-116  --------------------------------------------
T_theileri_Tth.11.3480/1-173   --------------------------------------------
T_theileri_Tth.31.1070/1-736   -------------------------------TPEVEKKPTTEIS
T_theileri_Tth.19.2140/1-590   --------------------------------------------
T_theileri_Tth.10.2870/1-312   ------------------------------------------EE
T_theileri_Tth.46.1010/1-442   --------------------------------------------
T_theileri_Tth.12.1820/1-197   --------------------------------------------
T_theileri_Tth.2.2110/1-566    --------------------------------------------
T_theileri_Tth.70.1050/1-869   -------------------------------KPEVENMPNTENP
T_theileri_Tth.71.1110/1-97    --------------------------------------------
T_theileri_Tth.4.4270/1-365    --------------------------------------------
T_theileri_Tth.70.1120/1-459   --------------------------------------------
T_theileri_Tth.36.1940/1-208   --------------------------------------------
T_theileri_Tth.14.1390/1-407   --------------------------------------------
T_theileri_Tth.24.2340/1-572   --------------------------------------------
T_theileri_Tth.101.1060/1-233  --------------------------------------------
T_theileri_Tth.9.3510/1-513    ---------------------------NASEIPSPNVDSPEKND
T_theileri_Tth.33.1860/1-659   --------------------------------------------
T_theileri_Tth.134.1010/1-723  SHESSA----------------ATPG-------NSTNAESAEEE
T_theileri_Tth.11.3620/1-114   --------------------------------------------
T_theileri_Tth.144.1000/1-229  --------------------------------------------
T_theileri_Tth.24.1780/1-518   --------------------------------------------
T_theileri_Tth.12.1030/1-678   SQESST----------------ATQS-----------------V
T_theileri_Tth.129.1010/1-165  --------------------------------------------
T_theileri_Tth.2.1510/1-887    --------------------------------------------
T_theileri_Tth.31.1030/1-517   --------------------------------------------
T_theileri_Tth.36.2110/1-825   --------------------------------------------
T_theileri_Tth.151.1000/1-123  --------------------------------------------
T_theileri_Tth.136.1000/1-654  --------------------------------------------
T_theileri_Tth.14.1410/1-752   --------------------------------------------
T_theileri_Tth.46.1740/1-120   --------------------------------------------
T_theileri_Tth.31.1020/1-118   --------------------------------------------
T_theileri_Tth.124.1030/1-577  --------------------------------------------
```

```
                                  1630      1640      1650      1660      1670
T_theileri_Tth.54.1380/1-425   --------------------------------------------
T_theileri_Tth.107.1000/1-244  --------------------------------------------
T_theileri_Tth.54.1340/1-680   --------------------------------------------
T_theileri_Tth.117.1040/1-195  --------------------------------------------
T_theileri_Tth.6.5060/1-554    --------------------------------------------
T_theileri_Tth.1.6360/1-511    --------------------------------------------
T_theileri_Tth.46.1110/1-516   --------------------------------------------
T_theileri_Tth.33.1800/1-520   --------------------------------------------
T_theileri_Tth.46.1020/1-842   KPVKTEPNAVS-EQTSTIKDEDKKNKTENIEQVQETITQSQPQN
T_theileri_Tth.70.1140/1-882   KPVVSEPIAVS-EDTPSKKEEDHNNTTENIEQVQETITESPSQN
T_theileri_Tth.87.1050/1-130   --------------------------------------------
T_theileri_Tth.144.1010/1-374  --------------------------------------------
T_theileri_Tth.39.1000/1-654   --------------------------------------------
T_theileri_Tth.12.1010/1-802   NNNSSASEVPPNTED-------------------SPQEKPSQGE
T_theileri_Tth.11.3500/1-528   --------------------------------------------
T_theileri_Tth.124.1020/1-215  --------------------------------------------
T_theileri_Tth.54.1390/1-443   --------------------------------------------
T_theileri_Tth.26.2480/1-314   --------------------------------------------
T_theileri_Tth.43.1970/1-318   --------------------------------------------
T_theileri_Tth.37.1300/1-102   --------------------------------------------
T_theileri_Tth.11.2410/1-509   --------------------------------------------
T_theileri_Tth.70.1150/1-914   TPVVSKPTITL-DDTASIKEEDNNNKTESSEQVQGTITQPPTQD
T_theileri_Tth.31.1050/1-313   --------------------------------------------
T_theileri_Tth.70.1010/1-670   --------------------------------------------
T_theileri_Tth.36.2090/1-806   --------------------------------------------
T_theileri_Tth.117.1050/1-116  --------------------------------------------
T_theileri_Tth.11.3480/1-173   --------------------------------------------
T_theileri_Tth.31.1070/1-736   KPVASEPIAVSGHTPSV--EEDHNNTTENIQQVQETITDSPSQD
T_theileri_Tth.19.2140/1-590   --------------------------------------------
T_theileri_Tth.10.2870/1-312   GNDQNSS-------------------------------------
T_theileri_Tth.46.1010/1-442   --------------------------------------------
T_theileri_Tth.12.1820/1-197   --------------------------------------------
T_theileri_Tth.2.2110/1-566    --------------------------------------------
T_theileri_Tth.70.1050/1-869   KPVASEPIAVS-EQPSSIKEEDKNNTTEGIEQVRETSTQPQPQN
T_theileri_Tth.71.1110/1-97    --------------------------------------------
T_theileri_Tth.4.4270/1-365    --------------------------------------------
T_theileri_Tth.70.1120/1-459   --------------------------------------------
T_theileri_Tth.36.1940/1-208   --------------------------------------------
T_theileri_Tth.14.1390/1-407   --------------------------------------------
T_theileri_Tth.24.2340/1-572   --------------------------------------------
T_theileri_Tth.101.1060/1-233  --------------------------------------------
T_theileri_Tth.9.3510/1-513    SSE-----------------------------------------
T_theileri_Tth.33.1860/1-659   --------------------------------------------
T_theileri_Tth.134.1010/1-723  GQRAPRS-------------------------------------
T_theileri_Tth.11.3620/1-114   --------------------------------------------
T_theileri_Tth.144.1000/1-229  --------------------------------------------
T_theileri_Tth.24.1780/1-518   --------------------------------------------
T_theileri_Tth.12.1030/1-678   MQRASRS-------------------------------------
T_theileri_Tth.129.1010/1-165  --------------------------------------------
T_theileri_Tth.2.1510/1-887    --------------------------------------------
T_theileri_Tth.31.1030/1-517   --------------------------------------------
T_theileri_Tth.36.2110/1-825   --------------------------------------------
T_theileri_Tth.151.1000/1-123  --------------------------------------------
T_theileri_Tth.136.1000/1-654  --------------------------------------------
T_theileri_Tth.14.1410/1-752   --------------------------------------------
T_theileri_Tth.46.1740/1-120   --------------------------------------------
T_theileri_Tth.31.1020/1-118   --------------------------------------------
T_theileri_Tth.124.1030/1-577  --------------------------------------------
```

```
                                  1680      1690      1700      1710
T_theileri_Tth.54.1380/1-425   --------------------------------------------
T_theileri_Tth.107.1000/1-244  --------------------------------------------
T_theileri_Tth.54.1340/1-680   -----------------------------------LSVGTQAAA
T_theileri_Tth.117.1040/1-195  ------------------PSPLPHPPTEPTMETVGAPHVERELT
T_theileri_Tth.6.5060/1-554    --------------------------------------------
T_theileri_Tth.1.6360/1-511    --------------------------------------------
T_theileri_Tth.46.1110/1-516   --------------------------------------------
T_theileri_Tth.33.1800/1-520   --------------------------------------------
T_theileri_Tth.46.1020/1-842   GNDEGINTTVDTTLDP---NSPATDNEK----------------
T_theileri_Tth.70.1140/1-882   GNDEGINTTVDTTHSP---NSPATDDEK--EGGVNADNNTT---
T_theileri_Tth.87.1050/1-130   --------------------------------------------
T_theileri_Tth.144.1010/1-374  --------------------------------------------
T_theileri_Tth.39.1000/1-654   ---------------------------IDVPPHSSVQRGQSPQT
T_theileri_Tth.12.1010/1-802   NSPEAEIPS---------------------EEIES---------
T_theileri_Tth.11.3500/1-528   --------------------------------------------
T_theileri_Tth.124.1020/1-215  --------------------------------------------
T_theileri_Tth.54.1390/1-443   --------------------------------------------
T_theileri_Tth.26.2480/1-314   --------------------------------------------
T_theileri_Tth.43.1970/1-318   --------------------------------------------
T_theileri_Tth.37.1300/1-102   --------------------------------------------
T_theileri_Tth.11.2410/1-509   --------------------------------------------
T_theileri_Tth.70.1150/1-914   GNDE-TKPTVDPVRSPEDVESPSTSDEKRNETIVNTDNNATTST
T_theileri_Tth.31.1050/1-313   --------------------------------------------
T_theileri_Tth.70.1010/1-670   --------------------------------------------
T_theileri_Tth.36.2090/1-806   -----------------------------------LSDGNQP--
T_theileri_Tth.117.1050/1-116  --------------------------------------------
T_theileri_Tth.11.3480/1-173   --------------------------------------------
T_theileri_Tth.31.1070/1-736   GNDEGINTTVDPTLDP---NSPSPDDEK-NETIVNTDNNTT---
T_theileri_Tth.19.2140/1-590   --------------------------------------------
T_theileri_Tth.10.2870/1-312   ---------------SASAGSVGSS-------------------
T_theileri_Tth.46.1010/1-442   --------------------------------------------
T_theileri_Tth.12.1820/1-197   --------------------------------------------
T_theileri_Tth.2.2110/1-566    --------------------------------------------
T_theileri_Tth.70.1050/1-869   GNDEGINTTVDPTHSP---NSPATVDENKNETTVNTDNNKT---
T_theileri_Tth.71.1110/1-97    --------------------------------------------
T_theileri_Tth.4.4270/1-365    --------------------------------------------
T_theileri_Tth.70.1120/1-459   --------------------------------------------
T_theileri_Tth.36.1940/1-208   --------------------------------------------
T_theileri_Tth.14.1390/1-407   -----------------SSDPLPHPAPEPTMETVGAPHVEKEPS
T_theileri_Tth.24.2340/1-572   --------------------------------------------
T_theileri_Tth.101.1060/1-233  --------------------------------------------
T_theileri_Tth.9.3510/1-513    ------------TEKAPQTEGSNPQE-------------KPSQE
T_theileri_Tth.33.1860/1-659   ------------------SEKPNETVDAESESTNSNSGSRPSQG
T_theileri_Tth.134.1010/1-723  ---------------RRHIADAT----------TS---------
T_theileri_Tth.11.3620/1-114   --------------------------------------------
T_theileri_Tth.144.1000/1-229  --------------------------------------------
T_theileri_Tth.24.1780/1-518   ------------------------------------TSVQGDQV
T_theileri_Tth.12.1030/1-678   ---------------RRHIADAT----------AS---------
T_theileri_Tth.129.1010/1-165  --------------------------------------------
T_theileri_Tth.2.1510/1-887    --------------------------------------------
T_theileri_Tth.31.1030/1-517   --------------------------------------------
T_theileri_Tth.36.2110/1-825   -----------------------------------LSDGTQPAT
T_theileri_Tth.151.1000/1-123  --------------------------------------------
T_theileri_Tth.136.1000/1-654  ---------------------------INDPPYSSVQRGQSPQT
T_theileri_Tth.14.1410/1-752   ------------------PSPLTHPPPEPTMETVGAPHVERGLT
T_theileri_Tth.46.1740/1-120   --------------------------------------------
T_theileri_Tth.31.1020/1-118   --------------------------------------------
T_theileri_Tth.124.1030/1-577  ------------------------------------TSLQRGRE
```

```
                                     1720        1730        1740        1750
T_theileri_Tth.54.1380/1-425   --------------------------------------------
T_theileri_Tth.107.1000/1-244  --------------------------------------------
T_theileri_Tth.54.1340/1-680   EATGTAKP---------NE------N-DGNFGVSLPVVQNEAST
T_theileri_Tth.117.1040/1-195  SSNPNVPTDSSGPAHTTTPVQRDVFEKERIDSPTSTAHSETMLD
T_theileri_Tth.6.5060/1-554    -----------ED-AIQSPNTV---------------LNGTNLT
T_theileri_Tth.1.6360/1-511    --------------------------------------------
T_theileri_Tth.46.1110/1-516   --------------------------------------------
T_theileri_Tth.33.1800/1-520   --------------------------------------------
T_theileri_Tth.46.1020/1-842   ----------------------EGGVNADNNTTNTTTSIPENVS
T_theileri_Tth.70.1140/1-882   --------------------------------NTTTSSIPENVN
T_theileri_Tth.87.1050/1-130   --------------------------------------------
T_theileri_Tth.144.1010/1-374  --------------------------------------------
T_theileri_Tth.39.1000/1-654   STVNNAQQTTSTTTSADANRQR-------SSTTSTAIVNNNGKP
T_theileri_Tth.12.1010/1-802   --SKPESPDKND-----TTNIENGQDDNNTGNDSTD--------
T_theileri_Tth.11.3500/1-528   ----------------------------------------DNNG
T_theileri_Tth.124.1020/1-215  -------------------DDAGGDGGGGGDNVGDDNAGGGGGG
T_theileri_Tth.54.1390/1-443   ---------------------------RGNDGNT----------
T_theileri_Tth.26.2480/1-314   --------------------------------------------
T_theileri_Tth.43.1970/1-318   --------------------------------------------
T_theileri_Tth.37.1300/1-102   --------------------------------------------
T_theileri_Tth.11.2410/1-509   --------------------------------------------
T_theileri_Tth.70.1150/1-914   NPVENAQTETSAPTSTPDLSETIIASSEENI-HTTTSSIPENVN
T_theileri_Tth.31.1050/1-313   ---------------------------------------GEENK
T_theileri_Tth.70.1010/1-670   --------------------------------------------
T_theileri_Tth.36.2090/1-806   -ATGTPKP---------SE------N-AGNEGETAPVVQNDAS-
T_theileri_Tth.117.1050/1-116  --------------------------------------------
T_theileri_Tth.11.3480/1-173   --------------------------------------------
T_theileri_Tth.31.1070/1-736   ---------------------------------NTTSSISENVN
T_theileri_Tth.19.2140/1-590   --------------------------------------------
T_theileri_Tth.10.2870/1-312   --GSHDTAAAAATTTSQSSSN----------------PNGTNLT
T_theileri_Tth.46.1010/1-442   --------------------------------------------
T_theileri_Tth.12.1820/1-197   --------------------------------------------
T_theileri_Tth.2.2110/1-566    --------------------------------------------
T_theileri_Tth.70.1050/1-869   --------------------------------NTTTSSIPENVN
T_theileri_Tth.71.1110/1-97    --------------------------------------------
T_theileri_Tth.4.4270/1-365    --------------------------------------------
T_theileri_Tth.70.1120/1-459   --------------------------------------------
T_theileri_Tth.36.1940/1-208   ---------------------------RGNDGNT----------
T_theileri_Tth.14.1390/1-407   SSYPNVPTDSSGSTDTTTPVQRDVFEKERIDSPTSTAHSETMLD
T_theileri_Tth.24.2340/1-572   --------------------------------------------
T_theileri_Tth.101.1060/1-233  --------------------------------------------
T_theileri_Tth.9.3510/1-513    EDTQNAEIPSKEGGLSNP-------------ESPDNNDTTNIEN
T_theileri_Tth.33.1860/1-659   GQGADNTTSAEKALSEDVGASSSSSSSSSSSSSSSSRSRRNSET
T_theileri_Tth.134.1010/1-723  --QSAANPNGTED-TIQSSNTV---------------LNRTNLT
T_theileri_Tth.11.3620/1-114   --------------------------------------------
T_theileri_Tth.144.1000/1-229  --------------------------------------------
T_theileri_Tth.24.1780/1-518   NNGTATNSRSRRDT-TGVTSSTTSNNTQAAAAAGVTSPSGEENA
T_theileri_Tth.12.1030/1-678   --QSTSNPNSTED-AIQSPSTV---------------LNGTNLT
T_theileri_Tth.129.1010/1-165  --------------------------------------------
T_theileri_Tth.2.1510/1-887    ------------------------------------KGKDLPKS
T_theileri_Tth.31.1030/1-517   --------------------------------------------
T_theileri_Tth.36.2110/1-825   EATGTPKP---------SE------N-DGNQGVTSTVQQSGAST
T_theileri_Tth.151.1000/1-123  --------------------------------------------
T_theileri_Tth.136.1000/1-654  VTVNNAQQTTSTTTSADANRQR-------SSTTPTATVNNNGKT
T_theileri_Tth.14.1410/1-752   STHPNVPTDSSGH-HTSERDGLFGSSTHNVPTTGATVEEHMELA
T_theileri_Tth.46.1740/1-120   --------------------------------------------
T_theileri_Tth.31.1020/1-118   --------------------------------------------
T_theileri_Tth.124.1030/1-577  DNGTTINTRGRRDT-TGVTSSTTSNNTQDAAATGASSPSGKENA
```

Sequence	1770 · 1780 · 1790 · 1800
T_theileri_Tth.54.1380/1-425	--
T_theileri_Tth.107.1000/1-244	--
T_theileri_Tth.54.1340/1-680	TKESNPSETKEENITSFLNGQNNNIVAKKGIDGSLKT-YVFASV
T_theileri_Tth.117.1040/1-195	Q---
T_theileri_Tth.6.5060/1-554	ESQMEEES-KNANGTDVLG-----------TDSSTVA-SYMAPL
T_theileri_Tth.1.6360/1-511	--
T_theileri_Tth.46.1110/1-516	--
T_theileri_Tth.33.1800/1-520	--
T_theileri_Tth.46.1020/1-842	ASA-VPATLTELNKTQMGQVINQVG-------------------
T_theileri_Tth.70.1140/1-882	ASA-APATLTELNNTQVGQVINEATTIAIVGADSSIATSYQIPL
T_theileri_Tth.87.1050/1-130	--
T_theileri_Tth.144.1010/1-374	--
T_theileri_Tth.39.1000/1-654	TRSS-TESTTTQGSVKQEDTNKNSKKASNRRKDNSQATKVYSPL
T_theileri_Tth.12.1010/1-802	-GNV-ENTNTPTGVNTGGN-----------ADKSIAL-SFFEPL
T_theileri_Tth.11.3500/1-528	GGDNNGGGGDNVGDDNGGGGGGGHGDGCATASPIALSAIALMVI
T_theileri_Tth.124.1020/1-215	GDNVGDDNAGDDDNAGGGGGGGGHGDGCATASPIALSALALMVI
T_theileri_Tth.54.1390/1-443	-------------------------------DGSVTA-AIFAPL
T_theileri_Tth.26.2480/1-314	--
T_theileri_Tth.43.1970/1-318	--
T_theileri_Tth.37.1300/1-102	--
T_theileri_Tth.11.2410/1-509	--------------------HDQSDIAHRVGMNGNSAAHSLLHA
T_theileri_Tth.70.1150/1-914	ASA-AHATLTGLNNTRMGQALNQATTIAIVGADSSIATSYQISL
T_theileri_Tth.31.1050/1-313	TANNSS-SNHTVAENITCTVGNCDQHTAGEGADKSIVTSYFGAV
T_theileri_Tth.70.1010/1-670	--
T_theileri_Tth.36.2090/1-806	TTKSKPSETKEENITSFLNGQNNNIIAGKGTDGCFKA-SVFASV
T_theileri_Tth.117.1050/1-116	--
T_theileri_Tth.11.3480/1-173	--
T_theileri_Tth.31.1070/1-736	ASATVPATLTGPNKTQMGQVINQATTIAIAGTDSSIATSYQIPL
T_theileri_Tth.19.2140/1-590	--
T_theileri_Tth.10.2870/1-312	EGQMKE-KPNHTN--DARR-----------PDSSIMVISYMAPL
T_theileri_Tth.46.1010/1-442	--
T_theileri_Tth.12.1820/1-197	--
T_theileri_Tth.2.2110/1-566	----------------------KREEPQHKRSDSVANVFFVLPL
T_theileri_Tth.70.1050/1-869	ASATAPATLTGLNNTQMGQVINQATTIAIVGADSSIATSYQIPL
T_theileri_Tth.71.1110/1-97	--
T_theileri_Tth.4.4270/1-365	--
T_theileri_Tth.70.1120/1-459	--
T_theileri_Tth.36.1940/1-208	-------------------------------DGSVTA-AIFAPL
T_theileri_Tth.14.1390/1-407	QNEKHHDGLDGSQHGS----------------------------
T_theileri_Tth.24.2340/1-572	-----------------------------------LSALVPLAM
T_theileri_Tth.101.1060/1-233	--
T_theileri_Tth.9.3510/1-513	GQDDHNTGNDLTDSKVENTNSPTGVISGKDTDNSIIALSFFGPL
T_theileri_Tth.33.1860/1-659	DADVPPTTEGNTNKDNHTSLQVPGELSIHVGMDGTPAAACVLHA
T_theileri_Tth.134.1010/1-723	ESEIKEETLKHTNVTVMFG-----------TDSSIMV-SCMAPL
T_theileri_Tth.11.3620/1-114	--
T_theileri_Tth.144.1000/1-229	--
T_theileri_Tth.24.1780/1-518	NTNA--
T_theileri_Tth.12.1030/1-678	ESQIKEETLKHTNVTVMFG-----------TDSSITV-SYMAPL
T_theileri_Tth.129.1010/1-165	--
T_theileri_Tth.2.1510/1-887	FHGYMLPPEVEHLTEEERAMYREHFRANGGLDSSSTTTLMTSIL
T_theileri_Tth.31.1030/1-517	--
T_theileri_Tth.36.2110/1-825	TTEAKPSETKEEDITSFLSGQNNNIIAGKGTDGSFKA-SAFASV
T_theileri_Tth.151.1000/1-123	--
T_theileri_Tth.136.1000/1-654	TRSS-TESTTTQESVKQEDTNKNSKKASNRRKDNSKATKVYSPL
T_theileri_Tth.14.1410/1-752	DLHIHHSAALDTREAQSEDVQGNHVDAGPSSNSVRTRRELPHEG
T_theileri_Tth.46.1740/1-120	--
T_theileri_Tth.31.1020/1-118	--
T_theileri_Tth.124.1030/1-577	NINDPSKYPGVVSQAQINNMQNESKLVIELGKDDALSAVCSLPI

```
                                 1810      1820      1830      1840
T_theileri_Tth.54.1380/1-425    --------------------------------------------
T_theileri_Tth.107.1000/1-244   --------------------------------------------
T_theileri_Tth.54.1340/1-680    MFVFLTLSMIMLP-------------------------------
T_theileri_Tth.117.1040/1-195   --------------------------------------------
T_theileri_Tth.6.5060/1-554     ALLVCVLLVL----------------------------------
T_theileri_Tth.1.6360/1-511     --------------------------------------------
T_theileri_Tth.46.1110/1-516    --------------------------------------------
T_theileri_Tth.33.1800/1-520    --------------------------------------------
T_theileri_Tth.46.1020/1-842    --------------------------------------------
T_theileri_Tth.70.1140/1-882    LLIVSALVALASP-------------------------------
T_theileri_Tth.87.1050/1-130    --------------------------------------------
T_theileri_Tth.144.1010/1-374   --------------------------------------------
T_theileri_Tth.39.1000/1-654    VLLVLLVSALLISF------------------------------
T_theileri_Tth.12.1010/1-802    MLLVCVVAAVVAL-------------------------------
T_theileri_Tth.11.3500/1-528    TLMLLP--------------------------------------
T_theileri_Tth.124.1020/1-215   TPMLLP--------------------------------------
T_theileri_Tth.54.1390/1-443    LFIFLAVSTIMAP-------------------------------
T_theileri_Tth.26.2480/1-314    --------------------------------------------
T_theileri_Tth.43.1970/1-318    --------------------------------------------
T_theileri_Tth.37.1300/1-102    --------------------------------------------
T_theileri_Tth.11.2410/1-509    LLLAVTLVVVVSL-------------------------------
T_theileri_Tth.70.1150/1-914    LLIVSALVALASP-------------------------------
T_theileri_Tth.31.1050/1-313    LFLVCIAAVMM---------------------------------
T_theileri_Tth.70.1010/1-670    --------------------------------------------
T_theileri_Tth.36.2090/1-806    MFVFLTLSVIMSP-------------------------------
T_theileri_Tth.117.1050/1-116   --------------------------------------------
T_theileri_Tth.11.3480/1-173    --------------------------------------------
T_theileri_Tth.31.1070/1-736    LLIVCALVALASP-------------------------------
T_theileri_Tth.19.2140/1-590    --------------------------------------------
T_theileri_Tth.10.2870/1-312    ALLMCVVGFVMVP-------------------------------
T_theileri_Tth.46.1010/1-442    --------------------------------------------
T_theileri_Tth.12.1820/1-197    --------------------------------------------
T_theileri_Tth.2.2110/1-566     MFLELLVFIVLMA-------------------------------
T_theileri_Tth.70.1050/1-869    LLLFFALVALASP-------------------------------
T_theileri_Tth.71.1110/1-97     --------------------------------------------
T_theileri_Tth.4.4270/1-365     --------------------------------------------
T_theileri_Tth.70.1120/1-459    --------------------------------------------
T_theileri_Tth.36.1940/1-208    LFIFLAVSTIMAP-------------------------------
T_theileri_Tth.14.1390/1-407    --------------------------------------------
T_theileri_Tth.24.2340/1-572    TFTLMGLL------------------------------------
T_theileri_Tth.101.1060/1-233   --------------------------------------------
T_theileri_Tth.9.3510/1-513     MLLVCVVA------------------------------------
T_theileri_Tth.33.1860/1-659    LFLLMAAAAALAVPL-----------------------------
T_theileri_Tth.134.1010/1-723   ALLVCVVGFVMVP-------------------------------
T_theileri_Tth.11.3620/1-114    --------------------------------------------
T_theileri_Tth.144.1000/1-229   --------------------------------------------
T_theileri_Tth.24.1780/1-518    --------------------------------------------
T_theileri_Tth.12.1030/1-678    ALLVCVVGFVMVP-------------------------------
T_theileri_Tth.129.1010/1-165   --------------------------------------------
T_theileri_Tth.2.1510/1-887     ALLCLAVAILVTP-------------------------------
T_theileri_Tth.31.1030/1-517    --------------------------------------------
T_theileri_Tth.36.2110/1-825    MFVFLTLSMIMAP-------------------------------
T_theileri_Tth.151.1000/1-123   --------------------------------------------
T_theileri_Tth.136.1000/1-654   VLLVLVVSALLISF------------------------------
T_theileri_Tth.14.1410/1-752    VTQVATTNENLMQ---DSDTKNNLATNGKGPAAHVLNNNDAITL
T_theileri_Tth.46.1740/1-120    --------------------------------------------
T_theileri_Tth.31.1020/1-118    --------------------------------------------
T_theileri_Tth.124.1030/1-577   MIVTMVLAVVLSC-------------------------------
```

```
                              1850      1860      1870
T_theileri_Tth.54.1380/1-425   --------------------------------
T_theileri_Tth.107.1000/1-244  --------------------------------
T_theileri_Tth.54.1340/1-680   --------------------------------
T_theileri_Tth.117.1040/1-195  --------------------------------
T_theileri_Tth.6.5060/1-554    --------------------------------
T_theileri_Tth.1.6360/1-511    --------------------------------
T_theileri_Tth.46.1110/1-516   --------------------------------
T_theileri_Tth.33.1800/1-520   --------------------------------
T_theileri_Tth.46.1020/1-842   --------------------------------
T_theileri_Tth.70.1140/1-882   --------------------------------
T_theileri_Tth.87.1050/1-130   --------------------------------
T_theileri_Tth.144.1010/1-374  --------------------------------
T_theileri_Tth.39.1000/1-654   --------------------------------
T_theileri_Tth.12.1010/1-802   --------------------------------
T_theileri_Tth.11.3500/1-528   --------------------------------
T_theileri_Tth.124.1020/1-215  --------------------------------
T_theileri_Tth.54.1390/1-443   --------------------------------
T_theileri_Tth.26.2480/1-314   --------------------------------
T_theileri_Tth.43.1970/1-318   --------------------------------
T_theileri_Tth.37.1300/1-102   --------------------------------
T_theileri_Tth.11.2410/1-509   --------------------------------
T_theileri_Tth.70.1150/1-914   --------------------------------
T_theileri_Tth.31.1050/1-313   --------------------------------
T_theileri_Tth.70.1010/1-670   --------------------------------
T_theileri_Tth.36.2090/1-806   --------------------------------
T_theileri_Tth.117.1050/1-116  --------------------------------
T_theileri_Tth.11.3480/1-173   --------------------------------
T_theileri_Tth.31.1070/1-736   --------------------------------
T_theileri_Tth.19.2140/1-590   --------------------------------
T_theileri_Tth.10.2870/1-312   --------------------------------
T_theileri_Tth.46.1010/1-442   --------------------------------
T_theileri_Tth.12.1820/1-197   --------------------------------
T_theileri_Tth.2.2110/1-566    --------------------------------
T_theileri_Tth.70.1050/1-869   --------------------------------
T_theileri_Tth.71.1110/1-97    --------------------------------
T_theileri_Tth.4.4270/1-365    --------------------------------
T_theileri_Tth.70.1120/1-459   --------------------------------
T_theileri_Tth.36.1940/1-208   --------------------------------
T_theileri_Tth.14.1390/1-407   --------------------------------
T_theileri_Tth.24.2340/1-572   --------------------------------
T_theileri_Tth.101.1060/1-233  --------------------------------
T_theileri_Tth.9.3510/1-513    --------------------------------
T_theileri_Tth.33.1860/1-659   --------------------------------
T_theileri_Tth.134.1010/1-723  --------------------------------
T_theileri_Tth.11.3620/1-114   --------------------------------
T_theileri_Tth.144.1000/1-229  --------------------------------
T_theileri_Tth.24.1780/1-518   --------------------------------
T_theileri_Tth.12.1030/1-678   --------------------------------
T_theileri_Tth.129.1010/1-165  --------------------------------
T_theileri_Tth.2.1510/1-887    --------------------------------
T_theileri_Tth.31.1030/1-517   --------------------------------
T_theileri_Tth.36.2110/1-825   --------------------------------
T_theileri_Tth.151.1000/1-123  --------------------------------
T_theileri_Tth.136.1000/1-654  --------------------------------
T_theileri_Tth.14.1410/1-752   NIGIGDGTVTPAGCAPLLLLILASAVAVIFSL
T_theileri_Tth.46.1740/1-120   --------------------------------
T_theileri_Tth.31.1020/1-118   --------------------------------
T_theileri_Tth.124.1030/1-577  --------------------------------
```

```
                                    10        20        30        40
T_theileri_Tth.124.1010/1-124   --------------------------------------------
T_theileri_Tth.52.1320/1-345    --------------------------------------------
T_theileri_Tth.46.1750/1-879    --------------------------------------------
T_theileri_Tth.85.1050/1-656    --------------------------------------------
T_theileri_Tth.63.1230/1-856    --------------------------------------------
T_theileri_Tth.19.2160/1-140    --------------------------------------------
T_theileri_Tth.2.1500/1-116     --------------------------------------------
T_theileri_Tth.11.2420/1-601    --------------------------------------------
T_theileri_Tth.4.4280/1-686     --------------------------------------------
T_theileri_Tth.5.3690/1-642     --------------------------------------------
T_theileri_Tth.24.2350/1-645    --------------------------------------------
T_theileri_Tth.36.2130/1-775    --------------------------------------------
T_theileri_Tth.13.2470/1-271    --------------------------------------------
T_theileri_Tth.38.1990/1-923    --------------------------------------------
T_theileri_Tth.17.2230/1-737    --------------------------------------------
T_theileri_Tth.36.1950/1-407    --------------------------------------------
T_theileri_Tth.71.1100/1-161    --------------------------------------------
T_theileri_Tth.117.1060/1-252   --------------------------------------------
T_theileri_Tth.4.4250/1-116     --------------------------------------------
T_theileri_Tth.97.1070/1-422    --------------------------------------------
T_theileri_Tth.7.4640/1-495     --------------------------------------------
T_theileri_Tth.71.1060/1-278    --------------------------------------------
T_theileri_Tth.11.3640/1-145    --------------------------------------------
T_theileri_Tth.12.1860/1-209    --------------------------------------------
T_theileri_Tth.13.3150/1-731    --------------------------------------------
T_theileri_Tth.12.1040/1-712    --------------------------------------------
T_theileri_Tth.120.1090/1-644   --------------------------------------------
T_theileri_Tth.107.1060/1-758   --------------------------------------------
T_theileri_Tth.71.1000/1-211    --------------------------------------------
T_theileri_Tth.19.2170/1-599    --------------------------------------------
T_theileri_Tth.132.1040/1-509   --------------------------------------------
T_theileri_Tth.39.1020/1-195    --------------------------------------------
T_theileri_Tth.14.1420/1-170    --------------------------------------------
T_theileri_Tth.101.1040/1-225   --------------------------------------------
T_theileri_Tth.39.1010/1-861    --------------------------------------------
T_theileri_Tth.27.1400/1-341    --------------------------------------------
T_theileri_Tth.54.1360/1-370    --------------------------------------------
T_theileri_Tth.13.3260/1-705    --------------------------------------------
T_theileri_Tth.101.1030/1-620   --------------------------------------------
T_theileri_Tth.54.1310/1-800    --------------------------------------------
T_theileri_Tth.54.1370/1-309    --------------------------------------------
T_theileri_Tth.32.2270/1-628    --------------------------------------------
T_theileri_Tth.17.2210/1-197    --------------------------------------------
T_theileri_Tth.36.2100/1-574    --------------------------------------------
T_theileri_Tth.10.2530/1-248    --------------------------------------------
T_theileri_Tth.165.1000/1-309   --------------------------------------------
T_theileri_Tth.29.1010/1-643    --------------------------------------------
T_theileri_Tth.13.3140/1-290    --------------------------------------------
T_theileri_Tth.124.1000/1-107   --------------------------------------------
T_theileri_Tth.4.4470/1-974     --------------------------------------------
T_theileri_Tth.24.1440/1-461    --------------------------------------------
T_theileri_Tth.44.1540/1-561    --------------------------------------------
T_theileri_Tth.91.1000/1-302    --------------------------------------------
T_theileri_Tth.26.2510/1-186    --------------------------------------------
T_theileri_Tth.83.1090/1-415    --------------------------------------------
T_theileri_Tth.61.1050/1-209    --------------------------------------------
T_theileri_Tth.54.1330/1-803    --------------------------------------------
T_theileri_Tth.87.1010/1-146    --------------------------------------------
```

```
                                            50        60        70        80
T_theileri_Tth.124.1010/1-124   --------------------------------------------
T_theileri_Tth.52.1320/1-345    --------------------------------------------
T_theileri_Tth.46.1750/1-879    --------------------------------------------
T_theileri_Tth.85.1050/1-656    --------------------------------------------
T_theileri_Tth.63.1230/1-856    --------------------------------------------
T_theileri_Tth.19.2160/1-140    --------------------------------------------
T_theileri_Tth.2.1500/1-116     --------------------------------------------
T_theileri_Tth.11.2420/1-601    --------------------------------------------
T_theileri_Tth.4.4280/1-686     --------------------------------------------
T_theileri_Tth.5.3690/1-642     --------------------------------------------
T_theileri_Tth.24.2350/1-645    -----------------------------MNILPLHNGLCFFAT
T_theileri_Tth.36.2130/1-775    --------------------------------------------
T_theileri_Tth.13.2470/1-271    --------------------------------------------
T_theileri_Tth.38.1990/1-923    --------------------------------------------
T_theileri_Tth.17.2230/1-737    --------------------------------------------
T_theileri_Tth.36.1950/1-407    --------------------------------------------
T_theileri_Tth.71.1100/1-161    --------------------------------------------
T_theileri_Tth.117.1060/1-252   --------------------------------------------
T_theileri_Tth.4.4250/1-116     --------------------------------------------
T_theileri_Tth.97.1070/1-422    --------------------------------------------
T_theileri_Tth.7.4640/1-495     --------------------------------------------
T_theileri_Tth.71.1060/1-278    --------------------------------------------
T_theileri_Tth.11.3640/1-145    --------------------------------------------
T_theileri_Tth.12.1860/1-209    --------------------------------------------
T_theileri_Tth.13.3150/1-731    --------------------------------------------
T_theileri_Tth.12.1040/1-712    --------------------------------------------
T_theileri_Tth.120.1090/1-644   --------------------------------------------
T_theileri_Tth.107.1060/1-758   --------------------------------------------
T_theileri_Tth.71.1000/1-211    --------------------------------------------
T_theileri_Tth.19.2170/1-599    --------------------------------------------
T_theileri_Tth.132.1040/1-509   --------------------------------------------
T_theileri_Tth.39.1020/1-195    --------------------------------------------
T_theileri_Tth.14.1420/1-170    --------------------------------------------
T_theileri_Tth.101.1040/1-225   --------------------------------------------
T_theileri_Tth.39.1010/1-861    --------------------------------------------
T_theileri_Tth.27.1400/1-341    --------------------------------------------
T_theileri_Tth.54.1360/1-370    --------------------------------------------
T_theileri_Tth.13.3260/1-705    --------------------------------------------
T_theileri_Tth.101.1030/1-620   --------------------------------------------
T_theileri_Tth.54.1310/1-800    --------------------------------------------
T_theileri_Tth.54.1370/1-309    --------------------------------------------
T_theileri_Tth.32.2270/1-628    --------------------------------------------
T_theileri_Tth.17.2210/1-197    --------------------------------------------
T_theileri_Tth.36.2100/1-574    --------------------------------------------
T_theileri_Tth.10.2530/1-248    --------------------------------------------
T_theileri_Tth.165.1000/1-309   --------------------------------------------
T_theileri_Tth.29.1010/1-643    --------------------------------------------
T_theileri_Tth.13.3140/1-290    --------------------------------------------
T_theileri_Tth.124.1000/1-107   --------------------------------------------
T_theileri_Tth.4.4470/1-974     --------------------------------------------
T_theileri_Tth.24.1440/1-461    --------------------------------------------
T_theileri_Tth.44.1540/1-561    --------------------------------------------
T_theileri_Tth.91.1000/1-302    --------------------------------------------
T_theileri_Tth.26.2510/1-186    --------------------------------------------
T_theileri_Tth.83.1090/1-415    --------------------------------------------
T_theileri_Tth.61.1050/1-209    --------------------------------------------
T_theileri_Tth.54.1330/1-803    --------------------------------------------
T_theileri_Tth.87.1010/1-146    --------------------------------------------
```

```
                                    90        100       110       120       130
T_theileri_Tth.124.1010/1-124   --------------------------------------------
T_theileri_Tth.52.1320/1-345    --------------------------------------------
T_theileri_Tth.46.1750/1-879    --------------------------------------------
T_theileri_Tth.85.1050/1-656    --------------------------------------------
T_theileri_Tth.63.1230/1-856    --------------------------------------------
T_theileri_Tth.19.2160/1-140    --------------------------------------------
T_theileri_Tth.2.1500/1-116     --------------------------------------------
T_theileri_Tth.11.2420/1-601    --------------------------------------------
T_theileri_Tth.4.4280/1-686     --------------------------------------------
T_theileri_Tth.5.3690/1-642     --------------------------------------------
T_theileri_Tth.24.2350/1-645    TVVPFDLDWHVRDCSCPALYCAPHSFFSL--SPSLLLVSFSTVE
T_theileri_Tth.36.2130/1-775    --------------------------------------------
T_theileri_Tth.13.2470/1-271    --------------------------------------------
T_theileri_Tth.38.1990/1-923    --------------------------------------------
T_theileri_Tth.17.2230/1-737    --------------------------------------------
T_theileri_Tth.36.1950/1-407    --------------------------------------------
T_theileri_Tth.71.1100/1-161    --------------------------------------------
T_theileri_Tth.117.1060/1-252   --------------------------------------------
T_theileri_Tth.4.4250/1-116     --------------------------------------------
T_theileri_Tth.97.1070/1-422    --------------------------------------------
T_theileri_Tth.7.4640/1-495     ----------------------------------------MLFF
T_theileri_Tth.71.1060/1-278    --------------------------------------------
T_theileri_Tth.11.3640/1-145    --------------------------------------------
T_theileri_Tth.12.1860/1-209    --------------------------------------------
T_theileri_Tth.13.3150/1-731    --------------------------------------------
T_theileri_Tth.12.1040/1-712    --------------------------------------------
T_theileri_Tth.120.1090/1-644   --------------------------------------------
T_theileri_Tth.107.1060/1-758   --------------------------------------------
T_theileri_Tth.71.1000/1-211    --------------------------------------------
T_theileri_Tth.19.2170/1-599    --------------------------------------------
T_theileri_Tth.132.1040/1-509   --------------------------------------------
T_theileri_Tth.39.1020/1-195    --------------------------------------------
T_theileri_Tth.14.1420/1-170    --------------------------------------------
T_theileri_Tth.101.1040/1-225   --------------------------------------------
T_theileri_Tth.39.1010/1-861    --------------------------------------------
T_theileri_Tth.27.1400/1-341    --------------------------------------------
T_theileri_Tth.54.1360/1-370    --------------------------------------------
T_theileri_Tth.13.3260/1-705    --------------------------------------------
T_theileri_Tth.101.1030/1-620   --------------------------------------------
T_theileri_Tth.54.1310/1-800    --------------------------------------------
T_theileri_Tth.54.1370/1-309    --------------------------------------------
T_theileri_Tth.32.2270/1-628    --------------------------------------------
T_theileri_Tth.17.2210/1-197    --------------------------------------------
T_theileri_Tth.36.2100/1-574    --------------------------------------------
T_theileri_Tth.10.2530/1-248    --------------------------------------------
T_theileri_Tth.165.1000/1-309   --------------------------------------------
T_theileri_Tth.29.1010/1-643    --------------------------------------------
T_theileri_Tth.13.3140/1-290    --------------------------------------------
T_theileri_Tth.124.1000/1-107   --------------------------------------------
T_theileri_Tth.4.4470/1-974     --------------------------------------------
T_theileri_Tth.24.1440/1-461    --------------------------------------------
T_theileri_Tth.44.1540/1-561    --------------------------------------------
T_theileri_Tth.91.1000/1-302    --------------------------------------------
T_theileri_Tth.26.2510/1-186    --------------------------------------------
T_theileri_Tth.83.1090/1-415    --------------------------------------------
T_theileri_Tth.61.1050/1-209    --------------------------------------------
T_theileri_Tth.54.1330/1-803    --------------------------------------------
T_theileri_Tth.87.1010/1-146    --------------------------------------------
```

```
                                          140       150       160       170
T_theileri_Tth.124.1010/1-124  ---------------------------MPLLLLLLFLCCASVCV
T_theileri_Tth.52.1320/1-345   ------------------MTTTFVQLRHVVYLLVFLQCCVCAAN
T_theileri_Tth.46.1750/1-879   ------------------MEKHSMRHLLWTAL--FLLYWSCGC-
T_theileri_Tth.85.1050/1-656   ----------------MTRQMHVSLLLLLLLFLFVQLCSTSGIL
T_theileri_Tth.63.1230/1-856   ------------------MEKHSMRHLLWTAL--FLLYCSCGC-
T_theileri_Tth.19.2160/1-140   --------------------------------------------
T_theileri_Tth.2.1500/1-116    ------------------MGFFFFLFFSFALFSPSLLCCACYAR
T_theileri_Tth.11.2420/1-601   ------------MLDTVYRHLHCVNLVQVMFFSFFLFCISEFVV
T_theileri_Tth.4.4280/1-686    ---------MHNSYTCAVVGRYSSHPPVLTLLLLVLLCCTAGCL
T_theileri_Tth.5.3690/1-642    ----------------MTRQMHVSLFLLLLLFLFVQLCSTSGIL
T_theileri_Tth.24.2350/1-645   SIHYMPQQVNKLSARTPATVRQSLYVMP-LLLLLLFLCCACVCV
T_theileri_Tth.36.2130/1-775   ------------------MRRLFL----FAPL--LLLYCTLVT-
T_theileri_Tth.13.2470/1-271   --------------------------------------------
T_theileri_Tth.38.1990/1-923   ------------------MEKHSMRHILWAAL--FLLYCSCGC-
T_theileri_Tth.17.2230/1-737   -------MLAPYACVRAMGHRRYSPHPPVFMLLLMLLCCTAGCL
T_theileri_Tth.36.1950/1-407   --------------------------------------------
T_theileri_Tth.71.1100/1-161   --------------------------------------------
T_theileri_Tth.117.1060/1-252  --------------------------------------------
T_theileri_Tth.4.4250/1-116    ---------MHTPCARAVVGRYSSHPPVLTLLLLVLLCCTAGCL
T_theileri_Tth.97.1070/1-422   --------------------------------------------
T_theileri_Tth.7.4640/1-495    VLFHCFYLGFLGFLRQDFQKVMRFYLVFTTFLLLLLFQCVRSSL
T_theileri_Tth.71.1060/1-278   ------------------MKKLLETIISVMLFLLLFLHCVCGLA
T_theileri_Tth.11.3640/1-145   --------------------------------------------
T_theileri_Tth.12.1860/1-209   --------------------------------------------
T_theileri_Tth.13.3150/1-731   ---------MNTPCARAVVSRYSPHPRVLTLLLLLLLCCTAGCL
T_theileri_Tth.12.1040/1-712   ------------------MEKHSMRHLLWTAL--FLLYCSCGC-
T_theileri_Tth.120.1090/1-644  ----------------MTRQMHVSLFLMLLLFLFVQLCSTSGIL
T_theileri_Tth.107.1060/1-758  --------------------------------------------
T_theileri_Tth.71.1000/1-211   --------------------------------------------
T_theileri_Tth.19.2170/1-599   --------------------MRQLLYVTVLLLVLLPLYCTNGLA
T_theileri_Tth.132.1040/1-509  -------------------------QLLCSALLLFLLCCAYGCA
T_theileri_Tth.39.1020/1-195   --------------------------------------------
T_theileri_Tth.14.1420/1-170   --------------------------------------------
T_theileri_Tth.101.1040/1-225  --------------------------------------------
T_theileri_Tth.39.1010/1-861   ------------------MEKHSMRHLLWAAL--LLLYCSCGC-
T_theileri_Tth.27.1400/1-341   --------------------------------------------
T_theileri_Tth.54.1360/1-370   --------------------------------------------
T_theileri_Tth.13.3260/1-705   ------------------MENYFMRHLLYPVLLLFLLLCCAGTS
T_theileri_Tth.101.1030/1-620  --------------------------------------------
T_theileri_Tth.54.1310/1-800   ------------------MRRLFL----FAPL--LLLYCTLVT-
T_theileri_Tth.54.1370/1-309   --------------------------------------------
T_theileri_Tth.32.2270/1-628   ------------------MEKYFMRHLLYPVLLLFLLLCCTGTS
T_theileri_Tth.17.2210/1-197   --------------------------------------------
T_theileri_Tth.36.2100/1-574   --------------------------------------------
T_theileri_Tth.10.2530/1-248   ----------------------MMRHPLLQVVLLLFICGAMAGP
T_theileri_Tth.165.1000/1-309  --------------------------------------------
T_theileri_Tth.29.1010/1-643   ------------------MEKYFMRHLLYPVLLLFLLLCCAGTS
T_theileri_Tth.13.3140/1-290   --------------------------------------------
T_theileri_Tth.124.1000/1-107  --------------------------------------------
T_theileri_Tth.4.4470/1-974    -----------------------------MQLLMLLLFFVPETM
T_theileri_Tth.24.1440/1-461   --------------------------------------------
T_theileri_Tth.44.1540/1-561   ------------------MSRWQHTFLLLTAL---LLSSASVCF
T_theileri_Tth.91.1000/1-302   --------------------------------------------
T_theileri_Tth.26.2510/1-186   ------------------------------MFLLLLLPCVCGLA
T_theileri_Tth.83.1090/1-415   --------------------------------------------
T_theileri_Tth.61.1050/1-209   ------------------MKNLLEMIISVLLFLLLLSPFVCGLS
T_theileri_Tth.54.1330/1-803   ------------------MRRLFL----FAPL--LLLYCTLVT-
T_theileri_Tth.87.1010/1-146   ------------------MKKLLEMIISVLLFLLLLLPCVCGLV
```

```
                                   180       190       200       210
T_theileri_Tth.124.1010/1-124  A-------------------------------------------
T_theileri_Tth.52.1320/1-345   AGGVWRGYPTAGSAEMGKVDQVLRGRVARAHEQIAVMNGCLSAL
T_theileri_Tth.46.1750/1-879   L-------------------------------------------
T_theileri_Tth.85.1050/1-656   A-------------------------------------------
T_theileri_Tth.63.1230/1-856   L-------------------------------------------
T_theileri_Tth.19.2160/1-140   --------------------------------------------
T_theileri_Tth.2.1500/1-116    T-------------------------------------------
T_theileri_Tth.11.2420/1-601   A-------------------------------------------
T_theileri_Tth.4.4280/1-686    A-------------------------------------------
T_theileri_Tth.5.3690/1-642    A-------------------------------------------
T_theileri_Tth.24.2350/1-645   A-------------------------------------------
T_theileri_Tth.36.2130/1-775   L-------------------------------------------
T_theileri_Tth.13.2470/1-271   --------------------------------------------
T_theileri_Tth.38.1990/1-923   L-------------------------------------------
T_theileri_Tth.17.2230/1-737   A-------------------------------------------
T_theileri_Tth.36.1950/1-407   --------------------------------------------
T_theileri_Tth.71.1100/1-161   --------------------------------------------
T_theileri_Tth.117.1060/1-252  --------------------------------------------
T_theileri_Tth.4.4250/1-116    A-------------------------------------------
T_theileri_Tth.97.1070/1-422   --------------------------------------------
T_theileri_Tth.7.4640/1-495    A-------------------------------------------
T_theileri_Tth.71.1060/1-278   A-------------------------------------------
T_theileri_Tth.11.3640/1-145   --------------------------------------------
T_theileri_Tth.12.1860/1-209   --------------------------------------------
T_theileri_Tth.13.3150/1-731   A-------------------------------------------
T_theileri_Tth.12.1040/1-712   L-------------------------------------------
T_theileri_Tth.120.1090/1-644  A-------------------------------------------
T_theileri_Tth.107.1060/1-758  --------------------------------------------
T_theileri_Tth.71.1000/1-211   --------------------------------------------
T_theileri_Tth.19.2170/1-599   A-------------------------------------------
T_theileri_Tth.132.1040/1-509  A-------------------------------------------
T_theileri_Tth.39.1020/1-195   --------------------------------------------
T_theileri_Tth.14.1420/1-170   --------------------------------------------
T_theileri_Tth.101.1040/1-225  --------------------------------------------
T_theileri_Tth.39.1010/1-861   L-------------------------------------------
T_theileri_Tth.27.1400/1-341   --------------------------------------------
T_theileri_Tth.54.1360/1-370   --------------------------------------------
T_theileri_Tth.13.3260/1-705   F-------------------------------------------
T_theileri_Tth.101.1030/1-620  --------------------------------------------
T_theileri_Tth.54.1310/1-800   L-------------------------------------------
T_theileri_Tth.54.1370/1-309   --------------------------------------------
T_theileri_Tth.32.2270/1-628   F-------------------------------------------
T_theileri_Tth.17.2210/1-197   --------------------------------------------
T_theileri_Tth.36.2100/1-574   --------------------------------------------
T_theileri_Tth.10.2530/1-248   A-------------------------------------------
T_theileri_Tth.165.1000/1-309  --------------------------------------------
T_theileri_Tth.29.1010/1-643   F-------------------------------------------
T_theileri_Tth.13.3140/1-290   --------------------------------------------
T_theileri_Tth.124.1000/1-107  --------------------------------------------
T_theileri_Tth.4.4470/1-974    A-------------------------------------------
T_theileri_Tth.24.1440/1-461   --------------------------------------------
T_theileri_Tth.44.1540/1-561   L-------------------------------------------
T_theileri_Tth.91.1000/1-302   --------------------------------------------
T_theileri_Tth.26.2510/1-186   A-------------------------------------------
T_theileri_Tth.83.1090/1-415   --------------------------------------------
T_theileri_Tth.61.1050/1-209   A-------------------------------------------
T_theileri_Tth.54.1330/1-803   H-------------------------------------------
T_theileri_Tth.87.1010/1-146   A-------------------------------------------
```

```
                                          230       240       250       260
T_theileri_Tth.124.1010/1-124   --------------------------------------------
T_theileri_Tth.52.1320/1-345    KEEMKLCEEGSRRAAAVAKNVRAIVKEFGEMKKKRGEDWEPFEW
T_theileri_Tth.46.1750/1-879    --------------------------------------------
T_theileri_Tth.85.1050/1-656    --------------------------------------------
T_theileri_Tth.63.1230/1-856    --------------------------------------------
T_theileri_Tth.19.2160/1-140    --------------------------------------------
T_theileri_Tth.2.1500/1-116     --------------------------------------------
T_theileri_Tth.11.2420/1-601    --------------------------------------------
T_theileri_Tth.4.4280/1-686     --------------------------------------------
T_theileri_Tth.5.3690/1-642     --------------------------------------------
T_theileri_Tth.24.2350/1-645    --------------------------------------------
T_theileri_Tth.36.2130/1-775    --------------------------------------------
T_theileri_Tth.13.2470/1-271    --------------------------------------------
T_theileri_Tth.38.1990/1-923    --------------------------------------------
T_theileri_Tth.17.2230/1-737    --------------------------------------------
T_theileri_Tth.36.1950/1-407    --------------------------------------------
T_theileri_Tth.71.1100/1-161    --------------------------------------------
T_theileri_Tth.117.1060/1-252   --------------------------------------------
T_theileri_Tth.4.4250/1-116     --------------------------------------------
T_theileri_Tth.97.1070/1-422    --------------------------------------------
T_theileri_Tth.7.4640/1-495     --------------------------------------------
T_theileri_Tth.71.1060/1-278    ----------------------------------------LRDD
T_theileri_Tth.11.3640/1-145    -------------------------------------------A
T_theileri_Tth.12.1860/1-209    --------------------------------------------
T_theileri_Tth.13.3150/1-731    --------------------------------------------
T_theileri_Tth.12.1040/1-712    --------------------------------------------
T_theileri_Tth.120.1090/1-644   --------------------------------------------
T_theileri_Tth.107.1060/1-758   --------------------------------------------
T_theileri_Tth.71.1000/1-211    --------------------------------------------
T_theileri_Tth.19.2170/1-599    --------------------------------------------
T_theileri_Tth.132.1040/1-509   --------------------------------------------
T_theileri_Tth.39.1020/1-195    --------------------------------------------
T_theileri_Tth.14.1420/1-170    --------------------------------------------
T_theileri_Tth.101.1040/1-225   --------------------------------------------
T_theileri_Tth.39.1010/1-861    --------------------------------------------
T_theileri_Tth.27.1400/1-341    --------------------------------------------
T_theileri_Tth.54.1360/1-370    --------------------------------------------
T_theileri_Tth.13.3260/1-705    --------------------------------------------
T_theileri_Tth.101.1030/1-620   --------------------------------------------
T_theileri_Tth.54.1310/1-800    --------------------------------------------
T_theileri_Tth.54.1370/1-309    --------------------------------------------
T_theileri_Tth.32.2270/1-628    --------------------------------------------
T_theileri_Tth.17.2210/1-197    --------------------------------------------
T_theileri_Tth.36.2100/1-574    --------------------------------------------
T_theileri_Tth.10.2530/1-248    --------------------------------------------
T_theileri_Tth.165.1000/1-309   --------------------------------------------
T_theileri_Tth.29.1010/1-643    --------------------------------------------
T_theileri_Tth.13.3140/1-290    --------------------------------------------
T_theileri_Tth.124.1000/1-107   --------------------------------------------
T_theileri_Tth.4.4470/1-974     ---------------------------IRKVEHHCIHAESRVQR
T_theileri_Tth.24.1440/1-461    --------------------------------------------
T_theileri_Tth.44.1540/1-561    ------------------------------------------AD
T_theileri_Tth.91.1000/1-302    --------------------------------------------
T_theileri_Tth.26.2510/1-186    ----------------------------------------LRDD
T_theileri_Tth.83.1090/1-415    --------------------------------------------
T_theileri_Tth.61.1050/1-209    ----------------------------------------LRDD
T_theileri_Tth.54.1330/1-803    --------------------------------------------
T_theileri_Tth.87.1010/1-146    ----------------------------------------LRDD
```

```
                                         270       280       290       300
T_theileri_Tth.124.1010/1-124   --------------QKDGGVRSTGVVR----ELPRK--------
T_theileri_Tth.52.1320/1-345    IGENTNRIVKSINEFGSVRLSXVVVVR----NTPLR--------
T_theileri_Tth.46.1750/1-879    ----------------------AAVVQ----QLPQK--------
T_theileri_Tth.85.1050/1-656    -------------------EPSTKVVL----ELPQQ--------
T_theileri_Tth.63.1230/1-856    ----------------------ATVVQ----QLPQK--------
T_theileri_Tth.19.2160/1-140    --------------------------------------------
T_theileri_Tth.2.1500/1-116     ----------RKANQSSYIANHTXVVR----HLPRA--------
T_theileri_Tth.11.2420/1-601    SSGVRMKYCMFDEVMSRIGNDPVAVIP----EVPRK--------
T_theileri_Tth.4.4280/1-686     -----AIPHRCIHGEISRKNRATGVVR----EIPHR--------
T_theileri_Tth.5.3690/1-642     -------------------EESAGVVQ----NLPQE--------
T_theileri_Tth.24.2350/1-645    QKDG--------------GVQSTGVVR----ELPRK--------
T_theileri_Tth.36.2130/1-775    ----------------------AVTEH----HRCMFDTVALRVG
T_theileri_Tth.13.2470/1-271    ------------DRVAEMSSPPVAVVR----EVPKR--------
T_theileri_Tth.38.1990/1-923    ----------------------AAVVQ----QLPQK--------
T_theileri_Tth.17.2230/1-737    -----ESSHRSMHGETSQKSIQTAMVR----EIPLK--------
T_theileri_Tth.36.1950/1-407    --------------------------------------------
T_theileri_Tth.71.1100/1-161    --------------------------------------------
T_theileri_Tth.117.1060/1-252   --------------------------------------------
T_theileri_Tth.4.4250/1-116     -----TIPHHSMYGETSQKNRPTGVVR----EIPRR--------
T_theileri_Tth.97.1070/1-422    --------------------------------------------
T_theileri_Tth.7.4640/1-495     ---VTEYHHCIHDFITERLSSPEPDIRMSHVSAPRR--------
T_theileri_Tth.71.1060/1-278    SCTYDTTVWKNMKNKTDYTPLPVSIIR----ELPQS--------
T_theileri_Tth.11.3640/1-145    QKDG--------------GVQSTGVVR----ELPRK--------
T_theileri_Tth.12.1860/1-209    --------------------------------------------
T_theileri_Tth.13.3150/1-731    -----AGHHRCMHDQMVQRSEQMAVVR----EIPRK--------
T_theileri_Tth.12.1040/1-712    ----------------------AAVVQ----QLPQK--------
T_theileri_Tth.120.1090/1-644   -------------------ADSAEVVQ----NLPQQ--------
T_theileri_Tth.107.1060/1-758   ----------------------------------MFHTVSSKLG
T_theileri_Tth.71.1000/1-211    --------------------------------------------
T_theileri_Tth.19.2170/1-599    ---TGGRSCGFDAVRRRRGNTPVAVVR----ELPVK--------
T_theileri_Tth.132.1040/1-509   ----------------------AAVVK----PLPEK--------
T_theileri_Tth.39.1020/1-195    --------------------------------------------
T_theileri_Tth.14.1420/1-170    --------------------------------------------
T_theileri_Tth.101.1040/1-225   --------------------------------------------
T_theileri_Tth.39.1010/1-861    ----------------------AAVVQ----QLPQK--------
T_theileri_Tth.27.1400/1-341    --------------------------------------------
T_theileri_Tth.54.1360/1-370    --------------------------------------------
T_theileri_Tth.13.3260/1-705    ----------------------AAVVQ----HLPQR--------
T_theileri_Tth.101.1030/1-620   ----------------------TGVVR----ELPRK--------
T_theileri_Tth.54.1310/1-800    ----------------------AVTEH----HRCIHDRVMMHVG
T_theileri_Tth.54.1370/1-309    --------------------------------------------
T_theileri_Tth.32.2270/1-628    ----------------------SAVVQ----HLPQK--------
T_theileri_Tth.17.2210/1-197    --------------------------------------------
T_theileri_Tth.36.2100/1-574    --------------------------------------------
T_theileri_Tth.10.2530/1-248    -------YACNYDEIKEKSGPPVVVVR----EVPKK--------
T_theileri_Tth.165.1000/1-309   ----------------------------------TK--------
T_theileri_Tth.29.1010/1-643    ----------------------AAVVQ----HLPQR--------
T_theileri_Tth.13.3140/1-290    --------------------------------------------
T_theileri_Tth.124.1000/1-107   -------------------MFSTGVVR----ELPRK--------
T_theileri_Tth.4.4470/1-974     LIIGNTNTSAYSHESNNNNKRKSNNNN----DNEEEEKEGLNTG
T_theileri_Tth.24.1440/1-461    --------------------------R----ELPRK--------
T_theileri_Tth.44.1540/1-561    ATATSPDVHHCSFDDVMYKKGVQNIIK----IVPKQ--------
T_theileri_Tth.91.1000/1-302    --------------------------------------------
T_theileri_Tth.26.2510/1-186    SCTYDTTAWKNMRNKTDYTPLSVSIVR----ELPQS--------
T_theileri_Tth.83.1090/1-415    -------MFDELEVKDENKGVAESVVY----ELPKP--------
T_theileri_Tth.61.1050/1-209    SCTYDTTVWKNMRNKTVYTPSSVPIVR----ELPQS--------
T_theileri_Tth.54.1330/1-803    ----------------------AVTEH----HQCMFDRVTTRIG
T_theileri_Tth.87.1010/1-146    SCKYDTTVWKNMRNKTDYTPSSISIVR----ELPQN--------
```

```
                                   310        320        330        340        350
T_theileri_Tth.124.1010/1-124   -----------------GQSGV-QAYAVST--------------
T_theileri_Tth.52.1320/1-345    -----------------GQSGV-QAYTVSA---------DPNGV
T_theileri_Tth.46.1750/1-879    -----------------RQSGL-QAYTVST---------DTVPG
T_theileri_Tth.85.1050/1-656    -----------------GESAS-RAYTVAT--------------
T_theileri_Tth.63.1230/1-856    -----------------GQSAL-QAYTVSN------------TA
T_theileri_Tth.19.2160/1-140    --------------------------------------------
T_theileri_Tth.2.1500/1-116     -----------------GRAAQ-DTYTVAA--------------
T_theileri_Tth.11.2420/1-601    -----------------GHSAV-QVYTVGG--------------
T_theileri_Tth.4.4280/1-686     -----------------GQGAM-QAYTASA--------------
T_theileri_Tth.5.3690/1-642     -----------------GQGAS-WVYTIVT--------------
T_theileri_Tth.24.2350/1-645    -----------------GQSGL-QAYTVSA--------------
T_theileri_Tth.36.2130/1-775    TTPNGEMFQKMLG----KRKNQ-TTRFA---------EDQTTPS
T_theileri_Tth.13.2470/1-271    -----------------GQGAW-QTYTVAT--------EEGVTG
T_theileri_Tth.38.1990/1-923    -----------------GESAL-QAYTVSA-------PP-APPA
T_theileri_Tth.17.2230/1-737    -----------------GQGGM-QAYTATA------------AV
T_theileri_Tth.36.1950/1-407    --------------------------------------------
T_theileri_Tth.71.1100/1-161    --------------------------------------------
T_theileri_Tth.117.1060/1-252   --------------------------------------------
T_theileri_Tth.4.4250/1-116     -----------------GQGAV-QAYTTSA--------------
T_theileri_Tth.97.1070/1-422    --------------------------------------------
T_theileri_Tth.7.4640/1-495     -----------------SEARS----------------SDGGLK
T_theileri_Tth.71.1060/1-278    -----------------GKSTV-SLYTTTT-----TSTTTTTTT
T_theileri_Tth.11.3640/1-145    -----------------GQSGV-QAYTVAT--------------
T_theileri_Tth.12.1860/1-209    --------------------------------------------
T_theileri_Tth.13.3150/1-731    -----------------GQGAV-QAYTASA--------------
T_theileri_Tth.12.1040/1-712    -----------------GESAL-QAYTVS---------------
T_theileri_Tth.120.1090/1-644   -----------------GQGAS-WGYTVVT--------------
T_theileri_Tth.107.1060/1-758   TSYVLSEHETLRTLNP-KKSTE-LLQTA-----------KAKPP
T_theileri_Tth.71.1000/1-211    --------------------------------------------
T_theileri_Tth.19.2170/1-599    -----------------GQGPW-QAYTAAT--------------
T_theileri_Tth.132.1040/1-509   -----------------GQGAW-DAYTVAV--------------
T_theileri_Tth.39.1020/1-195    --------------------------------------------
T_theileri_Tth.14.1420/1-170    --------------------------------------------
T_theileri_Tth.101.1040/1-225   --------------------------------------------
T_theileri_Tth.39.1010/1-861    -----------------GESAL-QAYTVSA-------VA-LAAT
T_theileri_Tth.27.1400/1-341    --------------------------------------------
T_theileri_Tth.54.1360/1-370    --------------------------------------------
T_theileri_Tth.13.3260/1-705    -----------------GESAL-HAYTVAD--------------
T_theileri_Tth.101.1030/1-620   -----------------GQSGV-QAYAVST--------------
T_theileri_Tth.54.1310/1-800    AEFNGDDCLLGMTTESRRRDPK-ESADSFE-------PKKAKLP
T_theileri_Tth.54.1370/1-309    --------------------------------------------
T_theileri_Tth.32.2270/1-628    -----------------GESAL-HAYTVAE--------------
T_theileri_Tth.17.2210/1-197    --------------------------------------------
T_theileri_Tth.36.2100/1-574    --------------------------------------------
T_theileri_Tth.10.2530/1-248    -----------------GEGTW-QAYTVAT--------------
T_theileri_Tth.165.1000/1-309   -----------------GDELA-EKYAIVT--------------
T_theileri_Tth.29.1010/1-643    -----------------GESAL-HAYTVTE--------------
T_theileri_Tth.13.3140/1-290    --------------------------------------------
T_theileri_Tth.124.1000/1-107   -----------------GQSGV-QAYTVAT--------------
T_theileri_Tth.4.4470/1-974     YEHQKSDNARKSLQGSKSKSRV-KRFNIARRNKKLSNKKDNDGK
T_theileri_Tth.24.1440/1-461    -----------------GQSGV-QAYTVST--------------
T_theileri_Tth.44.1540/1-561    -----------------GEESI-QPYLVST--------------
T_theileri_Tth.91.1000/1-302    --------------------------------------------
T_theileri_Tth.26.2510/1-186    -----------------GKSTV-SLYTTTT---------TTTST
T_theileri_Tth.83.1090/1-415    -----------------GERRL-KATTYLL--------------
T_theileri_Tth.61.1050/1-209    -----------------GKSTV-SLYTTTT--------TSTTNT
T_theileri_Tth.54.1330/1-803    ADFKVDDRDS-IGMQNLRGRRV-SNANG---------RSSPTFK
T_theileri_Tth.87.1010/1-146    -----------------GKSTV-SLYTTTT----------ATTT
```

```
                                  360       370       380       390
T_theileri_Tth.124.1010/1-124   ------QKKNENEGWELIRIEALTENLDE---------------
T_theileri_Tth.52.1320/1-345    TD-TVENNK----TWAPIRIEIFTKDMED---------------
T_theileri_Tth.46.1750/1-879    AT-PDKNGK----EWEPIRIGVYTKLVED---------------
T_theileri_Tth.85.1050/1-656    ------KNR------KFIRINASTLDLKE---------------
T_theileri_Tth.63.1230/1-856    ---DDKTGK----EWQPIRIGVYTKRVED---------------
T_theileri_Tth.19.2160/1-140    --------------------------------------------
T_theileri_Tth.2.1500/1-116     ----ESDTD----NWQPIRIHVHKDKVEK---------------
T_theileri_Tth.11.2420/1-601    --------D----EFSPIRIKALLNNLDE---------------
T_theileri_Tth.4.4280/1-686     ---AITEEEKNKLNWAPIRIKAFTRDLDD---------------
T_theileri_Tth.5.3690/1-642     ------TNR------TFIRINTSTLDLT----------------
T_theileri_Tth.24.2350/1-645    -------QEKDDKEWKLIRIKAFTKDLDN---------------
T_theileri_Tth.36.2130/1-775    GR-EEPVGE----EWKPIRIELVTDYLKK---------------
T_theileri_Tth.13.2470/1-271    EAVAATGEE----KWEPLRINVSYENLED---------------
T_theileri_Tth.38.1990/1-923    ---APKTGV----EWQPIRIAVYTKRVED---------------
T_theileri_Tth.17.2230/1-737    DSGESKDAK----IFEPIRIKAYTRDLND---------------
T_theileri_Tth.36.1950/1-407    --------------------------------------------
T_theileri_Tth.71.1100/1-161    --------------------------------------------
T_theileri_Tth.117.1060/1-252   --------------------------------------------
T_theileri_Tth.4.4250/1-116     --------------------------------------------
T_theileri_Tth.97.1070/1-422    --------------------------------------------
T_theileri_Tth.7.4640/1-495     LPERSFGDE----GWKRIRIGVFTHDLEK---------------
T_theileri_Tth.71.1060/1-278    TS-VKSDEE----DWMPIRIHLSSDELDR---------------
T_theileri_Tth.11.3640/1-145    ---------DENEGWKLIRIKAFTKDLEN---------------
T_theileri_Tth.12.1860/1-209    ---------------HPI--------------------------
T_theileri_Tth.13.3150/1-731    ---AITEEEKDKLNWTPIRIKAFTRDLED---------------
T_theileri_Tth.12.1040/1-712    -----TKDE----NWYPIQITVSAENVDQ---------------
T_theileri_Tth.120.1090/1-644   ------TNR------TFIRINTSTLDLV----------------
T_theileri_Tth.107.1060/1-758   IR-EDP-TN----GWQKIRIKLVTDHLKK---------------
T_theileri_Tth.71.1000/1-211    --------------------------------------------
T_theileri_Tth.19.2170/1-599    -----------YSPWAPIRIFVSTIDLED---------------
T_theileri_Tth.132.1040/1-509   --EDTKSED----GWEPIRIGVYPGGVSV---------------
T_theileri_Tth.39.1020/1-195    ----------------------------M---------------
T_theileri_Tth.14.1420/1-170    --------------------------------------------
T_theileri_Tth.101.1040/1-225   --------------------------------------------
T_theileri_Tth.39.1010/1-861    ---PDKKEG----DWQPIRIGVYTKFVED---------------
T_theileri_Tth.27.1400/1-341    --------------------------------------------
T_theileri_Tth.54.1360/1-370    --------------------------------------------
T_theileri_Tth.13.3260/1-705    ---LEKSGR----NWKPIHIEVSAEGVNN---------------
T_theileri_Tth.101.1030/1-620   ------QKKNENEGWELIRIKALTENLDE---------------
T_theileri_Tth.54.1310/1-800    IR-KDP-QE----GWEPIRIKLVSDHLKE---------------
T_theileri_Tth.54.1370/1-309    --------------------------------------------
T_theileri_Tth.32.2270/1-628    ---DDKSGK----HWKPIHIEVSAEGVNN---------------
T_theileri_Tth.17.2210/1-197    --------------------------------------------
T_theileri_Tth.36.2100/1-574    --------------------------------------------
T_theileri_Tth.10.2530/1-248    ---SEGEND----KWEPLRINVSYENLKE---------------
T_theileri_Tth.165.1000/1-309   -----KEGQ----EWQPIRIHVSAEAVDA---------------
T_theileri_Tth.29.1010/1-643    ---DDKNGK----HWKPIHIEVSAEGVNN---------------
T_theileri_Tth.13.3140/1-290    --------------------------------------------
T_theileri_Tth.124.1000/1-107   ------QKKNENERWELIRIEAFTEDLED---------------
T_theileri_Tth.4.4470/1-974     IK-LKNGTE----TWEPLRIVVDTSLLVD---------------
T_theileri_Tth.24.1440/1-461    ---------EKNEGWELIRIEVFTKDLED---------------
T_theileri_Tth.44.1540/1-561    -------DD----EWGTIRINVSMKDLQS---------------
T_theileri_Tth.91.1000/1-302    --------------------------------------------
T_theileri_Tth.26.2510/1-186    TT-VKSEEE----DWMPIRIHVSSEELDR---------------
T_theileri_Tth.83.1090/1-415    ------NDR----DWAPIRIKVSAEDLKN---------------
T_theileri_Tth.61.1050/1-209    SG-VKSDEE----DWMPIRIHVSSEELDR---------------
T_theileri_Tth.54.1330/1-803    TR-EDP-SN----GWDPIRIKLVTDNLKK---------------
T_theileri_Tth.87.1010/1-146    TT-VNGEEE----DWMPIRIHVSSEELDR---------------
```

```
                                      400       410       420       430
T_theileri_Tth.124.1010/1-124   -----HSLLCSKVNETKH-EEKVKRENRFGEDDGRAEHTKVTVE
T_theileri_Tth.52.1320/1-345    -----PKKHCTTHGQTLVDQM-----------------------
T_theileri_Tth.46.1750/1-879    -----IIEFCERENEDEDEDEELG--------------------
T_theileri_Tth.85.1050/1-656    ------GEYCTEKVTLIKNLKG----------------------
T_theileri_Tth.63.1230/1-856    -----VLKFCEDGK--LPPEYEDEFLETDEVQE-----------
T_theileri_Tth.19.2160/1-140    --------------------------------------------
T_theileri_Tth.2.1500/1-116     -----AFTECSKKVSDLPSINN----------------------
T_theileri_Tth.11.2420/1-601    -----PSKYCSNEGEVRLGLR-----------------------
T_theileri_Tth.4.4280/1-686     -----PSKYCSGIGEVRYDALN----------------------
T_theileri_Tth.5.3690/1-642     -----KDNYCSKGEKSSKPDLNE---------------------
T_theileri_Tth.24.2350/1-645    -----KSRYCTKVDDKVMDFQG----------------------
T_theileri_Tth.36.2130/1-775    ------GQNCSGSVGRK-IDTLL---------------------
T_theileri_Tth.13.2470/1-271    ------GKYCKDKDDKVMNYWTGN--------------------
T_theileri_Tth.38.1990/1-923    -----VLKFCTTGA--LPPEYQDEFLETDEVED-----------
T_theileri_Tth.17.2230/1-737    -----PSKYCTRAGDYCDTPSY----------------------
T_theileri_Tth.36.1950/1-407    --------------------------------------------
T_theileri_Tth.71.1100/1-161    --------------------------------------------
T_theileri_Tth.117.1060/1-252   --------------------------------------------
T_theileri_Tth.4.4250/1-116     --------------------------------------------
T_theileri_Tth.97.1070/1-422    --------------------------------------------
T_theileri_Tth.7.4640/1-495     -----QGRYCAKKDDEVVNDFD----------------------
T_theileri_Tth.71.1060/1-278    -----VMRRCSSGMTVSSHR------------------------
T_theileri_Tth.11.3640/1-145    -----KSRYCTKVGDEVMDFQD----------------------
T_theileri_Tth.12.1860/1-209    --------------------------------------------
T_theileri_Tth.13.3150/1-731    -----PKKYCTAAGEVIWNNGF----------------------
T_theileri_Tth.12.1040/1-712    -----VIRRCMKRKELSEDDRAI---EMKETEE-----------
T_theileri_Tth.120.1090/1-644   -----EGNHCSKVNQPSIKDFK----------------------
T_theileri_Tth.107.1060/1-758   ------GRSCSDKVNGK-IETLL---------------------
T_theileri_Tth.71.1000/1-211    --------------------------------------------
T_theileri_Tth.19.2170/1-599    -----DSRYCPAAGRYRPIYVD----------------------
T_theileri_Tth.132.1040/1-509   -----WIKSCANMRAPTFGRS-----------------------
T_theileri_Tth.39.1020/1-195    -----RRSTARGHLDERKSYFK----------------------
T_theileri_Tth.14.1420/1-170    --------------------------------------------
T_theileri_Tth.101.1040/1-225   --------------------------------------------
T_theileri_Tth.39.1010/1-861    -----MVKFCTTRNPEVLNEYVTAGEEFEQSLEDEEDESEELDH
T_theileri_Tth.27.1400/1-341    --------------------------------------------
T_theileri_Tth.54.1360/1-370    --------------------------------------------
T_theileri_Tth.13.3260/1-705    -----VLRDCDRKRALAEQLKGTGTRRYD---------------
T_theileri_Tth.101.1030/1-620   -----HLLLCSKVNETKH-EEKVIRENRFGEDDGRAEHTKVTVE
T_theileri_Tth.54.1310/1-800    -----GAHHCSDGVST--IKFFL---------------------
T_theileri_Tth.54.1370/1-309    --------------------------------------------
T_theileri_Tth.32.2270/1-628    -----VLRDCDRKRALAEQFNKTVHWNSK---------------
T_theileri_Tth.17.2210/1-197    --------------------------------------------
T_theileri_Tth.36.2100/1-574    --------------------------------------------
T_theileri_Tth.10.2530/1-248    ------DKYCVNKDEKRRDFLD----------------------
T_theileri_Tth.165.1000/1-309   -----AVEKCKDWAAQATKKNIRGDTVDPNYVELVYPE------
T_theileri_Tth.29.1010/1-643    -----VLRDCDRKRALAEQLKGTGTRRYD---------------
T_theileri_Tth.13.3140/1-290    --------------------------------------------
T_theileri_Tth.124.1000/1-107   -----KSRYCTTSGEQVMNFKG----------------------
T_theileri_Tth.4.4470/1-974     -----PSKICTAVGQVRTDFM-----------------------
T_theileri_Tth.24.1440/1-461    -----ASKYCTEAEKEIKKYDG----------------------
T_theileri_Tth.44.1540/1-561    -----ESKYCKKGDAEKRETRPDFL-------------------
T_theileri_Tth.91.1000/1-302    --------------------------------------------
T_theileri_Tth.26.2510/1-186    -----AMRRCSSGMTDSSHR------------------------
T_theileri_Tth.83.1090/1-415    -----ESMYCKAAGEKRPNFL-----------------------
T_theileri_Tth.61.1050/1-209    -----AMRRCSSEMTGSFHR------------------------
T_theileri_Tth.54.1330/1-803    -----DGHHCSDKVKT--LKTLL---------------------
T_theileri_Tth.87.1010/1-146    -----AMRRCSSGMTDSSHR------------------------
```

```
                                       450       460       470       480
T_theileri_Tth.124.1010/1-124  ---------------------DDKKC----GYERLPLEER----
T_theileri_Tth.52.1320/1-345   -----------M---------NTVRCD---GNGVLTVRRKRILL
T_theileri_Tth.46.1750/1-879   -----------V---------FDDICD--GDHKKMTAQKKGILF
T_theileri_Tth.85.1050/1-656   ---------------------EKYAC---EAVDFFTDEMISSFV
T_theileri_Tth.63.1230/1-856   -----------E---------FAELCGVKRGNKKLIAEKKDILL
T_theileri_Tth.19.2160/1-140   --------------------------------------------
T_theileri_Tth.2.1500/1-116    -----------S---------DTHECG---HKNRITAERM----
T_theileri_Tth.11.2420/1-601   -----------K---------VEVFC---RSVSVLTEEKKKIIV
T_theileri_Tth.4.4280/1-686    ---------------------DIIYC---GANDILTVRMKRIIV
T_theileri_Tth.5.3690/1-642    ---------------------EDDQC---QGQLFLSEEMKKTFL
T_theileri_Tth.24.2350/1-645   ---------------------DITKC---YENEILTDKKKETLI
T_theileri_Tth.36.2130/1-775   -----------K---------KEFPCT---EDDVLTPEKEEILV
T_theileri_Tth.13.2470/1-271   ---------------------DKVQC---REVDLMKTDKKNELI
T_theileri_Tth.38.1990/1-923   -----------E---------FEELCGVKGVNKKIIAERKEILL
T_theileri_Tth.17.2230/1-737   ---------------------NRQYC---YQGSLLTVRKKKILE
T_theileri_Tth.36.1950/1-407   --------------------------------------------
T_theileri_Tth.71.1100/1-161   --------------------------------------------
T_theileri_Tth.117.1060/1-252  --------------------------------------------
T_theileri_Tth.4.4250/1-116    --------------------------------------------
T_theileri_Tth.97.1070/1-422   --------------------------------------------
T_theileri_Tth.7.4640/1-495    ---------------------SKHTCT---EEDVLTPEKKNILV
T_theileri_Tth.71.1060/1-278   ---------------------HSPSCR---DGNVLTPQKRDILL
T_theileri_Tth.11.3640/1-145   ---------------------DTDDC---VGSQILTDEKKSMLI
T_theileri_Tth.12.1860/1-209   --------------------------------------------
T_theileri_Tth.13.3150/1-731   ---------------------QRIVC---TYGGILTVRKKRIIL
T_theileri_Tth.12.1040/1-712   -----------D---------NKVLCD-DEN--GITWAKRNLLL
T_theileri_Tth.120.1090/1-644  -----------G---------DDAPC---LDQFVLSEEMKKTFL
T_theileri_Tth.107.1060/1-758  -----------K---------KEFTCT---DHDVLTPEKEKILV
T_theileri_Tth.71.1000/1-211   --------------------------------------------
T_theileri_Tth.19.2170/1-599   -----------K---------RYVHC---QPKDVLRDEQRNALI
T_theileri_Tth.132.1040/1-509  ---------------------IHSYCS---GGYRITPEKVQ-ML
T_theileri_Tth.39.1020/1-195   -----------G---------STLSCR---GDDVLSEDKKNTLL
T_theileri_Tth.14.1420/1-170   --------------------------------------------
T_theileri_Tth.101.1040/1-225  --------------------------------------------
T_theileri_Tth.39.1010/1-861   LE---------Q---------LKQRCQ-SDE--KMTEEKKKVLF
T_theileri_Tth.27.1400/1-341   --------------------------------------------
T_theileri_Tth.54.1360/1-370   --------------------------------------------
T_theileri_Tth.13.3260/1-705   ---------------------DSVFCD---NKTGITDEKKDLLL
T_theileri_Tth.101.1030/1-620  ---------------------DDKKC----GYKRLPPEERNELM
T_theileri_Tth.54.1310/1-800   -----------K---------DNFTCK---EDDILTAEKKKILV
T_theileri_Tth.54.1370/1-309   --------------------------------------------
T_theileri_Tth.32.2270/1-628   ---------------------EKVFCD---NETGITDAKKDLLL
T_theileri_Tth.17.2210/1-197   --------------------------------------------
T_theileri_Tth.36.2100/1-574   --------------------------------------------
T_theileri_Tth.10.2530/1-248   -----------G---------EEQNC---VSIDLMNEEKKKRLT
T_theileri_Tth.165.1000/1-309  ---------------------ATEHCS---EETLITEEKRDILV
T_theileri_Tth.29.1010/1-643   ---------------------DSVFCD---NKTGITDEKKELLL
T_theileri_Tth.13.3140/1-290   --------------------------------------------
T_theileri_Tth.124.1000/1-107  ---------------------GTAIC---NDDNLLTDKKKETLI
T_theileri_Tth.4.4470/1-974    -----------G---------STHTCL---SQDIMTLEKVWIIE
T_theileri_Tth.24.1440/1-461   ---------------------TTHYC---SRIDVVVVDTIKKFM
T_theileri_Tth.44.1540/1-561   -----------G---------GNLPCT---KEAEITPEKMKNLT
T_theileri_Tth.91.1000/1-302   --------------------------------------------
T_theileri_Tth.26.2510/1-186   ---------------------HSPSCR---DGNVLTPQKRDILL
T_theileri_Tth.83.1090/1-415   -----------G---------ELVEC---EETDVITEAKKNDLL
T_theileri_Tth.61.1050/1-209   ---------------------HSPSCR---DGNVLTPQKRDILL
T_theileri_Tth.54.1330/1-803   -----------N---------GEFSCT---KDDVLTPEKEKILV
T_theileri_Tth.87.1010/1-146   ---------------------HSPSCR---DGNVLTPQKRDILL
```

```
                                    490       500       510       520
T_theileri_Tth.124.1010/1-124  --------------------------------------------
T_theileri_Tth.52.1320/1-345   ERVIPAAIKLHRDRLSVVPVTGTILVP----------RKNIGY-
T_theileri_Tth.46.1750/1-879   TEVLPKAIKLHTDRLKVKRVGKSLNIRIADL------DSPTKKR
T_theileri_Tth.85.1050/1-656   ETIIPAAIKLHADRLLVDPESGPLIVPE---------FNETSV-
T_theileri_Tth.63.1230/1-856   NKVLPKAIKLHADRLNVKQVKESLKIESEDV--------LPEE-
T_theileri_Tth.19.2160/1-140   -------------RLLVKPLKGSFKVPQ---------YNKSDT-
T_theileri_Tth.2.1500/1-116    --------------------------------------------
T_theileri_Tth.11.2420/1-601   KQVIPAAINLHAERLSVVPLKGPVRIP----------QKNIGL-
T_theileri_Tth.4.4280/1-686    EQIIPEAIKMHSERLSVVPTEG-IKVPR----------MSGGY-
T_theileri_Tth.5.3690/1-642    DTILPAAIKLHADRLLVDPVEGPLKVPD---------FEEGSV-
T_theileri_Tth.24.2350/1-645   NEMIPAAIKLHRDRLRVQPHKGNLTVPP---------FQDKSY-
T_theileri_Tth.36.2130/1-775   KKILPEAIELHKERLFVKPLKGPIVVPQ---------FSKNDGL
T_theileri_Tth.13.2470/1-271   KKILPEAIKLHTDRLLVKRVKTPLNELT---------FENKEV-
T_theileri_Tth.38.1990/1-923   NKVLPKAIKLHADRLNVEQVKESLKIETDSV------KKPPQE-
T_theileri_Tth.17.2230/1-737   EWSVPAAIKLHMDRLQVQRAEEIVKMPR-----------EGGV-
T_theileri_Tth.36.1950/1-407   --------------------------------------------
T_theileri_Tth.71.1100/1-161   --------------------------------------------
T_theileri_Tth.117.1060/1-252  --------------------------------------------
T_theileri_Tth.4.4250/1-116    --------------------------------------------
T_theileri_Tth.97.1070/1-422   --------------------------------------------
T_theileri_Tth.7.4640/1-495    NTILKEAVQLHSERLRVQPFKGKLVVPE---------FSNESI-
T_theileri_Tth.71.1060/1-278   NELLPAAIALHSERLLVVRSRFNLVIIQFISEM-----------
T_theileri_Tth.11.3640/1-145   DHIIPGAIKLHRDRLRVQPQEGKLKVPK---------FENGNP-
T_theileri_Tth.12.1860/1-209   -------------------------------------SDAEGTM
T_theileri_Tth.13.3150/1-731   EQALPAAIKMHAERISVVRETGIVKVPR----------EDIGH-
T_theileri_Tth.12.1040/1-712   REILPAAIKLHRDRLMVQRGDGKFVTKKVLM------GSFKDK-
T_theileri_Tth.120.1090/1-644  DTILPAAIKLHADRLLVDPVEGPLKVPE---------FEDDNV-
T_theileri_Tth.107.1060/1-758  EEILPEAIKLHEERLLVEPLKGPIVMPK---------FSKPNAL
T_theileri_Tth.71.1000/1-211   --------------------------------------------
T_theileri_Tth.19.2170/1-599   NHIIPGAMKLHQERLLVKPLKGTFKVPV---------FGEHDT-
T_theileri_Tth.132.1040/1-509  NDIVAEGAKMHSDRLRVKRVKGRLFLDKNGG------KDFKKN-
T_theileri_Tth.39.1020/1-195   NKILPAAIKLHSDLLLVKQLETPFKVPDFGK-----------TL
T_theileri_Tth.14.1420/1-170   --------------------------------------------
T_theileri_Tth.101.1040/1-225  --------------------------------------------
T_theileri_Tth.39.1010/1-861   TEVLPKAIKLHTDRLKVEMKRSSTSETE---------INILIG-
T_theileri_Tth.27.1400/1-341   --------------------------------------------
T_theileri_Tth.54.1360/1-370   --------------------------------------------
T_theileri_Tth.13.3260/1-705   NKLLLAAIKLHTDRLNIEQKEGEVVVSTSTI------NSFSGE-
T_theileri_Tth.101.1030/1-620  NEVIPAAIQLHRDRLLVRPVSGKLKVPE---------FKDEA--
T_theileri_Tth.54.1310/1-800   DEILPEAIKLHKERLFVKRLNGSIVVPN---------FSDEN-L
T_theileri_Tth.54.1370/1-309   --------------------------------------------
T_theileri_Tth.32.2270/1-628   NKLLLAAIQLHTDRLNVEEKEGEAVVSTSTI------NSFSGE-
T_theileri_Tth.17.2210/1-197   --------------------------------------------
T_theileri_Tth.36.2100/1-574   --------------------------------------------
T_theileri_Tth.10.2530/1-248   DEIVPEAIKLHTDRLLVQRIKTPWKVPN---------LRDHAV-
T_theileri_Tth.165.1000/1-309  KKLLPAAIKLHSERLSVHPVQGNLVLHKTL-------FEGDAP-
T_theileri_Tth.29.1010/1-643   NKLLLAAIKLHTDRLNIEQKEGEVVVSTSTI------NSFSGE-
T_theileri_Tth.13.3140/1-290   --------------------------------------------
T_theileri_Tth.124.1000/1-107  KEVIPAAIKLHRDRLLVKPQEG----------------------
T_theileri_Tth.4.4470/1-974    NVALPLAIYRVQQLLNVNPLQGPLLVTKNI--------------
T_theileri_Tth.24.1440/1-461   EDIIPTAIQLHSDRLLVRPVSGKLRVPK---------FADDSY-
T_theileri_Tth.44.1540/1-561   DVIIPEAVKLHAQRLEVVHVKEPFKVPD---------FTNDDSV
T_theileri_Tth.91.1000/1-302   --------------------------------------------
T_theileri_Tth.26.2510/1-186   NELLPAAIALHSERLLVVRSRFNLVIMQFISEM-----------
T_theileri_Tth.83.1090/1-415   KTILPEAIKLHADRLLVKPVPGPVVVS----------VPSKGV-
T_theileri_Tth.61.1050/1-209   NELLPAAITLHSERLLVVRSRFNLVIMQFISEM-----------
T_theileri_Tth.54.1330/1-803   KEILPEAIKLHEERLFVERLKGPIVVPQ---------FSKNDGL
T_theileri_Tth.87.1010/1-146   NELLPAAI------------------------------------
```

```
                                530       540       550       560       570
T_theileri_Tth.124.1010/1-124   --------------------------------------------
T_theileri_Tth.52.1320/1-345    CDKFNIP-EEHHT-------TGLKGADLYLYVSAVPGV------
T_theileri_Tth.46.1750/1-879    CKVIKG-SLEQQM-------QYSVDVDYMIYVGLS---TA----
T_theileri_Tth.85.1050/1-656    CSKFTVP-TEHHS-------KGVENTDMVLYVAARP--------
T_theileri_Tth.63.1230/1-856    CEGFGI-PEGHKE-------KGIPDADFVIYASLS---TN----
T_theileri_Tth.19.2160/1-140    CSQFSVP-PEHYN-------PGISGYDTVMYAAAGPEHME----
T_theileri_Tth.2.1500/1-116     --------------------------------------------
T_theileri_Tth.11.2420/1-601    CQWFIIP-REHHT-------VGVDGGDFFLYVSAMQSE------
T_theileri_Tth.4.4280/1-686     CGYFNIP-EEHHT-------EGLVDADLYIYVSAMPSR------
T_theileri_Tth.5.3690/1-642     CKNFTVP-DGHRK-------EGVENADMVLYVAARP--------
T_theileri_Tth.24.2350/1-645    CKHFTVP-DEHHD-------KGVENADFVLYVAAGT--------
T_theileri_Tth.36.2130/1-775    CYQFM--SQDDER-------EKSFTTDMVLFAAAQPTT-D----
T_theileri_Tth.13.2470/1-271    CSRFAIP-KEVDGDKLKVDEVKLSEADFLLYVASGSSGDK----
T_theileri_Tth.38.1990/1-923    CGEFGI-PKEHET-------NGIPNADFVIYARLS---TN----
T_theileri_Tth.17.2230/1-737    CGWYHIP-EEHFT-------KGLADADLHVYVSAAAS-------
T_theileri_Tth.36.1950/1-407    --------------------------------------------
T_theileri_Tth.71.1100/1-161    --------------------------------------------
T_theileri_Tth.117.1060/1-252   --------------------------------------------
T_theileri_Tth.4.4250/1-116     --------------------------------------------
T_theileri_Tth.97.1070/1-422    --------------------------------------------
T_theileri_Tth.7.4640/1-495     CATFAVP-KEHHT-------DGVPGADMVLYVSALPSMYP----
T_theileri_Tth.71.1060/1-278    CYTYVELPAAYES-------VGVVEADFVLFVLAETVAPFVVIC
T_theileri_Tth.11.3640/1-145    CTHFTVP-SDHHS-------EGVENADFVLY-------------
T_theileri_Tth.12.1860/1-209    CAEMVPVFDGDEG-------MGIPNADFVIYLGLSTKKP-----
T_theileri_Tth.13.3150/1-731    CSGFHIP-EEHHT-------IGLQDADMHLYVAALQCR------
T_theileri_Tth.12.1040/1-712    CTDVVV-GERFEN-------RGFNDADFVLFVGLA---AS----
T_theileri_Tth.120.1090/1-644   CKKFIVP-QDHHS-------KGVENADMVLYVAARP--------
T_theileri_Tth.107.1060/1-758   CSQFTI-PTDGGS-------TGISGYDMVLFAAAEPTP-E----
T_theileri_Tth.71.1000/1-211    --------------------------------------------
T_theileri_Tth.19.2170/1-599    CSQFSVP-PEHYN-------PGFSGYDTVMYAAAGPQHME----
T_theileri_Tth.132.1040/1-509   CPNAVA-RLDYLQ-------KGFPNVDFALFVGPTVTREH----
T_theileri_Tth.39.1020/1-195    CSHFTVP-PTHTS-------EGVKDTYMVLYIAVGPSNTP----
T_theileri_Tth.14.1420/1-170    --------------------------------------------
T_theileri_Tth.101.1040/1-225   --------------------------------------------
T_theileri_Tth.39.1010/1-861    CELFKV-PLEGKV-------HETLGVDFMIYVGLS---TE----
T_theileri_Tth.27.1400/1-341    --------------------------------------------
T_theileri_Tth.54.1360/1-370    --------------------------------------------
T_theileri_Tth.13.3260/1-705    CGVIKS--WEHK--------KSFSKADFVLFVGLDESEMS----
T_theileri_Tth.101.1030/1-620   CNFFTVP-KEHRD-------VGV-DADFALYVIAT---------
T_theileri_Tth.54.1310/1-800    CSKFIPEEYKDKT-------RNFSGYDMVLFAAAAPTP-K----
T_theileri_Tth.54.1370/1-309    --------------------------------------------
T_theileri_Tth.32.2270/1-628    CGVIKV--WDHKT-------NAFSNADFVLFVSLDESEMS----
T_theileri_Tth.17.2210/1-197    --------------------------------------------
T_theileri_Tth.36.2100/1-574    --------------------------------------------
T_theileri_Tth.10.2530/1-248    CSHFTSP-DDHTS-------QDVQDADYLLYVAAGPNKKL----
T_theileri_Tth.165.1000/1-309   CSFFKP-PEKHHS-------TGVPGADFVLYVTTNKKSDE----
T_theileri_Tth.29.1010/1-643    CGVIKA--WEHK--------KSFSKADFVLFVGPDESEMS----
T_theileri_Tth.13.3140/1-290    --------------------------------------------
T_theileri_Tth.124.1000/1-107   --------------------------------------------
T_theileri_Tth.4.4470/1-974     CGPDVTIPESHST-------VGVLDADMILYAHAGGMDNSATPG
T_theileri_Tth.24.1440/1-461    CKHFTVP-EKHRT-------EGVENADFVLYVAAGH--------
T_theileri_Tth.44.1540/1-561    CKYFTVP-EDHKE-------SGVPNADMVLYVAAGPGK------
T_theileri_Tth.91.1000/1-302    --------------------------------------------
T_theileri_Tth.26.2510/1-186    CYTYVELPAAYES-------VGVVQADFVLFVL-----------
T_theileri_Tth.83.1090/1-415    CPHFTVP-EEHMT-------RGVNLTDFVLYVAAGPGA------
T_theileri_Tth.61.1050/1-209    CYTYVELPAAYES-------VGVVQADFVLFVLAETVAPXCYF-
T_theileri_Tth.54.1330/1-803    CSQLI--PEEHKT-------TGVPEADMVLFVAAQPTT-N----
T_theileri_Tth.87.1010/1-146    --------------------------------------------
```

```
                                      580       590       600       610
T_theileri_Tth.124.1010/1-124  --------------------------------------------
T_theileri_Tth.52.1320/1-345   --SAVAWAIDYRRL--SNG------RPHAGIVGINPPLCE----
T_theileri_Tth.46.1750/1-879   --PQNV---EICTQD-EEN------RPTSAVISFIPDEIK----
T_theileri_Tth.85.1050/1-656   ---VNNIHEICARN--DAG------RPIAGAINILPFRTN----
T_theileri_Tth.63.1230/1-856   --IGH----GICSKD-KQG------RPTSAVIKFNLYDIE----
T_theileri_Tth.19.2160/1-140   --GTMAWGVMCATL--TDG------RPVAGGIYLSPREIT----
T_theileri_Tth.2.1500/1-116    --------------------------------------------
T_theileri_Tth.11.2420/1-601   --VVMAWAMPCARL--ESG------RPFLGGMNLDPMGVS----
T_theileri_Tth.4.4280/1-686    --DTAAWAVSCSYL--KDK------RPYAGVINLTPSYVK----
T_theileri_Tth.5.3690/1-642    ---EEAFGVTCAYDD-KSG------RPVAGAINVQIYPLK----
T_theileri_Tth.24.2350/1-645   ---GEPFGVTCAPAE-ASS------RPIAGAINISPYYLV----
T_theileri_Tth.36.2130/1-775   --GAFAWAATCATLS-SNG------RPVIGIINYGPRYIV----
T_theileri_Tth.13.2470/1-271   --DPASFALTCAVDA-ESK------RPIIGAMHVKAEVIK----
T_theileri_Tth.38.1990/1-923   --IGH----GICSKD-KQG------RPTSAVIKFVLYDIE----
T_theileri_Tth.17.2230/1-737   --DSYSYEMTCLRL--KNH------RPIAIAINLEPRSVM----
T_theileri_Tth.36.1950/1-407   --------------------------------------------
T_theileri_Tth.71.1100/1-161   --------------------------------------------
T_theileri_Tth.117.1060/1-252  --------------LLENK------RPIAVAINVEPMTVA----
T_theileri_Tth.4.4250/1-116    --------------------------------------------
T_theileri_Tth.97.1070/1-422   --------------------------------------------
T_theileri_Tth.7.4640/1-495    --RSFAWAVSCATLDSKRGRVSNPGRSVVGVMNYAPKYIL----
T_theileri_Tth.71.1060/1-278   SEADDG-------------------RPTSAAMNFAPADIV----
T_theileri_Tth.11.3640/1-145   --------------------------------------------
T_theileri_Tth.12.1860/1-209   -------GTKICTYD-VKG------RPTSAMIKLNPFEIK----
T_theileri_Tth.13.3150/1-731   --SESAWAIACGHLK-TNG------RPFIAAVNLDPRRVE----
T_theileri_Tth.12.1040/1-712   --RQDV---KICAED-PIYS----NRPISAYITFQPKEIE----
T_theileri_Tth.120.1090/1-644  ---ELAFGVPCAYDN-NSG------RPVAGAINVQIFPLK----
T_theileri_Tth.107.1060/1-758  --ETFAWAATCATLG-PNG------RPVVGIINYGPRYIV----
T_theileri_Tth.71.1000/1-211   --------------------------------------------
T_theileri_Tth.19.2170/1-599   --GTVAWAIKCATL--PNG------RPVTGILYFTPQYIT----
T_theileri_Tth.132.1040/1-509  --------PKICTKD-DKK------RPTSALIKVNTKEFM----
T_theileri_Tth.39.1020/1-195   --FIDNYNTS--------GN----ECLSCGERKNIPTEKS----
T_theileri_Tth.14.1420/1-170   --------------------------------------------
T_theileri_Tth.101.1040/1-225  --------------------------------------------
T_theileri_Tth.39.1010/1-861   --YKKI---KICTKD-KEN------RPTSAVIKFVPEEIK----
T_theileri_Tth.27.1400/1-341   -------------------------------MNFGAQVAT----
T_theileri_Tth.54.1360/1-370   --------------------------------------------
T_theileri_Tth.13.3260/1-705   --------TIVCSQD-LEE------RPTSALIKFVPKDIL----
T_theileri_Tth.101.1030/1-620  ---KTTFGYTCAWD--STG------RPIVGAVSYVREARG----
T_theileri_Tth.54.1310/1-800   --GTFAWAATCATLG-RNG------RPVIGIINYGPRYIV----
T_theileri_Tth.54.1370/1-309   --------------------------------------------
T_theileri_Tth.32.2270/1-628   --------TRVCSQD-LDQ------RPTSALIKFVPKDIL----
T_theileri_Tth.17.2210/1-197   -------------------------MPD----------------
T_theileri_Tth.36.2100/1-574   ---------TCATLG-PDG------RPVVGIINYGPRYIV----
T_theileri_Tth.10.2530/1-248   --WSSPWAVTCAIDE-QTK------RPMVGAMNIHPVYTD----
T_theileri_Tth.165.1000/1-309  -------SVKICAYG-YGH------RPIAAVKNFLPSEIG----
T_theileri_Tth.29.1010/1-643   --------TIVCSQD-LEE------RPTSALIKFVPKDIV----
T_theileri_Tth.13.3140/1-290   --------------------------------------------
T_theileri_Tth.124.1000/1-107  --------------------------------------------
T_theileri_Tth.4.4470/1-974    VMGTIAWAAHC-ELN-EAG------RPIVGHINFVPSFIQWFVS
T_theileri_Tth.24.1440/1-461   ---GGRFGVTCAVEN-SSG------RPIVGAVNYVPQLQD----
T_theileri_Tth.44.1540/1-561   -----TFAAPCATA--EDG------RPVVAAMNFAYFNLT----
T_theileri_Tth.91.1000/1-302   --------------------------------------------
T_theileri_Tth.26.2510/1-186   --------------------------------------------
T_theileri_Tth.83.1090/1-415   -----TFTSICSEES-IED------RPFSAVMNFEPARIL----
T_theileri_Tth.61.1050/1-209   --------------------------------------------
T_theileri_Tth.54.1330/1-803   --GTFAWAATCATLD-SDG------RPVIGIINYGPRYIV----
T_theileri_Tth.87.1010/1-146   --------------------------------------------
```

Alignment positions: 620, 630, 640, 650

T_theileri_Tth.124.1010/1-124	--
T_theileri_Tth.52.1320/1-345	--
T_theileri_Tth.46.1750/1-879	--ATRKYIRLTAHEIAHGLGFDYEVMETQ-GM-----IEPHK--
T_theileri_Tth.85.1050/1-656	--SQRYYVRVMAHGIAHVLGFDYNVFKNN-SM-----VVESN--
T_theileri_Tth.63.1230/1-856	--ATRKYIRFTAHEIAHGLGFQHEFMDNL-TM-----IEPSN--
T_theileri_Tth.19.2160/1-140	--NTSQMVRVVAHEMAHILGFDREVFSAN-KM-----ITLVH--
T_theileri_Tth.2.1500/1-116	--
T_theileri_Tth.11.2420/1-601	--STKIFARVVAHEIAHALGFNQHLFTRR-NM-----ISNIS--
T_theileri_Tth.4.4280/1-686	--PTDSFIRVMVHEIGHILGYDEFTFAAL-NI-----IGTHT--
T_theileri_Tth.5.3690/1-642	--MQRNNVRRVAHEIAHALGFDYKVFEKN-NM-----VAKIE--
T_theileri_Tth.24.2350/1-645	--YPRGSVRAVAHEMAHALGFDYERMEQL-KM-----VTKAN--
T_theileri_Tth.36.2130/1-775	--ATPQRVRVAAHEIAHALGFNLPEMEMK-DM-----VGKVN--
T_theileri_Tth.13.2470/1-271	--FGRSIVRLLAHELGHALGFDYKRMLDL-NM-----TAVHN--
T_theileri_Tth.38.1990/1-923	--ATRKYIRFTAHEIAHGLGFQHDLMKGL-GM-----IVSSK--
T_theileri_Tth.17.2230/1-737	--ATDHHIRTVAHEIAHGLGFESATFARL-NM-----TTVLEEK
T_theileri_Tth.36.1950/1-407	---------VAAHEIAHALGFNVHNMEGK-SM-----LRRQY--
T_theileri_Tth.71.1100/1-161	--
T_theileri_Tth.117.1060/1-252	--PTESHIRTVAHEIAHGLGFDGTTFALL-KM-----TSAVE--
T_theileri_Tth.4.4250/1-116	--AT--
T_theileri_Tth.97.1070/1-422	--
T_theileri_Tth.7.4640/1-495	--ATAQRVRVAAHEIAHVLGFSVSEMDRL-KM-----IKDVG--
T_theileri_Tth.71.1060/1-278	--NTRLFTRIIAHNLAHALGFDVRRISEM-GMIAKSGITTTT--
T_theileri_Tth.11.3640/1-145	--
T_theileri_Tth.12.1860/1-209	--YTPHYVRVVAHEIAHGLGFSMDVDKFR-EM-----VVGKE--
T_theileri_Tth.13.3150/1-731	--PTSSYVRVVAHEIGHALGFDSAMFPRL-GM-----MGTFF--
T_theileri_Tth.12.1040/1-712	--NARKVVRYAAHKIAHGLGLTYGR--ML-SR-----HKATN--
T_theileri_Tth.120.1090/1-644	--IQRYNVRRVAHEIAHVLGFDYTVFEKN-NM-----VAEIQ--
T_theileri_Tth.107.1060/1-758	--GTPQRVRVAAHEIAHALGFNFELMKMD-RL-----VGKVD--
T_theileri_Tth.71.1000/1-211	--
T_theileri_Tth.19.2170/1-599	--NTSQMVRAAAHEMAHVLGFHGEVFIKQSDM-------YAR--
T_theileri_Tth.132.1040/1-509	--PTRYYFRLAAHDIGHGLGFDLDVFKEH-SL-----MREIP--
T_theileri_Tth.39.1020/1-195	--INRRRTQLHVHGITAILLLTWMSKNKK-TI------------
T_theileri_Tth.14.1420/1-170	--
T_theileri_Tth.101.1040/1-225	--
T_theileri_Tth.39.1010/1-861	--ATRQYIRLTAHEIAHALGFHHDLMQTL-GM-----IETLD--
T_theileri_Tth.27.1400/1-341	--ATENNVRVVTHEIAHALGFSVTMLQSH-SS-----VVQIP--
T_theileri_Tth.54.1360/1-370	--
T_theileri_Tth.13.3260/1-705	--DTRHFVRTAAHEIAHGLGFEVTRMRKL-KL-----IKKGA--
T_theileri_Tth.101.1030/1-620	--GLRLNVRRAAQQIAHALGFKIAEMQKKQGM-----LPDVE--
T_theileri_Tth.54.1310/1-800	--ATPQRVRVAAHEIAHALGFDLEVMESK-SM-----VRRGV--
T_theileri_Tth.54.1370/1-309	--
T_theileri_Tth.32.2270/1-628	--DTRHFVRTAAHEIAHGLGFDVTRMQNY-GK-----IRLGG--
T_theileri_Tth.17.2210/1-197	--
T_theileri_Tth.36.2100/1-574	--ATPQRVRVAAHEIAHALGFNVPEMELK-DM-----VGRVN--
T_theileri_Tth.10.2530/1-248	--FTRANVRFIAHQLAHALGFDYKNMMKE-EL-----KDK----
T_theileri_Tth.165.1000/1-309	--ETRRLVRMAAHDIAHALGFDTRRMERI-GM-----IYSATIN
T_theileri_Tth.29.1010/1-643	--DTRHFIRTAAHEIAHGLGFEVTRMRKL-KL-----IKYGA--
T_theileri_Tth.13.3140/1-290	--
T_theileri_Tth.124.1000/1-107	--
T_theileri_Tth.4.4470/1-974	GWEYEYYVRIIIHELLHALGFTPTFIDPMVN------ISERR--
T_theileri_Tth.24.1440/1-461	--GIRFNVRRIAQEIAHALGFDHKRMKEK-GM-----LSTVE--
T_theileri_Tth.44.1540/1-561	--NVRVYTRFAAHEIAHALGFSFYQMNKL-NM-----TRATK--
T_theileri_Tth.91.1000/1-302	----------AAHEIAHALGFDYKNFVDN-KM-----VSTLE--
T_theileri_Tth.26.2510/1-186	--
T_theileri_Tth.83.1090/1-415	--PTRYAVRIAAHEIAHALGFSFRRMREL-EM-----TDYFD--
T_theileri_Tth.61.1050/1-209	--
T_theileri_Tth.54.1330/1-803	--ATPQRVRVAAHEIAHALGFNFELMKRK-DM-----VRNDV--
T_theileri_Tth.87.1010/1-146	--

```
                                        670       680       690       700
T_theileri_Tth.124.1010/1-124  --------------------------------------------
T_theileri_Tth.52.1320/1-345   -------------------------------------TDKLYHS
T_theileri_Tth.46.1750/1-879   -NGLS------SPGGKYGGK---EFYM-KSSETLEI-LKKFYEC
T_theileri_Tth.85.1050/1-656   -FEKGG-----------------KRVLVNSSKTVKK-VQEHYGC
T_theileri_Tth.63.1230/1-856   -KDGK------SKGVS-RGL---TPRMVKFVKSVDK-LKSHYNC
T_theileri_Tth.19.2160/1-140   -DVRG-------------KS---NVHMLTSEKVMEK-AQEH---
T_theileri_Tth.2.1500/1-116    --------------------------------------------
T_theileri_Tth.11.2420/1-601   -NVRG-------------KA---NVRVISTPKVKEF-TGKYYNC
T_theileri_Tth.4.4280/1-686    -NVRG-------------KK---KVWIINTTKSRDV-ARKYFDC
T_theileri_Tth.5.3690/1-642    -SKGTK-----------------GRVVVKSNQTKKM-AQKYYNC
T_theileri_Tth.24.2350/1-645   --VRGM-----------------NRKLVSSKRTKEK-AQAHYNC
T_theileri_Tth.36.2130/1-775   -STSD-------------KA---AFFVVASDNTRRE-AMKQYNC
T_theileri_Tth.13.2470/1-271   -IRGE------------------NRTVVNSTNVLKK-AKEHYNC
T_theileri_Tth.38.1990/1-923   -TYLKPDAVPCSKKRS-AGT---SDYMVKFKEALEV-LKNHYKC
T_theileri_Tth.17.2230/1-737   KYIRG-------------KP---YVFLIATPKAKEI-AQQYYNC
T_theileri_Tth.36.1950/1-407   -TIAT------------------ITPYVVSQNTRRA-AKLHYNC
T_theileri_Tth.71.1100/1-161   --------------------------------------------
T_theileri_Tth.117.1060/1-252  NVVRG-------------KP---HVFLLATPKAKEI-AQKYYNC
T_theileri_Tth.4.4250/1-116    -------------------------------------TEKPWQC
T_theileri_Tth.97.1070/1-422   --------------------------------------------
T_theileri_Tth.7.4640/1-495    -PLRNKER---------------KSVTVSSSNTLRE-TRSHYNC
T_theileri_Tth.71.1060/1-278   ------------------TAEER---------------------
T_theileri_Tth.11.3640/1-145   --------------------------------------------
T_theileri_Tth.12.1860/1-209   ------------------NSNYTGYEELSSPEINKK-VKEHYGC
T_theileri_Tth.13.3150/1-731   -GVRG-------------RE---FVWMINTSKAKEV-ARNYFNC
T_theileri_Tth.12.1040/1-712   -GGIF------YG-K---YD---GNRVKVNGRAGVE-AKKHYNC
T_theileri_Tth.120.1090/1-644  -FGNG------------------MRRVVNSNQTMKM-AQKYYNC
T_theileri_Tth.107.1060/1-758  -SLRD-------------KE---DVFVVSADNTRRE-TMKHYNC
T_theileri_Tth.71.1000/1-211   --------------------------------------------
T_theileri_Tth.19.2170/1-599   -RLRG-------------LE---KVPFLKCPKLVEK-AKKHYNC
T_theileri_Tth.132.1040/1-509  -LHLGW-----------------VIAVLESNIVKEK-VREHYGC
T_theileri_Tth.39.1020/1-195   --------------------------------------------
T_theileri_Tth.14.1420/1-170   --------------------------------------------
T_theileri_Tth.101.1040/1-225  --------------------------------------------
T_theileri_Tth.39.1010/1-861   -IDPS------PKQKQMDGR---KFHKVVSPQTLAK-LNEHYGC
T_theileri_Tth.27.1400/1-341   -GLRG-------------KS---NVLVVASPRTLEK-TRAHFNC
T_theileri_Tth.54.1360/1-370   -----------------------------SQNTRRA-AKLHYDC
T_theileri_Tth.13.3260/1-705   ------------------LRGKGKVTAVDSEIIQEM-MREHYDC
T_theileri_Tth.101.1030/1-620  -------------------LSEETRALVKSTRTVEK-AQKHYSC
T_theileri_Tth.54.1310/1-800   -TLRE-------------KT---IISLVTSDNTRRE-TMKHYKC
T_theileri_Tth.54.1370/1-309   --------------------------------------------
T_theileri_Tth.32.2270/1-628   ------------------LTS--NTRAVHSDIMLEK-MREHYDC
T_theileri_Tth.17.2210/1-197   --------------------------------------------
T_theileri_Tth.36.2100/1-574   -STDD-------------KA---EFFVVASENTRRE-AMNHYKC
T_theileri_Tth.10.2530/1-248   --------------------------------------------
T_theileri_Tth.165.1000/1-309  NNSDK------------------VVYFVKSSNTNEV-VRKHYAC
T_theileri_Tth.29.1010/1-643   ------------------LRGKGKVTAVDSEIMQEM-MREHYDC
T_theileri_Tth.13.3140/1-290   --------------------------------------------
T_theileri_Tth.124.1000/1-107  --------------------------------------------
T_theileri_Tth.4.4470/1-974    ------------------GK---SVHLVTAPAVIAA-AQTHLSC
T_theileri_Tth.24.1440/1-461   --LRGA-----------------KRKVVISSKTVEK-AQKHYNC
T_theileri_Tth.44.1540/1-561   -NVER-------------KV---YAVVVSSTRTKEE-AQKHYNC
T_theileri_Tth.91.1000/1-302   -NLRGK-----------------SRVVVVSAKLQEE-AQKHYGC
T_theileri_Tth.26.2510/1-186   --------------------------------------------
T_theileri_Tth.83.1090/1-415   --FPG-------------EK---IKYQVNSKMTRNV-SQRHYNC
T_theileri_Tth.61.1050/1-209   --------------------------------------------
T_theileri_Tth.54.1330/1-803   -NLRN-------------KS---NVALVNSENTLRE-TMDHYNC
T_theileri_Tth.87.1010/1-146   --------------------------------------------
```

```
                                           710        720        730        740
T_theileri_Tth.124.1010/1-124  --------------------------------------------
T_theileri_Tth.52.1320/1-345   HN------------------------------------------
T_theileri_Tth.46.1750/1-879   KDGEKNK---LEGLYLENE-------------------------
T_theileri_Tth.85.1050/1-656   NT--------AQGMELEYE-------------------------
T_theileri_Tth.63.1230/1-856   DG----E---VKGLYLENY-------------------------
T_theileri_Tth.19.2160/1-140   --------------------------------------------
T_theileri_Tth.2.1500/1-116    --------------------------------------------
T_theileri_Tth.11.2420/1-601   PE--------IPGMELEDE-------------------------
T_theileri_Tth.4.4280/1-686    PN--------ASGVELENQ-------------------------
T_theileri_Tth.5.3690/1-642    NE--------LEGLELGYV-------------------------
T_theileri_Tth.24.2350/1-645   ST--------LEGMELQLG-------------------------
T_theileri_Tth.36.2130/1-775   DT--------LKGMELQNM-------------------------
T_theileri_Tth.13.2470/1-271   DT--------MEGVEL----------------------------
T_theileri_Tth.38.1990/1-923   QG---DE---VKGLCLEND-------------------------
T_theileri_Tth.17.2230/1-737   KD--------APGLELEDQ-------------------------
T_theileri_Tth.36.1950/1-407   VS--------DRGLPLQSV-------------------------
T_theileri_Tth.71.1100/1-161   --------------------------------------------
T_theileri_Tth.117.1060/1-252  SN--------APGLELEDQ-------------------------
T_theileri_Tth.4.4250/1-116    RP------------------------------------------
T_theileri_Tth.97.1070/1-422   --------------------------------------------
T_theileri_Tth.7.4640/1-495    NT--------TEGMELEDQ-------------------------
T_theileri_Tth.71.1060/1-278   --------------------------------------------
T_theileri_Tth.11.3640/1-145   --------------------------------------------
T_theileri_Tth.12.1860/1-209   HE--------DIGMKLDNS-------------------------
T_theileri_Tth.13.3150/1-731   PN--------ASGLEMEST-------------------------
T_theileri_Tth.12.1040/1-712   -----PA---FDVMYLTQR-------------------------
T_theileri_Tth.120.1090/1-644  SE--------LEGLELAYA-------------------------
T_theileri_Tth.107.1060/1-758  DS--------AEGMELQGV-------------------------
T_theileri_Tth.71.1000/1-211   --------------------------------------------
T_theileri_Tth.19.2170/1-599   SS--------LDGVEMEND-------------------------
T_theileri_Tth.132.1040/1-509  PD--------ISTMELEGR-------------------------
T_theileri_Tth.39.1020/1-195   --------------------------------------------
T_theileri_Tth.14.1420/1-170   --------------------------------------------
T_theileri_Tth.101.1040/1-225  --------------------------------------------
T_theileri_Tth.39.1010/1-861   EKG--NE---LKGLYLENQ-------------------------
T_theileri_Tth.27.1400/1-341   GT--------APGMELEDE-------------------------
T_theileri_Tth.54.1360/1-370   VS--------DRGLPLQSV-------------------------
T_theileri_Tth.13.3260/1-705   HLD-------ITGMYMEDE-------------------------
T_theileri_Tth.101.1030/1-620  PD--------LEGMELETI-------------------------
T_theileri_Tth.54.1310/1-800   DS--------AKGMELQRL-------------------------
T_theileri_Tth.54.1370/1-309   --------------------------------------------
T_theileri_Tth.32.2270/1-628   HLH-------ITGMYMEDE-------------------------
T_theileri_Tth.17.2210/1-197   --------------------------------------------
T_theileri_Tth.36.2100/1-574   DT--------LKGMELQNM-------------------------
T_theileri_Tth.10.2530/1-248   --------------------------------------------
T_theileri_Tth.165.1000/1-309  KNS-------TNGMRLDMV-------------------------
T_theileri_Tth.29.1010/1-643   HLD-------ITGMYMEDE-------------------------
T_theileri_Tth.13.3140/1-290   --------------------------------------------
T_theileri_Tth.124.1000/1-107  --------------------------------------------
T_theileri_Tth.4.4470/1-974    PT--------LDGVELEDE-------------------------
T_theileri_Tth.24.1440/1-461   SN--------LKGMELKFV-------------------------
T_theileri_Tth.44.1540/1-561   TT--------IDGMELEDE-------------------------
T_theileri_Tth.91.1000/1-302   ED--------LEGVELAYA-------------------------
T_theileri_Tth.26.2510/1-186   --------------------------------------------
T_theileri_Tth.83.1090/1-415   EE--------SRGAFLEEE-------------------------
T_theileri_Tth.61.1050/1-209   --------------------------------------------
T_theileri_Tth.54.1330/1-803   DS--------TEGMELRVV-------------------------
T_theileri_Tth.87.1010/1-146   --------------------------------------------
```

```
                                    750       760       770       780       790
T_theileri_Tth.124.1010/1-124   --------------------------------------------
T_theileri_Tth.52.1320/1-345    --------------------------------------------
T_theileri_Tth.46.1750/1-879    --------------------------------------------
T_theileri_Tth.85.1050/1-656    --------------------------------------------
T_theileri_Tth.63.1230/1-856    --------------------------------------------
T_theileri_Tth.19.2160/1-140    --------------------------------------------
T_theileri_Tth.2.1500/1-116     --------------------------------------------
T_theileri_Tth.11.2420/1-601    --------------------------------------------
T_theileri_Tth.4.4280/1-686     --------------------------------------------
T_theileri_Tth.5.3690/1-642     --------------------------------------------
T_theileri_Tth.24.2350/1-645    --------------------------------------------
T_theileri_Tth.36.2130/1-775    ----------------------------AFVQV-----------
T_theileri_Tth.13.2470/1-271    --------------------------------------------
T_theileri_Tth.38.1990/1-923    --------------------------------------------
T_theileri_Tth.17.2230/1-737    --------------------------------------------
T_theileri_Tth.36.1950/1-407    --------------------------------------------
T_theileri_Tth.71.1100/1-161    --------------------------------------------
T_theileri_Tth.117.1060/1-252   --------------------------------------------
T_theileri_Tth.4.4250/1-116     --------------------------------------------
T_theileri_Tth.97.1070/1-422    --------------------------------------------
T_theileri_Tth.7.4640/1-495     --------------------------------------------
T_theileri_Tth.71.1060/1-278    --------------------------------------------
T_theileri_Tth.11.3640/1-145    --------------------------------------------
T_theileri_Tth.12.1860/1-209    --------------------------------------------
T_theileri_Tth.13.3150/1-731    --------------------------------------------
T_theileri_Tth.12.1040/1-712    --------------------------------------------
T_theileri_Tth.120.1090/1-644   --------------------------------------------
T_theileri_Tth.107.1060/1-758   ----------------------------AFVSG-----------
T_theileri_Tth.71.1000/1-211    --------------------------------------------
T_theileri_Tth.19.2170/1-599    --------------------------------------------
T_theileri_Tth.132.1040/1-509   --------------------------------------------
T_theileri_Tth.39.1020/1-195    --------------------------------------------
T_theileri_Tth.14.1420/1-170    --------------------------------------------
T_theileri_Tth.101.1040/1-225   --------------------------------------------
T_theileri_Tth.39.1010/1-861    --------------------------------------------
T_theileri_Tth.27.1400/1-341    --------------------------------------------
T_theileri_Tth.54.1360/1-370    --------------------------------------------
T_theileri_Tth.13.3260/1-705    --------------------------------------------
T_theileri_Tth.101.1030/1-620   --------------------------------------------
T_theileri_Tth.54.1310/1-800    ----------------------------VIEQK-----------
T_theileri_Tth.54.1370/1-309    --------------------------------------------
T_theileri_Tth.32.2270/1-628    --------------------------------------------
T_theileri_Tth.17.2210/1-197    --------------------------------------------
T_theileri_Tth.36.2100/1-574    ----------------------------AFVQV-----------
T_theileri_Tth.10.2530/1-248    --------------------------------------------
T_theileri_Tth.165.1000/1-309   --------------------------------------------
T_theileri_Tth.29.1010/1-643    --------------------------------------------
T_theileri_Tth.13.3140/1-290    --------------------------------------------
T_theileri_Tth.124.1000/1-107   --------------------------------------------
T_theileri_Tth.4.4470/1-974     --------------------------------------------
T_theileri_Tth.24.1440/1-461    --------------------------------------------
T_theileri_Tth.44.1540/1-561    --------------------------------------------
T_theileri_Tth.91.1000/1-302    --------------------------------------------
T_theileri_Tth.26.2510/1-186    --------------------------------------------
T_theileri_Tth.83.1090/1-415    --------------------------------------------
T_theileri_Tth.61.1050/1-209    --------------------------------------------
T_theileri_Tth.54.1330/1-803    ----------------------------LLPRQLRTKGNNGRLH
T_theileri_Tth.87.1010/1-146    --------------------------------------------
```

```
                                        800       810       820       830
T_theileri_Tth.124.1010/1-124   --------------------------------------------
T_theileri_Tth.52.1320/1-345    --------------------------------------------
T_theileri_Tth.46.1750/1-879    --------------------------------------------
T_theileri_Tth.85.1050/1-656    --------------------------------------------
T_theileri_Tth.63.1230/1-856    --------------------------------------------
T_theileri_Tth.19.2160/1-140    --------------------------------------------
T_theileri_Tth.2.1500/1-116     --------------------------------------------
T_theileri_Tth.11.2420/1-601    --------------------------------------------
T_theileri_Tth.4.4280/1-686     --------------------------------------------
T_theileri_Tth.5.3690/1-642     --------------------------------------------
T_theileri_Tth.24.2350/1-645    --------------------------------------------
T_theileri_Tth.36.2130/1-775    ----------------------------------PALPVQAAAI
T_theileri_Tth.13.2470/1-271    --------------------------------------------
T_theileri_Tth.38.1990/1-923    --------------------------------------------
T_theileri_Tth.17.2230/1-737    --------------------------------------------
T_theileri_Tth.36.1950/1-407    ------------------------------------------AS
T_theileri_Tth.71.1100/1-161    --------------------------------------------
T_theileri_Tth.117.1060/1-252   --------------------------------------------
T_theileri_Tth.4.4250/1-116     --------------------------------------------
T_theileri_Tth.97.1070/1-422    --------------------------------------------
T_theileri_Tth.7.4640/1-495     --------------------------------------------
T_theileri_Tth.71.1060/1-278    --------------------------------------------
T_theileri_Tth.11.3640/1-145    --------------------------------------------
T_theileri_Tth.12.1860/1-209    --------------------------------------------
T_theileri_Tth.13.3150/1-731    --------------------------------------------
T_theileri_Tth.12.1040/1-712    --------------------------------------------
T_theileri_Tth.120.1090/1-644   --------------------------------------------
T_theileri_Tth.107.1060/1-758   ----------------------------------TTLTARASTI
T_theileri_Tth.71.1000/1-211    --------------------------------------------
T_theileri_Tth.19.2170/1-599    --------------------------------------------
T_theileri_Tth.132.1040/1-509   --------------------------------------------
T_theileri_Tth.39.1020/1-195    --------------------------------------------
T_theileri_Tth.14.1420/1-170    --------------------------------------------
T_theileri_Tth.101.1040/1-225   --------------------------------------------
T_theileri_Tth.39.1010/1-861    --------------------------------------------
T_theileri_Tth.27.1400/1-341    --------------------------------------------
T_theileri_Tth.54.1360/1-370    ------------------------------------------AS
T_theileri_Tth.13.3260/1-705    --------------------------------------------
T_theileri_Tth.101.1030/1-620   --------------------------------------------
T_theileri_Tth.54.1310/1-800    -------------SKEEKGPLSGTSSVVSSQSAPTPHSAQAAAI
T_theileri_Tth.54.1370/1-309    --------------------------------------------
T_theileri_Tth.32.2270/1-628    --------------------------------------------
T_theileri_Tth.17.2210/1-197    --------------------------------------------
T_theileri_Tth.36.2100/1-574    -----REAGSRRNDRL------------------------SSTV
T_theileri_Tth.10.2530/1-248    --------------------------------------------
T_theileri_Tth.165.1000/1-309   --------------------------------------------
T_theileri_Tth.29.1010/1-643    --------------------------------------------
T_theileri_Tth.13.3140/1-290    --------------------------------------------
T_theileri_Tth.124.1000/1-107   --------------------------------------------
T_theileri_Tth.4.4470/1-974     --------------------------------------------
T_theileri_Tth.24.1440/1-461    --------------------------------------------
T_theileri_Tth.44.1540/1-561    --------------------------------------------
T_theileri_Tth.91.1000/1-302    --------------------------------------------
T_theileri_Tth.26.2510/1-186    --------------------------------------------
T_theileri_Tth.83.1090/1-415    --------------------------------------------
T_theileri_Tth.61.1050/1-209    --------------------------------------------
T_theileri_Tth.54.1330/1-803    EGRPNPREGRQRSDKRTRAH------------PHVQDGRRSLAP
T_theileri_Tth.87.1010/1-146    --------------------------------------------
```

```
                                       840       850       860       870
T_theileri_Tth.124.1010/1-124  --------------------------------------------
T_theileri_Tth.52.1320/1-345   --------------------------------------------
T_theileri_Tth.46.1750/1-879   --------------------------------------------
T_theileri_Tth.85.1050/1-656   --------------------------------------------
T_theileri_Tth.63.1230/1-856   --------------------------------------------
T_theileri_Tth.19.2160/1-140   --------------------------------------------
T_theileri_Tth.2.1500/1-116    --------------------------------------------
T_theileri_Tth.11.2420/1-601   --------------------------------------------
T_theileri_Tth.4.4280/1-686    --------------------------------------------
T_theileri_Tth.5.3690/1-642    --------------------------------------------
T_theileri_Tth.24.2350/1-645   --------------------------------------------
T_theileri_Tth.36.2130/1-775   LAQVASVQEDNEVGESH---------------SNVMDDRLTGEE
T_theileri_Tth.13.2470/1-271   --------------------------------------------
T_theileri_Tth.38.1990/1-923   --------------------------------------------
T_theileri_Tth.17.2230/1-737   --------------------------------------------
T_theileri_Tth.36.1950/1-407   YATVAYMRSSADTPES----------------------------
T_theileri_Tth.71.1100/1-161   --------------------------------------------
T_theileri_Tth.117.1060/1-252  --------------------------------------------
T_theileri_Tth.4.4250/1-116    --------------------------------------------
T_theileri_Tth.97.1070/1-422   --------------------------------------------
T_theileri_Tth.7.4640/1-495    -----------TVPRGHNVARPRPAAGGPGGLRAPGLRGGLLNG
T_theileri_Tth.71.1060/1-278   --------------------------------------------
T_theileri_Tth.11.3640/1-145   --------------------------------------------
T_theileri_Tth.12.1860/1-209   --------------------------------------------
T_theileri_Tth.13.3150/1-731   --------------------------------------------
T_theileri_Tth.12.1040/1-712   --------------------------------------------
T_theileri_Tth.120.1090/1-644  --------------------------------------------
T_theileri_Tth.107.1060/1-758  LAQVASIQEEYVLGKSH---------------SNVMDGRLPGEE
T_theileri_Tth.71.1000/1-211   --------------------------------------------
T_theileri_Tth.19.2170/1-599   --------------------------------------------
T_theileri_Tth.132.1040/1-509  --------------------------------------------
T_theileri_Tth.39.1020/1-195   --------------------------------------------
T_theileri_Tth.14.1420/1-170   --------------------------------------------
T_theileri_Tth.101.1040/1-225  --------------------------------------------
T_theileri_Tth.39.1010/1-861   --------------------------------------------
T_theileri_Tth.27.1400/1-341   --------------------------------------------
T_theileri_Tth.54.1360/1-370   YATVAYMRSSADTPER----------------------------
T_theileri_Tth.13.3260/1-705   --------------------------------------------
T_theileri_Tth.101.1030/1-620  --------------------------------------------
T_theileri_Tth.54.1310/1-800   LAQVASVQEEYELGESH---------------SNVIDGRLTAKE
T_theileri_Tth.54.1370/1-309   --------------------------------------------
T_theileri_Tth.32.2270/1-628   --------------------------------------------
T_theileri_Tth.17.2210/1-197   --------------------------------------------
T_theileri_Tth.36.2100/1-574   RALTASVQEEYELGKSH---------------SNVIDGRLTGKE
T_theileri_Tth.10.2530/1-248   --------------------------------------------
T_theileri_Tth.165.1000/1-309  --------------------------------------------
T_theileri_Tth.29.1010/1-643   --------------------------------------------
T_theileri_Tth.13.3140/1-290   --------------------------------------------
T_theileri_Tth.124.1000/1-107  --------------------------------------------
T_theileri_Tth.4.4470/1-974    --------------------------------------------
T_theileri_Tth.24.1440/1-461   --------------------------------------------
T_theileri_Tth.44.1540/1-561   --------------------------------------------
T_theileri_Tth.91.1000/1-302   --------------------------------------------
T_theileri_Tth.26.2510/1-186   --------------------------------------------
T_theileri_Tth.83.1090/1-415   --------------------------------------------
T_theileri_Tth.61.1050/1-209   --------------------------------------------
T_theileri_Tth.54.1330/1-803   RVVTASVQEEYELGKSH---------------SNAIDGRLTGKE
T_theileri_Tth.87.1010/1-146   --------------------------------------------
```

```
                                               890       900       910       920
                                                 |    |    |    |    |    |    |
T_theileri_Tth.124.1010/1-124 --------------------------------------------
T_theileri_Tth.52.1320/1-345  --------------------------------------------
T_theileri_Tth.46.1750/1-879  --------------------------------------------
T_theileri_Tth.85.1050/1-656  --------------------------------------------
T_theileri_Tth.63.1230/1-856  --------------------------------------------
T_theileri_Tth.19.2160/1-140  --------------------------------------------
T_theileri_Tth.2.1500/1-116   --------------------------------------------
T_theileri_Tth.11.2420/1-601  --------------------------------------------
T_theileri_Tth.4.4280/1-686   --------------------------------------------
T_theileri_Tth.5.3690/1-642   --------------------------------------------
T_theileri_Tth.24.2350/1-645  --------------------------------------------
T_theileri_Tth.36.2130/1-775  SVGSDVQERREEE---GSSAAKFTHNVHHSSDV--------LSK
T_theileri_Tth.13.2470/1-271  --------------------------------------------
T_theileri_Tth.38.1990/1-923  --------------------------------------------
T_theileri_Tth.17.2230/1-737  --------------------------------------------
T_theileri_Tth.36.1950/1-407  --TEMMSLRKPMN-------------------------------
T_theileri_Tth.71.1100/1-161  --------------------------------------------
T_theileri_Tth.117.1060/1-252 --------------------------------------------
T_theileri_Tth.4.4250/1-116   --------------------------------------------
T_theileri_Tth.97.1070/1-422  --------------------------------------------
T_theileri_Tth.7.4640/1-495   PLPPNSPPGFRGNVERRGGILRGPTDTQSLRTPPGTGLRNPPST
T_theileri_Tth.71.1060/1-278  --------------------------------------------
T_theileri_Tth.11.3640/1-145  --------------------------------------------
T_theileri_Tth.12.1860/1-209  --------------------------------------------
T_theileri_Tth.13.3150/1-731  --------------------------------------------
T_theileri_Tth.12.1040/1-712  --------------------------------------------
T_theileri_Tth.120.1090/1-644 --------------------------------------------
T_theileri_Tth.107.1060/1-758 SVGSVVQERREEE---GSSAAKFTQNVHHSSHV--------FSK
T_theileri_Tth.71.1000/1-211  --------------------------------------------
T_theileri_Tth.19.2170/1-599  --------------------------------------------
T_theileri_Tth.132.1040/1-509 --------------------------------------------
T_theileri_Tth.39.1020/1-195  --------------------------------------------
T_theileri_Tth.14.1420/1-170  --------------------------------------------
T_theileri_Tth.101.1040/1-225 --------------------------------------------
T_theileri_Tth.39.1010/1-861  --------------------------------------------
T_theileri_Tth.27.1400/1-341  --------------------------------------------
T_theileri_Tth.54.1360/1-370  --TEMMSLRKPVN-------------------------------
T_theileri_Tth.13.3260/1-705  --------------------------------------------
T_theileri_Tth.101.1030/1-620 --------------------------------------------
T_theileri_Tth.54.1310/1-800  SVDSVVQERREEE---GSSDAKFTQNVHHSSNV--------FSK
T_theileri_Tth.54.1370/1-309  --------------------------------------------
T_theileri_Tth.32.2270/1-628  --------------------------------------------
T_theileri_Tth.17.2210/1-197  --------------------------------------------
T_theileri_Tth.36.2100/1-574  AVGSVVQERRGEE---GSSAAKFTQNIHHSSNV--------WSK
T_theileri_Tth.10.2530/1-248  --------------------------------------------
T_theileri_Tth.165.1000/1-309 --------------------------------------------
T_theileri_Tth.29.1010/1-643  --------------------------------------------
T_theileri_Tth.13.3140/1-290  --------------------------------------------
T_theileri_Tth.124.1000/1-107 --------------------------------------------
T_theileri_Tth.4.4470/1-974   --------------------------------------------
T_theileri_Tth.24.1440/1-461  --------------------------------------------
T_theileri_Tth.44.1540/1-561  --------------------------------------------
T_theileri_Tth.91.1000/1-302  --------------------------------------------
T_theileri_Tth.26.2510/1-186  --------------------------------------------
T_theileri_Tth.83.1090/1-415  --------------------------------------------
T_theileri_Tth.61.1050/1-209  --------------------------------------------
T_theileri_Tth.54.1330/1-803  SVGSVVQERRGEE---GFSSAKFTQNVHHSSNV--------FSK
T_theileri_Tth.87.1010/1-146  --------------------------------------------
```

```
                                        930       940       950       960
T_theileri_Tth.124.1010/1-124  --------------------------------------------
T_theileri_Tth.52.1320/1-345   --------------------------------------------
T_theileri_Tth.46.1750/1-879   ------------------------------------------QN
T_theileri_Tth.85.1050/1-656   --------------------------------------------
T_theileri_Tth.63.1230/1-856   -------------------------------------------I
T_theileri_Tth.19.2160/1-140   --------------------------------------------
T_theileri_Tth.2.1500/1-116    --------------------------------------------
T_theileri_Tth.11.2420/1-601   --------------------------------------------
T_theileri_Tth.4.4280/1-686    --------------------------------------------
T_theileri_Tth.5.3690/1-642    --------------------------------------------
T_theileri_Tth.24.2350/1-645   --------------------------------------------
T_theileri_Tth.36.2130/1-775   RRSLYLQATVAESP--NKCTTNEEGDTATDE-ECIVKTTTVLPN
T_theileri_Tth.13.2470/1-271   --------------------------------------------
T_theileri_Tth.38.1990/1-923   -------------------------------------------D
T_theileri_Tth.17.2230/1-737   --------------------------------------------
T_theileri_Tth.36.1950/1-407   -QKLNGIDVVPAGE-----------SSSSYQGLQVVEK-----Y
T_theileri_Tth.71.1100/1-161   --------------------------------------------
T_theileri_Tth.117.1060/1-252  --------------------------------------------
T_theileri_Tth.4.4250/1-116    --------------------------------------------
T_theileri_Tth.97.1070/1-422   --------------------------------------------
T_theileri_Tth.7.4640/1-495    NPAGTNAVIHPVQRNSAGAGAAALLAEVEETPNSNEKVVAVEKH
T_theileri_Tth.71.1060/1-278   --------------------------------------------
T_theileri_Tth.11.3640/1-145   --------------------------------------------
T_theileri_Tth.12.1860/1-209   --------------------------------------------
T_theileri_Tth.13.3150/1-731   --------------------------------------------
T_theileri_Tth.12.1040/1-712   -------------------------------------------G
T_theileri_Tth.120.1090/1-644  --------------------------------------------
T_theileri_Tth.107.1060/1-758  RHSLHVQAAVTASEPSSECTDDKKVETEKGSKMCIVEA---PTF
T_theileri_Tth.71.1000/1-211   --------------------------------------------
T_theileri_Tth.19.2170/1-599   --------------------------------------------
T_theileri_Tth.132.1040/1-509  ------------------------------------------DK
T_theileri_Tth.39.1020/1-195   --------------------------------------------
T_theileri_Tth.14.1420/1-170   --------------------------------------------
T_theileri_Tth.101.1040/1-225  --------------------------------------------
T_theileri_Tth.39.1010/1-861   ------------------------------------------GI
T_theileri_Tth.27.1400/1-341   --------------------------------------------
T_theileri_Tth.54.1360/1-370   -QKLNGIDVVPAEE-----------SSSSFQGLQVVEK-----Y
T_theileri_Tth.13.3260/1-705   --------------------------------------------
T_theileri_Tth.101.1030/1-620  --------------------------------------------
T_theileri_Tth.54.1310/1-800   RRSLYAQTAMMASEPSSQCATRRKELEGVADTMKVVET---TRF
T_theileri_Tth.54.1370/1-309   --------------------------------------------
T_theileri_Tth.32.2270/1-628   --------------------------------------------
T_theileri_Tth.17.2210/1-197   --------------------------------------------
T_theileri_Tth.36.2100/1-574   RRSIYLQATVAESP--SGCTPGKEVDTATGK-QCIVETTQIPTN
T_theileri_Tth.10.2530/1-248   --------------------------------------------
T_theileri_Tth.165.1000/1-309  --------------------------------------------
T_theileri_Tth.29.1010/1-643   --------------------------------------------
T_theileri_Tth.13.3140/1-290   --------------------------------------------
T_theileri_Tth.124.1000/1-107  --------------------------------------------
T_theileri_Tth.4.4470/1-974    --------------------------------------------
T_theileri_Tth.24.1440/1-461   --------------------------------------------
T_theileri_Tth.44.1540/1-561   --------------------------------------------
T_theileri_Tth.91.1000/1-302   --------------------------------------------
T_theileri_Tth.26.2510/1-186   --------------------------------------------
T_theileri_Tth.83.1090/1-415   --------------------------------------------
T_theileri_Tth.61.1050/1-209   --------------------------------------------
T_theileri_Tth.54.1330/1-803   RRSLYLQATVAESQ--STCSPGKPVETEKGGTRCIVET---KRF
T_theileri_Tth.87.1010/1-146   --------------------------------------------
```

```
                                        970       980       990       1000      1010
T_theileri_Tth.124.1010/1-124   --------------------------------------------
T_theileri_Tth.52.1320/1-345    --------------------------------------------
T_theileri_Tth.46.1750/1-879    NGL------PPHWERRIAKDE--LMSTYSDT---FGTTGMYYSA
T_theileri_Tth.85.1050/1-656    -RWGEVGAVSSHWNYRVAKDD--LMSSRFTV------GGMYYTA
T_theileri_Tth.63.1230/1-856    SWT-------SHWRSRIAKDE--LMSTYIGE-----PTGMYYTA
T_theileri_Tth.19.2160/1-140    --------------------------------------------
T_theileri_Tth.2.1500/1-116     --------------------------------------------
T_theileri_Tth.11.2420/1-601    ---GGKGTLSSHWERRNAIDE--LMSGLIGK--------GYYTV
T_theileri_Tth.4.4280/1-686     ----GAASARSHIEERNARED--FMAPGAMI--------GRYSV
T_theileri_Tth.5.3690/1-642     --AKAHTPAWSHLAWRNTNDD--LMSSMYYY------GAMHYSA
T_theileri_Tth.24.2350/1-645    ----SGDKVQSHWTHRNAKDE--LMAPTDSL------GAGYYTA
T_theileri_Tth.36.2130/1-775    NEYNEGVMTRSHWSRRIAKDE--LMVGLVGA--------GYYTA
T_theileri_Tth.13.2470/1-271    --------------------------------------------
T_theileri_Tth.38.1990/1-923    QWT------PSHWEREFAKDE--LMSTYIGE-----PTGMYYTA
T_theileri_Tth.17.2230/1-737    -----TMSAVSHFEMRNINDE--MMTPVSSV-------GGAYSA
T_theileri_Tth.36.1950/1-407    KVSPGSMVYSSHWSRRIAKDE--LMVGLVGA--------GYYTA
T_theileri_Tth.71.1100/1-161    --------------------------------------------
T_theileri_Tth.117.1060/1-252   -----TSSVLSHFEMRNVNEE--IMSPVSSV-------GGAYSA
T_theileri_Tth.4.4250/1-116     -------------TQRGAKVQYFVLPEEAKS-------------
T_theileri_Tth.97.1070/1-422    ----------------------------------------TVDS
T_theileri_Tth.7.4640/1-495     MDIGKKNNLFSHWKRRNAKDE--LMAGVVGA--------GYYTA
T_theileri_Tth.71.1060/1-278    --------------------------------------------
T_theileri_Tth.11.3640/1-145    --------------------------------------------
T_theileri_Tth.12.1860/1-209    -PPESENDADTHFDGRVARND--LMAPLHGS----QHGEMSYTA
T_theileri_Tth.13.3150/1-731    -----GWGSASHFSIRVAKGD--MMGPGYSP--------LVYSE
T_theileri_Tth.12.1040/1-712    PSV------SNHWGWHIAKDE--LMSPYTAD-----SSGMFYTN
T_theileri_Tth.120.1090/1-644   -TVEAHILNLSHLAWRNAVDD--LMSSRYNF------GAMHYSA
T_theileri_Tth.107.1060/1-758   MSNHGSKMTRSHWSRRIAKDE--LMAGLVGA--------GYYTA
T_theileri_Tth.71.1000/1-211    --------------------------------------------
T_theileri_Tth.19.2170/1-599    ---GDDFTALSHWDVRSAKDE--LMAAFSGI--------GYYTA
T_theileri_Tth.132.1040/1-509   GHLFENYVFYPHIKRHFAKDE--LMSTDLER-----PSGMYYTN
T_theileri_Tth.39.1020/1-195    ----------IKWKPWKSQIL--ITSNYFINVLGIAIIKIVYQV
T_theileri_Tth.14.1420/1-170    --------------------------------------------
T_theileri_Tth.101.1040/1-225   -----------------------MAPREEE------GVGIYYTA
T_theileri_Tth.39.1010/1-861    EKN------SSHWERYIAKDE--LMSTYIGE-----PSGMYYTA
T_theileri_Tth.27.1400/1-341    ---GGEGTALSHWERRNAKDE--LMSGIAGA--------GYYTA
T_theileri_Tth.54.1360/1-370    KVSPGSMGYSSHWSRRIAKDE--LMVGLVGA--------GYYTA
T_theileri_Tth.13.3260/1-705    ----GNGRRKLHWERRIAKDE--LMSPYTGE-----PSGMFYTN
T_theileri_Tth.101.1030/1-620   -----DGEIQPHWSRRIAKDE--LMAPSDYN-----YGAGYYTA
T_theileri_Tth.54.1310/1-800    PLHYRGFRTLSHWSRRIAKDE--LMVGLVGA--------GYYTA
T_theileri_Tth.54.1370/1-309    --------------------------------------------
T_theileri_Tth.32.2270/1-628    ----GDGRSKLHWERRIAKDE--LMSPYTEE-----PSGMYYTN
T_theileri_Tth.17.2210/1-197    --------------------------------------------
T_theileri_Tth.36.2100/1-574    TQYNAGKMIRSHWSRRIAKDE--LMVGLVGA--------GYYTA
T_theileri_Tth.10.2530/1-248    --------------------------------------------
T_theileri_Tth.165.1000/1-309   ----SNNVPSSHWDRHVAKDE--LMSTYGNE-----SSGMFYTA
T_theileri_Tth.29.1010/1-643    ----GDGRRKLHWERRIAKDE--LMSPYTEE-----PSGMYYTN
T_theileri_Tth.13.3140/1-290    --------------------------------------------
T_theileri_Tth.124.1000/1-107   --------------------------------------------
T_theileri_Tth.4.4470/1-974     ---GGSGTAWTHWERRGWMDE--LMAGIPSR--------AAISA
T_theileri_Tth.24.1440/1-461    -----NGEVFSYWDARNAKDE--LMSSARSS------SAGYYTT
T_theileri_Tth.44.1540/1-561    --DTAKGTVLSHWKMRNAKDE--LMSTRQMA------SAGLYTV
T_theileri_Tth.91.1000/1-302    --DIGMTEVASHWAYRVAKDE--LMSSDTTY------GAGYYTA
T_theileri_Tth.26.2510/1-186    --------------------------------------------
T_theileri_Tth.83.1090/1-415    ---GGTWNMVSHWERRDAKDE--LMSVYFDL-----PGAMLYTA
T_theileri_Tth.61.1050/1-209    --------------------------------------------
T_theileri_Tth.54.1330/1-803    LPENMEIITQSHWSRRNAKDE--LMVGLVGA--------GYYTA
T_theileri_Tth.87.1010/1-146    --------------------------------------------
```

```
                                       1020      1030      1040      1050
T_theileri_Tth.124.1010/1-124   --------------------------------------------
T_theileri_Tth.52.1320/1-345    --------------------------------------------
T_theileri_Tth.46.1750/1-879    LTLAAFHSMPFYSANFEKAEPMSWGKQYICDLFKG-KKDLTQT-
T_theileri_Tth.85.1050/1-656    LTMSVFDGLPYYSVNWDMAEPMAWGNKSNCDLLDNNFD-KVTFI
T_theileri_Tth.63.1230/1-856    LTLATFHEMPFYSANFTMAEPMSWGNQSICEFLQG-KKDPAE--
T_theileri_Tth.19.2160/1-140    --------------------------------------------
T_theileri_Tth.2.1500/1-116     --------------------------------------------
T_theileri_Tth.11.2420/1-601    LTLAAFHDTGFFKANFSMAEPMKWGNNSGCQFLKEPCI--KNEI
T_theileri_Tth.4.4280/1-686     LSLAIFDSMPFYKANFDKAEPMKWGNKSGCKFLDEKCIKDGKS-
T_theileri_Tth.5.3690/1-642     LTMSVFDDLPFYTVNWGMEESITWGNQSGCELFKSECK--DEGA
T_theileri_Tth.24.2350/1-645    LTMSTFEDMGYYRANWGMEEPMGWGNHSGCDFFEALCI--VNNA
T_theileri_Tth.36.2130/1-775    ITLGAFADLGYYKVNWTMAEQMSWGNNSNCEFLNNKCVNSGE--
T_theileri_Tth.13.2470/1-271    --------------------------------------------
T_theileri_Tth.38.1990/1-923    LTLAAFHGMSFYRANFSMAEPMGWSNQSICEFLQG-KKDPTQ--
T_theileri_Tth.17.2230/1-737    LTLAVFDDMPFYKANFSRAEPLRWANNSGCDFLEKKCI--ENKK
T_theileri_Tth.36.1950/1-407    LTLGAFADLGYYKVNWTMAEQMSWGNNSGCGLLEKKCVEGGR--
T_theileri_Tth.71.1100/1-161    --------------------------------------------
T_theileri_Tth.117.1060/1-252   LTLAVFDDMPFYKANFSRAEPLRWANNSGCDFLEKKCI--ENKT
T_theileri_Tth.4.4250/1-116     --------------------------------------------
T_theileri_Tth.97.1070/1-422    RTIGVISIKTQNNADFKKAEPMSWGNETICDFLEGKKKNELL--
T_theileri_Tth.7.4640/1-495     LTMAFFADM-----------------------------------
T_theileri_Tth.71.1060/1-278    --------------------------------------------
T_theileri_Tth.11.3640/1-145    --------------------------------------------
T_theileri_Tth.12.1860/1-209    LTLAAFESTGHYKVDYFKAEDM--GGKKSCEYLKGE--------
T_theileri_Tth.13.3150/1-731    LSLAVFDSMPFYKANFSMAEPLVWGKNAGCDFLEKKCL--ENGV
T_theileri_Tth.12.1040/1-712    LTLGAFDDMPYYRANFSMAEPMSWGKQLGCNFVGK-DKAEQRDK
T_theileri_Tth.120.1090/1-644   LTMSVFDGLPFYTVNWGMEESMSWGNQSGCDLFKSECK--AEDA
T_theileri_Tth.107.1060/1-758   ITMGAFADLRYYKVNWAMAEQMSWGNNSGCGLLEDKCVNSGE--
T_theileri_Tth.71.1000/1-211    -----------------MAETMRWGNHSGCGFLSGDCVEKSEG-
T_theileri_Tth.19.2170/1-599    LTMAAFECTRYYKANWGMEETMSWGKDAGCSLHTDKCIKENVT-
T_theileri_Tth.132.1040/1-509   LTLAVFDSMPFYKADFSRAETMSWGRKAGCGFIKGKCVEWGIS-
T_theileri_Tth.39.1020/1-195    ELVG----------------------------------------
T_theileri_Tth.14.1420/1-170    --------------------------------------------
T_theileri_Tth.101.1040/1-225   LTMAFFEDLGYYKANWGMEEPMSWGHKKGCSFINEACI--KNGI
T_theileri_Tth.39.1010/1-861    LTLAVFHAMPFYSADFDNAETMSWGNQSICELLEG-KKKVPTK-
T_theileri_Tth.27.1400/1-341    LTMGAMEDLGFYKAVWGMEEPMSWGRMSGCKLLTDKCV--ENGV
T_theileri_Tth.54.1360/1-370    LTLGAFADLGYYKVNWTMAEQMSWGNNSGCGLLEDKCVEGGS--
T_theileri_Tth.13.3260/1-705    LTLAAFHSLPFYSANFNMAEPMSWGKKSGCNLLHKTCDRYRDEL
T_theileri_Tth.101.1030/1-620   LTMALFEDLQYYKANWGMEEQMSWGNQSGCPFLEKDCR--KKHN
T_theileri_Tth.54.1310/1-800    LTLGAFADLGYYKVNWTMAEQMSWGNKAGCEFLNDKCVKEGS--
T_theileri_Tth.54.1370/1-309    -----------------MAEQMSWGNNSNCEFLNKKCVNNGE--
T_theileri_Tth.32.2270/1-628    LTLAAFHSLPFYSANFNMAEPMSWGNKSGCNLLDKTCDRYRDEL
T_theileri_Tth.17.2210/1-197    --------------------------------------------
T_theileri_Tth.36.2100/1-574    LTLGAFADLGYYKVNWTMAEQMSWGNNSGCELLNNKCVNSGN--
T_theileri_Tth.10.2530/1-248    --------------------------------------------
T_theileri_Tth.165.1000/1-309   LTLATFHDMKFYQANFSMAESM----------------------
T_theileri_Tth.29.1010/1-643    LTLAAFHSMPFYKAHFNMAEPMSWGKKSGCDLLHNTCTGGNDEL
T_theileri_Tth.13.3140/1-290    --------------------------------------------
T_theileri_Tth.124.1000/1-107   --------------------------------------------
T_theileri_Tth.4.4470/1-974     LTLAFFSSLNLYTVNMEYNETMSWSYHAGCPFLEERCVANAQ--
T_theileri_Tth.24.1440/1-461    LTMALFEDLQYYKANWGMEEQMSWGNQSGCDFLQEKCM--ENNK
T_theileri_Tth.44.1540/1-561    LTLSTFHDMGFYRAKFDMAESMTWGSNIGCSLMDGMCVNESDTI
T_theileri_Tth.91.1000/1-302    LTMGAFDGLPYYTANWGMEEPMTWGNGSGCEFIRGNCI--TNGQ
T_theileri_Tth.26.2510/1-186    --------------------------------------------
T_theileri_Tth.83.1090/1-415    FTMAAFEDMKYFRANWGKEETMSWGKDAGCEFQYINCVEDKKS-
T_theileri_Tth.61.1050/1-209    --------------------------------------------
T_theileri_Tth.54.1330/1-803    LTLGAFADLGYYKVNWTMAEQMSWGNKAGCEFLNDKCVNDGE--
T_theileri_Tth.87.1010/1-146    --------------------------------------------
```

```
                                1060      1070      1080      1090
T_theileri_Tth.124.1010/1-124   --------------------------------------------
T_theileri_Tth.52.1320/1-345    ---------------------------------GTRNRT-----
T_theileri_Tth.46.1750/1-879    -----------HYPDMFCEDD------TKTILKCTSDRFALGIC
T_theileri_Tth.85.1050/1-656    E----------KYPKMFCK--------SDGSYSCTTDRSALGVC
T_theileri_Tth.63.1230/1-856    ----T------RHPNVFCEENE-----KV-TLQCTSDRFALGMC
T_theileri_Tth.19.2160/1-140    --------------------------------------------
T_theileri_Tth.2.1500/1-116     --------------------------------------------
T_theileri_Tth.11.2420/1-601    S----------SFPDLFCN-----WNGKNSLPSCTYDHLSLGYC
T_theileri_Tth.4.4280/1-686     -----------SFPEMVCD-----TPFPKVLNLCTLDRLALGYC
T_theileri_Tth.5.3690/1-642     T----------KYPGRFCD-----INEGEVVYSCTSDRLGFGRC
T_theileri_Tth.24.2350/1-645    A----------KYPEMFCS---------ESVSRCTTDRSSPGYC
T_theileri_Tth.36.2130/1-775    ----T------KFSNMFCTTKSTV---GTESLQCTSDRQSMGSC
T_theileri_Tth.13.2470/1-271    --------------------------------------------
T_theileri_Tth.38.1990/1-923    ----T------DRPYMFCKENE-----KKEILQCTSDRFALGMC
T_theileri_Tth.17.2230/1-737    S----------NFPEIFCN----TRYIIKDYFQCTYDRMALGVC
T_theileri_Tth.36.1950/1-407    ----T------KFPDMFCTTESTE---GSAGLQCTSDRQSLGTC
T_theileri_Tth.71.1100/1-161    --------------------------------------FALGHC
T_theileri_Tth.117.1060/1-252   S----------NFPDIFCT----TTHIIKDYFQCTYDRMALGVC
T_theileri_Tth.4.4250/1-116     --------------------------------------------
T_theileri_Tth.97.1070/1-422    ----K------KHPNMFCKEDE-------KTLQCTSDRFALGMC
T_theileri_Tth.7.4640/1-495     --------------------------------------------
T_theileri_Tth.71.1060/1-278    --------------------------------------------
T_theileri_Tth.11.3640/1-145    --------------------------------------------
T_theileri_Tth.12.1860/1-209    --------------------------------------------
T_theileri_Tth.13.3150/1-731    S----------PNPEFFCN-----KFRSTNIDFCTPDRMGLGYC
T_theileri_Tth.12.1040/1-712    I--TR------ENPNMFCTG-------EKQGLQCTSDRFALGVC
T_theileri_Tth.120.1090/1-644   T----------TISTKICP-------KDKNVFSCTSDRLGYGVC
T_theileri_Tth.107.1060/1-758   ----T------QLPNMFCTSTSTD---SSESLQCTSDRQSLGRC
T_theileri_Tth.71.1000/1-211    -----------KHSDVSCDSSS--VATRTTTLQCTSDRFALGHC
T_theileri_Tth.19.2170/1-599    -----------EFPDLFCT-------ALSETPVCTSDRRALGIC
T_theileri_Tth.132.1040/1-509   -----------RFPDMFCVDES-------YDLHCTTDRTALGKC
T_theileri_Tth.39.1020/1-195    --------------------------------------------
T_theileri_Tth.14.1420/1-170    --------------------------------------------
T_theileri_Tth.101.1040/1-225   S----------RYPETFCN---------TSTTRCTSNRYALGEC
T_theileri_Tth.39.1010/1-861    ----T------DYPNMFCEEDA-----ENVILQCTSDRFALGIC
T_theileri_Tth.27.1400/1-341    T----------AYPDMFCT-------ADSDTLRCTSDRRALGIC
T_theileri_Tth.54.1360/1-370    ----T------KFPDMFCTSKPGS---LPAGLQCTSDRQSLGSC
T_theileri_Tth.13.3260/1-705    M----------KYTNMFCDENG-------PVLQCTSDRFALGTC
T_theileri_Tth.101.1030/1-620   E----------FYDIIID----------EKFSRCTSDRTAYGKF
T_theileri_Tth.54.1310/1-800    ----T------KFPDMFCTTESTE---GPESLQCTSDRQSLGSC
T_theileri_Tth.54.1370/1-309    ----T------KFPDMFCTTKSGDNPNIPAGLQCTSDRQSLGTC
T_theileri_Tth.32.2270/1-628    M----------KYTSMFCDENE-------PVLQCTSDRFALGMC
T_theileri_Tth.17.2210/1-197    -----------KCRKMFCDEIK-------PALQCTSDRFALGMC
T_theileri_Tth.36.2100/1-574    ----P------DFSKMFCTTESTD---FPAGLKCTSDRQSLGSC
T_theileri_Tth.10.2530/1-248    --------------------------------------------
T_theileri_Tth.165.1000/1-309   --------------------------------------------
T_theileri_Tth.29.1010/1-643    M----------NHTSMFCKENE-------PVLQCTSDRFALGMC
T_theileri_Tth.13.3140/1-290    ---------------MFCT----ATVRIKGYLQCTYDRMTLGQC
T_theileri_Tth.124.1000/1-107   --------------------------------------------
T_theileri_Tth.4.4470/1-974     -----------RMGFEWCN-------DTSTAQLCTFDRLAVGLC
T_theileri_Tth.24.1440/1-461    V----------RYPQYFCN---------ETISGCTSDRTAYGKC
T_theileri_Tth.44.1540/1-561    DSWTKKKWKVPNFPSLFCD-------TVRHRFPCTTDRLGVGTC
T_theileri_Tth.91.1000/1-302    S----------KFPDMFCK---------EAVSRCTSDRFSVGVC
T_theileri_Tth.26.2510/1-186    --------------------------------------------
T_theileri_Tth.83.1090/1-415    -----------TYPSMFCN-------STSETLKCTSDRFALGEC
T_theileri_Tth.61.1050/1-209    --------------------------------------------
T_theileri_Tth.54.1330/1-803    ----T------KFPNMFCTTEATD---GTESLQCTSDRQSLGSC
T_theileri_Tth.87.1010/1-146    --------------------------------------------
```

```
                                          1110      1120      1130      1140
T_theileri_Tth.124.1010/1-124 --------------------------------------------
T_theileri_Tth.52.1320/1-345  --------------------------------------------
T_theileri_Tth.46.1750/1-879  STKTNRN-----KLTGRY-QYFKD--EFRQKDAK-DLMDGVPFI
T_theileri_Tth.85.1050/1-656  DPSEYYRDSTFNEIRAVFVR------------------------
T_theileri_Tth.63.1230/1-856  LTKN-N---LG-QLPNGY-QYFN----DISVEWDGDLMDGDAII
T_theileri_Tth.19.2160/1-140  --------------------------------------------
T_theileri_Tth.2.1500/1-116   --------------------------------------------
T_theileri_Tth.11.2420/1-601  TLDSFTE-----DRPPEY-RYFTD--PKRGGSS--SFMDFCPHV
T_theileri_Tth.4.4280/1-686   TLKEHDY-----VLAEEY-RYFPDN-NRYGGSN--NFMDFCPIV
T_theileri_Tth.5.3690/1-642   KGVPEDRYT--------------------------KENEKCNVA
T_theileri_Tth.24.2350/1-645  SVPASYHWE--------------------------SEKNLCPFT
T_theileri_Tth.36.2130/1-775  SSIEAKLV-NK-GIPKQF-QYFSEN-NKVGSEES-EQMDYCPFI
T_theileri_Tth.13.2470/1-271  --------------------------------------------
T_theileri_Tth.38.1990/1-923  LTKK-N---LG-QLPNGY-QYFHG--EDSETTMD-DLMDGYPII
T_theileri_Tth.17.2230/1-737  GTKRYPE-----KLEPQF-QYLWN--AHYGGDK--VYMDYCPYV
T_theileri_Tth.36.1950/1-407  T---IQTH-ER-DLPGQF-QYFSD--GKLGGNKT-DLMDYCPII
T_theileri_Tth.71.1100/1-161  TRSLSSQP------------------ISVISAEHRGEKRECAVV
T_theileri_Tth.117.1060/1-252 GTRSYPE-----ELEPHF-RYLRN--AHLGGSK--VHMDYCPYV
T_theileri_Tth.4.4250/1-116   --------------------------------------------
T_theileri_Tth.97.1070/1-422  LTKENF----E-RLPDEY-RHFNG--KELE-LKD-DLMDGYTVI
T_theileri_Tth.7.4640/1-495   --------------------------------------------
T_theileri_Tth.71.1060/1-278  --------------------------------------------
T_theileri_Tth.11.3640/1-145  --------------------------------------------
T_theileri_Tth.12.1860/1-209  --------------------------------------------
T_theileri_Tth.13.3150/1-731  TLKAHYR-----ILPKHF-QYFKK--LFWGGDL--SALDYCPYV
T_theileri_Tth.12.1040/1-712  SKKDTP---PK-AISEEY-HYFEK--A-AGIGRG-DLFNDFSVI
T_theileri_Tth.120.1090/1-644 TPLPDDRY---------------------------PDKKKCNVA
T_theileri_Tth.107.1060/1-758 SSIGASPV-KN-DLPVGF-QYFSENTKKVGSEES-EQMDYCPFI
T_theileri_Tth.71.1000/1-211  TRSLSSQP------------------ISVFSAEHRGEKRECAVV
T_theileri_Tth.19.2170/1-599  TLGVYNA-----DLPEEY-RYFTN--PRVGGVYE-YMTDYCPFI
T_theileri_Tth.132.1040/1-509 SLQFLPWD----YVQSEF-NYFPR--YPRGSAYS-DWMDFCPAI
T_theileri_Tth.39.1020/1-195  --------------------------------------------
T_theileri_Tth.14.1420/1-170  --------------------------------------------
T_theileri_Tth.101.1040/1-225 ESLEDLDAD--------------------------NEIDFCPII
T_theileri_Tth.39.1010/1-861  SKTER-----N-KLPEGY-EYVKD--ENTNSEAN-DLMNGYKFI
T_theileri_Tth.27.1400/1-341  RIGTYTS-----SLPTQY-QYFAN--PTVGSSAS-NLMDYCPYI
T_theileri_Tth.54.1360/1-370  G---IQTY-RN-PLPGHF-QYFSD--GKLGGNKA-DLMDYCPII
T_theileri_Tth.13.3260/1-705  SSKSLPG-----TLPGEY-HYFTK-----TAGQN-EMTNGCPII
T_theileri_Tth.101.1030/1-620 LLNERLSK---------------------------EYQRICNLS
T_theileri_Tth.54.1310/1-800  -SITTTTD-GN-PLPEQF-QYFSEN-KKVGSRPP-QIMDYCPFI
T_theileri_Tth.54.1370/1-309  SSIEAKKT-VD-NLAEHF-QYFSEN-NKVGSEES-EQMDYCPII
T_theileri_Tth.32.2270/1-628  SSKSLPG-----NLPKVY-HYFTK-----TAGQN-EMTNGCPIV
T_theileri_Tth.17.2210/1-197  SKEGDLH-----KFHVDY-FLFSA--ITTKRAS--EWTDGYPII
T_theileri_Tth.36.2100/1-574  SSIGANPV-AK-PLPEQF-QYFSGNTNKVGSEES-EQMDFCPFI
T_theileri_Tth.10.2530/1-248  --------------------------------------------
T_theileri_Tth.165.1000/1-309 --------------------------------------------
T_theileri_Tth.29.1010/1-643  SSISVLD-----TLPEVY-SYFTK-----TAGQN-EMTNGCPII
T_theileri_Tth.13.3140/1-290  LRRTYPE-----VIEPYF-RYLSN--AHLGGNK--VNMDCCPYV
T_theileri_Tth.124.1000/1-107 --------------------------------------------
T_theileri_Tth.4.4470/1-974   NTGLVP------NLPPYF-QYFPA--EPMRAGVT-PLMDYCPIV
T_theileri_Tth.24.1440/1-461  NPPSRLNE---------------------------FFEDNCEVT
T_theileri_Tth.44.1540/1-561  EPEENKWW---------------------------TQPEGCPVV
T_theileri_Tth.91.1000/1-302  SSLNGFYGK--------------------------DEADECPYA
T_theileri_Tth.26.2510/1-186  --------------------------------------------
T_theileri_Tth.83.1090/1-415  SLTTYNN-----DLPLKY-RYFLRARKEAGSAE--TLTDRCPII
T_theileri_Tth.61.1050/1-209  --------------------------------------------
T_theileri_Tth.54.1330/1-803  SSFGAKKK-DNKPLPEHF-RYFSEN-NKVGSQEP-AQMDYCPFI
T_theileri_Tth.87.1010/1-146  --------------------------------------------
```

```
                                     1150      1160      1170      1180
T_theileri_Tth.124.1010/1-124  --------------------------------------------
T_theileri_Tth.52.1320/1-345   --------------------------------------------
T_theileri_Tth.46.1750/1-879   RPL-----E---------GTAC----EDGNQ-SL--MPGSIVD-
T_theileri_Tth.85.1050/1-656   --------GRSDNSGTSGTLLC----FAENT-IE--MEGSVVG-
T_theileri_Tth.63.1230/1-856   RPF-----I---------GTDC----EDGNE-TL--LPGSLFG-
T_theileri_Tth.19.2160/1-140   --------------------------------------------
T_theileri_Tth.2.1500/1-116    --------------------------------------------
T_theileri_Tth.11.2420/1-601   DED-----P---------KKSC----TSGNA-MD--MKGSYIG-
T_theileri_Tth.4.4280/1-686    GRHR--------------EPWC----TKSFS-LN--GEGSYVG-
T_theileri_Tth.5.3690/1-642    EMEHPP--RGDKT-----SLLC----SEIPM-FN--ISGSIFDM
T_theileri_Tth.24.2350/1-645   KPD------YGKFEEKSRYVFC----FSDDV-PA--LDGSLMG-
T_theileri_Tth.36.2130/1-775   KAK-----V---------GYSC----INGVQ-SK--MPESKIG-
T_theileri_Tth.13.2470/1-271   --------------------------------------------
T_theileri_Tth.38.1990/1-923   RPL-----N---------GTVC----EGGDE-NL--LPGSLLG-
T_theileri_Tth.17.2230/1-737   QKVA--------------QGGC----TDGSR-ST--IVGSFIG-
T_theileri_Tth.36.1950/1-407   VGK-----R---------ETSC----TDGDQ-SQ--MPGSLIA-
T_theileri_Tth.71.1100/1-161   KATA--------------ATVC----ENGSE-AH--MPGSRIG-
T_theileri_Tth.117.1060/1-252  EKVS--------------RGGC----TDGSR-WT--IIGSFVG-
T_theileri_Tth.4.4250/1-116    --------------------------------QRRLSSGRPVG-
T_theileri_Tth.97.1070/1-422   KPL-----E---------STAC----EDGNE-TL--LPGSLLS-
T_theileri_Tth.7.4640/1-495    --------------------------------------------
T_theileri_Tth.71.1060/1-278   --------------------------------------------
T_theileri_Tth.11.3640/1-145   --------------------------------------------
T_theileri_Tth.12.1860/1-209   --------------------------------------------
T_theileri_Tth.13.3150/1-731   EPYG--------------YTRC----ATSLY-LQ--SRGSFIG-
T_theileri_Tth.12.1040/1-712   KPF-----R---------ETMC----EDGNE-TL--MPGSIVD-
T_theileri_Tth.120.1090/1-644  ELVEPP--RGDKT-----SLLC----SEKPQ-LN--ISGSIFER
T_theileri_Tth.107.1060/1-758  KAL-----P---------QKSC----IDGEQ-NQ--MPGSVIA-
T_theileri_Tth.71.1000/1-211   KATA--------------ATVC----ENGSE-AH--MPGSRIG-
T_theileri_Tth.19.2170/1-599   SPI-----P---------YGDC----KASIS-YG--YLGSRIG-
T_theileri_Tth.132.1040/1-509  EPT-----F---------ETSC----EFGYE-RE--MPGSIVS-
T_theileri_Tth.39.1020/1-195   --------------------------------------------
T_theileri_Tth.14.1420/1-170   --------------------------------LQ--SRGSFIG-
T_theileri_Tth.101.1040/1-225  VGST----VIQEGGDSQPTSFC----TFGDE-SL--LTGSLIG-
T_theileri_Tth.39.1010/1-861   RSL-----E---------GTGC----EDGKE-DL--LPGSVLG-
T_theileri_Tth.27.1400/1-341   AEY-----T---------NTGC----TNGEL-LA--MPGSRVS-
T_theileri_Tth.54.1360/1-370   VGK-----R---------ETSC----TDGEQ-SK--MPGSVIA-
T_theileri_Tth.13.3260/1-705   KPL-----K---------ETTC----ESGDV-AL--MPGSIVS-
T_theileri_Tth.101.1030/1-620  ESYY-----IGRITSED-PLYC----NDESKAET--LPGSIKG-
T_theileri_Tth.54.1310/1-800   MAL-----P---------QKSC----TDGDQ-SK--MPGSVIA-
T_theileri_Tth.54.1370/1-309   MAK-----K---------GYSC----INGEQRNK--MPGSLIV-
T_theileri_Tth.32.2270/1-628   KPL-----K---------ETTC----ESGDV-AL--MPGSIVS-
T_theileri_Tth.17.2210/1-197   KAI-----P---------QTNC----EKGQL-KY--MTGSVVG-
T_theileri_Tth.36.2100/1-574   MAK-----K---------GYSC----ITGEEKNK--MPGSVIA-
T_theileri_Tth.10.2530/1-248   --------------------------------------------
T_theileri_Tth.165.1000/1-309  --------------------------------------------
T_theileri_Tth.29.1010/1-643   KPL-----K---------KTTC----ENGDV-AL--MPGSIVS-
T_theileri_Tth.13.3140/1-290   EKVR--------------YGDC----TDGRR-ST--IIGSFVG-
T_theileri_Tth.124.1000/1-107  --------------------------------------------
T_theileri_Tth.4.4470/1-974    VGY-----G---------NRQCTQPVESTSD-DA--LYGFYFG-
T_theileri_Tth.24.1440/1-461   EKY-----IISMRKSWT-YSYC----TGIYS-DA--LPGSIIG-
T_theileri_Tth.44.1540/1-561   VPY-----FDSLDSNTYISGIC----ADESA-KP--TMGSIKG-
T_theileri_Tth.91.1000/1-302   SPE-----ATQTSEGLM-RAPC----TLMLT-NK--IPGSVTG-
T_theileri_Tth.26.2510/1-186   --------------------------------------------
T_theileri_Tth.83.1090/1-415   APTA--------------ATVC----TNGKE-ES--MPGSRVG-
T_theileri_Tth.61.1050/1-209   --------------------------------------------
T_theileri_Tth.54.1330/1-803   KAK-----H---------GYSC----ISGEQ-SN--MPESKIG-
T_theileri_Tth.87.1010/1-146   --------------------------------------------
```

```
                               1190      1200      1210      1220      1230
T_theileri_Tth.124.1010/1-124  --------------------------------------------
T_theileri_Tth.52.1320/1-345   -------------------------------------CSGILRR
T_theileri_Tth.46.1750/1-879   KMSRCLHVKEP-VLQVVGSS----GTNFT----VGGVCAKVECD
T_theileri_Tth.85.1050/1-656   NDSFCLDTDEY-TISGRTGK-------------STGVCARVRCE
T_theileri_Tth.63.1230/1-856   RDSRCLKAEGL-KITG----SS--DTHLN----IAGVCAKVKCD
T_theileri_Tth.19.2160/1-140   --------------------------------------------
T_theileri_Tth.2.1500/1-116    --------------------------------------------
T_theileri_Tth.11.2420/1-601   PNSRCVKGVDL-RLSNVP---------------LGDVCVNTLCE
T_theileri_Tth.4.4280/1-686    PDSRCVKGEGL-KYMKRA---------------IGDVCVKTDCN
T_theileri_Tth.5.3690/1-642    DDSLCLDTEKY-NADVVIEVRENQRETGT--VSLTGICAQVSCE
T_theileri_Tth.24.2350/1-645   NDSWCLDGESL-HVNFD---------DDTVKENVAGVCALVSCD
T_theileri_Tth.36.2130/1-775   INSRCVEGKDL-KTDK----------KAA----VGAVCVEVSCK
T_theileri_Tth.13.2470/1-271   --------------------------------------------
T_theileri_Tth.38.1990/1-923   RDSRCLNAKGL-FLRD---PNG--HRSHT----MGGVCAKVKCD
T_theileri_Tth.17.2230/1-737   PDARCVKGEDL-KYRTRS---------------IGDVCVNTKCE
T_theileri_Tth.36.1950/1-407   ANSRCVQGENL-IAD-----------DTA----VGAVCVEVSCK
T_theileri_Tth.71.1100/1-161   NTSRCLKGDAL-RLRD--------VSTIDIHSIIGDICANVKCE
T_theileri_Tth.117.1060/1-252  PN------------------------------------------
T_theileri_Tth.4.4250/1-116    --------------------------------------------
T_theileri_Tth.97.1070/1-422   NTSRCLNAEGV-VLRD---SNG--HRSLN----IAGVCAKVKCD
T_theileri_Tth.7.4640/1-495    --------------------------------------------
T_theileri_Tth.71.1060/1-278   --------------------------------------------
T_theileri_Tth.11.3640/1-145   --------------------------------------------
T_theileri_Tth.12.1860/1-209   --------------------------------------------
T_theileri_Tth.13.3150/1-731   ENARCVKGVEL-NFSGQP---------------IGDVCVNTKCD
T_theileri_Tth.12.1040/1-712   KMSRCLNVKDP-QIKD----VN--GNGVT----VQGICAKVKCE
T_theileri_Tth.120.1090/1-644  VDSFCLDTEEY-NAGSMPGVFD--DETIP--LFATGICARVSCE
T_theileri_Tth.107.1060/1-758  NNSRCVQGKEL-KTIK---------DEAA----VGAVCVEVSCK
T_theileri_Tth.71.1000/1-211   NTSRCLKGDAL-RLRD--------VSTIDIHSVIGDICANVKCE
T_theileri_Tth.19.2170/1-599   PSSWCLKGESL-MLNGTI---------------VGDVCAEVQCD
T_theileri_Tth.132.1040/1-509  STSRCLKVDFV-RLRNKGVMSN-----------VKGICAEVKCE
T_theileri_Tth.39.1020/1-195   --------------------------------------------
T_theileri_Tth.14.1420/1-170   ENARCVKGVEL-NFSGKS---------------VGDVCVNTKCD
T_theileri_Tth.101.1040/1-225  PDSWCLDGEGL-QVQNT----------RKSVESLSGVCAQVSCD
T_theileri_Tth.39.1010/1-861   QDSRCLNLENP-LEFK--KGDN--GNGVM----FQGICAKVKCN
T_theileri_Tth.27.1400/1-341   SSSRCVKGDSL-HLLFVP---------------IGDVCAEIKCK
T_theileri_Tth.54.1360/1-370   ANSRCVQGENL-KAK-----------NAA----IGAVCVEVSCK
T_theileri_Tth.13.3260/1-705   NMSRCLNVNKPLEFSEGDQ-----RNGVT----AKGICAKVKCE
T_theileri_Tth.101.1030/1-620  PNSWCLDGEGL-KVKE--------NGETI--QSVNGVCAQVSCD
T_theileri_Tth.54.1310/1-800   ANSRCVQGENL-KAK-----------NAA----VGAVCVEVSCK
T_theileri_Tth.54.1370/1-309   NNSRCVEGNEL-KTIK---------DEAA----VGAVCVEVSCK
T_theileri_Tth.32.2270/1-628   KMSRCLNVDKPLEFSEGVQ-----RNGVT----VKGICAKVKCE
T_theileri_Tth.17.2210/1-197   KESRCLKGEDL-TLKMP-------SGKPP----VGDICADVKCE
T_theileri_Tth.36.2100/1-574   NNSRCVEGKGL-KTKE----------EAA----VGAVCVEVSCK
T_theileri_Tth.10.2530/1-248   --------------------------------------------
T_theileri_Tth.165.1000/1-309  --------------------------------------------
T_theileri_Tth.29.1010/1-643   KMSRCLNVKEP-EFSEGVQ-----KNGVT----VKGICAKVKCE
T_theileri_Tth.13.3140/1-290   PDARCVKGNEL-KYRTTP---------------IGDVCVNTKCE
T_theileri_Tth.124.1000/1-107  --------------------------------------------
T_theileri_Tth.4.4470/1-974    TQSRCVPTHNM-VKKGYTIDDNNPR------------CLMVRCR
T_theileri_Tth.24.1440/1-461   PDSWCLDAEEL-KVNG--------PDNKV--SDVNGVCARVLCD
T_theileri_Tth.44.1540/1-561   SDAWCVDGKDL-RYKY-----SFDDISVR----LDSVCAKIKCE
T_theileri_Tth.91.1000/1-302   NDSWCLDGESL-VVKDAT------DTDKN----VGGVCARVSCE
T_theileri_Tth.26.2510/1-186   --------------------------------------------
T_theileri_Tth.83.1090/1-415   --------------------------------------------
T_theileri_Tth.61.1050/1-209   --------------------------------------------
T_theileri_Tth.54.1330/1-803   INSRCVEGKDL-KADN----------NAA----VGAVCVEVSCK
T_theileri_Tth.87.1010/1-146   --------------------------------------------
```

```
                                        1240      1250      1260      1270
T_theileri_Tth.124.1010/1-124  --------------------------------------------
T_theileri_Tth.52.1320/1-345   FLSKAK--------------------------------------
T_theileri_Tth.46.1750/1-879   -NANKVVRVQLKG-----NENN-------WHEC-KNDDKTTFEM
T_theileri_Tth.85.1050/1-656   ---GNEVEVMYAG-----SKE--------WHKCP-ENSTLN---
T_theileri_Tth.63.1230/1-856   -NEKKEVSVQLKG-----YTEN---NKE-WHEC-SDDKA---NV
T_theileri_Tth.19.2160/1-140   --------------------------------------------
T_theileri_Tth.2.1500/1-116    --------------------------------------------
T_theileri_Tth.11.2420/1-601   ---NGKLKVQFQL-----DSE--------WYLCE-ENKII----
T_theileri_Tth.4.4280/1-686    -KEKKKLRVQFYE-----SQT--------WYECE-EGKKI----
T_theileri_Tth.5.3690/1-642    ---EGKVRVIYNG-----ISE--------WQNCSKEGEI-----
T_theileri_Tth.24.2350/1-645   -ESSRTVKVQYNG-----SDE--------WHDCP-EGKSIE---
T_theileri_Tth.36.2130/1-775   -FNK--VIVRYNG-----NDK--------WYNC-PEGKNLT---
T_theileri_Tth.13.2470/1-271   --------------------------------------------
T_theileri_Tth.38.1990/1-923   -NANKKVSVQLKG-----YDKK---KELIWHEC-NDDNA---AV
T_theileri_Tth.17.2230/1-737   ---EGKLSVQFLG-----DDENK------WYECK-EGEHVI---
T_theileri_Tth.36.1950/1-407   -KNE--VSVRYSG-----NDN--------WYSC-PEGGRLA---
T_theileri_Tth.71.1100/1-161   ---GGKVSVQYKG-----DDD--------WYEC-KEGSSISPS-
T_theileri_Tth.117.1060/1-252  --------------------------------------------
T_theileri_Tth.4.4250/1-116    --------------------------------------------
T_theileri_Tth.97.1070/1-422   -NKEEKVLVQYKG-----QSDTA------WHVC-RNDDD---KI
T_theileri_Tth.7.4640/1-495    --------------------------------------------
T_theileri_Tth.71.1060/1-278   --------------------------------------------
T_theileri_Tth.11.3640/1-145   --------------------------------------------
T_theileri_Tth.12.1860/1-209   --------------------------------------------
T_theileri_Tth.13.3150/1-731   ---NGTLSVQFLGDG---DDE--------WHVCK-EKEEI----
T_theileri_Tth.12.1040/1-712   -NGK--VLVHYKG-----NKEH-NGKEK-WEEC-TEKG----TI
T_theileri_Tth.120.1090/1-644  ---EGKVRVMYNG-----ISD--------WKNCSGEGDI-----
T_theileri_Tth.107.1060/1-758  -FKK--VIVRYSG-----NND--------WYSC-PEGKNLT---
T_theileri_Tth.71.1000/1-211   ---GGKVSVQYKG-----DDH--------WYEC-KEGSSISPS-
T_theileri_Tth.19.2170/1-599   ---NKSVQVRYYG-----DDK--------WYPCP-EGKVL----
T_theileri_Tth.132.1040/1-509  ---NGTLKVRYKG-----NKN--------WYEC-PEGDSIDNI-
T_theileri_Tth.39.1020/1-195   --------------------------------------------
T_theileri_Tth.14.1420/1-170   ---NGTLSVQFLGDG---DDK--------WHVCE-EKMEI----
T_theileri_Tth.101.1040/1-225  -EGRRTVEVQYKG-----SNT--------FKECP-EGTS-----
T_theileri_Tth.39.1010/1-861   -NKTRNVSVQLKG-----YEDE---EKN-WRQC-SDESA---TI
T_theileri_Tth.27.1400/1-341   ---KDMVQLRYIG-----DNT--------WYDCP-EGRSI----
T_theileri_Tth.54.1360/1-370   -KNE--VSVRYSG-----NNE--------WYSC-PEGGRLT---
T_theileri_Tth.13.3260/1-705   ---NGKVSVQY-------KEDTTK-----WIECSGENATIEM--
T_theileri_Tth.101.1030/1-620  -YEKRTVSVKYKGN----QDK--------WHDCP-EGTS-----
T_theileri_Tth.54.1310/1-800   -FNK--VIVRYSG-----NDN--------WYSC-PEGKNLT---
T_theileri_Tth.54.1370/1-309   -FNK--VIVRYSG-----NDK--------WHSC-PEGKNLT---
T_theileri_Tth.32.2270/1-628   ---NGKVHVQYKG-----KEDTTE-----WIECKKDGHEITL--
T_theileri_Tth.17.2210/1-197   ---NNKLLVKYSG-----SNA--------WQEC-KDGKINVT--
T_theileri_Tth.36.2100/1-574   -FKK--VIVRYSG-----NNE--------WHSC-HEGENLT---
T_theileri_Tth.10.2530/1-248   --------------------------------------------
T_theileri_Tth.165.1000/1-309  --------------------------------------------
T_theileri_Tth.29.1010/1-643   ---NGKVSVQY-------KEDTTE-----WIECSGENATIEM--
T_theileri_Tth.13.3140/1-290   --EEGKLSLQFQG-----DEE--------WYQCE-EGEHV----
T_theileri_Tth.124.1000/1-107  --------------------------------------------
T_theileri_Tth.4.4470/1-974    LGKQVEVFV---------GSY--------WLECPADGSA-----
T_theileri_Tth.24.1440/1-461   -YEKRTVKVQYKGKEG--END--------WYECP-ENGT-----
T_theileri_Tth.44.1540/1-561   ---DNQVKVKYLG-----NESN-------WILC-PENQYIT---
T_theileri_Tth.91.1000/1-302   ---EGKVMVMYAG-----NDN--------WYHCP-EGDF-----
T_theileri_Tth.26.2510/1-186   --------------------------------------------
T_theileri_Tth.83.1090/1-415   --------------------------------------------
T_theileri_Tth.61.1050/1-209   --------------------------------------------
T_theileri_Tth.54.1330/1-803   -FKK--VIVRYSG-----NDK--------WYSC-PEGENLT---
T_theileri_Tth.87.1010/1-146   --------------------------------------------
```

```
                              1280      1290      1300      1310
T_theileri_Tth.124.1010/1-124 --------------------------------------------
T_theileri_Tth.52.1320/1-345  --------------------------------------------
T_theileri_Tth.46.1750/1-879  ----------KDPAF--NGGTIL-CPKYEEVCTGWPETNSP-VI
T_theileri_Tth.85.1050/1-656  ---------VSSTPF--VSKKIT-CPKYSEVCTVAPNGSSLRPM
T_theileri_Tth.63.1230/1-856  E---L-----TDSEF--SGGTIK-CPKYEEVCIGLLETEAPSNI
T_theileri_Tth.19.2160/1-140  --------------------------------------------
T_theileri_Tth.2.1500/1-116   --------------------------------------------
T_theileri_Tth.11.2420/1-601  ---------RSKNPQ--WSGGIR-CPKYNEVCIDLPDVSGVLPP
T_theileri_Tth.4.4280/1-686   ---------TPKSFYL-WSGNII-CPKYEEVCDVLPNLSIVLDP
T_theileri_Tth.5.3690/1-642   ----IE---VKPAGLN-NTITIK-CPKYSEVCTIPTDGSSLLPR
T_theileri_Tth.24.2350/1-645  ---------AKSPTF--KSGNIK-CPKYDEVCTIAPNGGSRLAL
T_theileri_Tth.36.2130/1-775  ----V-----KESVL--Q-GKIV-CPKYADVCNTINKTLDESQG
T_theileri_Tth.13.2470/1-271  --------------------------------------------
T_theileri_Tth.38.1990/1-923  K---L-----EGSFF--ESGTIK-CPKYEEVCTGLPKTNS-LKI
T_theileri_Tth.17.2230/1-737  ---------SSNETN--WSGKII-CPKYADVCTKYPNITHEDTL
T_theileri_Tth.36.1950/1-407  ----V-----KGNVL--Q-GKIV-CPKYADVCNTINRVIDKGRG
T_theileri_Tth.71.1100/1-161  ----------VTSAF--SSGRIV-CPPYSEVCMDLPIQPPPTEA
T_theileri_Tth.117.1060/1-252 --------------------------------------------
T_theileri_Tth.4.4250/1-116   -------------------------P------------------
T_theileri_Tth.97.1070/1-422  E---L-----KGSVF--SSGTIK-CPNFKEVCTGLPKTKPL-TI
T_theileri_Tth.7.4640/1-495   --------------------------------------------
T_theileri_Tth.71.1060/1-278  --------------------------------------------
T_theileri_Tth.11.3640/1-145  --------------------------------------------
T_theileri_Tth.12.1860/1-209  --------------------------------------------
T_theileri_Tth.13.3150/1-731  ---------IPNKAN--WSGKII-CPRYDAVCTKIPDITIPLKE
T_theileri_Tth.12.1040/1-712  -N--L-----HGSVF--SGGTIV-CPKYEEVCTKLPETDPP-TV
T_theileri_Tth.120.1090/1-644 ----IE---VKPAGLN-NTIKIK-CPKYSEVCTIFSDGSSLLPL
T_theileri_Tth.107.1060/1-758 ----V-----KGDVL--K-GEIV-CPKYADVCNTINKTLDESKG
T_theileri_Tth.71.1000/1-211  ----------VTSVF--SSGRIV-CPPYSEVCMDLPIEPLPTEA
T_theileri_Tth.19.2170/1-599  ---------KPNNTF--TSGSIV-CPKYREVCTIAADGSSRIVK
T_theileri_Tth.132.1040/1-509 ----------NGSVF--DDGRIL-CPKYADVCTK----------
T_theileri_Tth.39.1020/1-195  --------------------------------------------
T_theileri_Tth.14.1420/1-170  ---------VPTTAN--LSGKII-CPRYDAVCTKLPDITIPLKE
T_theileri_Tth.101.1040/1-225 ----ID---VESSAFQ-SGGKIK-CPKYDEVCTITPDGRSRLSM
T_theileri_Tth.39.1010/1-861  K---L-----EDSEF--IGGTIR-CPKYEEVCTGLLGAELPTNI
T_theileri_Tth.27.1400/1-341  ---------IPALTF--TSGRIV-CPSRADVCIGLGDKLPDLSI
T_theileri_Tth.54.1360/1-370  ----V-----QGNVL--Q-GKIV-CPKYADVCNTINRVIDKERG
T_theileri_Tth.13.3260/1-705  ----------KDSKF--KGVSIV-CPKYEEVCTGLNETD-----
T_theileri_Tth.101.1030/1-620 ----IN---VESSAFQ-SGGKIK-CPKYDEVCTIALNGSSHVPF
T_theileri_Tth.54.1310/1-800  ----V-----NGDVL--N-GKIV-CPKYADVCNTINKTLDESQG
T_theileri_Tth.54.1370/1-309  ----V-----NGSVL--K-GEIV-CPKYADVCNTINKTLDELQG
T_theileri_Tth.32.2270/1-628  ----------EGSAF--KGGSIV-CPRYEEVCTGLNETD-----
T_theileri_Tth.17.2210/1-197  ----------GSSEF--TGGSIL-CPNYTEVCNNFTEIDVT--P
T_theileri_Tth.36.2100/1-574  ----V-----NGTAL--Q-GKIV-CPKYADVCNTINKTLDESQG
T_theileri_Tth.10.2530/1-248  --------------------------------------------
T_theileri_Tth.165.1000/1-309 --------------------------------------------
T_theileri_Tth.29.1010/1-643  ----------KDSKF--KGVNIV-CPKYEEVCTGLNETD-----
T_theileri_Tth.13.3140/1-290  ---------KSNSTN--WSGTIT-CPKYADVCTRYPNITNKEPL
T_theileri_Tth.124.1000/1-107 --------------------------------------------
T_theileri_Tth.4.4470/1-974   -------GVITIPAYTGYNGEIH-CERAEKVCSSTLFYNPDSAA
T_theileri_Tth.24.1440/1-461  ----IV---VESSAFE-DGGKIK-CPKYD---------------
T_theileri_Tth.44.1540/1-561  ---------LVSANFT-AESQIK-CPKYSEVCTMAPNGSSLVVP
T_theileri_Tth.91.1000/1-302  ----LE---PTSTSF--VSGKIK-CPKYREVCTIAPDGTSLLPL
T_theileri_Tth.26.2510/1-186  --------------------------------------------
T_theileri_Tth.83.1090/1-415  --------------------------------------------
T_theileri_Tth.61.1050/1-209  --------------------------------------------
T_theileri_Tth.54.1330/1-803  ----V-----NGTAL--Q-GKIV-CPKYADVCNTINKTLDESQG
T_theileri_Tth.87.1010/1-146  --------------------------------------------
```

```
                                                     1330      1340      1350      1360
T_theileri_Tth.124.1010/1-124 --------------------------------------------
T_theileri_Tth.52.1320/1-345  --------------------------------------------
T_theileri_Tth.46.1750/1-879  KLNSDKEESDGYYIVVNDGEKEGKPEHGQKPTHAPSVSSVFPKE
T_theileri_Tth.85.1050/1-656  VYPSWYEETTTAPNTGEEGS------------------------
T_theileri_Tth.63.1230/1-856  TFYNGTKFTDGY-VDLHDENKEE----------EEKTTKNFEPP
T_theileri_Tth.19.2160/1-140  --------------------------------------------
T_theileri_Tth.2.1500/1-116   --------------------------------------------
T_theileri_Tth.11.2420/1-601  VVLPFSYRLTTENNITELQKEER---------------------
T_theileri_Tth.4.4280/1-686   LPVKEDDDNPAPGNITWKIPESA---------------------
T_theileri_Tth.5.3690/1-642   IEPKRLPAPAI---TTTTENNES---------------------
T_theileri_Tth.24.2350/1-645  IDPPDDPTPSPDGGGGGGGTGNA---------------------
T_theileri_Tth.36.2130/1-775  PATDPDIVEEVQTSEPE---------------------------
T_theileri_Tth.13.2470/1-271  --------------------------------------------
T_theileri_Tth.38.1990/1-923  KYYSGKQDLEARDVVVNDDDEET----------GAATNTDVRAQ
T_theileri_Tth.17.2230/1-737  PEVTDADLKPAPANITWPIIKRE---------------------
T_theileri_Tth.36.1950/1-407  RPMKLGAYVD----------------------------------
T_theileri_Tth.71.1100/1-161  VV-MEEEVYNFTSSIN----------------------------
T_theileri_Tth.117.1060/1-252 --------------------------------------------
T_theileri_Tth.4.4250/1-116   --------------------------------------------
T_theileri_Tth.97.1070/1-422  KLYTGEDLEASDVVVNDDDEETVTATHIDDRTQAESDKRENGVP
T_theileri_Tth.7.4640/1-495   --------------------------------------------
T_theileri_Tth.71.1060/1-278  --------------------------------------------
T_theileri_Tth.11.3640/1-145  --------------------------------------------
T_theileri_Tth.12.1860/1-209  --------------------------------------------
T_theileri_Tth.13.3150/1-731  IPYQDGDDKPAPGNITWNMTETP---------------------
T_theileri_Tth.12.1040/1-712  EYEEYVEPQK--------NKEKK----------EEE---E----
T_theileri_Tth.120.1090/1-644 IEPKPLPAPVITTTTTTTENNES---------------------
T_theileri_Tth.107.1060/1-758 PATDPDPVKEVKTSETEVEKTTA---------------------
T_theileri_Tth.71.1000/1-211  VV-MQEEVYNVTASI-----------------------------
T_theileri_Tth.19.2170/1-599  KIIPEARHSSDTESSMGPTVTLESVDYRTEGEMSDKKEVAGFFE
T_theileri_Tth.132.1040/1-509 --------------------------------------------
T_theileri_Tth.39.1020/1-195  --------------------------------------------
T_theileri_Tth.14.1420/1-170  LPYQDGDDKPAPGNITWNMTETP---------------------
T_theileri_Tth.101.1040/1-225 N-------------------------------------------
T_theileri_Tth.39.1010/1-861  RFFSGKSITHVYDVDVNGKKEEK----------QEKKQIDAP--
T_theileri_Tth.27.1400/1-341  SI------------------------------------------
T_theileri_Tth.54.1360/1-370  RPMKLGAYVD----------------------------------
T_theileri_Tth.13.3260/1-705  ---------LPTIEYDDK-SDNS---------------------
T_theileri_Tth.101.1030/1-620 VDPHPAPPAAGP--------------------------------
T_theileri_Tth.54.1310/1-800  PATDPDIVEAIQTSLPE---------------------------
T_theileri_Tth.54.1370/1-309  PQKDPDPVKEVQTSQPE---------------------------
T_theileri_Tth.32.2270/1-628  ---------LPTIEYDDKSDNSN---------------------
T_theileri_Tth.17.2210/1-197  IKYDDDEKKKWMRRMRKRNSKWKKRL------------------
T_theileri_Tth.36.2100/1-574  PATNPDIVEEVQTSQP----------------------------
T_theileri_Tth.10.2530/1-248  --------------------------------------------
T_theileri_Tth.165.1000/1-309 --------------------------------------------
T_theileri_Tth.29.1010/1-643  ---------LPTIEYDEKNNDNS---------------------
T_theileri_Tth.13.3140/1-290  PEVTDKDNEPAPENITWPITKSE---------------------
T_theileri_Tth.124.1000/1-107 --------------------------------------------
T_theileri_Tth.4.4470/1-974   SASTVDE-------------------------------------
T_theileri_Tth.24.1440/1-461  --------------------------------------------
T_theileri_Tth.44.1540/1-561  QPAKEKEPKEEIDD------------------------------
T_theileri_Tth.91.1000/1-302  VE------------------------------------------
T_theileri_Tth.26.2510/1-186  --------------------------------------------
T_theileri_Tth.83.1090/1-415  --------------------------------------------
T_theileri_Tth.61.1050/1-209  --------------------------------------------
T_theileri_Tth.54.1330/1-803  PSTDPDTVEKVQTSQTE---------------------------
T_theileri_Tth.87.1010/1-146  --------------------------------------------
```

```
                                       1370      1380      1390      1400
T_theileri_Tth.124.1010/1-124   --------------------------------------------
T_theileri_Tth.52.1320/1-345    --------------------------------------------
T_theileri_Tth.46.1750/1-879    DESHVELHEPEI--------------------------------
T_theileri_Tth.85.1050/1-656    --------------------------------------------
T_theileri_Tth.63.1230/1-856    HSQSVSPTVPTNVNQNVGSLPVATPLARENEERQKHQGPDEHRD
T_theileri_Tth.19.2160/1-140    --------------------------------------------
T_theileri_Tth.2.1500/1-116     --------------------------------------------
T_theileri_Tth.11.2420/1-601    --------------------------------------------
T_theileri_Tth.4.4280/1-686     --------------------------------------------
T_theileri_Tth.5.3690/1-642     --------------------------------------------
T_theileri_Tth.24.2350/1-645    --------------------------------------------
T_theileri_Tth.36.2130/1-775    --------------------------------------------
T_theileri_Tth.13.2470/1-271    --------------------------------------------
T_theileri_Tth.38.1990/1-923    ETTTRSPFSPDAV---VNEPHVPHTEQEEVKESPENKHPDKPED
T_theileri_Tth.17.2230/1-737    --------------------------------------------
T_theileri_Tth.36.1950/1-407    --------------------------------------------
T_theileri_Tth.71.1100/1-161    --------------------------------------------
T_theileri_Tth.117.1060/1-252   --------------------------------------------
T_theileri_Tth.4.4250/1-116     --------------------------------------------
T_theileri_Tth.97.1070/1-422    GAGSTGLE------------------------------------
T_theileri_Tth.7.4640/1-495     --------------------------------------------
T_theileri_Tth.71.1060/1-278    --------------------------------------------
T_theileri_Tth.11.3640/1-145    --------------------------------------------
T_theileri_Tth.12.1860/1-209    --------------------------------------------
T_theileri_Tth.13.3150/1-731    --------------------------------------------
T_theileri_Tth.12.1040/1-712    --------------------------------------------
T_theileri_Tth.120.1090/1-644   --------------------------------------------
T_theileri_Tth.107.1060/1-758   --------------------------------------------
T_theileri_Tth.71.1000/1-211    --------------------------------------------
T_theileri_Tth.19.2170/1-599    RFLAALSAFFLFLLALFSNILKPCLHL-----------------
T_theileri_Tth.132.1040/1-509   --------------------------------------------
T_theileri_Tth.39.1020/1-195    --------------------------------------------
T_theileri_Tth.14.1420/1-170    --------------------------------------------
T_theileri_Tth.101.1040/1-225   --------------------------------------------
T_theileri_Tth.39.1010/1-861    --------------------------------------------
T_theileri_Tth.27.1400/1-341    --------------------------------------------
T_theileri_Tth.54.1360/1-370    --------------------------------------------
T_theileri_Tth.13.3260/1-705    --------------------------------------------
T_theileri_Tth.101.1030/1-620   --------------------------------------------
T_theileri_Tth.54.1310/1-800    --------------------------------------------
T_theileri_Tth.54.1370/1-309    --------------------------------------------
T_theileri_Tth.32.2270/1-628    --------------------------------------------
T_theileri_Tth.17.2210/1-197    --------------------------------------------
T_theileri_Tth.36.2100/1-574    --------------------------------------------
T_theileri_Tth.10.2530/1-248    --------------------------------------------
T_theileri_Tth.165.1000/1-309   --------------------------------------------
T_theileri_Tth.29.1010/1-643    --------------------------------------------
T_theileri_Tth.13.3140/1-290    --------------------------------------------
T_theileri_Tth.124.1000/1-107   --------------------------------------------
T_theileri_Tth.4.4470/1-974     --------------------------------------------
T_theileri_Tth.24.1440/1-461    --------------------------------------------
T_theileri_Tth.44.1540/1-561    --------------------------------------------
T_theileri_Tth.91.1000/1-302    --------------------------------------------
T_theileri_Tth.26.2510/1-186    --------------------------------------------
T_theileri_Tth.83.1090/1-415    --------------------------------------------
T_theileri_Tth.61.1050/1-209    --------------------------------------------
T_theileri_Tth.54.1330/1-803    --------------------------------------------
T_theileri_Tth.87.1010/1-146    --------------------------------------------
```

```
                                 1410      1420      1430      1440      1450
T_theileri_Tth.124.1010/1-124  --------------------------------------------
T_theileri_Tth.52.1320/1-345   --------------------------------------------
T_theileri_Tth.46.1750/1-879   --------------------------------------------
T_theileri_Tth.85.1050/1-656   --------------------------------------------
T_theileri_Tth.63.1230/1-856   --------------------------------------------
T_theileri_Tth.19.2160/1-140   --------------------------------------------
T_theileri_Tth.2.1500/1-116    --------------------------------------------
T_theileri_Tth.11.2420/1-601   --------------------------------------------
T_theileri_Tth.4.4280/1-686    --------------------------------------------
T_theileri_Tth.5.3690/1-642    --------------------------------------------
T_theileri_Tth.24.2350/1-645   --------------------------------------------
T_theileri_Tth.36.2130/1-775   --------------------------------------------
T_theileri_Tth.13.2470/1-271   --------------------------------------------
T_theileri_Tth.38.1990/1-923   --------------------------------------------
T_theileri_Tth.17.2230/1-737   --------------------------------------------
T_theileri_Tth.36.1950/1-407   --------------------------------------------
T_theileri_Tth.71.1100/1-161   --------------------------------------------
T_theileri_Tth.117.1060/1-252  --------------------------------------------
T_theileri_Tth.4.4250/1-116    --------------------------------------------
T_theileri_Tth.97.1070/1-422   --------------------------------------------
T_theileri_Tth.7.4640/1-495    --------------------------------------------
T_theileri_Tth.71.1060/1-278   --------------------------------------------
T_theileri_Tth.11.3640/1-145   --------------------------------------------
T_theileri_Tth.12.1860/1-209   --------------------------------------------
T_theileri_Tth.13.3150/1-731   --------------------------------------------
T_theileri_Tth.12.1040/1-712   --------------------------------------------
T_theileri_Tth.120.1090/1-644  --------------------------------------------
T_theileri_Tth.107.1060/1-758  --------------------------------------------
T_theileri_Tth.71.1000/1-211   --------------------------------------------
T_theileri_Tth.19.2170/1-599   --------------------------------------------
T_theileri_Tth.132.1040/1-509  --------------------------------------------
T_theileri_Tth.39.1020/1-195   --------------------------------------------
T_theileri_Tth.14.1420/1-170   --------------------------------------------
T_theileri_Tth.101.1040/1-225  --------------------------------------------
T_theileri_Tth.39.1010/1-861   --------------------------------------------
T_theileri_Tth.27.1400/1-341   --------------------------------------------
T_theileri_Tth.54.1360/1-370   --------------------------------------------
T_theileri_Tth.13.3260/1-705   --------------------------------------------
T_theileri_Tth.101.1030/1-620  --------------------------------------------
T_theileri_Tth.54.1310/1-800   --------------------------------------------
T_theileri_Tth.54.1370/1-309   --------------------------------------------
T_theileri_Tth.32.2270/1-628   --------------------------------------------
T_theileri_Tth.17.2210/1-197   --------------------------------------------
T_theileri_Tth.36.2100/1-574   --------------------------------------------
T_theileri_Tth.10.2530/1-248   --------------------------------------------
T_theileri_Tth.165.1000/1-309  --------------------------------------------
T_theileri_Tth.29.1010/1-643   --------------------------------------------
T_theileri_Tth.13.3140/1-290   --------------------------------------------
T_theileri_Tth.124.1000/1-107  --------------------------------------------
T_theileri_Tth.4.4470/1-974    --------------------------------------------
T_theileri_Tth.24.1440/1-461   --------------------------------------------
T_theileri_Tth.44.1540/1-561   --------------------------------------------
T_theileri_Tth.91.1000/1-302   --------------------------------------------
T_theileri_Tth.26.2510/1-186   --------------------------------------------
T_theileri_Tth.83.1090/1-415   --------------------------------------------
T_theileri_Tth.61.1050/1-209   --------------------------------------------
T_theileri_Tth.54.1330/1-803   --------------------------------------------
T_theileri_Tth.87.1010/1-146   --------------------------------------------
```

```
                                       1460      1470      1480      1490
T_theileri_Tth.124.1010/1-124  --------------------------------------------
T_theileri_Tth.52.1320/1-345   --------------------------------------------
T_theileri_Tth.46.1750/1-879   ------------------------------------VKEVPESE
T_theileri_Tth.85.1050/1-656   --------------------------------------------
T_theileri_Tth.63.1230/1-856   ------------------------------------ASQKSPES
T_theileri_Tth.19.2160/1-140   --------------------------------------------
T_theileri_Tth.2.1500/1-116    --------------------------------------------
T_theileri_Tth.11.2420/1-601   --------------------------------------------
T_theileri_Tth.4.4280/1-686    --------------------------------------------
T_theileri_Tth.5.3690/1-642    --------------------------------------------
T_theileri_Tth.24.2350/1-645   --------------------------------------------
T_theileri_Tth.36.2130/1-775   --------------------------------------------
T_theileri_Tth.13.2470/1-271   --------------------------------------------
T_theileri_Tth.38.1990/1-923   ------------------------------------TSLKRPSS
T_theileri_Tth.17.2230/1-737   --------------------------------------------
T_theileri_Tth.36.1950/1-407   --------------------------------------------
T_theileri_Tth.71.1100/1-161   --------------------------------------------
T_theileri_Tth.117.1060/1-252  --------------------------------------------
T_theileri_Tth.4.4250/1-116    --------------------------------------------
T_theileri_Tth.97.1070/1-422   ------------------------------------NKDKTPES
T_theileri_Tth.7.4640/1-495    --------------------------------------------
T_theileri_Tth.71.1060/1-278   --------------------------------------------
T_theileri_Tth.11.3640/1-145   --------------------------------------------
T_theileri_Tth.12.1860/1-209   --------------------------------------------
T_theileri_Tth.13.3150/1-731   --------------------------------------------
T_theileri_Tth.12.1040/1-712   ------------------------------------ANEQSNSS
T_theileri_Tth.120.1090/1-644  --------------------------------------------
T_theileri_Tth.107.1060/1-758  --------------------------------------------
T_theileri_Tth.71.1000/1-211   --------------------------------------------
T_theileri_Tth.19.2170/1-599   --------------------------------------------
T_theileri_Tth.132.1040/1-509  --------------------------------------------
T_theileri_Tth.39.1020/1-195   --------------------------------------------
T_theileri_Tth.14.1420/1-170   --------------------------------------------
T_theileri_Tth.101.1040/1-225  --------------------------------------------
T_theileri_Tth.39.1010/1-861   ------------------------------------NKVVSTSS
T_theileri_Tth.27.1400/1-341   --------------------------------------------
T_theileri_Tth.54.1360/1-370   --------------------------------------------
T_theileri_Tth.13.3260/1-705   --------------------------------------------
T_theileri_Tth.101.1030/1-620  --------------------------------------------
T_theileri_Tth.54.1310/1-800   --------------------------------------------
T_theileri_Tth.54.1370/1-309   --------------------------------------------
T_theileri_Tth.32.2270/1-628   --------------------------------------------
T_theileri_Tth.17.2210/1-197   --------------------------------------------
T_theileri_Tth.36.2100/1-574   --------------------------------------------
T_theileri_Tth.10.2530/1-248   --------------------------------------------
T_theileri_Tth.165.1000/1-309  --------------------------------------------
T_theileri_Tth.29.1010/1-643   --------------------------------------------
T_theileri_Tth.13.3140/1-290   --------------------------------------------
T_theileri_Tth.124.1000/1-107  --------------------------------------------
T_theileri_Tth.4.4470/1-974    ----------------------GPLREYALDVILLLNTTVSGNK
T_theileri_Tth.24.1440/1-461   --------------------------------------------
T_theileri_Tth.44.1540/1-561   --------------------------------------------
T_theileri_Tth.91.1000/1-302   --------------------------------------------
T_theileri_Tth.26.2510/1-186   --------------------------------------------
T_theileri_Tth.83.1090/1-415   --------------------------------------------
T_theileri_Tth.61.1050/1-209   --------------------------------------------
T_theileri_Tth.54.1330/1-803   --------------------------------------------
T_theileri_Tth.87.1010/1-146   --------------------------------------------
```

```
                                     1500      1510      1520      1530
T_theileri_Tth.124.1010/1-124  --------------------------------------------
T_theileri_Tth.52.1320/1-345   --------------------------------------------
T_theileri_Tth.46.1750/1-879   LPDKHEGHQHVNGPVESQKSPLEKLDSKNVQNSVVSTAGSEGGI
T_theileri_Tth.85.1050/1-656   --------------------------------------------
T_theileri_Tth.63.1230/1-856   --------------------------------MGVQNRVAPTVV
T_theileri_Tth.19.2160/1-140   --------------------------------------------
T_theileri_Tth.2.1500/1-116    --------------------------------------------
T_theileri_Tth.11.2420/1-601   --------------------------------------------
T_theileri_Tth.4.4280/1-686    --------------------------------------------
T_theileri_Tth.5.3690/1-642    --------------------------------------------
T_theileri_Tth.24.2350/1-645   --------------------------------------------
T_theileri_Tth.36.2130/1-775   --------------------------------------------
T_theileri_Tth.13.2470/1-271   --------------------------------------------
T_theileri_Tth.38.1990/1-923   DTKIEQPHQEVNKPIESQNSSPNNPRIENNHDNGLPSRSAGSSS
T_theileri_Tth.17.2230/1-737   --------------------------------------------
T_theileri_Tth.36.1950/1-407   --------------------------------------------
T_theileri_Tth.71.1100/1-161   --------------------------------------------
T_theileri_Tth.117.1060/1-252  --------------------------------------------
T_theileri_Tth.4.4250/1-116    --------------------------------------------
T_theileri_Tth.97.1070/1-422   TERGKDRNGSVTTVGGVSSSTGSGGTSSTEPIKEDESNAAVAR-
T_theileri_Tth.7.4640/1-495    --------------------------------------------
T_theileri_Tth.71.1060/1-278   --------------------------------------------
T_theileri_Tth.11.3640/1-145   --------------------------------------------
T_theileri_Tth.12.1860/1-209   --------------------------------------------
T_theileri_Tth.13.3150/1-731   --------------------------------------------
T_theileri_Tth.12.1040/1-712   AGSKPNNDAGTDTRVETS-----------------------SN-
T_theileri_Tth.120.1090/1-644  --------------------------------------------
T_theileri_Tth.107.1060/1-758  --------------------------------------------
T_theileri_Tth.71.1000/1-211   --------------------------------------------
T_theileri_Tth.19.2170/1-599   --------------------------------------------
T_theileri_Tth.132.1040/1-509  --------------------------------------------
T_theileri_Tth.39.1020/1-195   --------------------------------------------
T_theileri_Tth.14.1420/1-170   --------------------------------------------
T_theileri_Tth.101.1040/1-225  --------------------------------------------
T_theileri_Tth.39.1010/1-861   LGNEEQKSHVKPTARPVKQQHQETEQEDQKVQQRVESQIPSSEI
T_theileri_Tth.27.1400/1-341   --------------------------------------------
T_theileri_Tth.54.1360/1-370   --------------------------------------------
T_theileri_Tth.13.3260/1-705   --------------------------------------------
T_theileri_Tth.101.1030/1-620  --------------------------------------------
T_theileri_Tth.54.1310/1-800   --------------------------------------------
T_theileri_Tth.54.1370/1-309   --------------------------------------------
T_theileri_Tth.32.2270/1-628   --------------------------------------------
T_theileri_Tth.17.2210/1-197   --------------------------------------------
T_theileri_Tth.36.2100/1-574   --------------------------------------------
T_theileri_Tth.10.2530/1-248   --------------------------------------------
T_theileri_Tth.165.1000/1-309  --------------------------------------------
T_theileri_Tth.29.1010/1-643   --------------------------------------------
T_theileri_Tth.13.3140/1-290   --------------------------------------------
T_theileri_Tth.124.1000/1-107  --------------------------------------------
T_theileri_Tth.4.4470/1-974    SNVMHTLGRMATVEPGMFFILLQEEITTLCKCKLGTSNIRILPE
T_theileri_Tth.24.1440/1-461   --------------------------------------------
T_theileri_Tth.44.1540/1-561   --------------------------------------------
T_theileri_Tth.91.1000/1-302   --------------------------------------------
T_theileri_Tth.26.2510/1-186   --------------------------------------------
T_theileri_Tth.83.1090/1-415   --------------------------------------------
T_theileri_Tth.61.1050/1-209   --------------------------------------------
T_theileri_Tth.54.1330/1-803   --------------------------------------------
T_theileri_Tth.87.1010/1-146   --------------------------------------------
```

```
                                          1550      1560      1570      1580
T_theileri_Tth.124.1010/1-124  --------------------------------------------
T_theileri_Tth.52.1320/1-345   --------------------------------------------
T_theileri_Tth.46.1750/1-879   RTSGTNTNNTSVVGPGGTGSGKPLVPGPVQTPPPPPTPAAPAPS
T_theileri_Tth.85.1050/1-656   --------------------------------------------
T_theileri_Tth.63.1230/1-856   QV-SSSGTANAG------NDNGSAVGTVGTSSNGRNLINAGRST
T_theileri_Tth.19.2160/1-140   --------------------------------------------
T_theileri_Tth.2.1500/1-116    --------------------------------------------
T_theileri_Tth.11.2420/1-601   --------------------------------------------
T_theileri_Tth.4.4280/1-686    --------------------------------------------
T_theileri_Tth.5.3690/1-642    --------------------------------------------
T_theileri_Tth.24.2350/1-645   --------------------------------------------
T_theileri_Tth.36.2130/1-775   --------------------------------------------
T_theileri_Tth.13.2470/1-271   --------------------------------------------
T_theileri_Tth.38.1990/1-923   GIGSASGIHNGGVSRGTNDDNSSALGS-GASGNERNPVNGDQSV
T_theileri_Tth.17.2230/1-737   --------------------------------------------
T_theileri_Tth.36.1950/1-407   --------------------------------------------
T_theileri_Tth.71.1100/1-161   --------------------------------------------
T_theileri_Tth.117.1060/1-252  --------------------------------------------
T_theileri_Tth.4.4250/1-116    --------------------------------------------
T_theileri_Tth.97.1070/1-422   -----------------------------PGHNSSSSLPAADA-
T_theileri_Tth.7.4640/1-495    --------------------------------------------
T_theileri_Tth.71.1060/1-278   --------------------------------------------
T_theileri_Tth.11.3640/1-145   --------------------------------------------
T_theileri_Tth.12.1860/1-209   --------------------------------------------
T_theileri_Tth.13.3150/1-731   --------------------------------------------
T_theileri_Tth.12.1040/1-712   -----------------------------NQVESVPAVLGVNGS
T_theileri_Tth.120.1090/1-644  --------------------------------------------
T_theileri_Tth.107.1060/1-758  --------------------------------------------
T_theileri_Tth.71.1000/1-211   --------------------------------------------
T_theileri_Tth.19.2170/1-599   --------------------------------------------
T_theileri_Tth.132.1040/1-509  --------------------------------------------
T_theileri_Tth.39.1020/1-195   --------------------------------------------
T_theileri_Tth.14.1420/1-170   --------------------------------------------
T_theileri_Tth.101.1040/1-225  --------------------------------------------
T_theileri_Tth.39.1010/1-861   LRSENDNGSNQQKVTGPLRGSDSIVGGLAVQSVQNSPSPSVSPA
T_theileri_Tth.27.1400/1-341   --------------------------------------------
T_theileri_Tth.54.1360/1-370   --------------------------------------------
T_theileri_Tth.13.3260/1-705   --------------------------------------------
T_theileri_Tth.101.1030/1-620  --------------------------------------------
T_theileri_Tth.54.1310/1-800   --------------------------------------------
T_theileri_Tth.54.1370/1-309   --------------------------------------------
T_theileri_Tth.32.2270/1-628   --------------------------------------------
T_theileri_Tth.17.2210/1-197   --------------------------------------------
T_theileri_Tth.36.2100/1-574   --------------------------------------------
T_theileri_Tth.10.2530/1-248   --------------------------------------------
T_theileri_Tth.165.1000/1-309  --------------------------------------------
T_theileri_Tth.29.1010/1-643   --------------------------------------------
T_theileri_Tth.13.3140/1-290   --------------------------------------------
T_theileri_Tth.124.1000/1-107  --------------------------------------------
T_theileri_Tth.4.4470/1-974    YASAAVVNGGIVVNFQLGLMIGAGSATIENMRNMYESKFMVNSV
T_theileri_Tth.24.1440/1-461   --------------------------------------------
T_theileri_Tth.44.1540/1-561   --------------------------------------------
T_theileri_Tth.91.1000/1-302   --------------------------------------------
T_theileri_Tth.26.2510/1-186   --------------------------------------------
T_theileri_Tth.83.1090/1-415   --------------------------------------------
T_theileri_Tth.61.1050/1-209   --------------------------------------------
T_theileri_Tth.54.1330/1-803   --------------------------------------------
T_theileri_Tth.87.1010/1-146   --------------------------------------------
```

```
                                   1590      1600      1610      1620
T_theileri_Tth.124.1010/1-124  --------------------------------------------
T_theileri_Tth.52.1320/1-345   --------------------------------------------
T_theileri_Tth.46.1750/1-879   PSPATA------------------SSEAPAHGISQQIADPPAEN
T_theileri_Tth.85.1050/1-656   --------------------------------------------
T_theileri_Tth.63.1230/1-856   QSVPSS--------------APAPANTDLHETLKEKIADPPAAN
T_theileri_Tth.19.2160/1-140   --------------------------------------------
T_theileri_Tth.2.1500/1-116    --------------------------------------------
T_theileri_Tth.11.2420/1-601   --------------------------------------------
T_theileri_Tth.4.4280/1-686    --------------------------------------------
T_theileri_Tth.5.3690/1-642    --------------------------------------------
T_theileri_Tth.24.2350/1-645   --------------------------------------------
T_theileri_Tth.36.2130/1-775   --------------------------------------------
T_theileri_Tth.13.2470/1-271   --------------------------------------------
T_theileri_Tth.38.1990/1-923   QSETSSSAPVVPVVPAKTPAAPAPADTDTNE-IPQQPADSRAEN
T_theileri_Tth.17.2230/1-737   --------------------------------------------
T_theileri_Tth.36.1950/1-407   --------------------------------------------
T_theileri_Tth.71.1100/1-161   --------------------------------------------
T_theileri_Tth.117.1060/1-252  --------------------------------------------
T_theileri_Tth.4.4250/1-116    --------------------------------------------
T_theileri_Tth.97.1070/1-422   --------------------------------------------
T_theileri_Tth.7.4640/1-495    --------------------------------------------
T_theileri_Tth.71.1060/1-278   --------------------------------------------
T_theileri_Tth.11.3640/1-145   --------------------------------------------
T_theileri_Tth.12.1860/1-209   --------------------------------------------
T_theileri_Tth.13.3150/1-731   --------------------------------------------
T_theileri_Tth.12.1040/1-712   SQESST----------------ATQSVIQHTEKNSTNTETAVEK
T_theileri_Tth.120.1090/1-644  --------------------------------------------
T_theileri_Tth.107.1060/1-758  --------------------------------------------
T_theileri_Tth.71.1000/1-211   --------------------------------------------
T_theileri_Tth.19.2170/1-599   --------------------------------------------
T_theileri_Tth.132.1040/1-509  --------------------------------------------
T_theileri_Tth.39.1020/1-195   --------------------------------------------
T_theileri_Tth.14.1420/1-170   --------------------------------------------
T_theileri_Tth.101.1040/1-225  --------------------------------------------
T_theileri_Tth.39.1010/1-861   AAPAHA----------------TASSKAPAHGISQQIADPPAEN
T_theileri_Tth.27.1400/1-341   --------------------------------------------
T_theileri_Tth.54.1360/1-370   --------------------------------------------
T_theileri_Tth.13.3260/1-705   ---------------------------NASEIPSPNVDSPEKND
T_theileri_Tth.101.1030/1-620  --------------------------------------------
T_theileri_Tth.54.1310/1-800   --------------------------------------------
T_theileri_Tth.54.1370/1-309   --------------------------------------------
T_theileri_Tth.32.2270/1-628   ----------------------------ASEIPSPNVDSPEKND
T_theileri_Tth.17.2210/1-197   --------------------------------------------
T_theileri_Tth.36.2100/1-574   --------------------------------------------
T_theileri_Tth.10.2530/1-248   --------------------------------------------
T_theileri_Tth.165.1000/1-309  --------------------------------------------
T_theileri_Tth.29.1010/1-643   ---------------------------NASEIPSPNVDSAEKND
T_theileri_Tth.13.3140/1-290   --------------------------------------------
T_theileri_Tth.124.1000/1-107  --------------------------------------------
T_theileri_Tth.4.4470/1-974    LSRIGAYITEEEKEEEGGIFTVTIASSEILPEAVIVDEITSLDQ
T_theileri_Tth.24.1440/1-461   --------------------------------------------
T_theileri_Tth.44.1540/1-561   --------------------------------------------
T_theileri_Tth.91.1000/1-302   --------------------------------------------
T_theileri_Tth.26.2510/1-186   --------------------------------------------
T_theileri_Tth.83.1090/1-415   --------------------------------------------
T_theileri_Tth.61.1050/1-209   --------------------------------------------
T_theileri_Tth.54.1330/1-803   --------------------------------------------
T_theileri_Tth.87.1010/1-146   --------------------------------------------
```

```
                                    1630      1640      1650      1660      1670
T_theileri_Tth.124.1010/1-124   --------------------------------------------
T_theileri_Tth.52.1320/1-345    --------------------------------------------
T_theileri_Tth.46.1750/1-879    NDQQNGTLTPTQAQDRQQGPNQRQNEVQSPTETNAPTQESTPQY
T_theileri_Tth.85.1050/1-656    --------------------------------------------
T_theileri_Tth.63.1230/1-856    NDQQNATQTSTKAEP-SSGP-------------RTPAEANAQTD
T_theileri_Tth.19.2160/1-140    --------------------------------------------
T_theileri_Tth.2.1500/1-116     --------------------------------------------
T_theileri_Tth.11.2420/1-601    --------------------------------------------
T_theileri_Tth.4.4280/1-686     --------------------------------------------
T_theileri_Tth.5.3690/1-642     --------------------------------------------
T_theileri_Tth.24.2350/1-645    --------------------------------------------
T_theileri_Tth.36.2130/1-775    --------------------------------------------
T_theileri_Tth.13.2470/1-271    --------------------------------------------
T_theileri_Tth.38.1990/1-923    KDQQNGIQTPTQAQDRQQGPNQTQNEVQFPNEVKTPSQ---QST
T_theileri_Tth.17.2230/1-737    --------------------------------------------
T_theileri_Tth.36.1950/1-407    --------------------------------------------
T_theileri_Tth.71.1100/1-161    --------------------------------------------
T_theileri_Tth.117.1060/1-252   --------------------------------------------
T_theileri_Tth.4.4250/1-116     --------------------------------------------
T_theileri_Tth.97.1070/1-422    --------------------------------------------
T_theileri_Tth.7.4640/1-495     --------------------------------------------
T_theileri_Tth.71.1060/1-278    --------------------------------------------
T_theileri_Tth.11.3640/1-145    --------------------------------------------
T_theileri_Tth.12.1860/1-209    --------------------------------------------
T_theileri_Tth.13.3150/1-731    --------------------------------------------
T_theileri_Tth.12.1040/1-712    GRRASRS-------------------------------------
T_theileri_Tth.120.1090/1-644   --------------------------------------------
T_theileri_Tth.107.1060/1-758   --------------------------------------------
T_theileri_Tth.71.1000/1-211    --------------------------------------------
T_theileri_Tth.19.2170/1-599    --------------------------------------------
T_theileri_Tth.132.1040/1-509   --------------------------------------------
T_theileri_Tth.39.1020/1-195    --------------------------------------------
T_theileri_Tth.14.1420/1-170    --------------------------------------------
T_theileri_Tth.101.1040/1-225   --------------------------------------------
T_theileri_Tth.39.1010/1-861    NDKQNEDHTTSKVQD--------------------------HQQ
T_theileri_Tth.27.1400/1-341    --------------------------------------------
T_theileri_Tth.54.1360/1-370    --------------------------------------------
T_theileri_Tth.13.3260/1-705    PSEAEKAPQTEESHHPNKSPQEEDTQNAEIPSKEGGSPEKNDPS
T_theileri_Tth.101.1030/1-620   --------------------------------------------
T_theileri_Tth.54.1310/1-800    --------------------------------------------
T_theileri_Tth.54.1370/1-309    --------------------------------------------
T_theileri_Tth.32.2270/1-628    SSE-----------------------------------------
T_theileri_Tth.17.2210/1-197    --------------------------------------------
T_theileri_Tth.36.2100/1-574    --------------------------------------------
T_theileri_Tth.10.2530/1-248    --------------------------------------------
T_theileri_Tth.165.1000/1-309   --------------------------------------------
T_theileri_Tth.29.1010/1-643    PSE-----------------------------------------
T_theileri_Tth.13.3140/1-290    --------------------------------------------
T_theileri_Tth.124.1000/1-107   --------------------------------------------
T_theileri_Tth.4.4470/1-974     LVALGEIHLHCKGDLTGMPSLTDQKVITLLENAVRQDISQIILL
T_theileri_Tth.24.1440/1-461    --------------------------------------------
T_theileri_Tth.44.1540/1-561    --------------------------------------------
T_theileri_Tth.91.1000/1-302    --------------------------------------------
T_theileri_Tth.26.2510/1-186    --------------------------------------------
T_theileri_Tth.83.1090/1-415    --------------------------------------------
T_theileri_Tth.61.1050/1-209    --------------------------------------------
T_theileri_Tth.54.1330/1-803    --------------------------------------------
T_theileri_Tth.87.1010/1-146    --------------------------------------------
```

```
                                 1680      1690      1700      1710
T_theileri_Tth.124.1010/1-124  --------------------------------------------
T_theileri_Tth.52.1320/1-345   --------------------------------------------
T_theileri_Tth.46.1750/1-879   KNSSTSPTTDERDAQEEDHTS-----------------------
T_theileri_Tth.85.1050/1-656   ------------------TQKPNETGGAESGSTNSSSDSRPSQD
T_theileri_Tth.63.1230/1-856   KHGKISSPASTEVQSDGKREENPSRWRRDVDDATS---------
T_theileri_Tth.19.2160/1-140   --------------------------------------------
T_theileri_Tth.2.1500/1-116    --------------------------------------------
T_theileri_Tth.11.2420/1-601   --------------------------------------------
T_theileri_Tth.4.4280/1-686    -------------------------MEDRNEEGEVNPSVNDESA
T_theileri_Tth.5.3690/1-642    ------------------HQEQGETPHTESGHTNNNSSSVPTQS
T_theileri_Tth.24.2350/1-645   --------------------------------------------
T_theileri_Tth.36.2130/1-775   ------------------------------------------ST
T_theileri_Tth.13.2470/1-271   --------------------------------------------
T_theileri_Tth.38.1990/1-923   PQYKNSSTSPTTDEKDAQEEDHTSRNRRNTDAATS---------
T_theileri_Tth.17.2230/1-737   -----------------VPTPQPTQWPTPIGE------------
T_theileri_Tth.36.1950/1-407   --------------------------------------------
T_theileri_Tth.71.1100/1-161   --------------------------------------------
T_theileri_Tth.117.1060/1-252  --------------------------------------------
T_theileri_Tth.4.4250/1-116    --------------------------------------------
T_theileri_Tth.97.1070/1-422   --------------------------------------------
T_theileri_Tth.7.4640/1-495    --------------------------------------------
T_theileri_Tth.71.1060/1-278   --------------------------------------------
T_theileri_Tth.11.3640/1-145   --------------------------------------------
T_theileri_Tth.12.1860/1-209   --------------------------------------------
T_theileri_Tth.13.3150/1-731   -----------------SSDPLPHPAPEPTVETVGAPHVEKEPS
T_theileri_Tth.12.1040/1-712   ---------------RRHIDDAT----------TS---------
T_theileri_Tth.120.1090/1-644  ------------------HQEQGETVGTESGHTNNNSSSVPSQS
T_theileri_Tth.107.1060/1-758  -----------------------------------LSDGTQPVT
T_theileri_Tth.71.1000/1-211   --------------------------------------------
T_theileri_Tth.19.2170/1-599   --------------------------------------------
T_theileri_Tth.132.1040/1-509  --------------------------------------------
T_theileri_Tth.39.1020/1-195   --------------------------------------------
T_theileri_Tth.14.1420/1-170   -----------------SSDPLPHPTPE----------------
T_theileri_Tth.101.1040/1-225  --------------------------------------------
T_theileri_Tth.39.1010/1-861   ASNQTQNEVQSPTEANAQTDDSTDVQNNTHGEESA---------
T_theileri_Tth.27.1400/1-341   --------------------------------------------
T_theileri_Tth.54.1360/1-370   --------------------------------------------
T_theileri_Tth.13.3260/1-705   E-----------TEKAPQTEGSHPEE-------------KPSQD
T_theileri_Tth.101.1030/1-620  ------------------------------------TSLQGDQG
T_theileri_Tth.54.1310/1-800   -------------------------------------------A
T_theileri_Tth.54.1370/1-309   ------------------------------------------VT
T_theileri_Tth.32.2270/1-628   ------------AEKAPQTEESHHPN-------------KSPQE
T_theileri_Tth.17.2210/1-197   --------------------------------------------
T_theileri_Tth.36.2100/1-574   ------------------------------------------AA
T_theileri_Tth.10.2530/1-248   --------------------------------------------
T_theileri_Tth.165.1000/1-309  --------------------------------------------
T_theileri_Tth.29.1010/1-643   ------------TEKAPQTEGSNPQE-------------KPSQD
T_theileri_Tth.13.3140/1-290   -----------------DTSPQPIPPTVPNAE------------
T_theileri_Tth.124.1000/1-107  --------------------------------------------
T_theileri_Tth.4.4470/1-974    TPNLIEVKHLQITGSTDLLITLTVYFPPVADGIGGNENILHLWE
T_theileri_Tth.24.1440/1-461   --------------------------------------------
T_theileri_Tth.44.1540/1-561   --------------------------------------------
T_theileri_Tth.91.1000/1-302   --------------------------------------------
T_theileri_Tth.26.2510/1-186   --------------------------------------------
T_theileri_Tth.83.1090/1-415   --------------------------------------------
T_theileri_Tth.61.1050/1-209   --------------------------------------------
T_theileri_Tth.54.1330/1-803   ------------------------------------------GE
T_theileri_Tth.87.1010/1-146   --------------------------------------------
```

```
                                    1720      1730      1740      1750
T_theileri_Tth.124.1010/1-124  --------------------------------------------
T_theileri_Tth.52.1320/1-345   --------------------------------------------
T_theileri_Tth.46.1750/1-879   --RNRRNTDATTSPNTSNPNTVDAANEPSSHTANNGALNGTKLT
T_theileri_Tth.85.1050/1-656   DQKAHDTTGAEKASSEDAGASSSSSSSSSSTSSLPSRSRRNSET
T_theileri_Tth.63.1230/1-856   --SSTSNPNSNDN-PLQSTNTVNGADDRSSGTVNSDALNGTKFT
T_theileri_Tth.19.2160/1-140   --------------------------------------------
T_theileri_Tth.2.1500/1-116    --------------------------------------------
T_theileri_Tth.11.2420/1-601   --------------------------------------------
T_theileri_Tth.4.4280/1-686    SPHTSNGAGSQGSITHNDPTTGTTVRGETNIPDVHIGDHDTIID
T_theileri_Tth.5.3690/1-642    AQRTQNTTGVNVST----------------------------ND
T_theileri_Tth.24.2350/1-645   --------------------------------------------
T_theileri_Tth.36.2130/1-775   EVTETAKP---------SESAAASNN-AGNQGVSVPVVQNETST
T_theileri_Tth.13.2470/1-271   --------------------------------------------
T_theileri_Tth.38.1990/1-923   --PNTSYPNTGD-----------AANDQSSHTVNSDSLKVPNLT
T_theileri_Tth.17.2230/1-737   -DRGDASPEETVRPHTSEEVGQHDSTHHNIPTTSVTVDEHMELA
T_theileri_Tth.36.1950/1-407   ---------------------------HGNDGRS----------
T_theileri_Tth.71.1100/1-161   --------------------------------------------
T_theileri_Tth.117.1060/1-252  --------------------------------------------
T_theileri_Tth.4.4250/1-116    --------------------------------------------
T_theileri_Tth.97.1070/1-422   --SSTSNPTSDGN-TIQSTNTV---DVRPSHKINNTVLNGTKFT
T_theileri_Tth.7.4640/1-495    --------------------------------------------
T_theileri_Tth.71.1060/1-278   --------------------------------------------
T_theileri_Tth.11.3640/1-145   --------------------------------------------
T_theileri_Tth.12.1860/1-209   --------------------------------------------
T_theileri_Tth.13.3150/1-731   SSYPNVPADSSGPTDTTTPVQRDVFEKERIDSPTSTAHSETMLD
T_theileri_Tth.12.1040/1-712   --QSTSNPNGTED-AIQSQNAV--------------LKGTTLT
T_theileri_Tth.120.1090/1-644  VQRTQNTTGDKVSA----------------------------ND
T_theileri_Tth.107.1060/1-758  ETTGTAIP---------SE------SAAGSEEVTAPVVQNGAST
T_theileri_Tth.71.1000/1-211   --------------------------------------------
T_theileri_Tth.19.2170/1-599   --------------------------------------------
T_theileri_Tth.132.1040/1-509  --------------------------------------------
T_theileri_Tth.39.1020/1-195   --------------------------------------------
T_theileri_Tth.14.1420/1-170   ----------ESEKDTTTPVQTDGLS------------------
T_theileri_Tth.101.1040/1-225  --------------------------------------------
T_theileri_Tth.39.1010/1-861   --SSTSNPNSDDN-TVQSTNTVNGADDRSSGTVNSDALNGTKLT
T_theileri_Tth.27.1400/1-341   --------------------------------------------
T_theileri_Tth.54.1360/1-370   ---------------------------HRNDGRS----------
T_theileri_Tth.13.3260/1-705   EVTQNAEIPSKEGGSSKP-------------ESPDNNDTTNIEN
T_theileri_Tth.101.1030/1-620  NNGTATVLRGRRDAGAAVNPSTTSDNTQDAAAAGTTSPSGKENA
T_theileri_Tth.54.1310/1-800   ETTETTKP---------SERAGAGNSGGTNENVTGPVVENEAST
T_theileri_Tth.54.1370/1-309   ETTGTPKP---------TE------N-AGNQGVTSPVVQNEAIT
T_theileri_Tth.32.2270/1-628   EDTQNAEIPSKEGGSSKP-------------ESPDNNDTTNIEN
T_theileri_Tth.17.2210/1-197   --------------------------------------------
T_theileri_Tth.36.2100/1-574   EATGTSKP---------SE------N-AGNQEVTSPVVQNEAST
T_theileri_Tth.10.2530/1-248   --------------------------------------------
T_theileri_Tth.165.1000/1-309  --------------------------------------------
T_theileri_Tth.29.1010/1-643   EVTQNAEIPSKEGGSSKP-------------ESPDNNDTTNIEN
T_theileri_Tth.13.3140/1-290   -SEKNSSEKRDGP-HNSEEDGLNGSTTH----------------
T_theileri_Tth.124.1000/1-107  --------------------------------------------
T_theileri_Tth.4.4470/1-974    QLLMNSTTITITT-TITSMPSPLPVLTSTVQFLSKGAPVSVTDN
T_theileri_Tth.24.1440/1-461   --------------------------------------------
T_theileri_Tth.44.1540/1-561   --------------------------------------------
T_theileri_Tth.91.1000/1-302   --------------------------------------------
T_theileri_Tth.26.2510/1-186   --------------------------------------------
T_theileri_Tth.83.1090/1-415   --------------------------------------------
T_theileri_Tth.61.1050/1-209   --------------------------------------------
T_theileri_Tth.54.1330/1-803   TTTGTTKP---------SE------N-DGNFGVSLPVVQNEAST
T_theileri_Tth.87.1010/1-146   --------------------------------------------
```

```
                                    1770      1780      1790      1800
T_theileri_Tth.124.1010/1-124  --------------------------------------------
T_theileri_Tth.52.1320/1-345   --------------------------------------------
T_theileri_Tth.46.1750/1-879   EDKMRE-TLNHTNVMGALG-----------PDSSIMFTSYMAPL
T_theileri_Tth.85.1050/1-656   DVAVPPTTEGKINEDNVTSSQVPGELSIHVGMDGAPAAACVLHA
T_theileri_Tth.63.1230/1-856   EGQIKEETLNHTNIMSALG-----------PDSSIMV-SYMAPL
T_theileri_Tth.19.2160/1-140   --------------------------------------------
T_theileri_Tth.2.1500/1-116    --------------------------------------------
T_theileri_Tth.11.2420/1-601   -----------TTTDGKIKLPGFKTANNNMFESGIILLSCFFPL
T_theileri_Tth.4.4280/1-686    PTEAPSKNVQ----------------------------------
T_theileri_Tth.5.3690/1-642    NVTVPPTTEGNINKENLTSPQVKGELKIRMGMDGTPAAACVLHA
T_theileri_Tth.24.2350/1-645   ------------------GGGGGHGDGCATAAPIALSAIALMVI
T_theileri_Tth.36.2130/1-775   TKESKPSETKEENITSFLNGQNNNAAAKKGTDGSFKA-SAFASV
T_theileri_Tth.13.2470/1-271   --------------------------------------------
T_theileri_Tth.38.1990/1-923   EDQIKEETLNHTNVMSALG-----------PDSSIMF-SHMAPL
T_theileri_Tth.17.2230/1-737   DSHSHPSAALDTRETPSENVQGNHVDAESPPHPVRARRE-----
T_theileri_Tth.36.1950/1-407   -------------------------------DGSVTA-AILVPL
T_theileri_Tth.71.1100/1-161   --------------------------------------------
T_theileri_Tth.117.1060/1-252  --------------------------------------------
T_theileri_Tth.4.4250/1-116    --------------------------------------------
T_theileri_Tth.97.1070/1-422   EDQMKE-TLNHTNVMGALG-----------PDSSIMV-SYTAPF
T_theileri_Tth.7.4640/1-495    --------------------------------------------
T_theileri_Tth.71.1060/1-278   --------------------------------------------
T_theileri_Tth.11.3640/1-145   --------------------------------------------
T_theileri_Tth.12.1860/1-209   --------------------------------------------
T_theileri_Tth.13.3150/1-731   QNENHHERLDGSQHGSGSDVLTPQTNSDGNTVE-----------
T_theileri_Tth.12.1040/1-712   ERQIKEETLKHTNVTVMFG-----------ADSSIMV-SYMAPL
T_theileri_Tth.120.1090/1-644  NVTVPVTTGGNINKNNLTSPQVKEELKIHMGMDGTPAAACVLHA
T_theileri_Tth.107.1060/1-758  TTESKPSETKEENITSFLSGQNNNIVAKKGTDGSFKA-SVFASV
T_theileri_Tth.71.1000/1-211   --------------------------------------------
T_theileri_Tth.19.2170/1-599   --------------------------------------------
T_theileri_Tth.132.1040/1-509  --------------------------------------------
T_theileri_Tth.39.1020/1-195   --------------------------------------------
T_theileri_Tth.14.1420/1-170   --------------------------------------------
T_theileri_Tth.101.1040/1-225  --------------------------------------------
T_theileri_Tth.39.1010/1-861   EDKMRE-TLNHTNVMGVMG-----------TDSSIMFTSYMAPL
T_theileri_Tth.27.1400/1-341   --------------------------------------SVLPPY
T_theileri_Tth.54.1360/1-370   -------------------------------DGSVTA-AILVPL
T_theileri_Tth.13.3260/1-705   GQDDHNTGNDLTDSKVENTNSPTGVISGKDTDNSIAL-SFFGPL
T_theileri_Tth.101.1030/1-620  NIIDPSKYPGVVNQAQINNMQNESKLLIELAKDDAHSAVCSLPI
T_theileri_Tth.54.1310/1-800   TMESNPSETKEEGNTSSVSGQNNKTTAKKGTDGTFKA-SSFASV
T_theileri_Tth.54.1370/1-309   TTESKPSETKEGRITSSVSGQNNNSTAKKGADGSFKA-SLFASV
T_theileri_Tth.32.2270/1-628   GQDDHNTGNDLTDSKVENTN------------------------
T_theileri_Tth.17.2210/1-197   --------------------------------------------
T_theileri_Tth.36.2100/1-574   TTEAKPSETKEENITSSVSGQNNNIVAGKGTDGSFKA-NVFASV
T_theileri_Tth.10.2530/1-248   --------------------------------------------
T_theileri_Tth.165.1000/1-309  --------------------------------------------
T_theileri_Tth.29.1010/1-643   GQDDHNTGKDSTDSKVENTNSPTGVISGKDTDNSII--------
T_theileri_Tth.13.3140/1-290   --------------------------------------------
T_theileri_Tth.124.1000/1-107  --------------------------------------------
T_theileri_Tth.4.4470/1-974    ISSSSALSLFSADVSEVQPDSTGDSRCVVQFTGCFAVQIVILII
T_theileri_Tth.24.1440/1-461   --------------------------------------------
T_theileri_Tth.44.1540/1-561   --------------------------------------------
T_theileri_Tth.91.1000/1-302   --------------------------------------------
T_theileri_Tth.26.2510/1-186   --------------------------------------------
T_theileri_Tth.83.1090/1-415   --------------------------------------------
T_theileri_Tth.61.1050/1-209   --------------------------------------------
T_theileri_Tth.54.1330/1-803   TTESNPSETKEENITSFLNGQNNNIVAKKGIDGSLKA-NVFASV
T_theileri_Tth.87.1010/1-146   --------------------------------------------
```

```
                                          1810      1820      1830      1840
T_theileri_Tth.124.1010/1-124   --------------------------------------------
T_theileri_Tth.52.1320/1-345    --------------------------------------------
T_theileri_Tth.46.1750/1-879    ALLVCVVGFVMVP-------------------------------
T_theileri_Tth.85.1050/1-656    LFLLMAAAAAALAVPL----------------------------
T_theileri_Tth.63.1230/1-856    ALLVCVVGFVMVP-------------------------------
T_theileri_Tth.19.2160/1-140    --------------------------------------------
T_theileri_Tth.2.1500/1-116     --------------------------------------------
T_theileri_Tth.11.2420/1-601    SFIVFFIVA-----------------------------------
T_theileri_Tth.4.4280/1-686     -----------------------------GGTTHLPNNTAGITL
T_theileri_Tth.5.3690/1-642     LFLLMAAAALAVPL------------------------------
T_theileri_Tth.24.2350/1-645    TLMLLP--------------------------------------
T_theileri_Tth.36.2130/1-775    MFALLTLSVIMAP-------------------------------
T_theileri_Tth.13.2470/1-271    --------------------------------------------
T_theileri_Tth.38.1990/1-923    ALLMCVVGFVMVP-------------------------------
T_theileri_Tth.17.2230/1-737    ---------IKDGDQEDSDTKNNWAANGKGRAAHVLNNNDAITM
T_theileri_Tth.36.1950/1-407    LFIFLAVFTIMAP-------------------------------
T_theileri_Tth.71.1100/1-161    --------------------------------------------
T_theileri_Tth.117.1060/1-252   --------------------------------------------
T_theileri_Tth.4.4250/1-116     --------------------------------------------
T_theileri_Tth.97.1070/1-422    ALLVCVVGFVMVP-------------------------------
T_theileri_Tth.7.4640/1-495     --------------------------------------------
T_theileri_Tth.71.1060/1-278    --------------------------------------------
T_theileri_Tth.11.3640/1-145    --------------------------------------------
T_theileri_Tth.12.1860/1-209    --------------------------------------------
T_theileri_Tth.13.3150/1-731    ----------------RNSDARDNGVANGGGVRSVASHGDLLTL
T_theileri_Tth.12.1040/1-712    ALLVGVVGFVMVP-------------------------------
T_theileri_Tth.120.1090/1-644   LFLLMAAAAAAAALAVPL--------------------------
T_theileri_Tth.107.1060/1-758   MFVFLTLSVIMAP-------------------------------
T_theileri_Tth.71.1000/1-211    --------------------------------------------
T_theileri_Tth.19.2170/1-599    --------------------------------------------
T_theileri_Tth.132.1040/1-509   --------------------------------------------
T_theileri_Tth.39.1020/1-195    --------------------------------------------
T_theileri_Tth.14.1420/1-170    --------------------------------------------
T_theileri_Tth.101.1040/1-225   --------------------------------------------
T_theileri_Tth.39.1010/1-861    ALLVCVVGFVMVP-------------------------------
T_theileri_Tth.27.1400/1-341    VLLAVVISVIALC-------------------------------
T_theileri_Tth.54.1360/1-370    LFIFLAVSTIMAP-------------------------------
T_theileri_Tth.13.3260/1-705    MLLVCVVAAVVAL-------------------------------
T_theileri_Tth.101.1030/1-620   MIVTMVLAVVLSC-------------------------------
T_theileri_Tth.54.1310/1-800    MFVFLILSVIMVP-------------------------------
T_theileri_Tth.54.1370/1-309    MFVFLTLSMIMLP-------------------------------
T_theileri_Tth.32.2270/1-628    --------------------------------------------
T_theileri_Tth.17.2210/1-197    --------------------------------------------
T_theileri_Tth.36.2100/1-574    MFLFLTLSVIMSP-------------------------------
T_theileri_Tth.10.2530/1-248    --------------------------------------------
T_theileri_Tth.165.1000/1-309   --------------------------------------------
T_theileri_Tth.29.1010/1-643    --------------------------------------------
T_theileri_Tth.13.3140/1-290    ---------------IDSDTKNNLATNGKGPAAHVPNNSDTITV
T_theileri_Tth.124.1000/1-107   --------------------------------------------
T_theileri_Tth.4.4470/1-974     LILIVLIMIVVFLKLCLCPKPKWRIQVETCELVVPLQEKYR---
T_theileri_Tth.24.1440/1-461    --------------------------------------------
T_theileri_Tth.44.1540/1-561    --------------------------------------------
T_theileri_Tth.91.1000/1-302    --------------------------------------------
T_theileri_Tth.26.2510/1-186    --------------------------------------------
T_theileri_Tth.83.1090/1-415    --------------------------------------------
T_theileri_Tth.61.1050/1-209    --------------------------------------------
T_theileri_Tth.54.1330/1-803    MFVFLTLSAIMLP-------------------------------
T_theileri_Tth.87.1010/1-146    --------------------------------------------
```

```
                             1850      1860      1870
T_theileri_Tth.124.1010/1-124  --------------------------------
T_theileri_Tth.52.1320/1-345   --------------------------------
T_theileri_Tth.46.1750/1-879   --------------------------------
T_theileri_Tth.85.1050/1-656   --------------------------------
T_theileri_Tth.63.1230/1-856   --------------------------------
T_theileri_Tth.19.2160/1-140   --------------------------------
T_theileri_Tth.2.1500/1-116    --------------------------------
T_theileri_Tth.11.2420/1-601   --------------------------------
T_theileri_Tth.4.4280/1-686    NIGVGDGSVTAAGCAPLLFLVVAVAVAVSL--
T_theileri_Tth.5.3690/1-642    --------------------------------
T_theileri_Tth.24.2350/1-645   --------------------------------
T_theileri_Tth.36.2130/1-775   --------------------------------
T_theileri_Tth.13.2470/1-271   --------------------------------
T_theileri_Tth.38.1990/1-923   --------------------------------
T_theileri_Tth.17.2230/1-737   NIGFGDGSITTAGHASHLLLVLASAVAVVFAL
T_theileri_Tth.36.1950/1-407   --------------------------------
T_theileri_Tth.71.1100/1-161   --------------------------------
T_theileri_Tth.117.1060/1-252  --------------------------------
T_theileri_Tth.4.4250/1-116    --------------------------------
T_theileri_Tth.97.1070/1-422   --------------------------------
T_theileri_Tth.7.4640/1-495    --------------------------------
T_theileri_Tth.71.1060/1-278   --------------------------------
T_theileri_Tth.11.3640/1-145   --------------------------------
T_theileri_Tth.12.1860/1-209   --------------------------------
T_theileri_Tth.13.3150/1-731   NIGVGDGSVTPAGHAPLLLLALASVVAVVVPL
T_theileri_Tth.12.1040/1-712   --------------------------------
T_theileri_Tth.120.1090/1-644  --------------------------------
T_theileri_Tth.107.1060/1-758  --------------------------------
T_theileri_Tth.71.1000/1-211   --------------------------------
T_theileri_Tth.19.2170/1-599   --------------------------------
T_theileri_Tth.132.1040/1-509  --------------------------------
T_theileri_Tth.39.1020/1-195   --------------------------------
T_theileri_Tth.14.1420/1-170   ------GSVNPAGHAPLLLLALASVVAVMVPL
T_theileri_Tth.101.1040/1-225  --------------------------------
T_theileri_Tth.39.1010/1-861   --------------------------------
T_theileri_Tth.27.1400/1-341   --------------------------------
T_theileri_Tth.54.1360/1-370   --------------------------------
T_theileri_Tth.13.3260/1-705   --------------------------------
T_theileri_Tth.101.1030/1-620  --------------------------------
T_theileri_Tth.54.1310/1-800   --------------------------------
T_theileri_Tth.54.1370/1-309   --------------------------------
T_theileri_Tth.32.2270/1-628   --------------------------------
T_theileri_Tth.17.2210/1-197   --------------------------------
T_theileri_Tth.36.2100/1-574   --------------------------------
T_theileri_Tth.10.2530/1-248   --------------------------------
T_theileri_Tth.165.1000/1-309  --------------------------------
T_theileri_Tth.29.1010/1-643   --------------------------------
T_theileri_Tth.13.3140/1-290   NIGIGDGTVTPAGHAPLLLLIFASAVAVVFSL
T_theileri_Tth.124.1000/1-107  --------------------------------
T_theileri_Tth.4.4470/1-974    --------------------------------
T_theileri_Tth.24.1440/1-461   --------------------------------
T_theileri_Tth.44.1540/1-561   --------------------------------
T_theileri_Tth.91.1000/1-302   --------------------------------
T_theileri_Tth.26.2510/1-186   --------------------------------
T_theileri_Tth.83.1090/1-415   --------------------------------
T_theileri_Tth.61.1050/1-209   --------------------------------
T_theileri_Tth.54.1330/1-803   --------------------------------
T_theileri_Tth.87.1010/1-146   --------------------------------
```

```
                                         10        20        30        40
T_theileri_Tth.43.1980/1-746   --------------------------------------------
T_theileri_Tth.32.2230/1-717   --------------------------------------------
T_theileri_Tth.11.3610/1-173   --------------------------------------------
T_theileri_Tth.46.1030/1-525   --------------------------------------------
T_theileri_Tth.36.1960/1-517   --------------------------------------------
T_theileri_Tth.165.1020/1-245  --------------------------------------------
T_theileri_Tth.46.1120/1-413   --------------------------------------------
T_theileri_Tth.24.2780/1-672   --------------------------------------------
T_theileri_Tth.8.4720/1-600    --------------------------------------------
T_theileri_Tth.165.1030/1-156  --------------------------------------------
T_theileri_Tth.6.5040/1-514    --------------------------------------------
T_theileri_Tth.31.1000/1-129   --------------------------------------------
T_theileri_Tth.11.3650/1-197   --------------------------------------------
T_theileri_Tth.19.2150/1-142   --------------------------------------------
T_theileri_Tth.4.4260/1-444    --------------------------------------------
T_theileri_Tth.70.1040/1-472   --------------------------------------------
T_theileri_Tth.46.1080/1-427   --------------------------------------------
T_theileri_Tth.251.1000/1-344  --------------------------------------------
T_theileri_Tth.27.1390/1-380   --------------------------------------------
T_theileri_Tth.12.1020/1-102   --------------------------------------------
T_theileri_Tth.12.1830/1-342   --------------------------------------------
T_theileri_Tth.11.3140/1-251   --------------------------------------------
T_theileri_Tth.286.1000/1-493  --------------------------------------------
T_theileri_Tth.61.1060/1-159   --------------------------------------------
T_theileri_Tth.10.2860/1-651   --------------------------------------------
T_theileri_Tth.11.2120/1-102   --------------------------------------------
T_theileri_Tth.132.1030/1-666  --------------------------------------------
T_theileri_Tth.101.1090/1-119  --------------------------------------------
T_theileri_Tth.23.2140/1-546   --------------------------------------------
T_theileri_Tth.10.2510/1-395   --------------------------------------------
T_theileri_Tth.107.1030/1-864  --------------------------------------------
T_theileri_Tth.31.1060/1-887   --------------------------------------------
T_theileri_Tth.132.1020/1-714  --------------------------------------------
T_theileri_Tth.136.1020/1-209  --------------------------------------------
T_theileri_Tth.129.1020/1-290  --------------------------------------------
T_theileri_Tth.71.1080/1-526   --------------------------------------------
T_theileri_Tth.36.2120/1-796   --------------------------------------------
T_theileri_Tth.63.1240/1-819   --------------------------------------------
T_theileri_Tth.11.3250/1-309   --------------------------------------------
T_theileri_Tth.11.3240/1-592   --------------------------------------------
T_theileri_Tth.12.1000/1-649   --------------------------------------------
T_theileri_Tth.83.1080/1-228   --------------------------------------------
T_theileri_Tth.85.1030/1-137   --------------------------------------------
T_theileri_Tth.71.1030/1-204   --------------------------------------------
T_theileri_Tth.97.1060/1-928   --------------------------------------------
T_theileri_Tth.7.4630/1-775    --------------------------------------------
T_theileri_Tth.24.1450/1-463   --------------------------------------------
T_theileri_Tth.6.1080/1-569    --------------------------------------------
T_theileri_Tth.14.1400/1-348   --------------------------------------------
T_theileri_Tth.21.1050/1-407   --------------------------------------------
T_theileri_Tth.144.1030/1-653  --------------------------------------------
T_theileri_Tth.269.1000/1-185  --------------------------------------------
T_theileri_Tth.144.1020/1-412  --------------------------------------------
T_theileri_Tth.151.1020/1-515  --------------------------------------------
T_theileri_Tth.11.3450/1-561   --------------------------------------------
T_theileri_Tth.166.1000/1-479  --------------------------------------------
T_theileri_Tth.54.1350/1-777   --------------------------------------------
T_theileri_Tth.10.2520/1-446   --------------------------------------------
```

```
                                        50        60        70        80
T_theileri_Tth.43.1980/1-746     --------------------------------------------
T_theileri_Tth.32.2230/1-717     --------------------------------------------
T_theileri_Tth.11.3610/1-173     --------------------------------------------
T_theileri_Tth.46.1030/1-525     --------------------------------------------
T_theileri_Tth.36.1960/1-517     --------------------------------------------
T_theileri_Tth.165.1020/1-245    --------------------------------------------
T_theileri_Tth.46.1120/1-413     --------------------------------------------
T_theileri_Tth.24.2780/1-672     --------------------------------------------
T_theileri_Tth.8.4720/1-600      --------------------------------------------
T_theileri_Tth.165.1030/1-156    --------------------------------------------
T_theileri_Tth.6.5040/1-514      --------------------------------------------
T_theileri_Tth.31.1000/1-129     --------------------------------------------
T_theileri_Tth.11.3650/1-197     --------------------------------------------
T_theileri_Tth.19.2150/1-142     --------------------------------------------
T_theileri_Tth.4.4260/1-444      --------------------------------------------
T_theileri_Tth.70.1040/1-472     --------------------------------------------
T_theileri_Tth.46.1080/1-427     --------------------------------------------
T_theileri_Tth.251.1000/1-344    --------------------------------------------
T_theileri_Tth.27.1390/1-380     --------------------------------------------
T_theileri_Tth.12.1020/1-102     --------------------------------------------
T_theileri_Tth.12.1830/1-342     --------------------------------------------
T_theileri_Tth.11.3140/1-251     --------------------------------------------
T_theileri_Tth.286.1000/1-493    --------------------------------------------
T_theileri_Tth.61.1060/1-159     --------------------------------------------
T_theileri_Tth.10.2860/1-651     --------------------------------------------
T_theileri_Tth.11.2120/1-102     --------------------------------------------
T_theileri_Tth.132.1030/1-666    --------------------------------------------
T_theileri_Tth.101.1090/1-119    --------------------------------------------
T_theileri_Tth.23.2140/1-546     --------------------------------------------
T_theileri_Tth.10.2510/1-395     --------------------------------------------
T_theileri_Tth.107.1030/1-864    --------------------------------------------
T_theileri_Tth.31.1060/1-887     --------------------------------------------
T_theileri_Tth.132.1020/1-714    --------------------------------------------
T_theileri_Tth.136.1020/1-209    --------------------------------------------
T_theileri_Tth.129.1020/1-290    --------------------------------------------
T_theileri_Tth.71.1080/1-526     --------------------------------------------
T_theileri_Tth.36.2120/1-796     --------------------------------------------
T_theileri_Tth.63.1240/1-819     --------------------------------------------
T_theileri_Tth.11.3250/1-309     --------------------------------------------
T_theileri_Tth.11.3240/1-592     --------------------------------------------
T_theileri_Tth.12.1000/1-649     --------------------------------------------
T_theileri_Tth.83.1080/1-228     --------------------------------------------
T_theileri_Tth.85.1030/1-137     --------------------------------------------
T_theileri_Tth.71.1030/1-204     --------------------------------------------
T_theileri_Tth.97.1060/1-928     --------------------------------------------
T_theileri_Tth.7.4630/1-775      --------------------------------------------
T_theileri_Tth.24.1450/1-463     --------------------------------------------
T_theileri_Tth.6.1080/1-569      --------------------------------------------
T_theileri_Tth.14.1400/1-348     --------------------------------------------
T_theileri_Tth.21.1050/1-407     --------------------------------------------
T_theileri_Tth.144.1030/1-653    --------------------------------------------
T_theileri_Tth.269.1000/1-185    --------------------------------------------
T_theileri_Tth.144.1020/1-412    --------------------------------------------
T_theileri_Tth.151.1020/1-515    --------------------------------------------
T_theileri_Tth.11.3450/1-561     --------------------------------------------
T_theileri_Tth.166.1000/1-479    --------------------------------------------
T_theileri_Tth.54.1350/1-777     --------------------------------------------
T_theileri_Tth.10.2520/1-446     --------------------------------------------
```

```
                                  90        100       110       120       130
T_theileri_Tth.43.1980/1-746    --------------------------------------------
T_theileri_Tth.32.2230/1-717    --------------------------------------------
T_theileri_Tth.11.3610/1-173    --------------------------------------------
T_theileri_Tth.46.1030/1-525    --------------------------------------------
T_theileri_Tth.36.1960/1-517    --------------------------------------------
T_theileri_Tth.165.1020/1-245   --------------------------------------------
T_theileri_Tth.46.1120/1-413    --------------------------------------------
T_theileri_Tth.24.2780/1-672    --------------------------------------------
T_theileri_Tth.8.4720/1-600     --------------------------------------------
T_theileri_Tth.165.1030/1-156   --------------------------------------------
T_theileri_Tth.6.5040/1-514     --------------------------------------------
T_theileri_Tth.31.1000/1-129    --------------------------------------------
T_theileri_Tth.11.3650/1-197    --------------------------------------------
T_theileri_Tth.19.2150/1-142    --------------------------------------------
T_theileri_Tth.4.4260/1-444     --------------------------------------------
T_theileri_Tth.70.1040/1-472    --------------------------------------------
T_theileri_Tth.46.1080/1-427    --------------------------------------------
T_theileri_Tth.251.1000/1-344   --------------------------------------------
T_theileri_Tth.27.1390/1-380    --------------------------------------------
T_theileri_Tth.12.1020/1-102    --------------------------------------------
T_theileri_Tth.12.1830/1-342    --------------------------------------------
T_theileri_Tth.11.3140/1-251    --------------------------------------------
T_theileri_Tth.286.1000/1-493   --------------------------------------------
T_theileri_Tth.61.1060/1-159    --------------------------------------------
T_theileri_Tth.10.2860/1-651    --------------------------------------------
T_theileri_Tth.11.2120/1-102    --------------------------------------------
T_theileri_Tth.132.1030/1-666   --------------------------------------------
T_theileri_Tth.101.1090/1-119   --------------------------------------------
T_theileri_Tth.23.2140/1-546    --------------------------------------------
T_theileri_Tth.10.2510/1-395    --------------------------------------------
T_theileri_Tth.107.1030/1-864   --------------------------------------------
T_theileri_Tth.31.1060/1-887    --------------------------------------------
T_theileri_Tth.132.1020/1-714   --------------------------------------------
T_theileri_Tth.136.1020/1-209   --------------------------------------------
T_theileri_Tth.129.1020/1-290   --------------------------------------------
T_theileri_Tth.71.1080/1-526    --------------------------------------------
T_theileri_Tth.36.2120/1-796    --------------------------------------------
T_theileri_Tth.63.1240/1-819    --------------------------------------------
T_theileri_Tth.11.3250/1-309    --------------------------------------------
T_theileri_Tth.11.3240/1-592    --------------------------------------------
T_theileri_Tth.12.1000/1-649    --------------------------------------------
T_theileri_Tth.83.1080/1-228    --------------------------------------------
T_theileri_Tth.85.1030/1-137    --------------------------------------------
T_theileri_Tth.71.1030/1-204    --------------------------------------------
T_theileri_Tth.97.1060/1-928    --------------------------------------------
T_theileri_Tth.7.4630/1-775     --------------------------------------------
T_theileri_Tth.24.1450/1-463    --------------------------------------------
T_theileri_Tth.6.1080/1-569     --------------------------------------------
T_theileri_Tth.14.1400/1-348    --------------------------------------------
T_theileri_Tth.21.1050/1-407    --------------------------------------------
T_theileri_Tth.144.1030/1-653   --------------------------------------------
T_theileri_Tth.269.1000/1-185   --------------------------------------------
T_theileri_Tth.144.1020/1-412   --------------------------------------------
T_theileri_Tth.151.1020/1-515   --------------------------------------------
T_theileri_Tth.11.3450/1-561    --------------------------------------------
T_theileri_Tth.166.1000/1-479   --------------------------------------------
T_theileri_Tth.54.1350/1-777    --------------------------------------------
T_theileri_Tth.10.2520/1-446    --------------------------------------------
```

```
                                               140        150        160        170
T_theileri_Tth.43.1980/1-746    --------------------------------------------
T_theileri_Tth.32.2230/1-717    ------------------MEKYFMRHLLYPVLLLFLLLCCAGTS
T_theileri_Tth.11.3610/1-173    ----MPQQANKLSVRTPATVCQSLYVMP-MLLLLLFLCCASVCV
T_theileri_Tth.46.1030/1-525    -----------------------MHRLLCTAL--LLLCCAYGC-
T_theileri_Tth.36.1960/1-517    --------------------------------------------
T_theileri_Tth.165.1020/1-245   --------------------------------------------
T_theileri_Tth.46.1120/1-413    -----------------------MRRLLCTAL--LLLCCAYGCI
T_theileri_Tth.24.2780/1-672    -----MPQQANKLSALTPATVCQSLYVMPLLLLLLFLCCASVCV
T_theileri_Tth.8.4720/1-600     ------------------MEKHSMRHLLWAAL--FLLYCSCGC-
T_theileri_Tth.165.1030/1-156   --------------------------------------------
T_theileri_Tth.6.5040/1-514     -------------------MKSHVHQLLCSALLLFLLCCAYGCA
T_theileri_Tth.31.1000/1-129    ---------------------------LCTAL--LLLCCAYGC-
T_theileri_Tth.11.3650/1-197    --------------------------------------------
T_theileri_Tth.19.2150/1-142    --------------------------------------------
T_theileri_Tth.4.4260/1-444     --------------------------------------------
T_theileri_Tth.70.1040/1-472    --------------------------------------------
T_theileri_Tth.46.1080/1-427    --------------------------------------------
T_theileri_Tth.251.1000/1-344   ------------------MEKHSMRHLLWTAL--FLLYCSCGC-
T_theileri_Tth.27.1390/1-380    --------------------------------------------
T_theileri_Tth.12.1020/1-102    ------------------MEKHSMRHLLWTAL--FLLYCSCGC-
T_theileri_Tth.12.1830/1-342    -----------------------MRRLLFAAL--LLLCCAYGCI
T_theileri_Tth.11.3140/1-251    --------------------------------------------
T_theileri_Tth.286.1000/1-493   --------------------------------------------
T_theileri_Tth.61.1060/1-159    ------------------------MIISVMLFLLLLPNCVSGLA
T_theileri_Tth.10.2860/1-651    ------------------MEKHSMRHLLWAAL--FLLYCSCGC-
T_theileri_Tth.11.2120/1-102    -------------------------MFVLLLLLLLFLCCASVCI
T_theileri_Tth.132.1030/1-666   ------------------MEKHSMHHLLWTAL--FLLYCSCGC-
T_theileri_Tth.101.1090/1-119   --------------------------------------------
T_theileri_Tth.23.2140/1-546    -------------------------MKFHTLLLFLFVFCVLKCT
T_theileri_Tth.10.2510/1-395    ------------------MEKHSMRHLLWTAL--FLLYCSCGC-
T_theileri_Tth.107.1030/1-864   LLLTDTQIHKPFKITREGMRRLFL----FAPL--LLLYCTLVT-
T_theileri_Tth.31.1060/1-887    -----------------------MRRLLCTAL--LLLCCAYGC-
T_theileri_Tth.132.1020/1-714   ------------------MEKHSMRYLLWAAL--FLLYCSCGC-
T_theileri_Tth.136.1020/1-209   --------------------------------------------
T_theileri_Tth.129.1020/1-290   ------------------MENYFMRHLLYPVLLLFLLLCCAGTS
T_theileri_Tth.71.1080/1-526    --------------------------------------------
T_theileri_Tth.36.2120/1-796    ------------------MRRLFL----FAPL--LLLYCTLVT-
T_theileri_Tth.63.1240/1-819    ------------------MEKHSMRHLLWAAL--FLLYCSCGC-
T_theileri_Tth.11.3250/1-309    --------------------------------------------
T_theileri_Tth.11.3240/1-592    -------------MNKTPHSLLFLWRRPLLLLLLLFLCCASVCV
T_theileri_Tth.12.1000/1-649    -----------------------MRHPLLQVVLLLFICGAVAGP
T_theileri_Tth.83.1080/1-228    --------------------------------------------
T_theileri_Tth.85.1030/1-137    ------------------MSRWQQTFLLLTAL---LLSSATVCF
T_theileri_Tth.71.1030/1-204    ------------------MKTLLEMIISVLLFLLLLPYCVYGLV
T_theileri_Tth.97.1060/1-928    ------------------MEKHSMRHLLWTAL--FLLYCSCGC-
T_theileri_Tth.7.4630/1-775     ---------------------MRFFLVLTTFVLLLLFQCVRSSL
T_theileri_Tth.24.1450/1-463    --------------------------------------------
T_theileri_Tth.6.1080/1-569     ------------MRTRSTAMHCFPHTVPLLLLLVVLVYTTVGCL
T_theileri_Tth.14.1400/1-348    --------------------------------LLLLLCCTAGCL
T_theileri_Tth.21.1050/1-407    -----------------------MRHLLCPVLL-LLLLCCAGTS
T_theileri_Tth.144.1030/1-653   -----------------------MRHPLLQVVLLLFICGAMAGP
T_theileri_Tth.269.1000/1-185   ----------------------------------------MAGP
T_theileri_Tth.144.1020/1-412   --------------------------------------------
T_theileri_Tth.151.1020/1-515   --------------------------------------------
T_theileri_Tth.11.3450/1-561    ---------------------MNFVQYVLLSLTLLLGCVFIGCF
T_theileri_Tth.166.1000/1-479   --------------------------------------------
T_theileri_Tth.54.1350/1-777    -------------ITREGMRRLFL----FAPL--LLLYCTLVT-
T_theileri_Tth.10.2520/1-446    --------------------------------------------
```

```
                                          180       190       200       210
T_theileri_Tth.43.1980/1-746    --------------------------------------------
T_theileri_Tth.32.2230/1-717    F-------------------------------------------
T_theileri_Tth.11.3610/1-173    A-------------------------------------------
T_theileri_Tth.46.1030/1-525    I-------------------------------------------
T_theileri_Tth.36.1960/1-517    --------------------------------------------
T_theileri_Tth.165.1020/1-245   --------------------------------------------
T_theileri_Tth.46.1120/1-413    --------------------------------------------
T_theileri_Tth.24.2780/1-672    A-------------------------------------------
T_theileri_Tth.8.4720/1-600     L-------------------------------------------
T_theileri_Tth.165.1030/1-156   --------------------------------------------
T_theileri_Tth.6.5040/1-514     A-------------------------------------------
T_theileri_Tth.31.1000/1-129    I-------------------------------------------
T_theileri_Tth.11.3650/1-197    --------------------------------------------
T_theileri_Tth.19.2150/1-142    --------------------------------------------
T_theileri_Tth.4.4260/1-444     --------------------------------------------
T_theileri_Tth.70.1040/1-472    --------------------------------------------
T_theileri_Tth.46.1080/1-427    --------------------------------------------
T_theileri_Tth.251.1000/1-344   L-------------------------------------------
T_theileri_Tth.27.1390/1-380    --------------------------------------------
T_theileri_Tth.12.1020/1-102    L-------------------------------------------
T_theileri_Tth.12.1830/1-342    A-------------------------------------------
T_theileri_Tth.11.3140/1-251    --------------------------------------------
T_theileri_Tth.286.1000/1-493   --------------------------------------------
T_theileri_Tth.61.1060/1-159    A-------------------------------------------
T_theileri_Tth.10.2860/1-651    L-------------------------------------------
T_theileri_Tth.11.2120/1-102    A-------------------------------------------
T_theileri_Tth.132.1030/1-666   L-------------------------------------------
T_theileri_Tth.101.1090/1-119   --------------------------------------------
T_theileri_Tth.23.2140/1-546    A-------------------------------------------
T_theileri_Tth.10.2510/1-395    L-------------------------------------------
T_theileri_Tth.107.1030/1-864   L-------------------------------------------
T_theileri_Tth.31.1060/1-887    I-------------------------------------------
T_theileri_Tth.132.1020/1-714   L-------------------------------------------
T_theileri_Tth.136.1020/1-209   --------------------------------------------
T_theileri_Tth.129.1020/1-290   F-------------------------------------------
T_theileri_Tth.71.1080/1-526    --------------------------------------------
T_theileri_Tth.36.2120/1-796    L-------------------------------------------
T_theileri_Tth.63.1240/1-819    L-------------------------------------------
T_theileri_Tth.11.3250/1-309    --------------------------------------------
T_theileri_Tth.11.3240/1-592    A-------------------------------------------
T_theileri_Tth.12.1000/1-649    A-------------------------------------------
T_theileri_Tth.83.1080/1-228    --------------------------------------------
T_theileri_Tth.85.1030/1-137    L-------------------------------------------
T_theileri_Tth.71.1030/1-204    A-------------------------------------------
T_theileri_Tth.97.1060/1-928    L-------------------------------------------
T_theileri_Tth.7.4630/1-775     A-------------------------------------------
T_theileri_Tth.24.1450/1-463    --------------------------------------------
T_theileri_Tth.6.1080/1-569     A-------------------------------------------
T_theileri_Tth.14.1400/1-348    A-------------------------------------------
T_theileri_Tth.21.1050/1-407    L-------------------------------------------
T_theileri_Tth.144.1030/1-653   A-------------------------------------------
T_theileri_Tth.269.1000/1-185   A-------------------------------------------
T_theileri_Tth.144.1020/1-412   --------------------------------------------
T_theileri_Tth.151.1020/1-515   --------------------------------------------
T_theileri_Tth.11.3450/1-561    A-------------------------------------------
T_theileri_Tth.166.1000/1-479   --------------------------------------------
T_theileri_Tth.54.1350/1-777    L-------------------------------------------
T_theileri_Tth.10.2520/1-446    --------------------------------------------
```

```
                                          230       240       250       260
T_theileri_Tth.43.1980/1-746     --------------------------------------------
T_theileri_Tth.32.2230/1-717     --------------------------------------------
T_theileri_Tth.11.3610/1-173     --------------------------------------------
T_theileri_Tth.46.1030/1-525     --------------------------------------------
T_theileri_Tth.36.1960/1-517     --------------------------------------------
T_theileri_Tth.165.1020/1-245    --------------------------------------------
T_theileri_Tth.46.1120/1-413     --------------------------------------------
T_theileri_Tth.24.2780/1-672     --------------------------------------------
T_theileri_Tth.8.4720/1-600      --------------------------------------------
T_theileri_Tth.165.1030/1-156    --------------------------------------------
T_theileri_Tth.6.5040/1-514      --------------------------------------------
T_theileri_Tth.31.1000/1-129     --------------------------------------------
T_theileri_Tth.11.3650/1-197     -------------------------------------------A
T_theileri_Tth.19.2150/1-142     --------------------------------------------
T_theileri_Tth.4.4260/1-444      --------------------------------------------
T_theileri_Tth.70.1040/1-472     --------------------------------------------
T_theileri_Tth.46.1080/1-427     --------------------------------------------
T_theileri_Tth.251.1000/1-344    --------------------------------------------
T_theileri_Tth.27.1390/1-380     --------------------------------------------
T_theileri_Tth.12.1020/1-102     --------------------------------------------
T_theileri_Tth.12.1830/1-342     --------------------------------------------
T_theileri_Tth.11.3140/1-251     --------------------------------------------
T_theileri_Tth.286.1000/1-493    --------------------------------------------
T_theileri_Tth.61.1060/1-159     ----------------------------------------LRDD
T_theileri_Tth.10.2860/1-651     --------------------------------------------
T_theileri_Tth.11.2120/1-102     --------------------------------------------
T_theileri_Tth.132.1030/1-666    --------------------------------------------
T_theileri_Tth.101.1090/1-119    --------------------------------------------
T_theileri_Tth.23.2140/1-546     --------------------------------------------
T_theileri_Tth.10.2510/1-395     --------------------------------------------
T_theileri_Tth.107.1030/1-864    --------------------------------------------
T_theileri_Tth.31.1060/1-887     --------------------------------------------
T_theileri_Tth.132.1020/1-714    --------------------------------------------
T_theileri_Tth.136.1020/1-209    --------------------------------------------
T_theileri_Tth.129.1020/1-290    --------------------------------------------
T_theileri_Tth.71.1080/1-526     --------------------------------------------
T_theileri_Tth.36.2120/1-796     --------------------------------------------
T_theileri_Tth.63.1240/1-819     --------------------------------------------
T_theileri_Tth.11.3250/1-309     --------------------------------------------
T_theileri_Tth.11.3240/1-592     --------------------------------------------
T_theileri_Tth.12.1000/1-649     --------------------------------------------
T_theileri_Tth.83.1080/1-228     --------------------------------------------
T_theileri_Tth.85.1030/1-137     ------------------------------------------AD
T_theileri_Tth.71.1030/1-204     ----------------------------------------LRDD
T_theileri_Tth.97.1060/1-928     --------------------------------------------
T_theileri_Tth.7.4630/1-775      --------------------------------------------
T_theileri_Tth.24.1450/1-463     --------------------------------------------
T_theileri_Tth.6.1080/1-569      --------------------------------------------
T_theileri_Tth.14.1400/1-348     --------------------------------------------
T_theileri_Tth.21.1050/1-407     --------------------------------------------
T_theileri_Tth.144.1030/1-653    --------------------------------------------
T_theileri_Tth.269.1000/1-185    --------------------------------------------
T_theileri_Tth.144.1020/1-412    --------------------------------------------
T_theileri_Tth.151.1020/1-515    --------------------------------------------
T_theileri_Tth.11.3450/1-561     -------------------------NNDNENNNNNDNNNNNNNN
T_theileri_Tth.166.1000/1-479    --------------------------------------------
T_theileri_Tth.54.1350/1-777     --------------------------------------------
T_theileri_Tth.10.2520/1-446     --------------------------------------------
```

```
                                  270       280       290       300
T_theileri_Tth.43.1980/1-746    --------------------------------------------
T_theileri_Tth.32.2230/1-717    ----------------------AAVVR----HLPQK--------
T_theileri_Tth.11.3610/1-173    QKDG--------------GVQSTGVVR----ELPRK--------
T_theileri_Tth.46.1030/1-525    ----------------------AAVVQ----PLPQK--------
T_theileri_Tth.36.1960/1-517    --------------------------------------------
T_theileri_Tth.165.1020/1-245   --------------------------------------------
T_theileri_Tth.46.1120/1-413    ----------------------AAVVQ----PLPKR--------
T_theileri_Tth.24.2780/1-672    --------------QNDGGVQSTGVVR----ELPRK--------
T_theileri_Tth.8.4720/1-600     ----------------------ADVVQ----QLPQK--------
T_theileri_Tth.165.1030/1-156   --------------------------------------------
T_theileri_Tth.6.5040/1-514     ----------------------AAVVK----PLPEK--------
T_theileri_Tth.31.1000/1-129    ----------------------AAVVQ----PLPQK--------
T_theileri_Tth.11.3650/1-197    QNDG--------------GVQSTGVVR----ELPRK--------
T_theileri_Tth.19.2150/1-142    --------------------------------------------
T_theileri_Tth.4.4260/1-444     --------------------------------------------
T_theileri_Tth.70.1040/1-472    --------------------------------------------
T_theileri_Tth.46.1080/1-427    --------------------------------------------
T_theileri_Tth.251.1000/1-344   ----------------------AAVVQ----QLPQK--------
T_theileri_Tth.27.1390/1-380    --------------------------------------------
T_theileri_Tth.12.1020/1-102    ----------------------AAVVQ----QLPQK--------
T_theileri_Tth.12.1830/1-342    ------------KTEYRTPYPPDPDLE----VFPET--------
T_theileri_Tth.11.3140/1-251    --------------------------------------------
T_theileri_Tth.286.1000/1-493   ------------DRVAEMSSPPVAVVR----EVPKR--------
T_theileri_Tth.61.1060/1-159    RCTYDTTVWKNMRNKTIYTPSSVSIVR----ELPQS--------
T_theileri_Tth.10.2860/1-651    ----------------------AAVVQ----HLPQK--------
T_theileri_Tth.11.2120/1-102    -------------QKDGGDAPSTGVVR----ELPRK--------
T_theileri_Tth.132.1030/1-666   ----------------------AAVVQ----QLPQK--------
T_theileri_Tth.101.1090/1-119   --------------------------------------------
T_theileri_Tth.23.2140/1-546    ----AVPNGCVFNEVMKMSGPPVSVVR----ELPKK--------
T_theileri_Tth.10.2510/1-395    ----------------------AAVVQ----QLPQK--------
T_theileri_Tth.107.1030/1-864   ----------------------AVTEH----HWCIHDRVSTFAG
T_theileri_Tth.31.1060/1-887    ----------------------AAVVQ----PLPQK--------
T_theileri_Tth.132.1020/1-714   ----------------------AAVVQ----QLPQK--------
T_theileri_Tth.136.1020/1-209   -----------MIPRVRVDPPAKVVVR----ELPRA--------
T_theileri_Tth.129.1020/1-290   ----------------------SAVVQ----HLPQR--------
T_theileri_Tth.71.1080/1-526    -----------MRNKTNYTPSSVSIVR----ELPQS--------
T_theileri_Tth.36.2120/1-796    ----------------------AVTEH----HRCMHDRVVMHVG
T_theileri_Tth.63.1240/1-819    ----------------------AAVVQ----QLPQK--------
T_theileri_Tth.11.3250/1-309    --------------------------------------------
T_theileri_Tth.11.3240/1-592    QKDG-------------GDAPSTGVVR----ELPRK--------
T_theileri_Tth.12.1000/1-649    -------YACNYDEIKKKSGSPVVVVR----EVPKS--------
T_theileri_Tth.83.1080/1-228    --------------------------------------------
T_theileri_Tth.85.1030/1-137    ATATSPDVHHCGFDDVMYKKGVQNIIK----IVPKQ--------
T_theileri_Tth.71.1030/1-204    RCTYDTTVWKNMKNKTVYTPLPVSIVR----ELPQS--------
T_theileri_Tth.97.1060/1-928    ----------------------AAVVQ----QLPQK--------
T_theileri_Tth.7.4630/1-775     -----------VTEYHHCIHDKISGFY----EDPKS--------
T_theileri_Tth.24.1450/1-463    --------------------------------------------
T_theileri_Tth.6.1080/1-569     ---SAGHLCNNDKITRRADMQPIAVVR----EMPQGR-------
T_theileri_Tth.14.1400/1-348    -----ASHHRCMHDKISELNKPVTVVR----EIPRK--------
T_theileri_Tth.21.1050/1-407    ----------------------AAVVQ----HLPQK--------
T_theileri_Tth.144.1030/1-653   -------YACNYDEIKKKNSPPVVVVR----ELPKK--------
T_theileri_Tth.269.1000/1-185   -------YACNYDEIKEKSGPPVVVVR----ELPKK--------
T_theileri_Tth.144.1020/1-412   --------------------------------------------
T_theileri_Tth.151.1020/1-515   -------------------VQSTGVVR----ELPRK--------
T_theileri_Tth.11.3450/1-561    NNNNKSTPFGFLEAMVNHTAIFTAILK----ELPRE--------
T_theileri_Tth.166.1000/1-479   --------------------------------------------
T_theileri_Tth.54.1350/1-777    ----------------------AVTEH----HRCMHDTVALRVG
T_theileri_Tth.10.2520/1-446    --------------------------------------------
```

```
                                 310       320       330       340       350
T_theileri_Tth.43.1980/1-746   --------------------------------------------
T_theileri_Tth.32.2230/1-717   -----------------GESAL-HAYTVAE--------------
T_theileri_Tth.11.3610/1-173   -----------------GQSGV-QAYTVAT--------------
T_theileri_Tth.46.1030/1-525   -----------------GQGPW-DTYTVAD-----------TET
T_theileri_Tth.36.1960/1-517   --------------------------------------------
T_theileri_Tth.165.1020/1-245  --------------------------------------------
T_theileri_Tth.46.1120/1-413   -----------------GQGPW-DTYTVSV-------ADTDDDD
T_theileri_Tth.24.2780/1-672   -----------------GQSGV-QAYTVAT--------------
T_theileri_Tth.8.4720/1-600    -----------------GQSAL-QAYTVSD--------------
T_theileri_Tth.165.1030/1-156  --------------------------------------------
T_theileri_Tth.6.5040/1-514    -----------------GQGAW-DAHTVAV--------------
T_theileri_Tth.31.1000/1-129   -----------------GQGPW-DAYVVSA------------PK
T_theileri_Tth.11.3650/1-197   -----------------GKSGV-QAYTVAT--------------
T_theileri_Tth.19.2150/1-142   --------------------------------------------
T_theileri_Tth.4.4260/1-444    --------------------------------------------
T_theileri_Tth.70.1040/1-472   --------------------------------------------
T_theileri_Tth.46.1080/1-427   --------------------------------------------
T_theileri_Tth.251.1000/1-344  -----------------GESAL-QAYTVSD--------------
T_theileri_Tth.27.1390/1-380   --------------------------------------------
T_theileri_Tth.12.1020/1-102   -----------------GESGL-QAYTVSD--------------
T_theileri_Tth.12.1830/1-342   -----------------EESRTWGMKVVNV--------------
T_theileri_Tth.11.3140/1-251   --------------------------------------------
T_theileri_Tth.286.1000/1-493  -----------------GQGAW-QTYTVAE---------LTAGV
T_theileri_Tth.61.1060/1-159   -----------------GKSSV-SLYTTTT-------ATTTTTN
T_theileri_Tth.10.2860/1-651   -----------------GESGL-QAYTVAT---------PTTTT
T_theileri_Tth.11.2120/1-102   -----------------GRSGV-QAYTVAT--------------
T_theileri_Tth.132.1030/1-666  -----------------GESAL-QAYTVSD--------T-PPAA
T_theileri_Tth.101.1090/1-119  --------------------------------------------
T_theileri_Tth.23.2140/1-546   -----------------GQGSL-QAYTASS--------------
T_theileri_Tth.10.2510/1-395   -----------------GESAL-QAYTVAT------------TA
T_theileri_Tth.107.1030/1-864  ADPEKARDSNQMTILSARGVSG-LSRNV---------RTK-PPG
T_theileri_Tth.31.1060/1-887   -----------------GQGPW-DTYTLSV------------TE
T_theileri_Tth.132.1020/1-714  -----------------GESAL-QAYTVST--------A-A---
T_theileri_Tth.136.1020/1-209  -----------------GDRASHQTHIVSS--------------
T_theileri_Tth.129.1020/1-290  -----------------GESAL-HAYTVAN--------------
T_theileri_Tth.71.1080/1-526   -----------------GKSIV-SLYTTTI-----------TTT
T_theileri_Tth.36.2120/1-796   ADHKKARESNQMTILSAKRDFG-LSRTV---------RNK-TPA
T_theileri_Tth.63.1240/1-819   -----------------GESAL-QAYTVSD----------TPKN
T_theileri_Tth.11.3250/1-309   --------------------------------------------
T_theileri_Tth.11.3240/1-592   -----------------GQSGM-QAYTVAT--------------
T_theileri_Tth.12.1000/1-649   -----------------GQGAW-QTYTVAT--------------
T_theileri_Tth.83.1080/1-228   --------------------------------------------
T_theileri_Tth.85.1030/1-137   -----------------GEESI-QPYLVST--------------
T_theileri_Tth.71.1030/1-204   -----------------GKSTV-SLYTTTS------------TT
T_theileri_Tth.97.1060/1-928   -----------------GQSAL-HAYTVST-------------D
T_theileri_Tth.7.4630/1-775    -----------------RKEVSHEVKVIET-------ESGNQEL
T_theileri_Tth.24.1450/1-463   --------------------------------------------
T_theileri_Tth.6.1080/1-569    -----------------KRGAW-QAYTASV--------------
T_theileri_Tth.14.1400/1-348   -----------------GQGAV-QAYTANA------------AE
T_theileri_Tth.21.1050/1-407   -----------------GESAF-HAYTVAN--------------
T_theileri_Tth.144.1030/1-653  -----------------GEGAW-QAYTVAT--------------
T_theileri_Tth.269.1000/1-185  -----------------GEGAW-QAYTVST--------------
T_theileri_Tth.144.1020/1-412  --------------------------------------------
T_theileri_Tth.151.1020/1-515  -----------------GQSGV-QAYTVAT--------------
T_theileri_Tth.11.3450/1-561   -----------------GKAAW-HALTAVR--------------
T_theileri_Tth.166.1000/1-479  --------------------------------------------
T_theileri_Tth.54.1350/1-777   VGPIFEYDKEIEKK---RESQQ-IRLSS---------NSQRSPA
T_theileri_Tth.10.2520/1-446   --------------------------------------------
```

```
                                     360       370       380       390
T_theileri_Tth.43.1980/1-746  --------------------------------------------
T_theileri_Tth.32.2230/1-717  ---DEKSGE----NWRPIRIKVSSDDVDK---------------
T_theileri_Tth.11.3610/1-173  -------QKKNDKEWKLIRIEAFTKDLED---------------
T_theileri_Tth.46.1030/1-525  DP-NSEKKK----GFQPIRFAVSAKEVDR---------------
T_theileri_Tth.36.1960/1-517  --------------------------------------------
T_theileri_Tth.165.1020/1-245 --------------------------------------------
T_theileri_Tth.46.1120/1-413  DP-TEKVKE----NWQPIRIAVSPKGVSD---------------
T_theileri_Tth.24.2780/1-672  --------QNENEGWELIRIKALTENLTE---------------
T_theileri_Tth.8.4720/1-600   -----TP-E----EWQPIRISVYTKFVED---------------
T_theileri_Tth.165.1030/1-156 --------------------------------------------
T_theileri_Tth.6.5040/1-514   --EDTKSED----AWKSIRIGVYPGGVSL---------------
T_theileri_Tth.31.1000/1-129  TVDDVPEKK----DFQPIRFAVSAKEVDR---------------
T_theileri_Tth.11.3650/1-197  --------QNENEEWKLIRIKASTKDLDN---------------
T_theileri_Tth.19.2150/1-142  --------------------------------------------
T_theileri_Tth.4.4260/1-444   --------------------------------------------
T_theileri_Tth.70.1040/1-472  --------------------------------------------
T_theileri_Tth.46.1080/1-427  --------------------------------------------
T_theileri_Tth.251.1000/1-344 ------TPE----EWHPIRIAVYTKFVED---------------
T_theileri_Tth.27.1390/1-380  --------------------------------------------
T_theileri_Tth.12.1020/1-102  -----TPDE----EWHPIQITVSAENVDQ---------------
T_theileri_Tth.12.1830/1-342  ----VPREEVKEGEWQSIRIAVNPGMIQD---------------
T_theileri_Tth.11.3140/1-251  --------------------------------------------
T_theileri_Tth.286.1000/1-493 TG-KEDENK----GWESLRINVSHKDLDD---------------
T_theileri_Tth.61.1060/1-159  TA-VKSEEE----GWMPIRIHVSSEELDR---------------
T_theileri_Tth.10.2860/1-651  TD-DDKTGP----DWKPIRIGVYTKFVED---------------
T_theileri_Tth.11.2120/1-102  ------SKR------QLIRIKTSMEDLKK---------------
T_theileri_Tth.132.1030/1-666 ---DDKNGE----KWEPIRFAVYTKYVED---------------
T_theileri_Tth.101.1090/1-119 --------------------------------------------
T_theileri_Tth.23.2140/1-546  -----------SNEWAPIRIVVSSEDLND---------------
T_theileri_Tth.10.2510/1-395  ES-DDKNGE----EWKPIRIAVYTKLVED---------------
T_theileri_Tth.107.1030/1-864 ER-EEP-QE----GWEPIRIKLVSDNLNK---------------
T_theileri_Tth.31.1060/1-887  TE-LERKKE----GFQPIRFAVSAKEIDR---------------
T_theileri_Tth.132.1020/1-714 -----KAGE----DWQPIRIAVYTKYVED---------------
T_theileri_Tth.136.1020/1-209 -------SD----KWEPLRINVSSLDLD----------------
T_theileri_Tth.129.1020/1-290 ---LEKSGR----NWKPIHIEVSAEGVNN---------------
T_theileri_Tth.71.1080/1-526  TA-VKSDEE----DWMPIRIHVSSDELDR---------------
T_theileri_Tth.36.2120/1-796  FR-EERIGE----EWKPIRIKLVTDHLMR---------------
T_theileri_Tth.63.1240/1-819  ---DDKNGP----EWQPIRIAVYTKTVED---------------
T_theileri_Tth.11.3250/1-309  --------------------------------------------
T_theileri_Tth.11.3240/1-592  ---------QENEKWEPIRIKAFTRDLED---------------
T_theileri_Tth.12.1000/1-649  SE-GNENDD----KWEPLRINVSYENLKE---------------
T_theileri_Tth.83.1080/1-228  --------------------------------------------
T_theileri_Tth.85.1030/1-137  -------DD----DWDTIRINVSMKDLQS---------------
T_theileri_Tth.71.1030/1-204  TT-VKSDEE----DWMPIRIHVSSDELDR---------------
T_theileri_Tth.97.1060/1-928  TN-VQTDDK----DWHPIRIGVYTKFVED---------------
T_theileri_Tth.7.4630/1-775   PQ-RSAPNK----EWNPIRIEVFTHDLEK---------------
T_theileri_Tth.24.1450/1-463  --------------------------------------------
T_theileri_Tth.6.1080/1-569   ----GNSED----GWTPLRIYVSTRDLEN---------------
T_theileri_Tth.14.1400/1-348  NSNIFGDNK----NWVPIRIKAFTRDLND---------------
T_theileri_Tth.21.1050/1-407  ---HGKSGR----NWKPIHIEVSAEDVDK---------------
T_theileri_Tth.144.1030/1-653 SE-DSNSNE----GWEALRIRVSTEDLNK---------------
T_theileri_Tth.269.1000/1-185 SN-KENSNE----GWEPLRIKVSYEDLKS---------------
T_theileri_Tth.144.1020/1-412 --------------------------------------------
T_theileri_Tth.151.1020/1-515 ---------QENEGWELIRIEVSTRDLKN---------------
T_theileri_Tth.11.3450/1-561  --------S----EWNPLRIAVSTLDLND---------------
T_theileri_Tth.166.1000/1-479 ---------------------VSAKEIDR---------------
T_theileri_Tth.54.1350/1-777  GR-EEPVGE----EWKPIRIKLVTDGLKK---------------
T_theileri_Tth.10.2520/1-446  --------------------------------------------
```

```
                                 400       410       420       430
T_theileri_Tth.43.1980/1-746  --------------------------------------------
T_theileri_Tth.32.2230/1-717  -----VLEECKRKLEIAKDRRPVWWSRWNEK-------------
T_theileri_Tth.11.3610/1-173  -----KSKYCTKAGQEVIDFDG----------------------
T_theileri_Tth.46.1030/1-525  -----VLRICEHLSDGLDVD-------QSEDEREYGFKLDINE-
T_theileri_Tth.36.1960/1-517  --------------------------------------------
T_theileri_Tth.165.1020/1-245 --------------------------------------------
T_theileri_Tth.46.1120/1-413  -----AVHYCRSRKLGARWGYPRKTN------------------
T_theileri_Tth.24.2780/1-672  -----HLNLCGKVKEIKQ-EEKEKREEVNGADYGQAENTKVSLA
T_theileri_Tth.8.4720/1-600   -----VVKECIKQRSEGDSNTTV---------------------
T_theileri_Tth.165.1030/1-156 --------------------------------------------
T_theileri_Tth.6.5040/1-514   -----WIKACANMRVSPFGPA-----------------------
T_theileri_Tth.31.1000/1-129  -----VLKYCKQWKKDPSAK--------DVYDNEYGFTLEVAN-
T_theileri_Tth.11.3650/1-197  -----KSRYCTQIGDKVMNFQD----------------------
T_theileri_Tth.19.2150/1-142  --------------------------------------------
T_theileri_Tth.4.4260/1-444   --------------------------------------------
T_theileri_Tth.70.1040/1-472  --------------------------------------------
T_theileri_Tth.46.1080/1-427  --------------------------------------------
T_theileri_Tth.251.1000/1-344 -----IVNKCKKQRSESDSNPT----------------------
T_theileri_Tth.27.1390/1-380  --------------------------------------------
T_theileri_Tth.12.1020/1-102  -----VIRRCMKRKELSEDDRAIXKKKKEK--------------
T_theileri_Tth.12.1830/1-342  -----EIVYCMSKGRKINGYTRRIRNG-----------------
T_theileri_Tth.11.3140/1-251  --------------------------------------------
T_theileri_Tth.286.1000/1-493 -----EKKYCKDQNQKAKSFWS----------------------
T_theileri_Tth.61.1060/1-159  -----AMRRCSSGVIGSLHR------------------------
T_theileri_Tth.10.2860/1-651  -----ILKECKEQRPESNSN------------------------
T_theileri_Tth.11.2120/1-102  -----KTKYCTKKKDTSGECE-----------------------
T_theileri_Tth.132.1030/1-666 -----VMNYC--NTKKMPPEYNEVEAMKKELES-----------
T_theileri_Tth.101.1090/1-119 --------------------------------------------
T_theileri_Tth.23.2140/1-546  -----PSKYCDESFRVRPDYWL----------------------
T_theileri_Tth.10.2510/1-395  -----IMEYCERKKQDNVFYNDLEEKFE----------------
T_theileri_Tth.107.1030/1-864 ------GEHCSHDGTT--IKTLL---------------------
T_theileri_Tth.31.1060/1-887  -----VLKYCKHHKDGKLDD-------AEKDQLEYGFKIDIEK-
T_theileri_Tth.132.1020/1-714 -----VMNYC--NKKEMPSEYDKTTDLKKNLES-----------
T_theileri_Tth.136.1020/1-209 -----EKKYCRGHLDERKSYRM----------------------
T_theileri_Tth.129.1020/1-290 -----VLRDCDRKRALAEQLKGTGTRRYD---------------
T_theileri_Tth.71.1080/1-526  -----AMRRCSSGVTGSSHR------------------------
T_theileri_Tth.36.2120/1-796  -----VGHDCTGKKNNN-ITTLL---------------------
T_theileri_Tth.63.1240/1-819  -----IMNNCEKTLKEYNKLKEELQKELEGELEGEFEEKVDDPE
T_theileri_Tth.11.3250/1-309  --------------------------------------------
T_theileri_Tth.11.3240/1-592  -----KSKYCTEVNQKIVSFFG----------------------
T_theileri_Tth.12.1000/1-649  ------DKYCVREKQELRDMLN----------------------
T_theileri_Tth.83.1080/1-228  --------------------------------------------
T_theileri_Tth.85.1030/1-137  -----ESKYCKKGDAEKRETRPDFL-------------------
T_theileri_Tth.71.1030/1-204  -----VMRRCSSGMTGSSHR------------------------
T_theileri_Tth.97.1060/1-928  -----VVKYCNAKERGKPSEYTELDNKLKELKEKLEDEEFYEEE
T_theileri_Tth.7.4630/1-775   -----QGRYCAKKGEEVKIDVF----------------------
T_theileri_Tth.24.1450/1-463  --------------------------------------------
T_theileri_Tth.6.1080/1-569   -----DEKYCVAEDEMRP-TFR----------------------
T_theileri_Tth.14.1400/1-348  -----PSKYCTVAGEERYDQTY----------------------
T_theileri_Tth.21.1050/1-407  -----VLKSCDRKRAWAERTGRSFLRTDE---------------
T_theileri_Tth.144.1030/1-653 ------KKYCVNYGERRKDFLD----------------------
T_theileri_Tth.269.1000/1-185 ------DKYCVKEKEKRMDFLN----------------------
T_theileri_Tth.144.1020/1-412 --------------------------------------------
T_theileri_Tth.151.1020/1-515 -----KSRYCNEENKEIKKYDG----------------------
T_theileri_Tth.11.3450/1-561  -----TNKYCNISGVIRQDLH-----------------------
T_theileri_Tth.166.1000/1-479 -----IVKFCEHLSNGLDED-------DYDDAKDYGFKLNFKE-
T_theileri_Tth.54.1350/1-777  -----TGHDCTGK-ETNNITTLL---------------------
T_theileri_Tth.10.2520/1-446  --------------------------------------------
```

```
                                        450       460       470       480
T_theileri_Tth.43.1980/1-746   -----------------------------------MAEKKEILL
T_theileri_Tth.32.2230/1-717   ---------------------NNVLCN---NETGITDEKKDLLL
T_theileri_Tth.11.3610/1-173   ---------------------KKAKC---EEAEVLTDGRCREYI
T_theileri_Tth.46.1030/1-525   -----------I---------NEDFCD---WPNKVTPDRKRTLL
T_theileri_Tth.36.1960/1-517   ------------------------VCT---DHDVLTPEKKKILV
T_theileri_Tth.165.1020/1-245  --------------------------------------------
T_theileri_Tth.46.1120/1-413   -----------F---------DQALCH---PTEGMTLERANLFV
T_theileri_Tth.24.2780/1-672   ---------------------DDKKC----DYKRASEEERDNLV
T_theileri_Tth.8.4720/1-600    -----------------------EFCS-GDN--QMTAAKMNTLF
T_theileri_Tth.165.1030/1-156  --------------------------------------------
T_theileri_Tth.6.5040/1-514    ---------------------IYPYCM---GESRITPEKVQ-LL
T_theileri_Tth.31.1000/1-129   -----------S---------RFHFCK---EDSKMTAAKKRILL
T_theileri_Tth.11.3650/1-197   ---------------------DIDDC---VASQILTEEKKSMFI
T_theileri_Tth.19.2150/1-142   --------------------------------------------
T_theileri_Tth.4.4260/1-444    --------------------------------------------
T_theileri_Tth.70.1040/1-472   --------------------------------------------
T_theileri_Tth.46.1080/1-427   --------------------------------------------
T_theileri_Tth.251.1000/1-344  -----------H---------NVEFCR-GDN--EMTTEKMNTLF
T_theileri_Tth.27.1390/1-380   --------------------------------------------
T_theileri_Tth.12.1020/1-102   --------------------------------------------
T_theileri_Tth.12.1830/1-342   -----------F---------DEKVCG--AVSGYAGVQRMRTLM
T_theileri_Tth.11.3140/1-251   --------------------------------------------
T_theileri_Tth.286.1000/1-493  -----------G---------NEEQC---KDEDVLNDEKKKELI
T_theileri_Tth.61.1060/1-159   ---------------------HSPSCR---DGNVVTPQKRDILL
T_theileri_Tth.10.2860/1-651   -----------P---------TVEFCG-GDN--QMTTEKMNTLF
T_theileri_Tth.11.2120/1-102   ---------------------DPDNC------------------
T_theileri_Tth.132.1030/1-666  -----------Q---------FKQLCE-GEN--KMTTKRMHTLF
T_theileri_Tth.101.1090/1-119  --------------------------------------------
T_theileri_Tth.23.2140/1-546   -----------G---------INTSC---NPLEVLTDAKKKRLL
T_theileri_Tth.10.2510/1-395   -----------Q---------FGDHCE-EENANVMTPQKKDILF
T_theileri_Tth.107.1030/1-864  -----------N---------TSFNCT---KDDILTPEKKKILV
T_theileri_Tth.31.1060/1-887   ---------------------HDDFCD-EAGPNVVTPEKKRTLL
T_theileri_Tth.132.1020/1-714  -----------Q---------FEKLCT-GEN--KMTTKRMHILF
T_theileri_Tth.136.1020/1-209  -----------G---------STLSCP---EDDVLSGDKKNTLL
T_theileri_Tth.129.1020/1-290  ---------------------DSVFCD---NKTGITDEKKDLLL
T_theileri_Tth.71.1080/1-526   ---------------------HSPSCR---GGNVLTPQKRDILL
T_theileri_Tth.36.2120/1-796   -----------E---------ENYTCT---DYDELTPEKENILI
T_theileri_Tth.63.1240/1-819   AFLEVFEDEIEQ---------LKRLCR-GND--RMTEDKKKVLF
T_theileri_Tth.11.3250/1-309   --------------------------------------------
T_theileri_Tth.11.3240/1-592   ---------------------NVVKC---FEADDLNDERHREYI
T_theileri_Tth.12.1000/1-649   -----------G---------ETVPC---APYEVIDEKKKNTIT
T_theileri_Tth.83.1080/1-228   --------------------------------------------
T_theileri_Tth.85.1030/1-137   -----------G---------ENVPCT---KEAEITPEKMKNLT
T_theileri_Tth.71.1030/1-204   ---------------------HSPSCR---DGSVLTPQKRDILL
T_theileri_Tth.97.1060/1-928   FAGEDTFNLEREVEELQKELNFDSRCK-----DGITEKIKKVLF
T_theileri_Tth.7.4630/1-775    -----------N---------SKHTCT---EEDVLTPEKKNILV
T_theileri_Tth.24.1450/1-463   --------------------------------------------
T_theileri_Tth.6.1080/1-569    -----------G---------SEMHC---TEGDLFTMQKKRELI
T_theileri_Tth.14.1400/1-348   ---------------------TKLYC---RANGVLTVRKKRIIE
T_theileri_Tth.21.1050/1-407   ---------------------DKALCD---DETGITDKKKDLLL
T_theileri_Tth.144.1030/1-653  -----------G---------EEVNC---NFHDIMLRGKEEAIK
T_theileri_Tth.269.1000/1-185  -----------G---------ESADC---HAHDIMLRGKEEAIK
T_theileri_Tth.144.1020/1-412  --------------------------------------------
T_theileri_Tth.151.1020/1-515  ---------------------ELYKCR--DKEDVLGVDTMKKFE
T_theileri_Tth.11.3450/1-561   -----------G---------KDFMC---TETSILTPEKIQLII
T_theileri_Tth.166.1000/1-479  -----------I---------NADFCY---GPNEVSPDRRKTLL
T_theileri_Tth.54.1350/1-777   -----------K---------EDFTCT---ENDILTAEKKKILV
T_theileri_Tth.10.2520/1-446   --------------------------------------------
```

```
                                         490       500       510       520
T_theileri_Tth.43.1980/1-746    NKVLPKAIKLHADRLKVKRLGENKKSASEKT--------NVDD-
T_theileri_Tth.32.2230/1-717    NKLLPAAIKLHTDRLSIERERGGVGVSASTM------TPLSSID
T_theileri_Tth.11.3610/1-173    NKIIPAAIKLHRDRLLVQPHKGKIIVPE---------LEDEGL-
T_theileri_Tth.46.1030/1-525    RKVLPAALKLHSERLSVKREEENLIVRKDV-------DKFSSK-
T_theileri_Tth.36.1960/1-517    EWILPEAAKLHSDRLLVKPLQSPIVVPT---------FRPENL-
T_theileri_Tth.165.1020/1-245   --------------------------------------------
T_theileri_Tth.46.1120/1-413    NKILRAAIRLHAERLKVKRESGKLIITGDTV------LFFGKL-
T_theileri_Tth.24.2780/1-672    NNVIPAAIKLHRDRLLVRPVSGKLKVPE---------FKDDSN-
T_theileri_Tth.8.4720/1-600     KDLLPEAIKLHRDRLNVQPLQSNLKIQEEEV------RWFTSN-
T_theileri_Tth.165.1030/1-156   --------------------------------------------
T_theileri_Tth.6.5040/1-514     RRVVSAAAKLHSERLRVKRVKGRLFLNKNGG------KDFKKN-
T_theileri_Tth.31.1000/1-129    MDVLPAALKLHSER------------------------------
T_theileri_Tth.11.3650/1-197    DHIIPGAIRMHRDRLLVQPQEGKLEVPN---------FENGNP-
T_theileri_Tth.19.2150/1-142    ---------LHQDRLLVKPLKGTFRVPY---------SSSGHP-
T_theileri_Tth.4.4260/1-444     --------------------------------------------
T_theileri_Tth.70.1040/1-472    --------------------------------------------
T_theileri_Tth.46.1080/1-427    --------------------------------------------
T_theileri_Tth.251.1000/1-344   KDVLPKAIKLHTDRLKVKQVEKSQNTRSAEV------NLLPEK-
T_theileri_Tth.27.1390/1-380    --------------------------------------------
T_theileri_Tth.12.1020/1-102    --------------------------------------------
T_theileri_Tth.12.1830/1-342    LKVMPEAMALHVERLKVERVKEALRINVPFDLKPI--SDAEGTM
T_theileri_Tth.11.3140/1-251    --------------------------------------------
T_theileri_Tth.286.1000/1-493   EKILPEAIKLHTDRLLVKPMKTPWNVIP---------FENTNF-
T_theileri_Tth.61.1060/1-159    NELLPAAITLHSERLLVVRSRFNL--------------------
T_theileri_Tth.10.2860/1-651    KDILPKAIKLHTDRLKVKQVGKSENIISEEL------NPLPEK-
T_theileri_Tth.11.2120/1-102    --------------------------------------------
T_theileri_Tth.132.1030/1-666   KDLLPQAIKLHRDRLKVQPVQGKLKLWKEDYY-NTE-NIFAKF-
T_theileri_Tth.101.1090/1-119   --------------------------------------------
T_theileri_Tth.23.2140/1-546    SEILPVAIKLHSDRLLVHRESKPVVVPT----------FTSEI-
T_theileri_Tth.10.2510/1-395    KEVLPKAIKLHADRLSVKVKQEEEDLKITNI--------IDQDY
T_theileri_Tth.107.1030/1-864   EEILPEAIKMHEERLLVKRLEGTIVVPK---------FSDPNSL
T_theileri_Tth.31.1060/1-887    RDVLPVALKLHSERLSVKRVE-KLELPLKES------VEFSSK-
T_theileri_Tth.132.1020/1-714   RDLLPQAIKLHTDRLKVKPLQGNLKLWKEDYEGDIS-HPLIRI-
T_theileri_Tth.136.1020/1-209   NKILPAAINLHSDILLVKQLETPFKVPRFNT-----------TL
T_theileri_Tth.129.1020/1-290   NKLLLAAIKLHTDRLNIEQKEGEVVVSTSTI------NSFSGE-
T_theileri_Tth.71.1080/1-526    NELLPAAIALHSERLLVVRSRFNSVIIQFISEM-----------
T_theileri_Tth.36.2120/1-796    NEILPEAIELHKERLFVQPLKDPIVVPE---------FSDPKGL
T_theileri_Tth.63.1240/1-819    TEVLPKAIKLHTDRLKVNHEERKTISSVGEQ------ENFNIG-
T_theileri_Tth.11.3250/1-309    --------------------------------------------
T_theileri_Tth.11.3240/1-592    NEIIPAAIKLHEERLRVQPHKGKIIVPE---------LEGDY--
T_theileri_Tth.12.1000/1-649    NKILPEAIKLHTDRLLVQRIKTPWKVPK---------FNESSV-
T_theileri_Tth.83.1080/1-228    --------------------------------------------
T_theileri_Tth.85.1030/1-137    DVIIPEAV------------------------------------
T_theileri_Tth.71.1030/1-204    NELLPAAIALHSERLLVVRSRFNLVIIQFISEM-----------
T_theileri_Tth.97.1060/1-928    KEVLPKAIKLHTDRLKVKQMERTPILSAGEN------KRIQNS-
T_theileri_Tth.7.4630/1-775     NTILKEAVKLHSDRLRVERLKGPLVVPK---------FGVGTK-
T_theileri_Tth.24.1450/1-463    --------------------SGKLRVPP---------FEVNND-
T_theileri_Tth.6.1080/1-569     DHIIPTAIKLHTDRLLVQRDTTNAILTALKT------IQSNTA-
T_theileri_Tth.14.1400/1-348    EQLIPEAIKMHTERIFVVRETGIVKVPS----------MEHTY-
T_theileri_Tth.21.1050/1-407    NKLLPDAIQLHTDRLSIERKEGKVVVSASTI------RSFSTE-
T_theileri_Tth.144.1030/1-653   NEILPKAIKLHTDRLLVQRLKTPLQVPK---------FSKPSV-
T_theileri_Tth.269.1000/1-185   NEILPKAIKLHTDRLLVQRMKTPLQVPK---------FSKPSV-
T_theileri_Tth.144.1020/1-412   --------------------------------------------
T_theileri_Tth.151.1020/1-515   DIIIPAAMKLHEDRLFVRRGSGKLRVPQ---------FQDESY-
T_theileri_Tth.11.3450/1-561    EYILPEAVQLHSSRLLVESLNENLLIP----------LFNDTI-
T_theileri_Tth.166.1000/1-479   REVLPAALKLHSERLSVEQVE-KLDLSFEIKDS----YPFLPK-
T_theileri_Tth.54.1350/1-777    EEILPEAIKLHKERLFVQPLNGSIVVPQ---------FSKSNGL
T_theileri_Tth.10.2520/1-446    --------------------------------------------
```

```
                                   530       540       550       560       570
T_theileri_Tth.43.1980/1-746     CHIFGD-LLD-NL-------HEIPDVDFMIFVSLS---VD----
T_theileri_Tth.32.2230/1-717     CPKKIS--ELSKK-------ESFPKADFVLFVGLAESQTP----
T_theileri_Tth.11.3610/1-173     CGKFTVP-EEH---------------------------------
T_theileri_Tth.46.1030/1-525     CPNVSI-PKEHHQ--------GISNADFVLYVGVT---DE----
T_theileri_Tth.36.1960/1-517     CSKFTV-PQEHKG-------KGVPEADIVLYAAAPTRL------
T_theileri_Tth.165.1020/1-245    --------------------------------------------
T_theileri_Tth.46.1120/1-413     CPEVKI-PSEHEK-------EGVANADFVLYAGVSIYKPD----
T_theileri_Tth.24.2780/1-672     CNFFTVP-EKHRT-------VGF-DADFALYVIAVQ--------
T_theileri_Tth.8.4720/1-600      CWGFII-PSEHST-------EGIPNADFMLYVNLG---TE----
T_theileri_Tth.165.1030/1-156    --------------------------------------------
T_theileri_Tth.6.5040/1-514      CPYAVA-HPVLFQ-------KGFPFVDFALFVSPTMSHED----
T_theileri_Tth.31.1000/1-129     --------------------------------------------
T_theileri_Tth.11.3650/1-197     CTHFTVP-SDHHS-------EGVENADFVLYVAAGT--------
T_theileri_Tth.19.2150/1-142     CSAFSIP-KDHHR-------PGISGFDTVMYVAAGPSHLD----
T_theileri_Tth.4.4260/1-444      --------------------------------------------
T_theileri_Tth.70.1040/1-472     ---------------------GIEAAK-----------------
T_theileri_Tth.46.1080/1-427     --------------------------------------------
T_theileri_Tth.251.1000/1-344    CRAIKG-NPLGKA-------KKIPDVDFMIYVGLS---DE----
T_theileri_Tth.27.1390/1-380     --------------------------------------------
T_theileri_Tth.12.1020/1-102     --------------------------------------------
T_theileri_Tth.12.1830/1-342     CAEMVPVFDGDEG-------MGIPDTDFVIYLGLSTKKP-----
T_theileri_Tth.11.3140/1-251     --------------------------------------------
T_theileri_Tth.286.1000/1-493    CSHFTIP-KKDDG-KLKVDEEKLPETNFMLYVATGSRGNN----
T_theileri_Tth.61.1060/1-159     --------------------------------------------
T_theileri_Tth.10.2860/1-651     CGPFKD-PLGKAQ--------KIPDVDFMIYVGLS---AK----
T_theileri_Tth.11.2120/1-102     --------------------------------------------
T_theileri_Tth.132.1030/1-666    CNQFFV-PEEHLK-------DGVSDADFLLYVRLS---PY----
T_theileri_Tth.101.1090/1-119    --------------------------------------------
T_theileri_Tth.23.2140/1-546     CSRFSVP-NTHRT-------AGVSGADMVLYLAATPSN------
T_theileri_Tth.10.2510/1-395     CVDFKI-PEEHKT-------HGIPDVDFMIYVSLS---TN----
T_theileri_Tth.107.1030/1-864    CFMFI--PQDHIG-------SSISATDMVLYVAATPTR-N----
T_theileri_Tth.31.1060/1-887     CPNVSV-PKEHKQ--------GISDADFMLYVGLT---EK----
T_theileri_Tth.132.1020/1-714    CDQFLV-PEEHLD-------SGVSDADFMLYVRLS---PY----
T_theileri_Tth.136.1020/1-209    CSHFTVP-PTHTS-------EGVKDTYIMLYIATGPSNTP----
T_theileri_Tth.129.1020/1-290    CGVIKA--WEHK--------KSFSNADFVLFVGLDESEMS----
T_theileri_Tth.71.1080/1-526     CYTYVELPAAYES-------VGVVQADFVLFVLAETVAPFVVIC
T_theileri_Tth.36.2120/1-796     CFRFI--PKDHKT-------RSVSGYDMVLFAAATPTT-N----
T_theileri_Tth.63.1240/1-819     CHFFKN-PLKLKV-------HQSEDFDFMIYVGLS---TS----
T_theileri_Tth.11.3250/1-309     --------------------------------------------
T_theileri_Tth.11.3240/1-592     CENFTVP-EEHRT-------EGV-DADFVFYVAAVP--------
T_theileri_Tth.12.1000/1-649     CSNFTSP-GDHTS-------QDVQDADFLLYVAAGPRDDY----
T_theileri_Tth.83.1080/1-228     --------------------------------------------
T_theileri_Tth.85.1030/1-137     --------------------------------------------
T_theileri_Tth.71.1030/1-204     CYTYVELPAAYES-------VGVVQADFVLFVLAETVAPFVV--
T_theileri_Tth.97.1060/1-928     CQFFKD-SFKSNV-------QDSSDFDFMIFVGLS---DE----
T_theileri_Tth.7.4630/1-775      CATFTV-PDKHHT-------DGVPDVDMVLYVSARPSKYS----
T_theileri_Tth.24.1450/1-463     CKYFTVP-DEHRS-------EGVENADFVLYVAAGT--------
T_theileri_Tth.6.1080/1-569      CRYFTIP-QEHVD-------NGVEDADMVLYFASGPGE------
T_theileri_Tth.14.1400/1-348     CSHYTIP-EEHHT-------IGLQDAELYFYVSALQTD------
T_theileri_Tth.21.1050/1-407     CKVTVA--SDHKT-------NGFPNADFVLFVGLAASQTP----
T_theileri_Tth.144.1030/1-653    CSHFTSP-GDHAS-------HGVQDADFLLYVAAGPSKNT----
T_theileri_Tth.269.1000/1-185    CSHFTIP-GDRAP-------YGVENADFLLYVAAGPSKKL----
T_theileri_Tth.144.1020/1-412    --------------------------------------------
T_theileri_Tth.151.1020/1-515    CKHFTVP-DEHHS-------DGVDNADFVLYVAAVP--------
T_theileri_Tth.11.3450/1-561     CSSFTVP-ADHRR-------IGVKDADMILYVAAKPASG-----
T_theileri_Tth.166.1000/1-479    CTDVSI-PKEHQQ--------GITKADFMLYVGVT---DE----
T_theileri_Tth.54.1350/1-777     CSQFI--PTTVES-------TAISDYDMVLYVAATPTP-E----
T_theileri_Tth.10.2520/1-446     --------------------------------------------
```

```
                                              580       590       600       610
T_theileri_Tth.43.1980/1-746     --NRDF---AICSKD-NQG------RPTSAVIKFDVKEIK----
T_theileri_Tth.32.2230/1-717     --------TRVCSEE-ENK------RPTSALIHFVPKEIV----
T_theileri_Tth.11.3610/1-173     --------------------------------------------
T_theileri_Tth.46.1030/1-525     --HVPV---QICSNN-TEG------RPTSALIKFIPKEID----
T_theileri_Tth.36.1960/1-517     --GTFAWAATCATLG-RNG------RPVVGIINYGPRYIV----
T_theileri_Tth.165.1020/1-245    --------------------------------------------
T_theileri_Tth.46.1120/1-413     --------TVVCSYN-LQK------RPIAASMNIRPKDIA----
T_theileri_Tth.24.2780/1-672     ---KMRFGYTCAVEN-STG------RPIVGAVSYAPESND----
T_theileri_Tth.8.4720/1-600      --RKSS---AVCSID-ENK------RPTSARISFVPKEIV----
T_theileri_Tth.165.1030/1-156    --------------------------------------------
T_theileri_Tth.6.5040/1-514      --------PKICTRG-DDG------RPTSALIKLNPNAIK----
T_theileri_Tth.31.1000/1-129     --------------------------------------------
T_theileri_Tth.11.3650/1-197     ---GEPFGVTCAREE-SSG------RPIAGAMNFPPYYLV----
T_theileri_Tth.19.2150/1-142     --GNVAWAILCATL--TDG------RPVAGGIYLSPREIA----
T_theileri_Tth.4.4260/1-444      --------------------------------------------
T_theileri_Tth.70.1040/1-472     --------------------------------------------
T_theileri_Tth.46.1080/1-427     -----MTSAFVCSEG-VNK------RPTSGLISFYPKDIV----
T_theileri_Tth.251.1000/1-344    --PKNV---EICTQD-NEN------RPTSAVISFIPKEIN----
T_theileri_Tth.27.1390/1-380     --------------------------------------------
T_theileri_Tth.12.1020/1-102     --------------------------------------------
T_theileri_Tth.12.1830/1-342     -------GTKICTYD-KDG------RPTSAMIKLNPFEIQ----
T_theileri_Tth.11.3140/1-251     --------------------------------------------
T_theileri_Tth.286.1000/1-493    --APRSWALTCAVDANDNN------RPIIGAMRVNPKNIA----
T_theileri_Tth.61.1060/1-159     --------------------------------------------
T_theileri_Tth.10.2860/1-651     --REKV---EICTQD-NEK------RPTSAVIKFIPKEIN----
T_theileri_Tth.11.2120/1-102     --------------------------------------------
T_theileri_Tth.132.1030/1-666    --GQDY---HICTYD-EKKP----NRPRSARISFVPKEIN----
T_theileri_Tth.101.1090/1-119    --------------------------------------LV----
T_theileri_Tth.23.2140/1-546     -----VWAVPCAE---KEK------RTIVATMNVSPSYIE----
T_theileri_Tth.10.2510/1-395     --SIGR---WICSRG-GEN------RPTSALIKFVPEYIE----
T_theileri_Tth.107.1030/1-864    --RTFAWAATCATLG-PDG------RPVIGIINYGPRYIV----
T_theileri_Tth.31.1060/1-887     --YVPV---QICSYD-TQE------RPTSALIKFIPKEID----
T_theileri_Tth.132.1020/1-714    --GQDY---HICTRD-KNNQ----VRPTSARISFVPKEIN----
T_theileri_Tth.136.1020/1-209    --FIDNYNTS--------GN----ECLGCGERKNIPTEKS----
T_theileri_Tth.129.1020/1-290    --------TIVCSQD-LEE------RPTSALIKFVPKDIV----
T_theileri_Tth.71.1080/1-526     SEADDG-------------------RPTSAAMNFAPADIV----
T_theileri_Tth.36.2120/1-796     --GAFAWAATCATLG-HDG------RPVIGIINYGPRYIV----
T_theileri_Tth.63.1240/1-819     --GKTV---EICTQE-NENG-----RPTSAAIRFIPDEIK----
T_theileri_Tth.11.3250/1-309     --------------------------------------------
T_theileri_Tth.11.3240/1-592     --WHYTFGLTCATEE-SSG------RPIVGVMNFIPSLYG----
T_theileri_Tth.12.1000/1-649     --ASATWAVTCAIDG-QTN------RPMVGAMNIHPMHAD----
T_theileri_Tth.83.1080/1-228     --------------------------------------------
T_theileri_Tth.85.1030/1-137     --------------------------------------------
T_theileri_Tth.71.1030/1-204     --------------------------------------------
T_theileri_Tth.97.1060/1-928     --PKNV---EICSKD-DEK------RPTSAVIKFVPKEIK----
T_theileri_Tth.7.4630/1-775      --GSFAWALTCATLGNETAR----GRSVVGVMNYAPKYIL----
T_theileri_Tth.24.1450/1-463     ---RDPFGVTCAVEN-STG------RPIVGAMNYVPQLQD----
T_theileri_Tth.6.1080/1-569      -----TFAVTCATLE-KGG------RPIVGAMNFRMSDVF----
T_theileri_Tth.14.1400/1-348     --RAMAWGAYCACT--DDG------RPIVGSLNLNPTYVE----
T_theileri_Tth.21.1050/1-407     --------TRICSQE-LDK------RPTSALIHFVPKDIV----
T_theileri_Tth.144.1030/1-653    --NHSSWAVTCAVD--QTK------RPIVGAMNIHPLHAD----
T_theileri_Tth.269.1000/1-185    --TSFTWAVTCAIDD-QSK-------------------------
T_theileri_Tth.144.1020/1-412    --------------------------------------------
T_theileri_Tth.151.1020/1-515    ---LSSFGVTCAVEN-STG------RPIVGAVNYAPTTYE----
T_theileri_Tth.11.3450/1-561     --VDVAWAKSCAKM--KNG------RPIAGVINFIPQSIS----
T_theileri_Tth.166.1000/1-479    --HQHP---KICSRN-TKG------RPTSALIKFVPEEIA----
T_theileri_Tth.54.1350/1-777     --GTFAWAATCATLG-RNG------RPVIGIINYGPRYIV----
T_theileri_Tth.10.2520/1-446     --------------------------------------------
```

```
                                 620       630       640       650
                                   |         |         |         |
T_theileri_Tth.43.1980/1-746   --ATRQFIRLTAHEIAHGLGFEYKFMDRL-GM-----IKTKY--
T_theileri_Tth.32.2230/1-717   --DTRHFVRTAAHEIAHLLGFEVGRMQNY-DR-----IKYGT--
T_theileri_Tth.11.3610/1-173   --------------------------------------------
T_theileri_Tth.46.1030/1-525   --DTRYFIRFAAHEVAHALGFDIEIMKEN-NV-----IEEKL--
T_theileri_Tth.36.1960/1-517   --AIPQRVRVAAHEIAHALGFSADVMDEK-RM-----VESDE--
T_theileri_Tth.165.1020/1-245  --------------------------------------------
T_theileri_Tth.46.1120/1-413   --DTRHFTRIVAHELGHGLGFDNMALNKV-SA-----LVSVV--
T_theileri_Tth.24.2780/1-672   NRGTRFNVRRVAHEMAHALGFEYELMKGR-RM-----LSEVD--
T_theileri_Tth.8.4720/1-600    --DTRHYIRLAAHDIAVGLGFTTEY-MSK-YL-----TTASA--
T_theileri_Tth.165.1030/1-156  --------------------------------------------
T_theileri_Tth.6.5040/1-514    --NTRFHIRSAAHDIGHGLGFLLFMFKSY-GW-----VKESE--
T_theileri_Tth.31.1000/1-129   --------------------------------------------
T_theileri_Tth.11.3650/1-197   --DPRLNVRIAAHEMAHA--------------------------
T_theileri_Tth.19.2150/1-142   --NTSQMVRVVAHEMAHILGFDREVFSAN-KM-----ISLVH--
T_theileri_Tth.4.4260/1-444    ---------TVAHEIAHGLGFDGTTFAHL-KM-----ISAVE--
T_theileri_Tth.70.1040/1-472   --------------------------------------------
T_theileri_Tth.46.1080/1-427   --DTRHFIRLTAHEIAHALGFELNRMKDL-QM-----IMQRTVN
T_theileri_Tth.251.1000/1-344  --ATRQYIRLTAHEIAHGLGIQHDLMKGL-FM-----INERS--
T_theileri_Tth.27.1390/1-380   ------NVRVVTHEIAHALGFSIDTLMVH-TS-----LVAMP--
T_theileri_Tth.12.1020/1-102   --------------------------------------------
T_theileri_Tth.12.1830/1-342   --YTTKFVRLVAHEIAHGLGFSTDVKKFK-EM-----VRSGD--
T_theileri_Tth.11.3140/1-251   --------------------------------------------
T_theileri_Tth.286.1000/1-493  --FGKGIVRLLAHELGHALGFDYERMNER-GM-----ITVRD--
T_theileri_Tth.61.1060/1-159   --------------------------------------------
T_theileri_Tth.10.2860/1-651   --AAWQYIRVTAHEIAHGLGFQYELMEKL-GM-----IELNN--
T_theileri_Tth.11.2120/1-102   --------------------------------------------
T_theileri_Tth.132.1030/1-666  --ATRHFIRLAARFIAFGLGFGEFV--DT-S------LSGTL--
T_theileri_Tth.101.1090/1-119  --DPRLNVRIAAHEMAHALGFNIPSMKQK-GV-----LTQAN--
T_theileri_Tth.23.2140/1-546   --EIQVISRLVAHELGHALGFNYNQFITL-GM-----TKEVQ--
T_theileri_Tth.10.2510/1-395   --ATRQYIRLTAHEIAHGLGFDHKVMETL-VM-----IEPRE--
T_theileri_Tth.107.1030/1-864  --ATPQRVRVAAHEIAHALGFNFERMKRK-SM-----VRNDV--
T_theileri_Tth.31.1060/1-887   --DTRYFIRLTAHEVAHALGFEIEMMKKH-NV-----IAEEN--
T_theileri_Tth.132.1020/1-714  --ATRYQIRLAARNIAFGLGFGEFV--DI-EP-----IAGTL--
T_theileri_Tth.136.1020/1-209  --INRRRIQLHVHGITAILQGEKNNKNNYRE-------------
T_theileri_Tth.129.1020/1-290  --DTRHFVRTAAHEIAHGLGFEVTRMRKL-EL-----IKYGA--
T_theileri_Tth.71.1080/1-526   --NTRLFTRIIAHNLAHALGFDVRRISGM-GMTARSGITTTT--
T_theileri_Tth.36.2120/1-796   --ATPQRVRVAAHEIAHALGFNSQLMERK-SM-----VRRDV--
T_theileri_Tth.63.1240/1-819   --ATRQFIRFTAHEIAHGLGFQLDLMKTL-GM-----IKTRD--
T_theileri_Tth.11.3250/1-309   --------------------------------------------
T_theileri_Tth.11.3240/1-592   --GLRLNVRRAAHEMAHALGFNVIDMGEK-NM-----LSHVI--
T_theileri_Tth.12.1000/1-649   --FTRAYVRFIAHQLAHALGFDYERMKWR-GV-----TRDSS--
T_theileri_Tth.83.1080/1-228   --------------------------------------------
T_theileri_Tth.85.1030/1-137   --------------------------------------------
T_theileri_Tth.71.1030/1-204   --------------------------------------------
T_theileri_Tth.97.1060/1-928   --ATRHFIRLTAHEIAHGLGFQHDLMKQL-NM-----IKERE--
T_theileri_Tth.7.4630/1-775    --ATAQRVRVAAHEIAHVLGFSVSEMDRL-KM-----IKDLG--
T_theileri_Tth.24.1450/1-463   --GIRFDVRRVAREIAHALGFSFVEMEKK-GI-----VTEVE--
T_theileri_Tth.6.1080/1-569    --AASIAARVAAHEIAHALGFNYKMMREK-NM-----LSESY--
T_theileri_Tth.14.1400/1-348   --ATDAAVRTVAHEIAHLLGFDYYMFLKL-GI-----VDTGV--
T_theileri_Tth.21.1050/1-407   --DTRHFVRTAAHEIAHGLGFLVNRMKDL-GL-----IKYGA--
T_theileri_Tth.144.1030/1-653  --STRVNVRLAAHKLAHALGFDYERMKGR-GM-----TRHSS--
T_theileri_Tth.269.1000/1-185  --------------------------------------------
T_theileri_Tth.144.1020/1-412  --------------------------------------------
T_theileri_Tth.151.1020/1-515  --YMRFDVRRVAQLIAHALGFNLPEMERK-GI-----VTEVE--
T_theileri_Tth.11.3450/1-561   --LTRISIRITAHEIAHTLGFNYQQMVDL-NM-----ISTVP--
T_theileri_Tth.166.1000/1-479  --ATRHYIRLTAHEVAHALGFEIETMKEH-VK-----EGEET--
T_theileri_Tth.54.1350/1-777   --ATPQRVRVAAHEIAHALGFNLPEMERK-GM-----GPKWY--
T_theileri_Tth.10.2520/1-446   --------------------------------------------
```

```
                                        670       680       690       700
T_theileri_Tth.43.1980/1-746    -SYQQ------PTGGLGAGK---QLYMVELKNAVDK-LKEHLNC
T_theileri_Tth.32.2230/1-717    ------------------VASNKKENAVYSEIMEKK-MREHYGC
T_theileri_Tth.11.3610/1-173    --------------------------------------------
T_theileri_Tth.46.1030/1-525    -TD---------------KK---GVPLVKSKTVLDRVVKEHYGC
T_theileri_Tth.36.1960/1-517    -IYPG-------------GP---QQLYVVSPNTRRA-ARKHFGC
T_theileri_Tth.165.1020/1-245   --------------------------------------------
T_theileri_Tth.46.1120/1-413    ------------------RND--VHYELRHALIKEK-VSEHYGC
T_theileri_Tth.24.2780/1-672    -------------------PIEETRALLNSTKTVGK-AREHYGC
T_theileri_Tth.8.4720/1-600     -SIFM------NG-V---PK---WLMMVQSNTVKDR-AVKHYGC
T_theileri_Tth.165.1030/1-156   --------------------------------------------
T_theileri_Tth.6.5040/1-514     -TYTGR-----------------PVVVVQTNKLGEK-VQEHYGC
T_theileri_Tth.31.1000/1-129    --------------------------------------------
T_theileri_Tth.11.3650/1-197    --------------------------------------------
T_theileri_Tth.19.2150/1-142    -DVRG-------------KS---NVHMLTSEKVMEK-AR-----
T_theileri_Tth.4.4260/1-444     NDVRG-------------KP---HVFLVVSPKAKEV-AQKYYNC
T_theileri_Tth.70.1040/1-472    --------------------------------------------
T_theileri_Tth.46.1080/1-427    GV---------------------NVPEVRSETVVAK-VKEHFGC
T_theileri_Tth.251.1000/1-344   -DDLL------RDEKLNGRN---KFYM-NSTKFVDV-LKKHYNC
T_theileri_Tth.27.1390/1-380    -GLRG-------------KN---SALVEASPRTLEK-TRAHFNC
T_theileri_Tth.12.1020/1-102    --------------------------------------------
T_theileri_Tth.12.1830/1-342    ------------------NSSYKGYNELSSSQVKNA-LQDHFNC
T_theileri_Tth.11.3140/1-251    --------------------------------------------
T_theileri_Tth.286.1000/1-493   -IRGE------------------DRIVVKSNKTLMK-AKEHYDC
T_theileri_Tth.61.1060/1-159    --------------------------------------------
T_theileri_Tth.10.2860/1-651    -SDLQ------SSQGSSSVK---TSRMVKFEKSVDK-LKSHYKC
T_theileri_Tth.11.2120/1-102    -------------------------------------NKDHFG-
T_theileri_Tth.132.1030/1-666   -PIDV------NG-E---SR---STKVVLSTNVKKK-MVEYYKC
T_theileri_Tth.101.1090/1-119   --IRGK-----------------NRMVVSSKMTKEK-AQGHYGC
T_theileri_Tth.23.2140/1-546    -GIRG-------------KET--NVLLINTSMTKKK-AEEHYNC
T_theileri_Tth.10.2510/1-395    -NDLL------STGGSSSAK---KFYM-NSSETLEI-LKKHYNC
T_theileri_Tth.107.1030/1-864   -KLRD-------------KT---DVFVVSSENTRRE-AMDHYKC
T_theileri_Tth.31.1060/1-887    -TG---------------TN---KDQLVASNALIDKKMKEQYDC
T_theileri_Tth.132.1020/1-714   -PITV------NGVP---KR---STKVVLSGNVKKK-MVEHYNC
T_theileri_Tth.136.1020/1-209   --------------------------------------------
T_theileri_Tth.129.1020/1-290   ------------------LRGKGKVTAVDSKIMQEK-MREHYDC
T_theileri_Tth.71.1080/1-526    -------------------AEERVMFTVNSHSTVEA-AKWHYGC
T_theileri_Tth.36.2120/1-796    -ELRG-------------KS---HLALVASENTLRE-VVKHYNC
T_theileri_Tth.63.1240/1-819    -NDLF------YNPVVV-EK---KYYMVNSSKTVEM-MEKHYNC
T_theileri_Tth.11.3250/1-309    --------------------------------------------
T_theileri_Tth.11.3240/1-592    --IRGN-----------------NRTVVSSELTKKK-AQEHYDC
T_theileri_Tth.12.1000/1-649    -RHEK------------------DRVLIDSEKVLEK-AKEHYNC
T_theileri_Tth.83.1080/1-228    --------------------------------------------
T_theileri_Tth.85.1030/1-137    --------------------------------------------
T_theileri_Tth.71.1030/1-204    --------------------------------------------
T_theileri_Tth.97.1060/1-928    -SGVA------HMQRHILAR---KYFMVNSSKTVEM-MKNHYKC
T_theileri_Tth.7.4630/1-775     -EVRG-------------RE---KLYVVSSPNTLRE-TRSHYNC
T_theileri_Tth.24.1450/1-463    --LRGE-----------------KRKVVSSTKTVEK-AQKHYNC
T_theileri_Tth.6.1080/1-569     -SVRG-------------KT---DVKLVISDKTKEE-AQKHYKC
T_theileri_Tth.14.1400/1-348    -TLRQR-----------------KVAVINTTQSKKV-ASEHYGC
T_theileri_Tth.21.1050/1-407    ------------------MGA--NKMAVYSKIMQRK-MRKHYDC
T_theileri_Tth.144.1030/1-653   -NYHD------------------NRAVIDSAKVLEV-AKEHYKC
T_theileri_Tth.269.1000/1-185   --------------------------------------------
T_theileri_Tth.144.1020/1-412   --------------------------------------------
T_theileri_Tth.151.1020/1-515   --LRGA-----------------KRKVVNSSKTVEK-AQKHYNC
T_theileri_Tth.11.3450/1-561    -HVRG-------------KP---SVMVVNSTLTAKK-AKEFYGC
T_theileri_Tth.166.1000/1-479   -TG---------------KK---KVRLVTYPTVIKE-VNKHYGC
T_theileri_Tth.54.1350/1-777    -NGYL-------------RF---DVQHVNSLNVLRE-TKNHYNC
T_theileri_Tth.10.2520/1-446    --------------------------------------------
```

```
                                          710       720       730       740
T_theileri_Tth.43.1980/1-746     DSG---E---IEGLYLENE-------------------------
T_theileri_Tth.32.2230/1-717     VAQL------VTGMYMEDE-------------------------
T_theileri_Tth.11.3610/1-173     --------------------------------------------
T_theileri_Tth.46.1030/1-525     SNSD-----EIKGLPLQSK-------------------------
T_theileri_Tth.36.1960/1-517     GS--------LESVELDFN-------------------------
T_theileri_Tth.165.1020/1-245    --------------------------------------------
T_theileri_Tth.46.1120/1-413     PA--------ATGMRLEKD-------------------------
T_theileri_Tth.24.2780/1-672     PS--------LTVMELEAH-------------------------
T_theileri_Tth.8.4720/1-600      Q---GQT---LKGMILDNK-------------------------
T_theileri_Tth.165.1030/1-156    --------------------------------------------
T_theileri_Tth.6.5040/1-514      RT--------INTLQLESY-------------------------
T_theileri_Tth.31.1000/1-129     --------------------------------------------
T_theileri_Tth.11.3650/1-197     --------------------------------------------
T_theileri_Tth.19.2150/1-142     --------------------------------------------
T_theileri_Tth.4.4260/1-444      SK--------APGLELEDQ-------------------------
T_theileri_Tth.70.1040/1-472     --------------------------------------------
T_theileri_Tth.46.1080/1-427     DN--------ATGIYMESE-------------------------
T_theileri_Tth.251.1000/1-344    GDGEANT---VKGLYLENE-------------------------
T_theileri_Tth.27.1390/1-380     ST--------APGMELEDE-------------------------
T_theileri_Tth.12.1020/1-102     --------------------------------------------
T_theileri_Tth.12.1830/1-342     DD--------YIGMKLDNS-------------------------
T_theileri_Tth.11.3140/1-251     --------------------------------------------
T_theileri_Tth.286.1000/1-493    DT--------MEGVELQVT-------------------------
T_theileri_Tth.61.1060/1-159     --------------------------------------------
T_theileri_Tth.10.2860/1-651     KEDDQ-----VKGLFLENE-------------------------
T_theileri_Tth.11.2120/1-102     --------------------------------------------
T_theileri_Tth.132.1030/1-666    -----PG---YAGMILRYN-------------------------
T_theileri_Tth.101.1090/1-119    PS--------LPGMELDND-------------------------
T_theileri_Tth.23.2140/1-546     AN--------IDGVELEEG-------------------------
T_theileri_Tth.10.2510/1-395     KKNE------LKGFYLENE-------------------------
T_theileri_Tth.107.1030/1-864    NS--------ADGMELQSMQMG----------------------
T_theileri_Tth.31.1060/1-887     SK---DDDVEIKGMPSENK-------------------------
T_theileri_Tth.132.1020/1-714    -----QD---YAGMILRYN-------------------------
T_theileri_Tth.136.1020/1-209    --------------------------------------------
T_theileri_Tth.129.1020/1-290    HLD-------ITGMYMEDE-------------------------
T_theileri_Tth.71.1080/1-526     SS--------LREMRLDHN-------------------------
T_theileri_Tth.36.2120/1-796     NS--------TKGMELSVIRRPHPAA------------------
T_theileri_Tth.63.1240/1-819     ----KGT---LKGLYLENE-------------------------
T_theileri_Tth.11.3250/1-309     --------------------------------------------
T_theileri_Tth.11.3240/1-592     PD--------LEGVELTLR-------------------------
T_theileri_Tth.12.1000/1-649     DK--------LTEVELEHK-------------------------
T_theileri_Tth.83.1080/1-228     --------------------------------------------
T_theileri_Tth.85.1030/1-137     --------------------------------------------
T_theileri_Tth.71.1030/1-204     --------------------------------------------
T_theileri_Tth.97.1060/1-928     ENNDNNK---VEGLYLENQ-------------------------
T_theileri_Tth.7.4630/1-775      NT--------TKGMLLEDQ-------------------------
T_theileri_Tth.24.1450/1-463     SN--------LKGMELEVY-------------------------
T_theileri_Tth.6.1080/1-569      EN--------LKGMELEEE-------------------------
T_theileri_Tth.14.1400/1-348     PN--------ATGVELEST-------------------------
T_theileri_Tth.21.1050/1-407     EVEL------VTGMYMEDE-------------------------
T_theileri_Tth.144.1030/1-653    NS--------LKEVELEHT-------------------------
T_theileri_Tth.269.1000/1-185    --------------------------------------------
T_theileri_Tth.144.1020/1-412    --------------------------------------------
T_theileri_Tth.151.1020/1-515    SN--------LKGMELGTK-------------------------
T_theileri_Tth.11.3450/1-561     SS--------AVGMELEDE-------------------------
T_theileri_Tth.166.1000/1-479    PNVEGDN--KIKGIALEND-------------------------
T_theileri_Tth.54.1350/1-777     NS--------AKGMEIQKG-------------------------
T_theileri_Tth.10.2520/1-446     --------------------------------------------
```

```
                                      750       760       770       780       790
T_theileri_Tth.43.1980/1-746   --------------------------------------------
T_theileri_Tth.32.2230/1-717   --------------------------------------------
T_theileri_Tth.11.3610/1-173   --------------------------------------------
T_theileri_Tth.46.1030/1-525   --------------------------------------------
T_theileri_Tth.36.1960/1-517   --------------------------------------------
T_theileri_Tth.165.1020/1-245  --------------------------------------------
T_theileri_Tth.46.1120/1-413   --------------------------------------------
T_theileri_Tth.24.2780/1-672   --------------------------------------------
T_theileri_Tth.8.4720/1-600    --------------------------------------------
T_theileri_Tth.165.1030/1-156  --------------------------------------------
T_theileri_Tth.6.5040/1-514    --------------------------------------------
T_theileri_Tth.31.1000/1-129   --------------------------------------------
T_theileri_Tth.11.3650/1-197   --------------------------------------------
T_theileri_Tth.19.2150/1-142   --------------------------------------------
T_theileri_Tth.4.4260/1-444    --------------------------------------------
T_theileri_Tth.70.1040/1-472   --------------------------------------------
T_theileri_Tth.46.1080/1-427   --------------------------------------------
T_theileri_Tth.251.1000/1-344  --------------------------------------------
T_theileri_Tth.27.1390/1-380   --------------------------------------------
T_theileri_Tth.12.1020/1-102   --------------------------------------------
T_theileri_Tth.12.1830/1-342   --------------------------------------------
T_theileri_Tth.11.3140/1-251   --------------------------------------------
T_theileri_Tth.286.1000/1-493  --------------------------------------------
T_theileri_Tth.61.1060/1-159   --------------------------------------------
T_theileri_Tth.10.2860/1-651   --------------------------------------------
T_theileri_Tth.11.2120/1-102   --------------------------------------------
T_theileri_Tth.132.1030/1-666  --------------------------------------------
T_theileri_Tth.101.1090/1-119  --------------------------------------------
T_theileri_Tth.23.2140/1-546   --------------------------------------------
T_theileri_Tth.10.2510/1-395   --------------------------------------------
T_theileri_Tth.107.1030/1-864  ----------------------------PLTRKNRPKPLERPQR
T_theileri_Tth.31.1060/1-887   --------------------------------------------
T_theileri_Tth.132.1020/1-714  --------------------------------------------
T_theileri_Tth.136.1020/1-209  --------------------------------------------
T_theileri_Tth.129.1020/1-290  --------------------------------------------
T_theileri_Tth.71.1080/1-526   --------------------------------------------
T_theileri_Tth.36.2120/1-796   --------------------------------------------
T_theileri_Tth.63.1240/1-819   --------------------------------------------
T_theileri_Tth.11.3250/1-309   --------------------------------------------
T_theileri_Tth.11.3240/1-592   --------------------------------------------
T_theileri_Tth.12.1000/1-649   --------------------------------------------
T_theileri_Tth.83.1080/1-228   --------------------------------------------
T_theileri_Tth.85.1030/1-137   --------------------------------------------
T_theileri_Tth.71.1030/1-204   --------------------------------------------
T_theileri_Tth.97.1060/1-928   --------------------------------------------
T_theileri_Tth.7.4630/1-775    --------------------------------------------
T_theileri_Tth.24.1450/1-463   --------------------------------------------
T_theileri_Tth.6.1080/1-569    --------------------------------------------
T_theileri_Tth.14.1400/1-348   --------------------------------------------
T_theileri_Tth.21.1050/1-407   --------------------------------------------
T_theileri_Tth.144.1030/1-653  --------------------------------------------
T_theileri_Tth.269.1000/1-185  --------------------------------------------
T_theileri_Tth.144.1020/1-412  --------------------------------------------
T_theileri_Tth.151.1020/1-515  --------------------------------------------
T_theileri_Tth.11.3450/1-561   --------------------------------------------
T_theileri_Tth.166.1000/1-479  --------------------------------------------
T_theileri_Tth.54.1350/1-777   ----------------------------LFFVRKKEISTTGV--
T_theileri_Tth.10.2520/1-446   --------------------------------------------
```

```
                                        800       810       820       830
T_theileri_Tth.43.1980/1-746    --------------------------------------------
T_theileri_Tth.32.2230/1-717    --------------------------------------------
T_theileri_Tth.11.3610/1-173    --------------------------------------------
T_theileri_Tth.46.1030/1-525    --------------------------------------------
T_theileri_Tth.36.1960/1-517    ------------------------------------------DF
T_theileri_Tth.165.1020/1-245   --------------------------------------------
T_theileri_Tth.46.1120/1-413    --------------------------------------------
T_theileri_Tth.24.2780/1-672    --------------------------------------------
T_theileri_Tth.8.4720/1-600     --------------------------------------------
T_theileri_Tth.165.1030/1-156   --------------------------------------------
T_theileri_Tth.6.5040/1-514     --------------------------------------------
T_theileri_Tth.31.1000/1-129    --------------------------------------------
T_theileri_Tth.11.3650/1-197    --------------------------------------------
T_theileri_Tth.19.2150/1-142    --------------------------------------------
T_theileri_Tth.4.4260/1-444     --------------------------------------------
T_theileri_Tth.70.1040/1-472    --------------------------------------------
T_theileri_Tth.46.1080/1-427    --------------------------------------------
T_theileri_Tth.251.1000/1-344   --------------------------------------------
T_theileri_Tth.27.1390/1-380    --------------------------------------------
T_theileri_Tth.12.1020/1-102    --------------------------------------------
T_theileri_Tth.12.1830/1-342    --------------------------------------------
T_theileri_Tth.11.3140/1-251    --------------------------------------------
T_theileri_Tth.286.1000/1-493   --------------------------------------------
T_theileri_Tth.61.1060/1-159    --------------------------------------------
T_theileri_Tth.10.2860/1-651    --------------------------------------------
T_theileri_Tth.11.2120/1-102    --------------------------------------------
T_theileri_Tth.132.1030/1-666   --------------------------------------------
T_theileri_Tth.101.1090/1-119   --------------------------------------------
T_theileri_Tth.23.2140/1-546    --------------------------------------------
T_theileri_Tth.10.2510/1-395    --------------------------------------------
T_theileri_Tth.107.1030/1-864   PGAPRPPRPEVSESRSTHRRTNLQEGVLGAPGASSPKRPVRSPP
T_theileri_Tth.31.1060/1-887    --------------------------------------------
T_theileri_Tth.132.1020/1-714   --------------------------------------------
T_theileri_Tth.136.1020/1-209   --------------------------------------------
T_theileri_Tth.129.1020/1-290   --------------------------------------------
T_theileri_Tth.71.1080/1-526    --------------------------------------------
T_theileri_Tth.36.2120/1-796    ------------NGARTNEQ------------GTGLQGPTATAI
T_theileri_Tth.63.1240/1-819    --------------------------------------------
T_theileri_Tth.11.3250/1-309    --------------------------------------------
T_theileri_Tth.11.3240/1-592    --------------------------------------------
T_theileri_Tth.12.1000/1-649    --------------------------------------------
T_theileri_Tth.83.1080/1-228    --------------------------------------------
T_theileri_Tth.85.1030/1-137    --------------------------------------------
T_theileri_Tth.71.1030/1-204    --------------------------------------------
T_theileri_Tth.97.1060/1-928    --------------------------------------------
T_theileri_Tth.7.4630/1-775     --------------------------------------------
T_theileri_Tth.24.1450/1-463    --------------------------------------------
T_theileri_Tth.6.1080/1-569     --------------------------------------------
T_theileri_Tth.14.1400/1-348    --------------------------------------------
T_theileri_Tth.21.1050/1-407    --------------------------------------------
T_theileri_Tth.144.1030/1-653   --------------------------------------------
T_theileri_Tth.269.1000/1-185   --------------------------------------------
T_theileri_Tth.144.1020/1-412   --------------------------------------------
T_theileri_Tth.151.1020/1-515   --------------------------------------------
T_theileri_Tth.11.3450/1-561    --------------------------------------------
T_theileri_Tth.166.1000/1-479   --------------------------------------------
T_theileri_Tth.54.1350/1-777    ----------------------------------------LATN
T_theileri_Tth.10.2520/1-446    --------------------------------------------
```

```
                              840       850       860       870
T_theileri_Tth.43.1980/1-746   --------------------------------------------
T_theileri_Tth.32.2230/1-717   --------------------------------------------
T_theileri_Tth.11.3610/1-173   --------------------------------------------
T_theileri_Tth.46.1030/1-525   --------------------------------------------
T_theileri_Tth.36.1960/1-517   SGSVRSIMSGLHTNRTLLR-------------------------
T_theileri_Tth.165.1020/1-245  --------------------------------------------
T_theileri_Tth.46.1120/1-413   --------------------------------------------
T_theileri_Tth.24.2780/1-672   --------------------------------------------
T_theileri_Tth.8.4720/1-600    --------------------------------------------
T_theileri_Tth.165.1030/1-156  --------------------------------------------
T_theileri_Tth.6.5040/1-514    --------------------------------------------
T_theileri_Tth.31.1000/1-129   --------------------------------------------
T_theileri_Tth.11.3650/1-197   --------------------------------------------
T_theileri_Tth.19.2150/1-142   --------------------------------------------
T_theileri_Tth.4.4260/1-444    --------------------------------------------
T_theileri_Tth.70.1040/1-472   --------------------------------------------
T_theileri_Tth.46.1080/1-427   --------------------------------------------
T_theileri_Tth.251.1000/1-344  --------------------------------------------
T_theileri_Tth.27.1390/1-380   --------------------------------------------
T_theileri_Tth.12.1020/1-102   --------------------------------------------
T_theileri_Tth.12.1830/1-342   --------------------------------------------
T_theileri_Tth.11.3140/1-251   --------------------------------------------
T_theileri_Tth.286.1000/1-493  --------------------------------------------
T_theileri_Tth.61.1060/1-159   --------------------------------------------
T_theileri_Tth.10.2860/1-651   --------------------------------------------
T_theileri_Tth.11.2120/1-102   --------------------------------------------
T_theileri_Tth.132.1030/1-666  --------------------------------------------
T_theileri_Tth.101.1090/1-119  --------------------------------------------
T_theileri_Tth.23.2140/1-546   --------------------------------------------
T_theileri_Tth.10.2510/1-395   --------------------------------------------
T_theileri_Tth.107.1030/1-864  HALTASVQEEYELGESQ---------------SNVMDYRLTGKE
T_theileri_Tth.31.1060/1-887   --------------------------------------------
T_theileri_Tth.132.1020/1-714  --------------------------------------------
T_theileri_Tth.136.1020/1-209  --------------------------------------------
T_theileri_Tth.129.1020/1-290  --------------------------------------------
T_theileri_Tth.71.1080/1-526   --------------------------------------------
T_theileri_Tth.36.2120/1-796   LALTASVQEEYELGESH---------------SNVIDGRLTGEE
T_theileri_Tth.63.1240/1-819   --------------------------------------------
T_theileri_Tth.11.3250/1-309   --------------------------------------------
T_theileri_Tth.11.3240/1-592   --------------------------------------------
T_theileri_Tth.12.1000/1-649   --------------------------------------------
T_theileri_Tth.83.1080/1-228   --------------------------------------------
T_theileri_Tth.85.1030/1-137   --------------------------------------------
T_theileri_Tth.71.1030/1-204   --------------------------------------------
T_theileri_Tth.97.1060/1-928   --------------------------------------------
T_theileri_Tth.7.4630/1-775    -----------TLSVTKVIDGQRDASHPRLPPNSLIAPHLGGPG
T_theileri_Tth.24.1450/1-463   --------------------------------------------
T_theileri_Tth.6.1080/1-569    --------------------------------------------
T_theileri_Tth.14.1400/1-348   --------------------------------------------
T_theileri_Tth.21.1050/1-407   --------------------------------------------
T_theileri_Tth.144.1030/1-653  --------------------------------------------
T_theileri_Tth.269.1000/1-185  --------------------------------------------
T_theileri_Tth.144.1020/1-412  --------------------------------------------
T_theileri_Tth.151.1020/1-515  --------------------------------------------
T_theileri_Tth.11.3450/1-561   --------------------------------------------
T_theileri_Tth.166.1000/1-479  --------------------------------------------
T_theileri_Tth.54.1350/1-777   TIVAQTVSLENPLRSSL---------------LSAVQAEYNDEE
T_theileri_Tth.10.2520/1-446   --------------------------------------------
```

```
                                        890       900       910       920
T_theileri_Tth.43.1980/1-746    --------------------------------------------
T_theileri_Tth.32.2230/1-717    --------------------------------------------
T_theileri_Tth.11.3610/1-173    --------------------------------------------
T_theileri_Tth.46.1030/1-525    --------------------------------------------
T_theileri_Tth.36.1960/1-517    --TEMMLSRKPVN-------------------------------
T_theileri_Tth.165.1020/1-245   --------------------------------------------
T_theileri_Tth.46.1120/1-413    --------------------------------------------
T_theileri_Tth.24.2780/1-672    --------------------------------------------
T_theileri_Tth.8.4720/1-600     --------------------------------------------
T_theileri_Tth.165.1030/1-156   --------------------------------------------
T_theileri_Tth.6.5040/1-514     --------------------------------------------
T_theileri_Tth.31.1000/1-129    --------------------------------------------
T_theileri_Tth.11.3650/1-197    --------------------------------------------
T_theileri_Tth.19.2150/1-142    --------------------------------------------
T_theileri_Tth.4.4260/1-444     --------------------------------------------
T_theileri_Tth.70.1040/1-472    --------------------------------------------
T_theileri_Tth.46.1080/1-427    --------------------------------------------
T_theileri_Tth.251.1000/1-344   --------------------------------------------
T_theileri_Tth.27.1390/1-380    --------------------------------------------
T_theileri_Tth.12.1020/1-102    --------------------------------------------
T_theileri_Tth.12.1830/1-342    --------------------------------------------
T_theileri_Tth.11.3140/1-251    --------------------------------------------
T_theileri_Tth.286.1000/1-493   --------------------------------------------
T_theileri_Tth.61.1060/1-159    --------------------------------------------
T_theileri_Tth.10.2860/1-651    --------------------------------------------
T_theileri_Tth.11.2120/1-102    --------------------------------------------
T_theileri_Tth.132.1030/1-666   --------------------------------------------
T_theileri_Tth.101.1090/1-119   --------------------------------------------
T_theileri_Tth.23.2140/1-546    --------------------------------------------
T_theileri_Tth.10.2510/1-395    --------------------------------------------
T_theileri_Tth.107.1030/1-864   SVGSVVQERRGEE---DFSSTKFTQNVHHSSSF--------FSK
T_theileri_Tth.31.1060/1-887    --------------------------------------------
T_theileri_Tth.132.1020/1-714   --------------------------------------------
T_theileri_Tth.136.1020/1-209   --------------------------------------------
T_theileri_Tth.129.1020/1-290   --------------------------------------------
T_theileri_Tth.71.1080/1-526    --------------------------------------------
T_theileri_Tth.36.2120/1-796    SVGSDVQERRGEE---GSSAAKFTQNVHHSSND--------FSK
T_theileri_Tth.63.1240/1-819    --------------------------------------------
T_theileri_Tth.11.3250/1-309    --------------------------------------------
T_theileri_Tth.11.3240/1-592    --------------------------------------------
T_theileri_Tth.12.1000/1-649    --------------------------------------------
T_theileri_Tth.83.1080/1-228    --------------------------------------------
T_theileri_Tth.85.1030/1-137    --------------------------------------------
T_theileri_Tth.71.1030/1-204    --------------------------------------------
T_theileri_Tth.97.1060/1-928    --------------------------------------------
T_theileri_Tth.7.4630/1-775     SPLHGSLISSSPLRDSLDDPLPRLLQPHEKPPTMEGGETLHTTQ
T_theileri_Tth.24.1450/1-463    --------------------------------------------
T_theileri_Tth.6.1080/1-569     --------------------------------------------
T_theileri_Tth.14.1400/1-348    --------------------------------------------
T_theileri_Tth.21.1050/1-407    --------------------------------------------
T_theileri_Tth.144.1030/1-653   --------------------------------------------
T_theileri_Tth.269.1000/1-185   --------------------------------------------
T_theileri_Tth.144.1020/1-412   --------------------------------------------
T_theileri_Tth.151.1020/1-515   --------------------------------------------
T_theileri_Tth.11.3450/1-561    --------------------------------------------
T_theileri_Tth.166.1000/1-479   --------------------------------------------
T_theileri_Tth.54.1350/1-777    HSLEGAQPHVGENHLRNADTHSVSEITSDSSDL--------RGN
T_theileri_Tth.10.2520/1-446    --------------------------------------------
```

```
                                   930       940       950       960
T_theileri_Tth.43.1980/1-746    ------------------------------------------AE
T_theileri_Tth.32.2230/1-717    --------------------------------------------
T_theileri_Tth.11.3610/1-173    --------------------------------------------
T_theileri_Tth.46.1030/1-525    ------------------------------------------PT
T_theileri_Tth.36.1960/1-517    -QKLNGIDVVPAEE-----------SSSSFQGPQVVEK-----Y
T_theileri_Tth.165.1020/1-245   --------------------------------------------
T_theileri_Tth.46.1120/1-413    --------------------------------------------
T_theileri_Tth.24.2780/1-672    --------------------------------------------
T_theileri_Tth.8.4720/1-600     -------------------------------------------G
T_theileri_Tth.165.1030/1-156   --------------------------------------------
T_theileri_Tth.6.5040/1-514     -------------------------------------------M
T_theileri_Tth.31.1000/1-129    --------------------------------------------
T_theileri_Tth.11.3650/1-197    --------------------------------------------
T_theileri_Tth.19.2150/1-142    --------------------------------------------
T_theileri_Tth.4.4260/1-444     --------------------------------------------
T_theileri_Tth.70.1040/1-472    --------------------------------------------
T_theileri_Tth.46.1080/1-427    --------------------------------------------
T_theileri_Tth.251.1000/1-344   -----------------------------------------GED
T_theileri_Tth.27.1390/1-380    --------------------------------------------
T_theileri_Tth.12.1020/1-102    --------------------------------------------
T_theileri_Tth.12.1830/1-342    --------------------------------------------
T_theileri_Tth.11.3140/1-251    --------------------------------------------
T_theileri_Tth.286.1000/1-493   --------------------------------------------
T_theileri_Tth.61.1060/1-159    --------------------------------------------
T_theileri_Tth.10.2860/1-651    -----------------------------------------GED
T_theileri_Tth.11.2120/1-102    --------------------------------------------
T_theileri_Tth.132.1030/1-666   -------------------------------------------G
T_theileri_Tth.101.1090/1-119   --------------------------------------------
T_theileri_Tth.23.2140/1-546    --------------------------------------------
T_theileri_Tth.10.2510/1-395    ------------------------------------------PN
T_theileri_Tth.107.1030/1-864   RRSLYLQATVAESPTPSECTPSKDGEVA-GGTKCIVET---LRF
T_theileri_Tth.31.1060/1-887    ------------------------------------------AS
T_theileri_Tth.132.1020/1-714   -------------------------------------------G
T_theileri_Tth.136.1020/1-209   --------------------------------------------
T_theileri_Tth.129.1020/1-290   --------------------------------------------
T_theileri_Tth.71.1080/1-526    --------------------------------------------
T_theileri_Tth.36.2120/1-796    RRSLHVQAAVAASETPSECTDDKKVETATGK-MCIVET---FQF
T_theileri_Tth.63.1240/1-819    -------------------------------------------D
T_theileri_Tth.11.3250/1-309    --------------------------------------------
T_theileri_Tth.11.3240/1-592    --------------------------------------------
T_theileri_Tth.12.1000/1-649    --------------------------------------------
T_theileri_Tth.83.1080/1-228    --------------------------------------------
T_theileri_Tth.85.1030/1-137    --------------------------------------------
T_theileri_Tth.71.1030/1-204    --------------------------------------------
T_theileri_Tth.97.1060/1-928    ------------------------------------------ED
T_theileri_Tth.7.4630/1-775     LGQSLNSRRDTTIQENTRNTVEPNDADIMNQLKKEGKIIGVERH
T_theileri_Tth.24.1450/1-463    --------------------------------------------
T_theileri_Tth.6.1080/1-569     --------------------------------------------
T_theileri_Tth.14.1400/1-348    --------------------------------------------
T_theileri_Tth.21.1050/1-407    --------------------------------------------
T_theileri_Tth.144.1030/1-653   --------------------------------------------
T_theileri_Tth.269.1000/1-185   --------------------------------------------
T_theileri_Tth.144.1020/1-412   --------------------------------------------
T_theileri_Tth.151.1020/1-515   --------------------------------------------
T_theileri_Tth.11.3450/1-561    --------------------------------------------
T_theileri_Tth.166.1000/1-479   ------------------------------------------AT
T_theileri_Tth.54.1350/1-777    RRSYLLKSATKASNIAGLTTSSSK---EVNERIDKVKK---RRL
T_theileri_Tth.10.2520/1-446    --------------------------------------------
```

```
                                          970       980       990       1000      1010
T_theileri_Tth.43.1980/1-746      SPT------PSHWERRIAKDE--LMSTYSNS---MGVTGMYYTN
T_theileri_Tth.32.2230/1-717      ----GDGRSKLHWERRIAKDE--LMSPYTEE-----PSGMFYTN
T_theileri_Tth.11.3610/1-173      --------------------------------------------
T_theileri_Tth.46.1030/1-525      EKN------PPHWKRLIAKDE--LMSPYNADPNVKYVTGAYYTA
T_theileri_Tth.36.1960/1-517      KLPLESMGYSSHWSRRIAKDE--LMVGLIGA--------GYYTA
T_theileri_Tth.165.1020/1-245     --------------------------------------------
T_theileri_Tth.46.1120/1-413      ----KGNQVIRHFDRRIAKDE--LMSPYSGS-----SNGMFYTA
T_theileri_Tth.24.2780/1-672      --NGEEECQGVHWAMRVAKGE--LMTVPREF------GAGYYTA
T_theileri_Tth.8.4720/1-600       DTY------SGYWERRIARDE--LMSPYTGE-----PTGMFYTN
T_theileri_Tth.165.1030/1-156     --------------------------------------------
T_theileri_Tth.6.5040/1-514       SRTALDYTTYPHWKRHYAKDE--LMGTYLDR-----PSGMYYTN
T_theileri_Tth.31.1000/1-129      --------------------------------------------
T_theileri_Tth.11.3650/1-197      --------------------------------------------
T_theileri_Tth.19.2150/1-142      --------------------------------------------
T_theileri_Tth.4.4260/1-444       -----GNSALSHFEMRNVRGE--LMSPKAPG-------GGSYSA
T_theileri_Tth.70.1040/1-472      --------------------------------------------
T_theileri_Tth.46.1080/1-427      -----HLQFSSHLERRLAKDD--LMSTYSEE-----PSGMYYTS
T_theileri_Tth.251.1000/1-344     HEK------PSHWERRIAKDE--LMSTYIGE-----ASGMYYSA
T_theileri_Tth.27.1390/1-380      ---GGSGTALSHWERRNAKDE--LMSGIAGA--------GYYTA
T_theileri_Tth.12.1020/1-102      --------------------------------------------
T_theileri_Tth.12.1830/1-342      -PPETGRNDDPHFDGRVARDE--LMAPLHGV----TGDKRSYSS
T_theileri_Tth.11.3140/1-251      --------------------------------------------
T_theileri_Tth.286.1000/1-493     NGIDNNGPNCTHWTQRYAKDE--LMSITKFS--VVYENIGYYTA
T_theileri_Tth.61.1060/1-159      --------------------------------------------
T_theileri_Tth.10.2860/1-651      QEK------PSHWERLIAKDE--LMSTYIGE-----ASGMYYSA
T_theileri_Tth.11.2120/1-102      --------------------------------------------
T_theileri_Tth.132.1030/1-666     STS------HPEWERRIAKDE--LMSQNTGE-----PTGMFYTA
T_theileri_Tth.101.1090/1-119     -DETDDNGIHPHWTRRVAHDE--LMAPREEE-----GVEIYYTA
T_theileri_Tth.23.2140/1-546      ------VSEISHWGRRNAKDE--LMGFRDDN------GIMLYTA
T_theileri_Tth.10.2510/1-395      ERA------PPHCERRIAKDE--LMSAYSDT---LGVTGMYYTK
T_theileri_Tth.107.1030/1-864     HSDHREPKPFSHWSRRNAKDE--LMVGLVGA--------GYYTA
T_theileri_Tth.31.1060/1-887      ETG------PLHWKRLIAKDE--LMSPYTPD--DKYVTGAYYTS
T_theileri_Tth.132.1020/1-714     STS------HSEWERRIAKDE--LMSPYTGE-----PTGMFYTA
T_theileri_Tth.136.1020/1-209     --------------------------------------------
T_theileri_Tth.129.1020/1-290     ----GDGRRKLHWERR----------------------------
T_theileri_Tth.71.1080/1-526      ----MAVGVSSHWDGYIAKDE--LMSPYTTG----EPSGMFYTI
T_theileri_Tth.36.2120/1-796      PPNGNEIITRSHWSRRNAKDE--LMVGVVGA--------GYYTA
T_theileri_Tth.63.1240/1-819      TLT------PSHWERRIAKDE--LMSTYSIS---MGVTGMYYTN
T_theileri_Tth.11.3250/1-309      --------------------------------------------
T_theileri_Tth.11.3240/1-592      --------DEAHWLQRVAKDE--LMAAPRES------GAGYYTA
T_theileri_Tth.12.1000/1-649      ----AKGVTTSHWNLRNTKDE--LMSMHTST--GAIEGIGYYTA
T_theileri_Tth.83.1080/1-228      --------------------------------------------
T_theileri_Tth.85.1030/1-137      --------------------------------------------
T_theileri_Tth.71.1030/1-204      --------------------------------------------
T_theileri_Tth.97.1060/1-928      HEN------SSHWERLIAKDE--LMSTYIGE-----TSGMYYTN
T_theileri_Tth.7.4630/1-775       HYVDNLFKHRSHWKRHNAKDE--LMAGVVGA--------GYYTA
T_theileri_Tth.24.1450/1-463      -----SYGTQYHWARRIAKDE--LMALSYFN------NGGYYTA
T_theileri_Tth.6.1080/1-569       --EDSHNEGSSHWERRNAKDE--LMSPIRGG-----NSAMYYTA
T_theileri_Tth.14.1400/1-348      ---SSCGTARSHLEFRNAKDE--MMAPSSKA--------SYYTK
T_theileri_Tth.21.1050/1-407      ----GNGQYTSHWERRIAKDE--LMSPYTGE-----PSGMYYTA
T_theileri_Tth.144.1030/1-653     ----AEGNTTSHWNRRNMKDE--LMSMVSAA--GNIESVGYYTA
T_theileri_Tth.269.1000/1-185     --------------------------------------------
T_theileri_Tth.144.1020/1-412     --------------------------------------------
T_theileri_Tth.151.1020/1-515     -----GDDTRVYWLRHIARDE--LMTPMSVY----NYGAGYYTA
T_theileri_Tth.11.3450/1-561      ---GDNNTAGSHWKRRNARDE--LMASMGST-------GMYYTA
T_theileri_Tth.166.1000/1-479     DGA------PLHWERRIAKDE--LMSTYSGS-----SNGMFYTA
T_theileri_Tth.54.1350/1-777      PPRYVITLASSHWSRRNAKDE--LMVGLVGA--------GYYTA
T_theileri_Tth.10.2520/1-446      --------------------------------------------
```

```
                                         1020      1030      1040      1050
                                            |         |         |         |
T_theileri_Tth.43.1980/1-746    LTLAAFHSMPFYSANFSMAEPMSWGKQYICDLFKG-IKDLQL--
T_theileri_Tth.32.2230/1-717    LTLAAFHSLPFYSANFSMAEPMSWGNKSGCDLLHKTCTKDKNEL
T_theileri_Tth.11.3610/1-173    --------------------------------------------
T_theileri_Tth.46.1030/1-525    LTLAVFHSMPFYKADFDKAESMSWGKNAGCDFLKGEDKKKEEVI
T_theileri_Tth.36.1960/1-517    ITMGAFADLGYYKVEWAMAEQMSWGNNSGCGLLEKKCVEGGR--
T_theileri_Tth.165.1020/1-245   --------------------------------------------
T_theileri_Tth.46.1120/1-413    LTLAVFESTGHYKANFAKAENMSWGRNAGCVFLKKKCRDDSLS-
T_theileri_Tth.24.2780/1-672    LTMALFEDLQFYKANWGMEEQMSWGNQSGCTFLKEECA--KKHN
T_theileri_Tth.8.4720/1-600     LTLAAFGDMPFYEVNFSMAEPMSWGNQSTCDFIQS-TCTAIS-K
T_theileri_Tth.165.1030/1-156   --------------------------------------------
T_theileri_Tth.6.5040/1-514     LTLAVFDSMPFYEADFSRAETMSWGRNAGCGFLHGKCIEDDYP-
T_theileri_Tth.31.1000/1-129    --------------------------------------------
T_theileri_Tth.11.3650/1-197    --------------------------------------------
T_theileri_Tth.19.2150/1-142    --------------------------------------------
T_theileri_Tth.4.4260/1-444     LTLAVFDDMPFYKANFSRAEPLRWANNSGCDFLEKKCIENKTS-
T_theileri_Tth.70.1040/1-472    --------------------------------------------
T_theileri_Tth.46.1080/1-427    LTLAIFNDMKFYKANFSMAETMSWGSNAGCEFLTEKCIQENII-
T_theileri_Tth.251.1000/1-344   LTLAAFHSMPFYSAN---------SLVRMCYLIV----------
T_theileri_Tth.27.1390/1-380    LTMAVMEDLGFYKAVWGMEEPMSWGRMSGCKLLTDKCV--ENGT
T_theileri_Tth.12.1020/1-102    -NLQGIKDYTYY--------------------------------
T_theileri_Tth.12.1830/1-342    LTLAAFESTGYYX-------------------------------
T_theileri_Tth.11.3140/1-251    --MAAFDDMKYYSANWGMEEPMSWGNKSGCDFIQKGCL-TENGV
T_theileri_Tth.286.1000/1-493   LTIAAFEDMGFYKGKFEMAENMSWGRGAGCELIKGTYKEELEK-
T_theileri_Tth.61.1060/1-159    --------------------------------------------
T_theileri_Tth.10.2860/1-651    LTLAAFHSMGFYKAVFEKAEPMGWGKVSMCKLLKA-EKNLTVF-
T_theileri_Tth.11.2120/1-102    LTMGCLS-------------------------------------
T_theileri_Tth.132.1030/1-666   LTLAVFDAMDFYQADFDMAEPMSWGKGFGCELFTS-TCKDIIKK
T_theileri_Tth.101.1090/1-119   LTMPFRTWVAYK--------------------------------
T_theileri_Tth.23.2140/1-546    LTMATFEDMGVFKANWGMEETMRWGRNAGCEFLQQKCM--VDNI
T_theileri_Tth.10.2510/1-395    LTLAVFHSMPFYSANFNMAEPMSWGKDLNVSYLRVKRIQQRL--
T_theileri_Tth.107.1030/1-864   ITMGAFADLGYYKVNWTMAEQMSWGNNSKCAFLNNKCVNSGE--
T_theileri_Tth.31.1060/1-887    LTLAVFHSMPFYSANFSMAESMSWGKNAGCEFLKGKDKETYKGT
T_theileri_Tth.132.1020/1-714   LTLAVFDAMDFYQADFDMAEPMSWGKGFGCELFTS-TCKDIIEK
T_theileri_Tth.136.1020/1-209   --------------------------------------------
T_theileri_Tth.129.1020/1-290   --------------------------------------------
T_theileri_Tth.71.1080/1-526    LTLSLFEDMKFYRANYIMAETMRWGNHSGCGFLSGDCVEKDEG-
T_theileri_Tth.36.2120/1-796    ITMGVFADLGYYKVNWKRAEQMSWGNNSGCELLEDKCVKEGR--
T_theileri_Tth.63.1240/1-819    LTLAAFHSMPFYSANFTMAEPMSWGKGSECDLLEG-KKDPAK--
T_theileri_Tth.11.3250/1-309    --MAAFDDMKYYSANWGMEEPMSWGNKSGCDFIQKGCL-TEIGV
T_theileri_Tth.11.3240/1-592    LTMSTFEGLGYYRANWGMEEPMSWGHKGGCNFLKETCT--ENEK
T_theileri_Tth.12.1000/1-649    LTIASFEDLGFYKGNFNMSEPMSWGYHAGCEFLKATCKEKDKP-
T_theileri_Tth.83.1080/1-228    --------------------------------------------
T_theileri_Tth.85.1030/1-137    --------------------------------------------
T_theileri_Tth.71.1030/1-204    --------------------------------------------
T_theileri_Tth.97.1060/1-928    LTLAAFEDMKFYKAVFEKAEPMSWGNQSGCEFLQG-KKDTTKT-
T_theileri_Tth.7.4630/1-775     LTMAFFADMGYYKVEWSKAEPMSWGNQSGCKLLDDKCVNNGKT-
T_theileri_Tth.24.1450/1-463    LTMALFEDLQYYKANWGMEEQMSWGNQSGCDFLQERCM-EKNNK
T_theileri_Tth.6.1080/1-569     LTLAAFADMKFYKANFTMAETMTWGKDAECDFLEEKCM--ENGV
T_theileri_Tth.14.1400/1-348    LTLGIFESMPFYKVNYSMAEPMKWGNNSGCGFLEKKCL------
T_theileri_Tth.21.1050/1-407    LTLAAFHSMPFYKANFNMAESMSWGKKSGCDLLHKTCTRSKYEL
T_theileri_Tth.144.1030/1-653   LTIAAFHDMQYYRGNFSMAEPMSWGYMGGCNFGNVTIENTKRV-
T_theileri_Tth.269.1000/1-185   --------------------------------------------
T_theileri_Tth.144.1020/1-412   --------------------------------------------
T_theileri_Tth.151.1020/1-515   LTMALFEDLQYYKANWGMEEQMSWGNQSGCDFLHKWCI--VENK
T_theileri_Tth.11.3450/1-561    MTMATFEDMKFYKANWGKEERMSWGENAGCAFLEEKCI--VNNI
T_theileri_Tth.166.1000/1-479   LTLAVFDSMPFYKAEFKMAESMNWGKNAGCEFLK--DKEKYKET
T_theileri_Tth.54.1350/1-777    LTLGAFADLGYYKVNWTMAEQMSWGNNSGCEFLDNKCVNSGE--
T_theileri_Tth.10.2520/1-446    --------------------------------------------
```

```
                               1060      1070      1080      1090
T_theileri_Tth.43.1980/1-746   ----T------NYPDMFCKDDT------KVTLQCTSDRFALGIC
T_theileri_Tth.32.2230/1-717   M----------KYTSMFCDENG-------PVLQCTSDRFALGTC
T_theileri_Tth.11.3610/1-173   --------------------------------------------
T_theileri_Tth.46.1030/1-525   K----------KNTEMFCSEIN-------LTPQCTSDRFALGRC
T_theileri_Tth.36.1960/1-517   ----T------KFPDMFCTSKSTE---GPAGLQCTSDRQSLGRC
T_theileri_Tth.165.1020/1-245  --------------------------------QCTSDRFGLGIC
T_theileri_Tth.46.1120/1-413   -----------NYGDMFCDIDS------AGPLQCTSDRFALGKC
T_theileri_Tth.24.2780/1-672   E----------VINGLFDG---------QSVSRCTSDRTAYGKF
T_theileri_Tth.8.4720/1-600    T---L------KSSNVFCQES------DKPVLQCTSDRFGLGVC
T_theileri_Tth.165.1030/1-156  -----------EYPNMFCTESK-------TALQCTSDRFALGKC
T_theileri_Tth.6.5040/1-514    -----------KFPDMFCSKIS-------DDLHCTTDRTALGKC
T_theileri_Tth.31.1000/1-129   --------------------------------------------
T_theileri_Tth.11.3650/1-197   --------------------------------------------
T_theileri_Tth.19.2150/1-142   --------------------------------------------
T_theileri_Tth.4.4260/1-444    -----------NFPDIFCT----ATVRIKGYLQCTYDRMALGQC
T_theileri_Tth.70.1040/1-472   -----------KYSPVYLQ-------------------------
T_theileri_Tth.46.1080/1-427   -----------KYPNTFCT-------ESRVSLQCTSDRLSLGRC
T_theileri_Tth.251.1000/1-344  --------------------------------------------
T_theileri_Tth.27.1390/1-380   T----------DYPDMFCT-------TDSNTLRCTSDRRALGTC
T_theileri_Tth.12.1020/1-102   --------------------------------------------
T_theileri_Tth.12.1830/1-342   --------------------------------------------
T_theileri_Tth.11.3140/1-251   S----------NYPNAFCT--------GEEKIRCSTDHFGFAFC
T_theileri_Tth.286.1000/1-493  -----------KYPNMFCK--------YEGKSLCASNRMGMTC
T_theileri_Tth.61.1060/1-159   --------------------------------------------
T_theileri_Tth.10.2860/1-651   -----------ARSDLFCKEED------KVLLQCTSDRFALGIC
T_theileri_Tth.11.2120/1-102   --------------------------------------------
T_theileri_Tth.132.1030/1-666  D--LG------KFPNILCEESK-----GESTLQCTSDRFGLGVC
T_theileri_Tth.101.1090/1-119  --------------------------------------------
T_theileri_Tth.23.2140/1-546   T----------KYPHMFCN--------TTNPLRCTSDRRALGVC
T_theileri_Tth.10.2510/1-395   -----------TIPLCFAK-------------------------
T_theileri_Tth.107.1030/1-864  ----T------KFSNMFCTTKSTD---GPAGLQCTSDRQSLGRC
T_theileri_Tth.31.1060/1-887   K--SN------KYSEMFCDKVE-------PALQCTSDRFALGMC
T_theileri_Tth.132.1020/1-714  D--LG------NLSKTFCKESD-----GEHSLQCTSDRFGLGVC
T_theileri_Tth.136.1020/1-209  --------------------------------------------
T_theileri_Tth.129.1020/1-290  --------------------------------------------
T_theileri_Tth.71.1080/1-526   -----------KHSDVFCDSSS--VATRTTTLQCTSDRFALGHC
T_theileri_Tth.36.2120/1-796   ----T------KFPDMFCTTEPAS---LPAGLQCTSDRQSLGSC
T_theileri_Tth.63.1240/1-819   ----T------KYSTLFCKDD------DAKTLRCTSDRFALGMC
T_theileri_Tth.11.3250/1-309   S----------NYPDAFCT--------GEQRLRCSTGHFGLAFC
T_theileri_Tth.11.3240/1-592   -----------HYSEQFCN--------DTTALRCTSDRTAYGMW
T_theileri_Tth.12.1000/1-649   -----------VADGKSCS--------PYKAGSCTSDRLGVAKC
T_theileri_Tth.83.1080/1-228   --------------------------------------------
T_theileri_Tth.85.1030/1-137   --------------------------------------------
T_theileri_Tth.71.1030/1-204   --------------------------------------------
T_theileri_Tth.97.1060/1-928   -----------EYSTLFCKDDD-----AKTILKCTSDRFALGIC
T_theileri_Tth.7.4630/1-775    -----------EYTEMFCT----TEHTDKTLLQCTSDRHALGTC
T_theileri_Tth.24.1450/1-463   V----------RYPEYFCN---------ETIFRCTSDRIAYSSC
T_theileri_Tth.6.1080/1-569    T----------KYPGMFCS----DTSSITSQTHCTSDRRGVGYC
T_theileri_Tth.14.1400/1-348   --------------------------------------------
T_theileri_Tth.21.1050/1-407   M----------KYTSMFCDENE-------PVLQCTSDRFALGMC
T_theileri_Tth.144.1030/1-653  -----------LAPNGYCI--------EALEKSCSSDRLGVINS
T_theileri_Tth.269.1000/1-185  --------------------------------------------
T_theileri_Tth.144.1020/1-412  --------------------------------------------
T_theileri_Tth.151.1020/1-515  V----------RYPEYFCN---------ETIFRCRSDRLAYAPC
T_theileri_Tth.11.3450/1-561   T----------AFPHMFCN------ESKSSTLRCTFDRRALGSC
T_theileri_Tth.166.1000/1-479  K--SG------KYSEMFCDEVK-------PTLQCTSDRFALGMC
T_theileri_Tth.54.1350/1-777   ----S------NFSNMFCTTERAA---TPESLQCTSDRQSLGSC
T_theileri_Tth.10.2520/1-446   --------------------------------------------
```

```
                                                    1110      1120      1130      1140
T_theileri_Tth.43.1980/1-746    STKDNR----N-KLSGRY-QYFKD--EDTTKDAN-DLMDGVPFI
T_theileri_Tth.32.2230/1-717    SSKSLPG-----TLPGEY-HYFTK-----TAGQN-EMTNGCPIV
T_theileri_Tth.11.3610/1-173    --------------------------------------------
T_theileri_Tth.46.1030/1-525    SMKMSGEFED--KLPLYYQILFTS--INSDPEN--ELTDGYTII
T_theileri_Tth.36.1960/1-517    S---IQTH-RN-PLPDHF-QYFSD--GKLGGNKA-DLMDYCPII
T_theileri_Tth.165.1020/1-245   AKTNFSREIHGE-------KYLTA--ADLGENSS-WKRINWPVV
T_theileri_Tth.46.1120/1-413    SQTWKSD-----ALSWQKCM------SRYAENEMAELDDVCPII
T_theileri_Tth.24.2780/1-672    LLNESLFK---------------------------EYQSTCNVK
T_theileri_Tth.8.4720/1-600     SKVDTN---TH-SMSGEY-KLFEI--V--G-EQS-DMMKGCPII
T_theileri_Tth.165.1030/1-156   TVRTFDQ-----SLPDRY-RFFEK--ANVGAPSS-ELMNHCPAI
T_theileri_Tth.6.5040/1-514     SLEDLYWD----IIPREF-RHFTN--GDLGSPKE-DVMDYCPVI
T_theileri_Tth.31.1000/1-129    --------------------------------------------
T_theileri_Tth.11.3650/1-197    --------------------------------------------
T_theileri_Tth.19.2150/1-142    --------------------------------------------
T_theileri_Tth.4.4260/1-444     LRKTYPE-----VIEPHF-QYLWN--AHLGGTE--FWMDYCPYV
T_theileri_Tth.70.1040/1-472    -------------------FSPET--DTTDSKN--DLTDGYPII
T_theileri_Tth.46.1080/1-427    TSSNFPQ-----DYPEDY-RNFKN--NTFTYLYG-ELTDGCPII
T_theileri_Tth.251.1000/1-344   --------------------------------------------
T_theileri_Tth.27.1390/1-380    TIGTYTF-----PLPTEY-QYFTD--PTAGARTG-NLMDYCPYI
T_theileri_Tth.12.1020/1-102    --------------------------------------------
T_theileri_Tth.12.1830/1-342    --------------------------------------------
T_theileri_Tth.11.3140/1-251    ANESDIGRENA------------------------DSTGSCPVL
T_theileri_Tth.286.1000/1-493   SERESNNGDINFVTQNEF--------------------------
T_theileri_Tth.61.1060/1-159    --------------------------------------------
T_theileri_Tth.10.2860/1-651    SNETGI----N-DSSEGY-KYIKD----GTHIAK-DLMNGVPFI
T_theileri_Tth.11.2120/1-102    --------------------------------------------
T_theileri_Tth.132.1030/1-666   SKDDNT----H-GMPDGY-KFFEA--V--G-EQS-EMMKECPII
T_theileri_Tth.101.1090/1-119   --------------------------------------------
T_theileri_Tth.23.2140/1-546    NIVSNLV-----SLPEQF-QYFTS--NTVGGPSS-DAMDHCPIV
T_theileri_Tth.10.2510/1-395    ------------------------------------MMTRIPYS
T_theileri_Tth.107.1030/1-864   -SIEAKPA-EG-ELPGHF-QYFSDK-KKIGS-QA-FQMDYCPFI
T_theileri_Tth.31.1060/1-887    SMETSGV-----EVHYEYGGVFVS-VLTKRREN--DLMDGYSII
T_theileri_Tth.132.1020/1-714   SEDDNT----H-GMPDGY-KFFEA--V--G-EQS-EMMKECPII
T_theileri_Tth.136.1020/1-209   --------------------------------------------
T_theileri_Tth.129.1020/1-290   --------------------------------------------
T_theileri_Tth.71.1080/1-526    TRSLSSQP------------------ISVFSAEHREEKRECAVV
T_theileri_Tth.36.2120/1-796    -SITTKTD-GN-DVPEEF-RYFSEQ-NKVGS-EA-AQMDYCPFI
T_theileri_Tth.63.1240/1-819    LTKNNL----K-DLPNGY-EYFKD--EDGGLETN-DLMDSYPII
T_theileri_Tth.11.3250/1-309    FNESDPHYYNR------------------------DSRGSCPVL
T_theileri_Tth.11.3240/1-592    YRGQQREYQ--------------------------GPNDVCHVI
T_theileri_Tth.12.1000/1-649    VSSNRK-----------------------------NGNPICPIL
T_theileri_Tth.83.1080/1-228    --------------------------------------------
T_theileri_Tth.85.1030/1-137    --------------------------------------------
T_theileri_Tth.71.1030/1-204    --------------------------------------------
T_theileri_Tth.97.1060/1-928    STKDNRN-----NLPEGY-QYVKD--RSTGYTAK-DLMDGHTFI
T_theileri_Tth.7.4630/1-775     LLQELKHTPYYRGTTSPY-LYFEK--GHWGSPEG-ELMDYCPFI
T_theileri_Tth.24.1450/1-463    HVPSSMYL---------------------------ENKDICRVT
T_theileri_Tth.6.1080/1-569     AISNSHY-----NLPNHF-QYFWS--PNTGGEVS-SLTDYCPVI
T_theileri_Tth.14.1400/1-348    --------------------------------------------
T_theileri_Tth.21.1050/1-407    SSISVLD-----TLPEVY-SYFTK-----TAGQN-EMTNGCPI-
T_theileri_Tth.144.1030/1-653   VAPTTK-----------------------------EVESICPIL
T_theileri_Tth.269.1000/1-185   --------------------------------------------
T_theileri_Tth.144.1020/1-412   --------------------------------------------
T_theileri_Tth.151.1020/1-515   SVPASMIV---------------------------VPEDICHTS
T_theileri_Tth.11.3450/1-561    SLTVHNI-----SIPEVY-QYFTN--PYLGGNKD-DLMDYCPFI
T_theileri_Tth.166.1000/1-479   TKKQPDV-----KIPDEYDTYFPS--FLYDPLD--DLMDGYTII
T_theileri_Tth.54.1350/1-777    SIIGAKKE----NLSGEF-QYFSDT-TRIGSEES-EQMDYCPFI
T_theileri_Tth.10.2520/1-446    -------------------------------------MDNYPII
```

```
                                   1150      1160      1170      1180
T_theileri_Tth.43.1980/1-746    RPL-----N---------GTAC----EGGEE-EL--MPGSIVS-
T_theileri_Tth.32.2230/1-717    KPL-----K---------ETTC----ESGNV-EL--MPGSIVS-
T_theileri_Tth.11.3610/1-173    --------------------------------------------
T_theileri_Tth.46.1030/1-525    KPF-----F---------STSC----ENGEV-EY--MPGSLRG-
T_theileri_Tth.36.1960/1-517    VGK-----R---------ETSC----TDGEK-SK--MPGSVIA-
T_theileri_Tth.165.1020/1-245   QTS-----P---------TTAC----EGGNG-KM--MPGSRLG-
T_theileri_Tth.46.1120/1-413    K-------------------------------------------
T_theileri_Tth.24.2780/1-672    ED------YIAREMPQN-SSYC----ADTTT-DTSAPPGSIMG-
T_theileri_Tth.8.4720/1-600     RPF-----Q---------ETMC----ENGKE-TL--MPGSIVD-
T_theileri_Tth.165.1030/1-156   KPL-----A---------STSC----EDGDS-TQ--MPGSLLG-
T_theileri_Tth.6.5040/1-514     EPT-----F---------KTSC----ELGDE-NE--MPGSILS-
T_theileri_Tth.31.1000/1-129    --------------------------------------------
T_theileri_Tth.11.3650/1-197    --------------------------------------------
T_theileri_Tth.19.2150/1-142    --------------------------------------------
T_theileri_Tth.4.4260/1-444     EKVR--------------YGDC----TDGRR-ST--IIGSFVG-
T_theileri_Tth.70.1040/1-472    KPI-----H---------MTSC----ENGNV-EY--MPGSVVG-
T_theileri_Tth.46.1080/1-427    RQL-----E---------KTTC----QSGDL-EL--MPGSILG-
T_theileri_Tth.251.1000/1-344   --------------------------------------------
T_theileri_Tth.27.1390/1-380    AEY-----N---------NTGC----TNGNI-NV--MPGSRVS-
T_theileri_Tth.12.1020/1-102    --------------------------------------------
T_theileri_Tth.12.1830/1-342    --------------------------------------------
T_theileri_Tth.11.3140/1-251    VSPL----SVRSDGSLR-SRAC----PDLGD-AD--FPGFLTG-
T_theileri_Tth.286.1000/1-493   -------------------ALC----TSKEL-----TPGYGVGR
T_theileri_Tth.61.1060/1-159    --------------------------------------------
T_theileri_Tth.10.2860/1-651    RSL-----K---------GTAC----EGGEE-SL--MPGSILS-
T_theileri_Tth.11.2120/1-102    --------------------------------------------
T_theileri_Tth.132.1030/1-666   KPF-----Q---------QTMC----ETGDE-TL--MPGSIVS-
T_theileri_Tth.101.1090/1-119   --------------------------------------------
T_theileri_Tth.23.2140/1-546    SS----------------NLIC----SKENT-GD--TSASLLG-
T_theileri_Tth.10.2510/1-395    ALL-----T-----------------------------------
T_theileri_Tth.107.1030/1-864   TAK-----E---------GYSC----INGEE-SN--LPGSVIA-
T_theileri_Tth.31.1060/1-887    KPI-----P---------ETSC----EEDEL-DL--MPGSLVG-
T_theileri_Tth.132.1020/1-714   KPF-----Q---------QTMC----ETGDE-TL--MPGSIVS-
T_theileri_Tth.136.1020/1-209   --------------------------------------------
T_theileri_Tth.129.1020/1-290   --------------------------------------------
T_theileri_Tth.71.1080/1-526    KATA--------------ATVC----ENGSE-AH--MPGSRIG-
T_theileri_Tth.36.2120/1-796    MAK-----K---------GYSC----INGEESNN--MPGSVIA-
T_theileri_Tth.63.1240/1-819    KPL-----I---------GTTC----EGGKE-TL--IPGSVLG-
T_theileri_Tth.11.3250/1-309    IQPL----YV--ENGFH-SFAC----TDLGN-AN--FPGFLTD-
T_theileri_Tth.11.3240/1-592    GSF------VKGNDNVKTYTYC----TGAET-NA--LPGSLMG-
T_theileri_Tth.12.1000/1-649    KLN-----D---------SFYC----TATRW-GIPNYWPGLHG-
T_theileri_Tth.83.1080/1-228    --------------------------------------------
T_theileri_Tth.85.1030/1-137    --------------------------------------------
T_theileri_Tth.71.1030/1-204    --------------------------------------------
T_theileri_Tth.97.1060/1-928    RPL-----E---------GTAC----EGGKE-DL--LPGSIRS-
T_theileri_Tth.7.4630/1-775     TAN-----N---------DNWC----TDGIA-DK--MPGSVIA-
T_theileri_Tth.24.1450/1-463    ENYGSV--DVDNKTVYLPLALC----TDNKT-DTVKLPGSIMG-
T_theileri_Tth.6.1080/1-569     EPF-----P---------RGMC----SDPDI-IY--APGSRRG-
T_theileri_Tth.14.1400/1-348    --------------------------------------------
T_theileri_Tth.21.1050/1-407    --------------------------------------------
T_theileri_Tth.144.1030/1-653   VAD-----V---------SFFC----TSEG--DVRDRDVGLFR-
T_theileri_Tth.269.1000/1-185   --------------------------------------------
T_theileri_Tth.144.1020/1-412   --------------------------------------------
T_theileri_Tth.151.1020/1-515   GDYP----YVDRSGNHLTTYYC----DDNTT-DE--VAGSIMG-
T_theileri_Tth.11.3450/1-561    VPY-----E---------DSYC----NNGEK-EL--LPGSRIS-
T_theileri_Tth.166.1000/1-479   KPI-----P---------KTNC----EEDKF-NL--MPGSILS-
T_theileri_Tth.54.1350/1-777    LAK-----P---------GYSC----INGEQRTK--MPGSVIA-
T_theileri_Tth.10.2520/1-446    KPL-----I---------GTAC----EGGEE-SL--MPGSLVS-
```

```
                                   1190      1200      1210      1220      1230
T_theileri_Tth.43.1980/1-746    SMSRCLNLETP-ILKD-----N--KDDVK----VQAVCANVKCD
T_theileri_Tth.32.2230/1-717    KMSRCLNVKEP-EFSEGDQ-----RNGVT----VKGICAKVKCE
T_theileri_Tth.11.3610/1-173    --------------------------------------------
T_theileri_Tth.46.1030/1-525    KESRCLKGMDL-TLNN----RN--KRDLP----VGDICANVKCD
T_theileri_Tth.36.1960/1-517    ANSRCVQGENL-KAK-----------NAA----VGAVCVEVSCK
T_theileri_Tth.165.1020/1-245   PQSRCLTTESL-QVNDGT------SSDKS----VQGICANVKCD
T_theileri_Tth.46.1120/1-413    --------------------------------------------
T_theileri_Tth.24.2780/1-672    PDSWCLDAEL--TVKD--------AESTF--SNVKGVCAQVSCD
T_theileri_Tth.8.4720/1-600     KMSRCLDLKET-VEID----EN--GKEVK----VQGICAKVKCD
T_theileri_Tth.165.1030/1-156   PQSRCLKGESL-KVKDGI------SSYKS----VQGICANVKCD
T_theileri_Tth.6.5040/1-514     STSRCLDVNFV-RLRNEDVNDK-----------VKGICAEVKCE
T_theileri_Tth.31.1000/1-129    --------------------------------------------
T_theileri_Tth.11.3650/1-197    --------------------------------------------
T_theileri_Tth.19.2150/1-142    --------------------------------------------
T_theileri_Tth.4.4260/1-444     PDARCVKGNEL-KYRTTP---------------IGDVCVNTKCE
T_theileri_Tth.70.1040/1-472    NMSRCLKGKDL-ELKE----EN--EYGLT----VRGICAYVNCE
T_theileri_Tth.46.1080/1-427    KGSRCLQGDGL-IQKKSA------QTFLP----VGDICANVKCE
T_theileri_Tth.251.1000/1-344   --------------------------------------------
T_theileri_Tth.27.1390/1-380    SSSRCVKGDSL-HIATFP---------------IGDVCAEISCD
T_theileri_Tth.12.1020/1-102    --------------------------------------------
T_theileri_Tth.12.1830/1-342    --------------------------------------------
T_theileri_Tth.11.3140/1-251    ENSMCLTTEDY-EIVKN-------DDAIK----LSGVCAEVLCD
T_theileri_Tth.286.1000/1-493   PDSWCLDTDDL-VRRNNDENST-----------SYGVCGEVRCD
T_theileri_Tth.61.1060/1-159    --------------------------------------------
T_theileri_Tth.10.2860/1-651    NMSRCLNVEKP-VNFSE--GDQ--KNGVK----VYGICAKVKCE
T_theileri_Tth.11.2120/1-102    --------------------------------------------
T_theileri_Tth.132.1030/1-666   NMSRCLDVTEP-VEID---GNN--GTGLK----VQGICAKVECD
T_theileri_Tth.101.1090/1-119   --------------------------------------------
T_theileri_Tth.23.2140/1-546    RDSWCLDTDSL-IVKYN-------DADVE----MGGLCVVVKCD
T_theileri_Tth.10.2510/1-395    -------------------------------------VLPLVCA
T_theileri_Tth.107.1030/1-864   NNSRCVEGKDL-KANN----------NAA----VGAVCVEVSCK
T_theileri_Tth.31.1060/1-887    KESRCLKGEGL-KLQI----PN--KKNLP----VGDICAKVKCE
T_theileri_Tth.132.1020/1-714   NMSRCLDVEEP-VEID----EN--GKKVK----VQGICAKVKCD
T_theileri_Tth.136.1020/1-209   --------------------------------------------
T_theileri_Tth.129.1020/1-290   --------------------------------------------
T_theileri_Tth.71.1080/1-526    NISRCLKGDAL-RLRD--------VSTIDIHSIIGDICANVKCE
T_theileri_Tth.36.2120/1-796    NNSRCVEGKDL-KAN-----------NAA----IGAVCVEVSCK
T_theileri_Tth.63.1240/1-819    TESRCLDVKNP-L--------D--KNGVK----IQGICAKVKCN
T_theileri_Tth.11.3250/1-309    GSSMCLTTEDY-EIERE-------KGIEK----MNGVCAQVLCD
T_theileri_Tth.11.3240/1-592    PDSWCLDGEGL-QVQNA----------GESVESLSGVCARVSCD
T_theileri_Tth.12.1000/1-649    DNSWCLDADDL-TFNSDKGESVT----------INGMCTAVDCS
T_theileri_Tth.83.1080/1-228    --------------------------------------------
T_theileri_Tth.85.1030/1-137    --------------------------------------------
T_theileri_Tth.71.1030/1-204    --------------------------------------------
T_theileri_Tth.97.1060/1-928    SMSRCVDLNAP-LEPKNS------RNGVK----FQGICAKVKCN
T_theileri_Tth.7.4630/1-775     PNSRCVKGVGL-KVNEKN---------------IGDVCVNVSCK
T_theileri_Tth.24.1450/1-463    PDSWCLDAELT-VKGD--------AASAI--LNQNGVCARVLCD
T_theileri_Tth.6.1080/1-569     KSSWCLDGDSL-QVYNDEAFLE--EHKVT----VAGVCVEVFCS
T_theileri_Tth.14.1400/1-348    --------------------------------------------
T_theileri_Tth.21.1050/1-407    --------------------------------------------
T_theileri_Tth.144.1030/1-653   NDSWCLDVDYP-NLTIDEEKKM-----------KRGMCAAVNCS
T_theileri_Tth.269.1000/1-185   --------------------------------------------
T_theileri_Tth.144.1020/1-412   ----------------------------------QGICAKVRCD
T_theileri_Tth.151.1020/1-515   PDSFCLDAEEL-KIEGA----------ANAILNQNGVCARVLCD
T_theileri_Tth.11.3450/1-561    VNSRCLTGDHL-ILYNKP---------------VGDICVEVLCG
T_theileri_Tth.166.1000/1-479   NISRCLKGENL-QLKT----PN--ENNLT----VGDICANVKCD
T_theileri_Tth.54.1350/1-777    NNSRCVEGKGL-TANN----------NAA----VGAVCVEVSCK
T_theileri_Tth.10.2520/1-446    NMSRCLKAENL-KLKKGDMK-------------VLGVCAKLKCE
```

```
                                    1240      1250      1260      1270
T_theileri_Tth.43.1980/1-746    -NDNKKVSVQLKG-----HDNK---ELN-WYKC-ENDGE---SI
T_theileri_Tth.32.2230/1-717    ---NGKVSVQYKG-----MKDTH------WIECSGENATIKL--
T_theileri_Tth.11.3610/1-173    --------------------------------------------
T_theileri_Tth.46.1030/1-525    -KNTKKVSVQYSG-----TKD--------WQEC-KDGK-----V
T_theileri_Tth.36.1960/1-517    -KNE--VSVRYSG-----NNE--------WYSC-PEGGRLA---
T_theileri_Tth.165.1020/1-245   ---NDTVRVQYNG-----DDT--------WHLC-PQGQTIAV--
T_theileri_Tth.46.1120/1-413    --------------------------------------------
T_theileri_Tth.24.2780/1-672    -YEKRTVKVEYKGKGD--KNI--------LNECP-ENKM-----
T_theileri_Tth.8.4720/1-600     -NGK--VHVHYKG-----DKEKVNGEQE-WHEC-TEEG----TI
T_theileri_Tth.165.1030/1-156   ---NDTVSVQYKG-----DET--------WHLC-PQGETIAV--
T_theileri_Tth.6.5040/1-514     ---NGTLKVQYKG-----NKN--------WYEC-RDGGYIDYL-
T_theileri_Tth.31.1000/1-129    --------------------------------------------
T_theileri_Tth.11.3650/1-197    --------------------------------------------
T_theileri_Tth.19.2150/1-142    --------------------------------------------
T_theileri_Tth.4.4260/1-444     --EEGKLSVQFQG-----DDE--------WYQCE-EGAHV----
T_theileri_Tth.70.1040/1-472    ---DGKVKVKYSG-----TDN--------WHVC-NNDNS---VI
T_theileri_Tth.46.1080/1-427    ---DGKVKVQYKG-----NDK--------WHEC-PDGGKIENL-
T_theileri_Tth.251.1000/1-344   --------------------------------------------
T_theileri_Tth.27.1390/1-380    ---NDTVQLRYFG-----DDT--------WYECP-EGSTI----
T_theileri_Tth.12.1020/1-102    --------------------------------------------
T_theileri_Tth.12.1830/1-342    --------------------------------------------
T_theileri_Tth.11.3140/1-251    -ESNRKVKVRYSD-----LSE--------FQECP-EGGT-----
T_theileri_Tth.286.1000/1-493   ---TGFVEVRFDD-----KDE--------WHKCDEEDQTFKPN-
T_theileri_Tth.61.1060/1-159    --------------------------------------------
T_theileri_Tth.10.2860/1-651    -KETKKVSVLLKG-----ENE----KES-WHDC-SDGKK---TF
T_theileri_Tth.11.2120/1-102    --------------------------------------------
T_theileri_Tth.132.1030/1-666   -KDKQTVSVQLKG-----YD-E-N---K-WHKC-SDDEV---TI
T_theileri_Tth.101.1090/1-119   --------------------------------------------
T_theileri_Tth.23.2140/1-546    ---GNDVSVKYKG-----SDS--------WQKCH-EGETIT---
T_theileri_Tth.10.2510/1-395    --------------------------------------------
T_theileri_Tth.107.1030/1-864   -FNK--VIVRYNG-----NDN--------WYSC-PEGENLT---
T_theileri_Tth.31.1060/1-887    ---KNKVFVWYNG-----TND--------WQEC-SDGKK----I
T_theileri_Tth.132.1020/1-714   -KDKQTVSVQLKG-----YD-E-N---K-WHKC-SDDEV---TI
T_theileri_Tth.136.1020/1-209   --------------------------------------------
T_theileri_Tth.129.1020/1-290   --------------------------------------------
T_theileri_Tth.71.1080/1-526    ---GGKVSVQYKG-----DDH--------WYEC-KEGSSISPS-
T_theileri_Tth.36.2120/1-796    -FKK--VIVRYSG-----NKE--------WHSC-PEGKNLT---
T_theileri_Tth.63.1240/1-819    -NETKKVSV-LKG-----YKEN---KEE-WHEC-ANDGD---EF
T_theileri_Tth.11.3250/1-309    -ESNRTVKVKYSTSADQ-ATD--------WEECP-EGGN-----
T_theileri_Tth.11.3240/1-592    -ERRRTVSVQYKG-----SDT--------FKECP-EGTSID---
T_theileri_Tth.12.1000/1-649    --DDGVVKVQLKK-----DGE--------WYNCPKEGGEI----
T_theileri_Tth.83.1080/1-228    --------VQYKG-----DET--------WHLCPADQSL-----
T_theileri_Tth.85.1030/1-137    --------------------------------------------
T_theileri_Tth.71.1030/1-204    --------------------------------------------
T_theileri_Tth.97.1060/1-928    -NETKKVSV-LKG-----NDES---TEDNWHKC-PEEGG---SI
T_theileri_Tth.7.4630/1-775     ---YNVVSVKYKG-----NEK--------WHLC-AEGTKLE---
T_theileri_Tth.24.1450/1-463    -YEKRTVKVQYKG-----SNT--------FTECS-EGSS-----
T_theileri_Tth.6.1080/1-569     ---QQSVKVRYHG-----NED--------WYHCP-EGSFIT---
T_theileri_Tth.14.1400/1-348    --------------------------------------------
T_theileri_Tth.21.1050/1-407    --------------------------------------------
T_theileri_Tth.144.1030/1-653   --DNGVFRVQLTV-----DGP--------WHNC--WGNEF----
T_theileri_Tth.269.1000/1-185   --------------------------------------------
T_theileri_Tth.144.1020/1-412   --NDKKVSVQLKGHVGNKEER--------WHKCPEDGGS-----
T_theileri_Tth.151.1020/1-515   -YEKRTVSVKYNG-----SDT--------FKECP-EGSS-----
T_theileri_Tth.11.3450/1-561    ---NGKVHIRYLG-----DDE--------WYPCP-EGTSI----
T_theileri_Tth.166.1000/1-479   -KKNNKVSVQYSG-----SDK--------WHVC-EDGK-----I
T_theileri_Tth.54.1350/1-777    -FNK--VIVRYSG-----NKE--------WYSC-PEGKNLT---
T_theileri_Tth.10.2520/1-446    --DGKKVSVQLKGY---GKDE--------WHNCEEDKNE-----
```

```
                                  1280      1290      1300      1310
T_theileri_Tth.43.1980/1-746     P---L-----EDSLF--NKGSVK-CPNFKEVCTGLPKTEPT-KI
T_theileri_Tth.32.2230/1-717     ----------ESSAF--KGGSIV-CPRYEEVCTGLPDLVDVTST
T_theileri_Tth.11.3610/1-173     --------------------------------------------
T_theileri_Tth.46.1030/1-525     DV--------TXXXXXXXXXRI----------------------
T_theileri_Tth.36.1960/1-517     ----V-----QGTVL--Q-GKIV-CPKYADVCNTINRVIDKERG
T_theileri_Tth.165.1020/1-245    ----------TGSAF--SGGKIR-CPKYSEVCSREPSSAGITVF
T_theileri_Tth.46.1120/1-413     --------------------------------------------
T_theileri_Tth.24.2780/1-672     ----ID---VELSES--ASGKIK-CPKYDEVCTVASNGSSLVPF
T_theileri_Tth.8.4720/1-600      -N--L-----QGSVF--TSGTIV-CPKYAEVCTGLPETEPF-NI
T_theileri_Tth.165.1030/1-156    ----------TGSAF--SGGKIQ-CPKY----------------
T_theileri_Tth.6.5040/1-514      ----------QGSVF--DDGRIL-CPKYTDVCTK----------
T_theileri_Tth.31.1000/1-129     --------------------------------------------
T_theileri_Tth.11.3650/1-197     --------------------------------------------
T_theileri_Tth.19.2150/1-142     --------------------------------------------
T_theileri_Tth.4.4260/1-444      ---------KSNSAN--WSGTIT-CPKYADVCTRYPNITNKEPL
T_theileri_Tth.70.1040/1-472     TP--E-----GGSAF--RGGSIL-CPKYAEVCTELNST--IDFR
T_theileri_Tth.46.1080/1-427     ----------EGTEF--QSGSIL-CPKYAEVCTDLPQISELRIE
T_theileri_Tth.251.1000/1-344    ------------------------VPRLST--------------
T_theileri_Tth.27.1390/1-380     ---------TPVTTF--SSGRIV-CPPRADVCIDSGDEHSDSSD
T_theileri_Tth.12.1020/1-102     --------------------------------------------
T_theileri_Tth.12.1830/1-342     --------------------------------------------
T_theileri_Tth.11.3140/1-251     ----IT---VSSSSASLNFTTIH-CPNYTEVCTIAPNGSSLLPL
T_theileri_Tth.286.1000/1-493    ----------MAAAFDYVSGKVK-CPKYSEV-------------
T_theileri_Tth.61.1060/1-159     --------------------------------------------
T_theileri_Tth.10.2860/1-651     DV--V-----AGSVF--SGGKIE-CPKFEEVCTGLLEAETPTNI
T_theileri_Tth.11.2120/1-102     --------------------------------------------
T_theileri_Tth.132.1030/1-666    DD--V-----EGSVF--KSGTIIRCPKYEEVCTKERNTDHLPDI
T_theileri_Tth.101.1090/1-119    --------------------------------------------
T_theileri_Tth.23.2140/1-546     ---------PKGSKF--VSGSII-CPKYNEVCTVAPDGGSLAKA
T_theileri_Tth.10.2510/1-395     --------------------------------------------
T_theileri_Tth.107.1030/1-864    ----V-----NGSVL--Q-GEIV-CPKYADVCNTINKTLDESQG
T_theileri_Tth.31.1060/1-887     DV--K-----DSTEF--TGGSIV-CPNYAEVCTELNST--VDFY
T_theileri_Tth.132.1020/1-714    DD--V-----EGSVF--KSGTIV-CPKFEEVCTKETGTDPF-NI
T_theileri_Tth.136.1020/1-209    --------------------------------------------
T_theileri_Tth.129.1020/1-290    --------------------------------------------
T_theileri_Tth.71.1080/1-526     ----------VTSAF--SSGRIV-CPPYSEVCMDLPIQPPPVEA
T_theileri_Tth.36.2120/1-796     ----V-----NGTVL--Q-GNIV-CPKYADVCNTINKTLDESEG
T_theileri_Tth.63.1240/1-819     H---L-----VDSEF--NEGSVN-CPKYEEVCIGLLETEVPTNI
T_theileri_Tth.11.3250/1-309     ----IT---VSSSSASLKFKTIY-CPNYTEVCTIASNGSSLRPL
T_theileri_Tth.11.3240/1-592     ---------VESSAFQ-SGGKIK-CPKYDEVCTIALNGSSLVPF
T_theileri_Tth.12.1000/1-649     ----IPLNGTDKLSY--VSGSIK-CPKYDEVCTLRFNGSSRLRL
T_theileri_Tth.83.1080/1-228     -------ELAQKGVF--SSGSII-CPAKEEVCTSLKGAVLPVEN
T_theileri_Tth.85.1030/1-137     --------------------------------------------
T_theileri_Tth.71.1030/1-204     --------------------------------------------
T_theileri_Tth.97.1060/1-928     NIAHV-----AGTEF--SGGSIE-CPKYEEVCIGLLETEAPTNI
T_theileri_Tth.7.4630/1-775      ---------VNTNEL---KGQIV-CPKYADVCNTINVTLDELEG
T_theileri_Tth.24.1450/1-463     ----ID---VKSSVF--ESGKIK-CPKYDEVCTIAYNGSSHVPF
T_theileri_Tth.6.1080/1-569      ---------PNSSTF--AGGRIK-CPKRSEVCMIAPSGRSNVLR
T_theileri_Tth.14.1400/1-348     --------------------------------------------
T_theileri_Tth.21.1050/1-407     --------------------------------------------
T_theileri_Tth.144.1030/1-653    ----IMPNKTDYPMY--AGGRIK-CPIYDEVCTVKSDGSSRLTS
T_theileri_Tth.269.1000/1-185    --------------------------------------------
T_theileri_Tth.144.1020/1-412    ----INIADVAGTEF--SEGTIV-CPKYEEVCTGWPETNSPV-I
T_theileri_Tth.151.1020/1-515    ----ID---VKSSVF--ESGKIK-CPKYDEVCIIAFNGSSRVPF
T_theileri_Tth.11.3450/1-561     ---------TPMKTF--SSGRIQ-CPMVHEVCDESNVAFTTLTS
T_theileri_Tth.166.1000/1-479    EV--T-----DGQEF--QSGSIL-CPKYSEVCDDFSEVKDIN--
T_theileri_Tth.54.1350/1-777     ----V-----NGDLL--K-GEIV-CPKYADVCNTINKTLDESQG
T_theileri_Tth.10.2520/1-446     ----IKL---VGSEF--SEGSIK-CPKYEEVCIGLLEAEVPTSI
```

```
                                      1330      1340      1350      1360
T_theileri_Tth.43.1980/1-746    KFYNGTKITESYVDVKDDDENEE----------PAQAQNTVRTA
T_theileri_Tth.32.2230/1-717    EEEEQEVARLP--EVDNKGNEGT---------------------
T_theileri_Tth.11.3610/1-173    --------------------------------------------
T_theileri_Tth.46.1030/1-525    --------------------------------------------
T_theileri_Tth.36.1960/1-517    RPMKLGAYVD----------------------------------
T_theileri_Tth.165.1020/1-245   TPVDIKESTGTHSSSSAASTRSD---------------------
T_theileri_Tth.46.1120/1-413    --------------------------------------------
T_theileri_Tth.24.2780/1-672    VNPHPAPPAAGP--------------------------------
T_theileri_Tth.8.4720/1-600     PYEEVVEKPE---------EEEE----------EEK---P----
T_theileri_Tth.165.1030/1-156   --------------------------------------------
T_theileri_Tth.6.5040/1-514     --------------------------------------------
T_theileri_Tth.31.1000/1-129    --------------------------------------------
T_theileri_Tth.11.3650/1-197    --------------------------------------------
T_theileri_Tth.19.2150/1-142    --------------------------------------------
T_theileri_Tth.4.4260/1-444     PEVTDKDNEPAPANITWPIKPKD---------------------
T_theileri_Tth.70.1040/1-472    ITYDEDEKRLMEEEDKQEAEE---EKQRIMAQEESEGEA-----
T_theileri_Tth.46.1080/1-427    SNEAADIEILQDYVIGRDLHDIS---------------------
T_theileri_Tth.251.1000/1-344   --------------------------------------------
T_theileri_Tth.27.1390/1-380    GHSDSSDGHSDSSDGHSDSSDGHSDSSDEHSDSGDEHSDSGNKH
T_theileri_Tth.12.1020/1-102    --------------------------------------------
T_theileri_Tth.12.1830/1-342    --------------------------------------------
T_theileri_Tth.11.3140/1-251    VKPPDPTPPGGGGGHG----------------------------
T_theileri_Tth.286.1000/1-493   --------------------------------------------
T_theileri_Tth.61.1060/1-159    --------------------------------------------
T_theileri_Tth.10.2860/1-651    KFYDGTEVTNGYKGDVIPVKEEE---------------------
T_theileri_Tth.11.2120/1-102    --------------------------------------------
T_theileri_Tth.132.1030/1-666   KYNVVNVSEDVVE-E---PKQEE----------EEK---E----
T_theileri_Tth.101.1090/1-119   --------------------------------------------
T_theileri_Tth.23.2140/1-546    TS------------------------------------------
T_theileri_Tth.10.2510/1-395    --------------------------------------------
T_theileri_Tth.107.1030/1-864   PEKDPDIVEAIQTTLPEGETTSA---------------------
T_theileri_Tth.31.1060/1-887    ITYDEDEKRQMEKEKQEAEDK---RKQEEEERHRIMAQE-----
T_theileri_Tth.132.1020/1-714   SYNVVNVPED--E-E---PKQEE----------KKK---E----
T_theileri_Tth.136.1020/1-209   --------------------------------------------
T_theileri_Tth.129.1020/1-290   --------------------------------------------
T_theileri_Tth.71.1080/1-526    VV--MEEVYNFTSSINLINS------------------------
T_theileri_Tth.36.2120/1-796    PSKDPDIVEEVQTSQPE---------------------------
T_theileri_Tth.63.1240/1-819    KFHNGTKVTDVYD-GDVSGGNEE----------KEEVVENQDRT
T_theileri_Tth.11.3250/1-309    VKPSDRKPTPATQVPGNSSTTPV---------------------
T_theileri_Tth.11.3240/1-592    VNPHQPSAVGAT--------------------------------
T_theileri_Tth.12.1000/1-649    QVEEEKQNTPATEENVVNGTTDT---------------------
T_theileri_Tth.83.1080/1-228    ETDVLKENENDDIAPIADANPVT---------------------
T_theileri_Tth.85.1030/1-137    --------------------------------------------
T_theileri_Tth.71.1030/1-204    --------------------------------------------
T_theileri_Tth.97.1060/1-928    KFYNAKKITDGYVDVNVAKEEKKKEVKQEKKKPIDAQTQVVSPE
T_theileri_Tth.7.4630/1-775     PPPPPEPGIQSNTPGAKSPTGAE---------------------
T_theileri_Tth.24.1450/1-463    VVNRPPAATAEESASFGD--------------------------
T_theileri_Tth.6.1080/1-569     ERDAKSQKNLKKK-------------------------------
T_theileri_Tth.14.1400/1-348    --------------------------------------------
T_theileri_Tth.21.1050/1-407    --------------------------------------------
T_theileri_Tth.144.1030/1-653   QANENTPLEIKAIWVSSTPTNPT---------------------
T_theileri_Tth.269.1000/1-185   --------------------------------------------
T_theileri_Tth.144.1020/1-412   KLNSDKEESDGYYIVLNDGEKEA---------------------
T_theileri_Tth.151.1020/1-515   VVNRPPASSAAAEESASSVD------------------------
T_theileri_Tth.11.3450/1-561    IS------------------------------------------
T_theileri_Tth.166.1000/1-479   --------------------------------------------
T_theileri_Tth.54.1350/1-777    PATDPDTVEEVQTSLPE---------------------------
T_theileri_Tth.10.2520/1-446    KFYNGKSVTDGYVGDVKDEEKEA---------------------
```

```
                                    1370      1380      1390      1400
T_theileri_Tth.43.1980/1-746   GASAVANQGSSAPDARPSAQPEGQQHPNPQQEVQEVEKEIKSQT
T_theileri_Tth.32.2230/1-717   --------------------------------------------
T_theileri_Tth.11.3610/1-173   --------------------------------------------
T_theileri_Tth.46.1030/1-525   --------------------------------------------
T_theileri_Tth.36.1960/1-517   --------------------------------------------
T_theileri_Tth.165.1020/1-245  --------------------------------------------
T_theileri_Tth.46.1120/1-413   --------------------------------------------
T_theileri_Tth.24.2780/1-672   --------------------------------------------
T_theileri_Tth.8.4720/1-600    --------------------------------------------
T_theileri_Tth.165.1030/1-156  --------------------------------------------
T_theileri_Tth.6.5040/1-514    --------------------------------------------
T_theileri_Tth.31.1000/1-129   --------------------------------------------
T_theileri_Tth.11.3650/1-197   --------------------------------------------
T_theileri_Tth.19.2150/1-142   --------------------------------------------
T_theileri_Tth.4.4260/1-444    --------------------------------------------
T_theileri_Tth.70.1040/1-472   ----------------------------------------LQQK
T_theileri_Tth.46.1080/1-427   --------------------------------------------
T_theileri_Tth.251.1000/1-344  --------------------------------------------
T_theileri_Tth.27.1390/1-380   NDWINMIMWEISKRIEDMSIKIRAMSIKI---------------
T_theileri_Tth.12.1020/1-102   --------------------------------------------
T_theileri_Tth.12.1830/1-342   --------------------------------------------
T_theileri_Tth.11.3140/1-251   --------------------------------------------
T_theileri_Tth.286.1000/1-493  --------------------------------------------
T_theileri_Tth.61.1060/1-159   --------------------------------------------
T_theileri_Tth.10.2860/1-651   --------------------------------------------
T_theileri_Tth.11.2120/1-102   --------------------------------------------
T_theileri_Tth.132.1030/1-666  --------------------------------------------
T_theileri_Tth.101.1090/1-119  --------------------------------------------
T_theileri_Tth.23.2140/1-546   --------------------------------------------
T_theileri_Tth.10.2510/1-395   --------------------------------------------
T_theileri_Tth.107.1030/1-864  --------------------------------------------
T_theileri_Tth.31.1060/1-887   --------------------------------AHNERIQRDAQP
T_theileri_Tth.132.1020/1-714  --------------------------------------------
T_theileri_Tth.136.1020/1-209  --------------------------------------------
T_theileri_Tth.129.1020/1-290  --------------------------------------------
T_theileri_Tth.71.1080/1-526   --------------------------------------------
T_theileri_Tth.36.2120/1-796   --------------------------------------------
T_theileri_Tth.63.1240/1-819   --------------------------------------------
T_theileri_Tth.11.3250/1-309   --------------------------------------------
T_theileri_Tth.11.3240/1-592   --------------------------------------------
T_theileri_Tth.12.1000/1-649   --------------------------------------------
T_theileri_Tth.83.1080/1-228   --------------------------------------------
T_theileri_Tth.85.1030/1-137   --------------------------------------------
T_theileri_Tth.71.1030/1-204   --------------------------------------------
T_theileri_Tth.97.1060/1-928   PHTAPSNVIQNV--------------------------------
T_theileri_Tth.7.4630/1-775    --------------------------------------------
T_theileri_Tth.24.1450/1-463   --------------------------------------------
T_theileri_Tth.6.1080/1-569    --------------------------------------------
T_theileri_Tth.14.1400/1-348   --------------------------------------------
T_theileri_Tth.21.1050/1-407   --------------------------------------------
T_theileri_Tth.144.1030/1-653  --------------------------------------------
T_theileri_Tth.269.1000/1-185  --------------------------------------------
T_theileri_Tth.144.1020/1-412  --------------------------------------------
T_theileri_Tth.151.1020/1-515  --------------------------------------------
T_theileri_Tth.11.3450/1-561   --------------------------------------------
T_theileri_Tth.166.1000/1-479  --------------------------------------------
T_theileri_Tth.54.1350/1-777   --------------------------------------------
T_theileri_Tth.10.2520/1-446   --------------------------------------------
```

```
                                       1410      1420      1430      1440      1450
T_theileri_Tth.43.1980/1-746     --------------------------------------------
T_theileri_Tth.32.2230/1-717     --------------------------------------------
T_theileri_Tth.11.3610/1-173     --------------------------------------------
T_theileri_Tth.46.1030/1-525     --------------------------------------------
T_theileri_Tth.36.1960/1-517     --------------------------------------------
T_theileri_Tth.165.1020/1-245    --------------------------------------------
T_theileri_Tth.46.1120/1-413     --------------------------------------------
T_theileri_Tth.24.2780/1-672     --------------------------------------------
T_theileri_Tth.8.4720/1-600      --------------------------------------------
T_theileri_Tth.165.1030/1-156    --------------------------------------------
T_theileri_Tth.6.5040/1-514      --------------------------------------------
T_theileri_Tth.31.1000/1-129     --------------------------------------------
T_theileri_Tth.11.3650/1-197     --------------------------------------------
T_theileri_Tth.19.2150/1-142     --------------------------------------------
T_theileri_Tth.4.4260/1-444      --------------------------------------------
T_theileri_Tth.70.1040/1-472     NEQEEAKSREGKLASQTQPQPSIPALA-----------------
T_theileri_Tth.46.1080/1-427     --------------------------------------------
T_theileri_Tth.251.1000/1-344    --------------------------------------------
T_theileri_Tth.27.1390/1-380     --------------------------------------------
T_theileri_Tth.12.1020/1-102     --------------------------------------------
T_theileri_Tth.12.1830/1-342     --------------------------------------------
T_theileri_Tth.11.3140/1-251     --------------------------------------------
T_theileri_Tth.286.1000/1-493    --------------------------------------------
T_theileri_Tth.61.1060/1-159     --------------------------------------------
T_theileri_Tth.10.2860/1-651     --------------------------------------------
T_theileri_Tth.11.2120/1-102     --------------------------------------------
T_theileri_Tth.132.1030/1-666    --------------------------------------------
T_theileri_Tth.101.1090/1-119    --------------------------------------------
T_theileri_Tth.23.2140/1-546     --------------------------------------------
T_theileri_Tth.10.2510/1-395     --------------------------------------------
T_theileri_Tth.107.1030/1-864    --------------------------------------------
T_theileri_Tth.31.1060/1-887     EDQTPL---------HPDPKQNTPSKP-----------------
T_theileri_Tth.132.1020/1-714    --------------------------------------------
T_theileri_Tth.136.1020/1-209    --------------------------------------------
T_theileri_Tth.129.1020/1-290    --------------------------------------------
T_theileri_Tth.71.1080/1-526     --------------------------------------------
T_theileri_Tth.36.2120/1-796     --------------------------------------------
T_theileri_Tth.63.1240/1-819     --------------------------------------------
T_theileri_Tth.11.3250/1-309     --------------------------------------------
T_theileri_Tth.11.3240/1-592     --------------------------------------------
T_theileri_Tth.12.1000/1-649     --------------------------------------------
T_theileri_Tth.83.1080/1-228     --------------------------------------------
T_theileri_Tth.85.1030/1-137     --------------------------------------------
T_theileri_Tth.71.1030/1-204     --------------------------------------------
T_theileri_Tth.97.1060/1-928     --------------------------------------------
T_theileri_Tth.7.4630/1-775      --------------------------------------------
T_theileri_Tth.24.1450/1-463     --------------------------------------------
T_theileri_Tth.6.1080/1-569      --------------------------------------------
T_theileri_Tth.14.1400/1-348     --------------------------------------------
T_theileri_Tth.21.1050/1-407     --------------------------------------------
T_theileri_Tth.144.1030/1-653    --------------------------------------------
T_theileri_Tth.269.1000/1-185    --------------------------------------------
T_theileri_Tth.144.1020/1-412    --------------------------------------------
T_theileri_Tth.151.1020/1-515    --------------------------------------------
T_theileri_Tth.11.3450/1-561     --------------------------------------------
T_theileri_Tth.166.1000/1-479    --------------------------------------------
T_theileri_Tth.54.1350/1-777     --------------------------------------------
T_theileri_Tth.10.2520/1-446     --------------------------------------------
```

```
                                          1460      1470      1480      1490
T_theileri_Tth.43.1980/1-746    ------------------------------------LSLKHPES
T_theileri_Tth.32.2230/1-717    --------------------------------------------
T_theileri_Tth.11.3610/1-173    --------------------------------------------
T_theileri_Tth.46.1030/1-525    --------------------------------------------
T_theileri_Tth.36.1960/1-517    --------------------------------------------
T_theileri_Tth.165.1020/1-245   --------------------------------------------
T_theileri_Tth.46.1120/1-413    --------------------------------------------
T_theileri_Tth.24.2780/1-672    --------------------------------------------
T_theileri_Tth.8.4720/1-600     ------------------------------------EEEEN---
T_theileri_Tth.165.1030/1-156   --------------------------------------------
T_theileri_Tth.6.5040/1-514     --------------------------------------------
T_theileri_Tth.31.1000/1-129    --------------------------------------------
T_theileri_Tth.11.3650/1-197    --------------------------------------------
T_theileri_Tth.19.2150/1-142    --------------------------------------------
T_theileri_Tth.4.4260/1-444     --------------------------------------------
T_theileri_Tth.70.1040/1-472    -------------------------------------VGQRGLA
T_theileri_Tth.46.1080/1-427    --------------------------------------------
T_theileri_Tth.251.1000/1-344   --------------------------------------------
T_theileri_Tth.27.1390/1-380    --------------------------------------------
T_theileri_Tth.12.1020/1-102    --------------------------------------------
T_theileri_Tth.12.1830/1-342    --------------------------------------------
T_theileri_Tth.11.3140/1-251    --------------------------------------------
T_theileri_Tth.286.1000/1-493   --------------------------------------------
T_theileri_Tth.61.1060/1-159    --------------------------------------------
T_theileri_Tth.10.2860/1-651    --------------------------------------------
T_theileri_Tth.11.2120/1-102    --------------------------------------------
T_theileri_Tth.132.1030/1-666   ------------------------------------NKDQNNSS
T_theileri_Tth.101.1090/1-119   --------------------------------------------
T_theileri_Tth.23.2140/1-546    --------------------------------------------
T_theileri_Tth.10.2510/1-395    --------------------------------------------
T_theileri_Tth.107.1030/1-864   --------------------------------------------
T_theileri_Tth.31.1060/1-887    -------------------------------------------A
T_theileri_Tth.132.1020/1-714   ------------------------------------AEVQSNSS
T_theileri_Tth.136.1020/1-209   --------------------------------------------
T_theileri_Tth.129.1020/1-290   --------------------------------------------
T_theileri_Tth.71.1080/1-526    --------------------------------------------
T_theileri_Tth.36.2120/1-796    --------------------------------------------
T_theileri_Tth.63.1240/1-819    ------------------------------------DEEKPPAS
T_theileri_Tth.11.3250/1-309    --------------------------------------------
T_theileri_Tth.11.3240/1-592    --------------------------------------------
T_theileri_Tth.12.1000/1-649    --------------------------------------------
T_theileri_Tth.83.1080/1-228    --------------------------------------------
T_theileri_Tth.85.1030/1-137    --------------------------------------------
T_theileri_Tth.71.1030/1-204    --------------------------------------------
T_theileri_Tth.97.1060/1-928    ------------------------------------DSLPAATP
T_theileri_Tth.7.4630/1-775     --------------------------------------------
T_theileri_Tth.24.1450/1-463    --------------------------------------------
T_theileri_Tth.6.1080/1-569     --------------------------------------------
T_theileri_Tth.14.1400/1-348    --------------------------------------------
T_theileri_Tth.21.1050/1-407    --------------------------------------------
T_theileri_Tth.144.1030/1-653   --------------------------------------------
T_theileri_Tth.269.1000/1-185   --------------------------------------------
T_theileri_Tth.144.1020/1-412   ---------------------KPEKGQKPTHA--PSVSSVSPEE
T_theileri_Tth.151.1020/1-515   --------------------------------------------
T_theileri_Tth.11.3450/1-561    --------------------------------------------
T_theileri_Tth.166.1000/1-479   --------------------------------------------
T_theileri_Tth.54.1350/1-777    --------------------------------------------
T_theileri_Tth.10.2520/1-446    ---------------------KPVQVQKLPHVSLPPPGPLVNED
```

	1500 1510 1520 1530
T_theileri_Tth.43.1980/1-746	ENAEHTGSSSVTPGSNTGSGGNNNDNGSNEDQNMGQARESASTR
T_theileri_Tth.32.2230/1-717	--
T_theileri_Tth.11.3610/1-173	--
T_theileri_Tth.46.1030/1-525	--
T_theileri_Tth.36.1960/1-517	--
T_theileri_Tth.165.1020/1-245	--
T_theileri_Tth.46.1120/1-413	--
T_theileri_Tth.24.2780/1-672	--
T_theileri_Tth.8.4720/1-600	--
T_theileri_Tth.165.1030/1-156	--
T_theileri_Tth.6.5040/1-514	--
T_theileri_Tth.31.1000/1-129	--
T_theileri_Tth.11.3650/1-197	--
T_theileri_Tth.19.2150/1-142	--
T_theileri_Tth.4.4260/1-444	--
T_theileri_Tth.70.1040/1-472	VAGNANGASHVPTTPSNAGNQRGSPIGVSEKEVVQPSSAASEQH
T_theileri_Tth.46.1080/1-427	--
T_theileri_Tth.251.1000/1-344	--
T_theileri_Tth.27.1390/1-380	--
T_theileri_Tth.12.1020/1-102	--
T_theileri_Tth.12.1830/1-342	--
T_theileri_Tth.11.3140/1-251	--
T_theileri_Tth.286.1000/1-493	--
T_theileri_Tth.61.1060/1-159	--
T_theileri_Tth.10.2860/1-651	--
T_theileri_Tth.11.2120/1-102	--
T_theileri_Tth.132.1030/1-666	TNPEVSGGV-ANTGNETS-----------------------TN-
T_theileri_Tth.101.1090/1-119	--
T_theileri_Tth.23.2140/1-546	--
T_theileri_Tth.10.2510/1-395	--
T_theileri_Tth.107.1030/1-864	--
T_theileri_Tth.31.1060/1-887	EDSAGDSSGKQSLRPSQDRNQRDSA-GVGQDGVVSSLPAAPQSQ
T_theileri_Tth.132.1020/1-714	TNSEASGDL-ANADTQTS-----------------------AN-
T_theileri_Tth.136.1020/1-209	--
T_theileri_Tth.129.1020/1-290	--
T_theileri_Tth.71.1080/1-526	--
T_theileri_Tth.36.2120/1-796	--
T_theileri_Tth.63.1240/1-819	LPTAPNGDLVENEKVPTENGNNETVTEKEPEDTVSKPQVPPTT-
T_theileri_Tth.11.3250/1-309	--
T_theileri_Tth.11.3240/1-592	--
T_theileri_Tth.12.1000/1-649	--
T_theileri_Tth.83.1080/1-228	--
T_theileri_Tth.85.1030/1-137	--
T_theileri_Tth.71.1030/1-204	--
T_theileri_Tth.97.1060/1-928	LARENKHGQKNQGPIVPQDAHGKHPESENVEHTGSSSLGPESNT
T_theileri_Tth.7.4630/1-775	--
T_theileri_Tth.24.1450/1-463	--
T_theileri_Tth.6.1080/1-569	--
T_theileri_Tth.14.1400/1-348	--
T_theileri_Tth.21.1050/1-407	--
T_theileri_Tth.144.1030/1-653	--
T_theileri_Tth.269.1000/1-185	--
T_theileri_Tth.144.1020/1-412	DESHVELHESDIVKEAPESEHPDKHEAPKGQEPVKHRDASHNSL
T_theileri_Tth.151.1020/1-515	--
T_theileri_Tth.11.3450/1-561	--
T_theileri_Tth.166.1000/1-479	--
T_theileri_Tth.54.1350/1-777	--
T_theileri_Tth.10.2520/1-446	GESSALDHQVAREEAASLSNPPNVNRETESL-------PSHPTG

```
                                     1550      1560      1570      1580
T_theileri_Tth.43.1980/1-746    VDV--------------------------PSGQSLSSPPAAAGS
T_theileri_Tth.32.2230/1-717    --------------------------------------------
T_theileri_Tth.11.3610/1-173    --------------------------------------------
T_theileri_Tth.46.1030/1-525    --------------------------------------------
T_theileri_Tth.36.1960/1-517    --------------------------------------------
T_theileri_Tth.165.1020/1-245   --------------------------------------------
T_theileri_Tth.46.1120/1-413    --------------------------------------------
T_theileri_Tth.24.2780/1-672    --------------------------------------------
T_theileri_Tth.8.4720/1-600     --------------------------------------PGEEEE
T_theileri_Tth.165.1030/1-156   --------------------------------------------
T_theileri_Tth.6.5040/1-514     --------------------------------------------
T_theileri_Tth.31.1000/1-129    --------------------------------------------
T_theileri_Tth.11.3650/1-197    --------------------------------------------
T_theileri_Tth.19.2150/1-142    --------------------------------------------
T_theileri_Tth.4.4260/1-444     --------------------------------------------
T_theileri_Tth.70.1040/1-472    IERREKQAHESKTNNN-GDHSQRQPSISAPNVPSDTKRPEMEKN
T_theileri_Tth.46.1080/1-427    --------------------------------------------
T_theileri_Tth.251.1000/1-344   --------------------------------------------
T_theileri_Tth.27.1390/1-380    --------------------------------------------
T_theileri_Tth.12.1020/1-102    --------------------------------------------
T_theileri_Tth.12.1830/1-342    --------------------------------------------
T_theileri_Tth.11.3140/1-251    --------------------------------------------
T_theileri_Tth.286.1000/1-493   --------------------------------------------
T_theileri_Tth.61.1060/1-159    --------------------------------------------
T_theileri_Tth.10.2860/1-651    --------------------------------------------
T_theileri_Tth.11.2120/1-102    --------------------------------------------
T_theileri_Tth.132.1030/1-666   -----------------------------TQGESAPAAP-----
T_theileri_Tth.101.1090/1-119   --------------------------------------------
T_theileri_Tth.23.2140/1-546    --------------------------------------------
T_theileri_Tth.10.2510/1-395    --------------------------------------------
T_theileri_Tth.107.1030/1-864   --------------------------------------------
T_theileri_Tth.31.1060/1-887    PAREENRALENSNNNN-GDEPHQQPSISAPNVPSDTKTSEMEKN
T_theileri_Tth.132.1020/1-714   -----------------------------TQGGSAPAAPDVNGS
T_theileri_Tth.136.1020/1-209   --------------------------------------------
T_theileri_Tth.129.1020/1-290   --------------------------------------------
T_theileri_Tth.71.1080/1-526    --------------------------------------------
T_theileri_Tth.36.2120/1-796    --------------------------------------------
T_theileri_Tth.63.1240/1-819    -----------------------------SEEKDNEKVPTENGS
T_theileri_Tth.11.3250/1-309    --------------------------------------------
T_theileri_Tth.11.3240/1-592    --------------------------------------------
T_theileri_Tth.12.1000/1-649    --------------------------------------------
T_theileri_Tth.83.1080/1-228    --------------------------------------------
T_theileri_Tth.85.1030/1-137    --------------------------------------------
T_theileri_Tth.71.1030/1-204    --------------------------------------------
T_theileri_Tth.97.1060/1-928    GSRGTSTGNSGSDGSGGASGNGGNLVNVSQSAQSEPSSSASASA
T_theileri_Tth.7.4630/1-775     --------------------------------------------
T_theileri_Tth.24.1450/1-463    --------------------------------------------
T_theileri_Tth.6.1080/1-569     --------------------------------------------
T_theileri_Tth.14.1400/1-348    --------------------------------------------
T_theileri_Tth.21.1050/1-407    --------------------------------------------
T_theileri_Tth.144.1030/1-653   --------------------------------------------
T_theileri_Tth.269.1000/1-185   --------------------------------------------
T_theileri_Tth.144.1020/1-412   GNVR--VHNTGVSTASSDGGIGTGGTNTNNNSAVGSGGISNEKH
T_theileri_Tth.151.1020/1-515   --------------------------------------------
T_theileri_Tth.11.3450/1-561    --------------------------------------------
T_theileri_Tth.166.1000/1-479   --------------------------------------------
T_theileri_Tth.54.1350/1-777    --------------------------------------------
T_theileri_Tth.10.2520/1-446    ENVEKNIESTGLSNVGALRNDERLDTNAANPAEHSLSHKSNDTN
```

```
                                      1590      1600      1610      1620
T_theileri_Tth.43.1980/1-746    AT--------------------IDPANTVTREISQQPADQPAQN
T_theileri_Tth.32.2230/1-717    ----------------------------AGEPVSSSIVESETNM
T_theileri_Tth.11.3610/1-173    --------------------------------------------
T_theileri_Tth.46.1030/1-525    --------------------------------------------
T_theileri_Tth.36.1960/1-517    --------------------------------------------
T_theileri_Tth.165.1020/1-245   --------------------------------------------
T_theileri_Tth.46.1120/1-413    --------------------------------------------
T_theileri_Tth.24.2780/1-672    --------------------------------------------
T_theileri_Tth.8.4720/1-600     EMNSGT----------------GDQD-----------------Q
T_theileri_Tth.165.1030/1-156   --------------------------------------------
T_theileri_Tth.6.5040/1-514     --------------------------------------------
T_theileri_Tth.31.1000/1-129    --------------------------------------------
T_theileri_Tth.11.3650/1-197    --------------------------------------------
T_theileri_Tth.19.2150/1-142    --------------------------------------------
T_theileri_Tth.4.4260/1-444     --------------------------------------------
T_theileri_Tth.70.1040/1-472    SSEPAKAQDKEVSI--------------------------SEIS
T_theileri_Tth.46.1080/1-427    --------------------------------------------
T_theileri_Tth.251.1000/1-344   --------------------------------------------
T_theileri_Tth.27.1390/1-380    --------------------------------------------
T_theileri_Tth.12.1020/1-102    --------------------------------------------
T_theileri_Tth.12.1830/1-342    --------------------------------------------
T_theileri_Tth.11.3140/1-251    --------------------------------------------
T_theileri_Tth.286.1000/1-493   --------------------------------------------
T_theileri_Tth.61.1060/1-159    --------------------------------------------
T_theileri_Tth.10.2860/1-651    ------------------------------------------DE
T_theileri_Tth.11.2120/1-102    --------------------------------------------
T_theileri_Tth.132.1030/1-666   --------------------------------------------
T_theileri_Tth.101.1090/1-119   --------------------------------------------
T_theileri_Tth.23.2140/1-546    --------------------------------------------
T_theileri_Tth.10.2510/1-395    --------------------------------------------
T_theileri_Tth.107.1030/1-864   --------------------------------------------
T_theileri_Tth.31.1060/1-887    SSEPAKAQDKEVSISEMPTHVVPQEGPSVPQEPEVEKLPTTETP
T_theileri_Tth.132.1020/1-714   SQQASA----------------APQG-------NSTNTESAREE
T_theileri_Tth.136.1020/1-209   --------------------------------------------
T_theileri_Tth.129.1020/1-290   --------------------------------------------
T_theileri_Tth.71.1080/1-526    --------------------------------------------
T_theileri_Tth.36.2120/1-796    --------------------------------------------
T_theileri_Tth.63.1240/1-819    DGAETE----------------KEPVGTTNTSEVPPNREEEEEK
T_theileri_Tth.11.3250/1-309    --------------------------------------------
T_theileri_Tth.11.3240/1-592    --------------------------------------------
T_theileri_Tth.12.1000/1-649    --------------------------------------------
T_theileri_Tth.83.1080/1-228    --------------------------------------------
T_theileri_Tth.85.1030/1-137    --------------------------------------------
T_theileri_Tth.71.1030/1-204    --------------------------------------------
T_theileri_Tth.97.1060/1-928    PAPAPA--------------PDPASSKAPAHGISQQIADQPAVN
T_theileri_Tth.7.4630/1-775     --------------------------------------------
T_theileri_Tth.24.1450/1-463    --------------------------------------------
T_theileri_Tth.6.1080/1-569     --------------------------------------------
T_theileri_Tth.14.1400/1-348    --------------------------------------------
T_theileri_Tth.21.1050/1-407    --------------------------------------------
T_theileri_Tth.144.1030/1-653   --------------------------------------------
T_theileri_Tth.269.1000/1-185   --------------------------------------------
T_theileri_Tth.144.1020/1-412   PVTVPER-------VHTTPPPPTPAAPLPTPADPLVPAKTPADG
T_theileri_Tth.151.1020/1-515   --------------------------------------------
T_theileri_Tth.11.3450/1-561    --------------------------------------------
T_theileri_Tth.166.1000/1-479   --------------------------------------------
T_theileri_Tth.54.1350/1-777    --------------------------------------------
T_theileri_Tth.10.2520/1-446    QLTVAEQSRENESPVNSLPPAQSSPVPAPAVVGPYDRARANEDL
```

```
                                1630      1640      1650      1660      1670
T_theileri_Tth.43.1980/1-746    NDKQNENETPAEAQD--------------------------HQQ
T_theileri_Tth.32.2230/1-717    RSERTPVSEGSYGV------------------------------
T_theileri_Tth.11.3610/1-173    --------------------------------------------
T_theileri_Tth.46.1030/1-525    --------------------------------------------
T_theileri_Tth.36.1960/1-517    --------------------------------------------
T_theileri_Tth.165.1020/1-245   --------------------------------------------
T_theileri_Tth.46.1120/1-413    --------------------------------------------
T_theileri_Tth.24.2780/1-672    --------------------------------------------
T_theileri_Tth.8.4720/1-600     TQK-----------------------------------------
T_theileri_Tth.165.1030/1-156   --------------------------------------------
T_theileri_Tth.6.5040/1-514     --------------------------------------------
T_theileri_Tth.31.1000/1-129    --------------------------------------------
T_theileri_Tth.11.3650/1-197    --------------------------------------------
T_theileri_Tth.19.2150/1-142    --------------------------------------------
T_theileri_Tth.4.4260/1-444     --------------------------------------------
T_theileri_Tth.70.1040/1-472    KPAASESVVVS-GHTPSV-EEDHNNTTENIEQVQGISTQSPSQN
T_theileri_Tth.46.1080/1-427    --------------------------------------------
T_theileri_Tth.251.1000/1-344   --------------------------------------------
T_theileri_Tth.27.1390/1-380    --------------------------------------------
T_theileri_Tth.12.1020/1-102    --------------------------------------------
T_theileri_Tth.12.1830/1-342    --------------------------------------------
T_theileri_Tth.11.3140/1-251    --------------------------------------------
T_theileri_Tth.286.1000/1-493   --------------------------------------------
T_theileri_Tth.61.1060/1-159    --------------------------------------------
T_theileri_Tth.10.2860/1-651    GNDQNSS-------------------------------------
T_theileri_Tth.11.2120/1-102    --------------------------------------------
T_theileri_Tth.132.1030/1-666   --------------------------------------------
T_theileri_Tth.101.1090/1-119   --------------------------------------------
T_theileri_Tth.23.2140/1-546    --------------------------------------------
T_theileri_Tth.10.2510/1-395    --------------------------------------------
T_theileri_Tth.107.1030/1-864   --------------------------------------------
T_theileri_Tth.31.1060/1-887    THVAFESIAAS-EQTSSVKEEGKKNTAENIEQVQETITESPSHN
T_theileri_Tth.132.1020/1-714   -RRGPQR-------------------------------------
T_theileri_Tth.136.1020/1-209   --------------------------------------------
T_theileri_Tth.129.1020/1-290   --------------------------------------------
T_theileri_Tth.71.1080/1-526    --------------------------------------------
T_theileri_Tth.36.2120/1-796    --------------------------------------------
T_theileri_Tth.63.1240/1-819    ENENNETVVAPGGDG--------------------------ATT
T_theileri_Tth.11.3250/1-309    --------------------------------------------
T_theileri_Tth.11.3240/1-592    --------------------------------------------
T_theileri_Tth.12.1000/1-649    --------------------------------------------
T_theileri_Tth.83.1080/1-228    -------------------------------VDEVHNTPNDGST
T_theileri_Tth.85.1030/1-137    --------------------------------------------
T_theileri_Tth.71.1030/1-204    --------------------------------------------
T_theileri_Tth.97.1060/1-928    SDQQNEKQTSPRAQDQHQASNETENKVQSPNGKQVESAPVAPNG
T_theileri_Tth.7.4630/1-775     --------------------------------------------
T_theileri_Tth.24.1450/1-463    --------------------------------------------
T_theileri_Tth.6.1080/1-569     --------------------------------------------
T_theileri_Tth.14.1400/1-348    --------------------------------------------
T_theileri_Tth.21.1050/1-407    --------------------------------------------
T_theileri_Tth.144.1030/1-653   --------------------------------------------
T_theileri_Tth.269.1000/1-185   --------------------------------------------
T_theileri_Tth.144.1020/1-412   ALQQPVD-----PPAENNDKQNEIQTSPELKSPTGEQEKNSSPA
T_theileri_Tth.151.1020/1-515   --------------------------------------------
T_theileri_Tth.11.3450/1-561    --------------------------------------------
T_theileri_Tth.166.1000/1-479   --------------------------------------------
T_theileri_Tth.54.1350/1-777    --------------------------------------------
T_theileri_Tth.10.2520/1-446    PTEKSVEGDKTTSPKAEDQRQTPTQT-PQTKVPLPNEVKTPT--
```

```
                                   1680      1690      1700      1710
T_theileri_Tth.43.1980/1-746    VPNQTENKVQSPTEAKAQTDDSTDVQNNTHGEESTQRNRHDTAT
T_theileri_Tth.32.2230/1-717    ------------SSQTPIVPQAHESNPVRGDVDGSTGKEDAAVE
T_theileri_Tth.11.3610/1-173    --------------------------------------------
T_theileri_Tth.46.1030/1-525    --------------------------------------------
T_theileri_Tth.36.1960/1-517    --------------------------------------------
T_theileri_Tth.165.1020/1-245   --------------------------------------------
T_theileri_Tth.46.1120/1-413    --------------------------------------------
T_theileri_Tth.24.2780/1-672    ------------------------------------TSLQGDQG
T_theileri_Tth.8.4720/1-600     --------------------------------------------
T_theileri_Tth.165.1030/1-156   --------------------------------------------
T_theileri_Tth.6.5040/1-514     --------------------------------------------
T_theileri_Tth.31.1000/1-129    --------------------------------------------
T_theileri_Tth.11.3650/1-197    --------------------------------------------
T_theileri_Tth.19.2150/1-142    --------------------------------------------
T_theileri_Tth.4.4260/1-444     ------------------PSPLPHPTPEPTMETVGAPHVERELT
T_theileri_Tth.70.1040/1-472    GNDEGINTTVDTTHSP---NRPATDNEK-EETIVNTDNNTT---
T_theileri_Tth.46.1080/1-427    --------------------------------------------
T_theileri_Tth.251.1000/1-344   --------------------------------------------
T_theileri_Tth.27.1390/1-380    --------------------------------------------
T_theileri_Tth.12.1020/1-102    --------------------------------------------
T_theileri_Tth.12.1830/1-342    --------------------------------------------
T_theileri_Tth.11.3140/1-251    --------------------------------------------
T_theileri_Tth.286.1000/1-493   --------------------------------------------
T_theileri_Tth.61.1060/1-159    --------------------------------------------
T_theileri_Tth.10.2860/1-651    ---------------SASPSSVSSS-------------------
T_theileri_Tth.11.2120/1-102    --------------------------------------------
T_theileri_Tth.132.1030/1-666   --------------------------------------------
T_theileri_Tth.101.1090/1-119   --------------------------------------------
T_theileri_Tth.23.2140/1-546    --------------------------------------------
T_theileri_Tth.10.2510/1-395    --------------------------------------------
T_theileri_Tth.107.1030/1-864   -----------------------------------LSDGTQPVT
T_theileri_Tth.31.1060/1-887    GNGEGINTTVDTTHSP---NSPATDNEK-NETIVNTHNNTS---
T_theileri_Tth.132.1020/1-714   ---------------RRRDTDAT----------TS---------
T_theileri_Tth.136.1020/1-209   --------------------------------------------
T_theileri_Tth.129.1020/1-290   --------------------------------------------
T_theileri_Tth.71.1080/1-526    --------------------------------------------
T_theileri_Tth.36.2120/1-796    ------------------------------------------ST
T_theileri_Tth.63.1240/1-819    EEESEKHPTDKPTGPSDESNENV----------SN---------
T_theileri_Tth.11.3250/1-309    --------------------------------------------
T_theileri_Tth.11.3240/1-592    --------------------------------------------
T_theileri_Tth.12.1000/1-649    -------------------------TASDLPPSTAVSDNQSLPT
T_theileri_Tth.83.1080/1-228    GKDGTLTVNSGTSSGNRQQESTRTETNSGERNNNQNNDSSMTPQ
T_theileri_Tth.85.1030/1-137    --------------------------------------------
T_theileri_Tth.71.1030/1-204    --------------------------------------------
T_theileri_Tth.97.1060/1-928    EVHSSNSSTKASDDSTDVQNNTHGEESTPR--------------
T_theileri_Tth.7.4630/1-775     --------------------------------------------
T_theileri_Tth.24.1450/1-463    ------------------------------------TSVQGDQG
T_theileri_Tth.6.1080/1-569     --------------------------------------------
T_theileri_Tth.14.1400/1-348    --------------------------------------------
T_theileri_Tth.21.1050/1-407    --------------------------------------------
T_theileri_Tth.144.1030/1-653   ---------------------------IEDPPHSSVQRSQGPQT
T_theileri_Tth.269.1000/1-185   --------------------------------------------
T_theileri_Tth.144.1020/1-412   GPNDKVHSSNSSTTASEDSIKVQSDAREKESEQKKPRDNADDVA
T_theileri_Tth.151.1020/1-515   ------------------------------------TSLQKDQE
T_theileri_Tth.11.3450/1-561    --------------------------------------------
T_theileri_Tth.166.1000/1-479   --------------------------------------------
T_theileri_Tth.54.1350/1-777    ------------------------------------------AT
T_theileri_Tth.10.2520/1-446    -----VESTSQLENSSTSPTEVQSDAEREELPSRNRRGTDDD--
```

```
                                   1720      1730      1740      1750
T_theileri_Tth.43.1980/1-746     TFSSAANSNGNES-TIQSPHKA-----------NNGVLNGTNLT
T_theileri_Tth.32.2230/1-717     ESKLSNNVSGSRDNSAKPQGSDQEKNVLPPSEVPGSSDITHSDN
T_theileri_Tth.11.3610/1-173     --------------------------------------------
T_theileri_Tth.46.1030/1-525     --------------------------------------------
T_theileri_Tth.36.1960/1-517     ---------------------------HGNDGRS----------
T_theileri_Tth.165.1020/1-245    ------------------------------------KGKDLPKS
T_theileri_Tth.46.1120/1-413     --------------------------------------------
T_theileri_Tth.24.2780/1-672     NNGTTTNASSRRDT-TGVTSSTTSNNTQAAAAAGTTLLNGKDNA
T_theileri_Tth.8.4720/1-600      -------PNDNED-G-----------------------------
T_theileri_Tth.165.1030/1-156    --------------------------------------------
T_theileri_Tth.6.5040/1-514      --------------------------------------------
T_theileri_Tth.31.1000/1-129     --------------------------------------------
T_theileri_Tth.11.3650/1-197     --------------------------------------------
T_theileri_Tth.19.2150/1-142     --------------------------------------------
T_theileri_Tth.4.4260/1-444      SSNPNVPTDSSGH-HTSERDGLFGSTTH----------------
T_theileri_Tth.70.1040/1-472     --------------------------------NTTTSSIPENVN
T_theileri_Tth.46.1080/1-427     ---------------------------------------GEESK
T_theileri_Tth.251.1000/1-344    --------------------------------------------
T_theileri_Tth.27.1390/1-380     --------------------------------------------
T_theileri_Tth.12.1020/1-102     --------------------------------------------
T_theileri_Tth.12.1830/1-342     --------------------------------------------
T_theileri_Tth.11.3140/1-251     --------------------------------------------
T_theileri_Tth.286.1000/1-493    --------------------------------------------
T_theileri_Tth.61.1060/1-159     --------------------------------------------
T_theileri_Tth.10.2860/1-651     --GSHDTAAAAAA-TSQSSSN----------------PNGTNLT
T_theileri_Tth.11.2120/1-102     --------------------------------------------
T_theileri_Tth.132.1030/1-666    -----------ED-AIQSPNTV---------------LNGTNLT
T_theileri_Tth.101.1090/1-119    --------------------------------------------
T_theileri_Tth.23.2140/1-546     --------------------------------------------
T_theileri_Tth.10.2510/1-395     --------------------------------------------
T_theileri_Tth.107.1030/1-864    ETTGTAKP---------SESAGTSNN-AGNEGETAPVVKNEAST
T_theileri_Tth.31.1060/1-887     --------------------------------NTTSSSIPENVN
T_theileri_Tth.132.1020/1-714    --QSAANPNGNED-AIQSPNTV---------------LNGTTIA
T_theileri_Tth.136.1020/1-209    --------------------------------------------
T_theileri_Tth.129.1020/1-290    --------------------------------------------
T_theileri_Tth.71.1080/1-526     --------------------------------------------
T_theileri_Tth.36.2120/1-796     EATETAKPPESAG-AKPSESAGTSNN-SVNQEVAAPVVQNGAGT
T_theileri_Tth.63.1240/1-819     --SSSGVPPNTED-LPQGESPQ---------------ETETPSN
T_theileri_Tth.11.3250/1-309     ---------------------------VAVGNSSSSSSSSSSAE
T_theileri_Tth.11.3240/1-592     ------------------------------------LINGKDNA
T_theileri_Tth.12.1000/1-649     NTEDSDHRTTTTPTTAEPNNAHTTGSSTTDSTTPTATVNDNGKT
T_theileri_Tth.83.1080/1-228     ESRNTNTSDGERG-NTENNSSTTTTQESGNTNTSDGERGNTQVN
T_theileri_Tth.85.1030/1-137     --------------------------------------------
T_theileri_Tth.71.1030/1-204     --------------------------------------------
T_theileri_Tth.97.1060/1-928     --NRRDTADAATSPTTSNPNTVDAANEPSSHTANNAALNGTSLT
T_theileri_Tth.7.4630/1-775      ----------------GQGTAVGTGTTTTNTGTTTPTNQNPGTP
T_theileri_Tth.24.1450/1-463     NNGTTINSRSRRDT-TAVTSSTTSNNTQAAAAAGVTSPSGEENA
T_theileri_Tth.6.1080/1-569      --------------------------------------------
T_theileri_Tth.14.1400/1-348     --------------------------------------------
T_theileri_Tth.21.1050/1-407     --------------------------------------------
T_theileri_Tth.144.1030/1-653    STVNNAQQTTSTTTTADANRQR-------SSTTPTATVNKNGKT
T_theileri_Tth.269.1000/1-185    --------------------------------------------
T_theileri_Tth.144.1020/1-412    TAPSTSNPNSNGN-TMQSTNTINGANEPSTHTANNTVLNGTNLN
T_theileri_Tth.151.1020/1-515    NKE-----------------------------------------
T_theileri_Tth.11.3450/1-561     --------------------------------------------
T_theileri_Tth.166.1000/1-479    --------------------------------------------
T_theileri_Tth.54.1350/1-777     EATGTPKP---------SE------N-AGNQGVSVPVVQNDAS-
T_theileri_Tth.10.2520/1-446     -ASSTSNP-----------NTVDAANESSSHTANNGTLNGTNFT
```

```
                                       1770      1780      1790      1800
T_theileri_Tth.43.1980/1-746   EDQIKEETLKHTNVTGMLG-----------PDSSIMV-SYMAPL
T_theileri_Tth.32.2230/1-717   RYNFTDVSGDSTHVQQGVSSIHMGIVGVEGTDKSLRT-PYTASL
T_theileri_Tth.11.3610/1-173   --------------------------------------------
T_theileri_Tth.46.1030/1-525   --------------------------------------------
T_theileri_Tth.36.1960/1-517   -------------------------------DGSVTA-AILV--
T_theileri_Tth.165.1020/1-245  FDEYMLPPGMEHLTEEERARYREHFLANGGLDSSSTTTLMTSML
T_theileri_Tth.46.1120/1-413   --------------------------------------------
T_theileri_Tth.24.2780/1-672   NTNDPSKYPGVVNQAQINNMQNSTGILTELGKDDALSAVCSLPI
T_theileri_Tth.8.4720/1-600    ------------------A-----------TDNSIAL-SFFEPL
T_theileri_Tth.165.1030/1-156  --------------------------------------------
T_theileri_Tth.6.5040/1-514    --------------------------------------------
T_theileri_Tth.31.1000/1-129   --------------------------------------------
T_theileri_Tth.11.3650/1-197   --------------------------------------------
T_theileri_Tth.19.2150/1-142   --------------------------------------------
T_theileri_Tth.4.4260/1-444    --------------------------------------------
T_theileri_Tth.70.1040/1-472   ASV-APATLTGPNKTQMGQVINQATAIVIAGADSSIATSYQIPL
T_theileri_Tth.46.1080/1-427   VANNST-SDHTVVENITCTVGNCDQHTAGEGADKSIVSSYFGVV
T_theileri_Tth.251.1000/1-344  --------------------------------------------
T_theileri_Tth.27.1390/1-380   --------------------------------------------
T_theileri_Tth.12.1020/1-102   --------------------------------------------
T_theileri_Tth.12.1830/1-342   --------------------------------------------
T_theileri_Tth.11.3140/1-251   --------------------------------DGCATAAPIALS
T_theileri_Tth.286.1000/1-493  --------------------------------------------
T_theileri_Tth.61.1060/1-159   --------------------------------------------
T_theileri_Tth.10.2860/1-651   EGQMKE-KPNHTN--DARR-----------PDSSIMVISYMVPL
T_theileri_Tth.11.2120/1-102   --------------------------------------------
T_theileri_Tth.132.1030/1-666  ESQVEEES-KNANGTDVLG-----------TDSSTVT-SYMAPL
T_theileri_Tth.101.1090/1-119  --------------------------------------------
T_theileri_Tth.23.2140/1-546   -------------------------------GDKTVTDTLFLPF
T_theileri_Tth.10.2510/1-395   --------------------------------------------
T_theileri_Tth.107.1030/1-864  TTESKPSETKEEGITSFLNGQNNNILAGKGIDGSFKA-SVFASV
T_theileri_Tth.31.1060/1-887   ASA-VPATLTELNNTQMGQVINQATTIAIVGADSSIAASYQIPL
T_theileri_Tth.132.1020/1-714  ERQIKEETLKQTNVTVMFG-----------TDGSTVS-SYMVPL
T_theileri_Tth.136.1020/1-209  --------------------------------------------
T_theileri_Tth.129.1020/1-290  --------------------------------------------
T_theileri_Tth.71.1080/1-526   --------------------------------------------
T_theileri_Tth.36.2120/1-796   TTESKPSETKEENNTSSVSGQNNNAAAKKGTDGSFKA-SAFASV
T_theileri_Tth.63.1240/1-819   EVESSKPESPDNNGTTNNGNGQNDHNTGKDADNSITL-SFFEPL
T_theileri_Tth.11.3250/1-309   VQESVVQEQVMGETVTALVSHNQSDIAHRVGMDGSSAAHSLLHA
T_theileri_Tth.11.3240/1-592   NTNDPSKYPGVVNQAQINNMQNSTGILTELAKDNAFSAVYSFPI
T_theileri_Tth.12.1000/1-649   TGSSTTDSTTPQVSVDQKNNTMNIEIVSWPGKDQSQATK-----
T_theileri_Tth.83.1080/1-228   GSPITTQDDKNTNTNSRVRRNANEDGSSIPIQDSRNTNXYTYFT
T_theileri_Tth.85.1030/1-137   --------------------------------------------
T_theileri_Tth.71.1030/1-204   --------------------------------------------
T_theileri_Tth.97.1060/1-928   EDQMKEETLKHPNVMGALG-----------PDSSIMV-SYMAPL
T_theileri_Tth.7.4630/1-775    NSNANSATATGNTAAIDNNVTIQISHTDGVAAPAAALFGTFVCL
T_theileri_Tth.24.1450/1-463   NTNALSQYPG----------------------------------
T_theileri_Tth.6.1080/1-569    --------------------------------------------
T_theileri_Tth.14.1400/1-348   --------------------------------------------
T_theileri_Tth.21.1050/1-407   --------------------------------------------
T_theileri_Tth.144.1030/1-653  TRSS-TESTTTQGSVKQEDTNKNSKKASNRRKDNSKATKVYSPL
T_theileri_Tth.269.1000/1-185  --------------------------------------------
T_theileri_Tth.144.1020/1-412  EDQMK-ETLNHTNVMNVMG-----------PDSSIM-VSYMAPL
T_theileri_Tth.151.1020/1-515  --------------------------------------------
T_theileri_Tth.11.3450/1-561   --------------------------------------------
T_theileri_Tth.166.1000/1-479  --------------------------------------------
T_theileri_Tth.54.1350/1-777   TTKSKPSETKEENITSSVSGQNNNAAAKKGTDGSFKA-SVFASV
T_theileri_Tth.10.2520/1-446   EDQIKEETLKHTNVMGALG-----------PDSSIMFTSYMAPL
```

```
                                1810      1820      1830      1840
T_theileri_Tth.43.1980/1-746    ALLVCVVGFMMVP-------------------------------
T_theileri_Tth.32.2230/1-717    ALLVCVLAVVLVP-------------------------------
T_theileri_Tth.11.3610/1-173    --------------------------------------------
T_theileri_Tth.46.1030/1-525    --------------------------------------------
T_theileri_Tth.36.1960/1-517    --------------------------------------------
T_theileri_Tth.165.1020/1-245   ALLCLAAAVLVTP-------------------------------
T_theileri_Tth.46.1120/1-413    --------------------------------------------
T_theileri_Tth.24.2780/1-672    MFVTMVLAVVLSC-------------------------------
T_theileri_Tth.8.4720/1-600     MLPVCIVAAVVAL-------------------------------
T_theileri_Tth.165.1030/1-156   --------------------------------------------
T_theileri_Tth.6.5040/1-514     --------------------------------------------
T_theileri_Tth.31.1000/1-129    --------------------------------------------
T_theileri_Tth.11.3650/1-197    --------------------------------------------
T_theileri_Tth.19.2150/1-142    --------------------------------------------
T_theileri_Tth.4.4260/1-444     ---------------IDNDTKNNLATNGKGPAAHVPNNSDTITL
T_theileri_Tth.70.1040/1-472    LLIVSALVALASP-------------------------------
T_theileri_Tth.46.1080/1-427    LFLLFIAAVMV---------------------------------
T_theileri_Tth.251.1000/1-344   --------------------------------------------
T_theileri_Tth.27.1390/1-380    --------------------------------------------
T_theileri_Tth.12.1020/1-102    --------------------------------------------
T_theileri_Tth.12.1830/1-342    --------------------------------------------
T_theileri_Tth.11.3140/1-251    AIALMVITLMLLP-------------------------------
T_theileri_Tth.286.1000/1-493   --------------------------------------------
T_theileri_Tth.61.1060/1-159    --------------------------------------------
T_theileri_Tth.10.2860/1-651    ALLMCVVGFVMVP-------------------------------
T_theileri_Tth.11.2120/1-102    --------------------------------------------
T_theileri_Tth.132.1030/1-666   ALLVCVLLVL----------------------------------
T_theileri_Tth.101.1090/1-119   --------------------------------------------
T_theileri_Tth.23.2140/1-546    ISLVAAMVILLIS-------------------------------
T_theileri_Tth.10.2510/1-395    --------------------------------------------
T_theileri_Tth.107.1030/1-864   MFVFLTLSMIXXXXTTKLIGQWQSR-------------------
T_theileri_Tth.31.1060/1-887    LLLFSALVALASP-------------------------------
T_theileri_Tth.132.1020/1-714   ALLVCVVGFVIVP-------------------------------
T_theileri_Tth.136.1020/1-209   --------------------------------------------
T_theileri_Tth.129.1020/1-290   --------------------------------------------
T_theileri_Tth.71.1080/1-526    --------------------------------------------
T_theileri_Tth.36.2120/1-796    MFVFLSLSMIMAP-------------------------------
T_theileri_Tth.63.1240/1-819    MLLVCVVAAVVAL-------------------------------
T_theileri_Tth.11.3250/1-309    LLVAVTLVVVVSL-------------------------------
T_theileri_Tth.11.3240/1-592    MIVTMVLAVVLSC-------------------------------
T_theileri_Tth.12.1000/1-649    --------------------------------------------
T_theileri_Tth.83.1080/1-228    IFGRRCINR-----------------------------------
T_theileri_Tth.85.1030/1-137    --------------------------------------------
T_theileri_Tth.71.1030/1-204    --------------------------------------------
T_theileri_Tth.97.1060/1-928    ALLVCVVGFVMVP-------------------------------
T_theileri_Tth.7.4630/1-775     LLITTMVAVS----------------------------------
T_theileri_Tth.24.1450/1-463    --------------------------------------------
T_theileri_Tth.6.1080/1-569     --------------------------------------------
T_theileri_Tth.14.1400/1-348    --------------------------------------------
T_theileri_Tth.21.1050/1-407    --------------------------------------------
T_theileri_Tth.144.1030/1-653   VLLVLLVSALLISF------------------------------
T_theileri_Tth.269.1000/1-185   --------------------------------------------
T_theileri_Tth.144.1020/1-412   ALLVCVVGFVMVP-------------------------------
T_theileri_Tth.151.1020/1-515   --------------------------------------------
T_theileri_Tth.11.3450/1-561    --------------------------------------------
T_theileri_Tth.166.1000/1-479   --------------------------------------------
T_theileri_Tth.54.1350/1-777    MFVFLTLSMIMLP-------------------------------
T_theileri_Tth.10.2520/1-446    ALLVGVVGFVMVP-------------------------------
```

```
                              1850      1860      1870
T_theileri_Tth.43.1980/1-746   --------------------------------
T_theileri_Tth.32.2230/1-717   --------------------------------
T_theileri_Tth.11.3610/1-173   --------------------------------
T_theileri_Tth.46.1030/1-525   --------------------------------
T_theileri_Tth.36.1960/1-517   --------------------------------
T_theileri_Tth.165.1020/1-245  --------------------------------
T_theileri_Tth.46.1120/1-413   --------------------------------
T_theileri_Tth.24.2780/1-672   --------------------------------
T_theileri_Tth.8.4720/1-600    --------------------------------
T_theileri_Tth.165.1030/1-156  --------------------------------
T_theileri_Tth.6.5040/1-514    --------------------------------
T_theileri_Tth.31.1000/1-129   --------------------------------
T_theileri_Tth.11.3650/1-197   --------------------------------
T_theileri_Tth.19.2150/1-142   --------------------------------
T_theileri_Tth.4.4260/1-444    NIGIGDGTVTPAGHAPLLLLIFASVVAVVVPL
T_theileri_Tth.70.1040/1-472   --------------------------------
T_theileri_Tth.46.1080/1-427   --------------------------------
T_theileri_Tth.251.1000/1-344  --------------------------------
T_theileri_Tth.27.1390/1-380   --------------------------------
T_theileri_Tth.12.1020/1-102   --------------------------------
T_theileri_Tth.12.1830/1-342   --------------------------------
T_theileri_Tth.11.3140/1-251   --------------------------------
T_theileri_Tth.286.1000/1-493  --------------------------------
T_theileri_Tth.61.1060/1-159   --------------------------------
T_theileri_Tth.10.2860/1-651   --------------------------------
T_theileri_Tth.11.2120/1-102   --------------------------------
T_theileri_Tth.132.1030/1-666  --------------------------------
T_theileri_Tth.101.1090/1-119  --------------------------------
T_theileri_Tth.23.2140/1-546   --------------------------------
T_theileri_Tth.10.2510/1-395   --------------------------------
T_theileri_Tth.107.1030/1-864  --------------------------------
T_theileri_Tth.31.1060/1-887   --------------------------------
T_theileri_Tth.132.1020/1-714  --------------------------------
T_theileri_Tth.136.1020/1-209  --------------------------------
T_theileri_Tth.129.1020/1-290  --------------------------------
T_theileri_Tth.71.1080/1-526   --------------------------------
T_theileri_Tth.36.2120/1-796   --------------------------------
T_theileri_Tth.63.1240/1-819   --------------------------------
T_theileri_Tth.11.3250/1-309   --------------------------------
T_theileri_Tth.11.3240/1-592   --------------------------------
T_theileri_Tth.12.1000/1-649   --------------------------------
T_theileri_Tth.83.1080/1-228   --------------------------------
T_theileri_Tth.85.1030/1-137   --------------------------------
T_theileri_Tth.71.1030/1-204   --------------------------------
T_theileri_Tth.97.1060/1-928   --------------------------------
T_theileri_Tth.7.4630/1-775    --------------------------------
T_theileri_Tth.24.1450/1-463   --------------------------------
T_theileri_Tth.6.1080/1-569    --------------------------------
T_theileri_Tth.14.1400/1-348   --------------------------------
T_theileri_Tth.21.1050/1-407   --------------------------------
T_theileri_Tth.144.1030/1-653  --------------------------------
T_theileri_Tth.269.1000/1-185  --------------------------------
T_theileri_Tth.144.1020/1-412  --------------------------------
T_theileri_Tth.151.1020/1-515  --------------------------------
T_theileri_Tth.11.3450/1-561   ---------------------LASMLWLWFFY
T_theileri_Tth.166.1000/1-479  --------------------------------
T_theileri_Tth.54.1350/1-777   --------------------------------
T_theileri_Tth.10.2520/1-446   --------------------------------
```

10 20 30 40

T_theileri_Tth.11.2260/1-625 --
T_theileri_Tth.70.1030/1-768 --
T_theileri_Tth.107.1040/1-828 --
T_theileri_Tth.70.1110/1-944 --
T_theileri_Tth.26.2530/1-142 --
T_theileri_Tth.46.1090/1-145 --
T_theileri_Tth.24.1770/1-211 --
T_theileri_Tth.13.2480/1-341 --
T_theileri_Tth.6.5070/1-728 --
T_theileri_Tth.2.1490/1-336 --
T_theileri_Tth.136.1010/1-335 --
T_theileri_Tth.46.1100/1-749 --
T_theileri_Tth.83.1100/1-183 --
T_theileri_Tth.85.1040/1-640 --
T_theileri_Tth.36.2070/1-818 --
T_theileri_Tth.17.3250/1-783 --
T_theileri_Tth.38.2000/1-661 --
T_theileri_Tth.15.3170/1-626 --
T_theileri_Tth.36.2060/1-770 --
T_theileri_Tth.61.1040/1-155 --
T_theileri_Tth.121.1030/1-597 --
T_theileri_Tth.10.2550/1-638 --
T_theileri_Tth.54.1320/1-753 --
T_theileri_Tth.101.1070/1-104 --
T_theileri_Tth.2.4010/1-502 --
T_theileri_Tth.11.2100/1-425 --
T_theileri_Tth.25.1730/1-222 --
T_theileri_Tth.1.6340/1-550 --
T_theileri_Tth.2.4020/1-205 --
T_theileri_Tth.6.5050/1-642 --
T_theileri_Tth.121.1040/1-345 --
T_theileri_Tth.87.1070/1-161 --
T_theileri_Tth.10.2540/1-581 --
T_theileri_Tth.70.1130/1-603 --
T_theileri_Tth.125.1020/1-613 --
T_theileri_Tth.107.1020/1-785 --
T_theileri_Tth.24.1760/1-439 --
T_theileri_Tth.27.1380/1-325 --
T_theileri_Tth.44.1550/1-167 --
T_theileri_Tth.61.1080/1-163 --
T_theileri_Tth.85.1060/1-407 --
T_theileri_Tth.11.3490/1-239 --

Conservation

Quality

Consensus

```
                                   50        60        70        80
T_theileri_Tth.11.2260/1-625    --------------------------------------------
T_theileri_Tth.70.1030/1-768    --------------------------------------------
T_theileri_Tth.107.1040/1-828   --------------------------------------------
T_theileri_Tth.70.1110/1-944    --------------------------------------------
T_theileri_Tth.26.2530/1-142    --------------------------------------------
T_theileri_Tth.46.1090/1-145    --------------------------------------------
T_theileri_Tth.24.1770/1-211    --------------------------------------------
T_theileri_Tth.13.2480/1-341    --------------------------------------------
T_theileri_Tth.6.5070/1-728     --------------------------------------------
T_theileri_Tth.2.1490/1-336     --------------------------------------------
T_theileri_Tth.136.1010/1-335   --------------------------------------------
T_theileri_Tth.46.1100/1-749    --------------------------------------------
T_theileri_Tth.83.1100/1-183    --------------------------------------------
T_theileri_Tth.85.1040/1-640    --------------------------------------------
T_theileri_Tth.36.2070/1-818    --------------------------------------------
T_theileri_Tth.17.3250/1-783    --------------------------------------------
T_theileri_Tth.38.2000/1-661    --------------------------------------------
T_theileri_Tth.15.3170/1-626    --------------------------------------------
T_theileri_Tth.36.2060/1-770    --------------------------------------------
T_theileri_Tth.61.1040/1-155    --------------------------------------------
T_theileri_Tth.121.1030/1-597   --------------------------------------------
T_theileri_Tth.10.2550/1-638    --------------------------------------------
T_theileri_Tth.54.1320/1-753    --------------------------------------------
T_theileri_Tth.101.1070/1-104   --------------------------------------------
T_theileri_Tth.2.4010/1-502     --------------------------------------------
T_theileri_Tth.11.2100/1-425    --------------------------------------------
T_theileri_Tth.25.1730/1-222    --------------------------------------------
T_theileri_Tth.1.6340/1-550     --------------------------------------------
T_theileri_Tth.2.4020/1-205     --------------------------------------------
T_theileri_Tth.6.5050/1-642     --------------------------------------------
T_theileri_Tth.121.1040/1-345   --------------------------------------------
T_theileri_Tth.87.1070/1-161    --------------------------------------------
T_theileri_Tth.10.2540/1-581    --------------------------------------------
T_theileri_Tth.70.1130/1-603    --------------------------------------------
T_theileri_Tth.125.1020/1-613   --------------------------------------------
T_theileri_Tth.107.1020/1-785   --------------------------------------------
T_theileri_Tth.24.1760/1-439    --------------------------------------------
T_theileri_Tth.27.1380/1-325    --------------------------------------------
T_theileri_Tth.44.1550/1-167    --------------------------------------------
T_theileri_Tth.61.1080/1-163    --------------------------------------------
T_theileri_Tth.85.1060/1-407    --------------------------------------------
T_theileri_Tth.11.3490/1-239    --------------------------------------------

Conservation
                                --------------------------------------------

Quality

Consensus
                                -----------------------------MNILPLHNGLCFFAT
```

```
                                      90        100       110       120       130
T_theileri_Tth.11.2260/1-625    --------------------------------------------
T_theileri_Tth.70.1030/1-768    --------------------------------------------
T_theileri_Tth.107.1040/1-828   --------------------------------------------
T_theileri_Tth.70.1110/1-944    --------------------------------------------
T_theileri_Tth.26.2530/1-142    --------------------------------------------
T_theileri_Tth.46.1090/1-145    --------------------------------------------
T_theileri_Tth.24.1770/1-211    --------------------------------------------
T_theileri_Tth.13.2480/1-341    --------------------------------------------
T_theileri_Tth.6.5070/1-728     --------------------------------------------
T_theileri_Tth.2.1490/1-336     --------------------------------------------
T_theileri_Tth.136.1010/1-335   --------------------------------------------
T_theileri_Tth.46.1100/1-749    --------------------------------------------
T_theileri_Tth.83.1100/1-183    --------------------------------------------
T_theileri_Tth.85.1040/1-640    --------------------------------------------
T_theileri_Tth.36.2070/1-818    --------------------------------------------
T_theileri_Tth.17.3250/1-783    --------------------------------------------
T_theileri_Tth.38.2000/1-661    --------------------------------------------
T_theileri_Tth.15.3170/1-626    --------------------------------------------
T_theileri_Tth.36.2060/1-770    --------------------------------------------
T_theileri_Tth.61.1040/1-155    --------------------------------------------
T_theileri_Tth.121.1030/1-597   --------------------------------------------
T_theileri_Tth.10.2550/1-638    --------------------------------------------
T_theileri_Tth.54.1320/1-753    --------------------------------------------
T_theileri_Tth.101.1070/1-104   --------------------------------------------
T_theileri_Tth.2.4010/1-502     --------------------------------------------
T_theileri_Tth.11.2100/1-425    --------------------------------------------
T_theileri_Tth.25.1730/1-222    --------------------------------------------
T_theileri_Tth.1.6340/1-550     --------------------------------------------
T_theileri_Tth.2.4020/1-205     --------------------------------------------
T_theileri_Tth.6.5050/1-642     --------------------------------------------
T_theileri_Tth.121.1040/1-345   --------------------------------------------
T_theileri_Tth.87.1070/1-161    --------------------------------------------
T_theileri_Tth.10.2540/1-581    --------------------------------------------
T_theileri_Tth.70.1130/1-603    --------------------------------------------
T_theileri_Tth.125.1020/1-613   --------------------------------------------
T_theileri_Tth.107.1020/1-785   --------------------------------------------
T_theileri_Tth.24.1760/1-439    --------------------------------------------
T_theileri_Tth.27.1380/1-325    --------------------------------------------
T_theileri_Tth.44.1550/1-167    --------------------------------------------
T_theileri_Tth.61.1080/1-163    --------------------------------------------
T_theileri_Tth.85.1060/1-407    --------------------------------------------
T_theileri_Tth.11.3490/1-239    --------------------------------------------

Conservation
                                --------------------------------------------
Quality

Consensus
                                TVVPFDLDWHVRDC-CPALYCAPHSFFSL---PSLLLVSFST--
```

```
                             140                 150                 160                 170
T_theileri_Tth.11.2260/1-625  -------------MNKTPHSLLFLWRRPLLLLLLLFLCCASVCI
T_theileri_Tth.70.1030/1-768  -----------------------MRRLLCTAL--LLLCCAYGC-
T_theileri_Tth.107.1040/1-828 ------------------MRRLFL----FAPL--LLLHCTLVT-
T_theileri_Tth.70.1110/1-944  -----------------------MRRLLCTAL--LLLCCAYGC-
T_theileri_Tth.26.2530/1-142  --------------------------------------------
T_theileri_Tth.46.1090/1-145  -----------------------MRRLLCTAL--LLLCCAYGCI
T_theileri_Tth.24.1770/1-211  --------------------------------------------
T_theileri_Tth.13.2480/1-341  --------------------------------------------
T_theileri_Tth.6.5070/1-728   ------------------MEKHSMRHLLWTAL--FLLYCSCGC-
T_theileri_Tth.2.1490/1-336   --------------------------------------------
T_theileri_Tth.136.1010/1-335 ------------------MEKHSMRHLLWAAL--FLLYCSCGC-
T_theileri_Tth.46.1100/1-749  -----------------------MRRLLCTAL--LLLCCAYGC-
T_theileri_Tth.83.1100/1-183  --------------------------------------------
T_theileri_Tth.85.1040/1-640  ----------------MPRQMYVPLFLLLL--LFVQLCSTSGIL
T_theileri_Tth.36.2070/1-818  --------------------------------------------
T_theileri_Tth.17.3250/1-783  ------------------MEKHSMRHLLWAVL--FLLYCSCGC-
T_theileri_Tth.38.2000/1-661  ----------------------MMRHPLLQVVLLLFICGTVAGP
T_theileri_Tth.15.3170/1-626  ------------------MEKHSMRHLLWAAL--FLLYCSCGC-
T_theileri_Tth.36.2060/1-770  ------------------MRRLFL----FAPL--LLLYCTLVT-
T_theileri_Tth.61.1040/1-155  --------------------------------------------
T_theileri_Tth.121.1030/1-597 --------------------------------------------
T_theileri_Tth.10.2550/1-638  -----------------------MRHPLLQVVLLLFICGAVAGP
T_theileri_Tth.54.1320/1-753  ------------------MRRLFL----FAPL--LLLYCTLVI-
T_theileri_Tth.101.1070/1-104 --------------------------------------------
T_theileri_Tth.2.4010/1-502   -------------------MKSHVHQLICSALLLFLLCCAYGCA
T_theileri_Tth.11.2100/1-425  --------------------------------------------
T_theileri_Tth.25.1730/1-222  --------------------------------------------
T_theileri_Tth.1.6340/1-550   ----------------MTRQMHVSLFLLLLLFLFVQLCSTSGIL
T_theileri_Tth.2.4020/1-205   --------------------------------------------
T_theileri_Tth.6.5050/1-642   ------------------MEKHSMRHLLWTAL--FLLYYSCGC-
T_theileri_Tth.121.1040/1-345 ------------------MEKHSMRHLLWTAL--FLLYCSCRC-
T_theileri_Tth.87.1070/1-161  ------------------MKKLLEMIISVMLFLLLFLHCVCGLA
T_theileri_Tth.10.2540/1-581  --------------------------------------------
T_theileri_Tth.70.1130/1-603  -----------------------MRRLLCTAL--LLLCCAYGCI
T_theileri_Tth.125.1020/1-613 ----------------MTRQMHVSLFFLLLLFLFVQLCSTSGIL
T_theileri_Tth.107.1020/1-785 ------------------MQRLFL----FAPL--LLLYCTLVT-
T_theileri_Tth.24.1760/1-439  --------------------------------------------
T_theileri_Tth.27.1380/1-325  -----------------------MRCIPHTVV--LLLCCTIACF
T_theileri_Tth.44.1550/1-167  ----------------MPRQMHVSLFLLLL--LFVQLCSTSGIL
T_theileri_Tth.61.1080/1-163  --------------------------------------------
T_theileri_Tth.85.1060/1-407  ----------------MPRQMHVSLFLLLL--LFVQLCSTSGIL
T_theileri_Tth.11.3490/1-239  --------------------------------------------
```

Conservation

Quality

Consensus

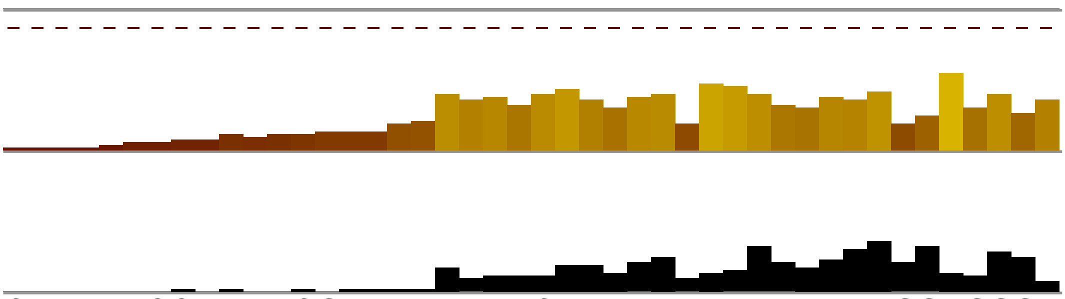

```
SLHYMPQQAM+LSC+RMTMEKHSMRHLLLTALLLLLLCCACGCL
```

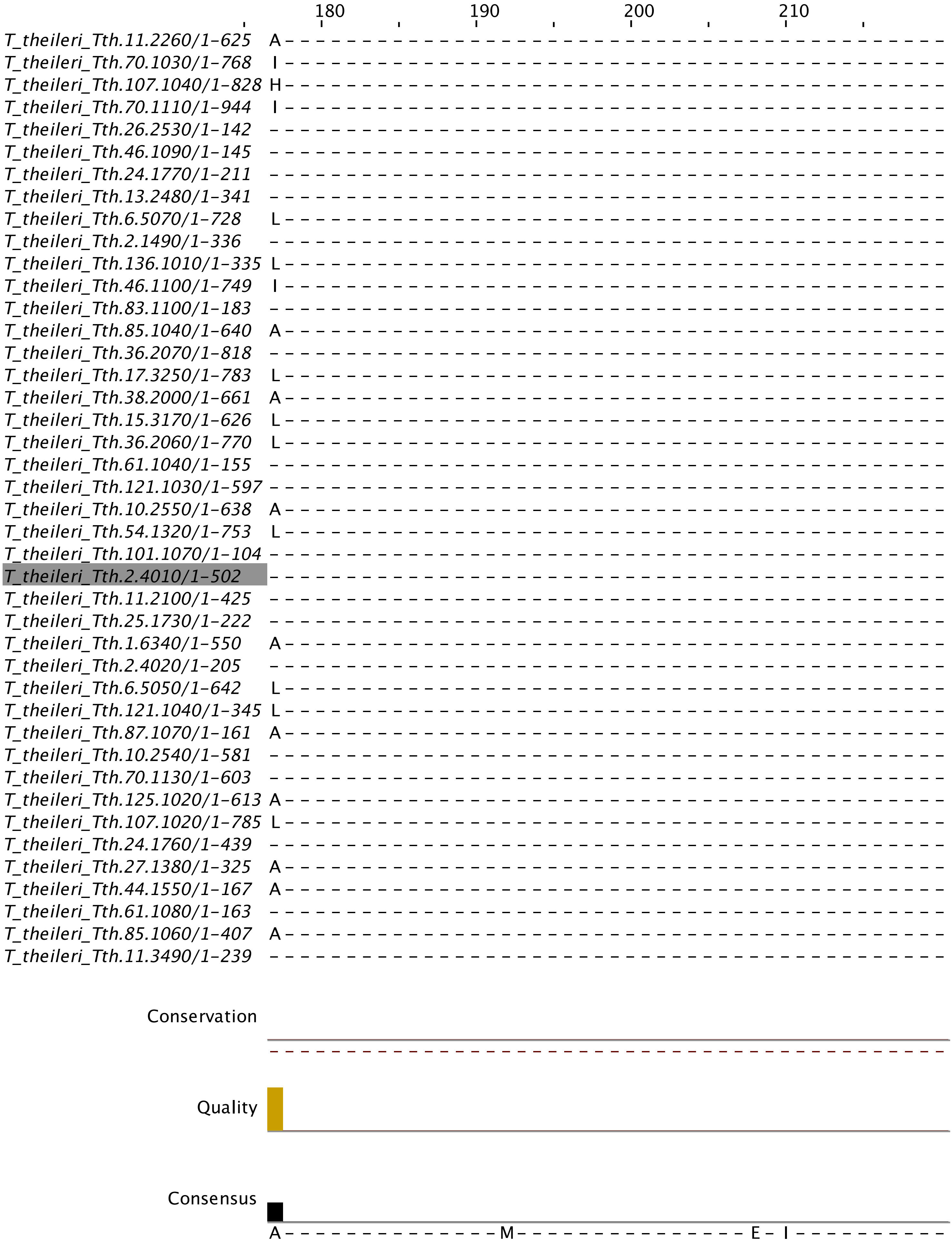
180
190
200
210
T_theileri_Tth.11.2260/1-625 A
T_theileri_Tth.70.1030/1-768 I
T_theileri_Tth.107.1040/1-828 H
T_theileri_Tth.70.1110/1-944 I
T_theileri_Tth.26.2530/1-142
T_theileri_Tth.46.1090/1-145
T_theileri_Tth.24.1770/1-211
T_theileri_Tth.13.2480/1-341
T_theileri_Tth.6.5070/1-728 L
T_theileri_Tth.2.1490/1-336
T_theileri_Tth.136.1010/1-335 L
T_theileri_Tth.46.1100/1-749 I
T_theileri_Tth.83.1100/1-183
T_theileri_Tth.85.1040/1-640 A
T_theileri_Tth.36.2070/1-818
T_theileri_Tth.17.3250/1-783 L
T_theileri_Tth.38.2000/1-661 A
T_theileri_Tth.15.3170/1-626 L
T_theileri_Tth.36.2060/1-770 L
T_theileri_Tth.61.1040/1-155
T_theileri_Tth.121.1030/1-597
T_theileri_Tth.10.2550/1-638 A
T_theileri_Tth.54.1320/1-753 L
T_theileri_Tth.101.1070/1-104
T_theileri_Tth.2.4010/1-502
T_theileri_Tth.11.2100/1-425
T_theileri_Tth.25.1730/1-222
T_theileri_Tth.1.6340/1-550 A
T_theileri_Tth.2.4020/1-205
T_theileri_Tth.6.5050/1-642 L
T_theileri_Tth.121.1040/1-345 L
T_theileri_Tth.87.1070/1-161 A
T_theileri_Tth.10.2540/1-581
T_theileri_Tth.70.1130/1-603
T_theileri_Tth.125.1020/1-613 A
T_theileri_Tth.107.1020/1-785 L
T_theileri_Tth.24.1760/1-439
T_theileri_Tth.27.1380/1-325 A
T_theileri_Tth.44.1550/1-167 A
T_theileri_Tth.61.1080/1-163
T_theileri_Tth.85.1060/1-407 A
T_theileri_Tth.11.3490/1-239
Conservation
Quality
Consensus
A M E I

```
                                        230       240       250       260
T_theileri_Tth.11.2260/1-625    --------------------------------------------
T_theileri_Tth.70.1030/1-768    --------------------------------------------
T_theileri_Tth.107.1040/1-828   --------------------------------------------
T_theileri_Tth.70.1110/1-944    --------------------------------------------
T_theileri_Tth.26.2530/1-142    --------------------------------------------
T_theileri_Tth.46.1090/1-145    --------------------------------------------
T_theileri_Tth.24.1770/1-211    --------------------------------------------
T_theileri_Tth.13.2480/1-341    --------------------------------------------
T_theileri_Tth.6.5070/1-728     --------------------------------------------
T_theileri_Tth.2.1490/1-336     --------------------------------------------
T_theileri_Tth.136.1010/1-335   --------------------------------------------
T_theileri_Tth.46.1100/1-749    --------------------------------------------
T_theileri_Tth.83.1100/1-183    --------------------------------------------
T_theileri_Tth.85.1040/1-640    --------------------------------------------
T_theileri_Tth.36.2070/1-818    --------------------------------------------
T_theileri_Tth.17.3250/1-783    --------------------------------------------
T_theileri_Tth.38.2000/1-661    --------------------------------------------
T_theileri_Tth.15.3170/1-626    --------------------------------------------
T_theileri_Tth.36.2060/1-770    --------------------------------------------
T_theileri_Tth.61.1040/1-155    --------------------------------------------
T_theileri_Tth.121.1030/1-597   --------------------------------------------
T_theileri_Tth.10.2550/1-638    --------------------------------------------
T_theileri_Tth.54.1320/1-753    --------------------------------------------
T_theileri_Tth.101.1070/1-104   --------------------------------------------
T_theileri_Tth.2.4010/1-502     --------------------------------------------
T_theileri_Tth.11.2100/1-425    --------------------------------------------
T_theileri_Tth.25.1730/1-222    --------------------------------------------
T_theileri_Tth.1.6340/1-550     --------------------------------------------
T_theileri_Tth.2.4020/1-205     --------------------------------------------
T_theileri_Tth.6.5050/1-642     --------------------------------------------
T_theileri_Tth.121.1040/1-345   --------------------------------------------
T_theileri_Tth.87.1070/1-161    ----------------------------------------FRDG
T_theileri_Tth.10.2540/1-581    --------------------------------------------
T_theileri_Tth.70.1130/1-603    --------------------------------------------
T_theileri_Tth.125.1020/1-613   --------------------------------------------
T_theileri_Tth.107.1020/1-785   --------------------------------------------
T_theileri_Tth.24.1760/1-439    --------------------------------------------
T_theileri_Tth.27.1380/1-325    --------------------------------------------
T_theileri_Tth.44.1550/1-167    --------------------------------------------
T_theileri_Tth.61.1080/1-163    --------------------------------------------
T_theileri_Tth.85.1060/1-407    --------------------------------------------
T_theileri_Tth.11.3490/1-239    --------------------------------------------

Conservation                    --------------------------------------------

Quality

Consensus                       ----------------------A--N---E------E---LRDD
```

```
                                   270       280       290       300
T_theileri_Tth.11.2260/1-625   -------------QKDGGDAPSTGVVR----ELPRK--------
T_theileri_Tth.70.1030/1-768   ----------------------AAVVQ----PLPQK--------
T_theileri_Tth.107.1040/1-828  ----------------------AVTEH----HRCMFHRVSKLVG
T_theileri_Tth.70.1110/1-944   ----------------------AAVVQ----PLPQT--------
T_theileri_Tth.26.2530/1-142   --------------------------------------------
T_theileri_Tth.46.1090/1-145   ----------------------AAVVQ----PLPQK--------
T_theileri_Tth.24.1770/1-211   ----------------DGGVQSTGVVR----ELPRK--------
T_theileri_Tth.13.2480/1-341   -----------HDRVAEMSSPPVAVVR----EVPKR--------
T_theileri_Tth.6.5070/1-728    ----------------------AAVVQ----QLPQK--------
T_theileri_Tth.2.1490/1-336    -SVGSAQQGEGGSDGDDEISSPASVVR----ELPTK--------
T_theileri_Tth.136.1010/1-335  ----------------------AAVVQ----QLPQK--------
T_theileri_Tth.46.1100/1-749   ----------------------AAVVQ----PLPKR--------
T_theileri_Tth.83.1100/1-183   --------------------------------------------
T_theileri_Tth.85.1040/1-640   -------------------EESTEVVR----ELPVK--------
T_theileri_Tth.36.2070/1-818   ----------------------------------------MLVG
T_theileri_Tth.17.3250/1-783   ----------------------AAVVQ----QLPQK--------
T_theileri_Tth.38.2000/1-661   -------YACNYDEIKKKNSPPVVVVR----ELPKK--------
T_theileri_Tth.15.3170/1-626   ----------------------AAVVQ----QLPQK--------
T_theileri_Tth.36.2060/1-770   ----------------------AVTEQ----HRCMFHKVSTELG
T_theileri_Tth.61.1040/1-155   --------------------------------------------
T_theileri_Tth.121.1030/1-597  --------------------------------------------
T_theileri_Tth.10.2550/1-638   -------YACNYDEIKKKNGSPVAVVR----ELPKS--------
T_theileri_Tth.54.1320/1-753   ----------------------AVTEH----HRCMHDRVVMHVG
T_theileri_Tth.101.1070/1-104  --------------------------------------------
T_theileri_Tth.2.4010/1-502    ----------------------AAVV------------------
T_theileri_Tth.11.2100/1-425   --------------------------------------------
T_theileri_Tth.25.1730/1-222   --------------------------------------------
T_theileri_Tth.1.6340/1-550    -------------------APSAGVVR----EVPLK--------
T_theileri_Tth.2.4020/1-205    --------------------------------------------
T_theileri_Tth.6.5050/1-642    ----------------------AAVVQ----QLPQK--------
T_theileri_Tth.121.1040/1-345  ----------------------AAVVQ----QLPQK--------
T_theileri_Tth.87.1070/1-161   SCTYDTTVWKNMRNKTDYTPLYVSIVR----ELSQS--------
T_theileri_Tth.10.2540/1-581   --------------------------------------------
T_theileri_Tth.70.1130/1-603   ----------------------AAVVQ----PLPQT--------
T_theileri_Tth.125.1020/1-613  ------------------EEQSTEVVQ----EVPKE--------
T_theileri_Tth.107.1020/1-785  ----------------------AVTEH----HRCVFDRVSVKLG
T_theileri_Tth.24.1760/1-439   -KDG--------------GVQSTGVVR----ELPRK--------
T_theileri_Tth.27.1380/1-325   ---SAEHRCIFDKIARKAGTPTTVVVR----EVPHQ--------
T_theileri_Tth.44.1550/1-167   -------------------EESTEVVL----ELPVK--------
T_theileri_Tth.61.1080/1-163   ----DTTVWKNMRNKTIYTPSSVSIVR----ELPQS--------
T_theileri_Tth.85.1060/1-407   -------------------QQLSKVVR----ELPVK--------
T_theileri_Tth.11.3490/1-239   --------------------------------------------
```

Conservation

Quality

Consensus

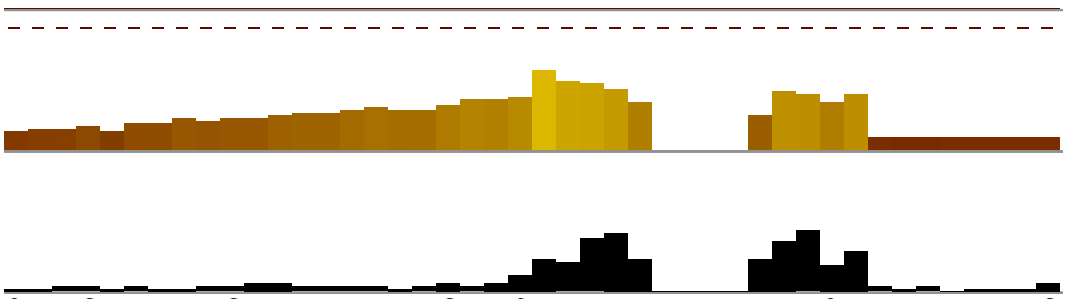

```
QKDGDTT+HCNMDEKTEYGVPSAAVVR----ELPQKDRV+MRVG
```

```
                                    310       320       330       340       350
T_theileri_Tth.11.2260/1-625   -----------------GRSGV-QAYTVAT--------------
T_theileri_Tth.70.1030/1-768   -----------------GQGPW-DTYIVSA-------------T
T_theileri_Tth.107.1040/1-828  AEPKGDDLKE-MTRLNLRRDTR-SSQSM---------RTK-TPA
T_theileri_Tth.70.1110/1-944   -----------------GQGPW-DTYTVAV------------TG
T_theileri_Tth.26.2530/1-142   --------------------------------------------
T_theileri_Tth.46.1090/1-145   -----------------GKELS-DGFIFFP-------------D
T_theileri_Tth.24.1770/1-211   -----------------GQSGV-RAYTVAT--------------
T_theileri_Tth.13.2480/1-341   -----------------GQGAW-QTYTVGV-----TGGQVEAVV
T_theileri_Tth.6.5070/1-728    -----------------GENAL-QAYTVST-------PS-A---
T_theileri_Tth.2.1490/1-336    -----------------DQKLE-EKYTIAT--------------
T_theileri_Tth.136.1010/1-335  -----------------GQNAL-QAYTVSA--------------
T_theileri_Tth.46.1100/1-749   -----------------GQGPW-DTYTVSA------------PD
T_theileri_Tth.83.1100/1-183   --------------------------------------------
T_theileri_Tth.85.1040/1-640   -----------------GENAS-QAYTVYT--------------
T_theileri_Tth.36.2070/1-818   AEFNGDVRGSIGHRGLKRPRHSGSSQTV---------------K
T_theileri_Tth.17.3250/1-783   -----------------GESGL-QAYTVST-------DT-----
T_theileri_Tth.38.2000/1-661   -----------------GEGAW-QTYTVAT--------------
T_theileri_Tth.15.3170/1-626   -----------------GESAL-QAYTVST--------------
T_theileri_Tth.36.2060/1-770   TTYDVSKHETLRTLNS-TSTAG-TLQTT---------RT--NPP
T_theileri_Tth.61.1040/1-155   --------------------------------------------
T_theileri_Tth.121.1030/1-597  --------------------------------------------
T_theileri_Tth.10.2550/1-638   -----------------GQGAW-QAYTVVA--------------
T_theileri_Tth.54.1320/1-753   AYIFSD-----------GSSAAEASPQ----------QEASNPL
T_theileri_Tth.101.1070/1-104  --------------------------------------------
T_theileri_Tth.2.4010/1-502    --------------------------------------------
T_theileri_Tth.11.2100/1-425   --------------------------------------------
T_theileri_Tth.25.1730/1-222   --------------------------------------------
T_theileri_Tth.1.6340/1-550    -----------------GQGAS-QAYTVAT--------------
T_theileri_Tth.2.4020/1-205    --------------------------------------------
T_theileri_Tth.6.5050/1-642    -----------------GQSAL-QAYTVSS--------------
T_theileri_Tth.121.1040/1-345  -----------------GQNAL-QAYTVAT--------------
T_theileri_Tth.87.1070/1-161   -----------------GKSTV-SLYTTTT--ATTTSTTTTTST
T_theileri_Tth.10.2540/1-581   --------------------------------------------
T_theileri_Tth.70.1130/1-603   -----------------GRDLL-DGFIFFP-------------V
T_theileri_Tth.125.1020/1-613  -----------------GQGAS-WGYTIVT--------------
T_theileri_Tth.107.1020/1-785  TSYDLSKQEKA------RDPVSGSLQTW----------STEAPS
T_theileri_Tth.24.1760/1-439   -----------------GQSGV-QAYTVAT--------------
T_theileri_Tth.27.1380/1-325   -----------------RSSGE-QAMAISS--------------
T_theileri_Tth.44.1550/1-167   -----------------GEDAS-QAYTVYT--------------
T_theileri_Tth.61.1080/1-163   -----------------GKSTV-SLYTTTT---------TTTTN
T_theileri_Tth.85.1060/1-407   -----------------GENAS-QAYTVYT--------------
T_theileri_Tth.11.3490/1-239   --------------------------------------------
```

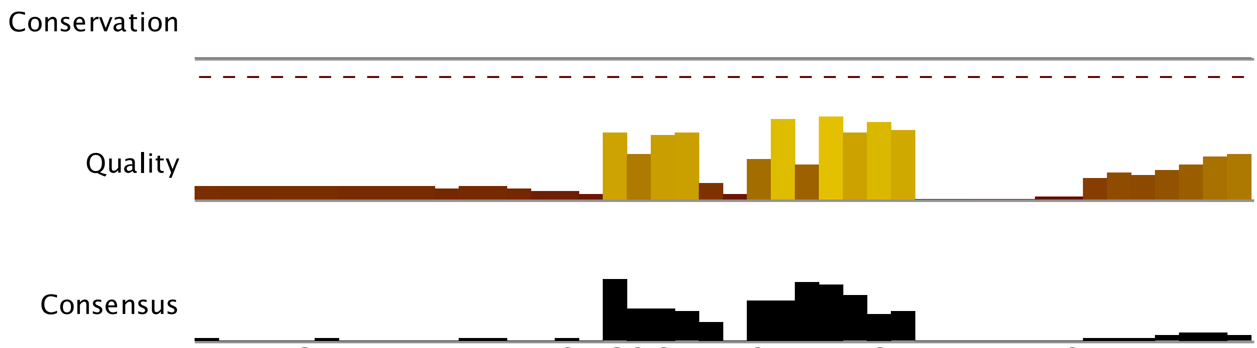


```
                               A++NGDD+EEIMTKLS+GQSAVHQAYTVST-----TSRTTTTPT
```

```
                              360       370       380       390
T_theileri_Tth.11.2260/1-625  ------SKR------QLIRIKTSMEDLKDNTLNNKSLKDSILDG
T_theileri_Tth.70.1030/1-768  DV-PGKVEE----NWQPIRFAVSAKEMNK---------------
T_theileri_Tth.107.1040/1-828 GR-EERIAD----EWKPIRIKLVTDILRK---------------
T_theileri_Tth.70.1110/1-944  EP-DLEKKK----EFKPIRFAVSAKEIDR---------------
T_theileri_Tth.26.2530/1-142  --------------------------------------------
T_theileri_Tth.46.1090/1-145  SR-GEEDEE----RWFPLRIGVFPGGVND---------------
T_theileri_Tth.24.1770/1-211  ------QKKNENEGWELIRIEV-SKDLEN---------------
T_theileri_Tth.13.2480/1-341  TG-KEDENK----DWQPLRINVSYENLKD---------------
T_theileri_Tth.6.5070/1-728   -----PNGE----EWKPIRIAVYTKYVED---------------
T_theileri_Tth.2.1490/1-336   -----KEGK----DWQPIRIHVSAEAVDA---------------
T_theileri_Tth.136.1010/1-335 ------PTE----EWQPIRIAVYTKLLED---------------
T_theileri_Tth.46.1100/1-749  AV-DLEKKK----DFKPIRFAVSAKEVDR---------------
T_theileri_Tth.83.1100/1-183  --------------------------------------------
T_theileri_Tth.85.1040/1-640  ------KGR------KLIRIRTSTRDLQN---------------
T_theileri_Tth.36.2070/1-818  IR-EDP-SE----GWKPIRIKLVSDHLKK---------------
T_theileri_Tth.17.3250/1-783  ----NVRTE----NWQPIRIGVYTKYVED---------------
T_theileri_Tth.38.2000/1-661  ---SEGEND----KWEPLRINVSYENLKE---------------
T_theileri_Tth.15.3170/1-626  ------AAE----QWQPIRIAVYTERVEE---------------
T_theileri_Tth.36.2060/1-770  NR-EEL-PS----GWESIRIKLVSDRLKK---------------
T_theileri_Tth.61.1040/1-155  ---------------MPIRIHVSSEELDR---------------
T_theileri_Tth.121.1030/1-597 --------------------------------------------
T_theileri_Tth.10.2550/1-638  ---SNNDNEIKNKGWEPLRINVSYENLKE---------------
T_theileri_Tth.54.1320/1-753  KR-QEP-TS----GWDPIRIKFFTDDLNT---------------
T_theileri_Tth.101.1070/1-104 --------------------------------------------
T_theileri_Tth.2.4010/1-502   ---NLKSED----GWEPIRIGVYPGGLSV---------------
T_theileri_Tth.11.2100/1-425  --------------------------------------------
T_theileri_Tth.25.1730/1-222  --------------------------------------------
T_theileri_Tth.1.6340/1-550   ------ENR------KFIRIRAFYEDLTN---------------
T_theileri_Tth.2.4020/1-205   --------------------------------------------
T_theileri_Tth.6.5050/1-642   ------DDE----KWKPIRIAVYTERVEE---------------
T_theileri_Tth.121.1040/1-345 ----AKTGE----EWKPIRIGVYTKLVED---------------
T_theileri_Tth.87.1070/1-161  TT-VKSEEE----DWMPIRIHVSSEELDR---------------
T_theileri_Tth.10.2540/1-581  --------------------------------------------
T_theileri_Tth.70.1130/1-603  SQ-AVNGEE----RWYPLRIGVFPDGVND---------------
T_theileri_Tth.125.1020/1-613 ------TNR------TFIRINTSTEDLT----------------
T_theileri_Tth.107.1020/1-785 KR-EEP-QE----GWEPIRIKLVFDNLKM---------------
T_theileri_Tth.24.1760/1-439  ----------QENEGWKLIRIKAFTRDLED---------------
T_theileri_Tth.27.1380/1-325  -------------STWETMRFRVFTEDIEN---------------
T_theileri_Tth.44.1550/1-167  ------KGR------KLIRINTSTRDLQN---------------
T_theileri_Tth.61.1080/1-163  TA-VKSEEE----GWMPIRIHVSSEELDR---------------
T_theileri_Tth.85.1060/1-407  ------RGR------KLIRINTSTRDLQN---------------
T_theileri_Tth.11.3490/1-239  --------------------------------------------
```

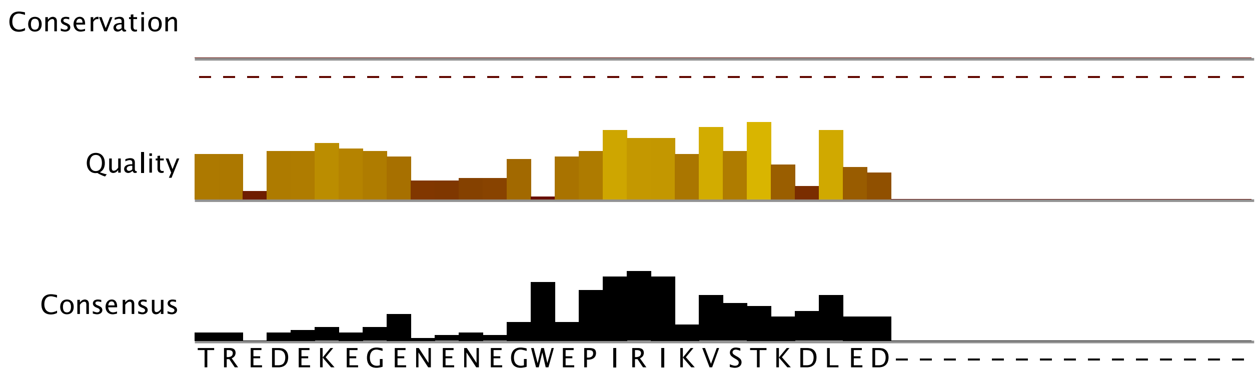

```
                                   400       410       420       430
T_theileri_Tth.11.2260/1-625   DNLESRKNYCTNTNDTAKGMDG----------------------
T_theileri_Tth.70.1030/1-768   -----ALAYCRRYEVL----------DEVGENEEYGFTLENDQ-
T_theileri_Tth.107.1040/1-828  -----DGHYCSDGVTQ--INTSL---------------------
T_theileri_Tth.70.1110/1-944   -----VLAYCNRYEEYALDE--------VQDEILYGFKLDVSK-
T_theileri_Tth.26.2530/1-142   --------------------------------------------
T_theileri_Tth.46.1090/1-145   -----VLNYCNGKKTPTPILGGLSFRES----------------
T_theileri_Tth.24.1770/1-211   -----ASEYCRKVNENNVRD------------------------
T_theileri_Tth.13.2480/1-341   ----EAGLYCKNGNEKKKSFWT----------------------
T_theileri_Tth.6.5070/1-728    -----VMNYCIAEKKEIPSEYNVHQGLKNDLES-----------
T_theileri_Tth.2.1490/1-336    -----AVEKCKDRAAQAREKTFRGDTFMPGHVELVYPD------
T_theileri_Tth.136.1010/1-335  -----IIKYCKNGSDEEDEEEELG--------------------
T_theileri_Tth.46.1100/1-749   -----VLKYCKQWKKDPSAK--------DVYDNEYGFKLEVAN-
T_theileri_Tth.83.1100/1-183   --------------------------------------------
T_theileri_Tth.85.1040/1-640   -----TKKYCSKEGMSIWDFEG----------------------
T_theileri_Tth.36.2070/1-818   -----GEHHCSDKLPS--IKTLL---------------------
T_theileri_Tth.17.3250/1-783   -----AVKYCEENK-DLDFEHGV---------------------
T_theileri_Tth.38.2000/1-661   ------DKYCVKEKQELKHILT----------------------
T_theileri_Tth.15.3170/1-626   -----IMTYC--NEKVMSSKYEPLQDLKNDLEQ-----------
T_theileri_Tth.36.2060/1-770   -----GEHHCSGKGDSKTIKTVL---------------------
T_theileri_Tth.61.1040/1-155   -----SMGRCSSGMTGSLHR------------------------
T_theileri_Tth.121.1030/1-597  --------------------------------------------
T_theileri_Tth.10.2550/1-638   ------DKYCVNKDEKRRDFLD----------------------
T_theileri_Tth.54.1320/1-753   -----SGRFCSTVDES---VFSV---------------------
T_theileri_Tth.101.1070/1-104  --------------------------------------------
T_theileri_Tth.2.4010/1-502    -----WIKSCRRYSTNKVSFGSFATVTY----------------
T_theileri_Tth.11.2100/1-425   --------------------------------------------
T_theileri_Tth.25.1730/1-222   --------------------------------------------
T_theileri_Tth.1.6340/1-550    ------DKYCTAVGELIKDFEG----------------------
T_theileri_Tth.2.4020/1-205    --------------------------------------------
T_theileri_Tth.6.5050/1-642    -----IMNYC--NTKEMSSKYD-IPPLREQLEK-----------
T_theileri_Tth.121.1040/1-345  -----IMEYCNKKVMSSELEYDQEDFE-----------------
T_theileri_Tth.87.1070/1-161   -----VMRRCSSGMTDSSHR------------------------
T_theileri_Tth.10.2540/1-581   --------------------------------------------
T_theileri_Tth.70.1130/1-603   -----VLNYCNGKKTLTPIFGGLSFRES----------------
T_theileri_Tth.125.1020/1-613  -----EDKYCTAVGEVIKDFKE----------------------
T_theileri_Tth.107.1020/1-785  -----AGHDCSENVNKNPIKTLL---------------------
T_theileri_Tth.24.1760/1-439   -----TSRYCTKAGQEVIDFVG----------------------
T_theileri_Tth.27.1380/1-325   -----SSRYCTSASDVRPDFT-----------------------
T_theileri_Tth.44.1550/1-167   -----TKKYCSKEGMSIWDFEG----------------------
T_theileri_Tth.61.1080/1-163   -----AMRRCSSGITDSSHR------------------------
T_theileri_Tth.85.1060/1-407   -----EKKYCSKEGMSIWDFEG----------------------
T_theileri_Tth.11.3490/1-239   --------------------------------------------
```

Conservation

Quality

Consensus

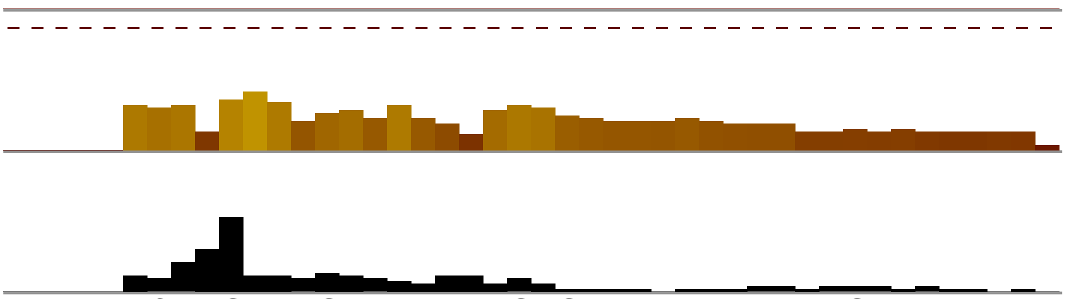

```
-----VSKYCTKKGEERPDFTGLG+RE+DE+EEEYGFELEV+EE
```

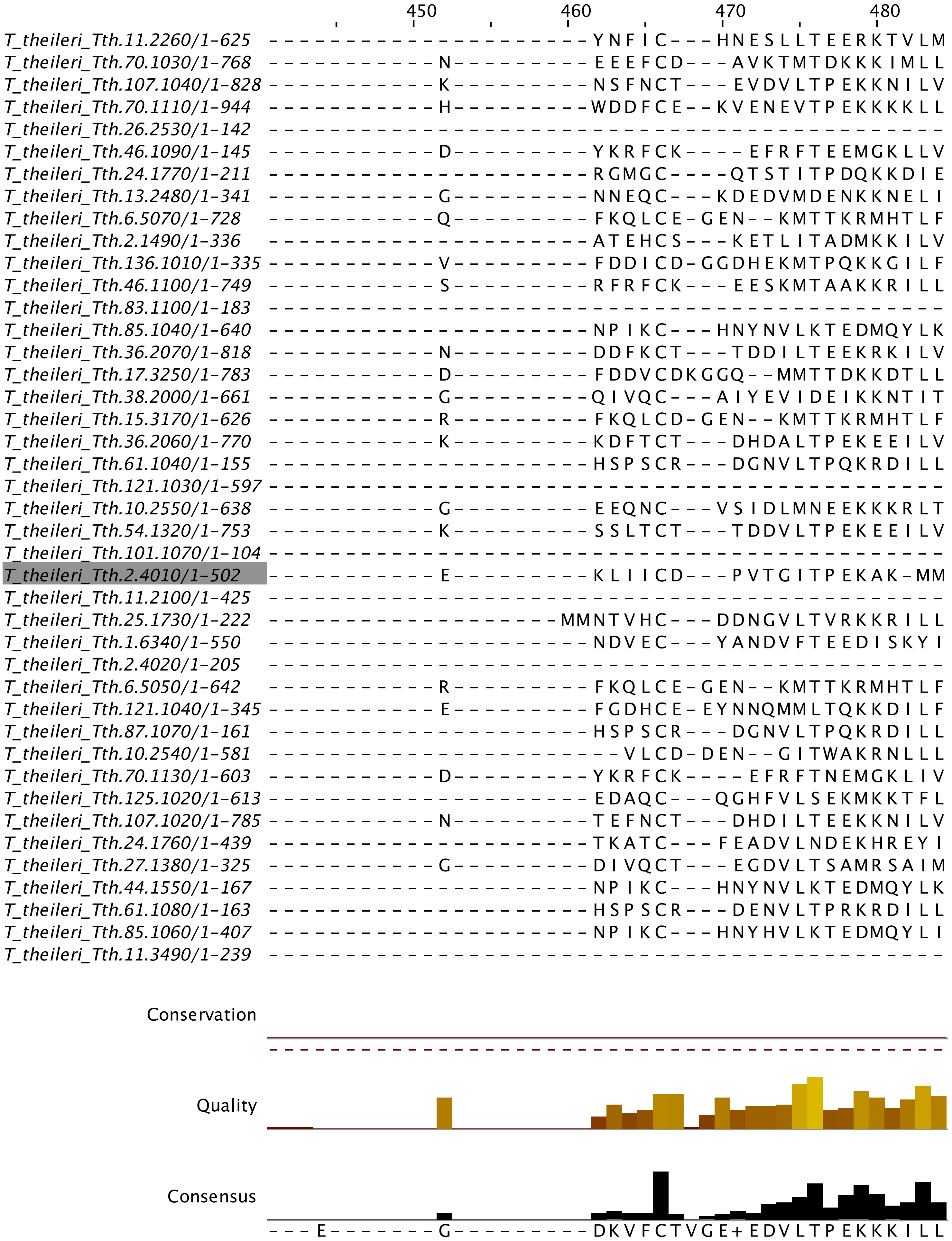
450 460 470 480
T_theileri_Tth.11.2260/1-625 ---------------------YNFIC---HNESLLTEERKTVLM
T_theileri_Tth.70.1030/1-768 -----------N---------EEEFCD---AVKTMTDKKKIMLL
T_theileri_Tth.107.1040/1-828 -----------K---------NSFNCT---EVDVLTPEKKNILV
T_theileri_Tth.70.1110/1-944 -----------H---------WDDFCE--KVENEVTPEKKKKLL
T_theileri_Tth.26.2530/1-142 --
T_theileri_Tth.46.1090/1-145 -----------D---------YKRFCK----EFRFTEEMGKLLV
T_theileri_Tth.24.1770/1-211 ---------------------RGMGC----QTSTITPDQKKDIE
T_theileri_Tth.13.2480/1-341 -----------G---------NNEQC---KDEDVMDENKKNELI
T_theileri_Tth.6.5070/1-728 -----------Q---------FKQLCE-GEN--KMTTKRMHTLF
T_theileri_Tth.2.1490/1-336 ---------------------ATEHCS---KETLITADMKKILV
T_theileri_Tth.136.1010/1-335 -----------V---------FDDICD-GGDHEKMTPQKKGILF
T_theileri_Tth.46.1100/1-749 -----------S---------RFRFCK---EESKMTAAKKRILL
T_theileri_Tth.83.1100/1-183 --
T_theileri_Tth.85.1040/1-640 ---------------------NPIKC---HNYNVLKTEDMQYLK
T_theileri_Tth.36.2070/1-818 -----------N---------DDFKCT---TDDILTEEKRKILV
T_theileri_Tth.17.3250/1-783 -----------D---------FDDVCDKGGQ--MMTTDKKDTLL
T_theileri_Tth.38.2000/1-661 -----------G---------QIVQC---AIYEVIDEIKKNTIT
T_theileri_Tth.15.3170/1-626 -----------R---------FKQLCD-GEN--KMTTKRMHTLF
T_theileri_Tth.36.2060/1-770 -----------K---------KDFTCT---DHDALTPEKEEILV
T_theileri_Tth.61.1040/1-155 ---------------------HSPSCR---DGNVLTPQKRDILL
T_theileri_Tth.121.1030/1-597 --
T_theileri_Tth.10.2550/1-638 -----------G---------EEQNC---VSIDLMNEEKKKRLT
T_theileri_Tth.54.1320/1-753 -----------K---------SSLTCT---TDDVLTPEKEEILV
T_theileri_Tth.101.1070/1-104 --
T_theileri_Tth.2.4010/1-502 -----------E---------KLIICD---PVTGITPEKAK-MM
T_theileri_Tth.11.2100/1-425 --
T_theileri_Tth.25.1730/1-222 -------------------MMNTVHC---DDNGVLTVRKKRILL
T_theileri_Tth.1.6340/1-550 ---------------------NDVEC---YANDVFTEEDISKYI
T_theileri_Tth.2.4020/1-205 --
T_theileri_Tth.6.5050/1-642 -----------R---------FKQLCE-GEN--KMTTKRMHTLF
T_theileri_Tth.121.1040/1-345 -----------E---------FGDHCE-EYNNQMMLTQKKDILF
T_theileri_Tth.87.1070/1-161 ---------------------HSPSCR---DGNVLTPQKRDILL
T_theileri_Tth.10.2540/1-581 -----------------------VLCD-DEN--GITWAKRNLLL
T_theileri_Tth.70.1130/1-603 -----------D---------YKRFCK----EFRFTNEMGKLIV
T_theileri_Tth.125.1020/1-613 ---------------------EDAQC---QGHFVLSEKMKKTFL
T_theileri_Tth.107.1020/1-785 -----------N---------TEFNCT---DHDILTEEKKNILV
T_theileri_Tth.24.1760/1-439 ---------------------TKATC---FEADVLNDEKHREYI
T_theileri_Tth.27.1380/1-325 -----------G---------DIVQCT---EGDVLTSAMRSAIM
T_theileri_Tth.44.1550/1-167 ---------------------NPIKC---HNYNVLKTEDMQYLK
T_theileri_Tth.61.1080/1-163 ---------------------HSPSCR---DENVLTPRKRDILL
T_theileri_Tth.85.1060/1-407 ---------------------NPIKC---HNYHVLKTEDMQYLI
T_theileri_Tth.11.3490/1-239 --
Conservation

Quality
Consensus
---E-------G---------DKVFCTVGE+EDVLTPEKKKILL

```
                                   490       500       510       520
T_theileri_Tth.11.2260/1-625      DKIIPAAVQLHAERLLVDPEGTPLIIPR---------FTEGA--
T_theileri_Tth.70.1030/1-768      RDVLPAALKLHSERLRVEREKLELSLNGK--------EEFSSK-
T_theileri_Tth.107.1040/1-828     NEILPEAIKLHEERLFVKPLNGPIVVPQ---------FSESNGL
T_theileri_Tth.70.1110/1-944      REVLPAALKLHSERLSVKHED-KLALTSENKDS----YPVLPN-
T_theileri_Tth.26.2530/1-142      --------------------------------------------
T_theileri_Tth.46.1090/1-145      EKILPAAVKLHTDRLSVQRSPRKLLVPR----------------
T_theileri_Tth.24.1770/1-211      EKIIPAAKKLHEDRLFVRRGSGKLRVPL---------FEVNND-
T_theileri_Tth.13.2480/1-341      DKILPAAIKLHTDRLLVKRVKTPLKELT---------VENTEI-
T_theileri_Tth.6.5070/1-728       KEVLPQAIKLHRDRLKVKPMKDKLKLWKEDYY-NTE-NIFGKF-
T_theileri_Tth.2.1490/1-336       EKLLPAAIKLHSERLSVHPLQENLVFHKTL-------FEDGAP-
T_theileri_Tth.136.1010/1-335     KDILPKAIKLHTDRLKVKRVGKSLNIRSKEL------YSISEK-
T_theileri_Tth.46.1100/1-749      MDVLPAALKLHSERLSIDLENEKLKLPFNETHP----YPVLPN-
T_theileri_Tth.83.1100/1-183      --------------------------------------------
T_theileri_Tth.85.1040/1-640      NDVLPAAVKLHADRLLVDRVNGSLVVPE---------FEEDSL-
T_theileri_Tth.36.2070/1-818      EEILPEAVKLHKERLFVKPLKGPIVVPE---------FTNKDGL
T_theileri_Tth.17.3250/1-783      NKVLPKAIKLHTDRLKVERVEGSPKKPNVDV------ENIPQK-
T_theileri_Tth.38.2000/1-661      NKILPEAIKLHTDRLLVQRIKTPWKVPD---------MKEHTV-
T_theileri_Tth.15.3170/1-626      KEVLPEAIKLHRDRLNVKRVENNLILWKEDYE-DPY-NIFVKY-
T_theileri_Tth.36.2060/1-770      KKILPEAIKLHEERLFVKRLSGPIIVPQ---------FSNKNGL
T_theileri_Tth.61.1040/1-155      NELLPAAIALHSERLLVVRSRFNLVIMQFISEM-----------
T_theileri_Tth.121.1030/1-597     --------------------------------------------
T_theileri_Tth.10.2550/1-638      DEIVPEAIKLHTDRLLVQRIKTPWKVPN---------LRDHAV-
T_theileri_Tth.54.1320/1-753      KKILPEAIKLHSDRLFVERLRGPIVVPT---------FDSR-SL
T_theileri_Tth.101.1070/1-104     --------------------------------------------
T_theileri_Tth.2.4010/1-502       HKVVVEGSRLHMDRLLVKPVKGNLLIEKHHG------YDFKER-
T_theileri_Tth.11.2100/1-425      ---------------------GVIQIPK---------FKKGEL-
T_theileri_Tth.25.1730/1-222      ERVIPAAIKLHRDRLSVVPVTGTIVVPR----------KNIGY-
T_theileri_Tth.1.6340/1-550       DTIIPAAIKLHEDRLLVDPVDGPLIVPE---------FEENNV-
T_theileri_Tth.2.4020/1-205       --------------------------------------------
T_theileri_Tth.6.5050/1-642       KEVLPEAIKLHRDRLNVKRVENNLILWKAEYN-APD-EPFGKY-
T_theileri_Tth.121.1040/1-345     KEVLPKAIKLHADRLKVKQVEEVLKI-----------TNITED-
T_theileri_Tth.87.1070/1-161      NELLPAAIALHSERL-----------------------------
T_theileri_Tth.10.2540/1-581      REILPAAIKLHRDRLMVQRGDGKFVTNKVSM------GSFKDK-
T_theileri_Tth.70.1130/1-603      EKILPAAVKLHTDRLSVERSSWKLLVPRLFS------QSFFPE-
T_theileri_Tth.125.1020/1-613     DTILPAAIKLHADRLLVDPVEGPLKVPE---------FEEGSV-
T_theileri_Tth.107.1020/1-785     NEILPEAINLHKERLLVKPLKGPIVVPE---------FSDPQGL
T_theileri_Tth.24.1760/1-439      KNIIPAAIKLHRERLRVQPHKGKIIVTQ---------LEGDY--
T_theileri_Tth.27.1380/1-325      DRLIPQAVQMHVDRLHVLRTGGNIVVPTYTGL------------
T_theileri_Tth.44.1550/1-167      NDVLPAAVKLHADRLLVDRVNGSLVVPE---------FEEDSL-
T_theileri_Tth.61.1080/1-163      NELLPAAIALHSERLLVVRSRFNLVIMQFISEM-----------
T_theileri_Tth.85.1060/1-407      NEVLPAAVKLHADRLLVDRVNGSLVVPE---------FEEDSV-
T_theileri_Tth.11.3490/1-239      --------------------------------------------
```

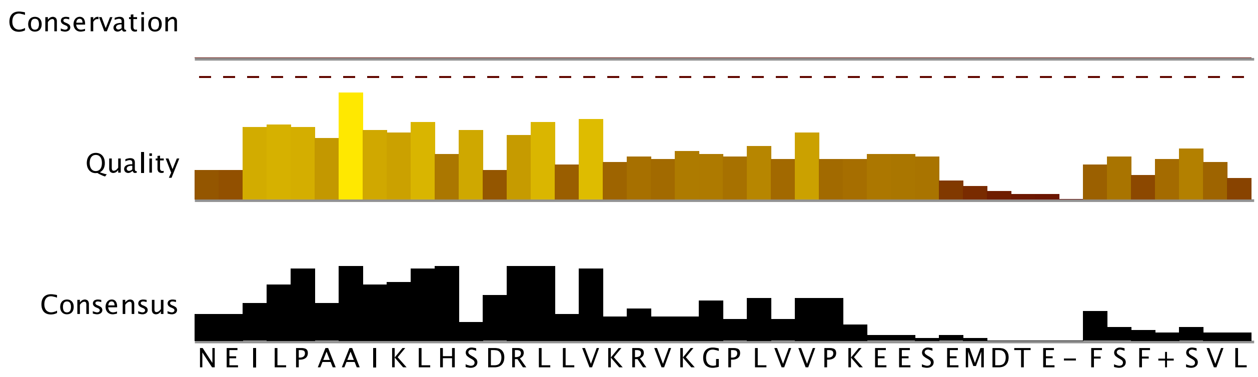

```
                                 530       540       550       560       570
T_theileri_Tth.11.2260/1-625     CSLFTVP-ENHHN-------PGVEKADFVLYVAAKP--------
T_theileri_Tth.70.1030/1-768     CPNVSI-PKEHQQ--------GIPNADFMLYVGLI---DE----
T_theileri_Tth.107.1040/1-828    CSQLIPEEHKHKT-------TGVPEADMVLFVAAEPTT-E----
T_theileri_Tth.70.1110/1-944     CSGVSI-PKEHLS--------GISDADFMLYVGLT---DE----
T_theileri_Tth.26.2530/1-142     --------------------------------------------
T_theileri_Tth.46.1090/1-145     --------------------------------------------
T_theileri_Tth.24.1770/1-211     CKHFTVP-DEHHS-------DGV-DADFVLYVAAVP--------
T_theileri_Tth.13.2480/1-341     CSHFAIP-TVEGSDKPKVDKVKLSESDFLLYVATGSSGNN----
T_theileri_Tth.6.5070/1-728      CQQFFV-PEEHVK-------DGVSDADFLLYVRLS---PY----
T_theileri_Tth.2.1490/1-336      CSFFKP-PEGHHS-------TGVPGADFVLYVTTNKKSNE----
T_theileri_Tth.136.1010/1-335    CSAIKENPLDHKA-------HETLDVDFIIYVALS---TK----
T_theileri_Tth.46.1100/1-749     CTDVSI-PKEHRE--------GIPNADFMLYVGLT---DA----
T_theileri_Tth.83.1100/1-183     --------------------------DFVLYVAAGPGA------
T_theileri_Tth.85.1040/1-640     CSYFTVP-PSHHC-------KGVNNTDMVLYVAAKP--------
T_theileri_Tth.36.2070/1-818     CSKFI--PEAHKT-------NGVDGADMVLFVAATPTT-E----
T_theileri_Tth.17.3250/1-783     CKYFKE-D-SVNL-------QESPDVDFMIFVHLS---AF----
T_theileri_Tth.38.2000/1-661     CSHFTSP-GDRAS-------HGVQDADFLLYVAAGPRDDF----
T_theileri_Tth.15.3170/1-626     CHQLKM-PDEHFK-------DGIPNADFMLYVDLH---PV----
T_theileri_Tth.36.2060/1-770     CFKFI--PEEQKT-------RNFSGADMVLFAAAEPTP-E----
T_theileri_Tth.61.1040/1-155     CYTYVELPAAYES-------VGVVQADFVLFVLAEAVAPFVVIC
T_theileri_Tth.121.1030/1-597    --------------------------------------------
T_theileri_Tth.10.2550/1-638     CSHFTSP-DDHTS-------QDVQDADYLLYVAAGPNKKL----
T_theileri_Tth.54.1320/1-753     CSKFTI-PQEHKT-------TGVDEADMVLYVAAAPTT-D----
T_theileri_Tth.101.1070/1-104    --------------------------------------------
T_theileri_Tth.2.4010/1-502      CPDAFP-RRDHLT-------KGIPKVDFALYLGLTASHEE----
T_theileri_Tth.11.2100/1-425     CAPFTIP-SEHHT-------TGISGADFVLYVAAGPSG------
T_theileri_Tth.25.1730/1-222     CDKFNIP-EEHHT-------TGLKGADLYLYINTVQS-------
T_theileri_Tth.1.6340/1-550      CNNITVP-EDHHS-------KGVNDADMVLYVSARP--------
T_theileri_Tth.2.4020/1-205      --------------------------------------------
T_theileri_Tth.6.5050/1-642      CQQLKM-PENHFN-------EGIPDADFMLYVDLH---PV----
T_theileri_Tth.121.1040/1-345    CVEFNI-PQEHKT-------NGISDVDFMIYVSLS---TA----
T_theileri_Tth.87.1070/1-161     --------------------------------------------
T_theileri_Tth.10.2540/1-581     CTDVVI-GERLEN-------RGFNDADFVLFVGLA---AS----
T_theileri_Tth.70.1130/1-603     CIDAII-PEKHHT-------VGIGNIDFALYVGLSKF-------
T_theileri_Tth.125.1020/1-613    CKNFTVP-PKHRK-------EGVENADMVLYVAARP--------
T_theileri_Tth.107.1020/1-785    CSKFI--PKDHKT-------RSVSGYDMVLYVAATPTT-D----
T_theileri_Tth.24.1760/1-439     CENFTIP-EEHRT-------EGV-DADFVLYVAAIP--------
T_theileri_Tth.27.1380/1-325     CGEFTIP-SDHHT-------VGVSNADMVSYAAAGPTSG-----
T_theileri_Tth.44.1550/1-167     CSYFTVP-PSHHC-------KGVNNTDMVLYVAAK---------
T_theileri_Tth.61.1080/1-163     CYTYVELPAAYES-------VGVVEADFVLFVL-----------
T_theileri_Tth.85.1060/1-407     CSYFTVP-ASHHC-------KGVNNTDMVLYVAAKP--------
T_theileri_Tth.11.3490/1-239     --------------------------------------------
```

Conservation

Quality

Consensus

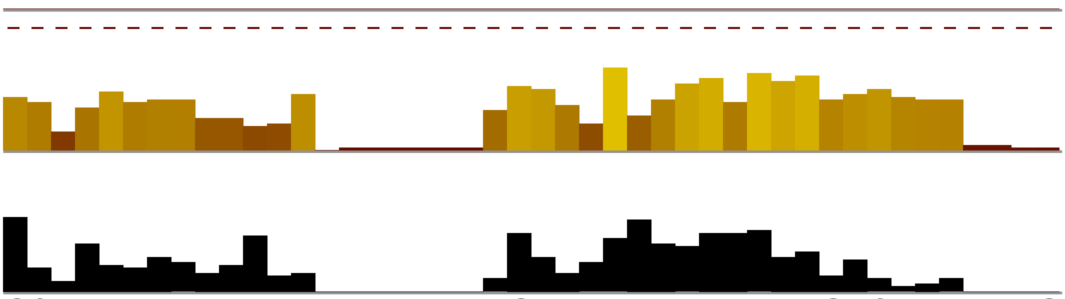

```
CSHFTVPPEEHHTDKLKVDEEGVPDADFVLYVAAGPSKPEVVIC
```

```
                                        580       590       600       610
T_theileri_Tth.11.2260/1-625   ---GGAFGITCADG--RSG------RPIAGGLNFIPYPTA----
T_theileri_Tth.70.1030/1-768   --HQPP---KICSRN-TKG------RPTSALIKFVPKEID----
T_theileri_Tth.107.1040/1-828  --GAFAWAATCATLG-LDG------RPVVGIINYGPRYIV----
T_theileri_Tth.70.1110/1-944   --YVPV---QICSKN-AEG------RPTSALIKFVPKEIA----
T_theileri_Tth.26.2530/1-142   --------------------------------------------
T_theileri_Tth.46.1090/1-145   --------------------------------------------
T_theileri_Tth.24.1770/1-211   ---LPSFGVTCAVEN-STG------RPIVGAVNYMPTPHE----
T_theileri_Tth.13.2480/1-341   --APSSWALTCAVDT-ESK------RPIVGAMRVNPKIIL----
T_theileri_Tth.6.5070/1-728    --GQDS---HICTYE-DYTK----VRPTSARISFVPKEIN----
T_theileri_Tth.2.1490/1-336    -------SVKICAYG-YGR------GPTSAVKNFLPSEIG----
T_theileri_Tth.136.1010/1-335  --PQNV---EICSKD-EEN------RPTSAVISFIPDEIK----
T_theileri_Tth.46.1100/1-749   --YKPV---KICSRN-ENNH-----RPTSALIKFIPREIA----
T_theileri_Tth.83.1100/1-183   -----TFTSICSEES-IEH------RTFSAVMNFEPARIL----
T_theileri_Tth.85.1040/1-640   ---VTPFATICARD--ESR------RPIAAGVNLAFYRKY----
T_theileri_Tth.36.2070/1-818   --ETFVWAATCAKLD-SDG------RPVVGIINYGPRYIV----
T_theileri_Tth.17.3250/1-783   --TEKV---EICTQD-GQK------RPTSAVISFIPEEIE----
T_theileri_Tth.38.2000/1-661   --ASATWAVTCAIDD-QTK------RPVVGAMNIDPSKIH----
T_theileri_Tth.15.3170/1-626   --DRTI---NDCTKD-NKNG----DRPTSARITFVPKEIV----
T_theileri_Tth.36.2060/1-770   --GTFAWAATCATLG-RNG------RPVVGIINYGPRYIV----
T_theileri_Tth.61.1040/1-155   SEADDG-------------------RPTSAAMNFAPADIV----
T_theileri_Tth.121.1030/1-597  --------------------------------------------
T_theileri_Tth.10.2550/1-638   --WSSPWAVTCAIDE-QTK------RPMVGAMNIHPVYTD----
T_theileri_Tth.54.1320/1-753   --GAFAWAATCATLG-PNG------RPVVGIINYGPRYIV----
T_theileri_Tth.101.1070/1-104  --------------------------------------------
T_theileri_Tth.2.4010/1-502    --------HRICSKD-ANG------RPTSGLIKLNPKEFN----
T_theileri_Tth.11.2100/1-425   --GAAAWAGPCARL--KNK------RPIVGVMNYDPMFID----
T_theileri_Tth.25.1730/1-222   --WLLGWASPCAFL--DDG------RPILAAINLSPTYID----
T_theileri_Tth.1.6340/1-550    ---VYDIYVICARN--DDG------RPIAGAINILPFAKK----
T_theileri_Tth.2.4020/1-205    --------------------------------------------
T_theileri_Tth.6.5050/1-642    --YTKV---DDCTKD-NKNG----DRPTSARITFVPKEIV----
T_theileri_Tth.121.1040/1-345  --PEKV---KICSKD-KQN------RPTSAVIKFVPEYIE----
T_theileri_Tth.87.1070/1-161   --------------------------------------------
T_theileri_Tth.10.2540/1-581   --SQDV---KICAED-PIYS----YRPISAYLTFQPKEIE----
T_theileri_Tth.70.1130/1-603   -----MTSAVVCSED-VNK------RPTSGLISFYPKDIV----
T_theileri_Tth.125.1020/1-613  ---ENAFGVPCAYDD-KSG------RPVAGAINVQIYPLK----
T_theileri_Tth.107.1020/1-785  --GAFAWAATCAKLD-SDG------RPVIGIINYGPRYIV----
T_theileri_Tth.24.1760/1-439   --WHWWSGVTCAREK-PSG------RPIVGVMSFIPSLYG----
T_theileri_Tth.27.1380/1-325   --STLAWATTCSAL--IIG------RPVVGVMNFGAQVAT----
T_theileri_Tth.44.1550/1-167   --------------------------------------------
T_theileri_Tth.61.1080/1-163   --------------------------------------------
T_theileri_Tth.85.1060/1-407   ---VTPFATICARD--ESR------RPIAAGVNLAFYRKY----
T_theileri_Tth.11.3490/1-239   --------------------------------------------
```

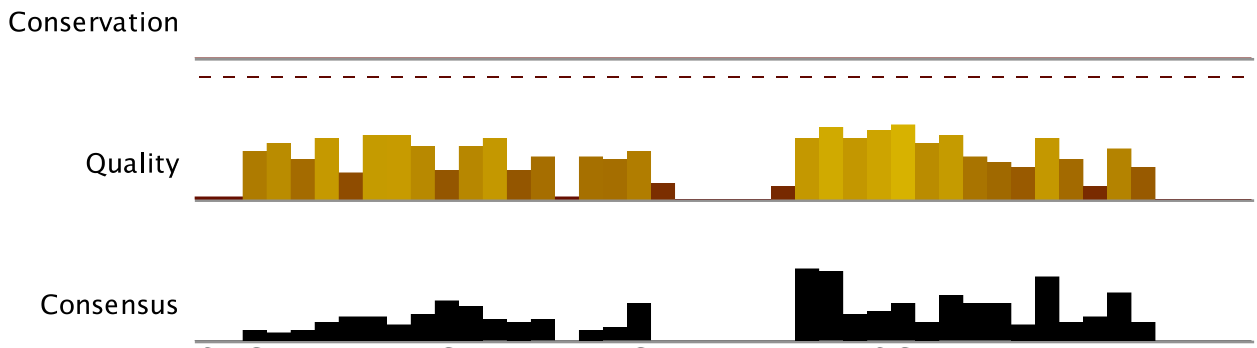


```
                               SEGTFAWAVTCATLDNENG+----ERPTSGAINFVPKEIV----
```

```
                                 620       630       640       650
T_theileri_Tth.11.2260/1-625   --STRPNVRQAAHHIAHALGFDYERMKSL-GM-----ISKIN--
T_theileri_Tth.70.1030/1-768   --DTRYYIRFTAHEVAHALGFEIETMTAQ-KV-----VEEEK--
T_theileri_Tth.107.1040/1-828  --ATPQRVRVAAHEIAHALGFNSQLMERK-GM-----VGEVN--
T_theileri_Tth.70.1110/1-944   --VTRHFIRFTAHEVAHALGFETETMMKH-GV-----IEENP--
T_theileri_Tth.26.2530/1-142   --------------------------------------------
T_theileri_Tth.46.1090/1-145   --------------LVHTVFFS----------------------
T_theileri_Tth.24.1770/1-211   --NVRSSVRRVAHEIAHALGFNLPEMERK-GI-----VTEVE--
T_theileri_Tth.13.2480/1-341   --AGKGIVRLLAHELGHALGFDYERMRER-GM-----ITFRN--
T_theileri_Tth.6.5070/1-728    --ATRHYIRLAARNIAFGLGFGEFV--DA-TA-----IAGNH--
T_theileri_Tth.2.1490/1-336    --ETRRLVRMAAHDIAHALGFDTRRMERI-GM-----IYSATIN
T_theileri_Tth.136.1010/1-335  --ATRKYIRLTAHEIAHGLGFDYEVMETQ-GM-----IEPRK--
T_theileri_Tth.46.1100/1-749   --ATRHYIRFAAHEVAHALGFDIEIMKEN-NVIDVVEVGEER--
T_theileri_Tth.83.1100/1-183   --PTRYAVRIAAHEIAHALGFSYKRMEAL-EM-----TKIIY--
T_theileri_Tth.85.1040/1-640   --GLRYNVRITAHEIAHALGFNYEDMVNA-SM-----VSNIT--
T_theileri_Tth.36.2070/1-818   --ATPQRVRVAAHEIAHALGFNLPEMERK-EL-----VGKGF--
T_theileri_Tth.17.3250/1-783   --ATRKYIRLTAHEIAHGLGFQHKVMEDQ-KM-----VKTRD--
T_theileri_Tth.38.2000/1-661   --FTRENVRFIAHQLAHALGFDYENMMKE--V-----LKNGE--
T_theileri_Tth.15.3170/1-626   --ATRQYIRLAARYIAVGLGFEIFY--MG-GQ-----NVQYS--
T_theileri_Tth.36.2060/1-770   --ATPQRVRVAAHEIAHALGFNFELMKMD-RL-----VGKAD--
T_theileri_Tth.61.1040/1-155   --NTRLFTRIIAHNLAH---------------------------
T_theileri_Tth.121.1030/1-597  --------------------------------------------
T_theileri_Tth.10.2550/1-638   --FTRANVRFIAHQLAHALGFDYKNMMKE-EL-----KDKEE--
T_theileri_Tth.54.1320/1-753   --ATPQRVRVAAHEIAHALGFNFPEMERK-DM-----VQIWE--
T_theileri_Tth.101.1070/1-104  --------------------------------------------
T_theileri_Tth.2.4010/1-502    --NTRHYIRLAAHEIGHALGFTLEEFKEN-DM-----LLEEK--
T_theileri_Tth.11.2100/1-425   --TADKSIRIVAHEIGHALGFGFTEMSMR-NI-----VREAF--
T_theileri_Tth.25.1730/1-222   --ATELYVRAVAHEIAHALGFSIHNFLKR-NM-----TSSLI--
T_theileri_Tth.1.6340/1-550    --SQRYNVRIMAHEIAHVLGFDYNVFKNY-SM-----VDEIN--
T_theileri_Tth.2.4020/1-205    --------------------------------------------
T_theileri_Tth.6.5050/1-642    --ATRQYIRLAARDIAVGLGFEDKY--MV-GQ-----KASN---
T_theileri_Tth.121.1040/1-345  --ATRRYIRVTAHEIAHGLGFDRNVMKTL-GM-----IEPRD--
T_theileri_Tth.87.1070/1-161   --------------------------------------------
T_theileri_Tth.10.2540/1-581   --NARKFVRYAAHKIAHGLGLTYGR--ML-SR-----HKATN--
T_theileri_Tth.70.1130/1-603   --DTRHFIRITAHEIAHALGFELNRMKDL-QM-----IVQRTVN
T_theileri_Tth.125.1020/1-613  --IQRYNVRRVAHEIAHALGFDYKVFEKN-NM-----VAKIE--
T_theileri_Tth.107.1020/1-785  --ATPQRVRVAAHEIAHALGFNFELMKMD-RL-----VGEDD--
T_theileri_Tth.24.1760/1-439   --GLRLNVRRFAHEMAHALGFNIFEMDKK-NM-----LFRVG--
T_theileri_Tth.27.1380/1-325   --DTEHNVRVATHEIAHALGFSIDALMVH-TS-----LVAMP--
T_theileri_Tth.44.1550/1-167   --------------------------------------------
T_theileri_Tth.61.1080/1-163   --------------------------------------------
T_theileri_Tth.85.1060/1-407   --GLRYNVRITAHEIAHALGFNYEDMVNA-SM-----VSNIT--
T_theileri_Tth.11.3490/1-239   --------------------------------------------
```

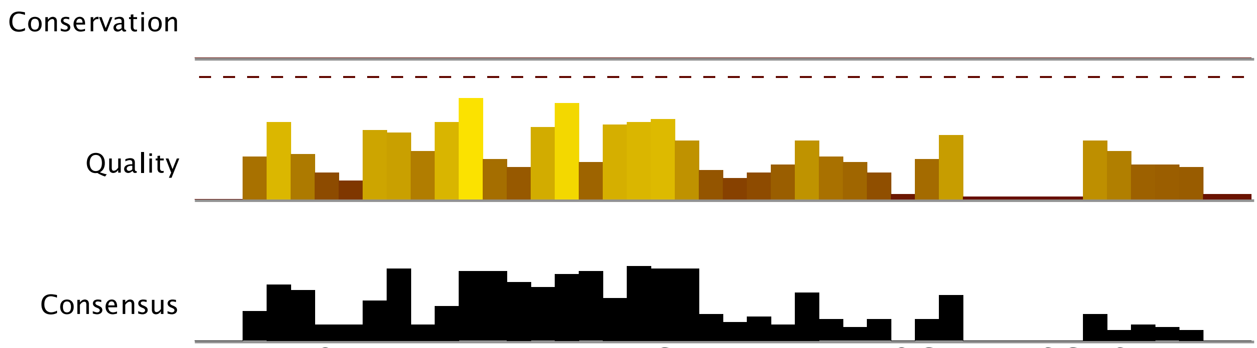


```
--ATRQ+VRVAAHEIAHALGFDYERMKKLQGMIARSGISEVE+N
```

```
                              670       680       690       700
T_theileri_Tth.11.2260/1-625  --VRGV-----------------ERVVVSSNMTKKK-AQEHYDC
T_theileri_Tth.70.1030/1-768  ------------------EK---KVLLAKSKTVLDKVVKEHYGC
T_theileri_Tth.107.1040/1-828 -SLSG-------------RP---FVALVTSDNTRRE-AMEHYKC
T_theileri_Tth.70.1110/1-944  -GG---------------DK---KVPLVKSTTVIKE-MKEHYGC
T_theileri_Tth.26.2530/1-142  --------------------------------------------
T_theileri_Tth.46.1090/1-145  --------------------------------------------
T_theileri_Tth.24.1770/1-211  --LR----------------------------------------
T_theileri_Tth.13.2480/1-341  -IRGE------------------NLTVVNSTNVLMK-AKEHYNC
T_theileri_Tth.6.5070/1-728   -RVFL------NG-G---WK---QVTVVTGGALKEK-MNQQFNC
T_theileri_Tth.2.1490/1-336   NNSDR------------------FVYFVKSPNTIEV-VRKHYAC
T_theileri_Tth.136.1010/1-335 -NGLL------SPGGKYGGE---EFYM-NSSETLEI-LKRLYEC
T_theileri_Tth.46.1100/1-749  -----------------NKK---KVPLLTSTNVLDK-VKAHYGC
T_theileri_Tth.83.1100/1-183  --VTG-------------KK---LRCRVISAVTTNV-SQRHYNC
T_theileri_Tth.85.1040/1-640  -DVRGKNRV--------------TRALVTSNKTLEK-ARKHYGC
T_theileri_Tth.36.2070/1-818  -TLRN-------------KS---NVALVTSDNTRRE-AIDHYNC
T_theileri_Tth.17.3250/1-783  -SGLL------RESRRSGGR---QFYMVISKKTVEM-MEKHYKC
T_theileri_Tth.38.2000/1-661  ------------------KK---TQTVVKSAKVLEE-AREHYGC
T_theileri_Tth.15.3170/1-626  -LLDL------NG-V---QK---YIRVLAGNTVKGK-MNEHYNS
T_theileri_Tth.36.2060/1-770  -SLRD-------------KE---DVFVVSTENTRRE-AMEHYKC
T_theileri_Tth.61.1040/1-155  --------------------------------------------
T_theileri_Tth.121.1030/1-597 ---------------------------MNSSETLEI-LKRLYEC
T_theileri_Tth.10.2550/1-638  ----------------------NIRGVVKSAKVLEK-AKEHYNC
T_theileri_Tth.54.1320/1-753  -NGND-------------QY---VVRRVNSSNVLRE-TKNHYNC
T_theileri_Tth.101.1070/1-104 -------------------------MVVSSELTKEK-VKEHYGC
T_theileri_Tth.2.4010/1-502   -SNTGR-----------------NIMALLTDTVKKK-AQDHYNC
T_theileri_Tth.11.2100/1-425  -KVRG-------------KP---YVTMVTSPLVQQM-VRKHYGC
T_theileri_Tth.25.1730/1-222  -GVRE-------------KP---RVIVINTTRVMDE-AKKHFGC
T_theileri_Tth.1.6340/1-550   -FDDGR-----------------KRMLVKSSKTKEK-VQEHYGC
T_theileri_Tth.2.4020/1-205   --------------------------------------------
T_theileri_Tth.6.5050/1-642   --LQV------NG-V---VK---YSVVLASEIVKKK-MNEHYKC
T_theileri_Tth.121.1040/1-345 -NGLL------STGGASSVK---KFYM-NSSKTLEI-LKKHYNC
T_theileri_Tth.87.1070/1-161  --------------------------------------------
T_theileri_Tth.10.2540/1-581  -GDLF------YG-N---YD---SYRVKVNGRVTVE-AQKHYKC
T_theileri_Tth.70.1130/1-603  GV---------------------NVPEVRSETVVAK-VKEHFGC
T_theileri_Tth.125.1020/1-613 -SNDRK-----------------GRVVVNSNQTKKM-TQKYYNC
T_theileri_Tth.107.1020/1-785 -RLSD-------------KA---DVFVVASKNTRRE-VMDHYNC
T_theileri_Tth.24.1760/1-439  --IRGM-----------------IRTVVRSHVAMGK-VKEHYGC
T_theileri_Tth.27.1380/1-325  -GLRG-------------KN---SVLVVASPRTLEK-TRANFNC
T_theileri_Tth.44.1550/1-167  --------------------------------------------
T_theileri_Tth.61.1080/1-163  --------------------------------------------
T_theileri_Tth.85.1060/1-407  -AVRGK-----------------TRALVTSNKTLEK-ARKHYGC
T_theileri_Tth.11.3490/1-239  --------------------------------------------
```

Conservation

Quality

Consensus

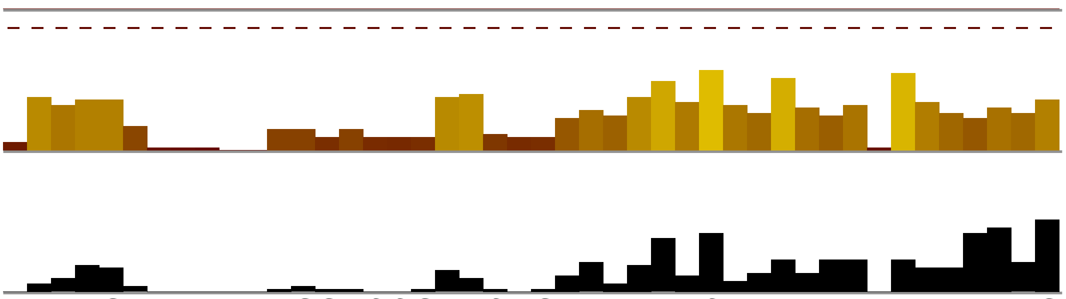

```
N+LRGK-RV--+GG+SSGKKSYGKVYVVNSPKTKEK+AK+HYNC
```

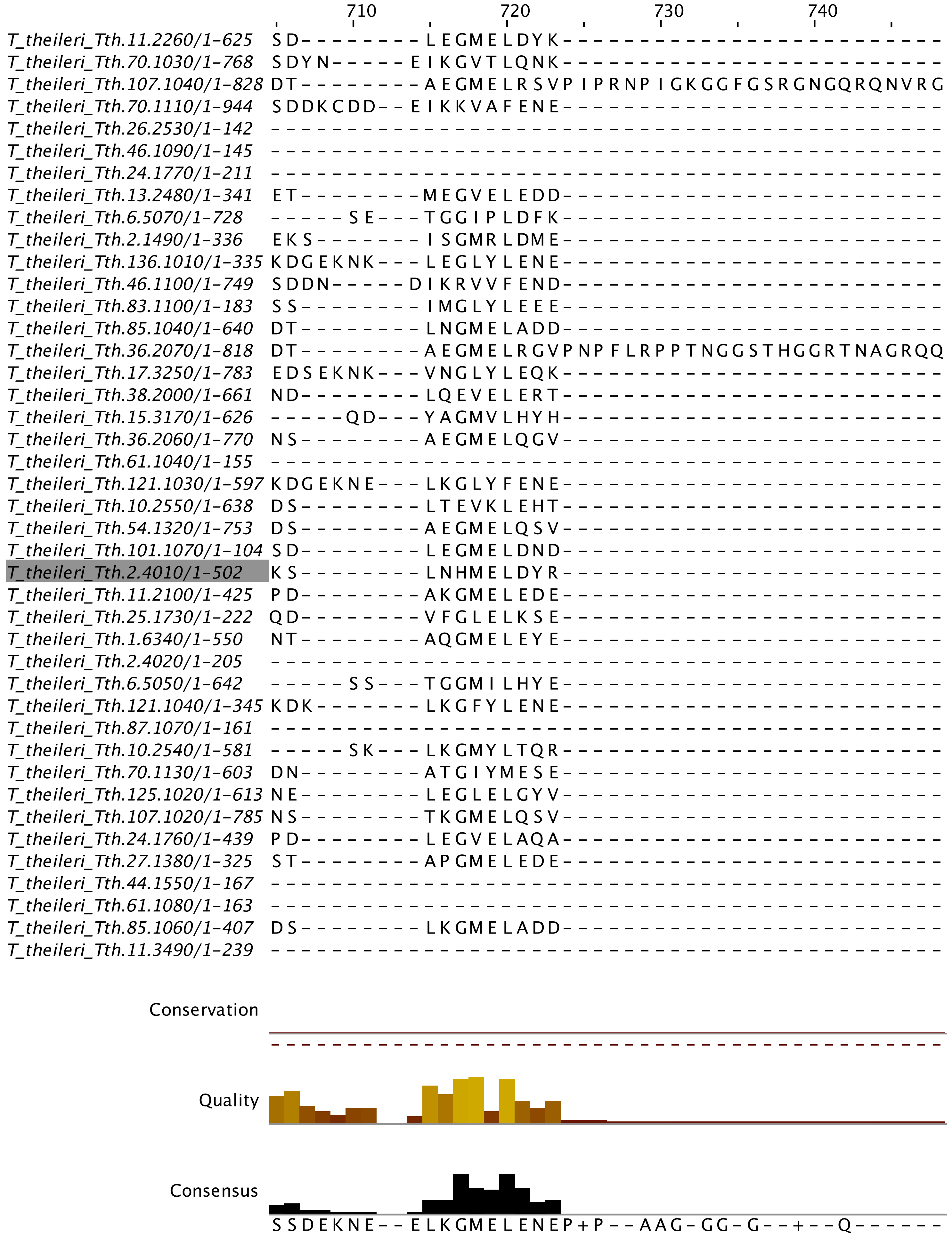
710 720 730 740
T_theileri_Tth.11.2260/1-625 SD--------LEGMELDYK-------------------------
T_theileri_Tth.70.1030/1-768 SDYN-----EIKGVTLQNK-------------------------
T_theileri_Tth.107.1040/1-828 DT--------AEGMELRSVPIPRNPIGKGGFGSRGNGQRQNVRG
T_theileri_Tth.70.1110/1-944 SDDKCDD--EIKKVAFENE-------------------------
T_theileri_Tth.26.2530/1-142 --
T_theileri_Tth.46.1090/1-145 --
T_theileri_Tth.24.1770/1-211 --
T_theileri_Tth.13.2480/1-341 ET--------MEGVELEDD-------------------------
T_theileri_Tth.6.5070/1-728 -----SE---TGGIPLDFK-------------------------
T_theileri_Tth.2.1490/1-336 EKS-------ISGMRLDME-------------------------
T_theileri_Tth.136.1010/1-335 KDGEKNK---LEGLYLENE-------------------------
T_theileri_Tth.46.1100/1-749 SDDN-----DIKRVVFEND-------------------------
T_theileri_Tth.83.1100/1-183 SS--------IMGLYLEEE-------------------------
T_theileri_Tth.85.1040/1-640 DT--------LNGMELADD-------------------------
T_theileri_Tth.36.2070/1-818 DT--------AEGMELRGVPNPFLRPPTNGGSTHGGRTNAGRQQ
T_theileri_Tth.17.3250/1-783 EDSEKNK---VNGLYLEQK-------------------------
T_theileri_Tth.38.2000/1-661 ND--------LQEVELERT-------------------------
T_theileri_Tth.15.3170/1-626 -----QD---YAGMVLHYH-------------------------
T_theileri_Tth.36.2060/1-770 NS--------AEGMELQGV-------------------------
T_theileri_Tth.61.1040/1-155 --
T_theileri_Tth.121.1030/1-597 KDGEKNE---LKGLYFENE-------------------------
T_theileri_Tth.10.2550/1-638 DS--------LTEVKLEHT-------------------------
T_theileri_Tth.54.1320/1-753 DS--------AEGMELQSV-------------------------
T_theileri_Tth.101.1070/1-104 SD--------LEGMELDND-------------------------
T_theileri_Tth.2.4010/1-502 KS--------LNHMELDYR-------------------------
T_theileri_Tth.11.2100/1-425 PD--------AKGMELEDE-------------------------
T_theileri_Tth.25.1730/1-222 QD--------VFGLELKSE-------------------------
T_theileri_Tth.1.6340/1-550 NT--------AQGMELEYE-------------------------
T_theileri_Tth.2.4020/1-205 --
T_theileri_Tth.6.5050/1-642 -----SS---TGGMILHYE-------------------------
T_theileri_Tth.121.1040/1-345 KDK-------LKGFYLENE-------------------------
T_theileri_Tth.87.1070/1-161 --
T_theileri_Tth.10.2540/1-581 -----SK---LKGMYLTQR-------------------------
T_theileri_Tth.70.1130/1-603 DN--------ATGIYMESE-------------------------
T_theileri_Tth.125.1020/1-613 NE--------LEGLELGYV-------------------------
T_theileri_Tth.107.1020/1-785 NS--------TKGMELQSV-------------------------
T_theileri_Tth.24.1760/1-439 PD--------LEGVELAQA-------------------------
T_theileri_Tth.27.1380/1-325 ST--------APGMELEDE-------------------------
T_theileri_Tth.44.1550/1-167 --
T_theileri_Tth.61.1080/1-163 --
T_theileri_Tth.85.1060/1-407 DS--------LKGMELADD-------------------------
T_theileri_Tth.11.3490/1-239 --
Conservation
Quality
Consensus
SSDEKNE--ELKGMELENEP+P--AAG-GG-G--+--Q------

```
                                   750       760       770       780       790
T_theileri_Tth.11.2260/1-625    --------------------------------------------
T_theileri_Tth.70.1030/1-768    --------------------------------------------
T_theileri_Tth.107.1040/1-828   IVTPSGAADATSRN------------------------------
T_theileri_Tth.70.1110/1-944    --------------------------------------------
T_theileri_Tth.26.2530/1-142    --------------------------------------------
T_theileri_Tth.46.1090/1-145    --------------------------------------------
T_theileri_Tth.24.1770/1-211    --------------------------------------------
T_theileri_Tth.13.2480/1-341    --------------------------------------------
T_theileri_Tth.6.5070/1-728     --------------------------------------------
T_theileri_Tth.2.1490/1-336     --------------------------------------------
T_theileri_Tth.136.1010/1-335   --------------------------------------------
T_theileri_Tth.46.1100/1-749    --------------------------------------------
T_theileri_Tth.83.1100/1-183    --------------------------------------------
T_theileri_Tth.85.1040/1-640    --------------------------------------------
T_theileri_Tth.36.2070/1-818    PPHRLRPHSGANANGITGGTMHGRLPNPSLVRQ-----------
T_theileri_Tth.17.3250/1-783    --------------------------------------------
T_theileri_Tth.38.2000/1-661    --------------------------------------------
T_theileri_Tth.15.3170/1-626    --------------------------------------------
T_theileri_Tth.36.2060/1-770    ----------------------------TIVQV-----------
T_theileri_Tth.61.1040/1-155    --------------------------------------------
T_theileri_Tth.121.1030/1-597   --------------------------------------------
T_theileri_Tth.10.2550/1-638    --------------------------------------------
T_theileri_Tth.54.1320/1-753    ----------------------------AFVSV-----------
T_theileri_Tth.101.1070/1-104   --------------------------------------------
T_theileri_Tth.2.4010/1-502     --------------------------------------------
T_theileri_Tth.11.2100/1-425    --------------------------------------------
T_theileri_Tth.25.1730/1-222    --------------------------------------------
T_theileri_Tth.1.6340/1-550     --------------------------------------------
T_theileri_Tth.2.4020/1-205     --------------------------------------------
T_theileri_Tth.6.5050/1-642     --------------------------------------------
T_theileri_Tth.121.1040/1-345   --------------------------------------------
T_theileri_Tth.87.1070/1-161    --------------------------------------------
T_theileri_Tth.10.2540/1-581    --------------------------------------------
T_theileri_Tth.70.1130/1-603    --------------------------------------------
T_theileri_Tth.125.1020/1-613   --------------------------------------------
T_theileri_Tth.107.1020/1-785   ----------------------------SFVKV-----------
T_theileri_Tth.24.1760/1-439    --------------------------------------------
T_theileri_Tth.27.1380/1-325    --------------------------------------------
T_theileri_Tth.44.1550/1-167    --------------------------------------------
T_theileri_Tth.61.1080/1-163    --------------------------------------------
T_theileri_Tth.85.1060/1-407    --------------------------------------------
T_theileri_Tth.11.3490/1-239    --------------------------------------------

Conservation
                                --------------------------------------------
Quality

Consensus
                                --TPSR-A-----N--------G-L--PAFVQVL+-K---G---
```

	800 810 820 830
T_theileri_Tth.11.2260/1-625	--
T_theileri_Tth.70.1030/1-768	--
T_theileri_Tth.107.1040/1-828	-----LKRGRVESGRPPQTQ--------------------RLEP
T_theileri_Tth.70.1110/1-944	--
T_theileri_Tth.26.2530/1-142	--
T_theileri_Tth.46.1090/1-145	--
T_theileri_Tth.24.1770/1-211	--
T_theileri_Tth.13.2480/1-341	--
T_theileri_Tth.6.5070/1-728	--
T_theileri_Tth.2.1490/1-336	--
T_theileri_Tth.136.1010/1-335	--
T_theileri_Tth.46.1100/1-749	--
T_theileri_Tth.83.1100/1-183	--
T_theileri_Tth.85.1040/1-640	--
T_theileri_Tth.36.2070/1-818	-----------------------QRQPRSPSGAARPLRPEGPAV
T_theileri_Tth.17.3250/1-783	--
T_theileri_Tth.38.2000/1-661	--
T_theileri_Tth.15.3170/1-626	--
T_theileri_Tth.36.2060/1-770	-----GQRAK------------------------------AAAI
T_theileri_Tth.61.1040/1-155	--
T_theileri_Tth.121.1030/1-597	--
T_theileri_Tth.10.2550/1-638	--
T_theileri_Tth.54.1320/1-753	------------------------------------PPAKTAAV
T_theileri_Tth.101.1070/1-104	--
T_theileri_Tth.2.4010/1-502	--
T_theileri_Tth.11.2100/1-425	--
T_theileri_Tth.25.1730/1-222	--
T_theileri_Tth.1.6340/1-550	--
T_theileri_Tth.2.4020/1-205	--
T_theileri_Tth.6.5050/1-642	--
T_theileri_Tth.121.1040/1-345	--
T_theileri_Tth.87.1070/1-161	--
T_theileri_Tth.10.2540/1-581	--
T_theileri_Tth.70.1130/1-603	--
T_theileri_Tth.125.1020/1-613	--
T_theileri_Tth.107.1020/1-785	-----RKEPEGADGRARARA------------SSMPAAQLQSTI
T_theileri_Tth.24.1760/1-439	--
T_theileri_Tth.27.1380/1-325	--
T_theileri_Tth.44.1550/1-167	--
T_theileri_Tth.61.1080/1-163	--
T_theileri_Tth.85.1060/1-407	--
T_theileri_Tth.11.3490/1-239	--
Conservation	--
Quality	
Consensus	-G-P-RKR+++E++RRR+R+--GQQGVL+-PGASTPLRPQASAI

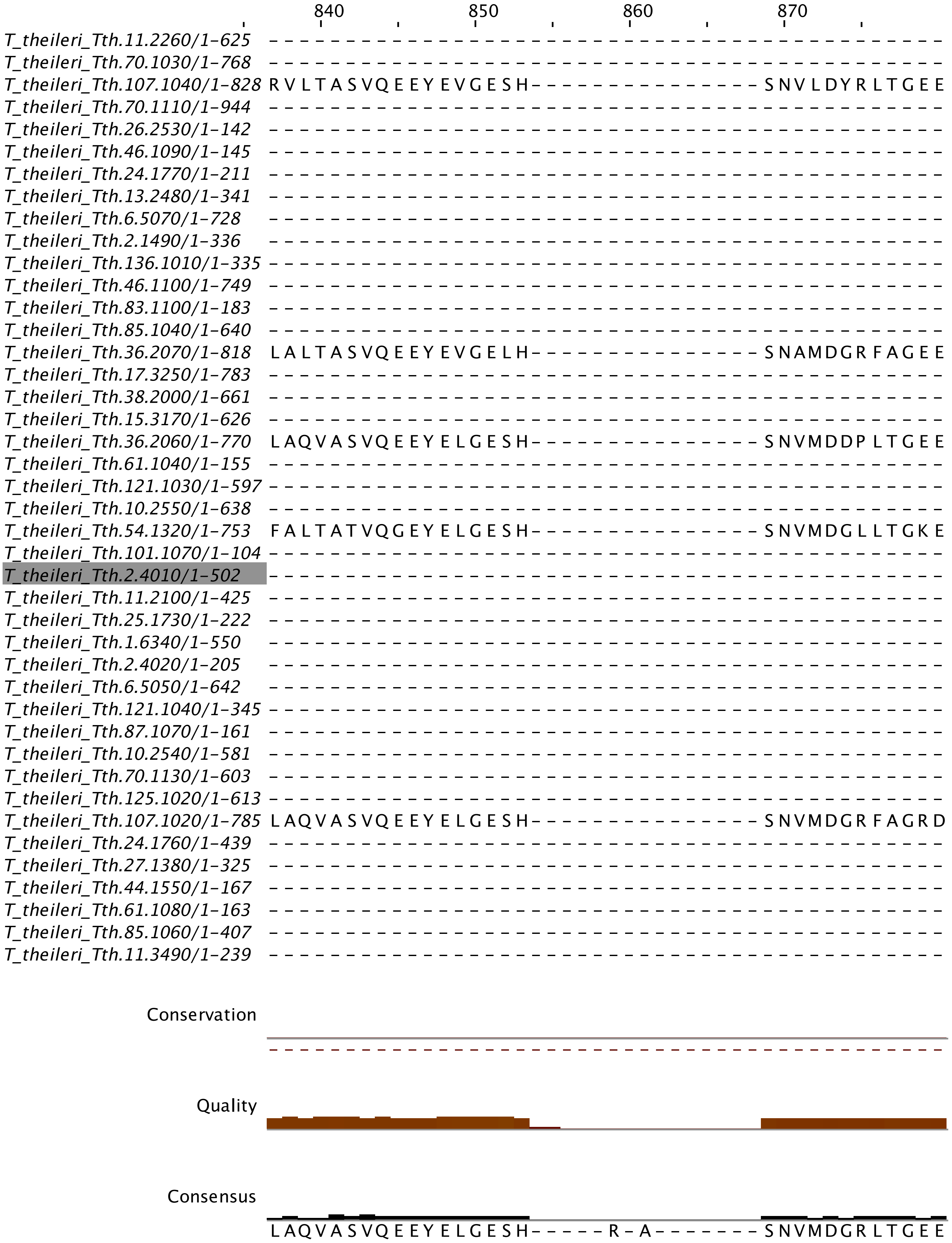

840
850
860
870
T_theileri_Tth.11.2260/1-625
T_theileri_Tth.70.1030/1-768
T_theileri_Tth.107.1040/1-828 RVLTASVQEEYEVGESH---------------SNVLDYRLTGEE
T_theileri_Tth.70.1110/1-944
T_theileri_Tth.26.2530/1-142
T_theileri_Tth.46.1090/1-145
T_theileri_Tth.24.1770/1-211
T_theileri_Tth.13.2480/1-341
T_theileri_Tth.6.5070/1-728
T_theileri_Tth.2.1490/1-336
T_theileri_Tth.136.1010/1-335
T_theileri_Tth.46.1100/1-749
T_theileri_Tth.83.1100/1-183
T_theileri_Tth.85.1040/1-640
T_theileri_Tth.36.2070/1-818 LALTASVQEEYEVGELH---------------SNAMDGRFAGEE
T_theileri_Tth.17.3250/1-783
T_theileri_Tth.38.2000/1-661
T_theileri_Tth.15.3170/1-626
T_theileri_Tth.36.2060/1-770 LAQVASVQEEYELGESH---------------SNVMDDPLTGEE
T_theileri_Tth.61.1040/1-155
T_theileri_Tth.121.1030/1-597
T_theileri_Tth.10.2550/1-638
T_theileri_Tth.54.1320/1-753 FALTATVQGEYELGESH---------------SNVMDGLLTGKE
T_theileri_Tth.101.1070/1-104
T_theileri_Tth.2.4010/1-502
T_theileri_Tth.11.2100/1-425
T_theileri_Tth.25.1730/1-222
T_theileri_Tth.1.6340/1-550
T_theileri_Tth.2.4020/1-205
T_theileri_Tth.6.5050/1-642
T_theileri_Tth.121.1040/1-345
T_theileri_Tth.87.1070/1-161
T_theileri_Tth.10.2540/1-581
T_theileri_Tth.70.1130/1-603
T_theileri_Tth.125.1020/1-613
T_theileri_Tth.107.1020/1-785 LAQVASVQEEYELGESH---------------SNVMDGRFAGRD
T_theileri_Tth.24.1760/1-439
T_theileri_Tth.27.1380/1-325
T_theileri_Tth.44.1550/1-167
T_theileri_Tth.61.1080/1-163
T_theileri_Tth.85.1060/1-407
T_theileri_Tth.11.3490/1-239
Conservation
Quality
Consensus
LAQVASVQEEYELGESH-----R-A-------SNVMDGRLTGEE

```
                                         890       900       910       920
T_theileri_Tth.11.2260/1-625   --------------------------------------------
T_theileri_Tth.70.1030/1-768   --------------------------------------------
T_theileri_Tth.107.1040/1-828  SVGSDVQERRGEE---GSSASKFTENVHHSSNV--------LSK
T_theileri_Tth.70.1110/1-944   --------------------------------------------
T_theileri_Tth.26.2530/1-142   --------------------------------------------
T_theileri_Tth.46.1090/1-145   --------------------------------------------
T_theileri_Tth.24.1770/1-211   --------------------------------------------
T_theileri_Tth.13.2480/1-341   --------------------------------------------
T_theileri_Tth.6.5070/1-728    --------------------------------------------
T_theileri_Tth.2.1490/1-336    --------------------------------------------
T_theileri_Tth.136.1010/1-335  --------------------------------------------
T_theileri_Tth.46.1100/1-749   --------------------------------------------
T_theileri_Tth.83.1100/1-183   --------------------------------------------
T_theileri_Tth.85.1040/1-640   --------------------------------------------
T_theileri_Tth.36.2070/1-818   SVGSVVEERREEE---GSSDAKFTQNVHHSSND--------ISK
T_theileri_Tth.17.3250/1-783   --------------------------------------------
T_theileri_Tth.38.2000/1-661   --------------------------------------------
T_theileri_Tth.15.3170/1-626   --------------------------------------------
T_theileri_Tth.36.2060/1-770   SVGSVVQERRGEE---GSSAAKFTENVHHSSNV--------WLK
T_theileri_Tth.61.1040/1-155   --------------------------------------------
T_theileri_Tth.121.1030/1-597  --------------------------------------------
T_theileri_Tth.10.2550/1-638   --------------------------------------------
T_theileri_Tth.54.1320/1-753   SVDSDVQERREEE---GSNDAKFTQNVHHSSHL--------LSK
T_theileri_Tth.101.1070/1-104  --------------------------------------------
T_theileri_Tth.2.4010/1-502    --------------------------------------------
T_theileri_Tth.11.2100/1-425   --------------------------------------------
T_theileri_Tth.25.1730/1-222   --------------------------------------------
T_theileri_Tth.1.6340/1-550    --------------------------------------------
T_theileri_Tth.2.4020/1-205    --------------------------------------------
T_theileri_Tth.6.5050/1-642    --------------------------------------------
T_theileri_Tth.121.1040/1-345  --------------------------------------------
T_theileri_Tth.87.1070/1-161   --------------------------------------------
T_theileri_Tth.10.2540/1-581   --------------------------------------------
T_theileri_Tth.70.1130/1-603   --------------------------------------------
T_theileri_Tth.125.1020/1-613  --------------------------------------------
T_theileri_Tth.107.1020/1-785  SVGSVVQERPEEE---GSSAAKFTQNVHHSSNV--------FST
T_theileri_Tth.24.1760/1-439   --------------------------------------------
T_theileri_Tth.27.1380/1-325   --------------------------------------------
T_theileri_Tth.44.1550/1-167   --------------------------------------------
T_theileri_Tth.61.1080/1-163   --------------------------------------------
T_theileri_Tth.85.1060/1-407   --------------------------------------------
T_theileri_Tth.11.3490/1-239   --------------------------------------------

Conservation                   --------------------------------------------

Quality

Consensus                      SVGSVVQERRGEEHLRGSSAAKFTQNVHHSSNV---G----+SK
```

```
                                     930       940       950       960
T_theileri_Tth.11.2260/1-625   --------------------------------------------
T_theileri_Tth.70.1030/1-768   ------------------------------------------PG
T_theileri_Tth.107.1040/1-828  RHSIYLQATVAASP--TTCGRGKPVETFAGATECIVETVQFPPN
T_theileri_Tth.70.1110/1-944   ------------------------------------------PS
T_theileri_Tth.26.2530/1-142   --------------------------------------------
T_theileri_Tth.46.1090/1-145   --------------------------------------------
T_theileri_Tth.24.1770/1-211   --------------------------------------------
T_theileri_Tth.13.2480/1-341   --------------------------------------------
T_theileri_Tth.6.5070/1-728    -------------------------------------------D
T_theileri_Tth.2.1490/1-336    --------------------------------------------
T_theileri_Tth.136.1010/1-335  ------------------------------------------QN
T_theileri_Tth.46.1100/1-749   ------------------------------------------RR
T_theileri_Tth.83.1100/1-183   --------------------------------------------
T_theileri_Tth.85.1040/1-640   --------------------------------------------
T_theileri_Tth.36.2070/1-818   RRSLHVQAAMMASE--NKCAKGRRVENAAGATECIVEA---VRP
T_theileri_Tth.17.3250/1-783   ------------------------------------------GN
T_theileri_Tth.38.2000/1-661   --------------------------------------------
T_theileri_Tth.15.3170/1-626   -------------------------------------------E
T_theileri_Tth.36.2060/1-770   SRSLYGQTAMMASEPSSECTPGEDGE-VAGGKQCIVEA---PVF
T_theileri_Tth.61.1040/1-155   --------------------------------------------
T_theileri_Tth.121.1030/1-597  ------------------------------------------QN
T_theileri_Tth.10.2550/1-638   --------------------------------------------
T_theileri_Tth.54.1320/1-753   RRSIYAQTAMMASQTPSDCVAEKQVKTAKG-TECIVEA---PIY
T_theileri_Tth.101.1070/1-104  --------------------------------------------
T_theileri_Tth.2.4010/1-502    --------------------------------------------
T_theileri_Tth.11.2100/1-425   --------------------------------------------
T_theileri_Tth.25.1730/1-222   --------------------------------------------
T_theileri_Tth.1.6340/1-550    --------------------------------------------
T_theileri_Tth.2.4020/1-205    --------------------------------------------
T_theileri_Tth.6.5050/1-642    -------------------------------------------S
T_theileri_Tth.121.1040/1-345  ------------------------------------------KN
T_theileri_Tth.87.1070/1-161   --------------------------------------------
T_theileri_Tth.10.2540/1-581   -------------------------------------------G
T_theileri_Tth.70.1130/1-603   --------------------------------------------
T_theileri_Tth.125.1020/1-613  --------------------------------------------
T_theileri_Tth.107.1020/1-785  RRSLHVQAAMMASE--SKCAKGQPVETMAGETECIVEA---PMF
T_theileri_Tth.24.1760/1-439   --------------------------------------------
T_theileri_Tth.27.1380/1-325   --------------------------------------------
T_theileri_Tth.44.1550/1-167   --------------------------------------------
T_theileri_Tth.61.1080/1-163   --------------------------------------------
T_theileri_Tth.85.1060/1-407   --------------------------------------------
T_theileri_Tth.11.3490/1-239   --------------------------------------------
```

Conservation

Quality

Consensus

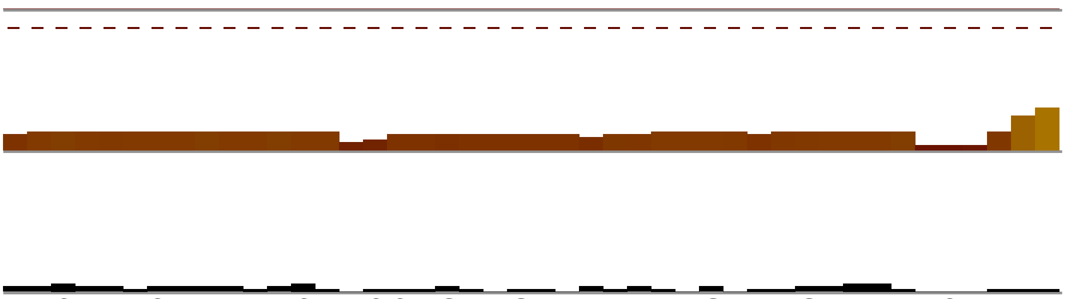

```
RRSLYLQAAVAASEPSSECTPGK+VETATG+TECIVET+QVP++
```

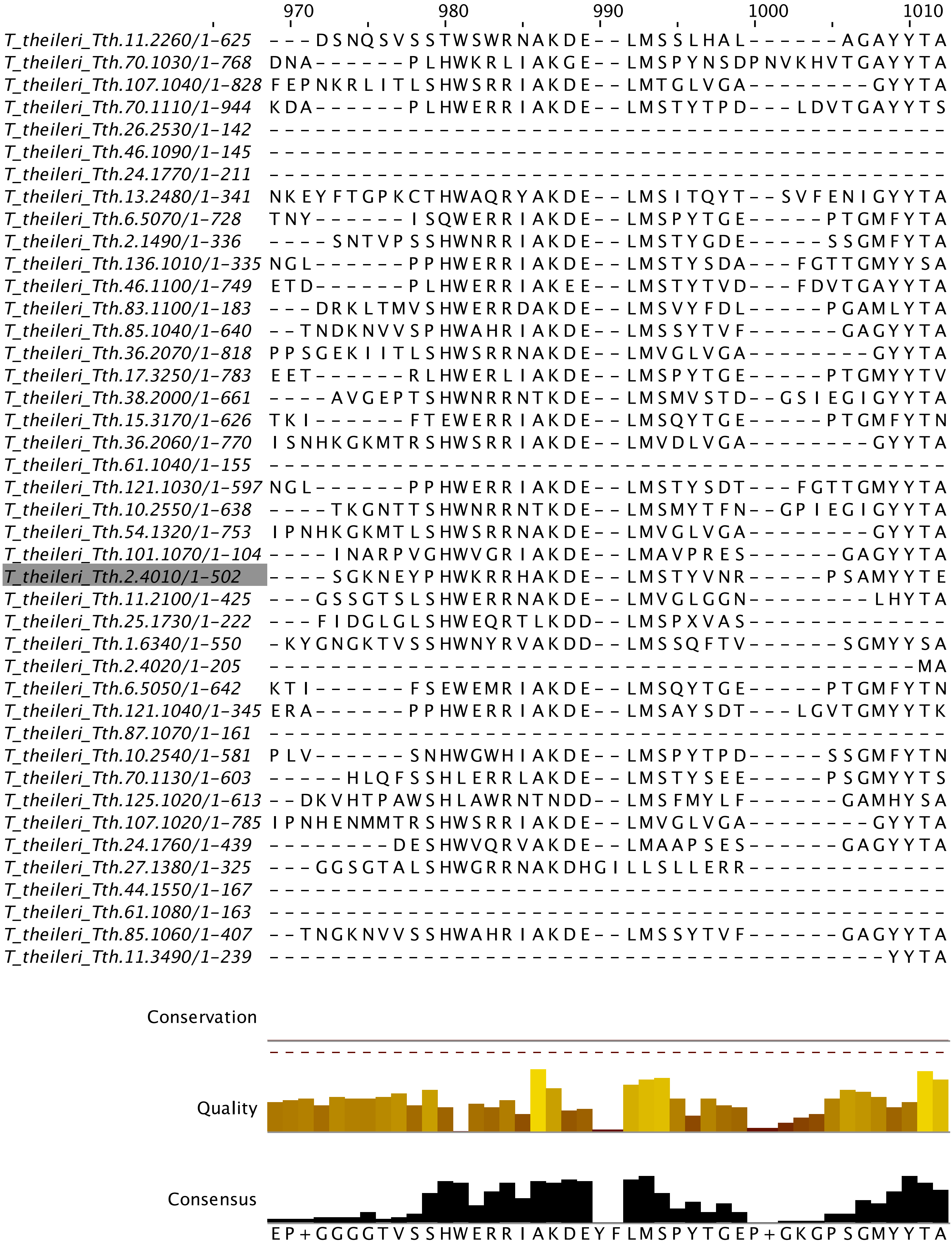
970 980 990 1000 1010
T_theileri_Tth.11.2260/1-625 ---DSNQSVSSTWSWRNAKDE--LMSSLHAL------AGAYYTA
T_theileri_Tth.70.1030/1-768 DNA------PLHWKRLIAKGE--LMSPYNSDPNVKHVTGAYYTA
T_theileri_Tth.107.1040/1-828 FEPNKRLITLSHWSRRIAKDE--LMTGLVGA--------GYYTA
T_theileri_Tth.70.1110/1-944 KDA------PLHWERRIAKDE--LMSTYTPD---LDVTGAYYTS
T_theileri_Tth.26.2530/1-142 --
T_theileri_Tth.46.1090/1-145 --
T_theileri_Tth.24.1770/1-211 --
T_theileri_Tth.13.2480/1-341 NKEYFTGPKCTHWAQRYAKDE--LMSITQYT--SVFENIGYYTA
T_theileri_Tth.6.5070/1-728 TNY------ISQWERRIAKDE--LMSPYTGE-----PTGMFYTA
T_theileri_Tth.2.1490/1-336 ----SNTVPSSHWNRRIAKDE--LMSTYGDE-----SSGMFYTA
T_theileri_Tth.136.1010/1-335 NGL------PPHWERRIAKDE--LMSTYSDA---FGTTGMYYSA
T_theileri_Tth.46.1100/1-749 ETD------PLHWERRIAKEE--LMSTYTVD---FDVTGAYYTA
T_theileri_Tth.83.1100/1-183 ---DRKLTMVSHWERRDAKDE--LMSVYFDL-----PGAMLYTA
T_theileri_Tth.85.1040/1-640 --TNDKNVVSPHWAHRIAKDE--LMSSYTVF------GAGYYTA
T_theileri_Tth.36.2070/1-818 PPSGEKIITLSHWSRRNAKDE--LMVGLVGA--------GYYTA
T_theileri_Tth.17.3250/1-783 EET------RLHWERLIAKDE--LMSPYTGE-----PTGMYYTV
T_theileri_Tth.38.2000/1-661 ----AVGEPTSHWNRRNTKDE--LMSMVSTD--GSIEGIGYYTA
T_theileri_Tth.15.3170/1-626 TKI------FTEWERRIAKDE--LMSQYTGE-----PTGMFYTN
T_theileri_Tth.36.2060/1-770 ISNHKGKMTRSHWSRRIAKDE--LMVDLVGA--------GYYTA
T_theileri_Tth.61.1040/1-155 --
T_theileri_Tth.121.1030/1-597 NGL------PPHWERRIAKDE--LMSTYSDT---FGTTGMYYTA
T_theileri_Tth.10.2550/1-638 ----TKGNTTSHWNRRNTKDE--LMSMYTFN--GPIEGIGYYTA
T_theileri_Tth.54.1320/1-753 IPNHKGKMTLSHWSRRNAKDE--LMVGLVGA--------GYYTA
T_theileri_Tth.101.1070/1-104 ----INARPVGHWVGRIAKDE--LMAVPRES------GAGYYTA
T_theileri_Tth.2.4010/1-502 ----SGKNEYPHWKRRHAKDE--LMSTYVNR-----PSAMYYTE
T_theileri_Tth.11.2100/1-425 ---GSSGTSLSHWERRNAKDE--LMVGLGGN--------LHYTA
T_theileri_Tth.25.1730/1-222 ---FIDGLGLSHWEQRTLKDD--LMSPXVAS-------------
T_theileri_Tth.1.6340/1-550 -KYGNGKTVSSHWNYRVAKDD--LMSSQFTV------SGMYYSA
T_theileri_Tth.2.4020/1-205 --MA
T_theileri_Tth.6.5050/1-642 KTI------FSEWEMRIAKDE--LMSQYTGE-----PTGMFYTN
T_theileri_Tth.121.1040/1-345 ERA------PPHWERRIAKDE--LMSAYSDT---LGVTGMYYTK
T_theileri_Tth.87.1070/1-161 --
T_theileri_Tth.10.2540/1-581 PLV------SNHWGWHIAKDE--LMSPYTPD-----SSGMFYTN
T_theileri_Tth.70.1130/1-603 -----HLQFSSHLERRLAKDE--LMSTYSEE-----PSGMYYTS
T_theileri_Tth.125.1020/1-613 --DKVHTPAWSHLAWRNTNDD--LMSFMYLF------GAMHYSA
T_theileri_Tth.107.1020/1-785 IPNHENMMTRSHWSRRIAKDE--LMVGLVGA--------GYYTA
T_theileri_Tth.24.1760/1-439 --------DESHWVQRVAKDE--LMAAPSES------GAGYYTA
T_theileri_Tth.27.1380/1-325 ---GGSGTALSHWGRRNAKDHGILLSLLERR-------------
T_theileri_Tth.44.1550/1-167 --
T_theileri_Tth.61.1080/1-163 --
T_theileri_Tth.85.1060/1-407 --TNGKNVVSSHWAHRIAKDE--LMSSYTVF------GAGYYTA
T_theileri_Tth.11.3490/1-239 --YYTA
Conservation
Quality
Consensus
EP+GGGGTVSSHWERRIAKDEYFLMSPYTGEP+GKGPSGMYYTA

```
                                      1020      1030      1040      1050
T_theileri_Tth.11.2260/1-625     LTMAAFDDMKYYSANWGMEEPMSWGNKSGCDFIRKGCL-TDKGV
T_theileri_Tth.70.1030/1-768     LTLAVFDSMPFYSANFDMAENMSWGKNVGCEFLKGKDKDKIITQ
T_theileri_Tth.107.1040/1-828    ITMGAFADLGYYKVNWTMAEQMSWGNNSKCEFLNNKCVNSGE--
T_theileri_Tth.70.1110/1-944     LTLAVFDSMPFYKASFDMAENMSWGKDAGCDFLKGGDEKKDEII
T_theileri_Tth.26.2530/1-142     --------------------------------------------
T_theileri_Tth.46.1090/1-145     --------------------------------------------
T_theileri_Tth.24.1770/1-211     --------------------------------------------
T_theileri_Tth.13.2480/1-341     LTIAAFEDMGFYKGKFXGS-------------------------
T_theileri_Tth.6.5070/1-728      LTLAVFDTRTYYQVDFSMAEPMSWGNQSGCELFKS-KCQDIIEK
T_theileri_Tth.2.1490/1-336      LTLATFHDMKFYQANFSMAE------------------------
T_theileri_Tth.136.1010/1-335    LTLAAFHSMPFYSANF----------------------------
T_theileri_Tth.46.1100/1-749     LTLAVFDSMPFYRADFKMAESMNWGKNASCDFLKDENKGKDKDT
T_theileri_Tth.83.1100/1-183     FTMAAFEDMKYFRANWGKEETMSWGKDAGCQFQHRKCVE-----
T_theileri_Tth.85.1040/1-640     LTMAFFEDLKYYTANWGMEEPMTWGNDSGCEFINDACT--NEMK
T_theileri_Tth.36.2070/1-818     LTLGAFADLGYYKVDWTMAEQMSWGNNAGCEFLNNKCVNSGE--
T_theileri_Tth.17.3250/1-783     LTLAVFADMKFYEVNFSMAEPMSWGNQSGCEFLQG-EKVTTK--
T_theileri_Tth.38.2000/1-661     LTIASFEDLGFYRGNFKMAEPMSWGYKANCKFLEATCKEKDKP-
T_theileri_Tth.15.3170/1-626     LTLAVFDTMSFYTANFSMAEPMSWGNQSGRDFITN-KCTELI-K
T_theileri_Tth.36.2060/1-770     ITMGAFADLGYYKVNWTMAEQMSWGNNSGCGLLEDKCVEGGS--
T_theileri_Tth.61.1040/1-155     --------------------------------------------
T_theileri_Tth.121.1030/1-597    LTLAAFHSMPFYSANFSMAEPMSWGKQYICDLFKG-KKDLTQ--
T_theileri_Tth.10.2550/1-638     LTIASFEDLGFYKGNFNMSEPMSWGYHAGCDFLEATCKEKDKR-
T_theileri_Tth.54.1320/1-753     ITMGAFADLGYYKVNWTMAEQMSWGNNSKCEFLNNKCVNSGE--
T_theileri_Tth.101.1070/1-104    LTMSTFESLGYYKANWGMEEPMGWGNRSGCDFLKGNCK--KDNK
T_theileri_Tth.2.4010/1-502      LTLAAFHSLPYYKADFSKAETMTWGRNASCEFLTGKCVEDSTP-
T_theileri_Tth.11.2100/1-425     ITMALFEDLGYYKADFSKAETMRWGKDAGCDFLRKPCF--VDGV
T_theileri_Tth.25.1730/1-222     --------------------------------------------
T_theileri_Tth.1.6340/1-550      LTMSVFDDLPYYSANWGMEEPMAWGNKSNCDLLDNNFE--NAFV
T_theileri_Tth.2.4020/1-205      FFRFLFLPIRFNSHN-------SWG-------------------
T_theileri_Tth.6.5050/1-642      LTLGVFDALSFYKSNFSMAEPMSWGNQSECEFITN-KCTELA-K
T_theileri_Tth.121.1040/1-345    LTLAAFEGMGFYTAN---------SLVRLCYLIV----------
T_theileri_Tth.87.1070/1-161     --------------------------------------------
T_theileri_Tth.10.2540/1-581     LTLGAFHDMPYYRANFSMAEPMSWGNQSGCNFVGK-EGDIQRDK
T_theileri_Tth.70.1130/1-603     LTLAIFNDMKFYKANFSMAETMSWGSNAGCEFLTGECIQENII-
T_theileri_Tth.125.1020/1-613    LTMSVFDDLPFYTVNWGMEESMSWGNQSGCELFKSECK--AEDA
T_theileri_Tth.107.1020/1-785    ITMGAFADLGYYKVNWTMAEQMSWGNNSNCEFLNNKCVNSGE--
T_theileri_Tth.24.1760/1-439     LTMSTFEGLGYYKANWGMEEPMSWGHKGGCDFLKETCT--ENEK
T_theileri_Tth.27.1380/1-325     --------------------------------------------
T_theileri_Tth.44.1550/1-167     --------------------------------------------
T_theileri_Tth.61.1080/1-163     --------------------------------------------
T_theileri_Tth.85.1060/1-407     LTMSVFDDLPYYSANWGMEEPMAWGNNSGCEFINGTCTVNTENT
T_theileri_Tth.11.3490/1-239     LTMSTFEGLGYYKANWGMEEPMSWGHKSGCNFLNGSCT--ENEK
```

Conservation

Quality

Consensus

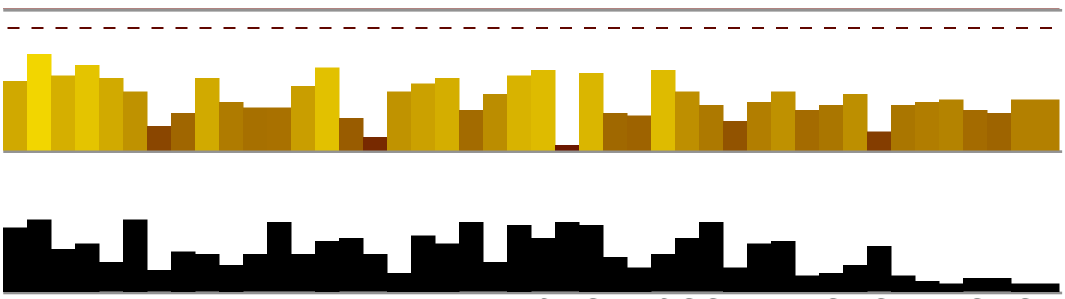

```
                                 LTLAAFEDMPFYKANF+MAEPMSWGNNSGCDFLKGKCVEEGEGK
```

```
                                1060      1070      1080      1090
T_theileri_Tth.11.2260/1-625   S----------NYPNAFCT---------DERLRCSSDRFGIARC
T_theileri_Tth.70.1030/1-768   KRIPK------KYSEMFCDEVK-------PAVQCTSDRFALGRC
T_theileri_Tth.107.1040/1-828  ----T------KFSNMFCTTKSTD---GPAGLQCTSDRQSLGRC
T_theileri_Tth.70.1110/1-944   Q----------KHNEMFCKRSE-------LILECTSDRFALGRC
T_theileri_Tth.26.2530/1-142   --------------------------------------------
T_theileri_Tth.46.1090/1-145   --------------------------------------------
T_theileri_Tth.24.1770/1-211   --------------------------------------------
T_theileri_Tth.13.2480/1-341   ---------------VFCA-------------------------
T_theileri_Tth.6.5070/1-728    D--LG------NLSKTFCKESK-----DEPTLQCTSDRFGLGVC
T_theileri_Tth.2.1490/1-336    --------------------------------------------
T_theileri_Tth.136.1010/1-335  --------------------------------------------
T_theileri_Tth.46.1100/1-749   VIK--------KYPEMFCNEIK-------GGLECTSDRFALGRC
T_theileri_Tth.83.1100/1-183   --------------------------------------------
T_theileri_Tth.85.1040/1-640   R----------KYNNMFCD---------TESPRCTSDRFAVGRC
T_theileri_Tth.36.2070/1-818   ----T------DFSNMFCTTKSTDSSDSPAGLQCTSDRQSLGRC
T_theileri_Tth.17.3250/1-783   ----T------DYPNVFCKKEEKT---ETKILQCTSDRFALGMC
T_theileri_Tth.38.2000/1-661   -----------VADGNSCS--------PYPAGSCTSDRLGVAKC
T_theileri_Tth.15.3170/1-626   D---P------KYNKVFCDEGE-----DKSVLKCTSDRFGLGVC
T_theileri_Tth.36.2060/1-770   ----T------KFPDMFCTTKSGD---IPESLQCTSDRQSMGRC
T_theileri_Tth.61.1040/1-155   --------------------------------------------
T_theileri_Tth.121.1030/1-597  ----T------HYPDMFCKDDT-------MTLRCTSDRFALGIC
T_theileri_Tth.10.2550/1-638   -----------IDAAKFCT--------TFPTDSCTSDRLGLGIC
T_theileri_Tth.54.1320/1-753   ----T------KFSNMFCTTKSSK---GTESLRCTSDRQSLGTC
T_theileri_Tth.101.1070/1-104  L-------------------------------------------
T_theileri_Tth.2.4010/1-502    -----------KFPEMFCN-------DSTTVLRCTTDRASLGTC
T_theileri_Tth.11.2100/1-425   S----------PYPDMFCN-----QLKGNKSLLCTFDRLSLGFC
T_theileri_Tth.25.1730/1-222   --------------------------------------------
T_theileri_Tth.1.6340/1-550    G----------KYPTMFCS--------IGKPYSCTTDRSAVGYC
T_theileri_Tth.2.4020/1-205    --------------------------------------------
T_theileri_Tth.6.5050/1-642    D---P------KYNKFFCDEGE-----DKSVLKCTSDRFGLGVC
T_theileri_Tth.121.1040/1-345  --------------------------------------------
T_theileri_Tth.87.1070/1-161   --------------------------------------------
T_theileri_Tth.10.2540/1-581   I--TK------ENPNMFCTG-------EKQGLQCTSDRFALGVC
T_theileri_Tth.70.1130/1-603   -----------KYPNTFCT-------ESRVSLQCTSDRLSLGRC
T_theileri_Tth.125.1020/1-613  T----------KYPGRFCD-----INKDKNVFSCTSDRLGFGTC
T_theileri_Tth.107.1020/1-785  ----T------NFLNMFCTTKSDDIPDAPAGLQCTSDRQSMGRC
T_theileri_Tth.24.1760/1-439   -----------HFSDQFCN--------DTTALGCTSDRTAYGMC
T_theileri_Tth.27.1380/1-325   --------------------------REEQSRTVTSDFLAHGFF
T_theileri_Tth.44.1550/1-167   --------------------------------------------
T_theileri_Tth.61.1080/1-163   --------------------------------------------
T_theileri_Tth.85.1060/1-407   K----------TTTKMFCS--------RGVYSQCTSDRFAVGSC
T_theileri_Tth.11.3490/1-239   -----------HYPKQFCS---------DATSRCTSDRSAYGKC
```

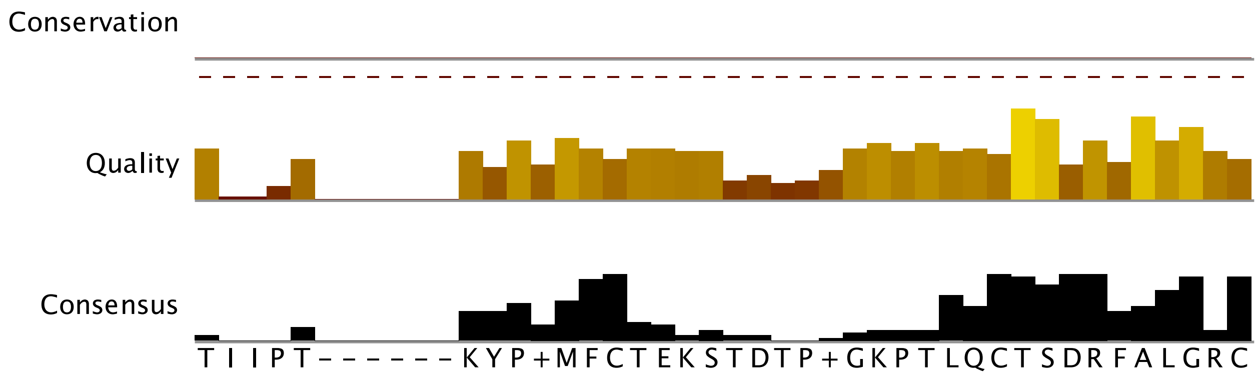

```
                                       1110      1120      1130      1140
T_theileri_Tth.11.2260/1-625    FSVDVDEVHE-------------------------DPTGSCPVF
T_theileri_Tth.70.1030/1-768    TKEMPKEIEK--KLPLDYLILFNS--ITSDPEK--DLMDDYPII
T_theileri_Tth.107.1040/1-828   S---IQTY-ER-DLPGHF-QYFSD--GKLGGNKT-DLMDYCPFI
T_theileri_Tth.70.1110/1-944    TKDIELK-----GSPFEY-AYFNHFFFTETDER--ELMDGYPFI
T_theileri_Tth.26.2530/1-142    ---------------------------------HREEKRECAVV
T_theileri_Tth.46.1090/1-145    --------------------------------------------
T_theileri_Tth.24.1770/1-211    --------------------------------------------
T_theileri_Tth.13.2480/1-341    --------------------------------------------
T_theileri_Tth.6.5070/1-728     SKDDNK---KD-GMPAEY-NYFKK--N--NIRNS-DMMEGCPII
T_theileri_Tth.2.1490/1-336     --------------------------------------------
T_theileri_Tth.136.1010/1-335   --------------------------------------------
T_theileri_Tth.46.1100/1-749    TKEMVLKDIQ--NISPEY-AYFND--LTSSYGDLYDLTDGYPII
T_theileri_Tth.83.1100/1-183    --------------------------------------------
T_theileri_Tth.85.1040/1-640    TSFTGYRVY--------------------------TEPDECPFA
T_theileri_Tth.36.2070/1-818    SSIGAIPV-KN-DLPVGF-RYFSDS-KTVGSEAR-EQMDFCPFI
T_theileri_Tth.17.3250/1-783    STKKSR----E-GLPDGY-RYFDD--ENYH-FES-NLMDGVPFI
T_theileri_Tth.38.2000/1-661    VEPK-------------------------------NGNPSCPIL
T_theileri_Tth.15.3170/1-626    SKDNTN---KH-SMSDEY-KFFET--V--G-EQS-EMIKECPII
T_theileri_Tth.36.2060/1-770    SSIGANPV-KN-DLPVGF-RYFSDS-KTVGSEES-EEMDYCPFV
T_theileri_Tth.61.1040/1-155    --------------------------------------------
T_theileri_Tth.121.1030/1-597   STDAKR----N-NLRGRY-QYIKD--EYRQNDAN-DLMDGVPFI
T_theileri_Tth.10.2550/1-638    FQSP-------------------------------ETTSNCPIF
T_theileri_Tth.54.1320/1-753    SSIEAKPV-EK-DLPARF-QYFSDD-KKVGSDSS-EQMDYCPFI
T_theileri_Tth.101.1070/1-104   --------------------------------------------
T_theileri_Tth.2.4010/1-502     SMKQLAS-----DVPREF-QYFTN--KRIGSPSS-DMMDHCPVI
T_theileri_Tth.11.2100/1-425    TLSKYVQ-----HLPQHF-QYFKD--PTLGGVG--LLADFCPFV
T_theileri_Tth.25.1730/1-222    --------------------------------------------
T_theileri_Tth.1.6340/1-550     IPESRYTDSDGNEINAAFVRS-----------------------
T_theileri_Tth.2.4020/1-205     ------------------------------IYRVYTEPDDCPFA
T_theileri_Tth.6.5050/1-642     SKDNTN---KH-GIPDEY-NFFEI--V--G-EQS-DMIKECPII
T_theileri_Tth.121.1040/1-345   --------------------------------------------
T_theileri_Tth.87.1070/1-161    --------------------------------------------
T_theileri_Tth.10.2540/1-581    SKKDTP---PK-AISEEY-HYFEK--D-AGIGRG-DLFNDFSVI
T_theileri_Tth.70.1130/1-603    TSSNFPQ-----DYPEDY-RNFKN--NTFTYLYG-ELTDGCPII
T_theileri_Tth.125.1020/1-613   KQHGDDRHP--------------------------ENKQKCFVS
T_theileri_Tth.107.1020/1-785   SSIESKTV-K--DLPEHF-RYFSEN-NKVGSDSS-EHMDYCPFI
T_theileri_Tth.24.1760/1-439    SQESQRQANP-------------------------SSNDVCHLI
T_theileri_Tth.27.1380/1-325    --------------------------------------------
T_theileri_Tth.44.1550/1-167    --------------------------------------------
T_theileri_Tth.61.1080/1-163    --------------------------------------------
T_theileri_Tth.85.1060/1-407    TSVMGYRVY--------------------------TEPDDCPFA
T_theileri_Tth.11.3490/1-239    YRGQQREYQ--------------------------GPNDVCRVI
```

Conservation

Quality

Consensus

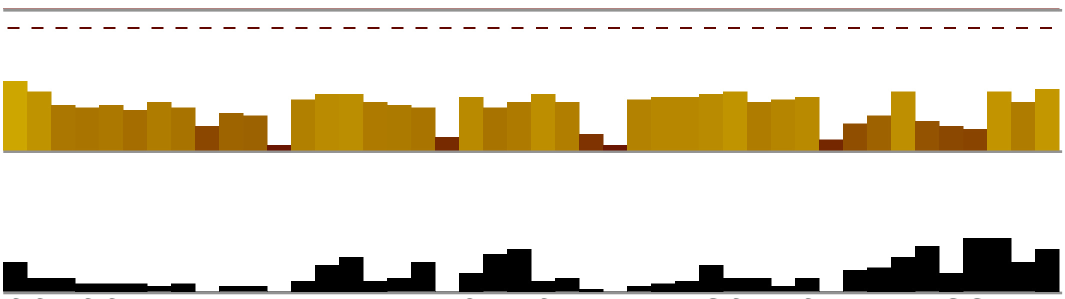

```
SSKSSPTVEKNNDLPEEY+QYFSDNT++VGSEESRELMDGCPII
```

```
                                   1150      1160      1170      1180
T_theileri_Tth.11.2260/1-625   LSPF----HMELSAPLH-SFPC----TELEE-GS--FLGFLTG-
T_theileri_Tth.70.1030/1-768   KPF-----S---------STSC----EKGTE-TQ--MPGSVVG-
T_theileri_Tth.107.1040/1-828  TAE-----K---------GYSC----INGEEGIK--MPGSVIA-
T_theileri_Tth.70.1110/1-944   KPI-----D---------WTNC----ENGRV-NL--MPGSFVG-
T_theileri_Tth.26.2530/1-142   KATA--------------ATVC----ENGSE-AH--MPGSRIG-
T_theileri_Tth.46.1090/1-145   --------------------------------------------
T_theileri_Tth.24.1770/1-211   --------------------------------------------
T_theileri_Tth.13.2480/1-341   --------------------------------------------
T_theileri_Tth.6.5070/1-728    KPF-----Q---------ETMC----ETGNE-TL--MPGSIVS-
T_theileri_Tth.2.1490/1-336    --------------------------------------------
T_theileri_Tth.136.1010/1-335  --------------------------------------------
T_theileri_Tth.46.1100/1-749   KPI-----Y---------TTSC----ESGNL-SL--MPGSIVG-
T_theileri_Tth.83.1100/1-183   --------------------------------------------
T_theileri_Tth.85.1040/1-640   KPE-----HVYTSNGML-SALC----TLTVN-NT--IPGSVTG-
T_theileri_Tth.36.2070/1-818   TAN-----Q---------GYSC----VTGEEKNK--MPGSVIA-
T_theileri_Tth.17.3250/1-783   RPL-----M---------GTAC----EGGNP-SL--MPGSVLG-
T_theileri_Tth.38.2000/1-661   KLN-----T---------EVYC----DITF--GLPKNWPGLHG-
T_theileri_Tth.15.3170/1-626   KPF-----Q---------QTMC----ENGKE-TL--MPGSIVS-
T_theileri_Tth.36.2060/1-770   KAL-----P---------QKSC----ISVEKSNN--MPGSKIG-
T_theileri_Tth.61.1040/1-155   --------------------------------------------
T_theileri_Tth.121.1030/1-597  RPL-----N---------GTAC----EGGEE-EL--MPGNLVS-
T_theileri_Tth.10.2550/1-638   AAN-----E---------MFFC----ATE---GTSYGWSGLFG-
T_theileri_Tth.54.1320/1-753   KAL-----P---------QKSC----ISGER-NQ--MPGSVIA-
T_theileri_Tth.101.1070/1-104  --------------------------------------------
T_theileri_Tth.2.4010/1-502    ESS-----W---------KTSC----EFGDE-DA--MPGSIVS-
T_theileri_Tth.11.2100/1-425   QEY-----V---------NGGC----TNGEA-RA--MLGSRIG-
T_theileri_Tth.25.1730/1-222   --------------------------------------------
T_theileri_Tth.1.6340/1-550    --------ELYNSANTG-SMSC----SDKVT-SD--IPGSIFD-
T_theileri_Tth.2.4020/1-205    KPE-----HVYTSNGML-SALC----TLTVN-NT--IPGSVTG-
T_theileri_Tth.6.5050/1-642    KPF-----Q---------ETMC----ETGNE-TF--MPGSIVS-
T_theileri_Tth.121.1040/1-345  --------------------------------------------
T_theileri_Tth.87.1070/1-161   --------------------------------------------
T_theileri_Tth.10.2540/1-581   KPF-----K---------ETMC----ENGNE-TL--MPGSIVD-
T_theileri_Tth.70.1130/1-603   RTL-----E---------ETTC----QSGDL-EL--MPGSILG-
T_theileri_Tth.125.1020/1-613  ESVIHP--YYEKGKDHM-FLLC----SEIPL-FN--FPGSIFGN
T_theileri_Tth.107.1020/1-785  TST-----E---------GYSC----INGVQ-SQ--MPGSVIA-
T_theileri_Tth.24.1760/1-439   GGS--------PEDLFTSFSYC----TEARG-GP--LTGSLMG-
T_theileri_Tth.27.1380/1-325   --------------------------------------------
T_theileri_Tth.44.1550/1-167   --------------------------------------------
T_theileri_Tth.61.1080/1-163   --------------------------------------------
T_theileri_Tth.85.1060/1-407   KPE-----HVYTSNGML-SALC----TL----------------
T_theileri_Tth.11.3490/1-239   GSF------TEGNDNVKTYTYC----TGAET-NA--LPGSLMG-

Conservation                   --------------------------------------------

Quality

Consensus                      KPLAPP--PVYTS+G+L+GTSC----EDGEESNLP+MPGSIVGR
```

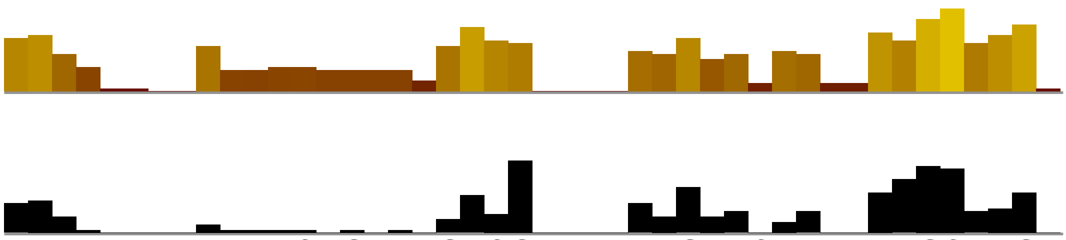

```
                                   1190      1200      1210      1220      1230
T_theileri_Tth.11.2260/1-625      ENSMCLTTEDY-NLERE-------NGTEKVTEIMSGVCAQVLCD
T_theileri_Tth.70.1030/1-768      KESRCLKGENL-TLKE----PN--EDGPF----VGDICAKVKCD
T_theileri_Tth.107.1040/1-828     NNSRCVEGKDL-KANN----------NAA----VGAVCVEVSCK
T_theileri_Tth.70.1110/1-944      SESRCLKGENL-ELKE----ER--EEKVT----VRGICADVKCD
T_theileri_Tth.26.2530/1-142      NTSRCLKGDAL-RLRD--------VSTIDIHSIIGDICANVRCE
T_theileri_Tth.46.1090/1-145      --------------------------------------------
T_theileri_Tth.24.1770/1-211      --------------------------------------------
T_theileri_Tth.13.2480/1-341      --------------------------------------------
T_theileri_Tth.6.5070/1-728       NMSRCLDVEDP-KFVD---GNN--GIGLK----VQGICAKVKCE
T_theileri_Tth.2.1490/1-336       --------------------------------------------
T_theileri_Tth.136.1010/1-335     --------------------------------------------
T_theileri_Tth.46.1100/1-749      NTSRCLKGDKL-KLHK----RN--KFSLS----VGDICADVKCD
T_theileri_Tth.83.1100/1-183      --------------------------------------------
T_theileri_Tth.85.1040/1-640      NDSWCLDGDSL-KVND--------VAGRKNNVSVGGVCARVKCE
T_theileri_Tth.36.2070/1-818      NNSRCVEGKDL-KTKN----------DAA----VGAVCVEVSCK
T_theileri_Tth.17.3250/1-783      PDSRCLTADGL-VLRD----SK--STALT----IAGVCAKVKCE
T_theileri_Tth.38.2000/1-661      EYSWCLDSDAL-TFNDDKGKSVT----------INGMCAAVECS
T_theileri_Tth.15.3170/1-626      NMSRCLDVKET-VEID---ENG---KKVK----VQGICAKVKCD
T_theileri_Tth.36.2060/1-770      MNSRCVEGKEL-KTKD----------DAA----VGAVCVEVSCK
T_theileri_Tth.61.1040/1-155      --------------------------------------------
T_theileri_Tth.121.1030/1-597     SMSRCLDLETP-ILKD-----N--KDDVK----VQAVCAKVKCD
T_theileri_Tth.10.2550/1-638      KDSWCLDVDYL-KPQQDGENNM-----------KQGMCAAVDCS
T_theileri_Tth.54.1320/1-753      NNSRCVEGNGL-TAN-----------NAA----VGAVCVEVSCK
T_theileri_Tth.101.1070/1-104     --------------------------------------------
T_theileri_Tth.2.4010/1-502       NMSRCLEGEFL-EPREP-------KYIGT----FRDICANVKCE
T_theileri_Tth.11.2100/1-425      PNSRCLKGNSL-RMGTYS---------------LGDVCVNTQCD
T_theileri_Tth.25.1730/1-222      ------------------------------------VCKE----
T_theileri_Tth.1.6340/1-550       NDSFCLDTEEY-TRVVG-------QDTLS----FTGVCARVSCD
T_theileri_Tth.2.4020/1-205       NDSWCLDGDSL-KVKVKG------SANKV----VGGVCARVSCD
T_theileri_Tth.6.5050/1-642       NMSRCLNVEEL-QFND---GNN--VTGLK----VQGICAKVKCE
T_theileri_Tth.121.1040/1-345     --------------------------------------------
T_theileri_Tth.87.1070/1-161      --------------------------------------------
T_theileri_Tth.10.2540/1-581      KMSRCLSVKDP-QIKD----VN--GNGVT----VQGICAKVKCE
T_theileri_Tth.70.1130/1-603      KESRCLQGNEL-IQKESA------QTFLP----IGDICANVKCE
T_theileri_Tth.125.1020/1-613     NDSLCLDTEEY-SAEV--------SENGE--MGLTGICARVSCD
T_theileri_Tth.107.1020/1-785     NNSRCVEGNEL-KAA-----------NAA----VGAVCVEVSCK
T_theileri_Tth.24.1760/1-439      PDSWCLDGEGL-QVQNA----------GGRVESLSGVCARVSCD
T_theileri_Tth.27.1380/1-325      --------------------------------------------
T_theileri_Tth.44.1550/1-167      --------------------------------------------
T_theileri_Tth.61.1080/1-163      --------------------------------------------
T_theileri_Tth.85.1060/1-407      --------------------------------------------
T_theileri_Tth.11.3490/1-239      PDSWCLDGEGL-QVQNA----------GESAESLSGVCARVSCD
```

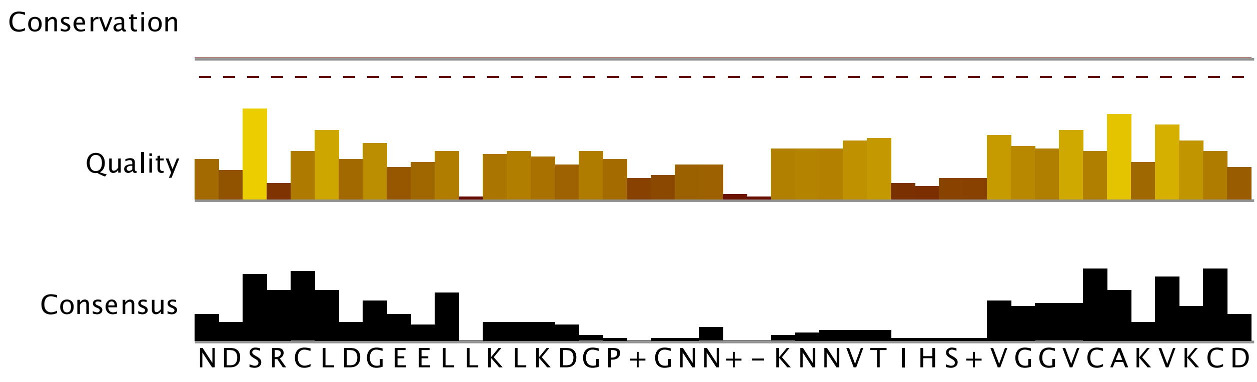

```
                              1240      1250      1260      1270
T_theileri_Tth.11.2260/1-625  -ESNRKVKVKYSG-----LSD--------FQECP-EGGN-----
T_theileri_Tth.70.1030/1-768  -KNTKKVSVQYSG-----TKD--------WQEC-KDGK-----V
T_theileri_Tth.107.1040/1-828 -FNK--VIVRYSG-----NDK--------WYSC-PEEENLT---
T_theileri_Tth.70.1110/1-944  ---DGKVKVKYNG-----SEI--------WHIC-TDDEK----I
T_theileri_Tth.26.2530/1-142  ---SGKVSVQYKG-----DDH--------WYEC-KEGNSISPS-
T_theileri_Tth.46.1090/1-145  --------------------------------------------
T_theileri_Tth.24.1770/1-211  --------------------------------------------
T_theileri_Tth.13.2480/1-341  --------------------------------------------
T_theileri_Tth.6.5070/1-728   -NDK--VLVHYKG-----QN-D-NGKEK-WHTC-ENDGQ---QI
T_theileri_Tth.2.1490/1-336   --------------------------------------------
T_theileri_Tth.136.1010/1-335 --------------------------------------------
T_theileri_Tth.46.1100/1-749  -KDNEKVMVRYSG-----SEI--------WHNC-SDGKKIDVE-
T_theileri_Tth.83.1100/1-183  --------------------------------------------
T_theileri_Tth.85.1040/1-640  --GNGVLKVMYAG-----NDT--------WHDCS-EGHSLEPS-
T_theileri_Tth.36.2070/1-818  -FNK--VSVRYSG-----NND--------WHSC-PEEENLT---
T_theileri_Tth.17.3250/1-783  -DDKK-VSVQLKQ----------DGGNN-WHDC-SQAGK---SI
T_theileri_Tth.38.2000/1-661  --DDGVVKVQLKK-----DGE--------WYNCPKEGVES----
T_theileri_Tth.15.3170/1-626  -NGK--VHVQYKG-----DKEKVNGEQK-WHEC-TEEGT---I-
T_theileri_Tth.36.2060/1-770  -FNK--VIVRYSG-----NDK--------WYSC-PEGTNLT---
T_theileri_Tth.61.1040/1-155  --------------------------------------------
T_theileri_Tth.121.1030/1-597 -NDNKKVSVQLKG-----HDNK---ELN-WYKC-ENDGE---SI
T_theileri_Tth.10.2550/1-638  --DDGVVKVQLKE-----DGE--------WFSCPREDGV-----
T_theileri_Tth.54.1320/1-753  -FNK--VIVRYSG-----NDE--------WYSC-PEGKNLT---
T_theileri_Tth.101.1070/1-104 --------------------------------------------
T_theileri_Tth.2.4010/1-502   ---NGILKVQYKG-----NEK--------WHEC-TDGKRIDIE-
T_theileri_Tth.11.2100/1-425  ---KGTLRVQLLD-----DDT--------WYTCK-EGEHI----
T_theileri_Tth.25.1730/1-222  -----------------------------WHGI-----------
T_theileri_Tth.1.6340/1-550   ---GGKVKVMYAG-----DNK--------WYDCP-EGNLLVP--
T_theileri_Tth.2.4020/1-205   ---EGKVKVMYAG-----NDT--------WHDCP-EGGS-----
T_theileri_Tth.6.5050/1-642   -DDKQTVSVQLKG-----QESK----ET-WHVC-ENDGT---T-
T_theileri_Tth.121.1040/1-345 --------------------------------------------
T_theileri_Tth.87.1070/1-161  --------------------------------------------
T_theileri_Tth.10.2540/1-581  -NGK--VLVHYKG-----NKEQ-NGKEK-WRTC-ENDGQ---QI
T_theileri_Tth.70.1130/1-603  ---DGKVKVQYKG-----NDK--------WHEC-PDGGKIENL-
T_theileri_Tth.125.1020/1-613 ---EGKVRVMYDG-----ISG--------WQDCS-ENTS-----
T_theileri_Tth.107.1020/1-785 -FNK--VIVRYSG-----NNE--------WYSC-PEGKNLT---
T_theileri_Tth.24.1760/1-439  -EGRRAVEVQYKG-----SDT--------FKE------------
T_theileri_Tth.27.1380/1-325  --------------------------------------------
T_theileri_Tth.44.1550/1-167  --------------------------------------------
T_theileri_Tth.61.1080/1-163  --------------------------------------------
T_theileri_Tth.85.1060/1-407  --------------------------------------------
T_theileri_Tth.11.3490/1-239  -EGRRAVEVQYKG-----SDT--------FKECP-EGTSID---
```

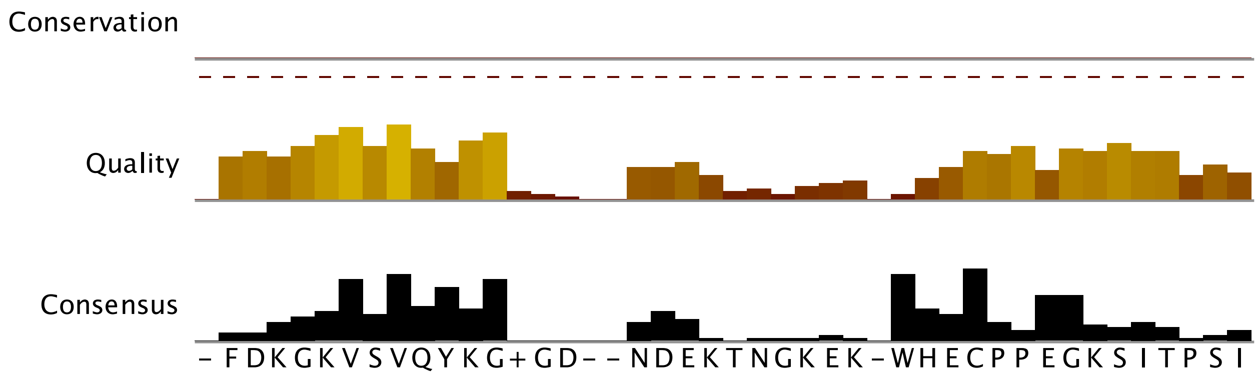

```
                              1280      1290      1300      1310
T_theileri_Tth.11.2260/1-625   ----IT---VSSSSASLNFKTIY-CPNYTEVCTVASDGSSLHPL
T_theileri_Tth.70.1030/1-768   DV--T-----GSTEF--ASGSIL-CPNYTEVCNDFSEVRDINFT
T_theileri_Tth.107.1040/1-828  ----L-----NGTVL--Q-GEIV-CPKYADVCNTINKTLDESQG
T_theileri_Tth.70.1110/1-944   NV--------ENSPDLKSGGSIF-CPKYSEVCNE-KKNNATEFP
T_theileri_Tth.26.2530/1-142   ----------VTSAF--SSGRIV-CPPYSEVCMDLPIEPPPTEA
T_theileri_Tth.46.1090/1-145   --------------------------------------------
T_theileri_Tth.24.1770/1-211   --------------------------------------------
T_theileri_Tth.13.2480/1-341   --------------------------------------------
T_theileri_Tth.6.5070/1-728    -T--L-----EGSVF--SGGTIIRCPKYAEVCTKETNTDLLPEI
T_theileri_Tth.2.1490/1-336    --------------------------------------------
T_theileri_Tth.136.1010/1-335  --------------------------------------------
T_theileri_Tth.46.1100/1-749   ----------KNSDF--QSGSIF-CPKYAEVCTDLESS--TKFP
T_theileri_Tth.83.1100/1-183   --------------------------------------------
T_theileri_Tth.85.1040/1-640   ---------TTSSSF--VSGKIK-CPKYSEVCTMAPNGSSLLRH
T_theileri_Tth.36.2070/1-818   ----L-----NGTVL--Q-GKIV-CPKYADVCNTINRTLDESQG
T_theileri_Tth.17.3250/1-783   NLVNL-----DSSTF--SSGSIK-CPKYEEVCTGLPKTEST-KI
T_theileri_Tth.38.2000/1-661   ----IPLNGTEYSTY--VGKNIK-CPKYDEVCTVRLNGSSRLRL
T_theileri_Tth.15.3170/1-626   -N--L-----QGSVF--TSGTIV-CPKYAEVCTGLPETEPF-NI
T_theileri_Tth.36.2060/1-770   ----V-----NGSVL--Q-GKIV-CPKYADVCNTINKTLDESQG
T_theileri_Tth.61.1040/1-155   --------------------------------------------
T_theileri_Tth.121.1030/1-597  P---L-----EDSLF--NKGSVK-CPNFKEVCTGLPKTEPT-KI
T_theileri_Tth.10.2550/1-638   ----ITPNGTEYPTY--VNRSIK-CPKYSEVCTVSYNGSSLVKP
T_theileri_Tth.54.1320/1-753   ----V-----DGTVL--Q-GKIV-CPKYADVCNTINKTLDESQG
T_theileri_Tth.101.1070/1-104  --------------------------------------------
T_theileri_Tth.2.4010/1-502    ----------KGPAL--YGGKIV-CPKYSDFCTDKPEESQ----
T_theileri_Tth.11.2100/1-425   ---------TPSKVF--TSGTIL-CPKYEEVCPEVTEDNNDAAP
T_theileri_Tth.25.1730/1-222   --------------------TVM---------------------
T_theileri_Tth.1.6340/1-550    ---------TSTSSF--VSGKIM-CPKYNEVCTVAPDGSSRRPM
T_theileri_Tth.2.4020/1-205    ----LEPS-TNSSIF--AGGSIK-CPKYSEVCTMTPDGGSRLIP
T_theileri_Tth.6.5050/1-642    -N--L-----DGSVF--KKGTIIRCPKYEEVCTGLPETEPF-NI
T_theileri_Tth.121.1040/1-345  ------------------------VPRLST--------------
T_theileri_Tth.87.1070/1-161   --------------------------------------------
T_theileri_Tth.10.2540/1-581   -N--L-----EGSVI--SGGTIV-CPKYEEVCTKLPETDPP-AV
T_theileri_Tth.70.1130/1-603   ----------EGSEF--QSGSIL-CPKYAEMCTDFPQISELRIE
T_theileri_Tth.125.1020/1-613  ----IE---VNHPDSS-NIIKIK-CPKYSEVCTIFSDGSSLLPQ
T_theileri_Tth.107.1020/1-785  ----V-----NGSVL--Q-GKIV-CPKYADVCNTINKTLDESEG
T_theileri_Tth.24.1760/1-439   --------------------------------------------
T_theileri_Tth.27.1380/1-325   --------------------------------------------
T_theileri_Tth.44.1550/1-167   --------------------------------------------
T_theileri_Tth.61.1080/1-163   --------------------------------------------
T_theileri_Tth.85.1060/1-407   --------------------------------------------
T_theileri_Tth.11.3490/1-239   ---------VESSAFQ-SGGKIK-CPKYDEVCTITPDGRSRLSM
```

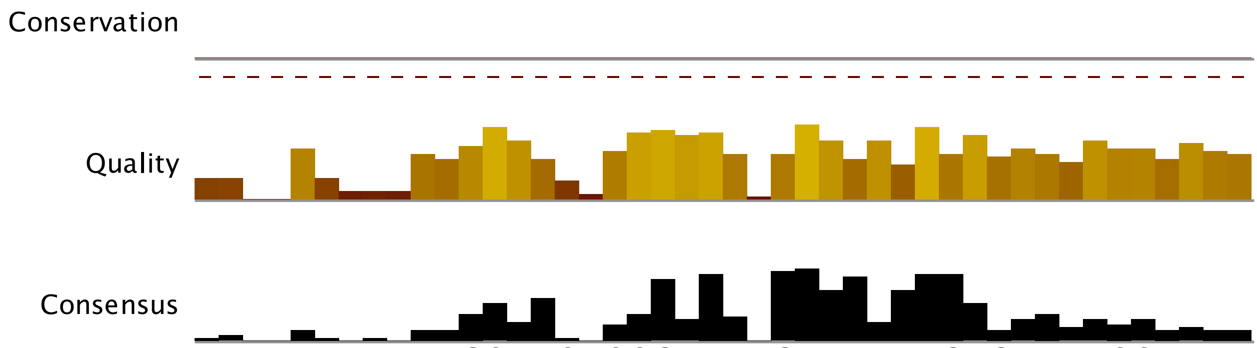


```
                               DV--V+PN+VEGSVFSLSSGKIVRCPKYAEVCTGLPNTSSLLPI
```

```
                              1330      1340      1350      1360
T_theileri_Tth.11.2260/1-625  LNRLPYAENTVSGAQGRLRGVRA---------------------
T_theileri_Tth.70.1030/1-768  IEYDEDVKLEIKKEK-EAEDKAQEKKQREEEDRKKNEQE-----
T_theileri_Tth.107.1040/1-828 PEKDPDIVEAVQTSETEVEKTTA---------------------
T_theileri_Tth.70.1110/1-944  IVYDKDERERMDKEDKEEEEKQRKEADRKKKEEEEERGR-----
T_theileri_Tth.26.2530/1-142  VVVMEEEVYNFTASINE---------------------------
T_theileri_Tth.46.1090/1-145  --------------------------------------------
T_theileri_Tth.24.1770/1-211  --------------------------------------------
T_theileri_Tth.13.2480/1-341  --------------------------------------------
T_theileri_Tth.6.5070/1-728   KYNVVQVPED--E-D---LKQEK----------REE---E----
T_theileri_Tth.2.1490/1-336   --------------------------------------------
T_theileri_Tth.136.1010/1-335 --------------------------------------------
T_theileri_Tth.46.1100/1-749  IEYDKDEEEKIKKEEEQEKKEMEEEKRKMEEEQRKMEEE-----
T_theileri_Tth.83.1100/1-183  --------------------------------------------
T_theileri_Tth.85.1040/1-640  IVPYPISPEEAESATESLQTSNE---------------------
T_theileri_Tth.36.2070/1-818  PASDPDPVKAIQTSQPEVETTTT---------------------
T_theileri_Tth.17.3250/1-783  KFYNGTGGTAMYEVDANDDEQSK----------KGEKATEGH--
T_theileri_Tth.38.2000/1-661  QVEEQKQSTPTNEENAVNGTTDT---------------------
T_theileri_Tth.15.3170/1-626  SYTEVNVPEDKVE-EEETPGEEE----------EEKPGDE----
T_theileri_Tth.36.2060/1-770  PATDPDPVKAIQTSQPE---------------------------
T_theileri_Tth.61.1040/1-155  --------------------------------------------
T_theileri_Tth.121.1030/1-597 KFYNGTKITESYVDVKDDDENEE----------PAQAQNTVRTA
T_theileri_Tth.10.2550/1-638  K--EEKLTTPTTEENTVSGTTDT---------------------
T_theileri_Tth.54.1320/1-753  PEKDPDTVEAIQTSQPE---------------------------
T_theileri_Tth.101.1070/1-104 --------------------------------------------
T_theileri_Tth.2.4010/1-502   --------------------------------------------
T_theileri_Tth.11.2100/1-425  ETSSETRSSKGDRNRTSSEKSSRQRSYPYRWNTRRRPRIE----
T_theileri_Tth.25.1730/1-222  --------------------------------------------
T_theileri_Tth.1.6340/1-550   IYPPGYEHPTESPGQGG---------------------------
T_theileri_Tth.2.4020/1-205   KTSERRQRLTTNSTRARGGSRRR---------------------
T_theileri_Tth.6.5050/1-642   SYTEVNVSEDVVE-E---PKQEE----------KEK---E----
T_theileri_Tth.121.1040/1-345 --------------------------------------------
T_theileri_Tth.87.1070/1-161  --------------------------------------------
T_theileri_Tth.10.2540/1-581  EYEEYVEPQK--------NKEKK----------EEE---E----
T_theileri_Tth.70.1130/1-603  SNEAADIEILQGYVIGRDLHDIS---------------------
T_theileri_Tth.125.1020/1-613 IEPKRPPAPATTTTTTTTENNES---------------------
T_theileri_Tth.107.1020/1-785 PSKDPDIVEEVQTSQPE---------------------------
T_theileri_Tth.24.1760/1-439  --------------------------------------------
T_theileri_Tth.27.1380/1-325  --------------------------------------------
T_theileri_Tth.44.1550/1-167  --------------------------------------------
T_theileri_Tth.61.1080/1-163  --------------------------------------------
T_theileri_Tth.85.1060/1-407  --------------------------------------------
T_theileri_Tth.11.3490/1-239  NVEPDSTRRAAA--------------------------------
```

Conservation

Quality

Consensus

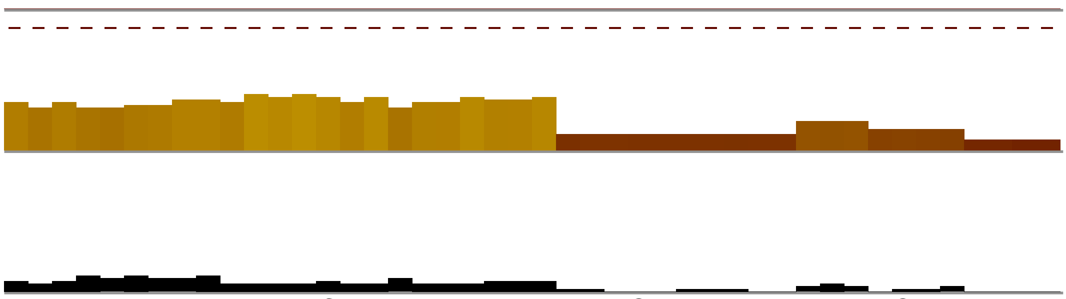

```
PVYDPDEVEEVTTSITEDETEEEEEKQREEE+EEEE+QEE+RPE
```

	1370 1380 1390 1400
T_theileri_Tth.11.2260/1-625	--
T_theileri_Tth.70.1030/1-768	--ANGK
T_theileri_Tth.107.1040/1-828	--
T_theileri_Tth.70.1110/1-944	------------------------------------AEELLSPL
T_theileri_Tth.26.2530/1-142	--
T_theileri_Tth.46.1090/1-145	--
T_theileri_Tth.24.1770/1-211	--
T_theileri_Tth.13.2480/1-341	--
T_theileri_Tth.6.5070/1-728	--
T_theileri_Tth.2.1490/1-336	--
T_theileri_Tth.136.1010/1-335	--
T_theileri_Tth.46.1100/1-749	--KR
T_theileri_Tth.83.1100/1-183	--
T_theileri_Tth.85.1040/1-640	--
T_theileri_Tth.36.2070/1-818	--
T_theileri_Tth.17.3250/1-783	--
T_theileri_Tth.38.2000/1-661	--
T_theileri_Tth.15.3170/1-626	--
T_theileri_Tth.36.2060/1-770	--
T_theileri_Tth.61.1040/1-155	--
T_theileri_Tth.121.1030/1-597	GASAVANQGSSAPDARPSAQPEGQQHPNPQQEVQEVEKEIKSQT
T_theileri_Tth.10.2550/1-638	--
T_theileri_Tth.54.1320/1-753	--
T_theileri_Tth.101.1070/1-104	--
T_theileri_Tth.2.4010/1-502	--
T_theileri_Tth.11.2100/1-425	--
T_theileri_Tth.25.1730/1-222	--
T_theileri_Tth.1.6340/1-550	--
T_theileri_Tth.2.4020/1-205	--
T_theileri_Tth.6.5050/1-642	--
T_theileri_Tth.121.1040/1-345	--
T_theileri_Tth.87.1070/1-161	--
T_theileri_Tth.10.2540/1-581	--
T_theileri_Tth.70.1130/1-603	--
T_theileri_Tth.125.1020/1-613	--
T_theileri_Tth.107.1020/1-785	--
T_theileri_Tth.24.1760/1-439	--
T_theileri_Tth.27.1380/1-325	--
T_theileri_Tth.44.1550/1-167	--
T_theileri_Tth.61.1080/1-163	--
T_theileri_Tth.85.1060/1-407	--
T_theileri_Tth.11.3490/1-239	--
Conservation	--
Quality	
Consensus	GASAVS+T+P+AV+A++S+IP++QQHP+E++EVQEV+KE+ESQK

```
                               1410      1420      1430      1440      1450
T_theileri_Tth.11.2260/1-625   --------------------------------------------
T_theileri_Tth.70.1030/1-768   AEEEEKSNTQSGLASQTQPQHHVPALA-----------------
T_theileri_Tth.107.1040/1-828  --------------------------------------------
T_theileri_Tth.70.1110/1-944   GSAARAVEENVESPVRPAPRAENGAQDSSGSKSGALPHKEPLVM
T_theileri_Tth.26.2530/1-142   --------------------------------------------
T_theileri_Tth.46.1090/1-145   --------------------------------------------
T_theileri_Tth.24.1770/1-211   --------------------------------------------
T_theileri_Tth.13.2480/1-341   --------------------------------------------
T_theileri_Tth.6.5070/1-728    --------------------------------------------
T_theileri_Tth.2.1490/1-336    --------------------------------------------
T_theileri_Tth.136.1010/1-335  --------------------------------------------
T_theileri_Tth.46.1100/1-749   KMEEKERQLREERSQVNNNSQQVPPKAKLQKKNHSD--------
T_theileri_Tth.83.1100/1-183   --------------------------------------------
T_theileri_Tth.85.1040/1-640   --------------------------------------------
T_theileri_Tth.36.2070/1-818   --------------------------------------------
T_theileri_Tth.17.3250/1-783   --------------------------------------------
T_theileri_Tth.38.2000/1-661   --------------------------------------------
T_theileri_Tth.15.3170/1-626   --------------------------------------------
T_theileri_Tth.36.2060/1-770   --------------------------------------------
T_theileri_Tth.61.1040/1-155   --------------------------------------------
T_theileri_Tth.121.1030/1-597  --------------------------------------------
T_theileri_Tth.10.2550/1-638   --------------------------------------------
T_theileri_Tth.54.1320/1-753   --------------------------------------------
T_theileri_Tth.101.1070/1-104  --------------------------------------------
T_theileri_Tth.2.4010/1-502    --------------------------------------------
T_theileri_Tth.11.2100/1-425   --------------------------------------------
T_theileri_Tth.25.1730/1-222   --------------------------------------------
T_theileri_Tth.1.6340/1-550    --------------------------------------------
T_theileri_Tth.2.4020/1-205    --------------------------------------------
T_theileri_Tth.6.5050/1-642    --------------------------------------------
T_theileri_Tth.121.1040/1-345  --------------------------------------------
T_theileri_Tth.87.1070/1-161   --------------------------------------------
T_theileri_Tth.10.2540/1-581   --------------------------------------------
T_theileri_Tth.70.1130/1-603   --------------------------------------------
T_theileri_Tth.125.1020/1-613  --------------------------------------------
T_theileri_Tth.107.1020/1-785  --------------------------------------------
T_theileri_Tth.24.1760/1-439   --------------------------------------------
T_theileri_Tth.27.1380/1-325   --------------------------------------------
T_theileri_Tth.44.1550/1-167   --------------------------------------------
T_theileri_Tth.61.1080/1-163   --------------------------------------------
T_theileri_Tth.85.1060/1-407   --------------------------------------------
T_theileri_Tth.11.3490/1-239   --------------------------------------------

Conservation                   --------------------------------------------

Quality

Consensus                      AE+EEE+Q+++ELASQP+PS+++PALAA-Q+K-QPNPRENPTS-
```

```
                              1460      1470      1480      1490
T_theileri_Tth.11.2260/1-625  --------------------------------------------
T_theileri_Tth.70.1030/1-768  -------------------------------------VDQRGSA
T_theileri_Tth.107.1040/1-828 --------------------------------------------
T_theileri_Tth.70.1110/1-944  EEEKKQKVEQQGQQSPRLQSI----------------PKEEPSA
T_theileri_Tth.26.2530/1-142  --------------------------------------------
T_theileri_Tth.46.1090/1-145  --------------------------------------------
T_theileri_Tth.24.1770/1-211  --------------------------------------------
T_theileri_Tth.13.2480/1-341  --------------------------------------------
T_theileri_Tth.6.5070/1-728   ------------------------------------AEVQSNSS
T_theileri_Tth.2.1490/1-336   --------------------------------------------
T_theileri_Tth.136.1010/1-335 --------------------------------------------
T_theileri_Tth.46.1100/1-749  --------------------------------------------
T_theileri_Tth.83.1100/1-183  --------------------------------------------
T_theileri_Tth.85.1040/1-640  --------------------------------------------
T_theileri_Tth.36.2070/1-818  --------------------------------------------
T_theileri_Tth.17.3250/1-783  ------------------------------------NRVQNSDS
T_theileri_Tth.38.2000/1-661  --------------------------------------------
T_theileri_Tth.15.3170/1-626  ------------------------------------TEEQSSSS
T_theileri_Tth.36.2060/1-770  --------------------------------------------
T_theileri_Tth.61.1040/1-155  --------------------------------------------
T_theileri_Tth.121.1030/1-597 ------------------------------------LSLKHPES
T_theileri_Tth.10.2550/1-638  --------------------------------------------
T_theileri_Tth.54.1320/1-753  --------------------------------------------
T_theileri_Tth.101.1070/1-104 --------------------------------------------
T_theileri_Tth.2.4010/1-502   --------------------------------------------
T_theileri_Tth.11.2100/1-425  --------------------------------------------
T_theileri_Tth.25.1730/1-222  --------------------------------------------
T_theileri_Tth.1.6340/1-550   --------------------------------------------
T_theileri_Tth.2.4020/1-205   --------------------------------------------
T_theileri_Tth.6.5050/1-642   ------------------------------------TKDQNNSS
T_theileri_Tth.121.1040/1-345 --------------------------------------------
T_theileri_Tth.87.1070/1-161  --------------------------------------------
T_theileri_Tth.10.2540/1-581  ------------------------------------ANKLSNSS
T_theileri_Tth.70.1130/1-603  --------------------------------------------
T_theileri_Tth.125.1020/1-613 --------------------------------------------
T_theileri_Tth.107.1020/1-785 --------------------------------------------
T_theileri_Tth.24.1760/1-439  --------------------------------------------
T_theileri_Tth.27.1380/1-325  --------------------------------------------
T_theileri_Tth.44.1550/1-167  --------------------------------------------
T_theileri_Tth.61.1080/1-163  --------------------------------------------
T_theileri_Tth.85.1060/1-407  --------------------------------------------
T_theileri_Tth.11.3490/1-239  --------------------------------------------
```

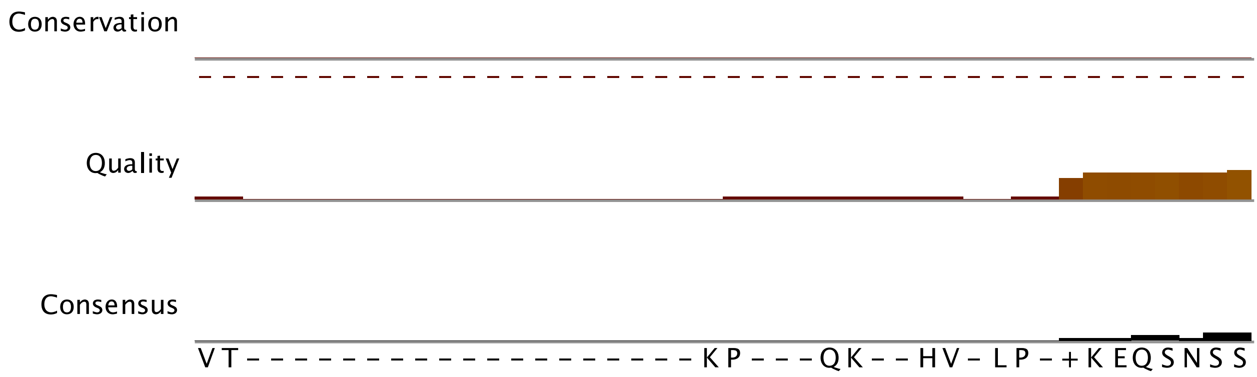

```
                                   1500      1510      1520      1530
T_theileri_Tth.11.2260/1-625   --------------------------------------------
T_theileri_Tth.70.1030/1-768   VADKENVESHVSPASLQARDQRGSS-AEGEKEVDLHPPAVPELQ
T_theileri_Tth.107.1040/1-828  --------------------------------------------
T_theileri_Tth.70.1110/1-944   RSDVLPKNEKDSHETSKVRNQSGAPGVNEQDVATNVRPSS----
T_theileri_Tth.26.2530/1-142   --------------------------------------------
T_theileri_Tth.46.1090/1-145   --------------------------------------------
T_theileri_Tth.24.1770/1-211   --------------------------------------------
T_theileri_Tth.13.2480/1-341   --------------------------------------------
T_theileri_Tth.6.5070/1-728    TNPEVSGGV-ANAGNETP-----------------------TT-
T_theileri_Tth.2.1490/1-336    --------------------------------------------
T_theileri_Tth.136.1010/1-335  --------------------------------------------
T_theileri_Tth.46.1100/1-749   -------------------------------------PVKPQEG
T_theileri_Tth.83.1100/1-183   --------------------------------------------
T_theileri_Tth.85.1040/1-640   --------------------------------------------
T_theileri_Tth.36.2070/1-818   --------------------------------------------
T_theileri_Tth.17.3250/1-783   SSDAVKKDGGGVPAVVLPTPNTVGQQEAKNQVVSKATSQKNRE-
T_theileri_Tth.38.2000/1-661   --------------------------------------------
T_theileri_Tth.15.3170/1-626   AESEM------NSGTGDQ-----------------------DQ-
T_theileri_Tth.36.2060/1-770   --------------------------------------------
T_theileri_Tth.61.1040/1-155   --------------------------------------------
T_theileri_Tth.121.1030/1-597  ENAEHTGSSSVTPGSNTGSGGNNNDNGSNEDQNMGQARESASTR
T_theileri_Tth.10.2550/1-638   --------------------------------------------
T_theileri_Tth.54.1320/1-753   --------------------------------------------
T_theileri_Tth.101.1070/1-104  --------------------------------------------
T_theileri_Tth.2.4010/1-502    --------------------------------------------
T_theileri_Tth.11.2100/1-425   --------------------------------------------
T_theileri_Tth.25.1730/1-222   --------------------------------------------
T_theileri_Tth.1.6340/1-550    --------------------------------------------
T_theileri_Tth.2.4020/1-205    --------------------------------------------
T_theileri_Tth.6.5050/1-642    TNSEVSGDV-ANTEGETS-----------------------TN-
T_theileri_Tth.121.1040/1-345  --------------------------------------------
T_theileri_Tth.87.1070/1-161   --------------------------------------------
T_theileri_Tth.10.2540/1-581   AESKPNNGAGTDTRVETS-----------------------SN-
T_theileri_Tth.70.1130/1-603   --------------------------------------------
T_theileri_Tth.125.1020/1-613  --------------------------------------------
T_theileri_Tth.107.1020/1-785  --------------------------------------------
T_theileri_Tth.24.1760/1-439   --------------------------------------------
T_theileri_Tth.27.1380/1-325   --------------------------------------------
T_theileri_Tth.44.1550/1-167   --------------------------------------------
T_theileri_Tth.61.1080/1-163   --------------------------------------------
T_theileri_Tth.85.1060/1-407   --------------------------------------------
T_theileri_Tth.11.3490/1-239   --------------------------------------------

Conservation                   --------------------------------------------

Quality

Consensus                      TNSE+NGDSHVNTASETSSNQRDSP+GEGE+EVV+PSPASPTNQ
```

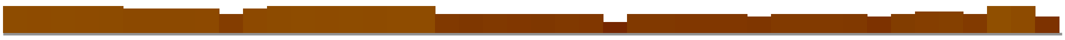

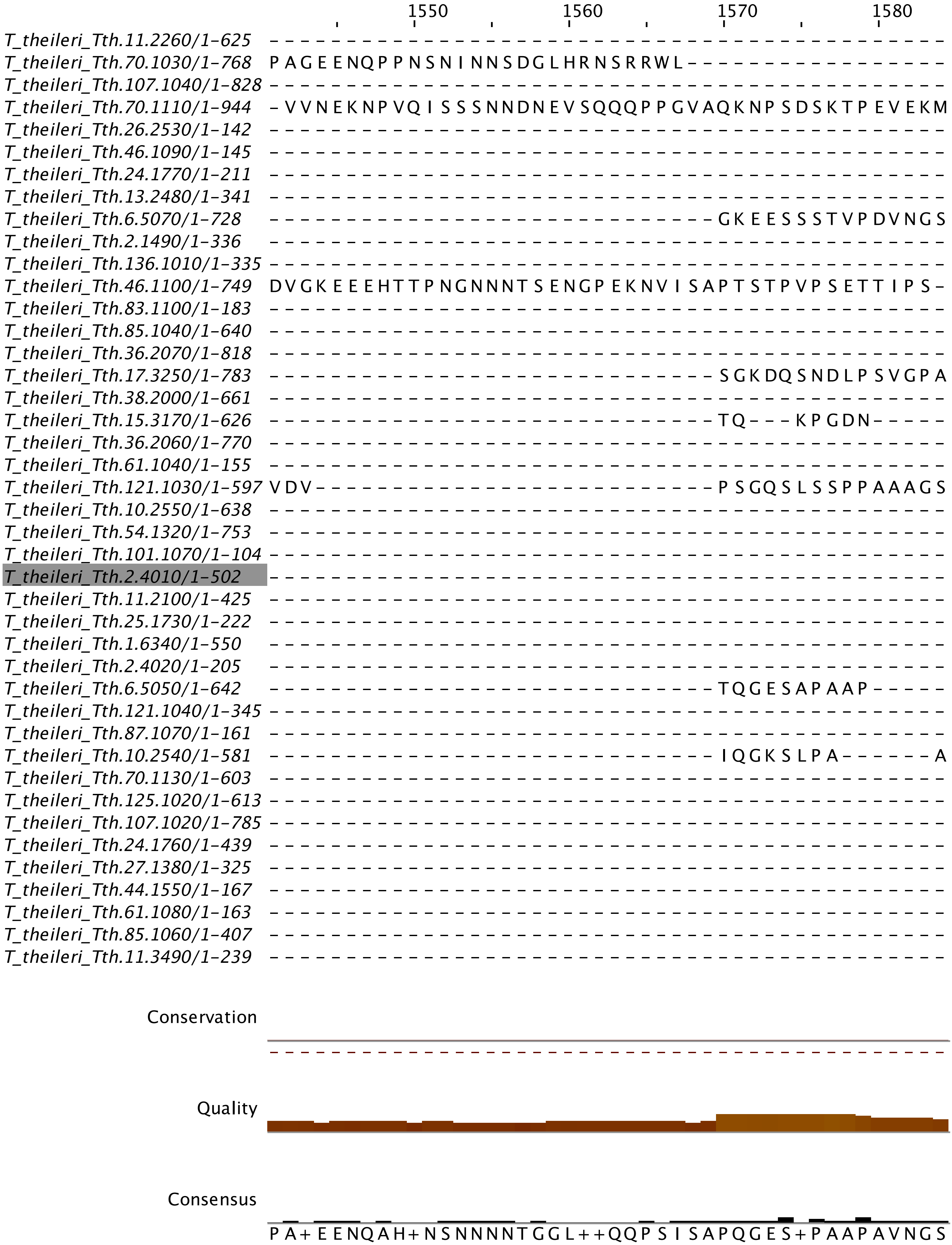
1550 1560 1570 1580
T_theileri_Tth.11.2260/1-625 --
T_theileri_Tth.70.1030/1-768 PAGEENQPPNSNINNSDGLHRNSRRWL-----------------
T_theileri_Tth.107.1040/1-828 --
T_theileri_Tth.70.1110/1-944 -VVNEKNPVQISSSNNDNEVSQQQPPGVAQKNPSDSKTPEVEKM
T_theileri_Tth.26.2530/1-142 --
T_theileri_Tth.46.1090/1-145 --
T_theileri_Tth.24.1770/1-211 --
T_theileri_Tth.13.2480/1-341 --
T_theileri_Tth.6.5070/1-728 -----------------------------GKEESSSTVPDVNGS
T_theileri_Tth.2.1490/1-336 --
T_theileri_Tth.136.1010/1-335 --
T_theileri_Tth.46.1100/1-749 DVGKEEEHTTPNGNNNTSENGPEKNVISAPTSTPVPSETTIPS-
T_theileri_Tth.83.1100/1-183 --
T_theileri_Tth.85.1040/1-640 --
T_theileri_Tth.36.2070/1-818 --
T_theileri_Tth.17.3250/1-783 -----------------------------SGKDQSNDLPSVGPA
T_theileri_Tth.38.2000/1-661 --
T_theileri_Tth.15.3170/1-626 -----------------------------TQ---KPGDN-----
T_theileri_Tth.36.2060/1-770 --
T_theileri_Tth.61.1040/1-155 --
T_theileri_Tth.121.1030/1-597 VDV--------------------------PSGQSLSSPPAAAGS
T_theileri_Tth.10.2550/1-638 --
T_theileri_Tth.54.1320/1-753 --
T_theileri_Tth.101.1070/1-104 --
T_theileri_Tth.2.4010/1-502 --
T_theileri_Tth.11.2100/1-425 --
T_theileri_Tth.25.1730/1-222 --
T_theileri_Tth.1.6340/1-550 --
T_theileri_Tth.2.4020/1-205 --
T_theileri_Tth.6.5050/1-642 -----------------------------TQGESAPAAP-----
T_theileri_Tth.121.1040/1-345 --
T_theileri_Tth.87.1070/1-161 --
T_theileri_Tth.10.2540/1-581 -----------------------------IQGKSLPA------A
T_theileri_Tth.70.1130/1-603 --
T_theileri_Tth.125.1020/1-613 --
T_theileri_Tth.107.1020/1-785 --
T_theileri_Tth.24.1760/1-439 --
T_theileri_Tth.27.1380/1-325 --
T_theileri_Tth.44.1550/1-167 --
T_theileri_Tth.61.1080/1-163 --
T_theileri_Tth.85.1060/1-407 --
T_theileri_Tth.11.3490/1-239 --
Conservation
Quality
Consensus
PA+EENQAH+NSNNNNTGGL++QQPSISAPQGES+PAAPAVNGS

```
                                     1590      1600      1610      1620
T_theileri_Tth.11.2260/1-625    --------------------------------------------
T_theileri_Tth.70.1030/1-768    --------------------------------------------
T_theileri_Tth.107.1040/1-828   --------------------------------------------
T_theileri_Tth.70.1110/1-944    PDSKI--------------------------TIPAPYGPSFEAP
T_theileri_Tth.26.2530/1-142    --------------------------------------------
T_theileri_Tth.46.1090/1-145    --------------------------------------------
T_theileri_Tth.24.1770/1-211    --------------------------------------------
T_theileri_Tth.13.2480/1-341    --------------------------------------------
T_theileri_Tth.6.5070/1-728     SQQPGT----------------ATQSVIQHTEKNSTNAESDGKE
T_theileri_Tth.2.1490/1-336     --------------------------------------------
T_theileri_Tth.136.1010/1-335   --------------------------------------------
T_theileri_Tth.46.1100/1-749    --------------------------------------------
T_theileri_Tth.83.1100/1-183    --------------------------------------------
T_theileri_Tth.85.1040/1-640    --------------------------------------------
T_theileri_Tth.36.2070/1-818    --------------------------------------------
T_theileri_Tth.17.3250/1-783    GSGIDS----------------AGSANNNAVGSNRGNASSGQQR
T_theileri_Tth.38.2000/1-661    --------------------------------------------
T_theileri_Tth.15.3170/1-626    --------------------------------------------
T_theileri_Tth.36.2060/1-770    --------------------------------------------
T_theileri_Tth.61.1040/1-155    --------------------------------------------
T_theileri_Tth.121.1030/1-597   AT--------------------IDPANTVTREISQQPADQPAQN
T_theileri_Tth.10.2550/1-638    --------------------------------------------
T_theileri_Tth.54.1320/1-753    --------------------------------------------
T_theileri_Tth.101.1070/1-104   --------------------------------------------
T_theileri_Tth.2.4010/1-502     --------------------------------------------
T_theileri_Tth.11.2100/1-425    --------------------------------------------
T_theileri_Tth.25.1730/1-222    --------------------------------------------
T_theileri_Tth.1.6340/1-550     --------------------------------------------
T_theileri_Tth.2.4020/1-205     --------------------------------------------
T_theileri_Tth.6.5050/1-642     --------------------------------------------
T_theileri_Tth.121.1040/1-345   --------------------------------------------
T_theileri_Tth.87.1070/1-161    --------------------------------------------
T_theileri_Tth.10.2540/1-581    SNQPST----------------TAQSATH-------------EE
T_theileri_Tth.70.1130/1-603    --------------------------------------------
T_theileri_Tth.125.1020/1-613   --------------------------------------------
T_theileri_Tth.107.1020/1-785   --------------------------------------------
T_theileri_Tth.24.1760/1-439    --------------------------------------------
T_theileri_Tth.27.1380/1-325    --------------------------------------------
T_theileri_Tth.44.1550/1-167    --------------------------------------------
T_theileri_Tth.61.1080/1-163    --------------------------------------------
T_theileri_Tth.85.1060/1-407    --------------------------------------------
T_theileri_Tth.11.3490/1-239    --------------------------------------------

Conservation                    --------------------------------------------

Quality
```

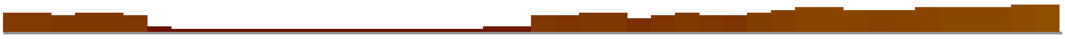

```
Consensus                       SSEPSAAQD+E+SIVE-TPPAPAPQS+T+AEEPS+QNAE+PEEN
```

```
                                   1630      1640      1650      1660      1670
T_theileri_Tth.11.2260/1-625   --------------------------------------------
T_theileri_Tth.70.1030/1-768   --------------------------------------------
T_theileri_Tth.107.1040/1-828  --------------------------------------------
T_theileri_Tth.70.1110/1-944   KPVVPESTVVTVEPTPSIKEDKKNNTAENTKQVHGTSSQSTSQG
T_theileri_Tth.26.2530/1-142   --------------------------------------------
T_theileri_Tth.46.1090/1-145   --------------------------------------------
T_theileri_Tth.24.1770/1-211   --------------------------------------------
T_theileri_Tth.13.2480/1-341   --------------------------------------------
T_theileri_Tth.6.5070/1-728    VQHASRS-------------------------------------
T_theileri_Tth.2.1490/1-336    --------------------------------------------
T_theileri_Tth.136.1010/1-335  --------------------------------------------
T_theileri_Tth.46.1100/1-749   --------------------------------------------
T_theileri_Tth.83.1100/1-183   --------------------------------------------
T_theileri_Tth.85.1040/1-640   --------------------------------------------
T_theileri_Tth.36.2070/1-818   --------------------------------------------
T_theileri_Tth.17.3250/1-783   MGKIKES-------------------------------------
T_theileri_Tth.38.2000/1-661   --------------------------------------------
T_theileri_Tth.15.3170/1-626   --------------------------------------------
T_theileri_Tth.36.2060/1-770   --------------------------------------------
T_theileri_Tth.61.1040/1-155   --------------------------------------------
T_theileri_Tth.121.1030/1-597  NDKQNENETPAEAQD--------------------------HQQ
T_theileri_Tth.10.2550/1-638   --------------------------------------------
T_theileri_Tth.54.1320/1-753   --------------------------------------------
T_theileri_Tth.101.1070/1-104  --------------------------------------------
T_theileri_Tth.2.4010/1-502    --------------------------------------------
T_theileri_Tth.11.2100/1-425   --------------------------------------------
T_theileri_Tth.25.1730/1-222   --------------------------------------------
T_theileri_Tth.1.6340/1-550    --------------------------------------------
T_theileri_Tth.2.4020/1-205    --------------------------------------------
T_theileri_Tth.6.5050/1-642    --------------------------------------------
T_theileri_Tth.121.1040/1-345  --------------------------------------------
T_theileri_Tth.87.1070/1-161   --------------------------------------------
T_theileri_Tth.10.2540/1-581   GR---GS-------------------------------------
T_theileri_Tth.70.1130/1-603   --------------------------------------------
T_theileri_Tth.125.1020/1-613  --------------------------------------------
T_theileri_Tth.107.1020/1-785  --------------------------------------------
T_theileri_Tth.24.1760/1-439   --------------------------------------------
T_theileri_Tth.27.1380/1-325   --------------------------------------------
T_theileri_Tth.44.1550/1-167   --------------------------------------------
T_theileri_Tth.61.1080/1-163   --------------------------------------------
T_theileri_Tth.85.1060/1-407   --------------------------------------------
T_theileri_Tth.11.3490/1-239   --------------------------------------------

                  Conservation
                               --------------------------------------------
                       Quality
```

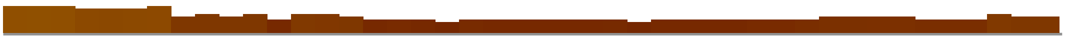

```
                     Consensus
                               K++Q+ESITVS+AQD+SIKEEDKNNTTENIEQVQ+TSTQSPSQN
```

```
                                 1680      1690      1700      1710
T_theileri_Tth.11.2260/1-625   --------------------------------------------
T_theileri_Tth.70.1030/1-768   --------------------------------------------
T_theileri_Tth.107.1040/1-828  -----------------------------------LSDGTQPVT
T_theileri_Tth.70.1110/1-944   GKNDERNTTVDPTHHP---NSTSPDNEKNNETIVNTDNKTTTST
T_theileri_Tth.26.2530/1-142   --------------------------------------------
T_theileri_Tth.46.1090/1-145   --------------------------------------------
T_theileri_Tth.24.1770/1-211   --------------------------------------------
T_theileri_Tth.13.2480/1-341   --------------------------------------------
T_theileri_Tth.6.5070/1-728    ---------------RRHIADAT----------AS---------
T_theileri_Tth.2.1490/1-336    --------------------------------------------
T_theileri_Tth.136.1010/1-335  --------------------------------------------
T_theileri_Tth.46.1100/1-749   --------------------------------------------
T_theileri_Tth.83.1100/1-183   --------------------------------------------
T_theileri_Tth.85.1040/1-640   -----------------------------------------ERT
T_theileri_Tth.36.2070/1-818   -----------------------------------LSEGTQPAT
T_theileri_Tth.17.3250/1-783   -ESTVIDAEVQPGLGSSSSLPAA----------DA---------
T_theileri_Tth.38.2000/1-661   -------------------------TPSDLPSPTAVSDTQGLPT
T_theileri_Tth.15.3170/1-626   --------------------------------------------
T_theileri_Tth.36.2060/1-770   ------------------------------------------VT
T_theileri_Tth.61.1040/1-155   --------------------------------------------
T_theileri_Tth.121.1030/1-597  VPNQTENKVQSPTKANAQTDDSTDVQNNTHGEESTQRNRHDTAT
T_theileri_Tth.10.2550/1-638   -------------------------TASALPSSTAVSDIQSRPT
T_theileri_Tth.54.1320/1-753   --------------------------------------------
T_theileri_Tth.101.1070/1-104  --------------------------------------------
T_theileri_Tth.2.4010/1-502    --------------------------------------------
T_theileri_Tth.11.2100/1-425   --------------------------------------------
T_theileri_Tth.25.1730/1-222   --------------------------------------------
T_theileri_Tth.1.6340/1-550    --------------------------------------------
T_theileri_Tth.2.4020/1-205    ----------------------------------SGAGTRQSSV
T_theileri_Tth.6.5050/1-642    --------------------------------------------
T_theileri_Tth.121.1040/1-345  --------------------------------------------
T_theileri_Tth.87.1070/1-161   --------------------------------------------
T_theileri_Tth.10.2540/1-581   ---------------RNHHDADT----------TS---------
T_theileri_Tth.70.1130/1-603   --------------------------------------------
T_theileri_Tth.125.1020/1-613  ------------------HQEQGETHHTESGHTNNNSSSVPSQS
T_theileri_Tth.107.1020/1-785  ------------------------------------------AA
T_theileri_Tth.24.1760/1-439   --------------------------------------------
T_theileri_Tth.27.1380/1-325   --------------------------------------------
T_theileri_Tth.44.1550/1-167   --------------------------------------------
T_theileri_Tth.61.1080/1-163   --------------------------------------------
T_theileri_Tth.85.1060/1-407   --------------------------------------------
T_theileri_Tth.11.3490/1-239   --------------------------------------------
```

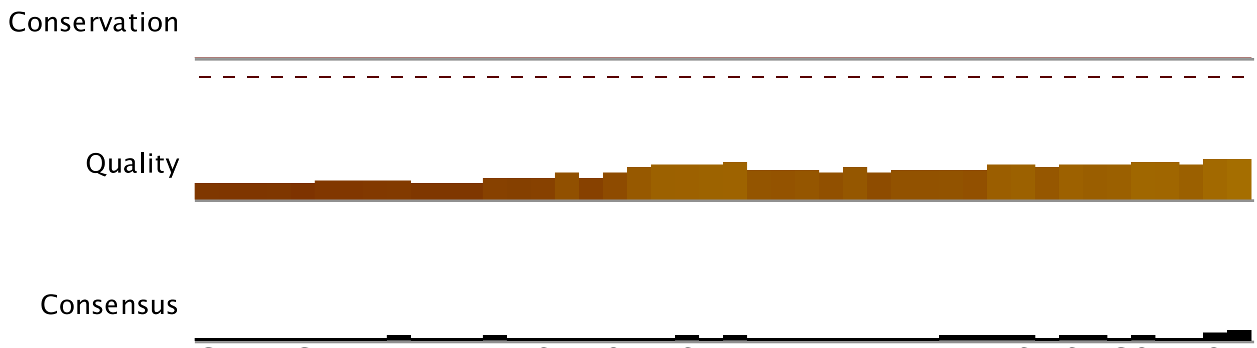


```
Consensus                      GNDEGINTTVDPTESP+QT+SPTT+NE+PNEETVS+SDGQ+PQT
```

```
                               1720      1730      1740      1750
T_theileri_Tth.11.2260/1-625   ------------------------------------------SV
T_theileri_Tth.70.1030/1-768   --------------------------------------------
T_theileri_Tth.107.1040/1-828  EATGTARP---------SE------SAAGSEEVTAPVVQNGVST
T_theileri_Tth.70.1110/1-944   DPVENAQTETS---VTGTPSPTDSNNTDGHNINSISATSPVNEK
T_theileri_Tth.26.2530/1-142   --------------------------------------------
T_theileri_Tth.46.1090/1-145   --------------------------------------------
T_theileri_Tth.24.1770/1-211   --------------------------------------------
T_theileri_Tth.13.2480/1-341   --------------------------------------------
T_theileri_Tth.6.5070/1-728    --QSTSNPNGTED-PIQSPNTV---------------LNGTTIT
T_theileri_Tth.2.1490/1-336    --------------------------------------------
T_theileri_Tth.136.1010/1-335  --------------------------------------------
T_theileri_Tth.46.1100/1-749   ---------------------------SENNINTTTSSIPENVI
T_theileri_Tth.83.1100/1-183   --------------------------------------------
T_theileri_Tth.85.1040/1-640   GSSQPPRDVPRETEGEMPSLIDDVNSHSVSNGDVTAASTGVDDS
T_theileri_Tth.36.2070/1-818   EATETPKP---------SESAGTSNN-AGNQGVSVPVVQNEAST
T_theileri_Tth.17.3250/1-783   --PSTSNPNGNDN-TVQSTNTINAADGRSSGTGNNGALNGTKLT
T_theileri_Tth.38.2000/1-661   NTEDSDQRTTTTPTTAEPNNAHTTGSSTTDSTTPTATVNNNGKT
T_theileri_Tth.15.3170/1-626   -----------ED-G-----------------------------
T_theileri_Tth.36.2060/1-770   EATGTARP---------SE------S-DGNQEVTAPVVQNEAST
T_theileri_Tth.61.1040/1-155   --------------------------------------------
T_theileri_Tth.121.1030/1-597  TFSSAANSNGIES-TIQSPHTA-----------NNGVLNGTNLT
T_theileri_Tth.10.2550/1-638   NTQDNNHRTTTTPTTAVPNNAH---------------------T
T_theileri_Tth.54.1320/1-753   -ATGTARP---------TE------N-DGNQEVTAPVVQNEAST
T_theileri_Tth.101.1070/1-104  --------------------------------------------
T_theileri_Tth.2.4010/1-502    --------------------------------------------
T_theileri_Tth.11.2100/1-425   --------------------------------------------
T_theileri_Tth.25.1730/1-222   --------------------------------------------
T_theileri_Tth.1.6340/1-550    --------------------------------------------
T_theileri_Tth.2.4020/1-205    MVSKNRTTRGRTDVAAN---------------------------
T_theileri_Tth.6.5050/1-642    -----------ED-AIQSPNTV---------------LNGTNLT
T_theileri_Tth.121.1040/1-345  --------------------------------------------
T_theileri_Tth.87.1070/1-161   --------------------------------------------
T_theileri_Tth.10.2540/1-581   --QSSSNPNGKK------------------------------LT
T_theileri_Tth.70.1130/1-603   ---------------------------------------GEENK
T_theileri_Tth.125.1020/1-613  AERTQDTTGVNVST----------------------------NE
T_theileri_Tth.107.1020/1-785  EATGTPKP---------TE------SAAGNQGVSAPVVQNEAST
T_theileri_Tth.24.1760/1-439   --------------------------------------------
T_theileri_Tth.27.1380/1-325   --------------------------------------------
T_theileri_Tth.44.1550/1-167   --------------------------------------------
T_theileri_Tth.61.1080/1-163   --------------------------------------------
T_theileri_Tth.85.1060/1-407   --------------------------------------------
T_theileri_Tth.11.3490/1-239   --------------------------------------------
```

Conservation

Quality

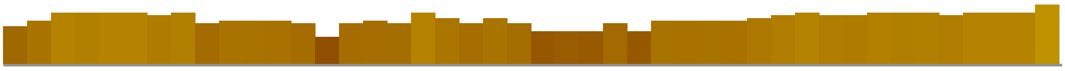

Consensus

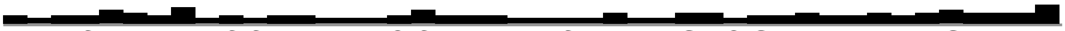

```
EATSTANPNSSEDTTTQSENTVTSNNTAGNQGVTTTVVNGTNLT
```

```
                                                     1770      1780      1790      1800
T_theileri_Tth.11.2260/1-625      NHIVVPRSTAVGSHDQGALVEGKKDIAHRVGMNGNSAAHSLLHA
T_theileri_Tth.70.1030/1-768      ----HKRSPVIFNRNSLLYLHKRIKVVPITLKWKSCPTLTTPSL
T_theileri_Tth.107.1040/1-828     TMEANRSETKEESITSFLNGQNTNVTLKKGIDGSFKV-SSVASV
T_theileri_Tth.70.1110/1-944      TAAADPATLTGLN-TQMGQVMNHTNAFAVVGADSSIATSYQITL
T_theileri_Tth.26.2530/1-142      --------------------------------------------
T_theileri_Tth.46.1090/1-145      --------------------------------------------
T_theileri_Tth.24.1770/1-211      --------------------------------------------
T_theileri_Tth.13.2480/1-341      --------------------------------------------
T_theileri_Tth.6.5070/1-728       ERQIKEETLKHTNVTVMFG-----------TDSSTVV-TYMAPL
T_theileri_Tth.2.1490/1-336       --------------------------------------------
T_theileri_Tth.136.1010/1-335     --------------------------------------------
T_theileri_Tth.46.1100/1-749      AAA-APATVAELNNTQMGQALNQATAIVIVGADSSISASYQIPL
T_theileri_Tth.83.1100/1-183      --------------------------------------------
T_theileri_Tth.85.1040/1-640      PLGSVPSPSTPTASTTVPVLLEEKTLGVRHPVDGVSALSGFVHV
T_theileri_Tth.36.2070/1-818      TKESKPSETKEENITSSVSGQNNKIVAGKGIDGSFKA-SAFASV
T_theileri_Tth.17.3250/1-783      EDKMKKETPNHTNIMGMLG-----------PDSSIMV-SYMAPL
T_theileri_Tth.38.2000/1-661      TGSSTTDSTTPQVSVDQKNNTMNIEIVSWPGKDQSQATKVYPPL
T_theileri_Tth.15.3170/1-626      ------------------A-----------TDNSIAL-SFFGPL
T_theileri_Tth.36.2060/1-770      ATESNRSETKEESNTSSVSGQNNNIVAGKGIDGSFKA-SVFASV
T_theileri_Tth.61.1040/1-155      --------------------------------------------
T_theileri_Tth.121.1030/1-597     EDQIKEETLKHTNVTGMLG-----------PDSSIMV-SYMAPL
T_theileri_Tth.10.2550/1-638      TGSSITDSTTPQGSINQKNNTMNIEIVSRVGNDHSEVTKVYTPL
T_theileri_Tth.54.1320/1-753      TTEANRSETKEENNTSSVSGQNNNATAKKGPDGSFKA-SSFASV
T_theileri_Tth.101.1070/1-104     --------------------------------------------
T_theileri_Tth.2.4010/1-502       --------------------------------------------
T_theileri_Tth.11.2100/1-425      --------------------------------------------
T_theileri_Tth.25.1730/1-222      --------------------------------------------
T_theileri_Tth.1.6340/1-550       --------------------------------DGCVAPALLAPV
T_theileri_Tth.2.4020/1-205       --------------------------------------------
T_theileri_Tth.6.5050/1-642       ESQMEEES-KNANGTDVLG-----------TDSSTVA-SYXRS-
T_theileri_Tth.121.1040/1-345     --------------------------------------------
T_theileri_Tth.87.1070/1-161      --------------------------------------------
T_theileri_Tth.10.2540/1-581      EDQMK-ETLNHTN--DVRR-----------TDSSIMF-SYIAPL
T_theileri_Tth.70.1130/1-603      TANNNNMSNHTVVGNITCTVGNCNKHTAGEGANKSIVTSYFGAV
T_theileri_Tth.125.1020/1-613     NVTVPRTTEGNINKDNLTSPRVKEELKIRMG-------------
T_theileri_Tth.107.1020/1-785     TTESKPSETKEENNTLSASGQNNKTTAKKGTDGSFKA-SADASV
T_theileri_Tth.24.1760/1-439      --------------------------------------------
T_theileri_Tth.27.1380/1-325      --------------------------------------------
T_theileri_Tth.44.1550/1-167      --------------------------------------------
T_theileri_Tth.61.1080/1-163      --------------------------------------------
T_theileri_Tth.85.1060/1-407      --------------------------------------------
T_theileri_Tth.11.3490/1-239      -----------------------------------LSALVPLAM

Conservation                      --------------------------------------------
```

Quality

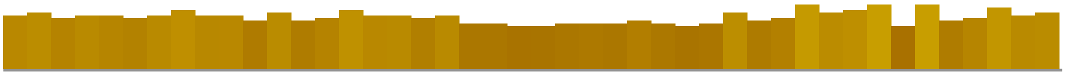

Consensus

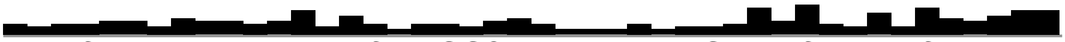

```
                                  TTESKPETLKETNVTSMLGGQNNNTIA+KGTDDSIKATSYFAPL
```

```
                              1810      1820      1830      1840
T_theileri_Tth.11.2260/1-625  LLLAVTLVVVVSL-------------------------------
T_theileri_Tth.70.1030/1-768  SHLNPLLFRGTFPPEWRRIRTIQLKAMNKCKEHYYSLLLKI---
T_theileri_Tth.107.1040/1-828 MFVFLTLSVIMLP-------------------------------
T_theileri_Tth.70.1110/1-944  LLLLCALVALASP-------------------------------
T_theileri_Tth.26.2530/1-142  --------------------------------------------
T_theileri_Tth.46.1090/1-145  --------------------------------------------
T_theileri_Tth.24.1770/1-211  --------------------------------------------
T_theileri_Tth.13.2480/1-341  --------------------------------------------
T_theileri_Tth.6.5070/1-728   ALLVCVVGFVMVP-------------------------------
T_theileri_Tth.2.1490/1-336   --------------------------------------------
T_theileri_Tth.136.1010/1-335 --------------------------------------------
T_theileri_Tth.46.1100/1-749  LLLLSALVTLASP-------------------------------
T_theileri_Tth.83.1100/1-183  --------------------------------------------
T_theileri_Tth.85.1040/1-640  AFLLMALAAAVPL-------------------------------
T_theileri_Tth.36.2070/1-818  MFLFLTLSVIMLP-------------------------------
T_theileri_Tth.17.3250/1-783  ALLVCVVGFVMVP-------------------------------
T_theileri_Tth.38.2000/1-661  VLLVLFVLTLLISF------------------------------
T_theileri_Tth.15.3170/1-626  MLPVCIVAAVVAL-------------------------------
T_theileri_Tth.36.2060/1-770  MFVFLTLSVIMAP-------------------------------
T_theileri_Tth.61.1040/1-155  --------------------------------------------
T_theileri_Tth.121.1030/1-597 ALLVCVVGFMMVP-------------------------------
T_theileri_Tth.10.2550/1-638  VLLVLVVSTLLISF------------------------------
T_theileri_Tth.54.1320/1-753  MFVFLTLSVIMLP-------------------------------
T_theileri_Tth.101.1070/1-104 --------------------------------------------
T_theileri_Tth.2.4010/1-502   --------------------------------------------
T_theileri_Tth.11.2100/1-425  --------------------------------------------
T_theileri_Tth.25.1730/1-222  --------------------------------------------
T_theileri_Tth.1.6340/1-550   VLFVMTLALSLLF-------------------------------
T_theileri_Tth.2.4020/1-205   --------------------------------------------
T_theileri_Tth.6.5050/1-642   --------------------------------------------
T_theileri_Tth.121.1040/1-345 --------------------------------------------
T_theileri_Tth.87.1070/1-161  --------------------------------------------
T_theileri_Tth.10.2540/1-581  ALLVGVVGFVMVP-------------------------------
T_theileri_Tth.70.1130/1-603  LLLLFIAAVMM---------------------------------
T_theileri_Tth.125.1020/1-613 --------------------------------------------
T_theileri_Tth.107.1020/1-785 MFVFLTLSMIMLP-------------------------------
T_theileri_Tth.24.1760/1-439  --------------------------------------------
T_theileri_Tth.27.1380/1-325  --------------------------------------------
T_theileri_Tth.44.1550/1-167  --------------------------------------------
T_theileri_Tth.61.1080/1-163  --------------------------------------------
T_theileri_Tth.85.1060/1-407  --------------------------------------------
T_theileri_Tth.11.3490/1-239  TFTLMGLL------------------------------------
```

Conservation

Quality

Consensus

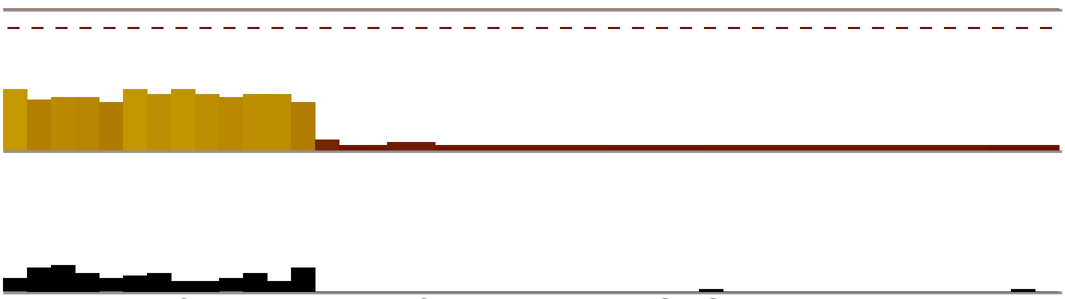

```
MLLVLVLSVVMVPFLIDSDTKNNLATNGKGPAAHV+NN+D+ITL
```

```
                                  1850      1860      1870
T_theileri_Tth.11.2260/1-625    --------------------------------
T_theileri_Tth.70.1030/1-768    --------------------------------
T_theileri_Tth.107.1040/1-828   --------------------------------
T_theileri_Tth.70.1110/1-944    --------------------------------
T_theileri_Tth.26.2530/1-142    --------------------------------
T_theileri_Tth.46.1090/1-145    --------------------------------
T_theileri_Tth.24.1770/1-211    --------------------------------
T_theileri_Tth.13.2480/1-341    --------------------------------
T_theileri_Tth.6.5070/1-728     --------------------------------
T_theileri_Tth.2.1490/1-336     --------------------------------
T_theileri_Tth.136.1010/1-335   --------------------------------
T_theileri_Tth.46.1100/1-749    --------------------------------
T_theileri_Tth.83.1100/1-183    --------------------------------
T_theileri_Tth.85.1040/1-640    --------------------------------
T_theileri_Tth.36.2070/1-818    --------------------------------
T_theileri_Tth.17.3250/1-783    --------------------------------
T_theileri_Tth.38.2000/1-661    --------------------------------
T_theileri_Tth.15.3170/1-626    --------------------------------
T_theileri_Tth.36.2060/1-770    --------------------------------
T_theileri_Tth.61.1040/1-155    --------------------------------
T_theileri_Tth.121.1030/1-597   --------------------------------
T_theileri_Tth.10.2550/1-638    --------------------------------
T_theileri_Tth.54.1320/1-753    --------------------------------
T_theileri_Tth.101.1070/1-104   --------------------------------
T_theileri_Tth.2.4010/1-502     --------------------------------
T_theileri_Tth.11.2100/1-425    --------------------------------
T_theileri_Tth.25.1730/1-222    --------------------------------
T_theileri_Tth.1.6340/1-550     --------------------------------
T_theileri_Tth.2.4020/1-205     --------------------------------
T_theileri_Tth.6.5050/1-642     --------------------------------
T_theileri_Tth.121.1040/1-345   --------------------------------
T_theileri_Tth.87.1070/1-161    --------------------------------
T_theileri_Tth.10.2540/1-581    --------------------------------
T_theileri_Tth.70.1130/1-603    --------------------------------
T_theileri_Tth.125.1020/1-613   --------------------------------
T_theileri_Tth.107.1020/1-785   --------------------------------
T_theileri_Tth.24.1760/1-439    --------------------------------
T_theileri_Tth.27.1380/1-325    --------------------------------
T_theileri_Tth.44.1550/1-167    --------------------------------
T_theileri_Tth.61.1080/1-163    --------------------------------
T_theileri_Tth.85.1060/1-407    --------------------------------
T_theileri_Tth.11.3490/1-239    --------------------------------

                   Conservation
                                --------------------------------

                        Quality

                      Consensus
                                NIGIGDGSVTPAGHAPLLLLILASAVAVVFPL
```
