# Supplementary figures and images for: An Alternative Strategy for Trypanosome Survival in the Mammalian Bloodstream Revealed through Genome and Transcriptome Analysis of the Ubiquitous Bovine Parasite Trypanosoma (Megatrypanum) theileri
Source: Genome Biol Evol. 2017 Aug 14;9(8):2093–109. doi: 10.1093/gbe/evx152 (PMC5737535; doi:10.1093/gbe/evx152)

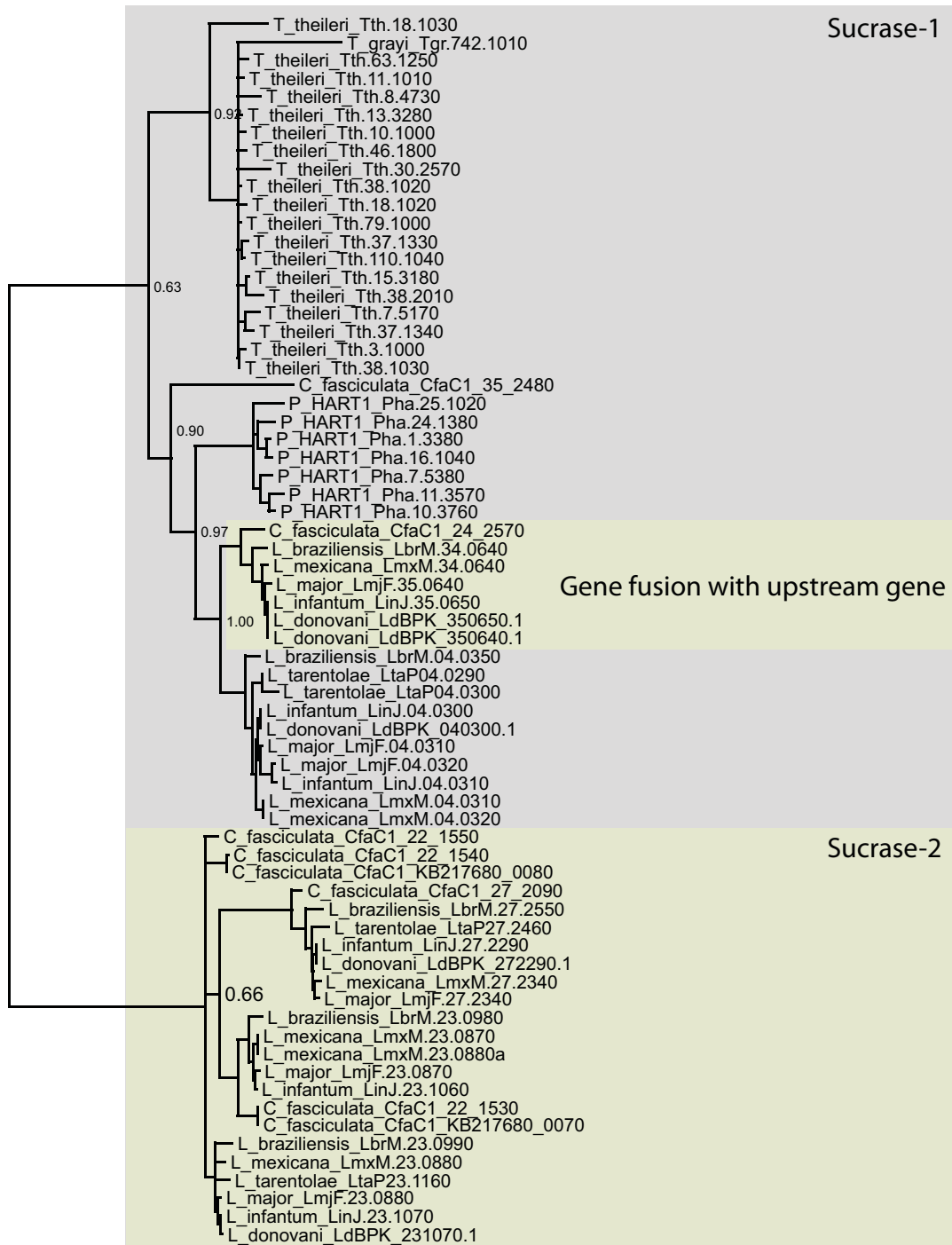

Supplement: Supplementary figure_7 [file evx152_suppfig_7.pdf]
