# Supplementary material for: An Alternative Strategy for Trypanosome Survival in the Mammalian Bloodstream Revealed through Genome and Transcriptome Analysis of the Ubiquitous Bovine Parasite Trypanosoma (Megatrypanum) theileri
Source: Genome Biol Evol. 2017 Aug 14;9(8):2093–109. doi: 10.1093/gbe/evx152 (PMC5737535; doi:10.1093/gbe/evx152)
Supplement: Supplementary materials [file evx152_suppfigurelegends.docx]

**An alternative strategy for trypanosome survival in the mammalian bloodstream revealed through genome and transcriptome analysis of the ubiquitous bovine parasite *Trypanosoma (Megatrypanum) theileri.***

Steven Kelly, Alasdair Ivens, G. Adam Mott, Ellis O’Neill, David Emms, Olivia Macleod, Paul Voorheis, Kevin Tyler, Matthew Clark, Jacqueline Matthews, Keith Matthews and Mark Carrington

**Supplementary Figure Legends**

Supplementary Figure 1

Representation of the genome sequence as the final set of scaffolds. The locations of open reading frames for abundant putative cell surface proteins, MSP and TTPSP1 to TTPSP4 families are shown coloured as in the key. Most are present in tandem arrays.

Supplementary Figure 2

Analysis of the 5’ and 3’ untranslated regions from mRNAs.

(a) The number of 5’ splice acceptor sites per gene plotted against the gene number. The insert shows a Logos analysis of conserved sequence around splice acceptor sites after alignment on the AG acceptor site.

(b) The variation of length of the 5’UTR plotted against gene number.

(c) The number of 3’ polyadenylation sites per gene plotted against the gene number. The insert shows a Logos analysis of conserved genomic sequence around polyadenylation sites after alignment on the first A of the poly A tail.

(d) The variation of length of the 3’UTR plotted against gene number.

Supplementary Figure 3

Conserved features of the four novel *T. theileri* gene families TTPSP1 to TTPSP4 encoding abundant putative cell surface proteins identified using Logos (Crooks, et al. 2004) after alignment of all members of the orthogroup.

(a) TTPSP1

(b) TTPSP2

(c) TTPSP3

(d) TTPSP4

Supplementary Figure 4

Homology model of Tth.6.1050 suggests a conserved three helical core.

Tth.6.1050 has a predicted N-terminal signal sequence and a GPI-anchor addition sequence and after these have been removed a predicted mature protein of 427 amino acids. The Phyre2 server (Kelley, et al. 2015) was used to predict the structure of Tth.6.1050. Truncating the protein at different positions yielded varying top hits. However, all high confidence models of Tth.6.1050 (>95% confidence) were modelled using 1) template structures with long alpha helices and 2) about the N-terminal half of Tth.6.1050. No structural homologues were found for the remaining residues towards the C-terminus. The highest confidence homology model for Tth.6.1050 was generated from the structure of the *Trypanosoma congolense* haptoglobin haemoglobin receptor (TcHpHbR; PDB ID 4E40) (99.9 % confidence, 12 % identity). The validity of the TcHpHbR structure as a template for Tth.6.1050 was tested further using Modeller (Sali and Blundell 1993). The alignment of Tth.6.1050 and TcHpHbR from Phyre2 was used to generate 100 homology models of Tth.6.1050 with Modeller.

a) The relative lengths of TcHpHbR and Tth.6.1050 are shown, including the region of TcHpHbR used to generate the model of Tth.6.1050 and the region of Tth.6.1050 that was not modelled.

b) The ‘best’ model of Tth.6.1050 had the lowest overall Discrete Optimized Protein Energy (DOPE) (Shen and Sali 2006) and is shown here alongside the TcHpHbR. The structures are coloured from the N-terminus (blue) to the C-terminus (red).

c) To investigate the quality of the alignment, the DOPE score for each residue of the ten ‘best’ models was calculated and plotted against the alignment position (red=best model, grey=models 2-10), as compared to the template TcHpHbR (black). Four regions of high DOPE are common to all models (yellow). Mapped on the ‘best’ Tth.6.1050 model (yellow), these regions localize to the N-terminal head region and gaps in the alignment. The alpha helical core region has lower DOPE scores and is better-aligned (red).

Crooks GE, Hon G, Chandonia JM, Brenner SE 2004. WebLogo: a sequence logo generator. Genome Res 14: 1188-1190. doi: 10.1101/gr.849004

Kelley LA, Mezulis S, Yates CM, Wass MN, Sternberg MJ 2015. The Phyre2 web portal for protein modeling, prediction and analysis. Nat Protoc 10: 845-858. doi: 10.1038/nprot.2015.053

Sali A, Blundell TL 1993. Comparative protein modelling by satisfaction of spatial restraints. J Mol Biol 234: 779-815. doi: 10.1006/jmbi.1993.1626

Shen MY, Sali A 2006. Statistical potential for assessment and prediction of protein structures. Protein Sci 15: 2507-2524. doi: 10.1110/ps.062416606

Supplementary Figure 5

Alignment of *T. theileri* trans-sialidase variants as both pdf and aln files. The active site motif GRW is located between residues 444 to 446 in this alignment

Supplementary Figure 6

Alignment of *T. theileri* MSP variants as both pdf and aln files. The active site motif HExxHxxGF is located between residues 629 to 638 in this alignment

Supplementary Figure 7

Bootstrap maximum likelihood phylogenetic tree of sucrase gene family. All *T. theileri* members of this family encode potential GPI-anchors at the C-terminus. Support values shown at key internal nodes. Nodes with less than 0.5 (50%) bootstrap support are collapsed.
